# Supplementary material for: BMAL2 is a druggable target for ovarian clear cell carcinoma (OCCC)
Source: EMBO Mol Med. 2026 Apr 3;18(5):1933–66. doi: 10.1038/s44321-026-00414-8 (PMC13179388; doi:10.1038/s44321-026-00414-8)
Supplement: Supplementary file 1 — Table EV1 [file 44321_2026_414_MOESM1_ESM.docx]

| **Table EV1. Short tandem repeat of OCCC cell lines, SOC cell lines and human fetal lung fibroblasts** | | | | | | | | | | |
| --- | --- | --- | --- | --- | --- | --- | --- | --- | --- | --- |
|  | | | | | | | | | | |
| **STR/Cell line** | **OVCA429** | **ES-2** | **JHOC5** | **OVISE** | **JHOC9** | **TOV21G** | **RMG1** | **KURAMOCHI** | **HEYA8** | **WI38** |
| **TH01** | 9,9 | 9.3, 9.3 | 7,7 | 9,9.3 | 6,9 | 7,9.3 | 11,11 | 9,9 | 8,9.3 | 8,9.3 |
| **D5S818** | 11,12 | 11,13 | 10,10 | 10,10 | 10,10 | 12,13 | 12,12 | 12,12 | 11,12 | 10,10 |
| **D13S317** | 7,12 | 11,11 | 9,12 | 11,12 | 10,10 | 11,12 | 12,12 | 9,12 | 11,11 | 11,11 |
| **D7820** | 11,12 | 11,11 | 12,12 | 11,12 | 8,12 | 12,12 | 11,11 | 10,11 | 12,12 | 9,11 |
| **D165539** | 12,12 | 11,13 | 11,13 | 9,9 | 12,12 | 10,12 | 9,10 | 10,10 | 8,12 | 11,12 |
| **CSF1PO** | 12,13 | 10,15 | 10,12 | 9,11 | 10,13 | 13,15 | 10,10 | 11,12 | 10,11 | 10,12 |
| **Amelogenin** | X,X | X,X | X,X | X,X | X,X | X,X | X,X | X,X | X,X | X,X |
| **vWA** | 16,18 | 16,17 | 14,16 | 18,18 | 14,17 | 17,17 | 17,18 | 16,19 | 16,17 | 19,20 |
| **TPOX** | 9,11 | 8,12 | 11,11 | 8,8 | 8,11 | 8,11 | 11,11 | 8,12 | 11,11 | 8,8 |
| **Match to Test Sample** | 100% | 100% | 100% | 100% | 96% | 100% | 95.65% | 100% | 96% | 100% |
| **Database** | ExPASy | ExPASy | ExPASy | ExPASy | ExPASy | ExPASy | ExPASy | ExPASy | ExPASy | ExPASy |
