# Supplementary material for: BMAL2 is a druggable target for ovarian clear cell carcinoma (OCCC)
Source: EMBO Mol Med. 2026 Apr 3;18(5):1933–66. doi: 10.1038/s44321-026-00414-8 (PMC13179388; doi:10.1038/s44321-026-00414-8)
Supplement: Supplementary file 2 — Table EV2 [file 44321_2026_414_MOESM2_ESM.docx]

| **Table EV2. Primers for qRT-PCR** | | |
| --- | --- | --- |
| **Gene** | **Forward (5’- 3’)** | **Reverse (5’- 3’)** |
| RNA18S5 | gtaacccgttgaaccccatt | ccatccaatcggtagtagcg |
| BMAL2 | agctgttggtcttgtccctg | caagtggctcctgcgatg |
| RAD51 | tctctggcagtgatgtcctgga | taaagggcggtggcactgtcta |
