# Supplementary material for: BMAL2 is a druggable target for ovarian clear cell carcinoma (OCCC)
Source: EMBO Mol Med. 2026 Apr 3;18(5):1933–66. doi: 10.1038/s44321-026-00414-8 (PMC13179388; doi:10.1038/s44321-026-00414-8)
Supplement: Supplementary file 3 — Table EV3 [file 44321_2026_414_MOESM3_ESM.docx]

| **Table EV3. Primers for ChIP-qPCR** | | |
| --- | --- | --- |
| **Promoter site** | **Forward (5’- 3’)** | **Reverse (5’- 3’)** |
| E-box 1 | cgattctcatgcctcagcct | ataaacctggccaacgtggt |
| E-box 2 | ttactggcgtgaaccaccg | agaggaagggggcattgaat |
| E-box 3 | gatactctcgcctcggcctc | tacagactgccctcttccct |
