# Supplementary material for: BMAL2 is a druggable target for ovarian clear cell carcinoma (OCCC)
Source: EMBO Mol Med. 2026 Apr 3;18(5):1933–66. doi: 10.1038/s44321-026-00414-8 (PMC13179388; doi:10.1038/s44321-026-00414-8)
Supplement: Supplementary file 4 — Appendix [file 44321_2026_414_MOESM4_ESM.pdf]

Appendix Supplementary Methods: Detailed GW833972A structural characterization

Table of contents

SUPPLEMENTARY MEHODS

1. General Information……………………………………………………page 2

2. Synthesis……………………………………………………………page 2

3. NMR spectra………………………………………………………...page 6

SUPPLEMENTARY FIGURES

Appendix Figure S1……………………………………………………page 3

Appendix Figure S2……………………………………………………page 6

Appendix Figure S3……………………………………………………page 6

Appendix Figure S4……………………………………………………page 7

Appendix Figure S5……………………………………………………page 7

Appendix Figure S6……………………………………………………page 8

Appendix Figure S7……………………………………………………page 8

Appendix Figure S8……………………………………………………page 9

Appendix Figure S9……………………………………………………page 9

Appendix Figure S10...…………………………………………………page 10

1. General Information

Nuclear magnetic resonance (NMR) spectra

All reported compounds were characterized by ^{1}H, ^{13}C, and ^{19}F NMR spectra recorded on a Bruker AVIII HD 400MHz spectrometer. Solvents used to dissolve compounds were indicated in the procedures accordingly. The chemical shifts are reported in ppm and were internally referenced to the residual solvent signals: for $CDCl_3$, δ 7.26 ppm for ^{1}H NMR and δ 77.16 ppm for ^{13}C NMR; for DMSO-d_6, δ 2.50 ppm for ^{1}H NMR and δ 39.52 ppm for ^{13}C NMR, respectively. The coupling constants (*J*) were reported in Hz, and the splitting patterns were singlet, doublet, triplet, quartet, multiplet, and broad peaks, abbreviated as s, d, t, q, m, and bs, respectively.

High-resolution mass spectroscopy (HRMS)

HRMS were obtained on a Bruker microTOF-QII mass spectrometer connected with an Agilent 1260 Infinity HPLC. The ionization source parameters were ESI positive, nebulizer (2.5 Bar), capillary (4.5 kV), dry heater (220 ºC), end plate offset (-500 V), scan region (50-3000 m/z), dry gas (8.0 L/min), and collision cell RF (250.0 Vpp).

2. Synthesis

Materials

All the reagents, solvents, and the starting materials were commercially purchased with high purities and used as received without further purification unless otherwise noted. Benzyl 2-chloro-4-(trifluoromethyl)pyrimidine-5-carboxylate was purchased from Matrix Scientific, 3-chloroaniline, methylene chloride from Thermo Scientific, 4-picolylamine from AK Scientific, ethanol (EtOH) from Shimakyu Chemical, potassium hydroxide from Showa Chemical, and 1,4-dioxane from Fisher Scientific. In general, the progress of the reactions was monitored by thin-layer chromatography (TLC) plates using Merck silica gel 60 F_{254}. The handheld UV lamp manufactured by Analytik Jena, UVG-11 (254 nm and 365 nm), was used

to identify the spots.

Benzyl 2-((3-chlorophenyl)amino)-4-(trifluoromethyl)pyrimidine-5-carboxylate (2)

A solution of compound **1** (1.27 g, 4.00 mmol, 1.00 equiv.) in 1,4-dioxane (13 mL) was stirred at room temperature under an argon atmosphere, followed by the addition of 3-chloroaniline (2.1 mL, 19.9 mmol, 4.96 equiv.), and the reaction mixture was stirred for 18 h at room temperature. Upon completion of the reaction, 1,4-dioxane was removed under reduced pressure using a rotary evaporator. The resulting solid was redissolved in ethyl acetate (50 mL) and washed with 2 N $HCl_{(aq)}$ (25 mL). The organic layer was dried over anhydrous Na_2SO_4, filtered, and concentrated to approximately 5 mL. The resulting mixture was triturated with hexane (170 mL) to afford a white solid, which was collected by suction filtration and dried under reduced pressure to afford a white solid (1.34 g, 3.28 mmol, 82%). R_f = 0.36 (EA/Hex = 9/1). ^{1}H NMR (400 MHz, $CDCl_3$): δ 9.06 (s, 1H), 7.81 (s, 1H), 7.77 (bs, 1H, NH), 7.46-7.36 (m, 6H), 7.28 (t, J = 8.1 Hz, 1H), 7.11 (d, J = 7.8 Hz, 1H), 5.38 (s, 2H). ^{13}C NMR (101 MHz, $CDCl_3$): δ 162.9, 159.8, 155.6 (q, J = 36.9 Hz), 140.0, 135.0, 134.9, 130.1, 128.8, 128.7, 124.3, 120.1 (q, J = 276.4 Hz), 120.0, 118.0, 114.3, 67.2. ^{19}F NMR (376 MHz, $CDCl_3$): δ -66.7. ESI-HRMS calculated for $C_{19}H_{14}ClF_3N_3O_2^+$, [M+H$^+$] 408.0721, found 408.0721.

2-((3-Chlorophenyl)amino)-4-(trifluoromethyl)pyrimidine-5-carboxylic acid (3)

To a stirred suspension of compound **2** (1.34 g, 3.28 mmol, 1.00 equiv.) in ethanol (33 mL) was added a solution of KOH (575 mg, 10.2 mmol, 3.12 equiv.) in ethanol (11 mL), and the mixture was refluxed for 12 h under an argon atmosphere using a reflux condenser. After completion of the reaction, the mixture was cooled to room temperature, and ethanol was removed under reduced pressure using a rotary evaporator. The resulting solid was taken up in water (33 mL) and extracted with diethyl ether (50 mL). The combined aqueous layers were then acidified to pH 1 with 12 N $HCl_{(aq)}$, during which a precipitate formed. The solid was then collected by suction filtration and dried under reduced pressure to afford a yellow solid (965

mg, 3.03 mmol, 93%). R_f= 0.01 (EA). ^{1}H NMR (400 MHz, DMSO-d_6): δ 10.8 (bs, 1H, NH), 9.08 (s, 1H), 7.97 (t, J = 2.0 Hz, 1H), 7.68 (ddd, J = 8.3, 2.0, 0.8 Hz, 1H), 7.37 (t, J = 8.1 Hz, 1H), 7.12 (ddd, J = 8.0, 2.0, 0.8 Hz, 1H). ^{13}C NMR (101 MHz, DMSO-d_6): δ 164.1, 162.8, 159.6, 153.6 (q, J = 35.5 Hz), 140.4, 133.2, 130.2, 122.8, 121.7, 120.3 (q, J = 276.1 Hz), 118.3, 114.5. ^{19}F NMR (376 MHz, $CDCl_3$): δ -65.6.

2-((3-Chlorophenyl)amino)-*N*-(pyridin-4-ylmethyl)-4-(trifluoromethyl)pyrimidine -5-carboxamide (GW833972A)

A suspension of compound **3** (965 mg, 3.03 mmol, 1.00 equiv.) in methylene chloride (30 mL) was stirred at room temperature under an argon atmosphere and were added 1-hydroxybenzotriazole hydrate (701 mg, 4.58 mmol, 1.51 equiv.), 1-(3-dimethylamino-propyl)-3-ethylcarbodiimide hydrochloride (883 mg, 4.60 mmol, 1.52 equiv.), *N*,*N*-diisopropylethylamine (1.6 mL, 9.19 mmol, 3.03 equiv.), and 4-picolylamine (0.46 mL, 4.53 mmol, 1.50 equiv.) sequentially, and the resulting reaction mixture was stirred for 18 h at room temperature. Upon completion of the reaction, methylene chloride was removed under reduced pressure using a rotary evaporator. The resulting solid was redissolved in ethyl acetate (70 mL) and extracted sequentially with water (50 mL) and saturated $NaHCO_{3(aq)}$ (50 mL). The combined organic layers were dried over anhydrous Na_2SO_4, filtered, and concentrated to approximately 5 mL. The resulting solution was triturated with hexane (150 mL) to afford a white solid, which was collected by suction filtration and dried under reduced pressure to afford a white solid (1.22 g, 2.99 mmol, 98%). R_f= 0.33 (EA). ^{1}H NMR (400 MHz, DMSO-d_6): δ 10.6 (bs, 1H, NH), 9.23 (t, J = 6.0 Hz, 1H, NH), 8.93 (s, 1H), 8.53 (dd, J = 4.4, 1.6 Hz, 1H), 7.97 (t, J = 2.0 Hz, 1H), 7.66 (dd, J = 8.3, 1.9 Hz, 1H), 7.39-7.34 (m, 3H), 7.10 (ddd, J = 8.0, 2.0, 0.8 Hz, 1H), 4.49 (d, J = 6.0 Hz, 2H). ^{13}C NMR (101 MHz, DMSO-d_6): δ 164.3, 160.6, 159.4, 152.1 (q, J = 36.9 Hz), 149.9, 148.1, 141.1, 133.5, 130.6, 122.7, 122.5, 120.7 (q, J = 276.9 Hz), 119.7, 119.2, 118.2, 42.2. ^{19}F NMR (376 MHz, $CDCl_3$): δ -65.6. ESI-HRMS

calculated for $C_{18}H_{14}ClF_3N_5O^+$ [M+H$^+$] 408.0833, found 408.0830.

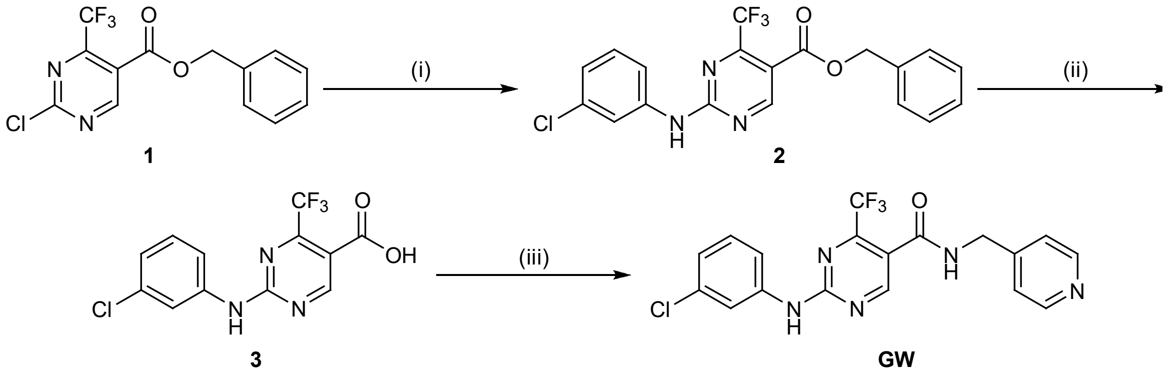

Appendix Figure S1. Schematic diagram of GW833972A synthesis.

Compound 1: Benzyl 2-chloro-4-(trifluoromethyl)pyrimidine-5-carboxylate

Compound 2: Benzyl 2-((3-chlorophenyl)amino)-4-(trifluoromethyl)pyrimidine-5-carboxylate

Compound 3: 2-((3-Chlorophenyl)amino)-4-(trifluoromethyl)pyrimidine-5-carboxylic acid

GW: GW833972A [2-((3-Chlorophenyl)amino)-N-(pyridin-4-ylmethyl)-4-(trifluoromethyl)pyrimidine -5-carboxamide]

(i) 3-chloroaniline, 1,4-dioxane, RT, 18 h

(ii) KOH, EtOH, reflux, 12 h

(iii) EDC·HCl, HOBt·H2O, 4-picolylamine, RT, 18 h.

All reactions were carried out under an argon atmosphere.

3. NMR spectra

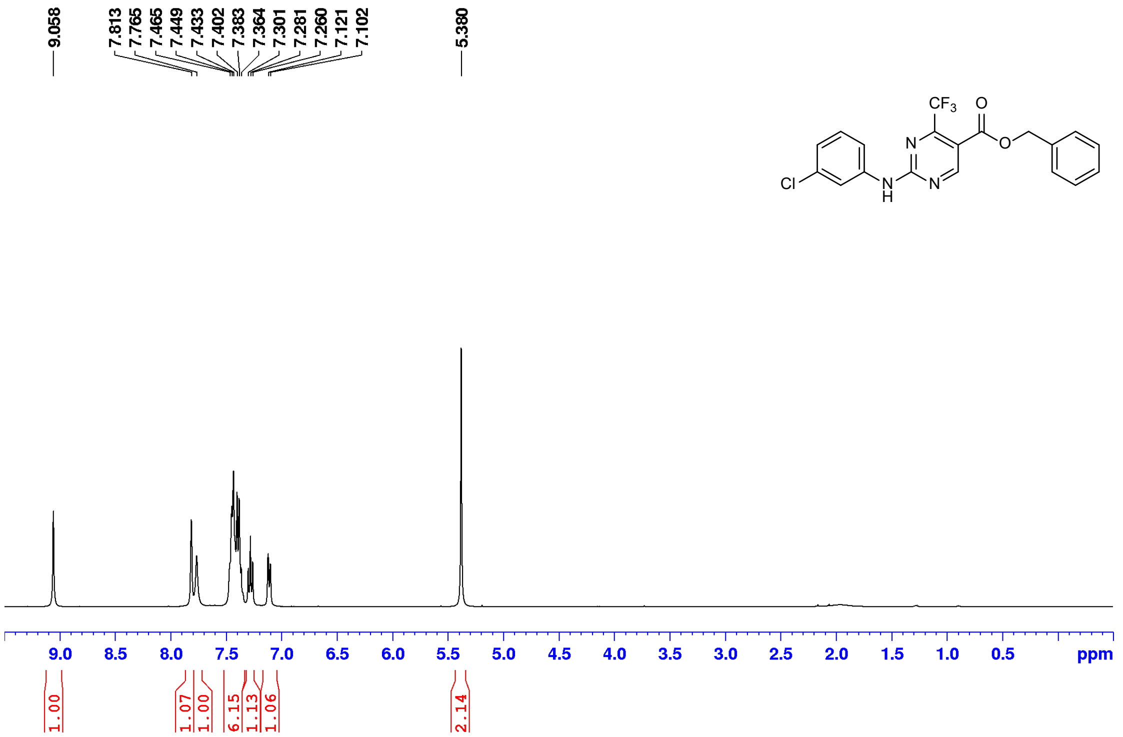

Appendix Figure S2. ^{1}H-NMR spectrum of benzyl 2-((3-chlorophenyl)amino)-4-(trifluoromethyl)pyrimidine -5-carboxylate (**2**) in $CDCl_3$.

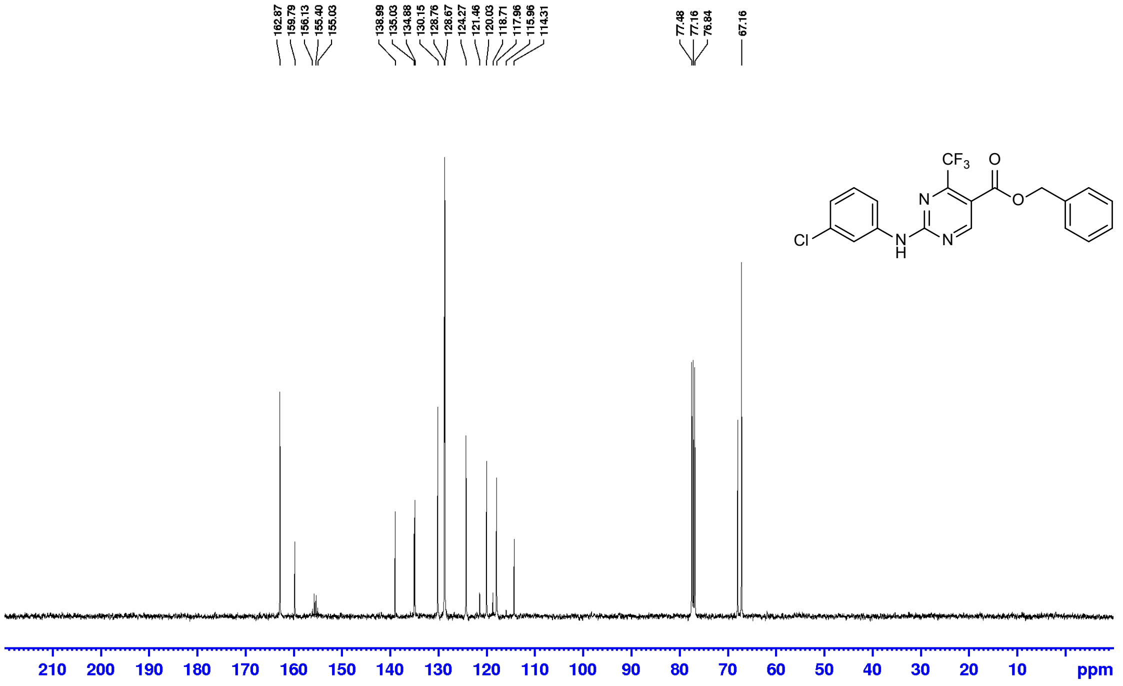

Appendix Figure S3. ^{13}C-NMR spectrum of benzyl 2-((3-chlorophenyl)amino)-4-(trifluoromethyl)pyrimidine -5-carboxylate (**2**) in $CDCl_3$.

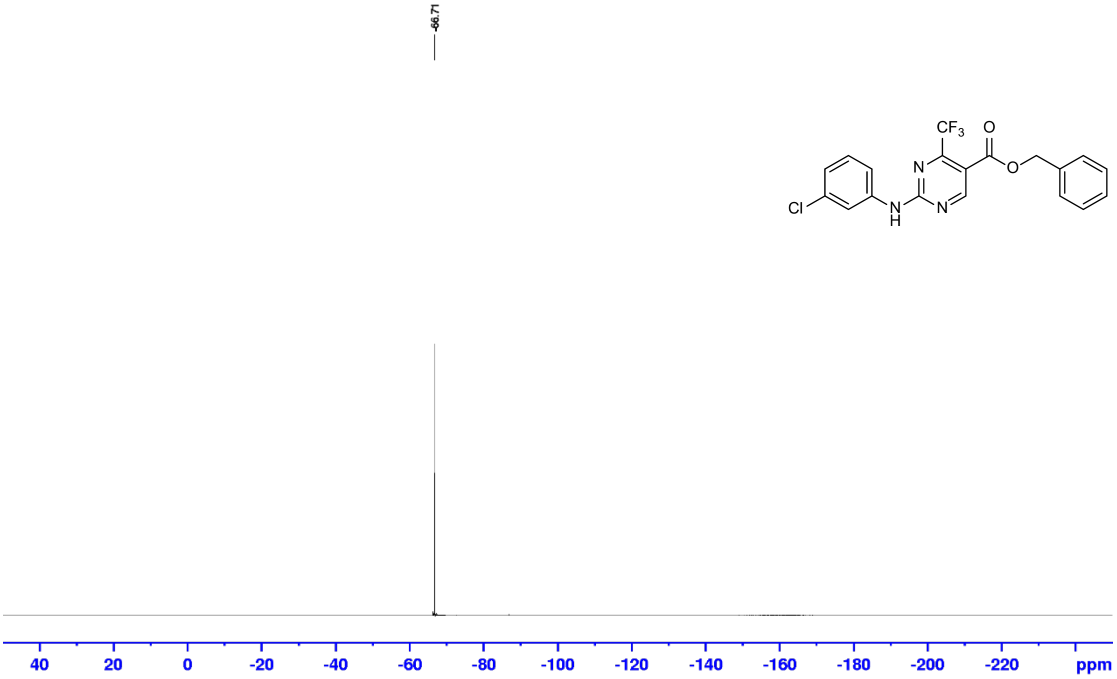

Appendix Figure S4. ^{19}F-NMR spectrum of benzyl 2-((3-chlorophenyl)amino)-4-(trifluoromethyl)pyrimidine -5-carboxylate (**2**) in $CDCl_3$.

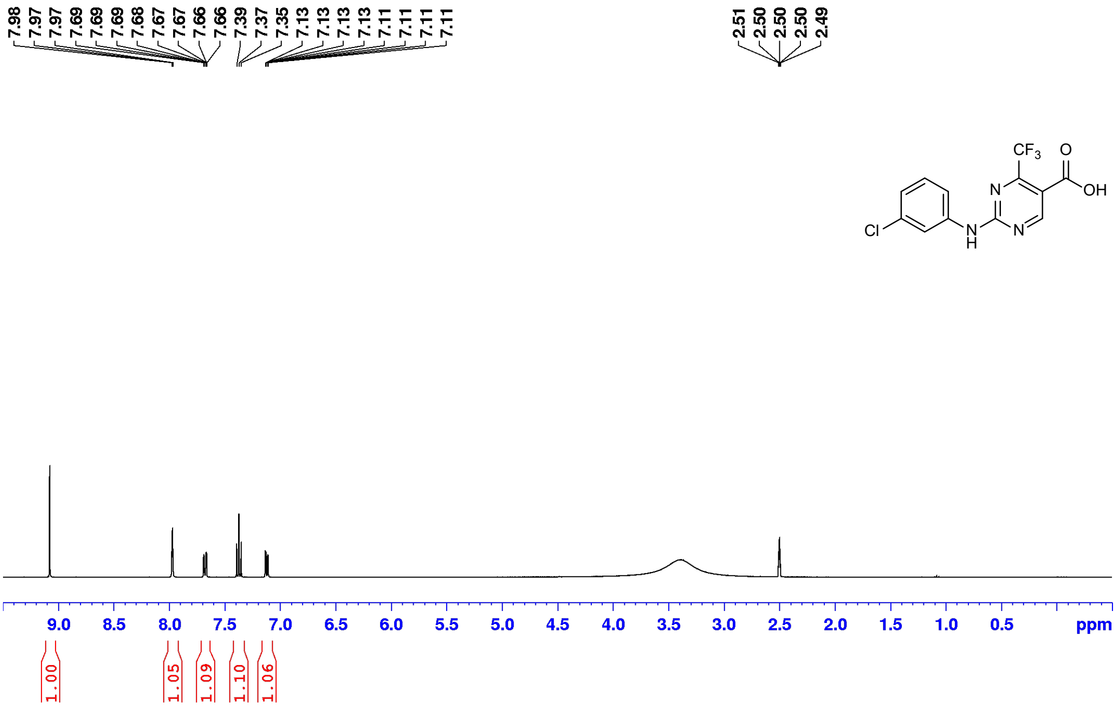

Appendix Figure S5. ^{1}H-NMR spectrum of 2-((3-chlorophenyl)amino)-4-(trifluoromethyl)pyrimidine-5-carboxylic acid (**3**) in DMSO-d_6.

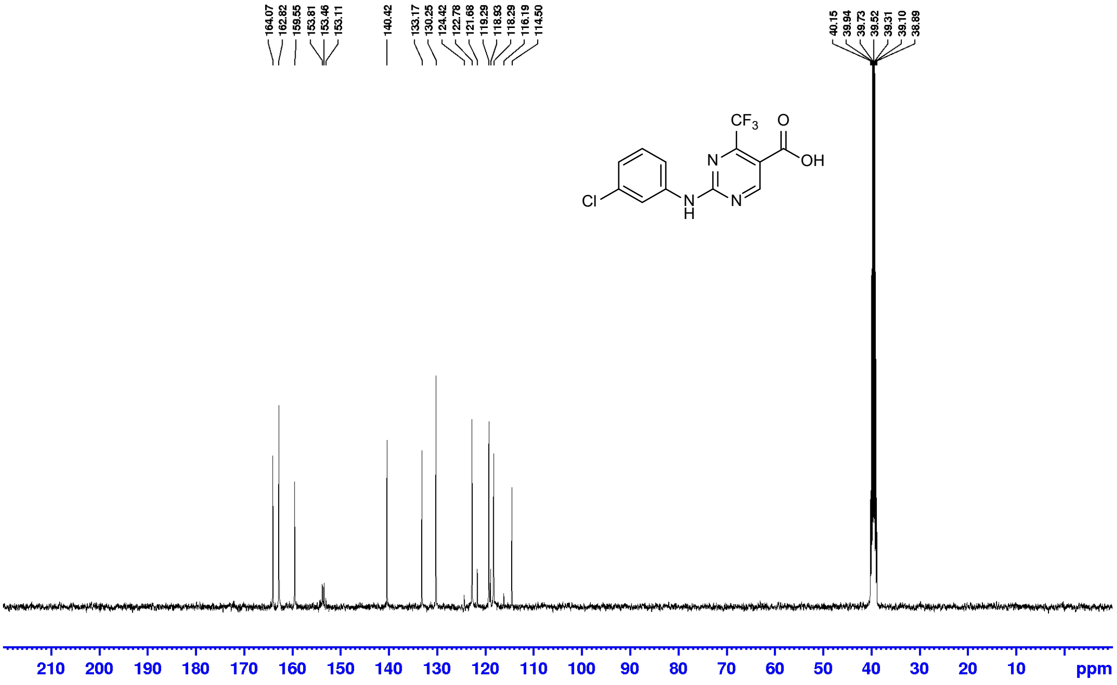

Appendix Figure S6. ^{13}C-NMR spectrum of 2-((3-chlorophenyl)amino)-4-(trifluoromethyl)pyrimidine-5-carboxylic acid (**3**) in DMSO-d_6.

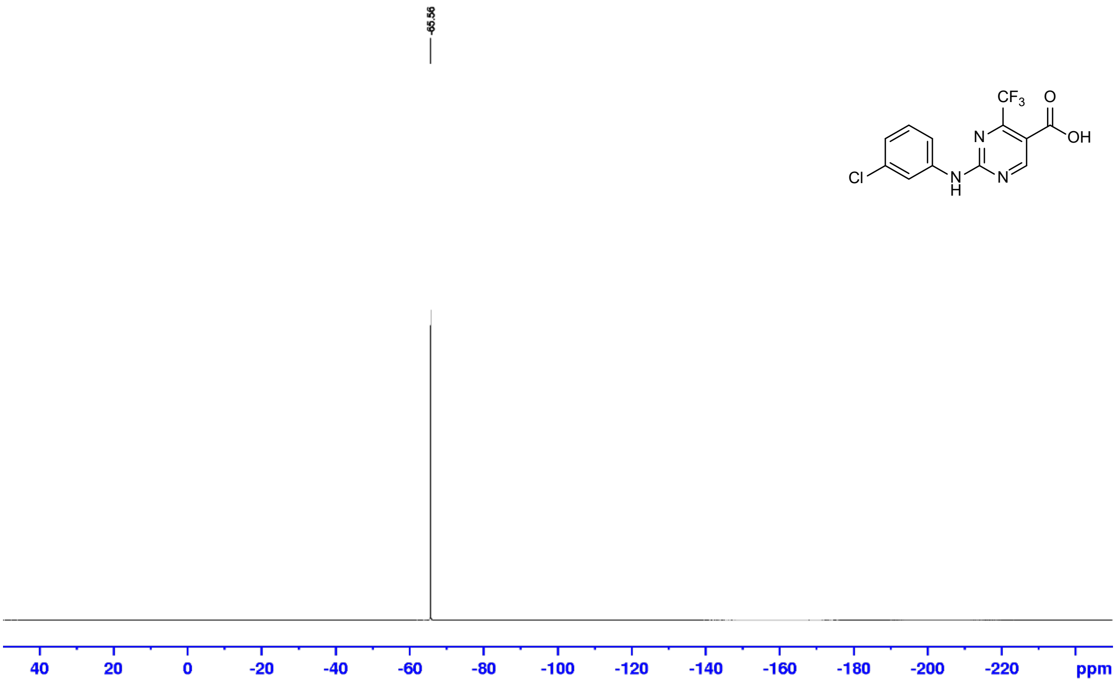

Appendix Figure S7. ^{19}F-NMR spectrum of 2-((3-chlorophenyl)amino)-4-(trifluoromethyl)pyrimidine-5-carboxylic acid (**3**) in DMSO-d_6.

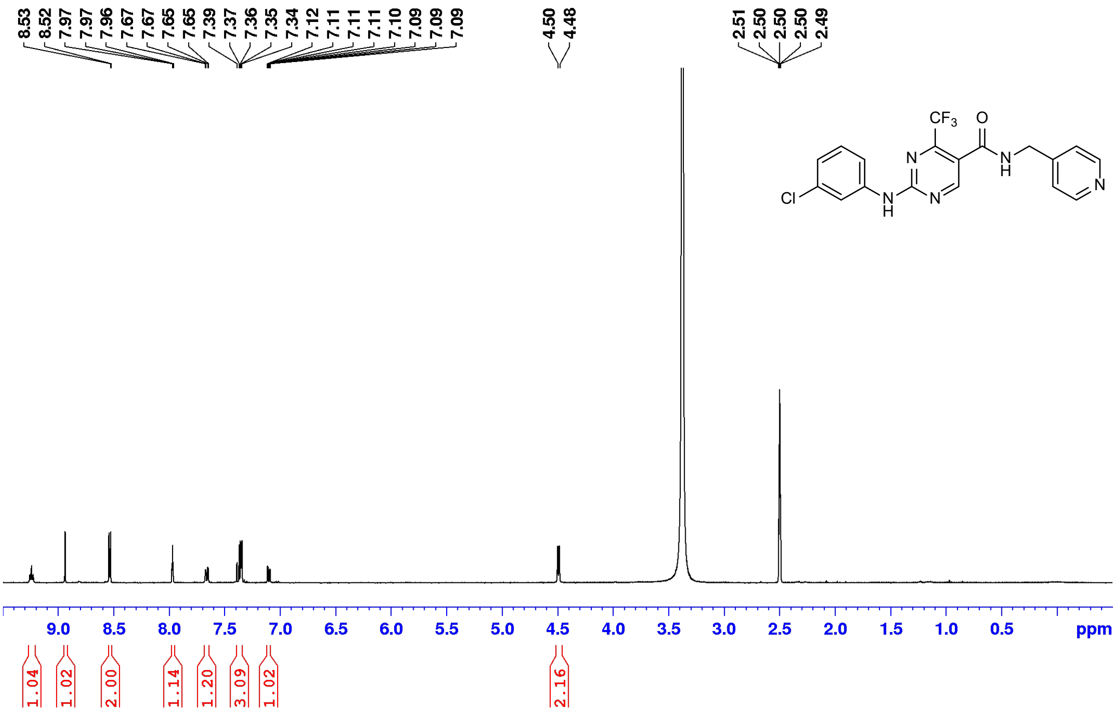

Appendix Figure S8. ^{1}H-NMR spectrum of 2-((3-chlorophenyl)amino)-*N*-(pyridin-4-ylmethyl)-4-(trifluoro methyl)pyrimidine -5-carboxamide (**GW833972A**) in DMSO-d_6.

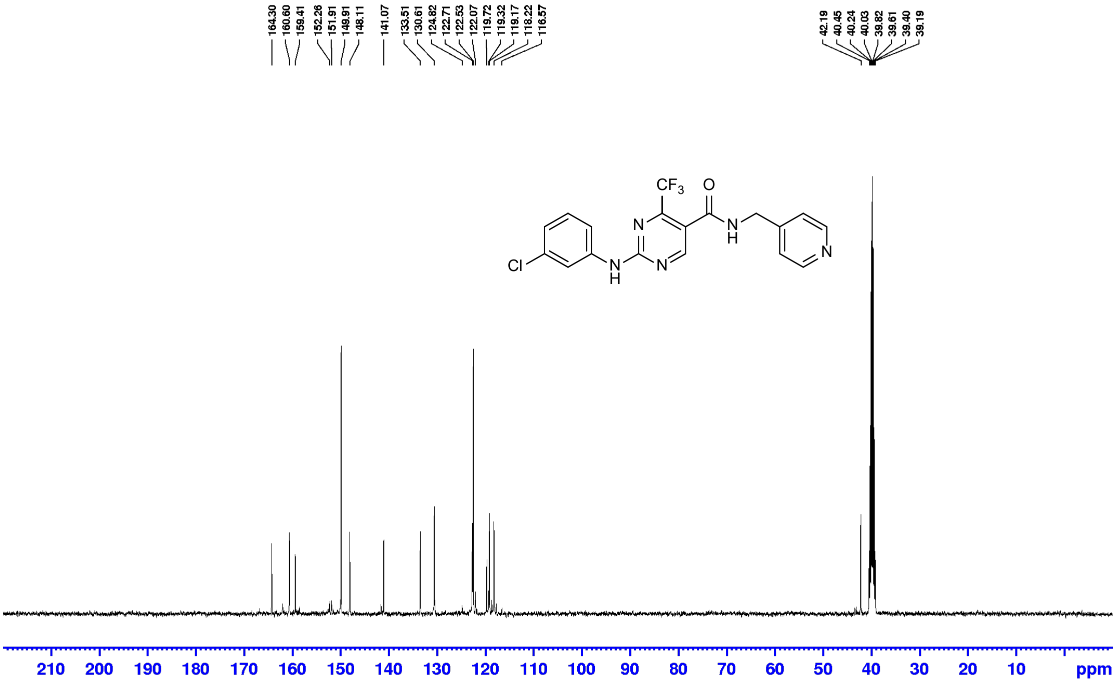

Appendix Figure S9. ^{13}C-NMR spectrum of 2-((3-chlorophenyl)amino)-*N*-(pyridin-4-ylmethyl)-4-(trifluoro methyl)pyrimidine -5-carboxamide (**GW833972A**) in DMSO-d_6.

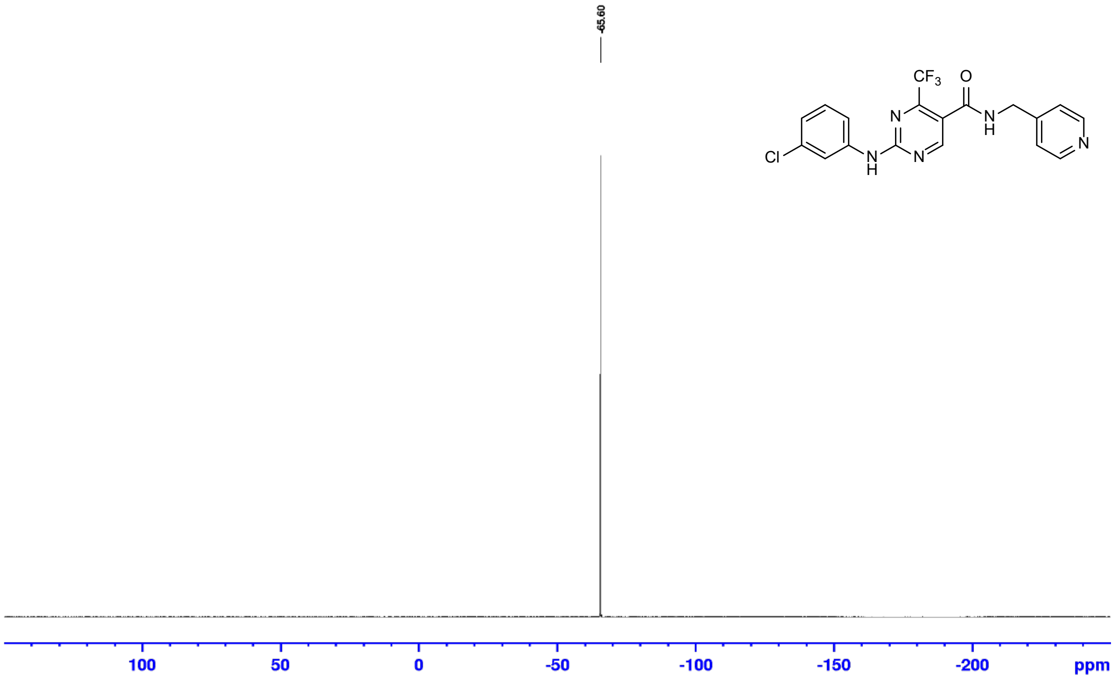

Appendix Figure S10. ^{19}F-NMR spectrum of 2-((3-chlorophenyl)amino)-*N*-(pyridin-4-ylmethyl)-4-(trifluoro methyl)pyrimidine -5-carboxamide (**GW833972A**) in DMSO-d_6.
