# Supplementary figures and images for: BMAL2 is a druggable target for ovarian clear cell carcinoma (OCCC) (part 1 of 3)
Source: EMBO Mol Med. 2026 Apr 3;18(5):1933–66. doi: 10.1038/s44321-026-00414-8 (PMC13179388; doi:10.1038/s44321-026-00414-8)

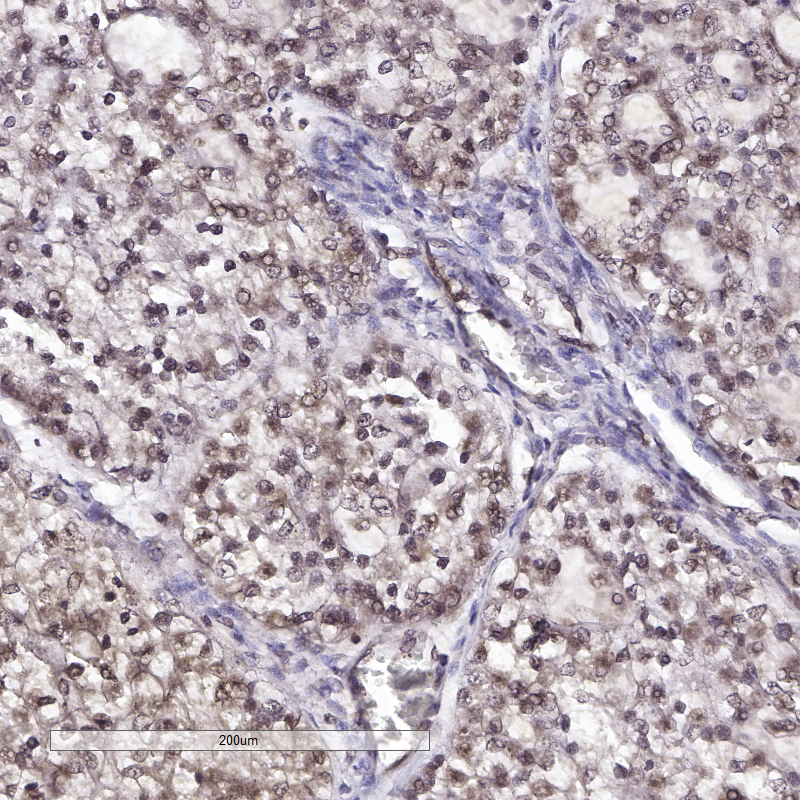

Supplement: Supplementary file 8 — Source data Fig. 1 [file 44321_2026_414_MOESM8_ESM.zip › Fig. 1/Fig. 1G/Clinical sample - BMAL2 IHC.tif]

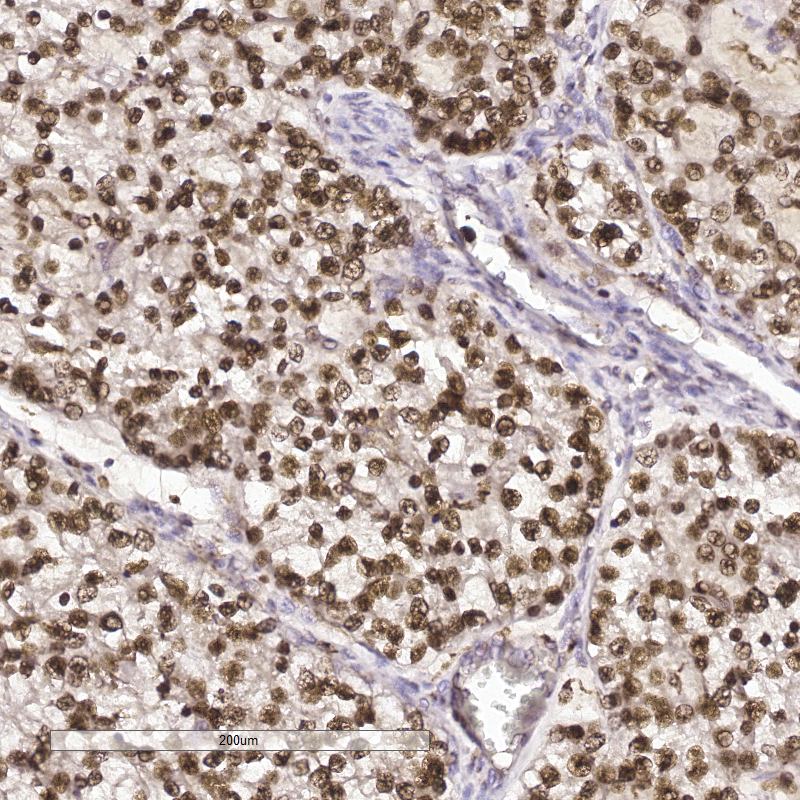

Supplement: Supplementary file 8 — Source data Fig. 1 [file 44321_2026_414_MOESM8_ESM.zip › Fig. 1/Fig. 1G/Clinical sample - PAX8 IHC.tif]

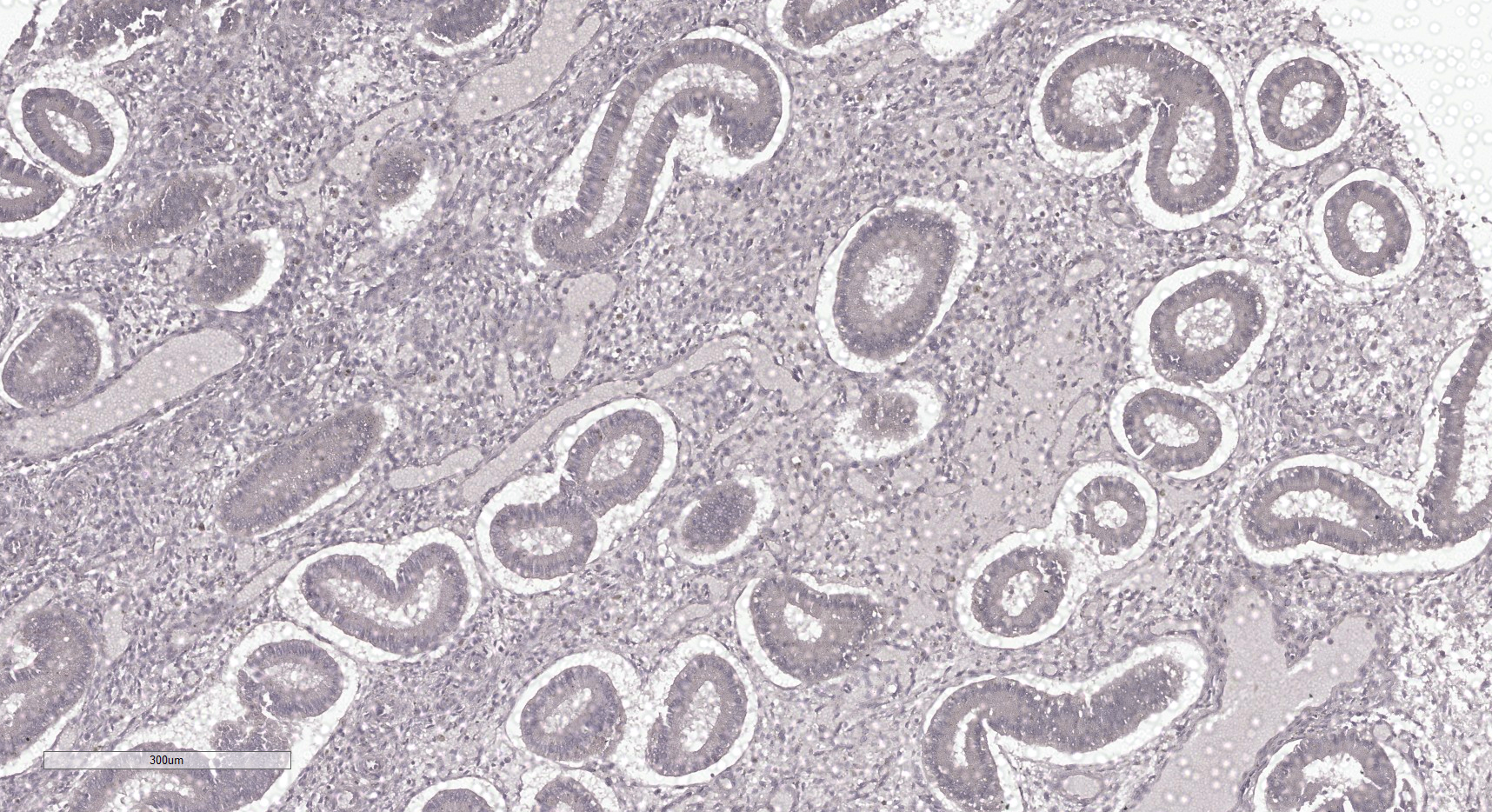

Supplement: Supplementary file 8 — Source data Fig. 1 [file 44321_2026_414_MOESM8_ESM.zip › Fig. 1/Fig. 1G/Normal endometrium tissue - BMAL2 IHC.tif]

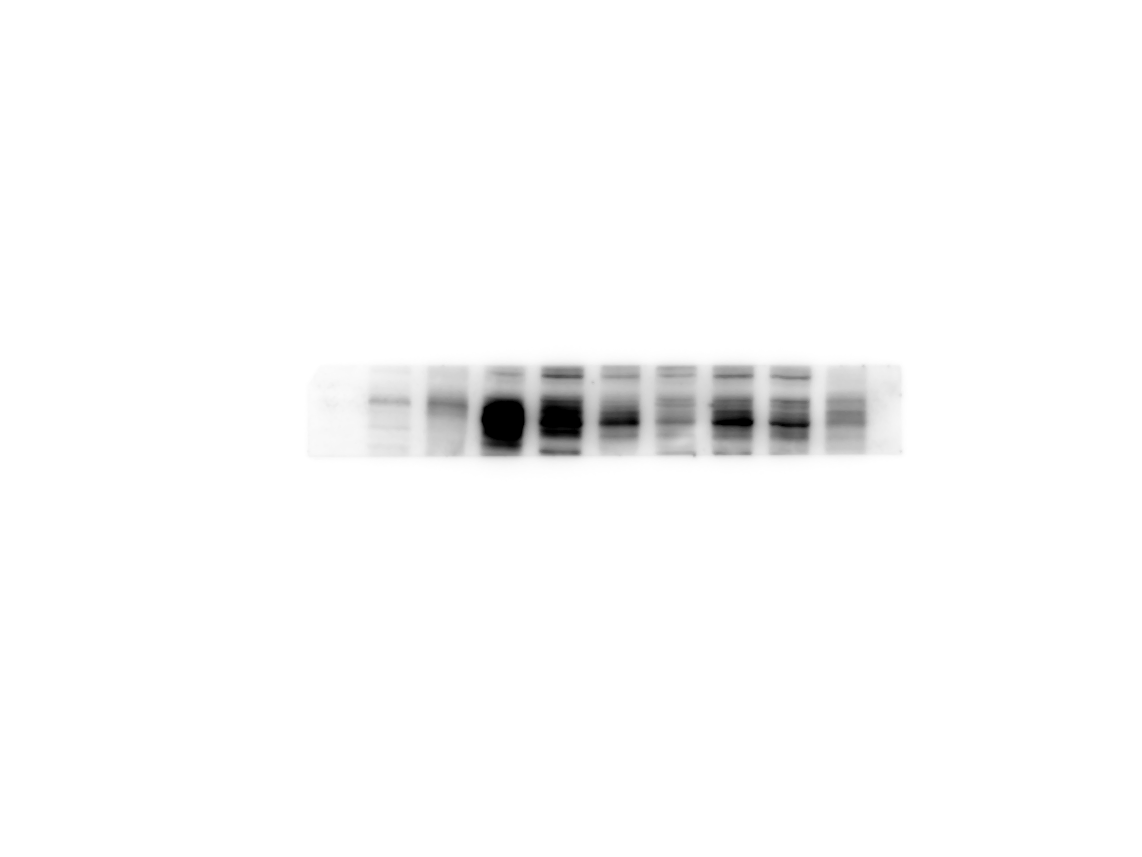

Supplement: Supplementary file 9 — Source data Fig. 2 [file 44321_2026_414_MOESM9_ESM.zip › Fig. 2/Fig. 2A/BMAL2 IB.tif]

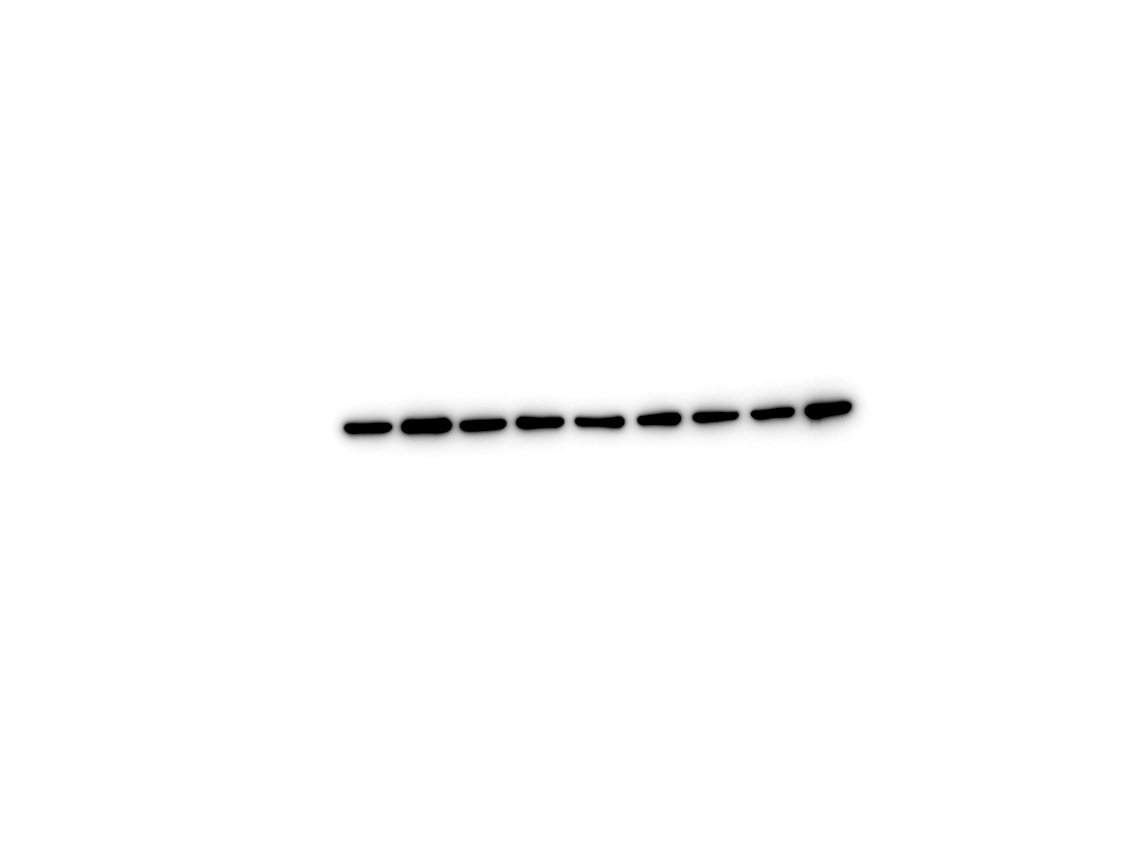

Supplement: Supplementary file 9 — Source data Fig. 2 [file 44321_2026_414_MOESM9_ESM.zip › Fig. 2/Fig. 2A/GAPDH IB.tiff]

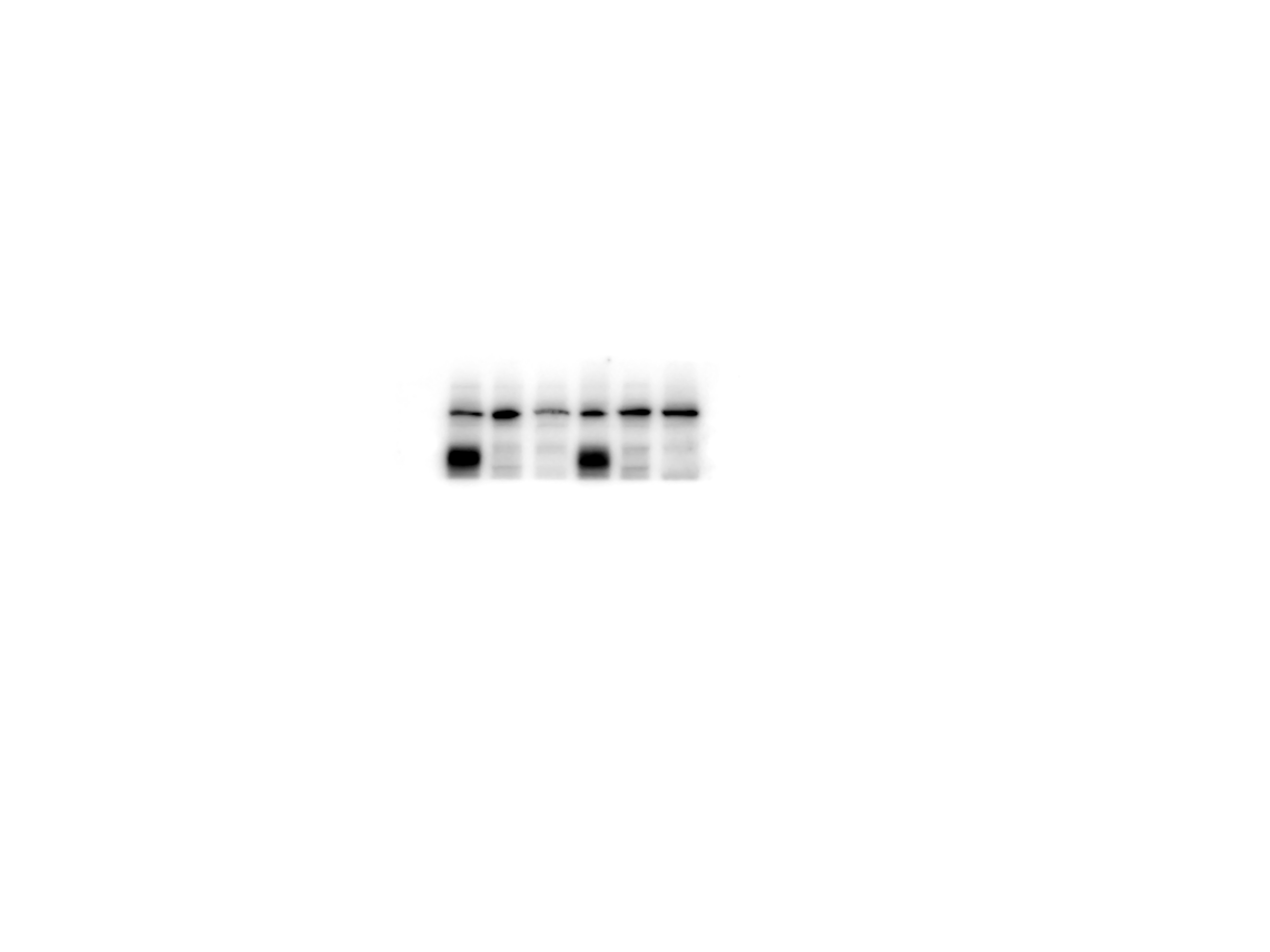

Supplement: Supplementary file 9 — Source data Fig. 2 [file 44321_2026_414_MOESM9_ESM.zip › Fig. 2/Fig. 2B/ES-2 BMAL2.tif]

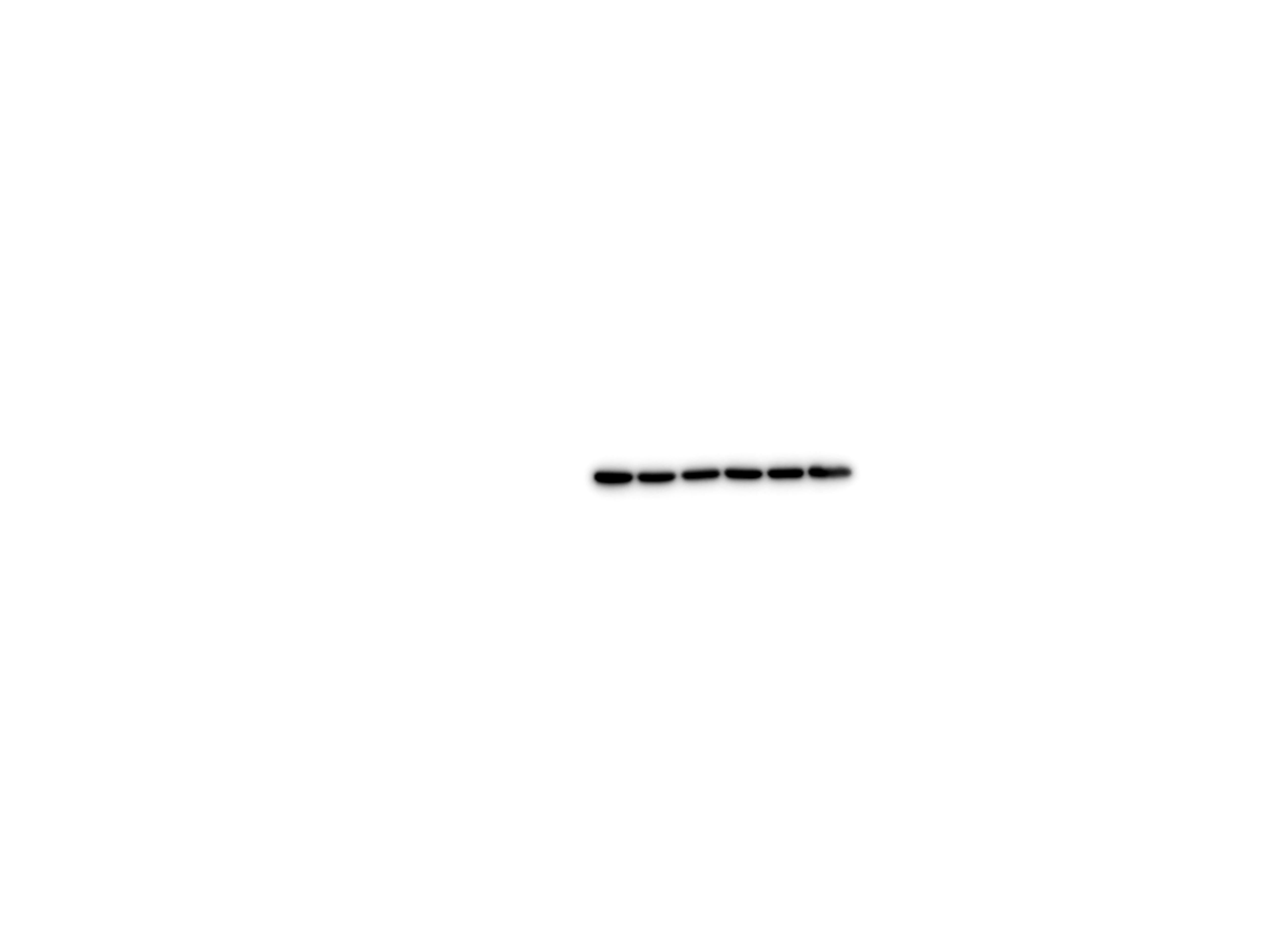

Supplement: Supplementary file 9 — Source data Fig. 2 [file 44321_2026_414_MOESM9_ESM.zip › Fig. 2/Fig. 2B/ES-2 GAPDH.tif]

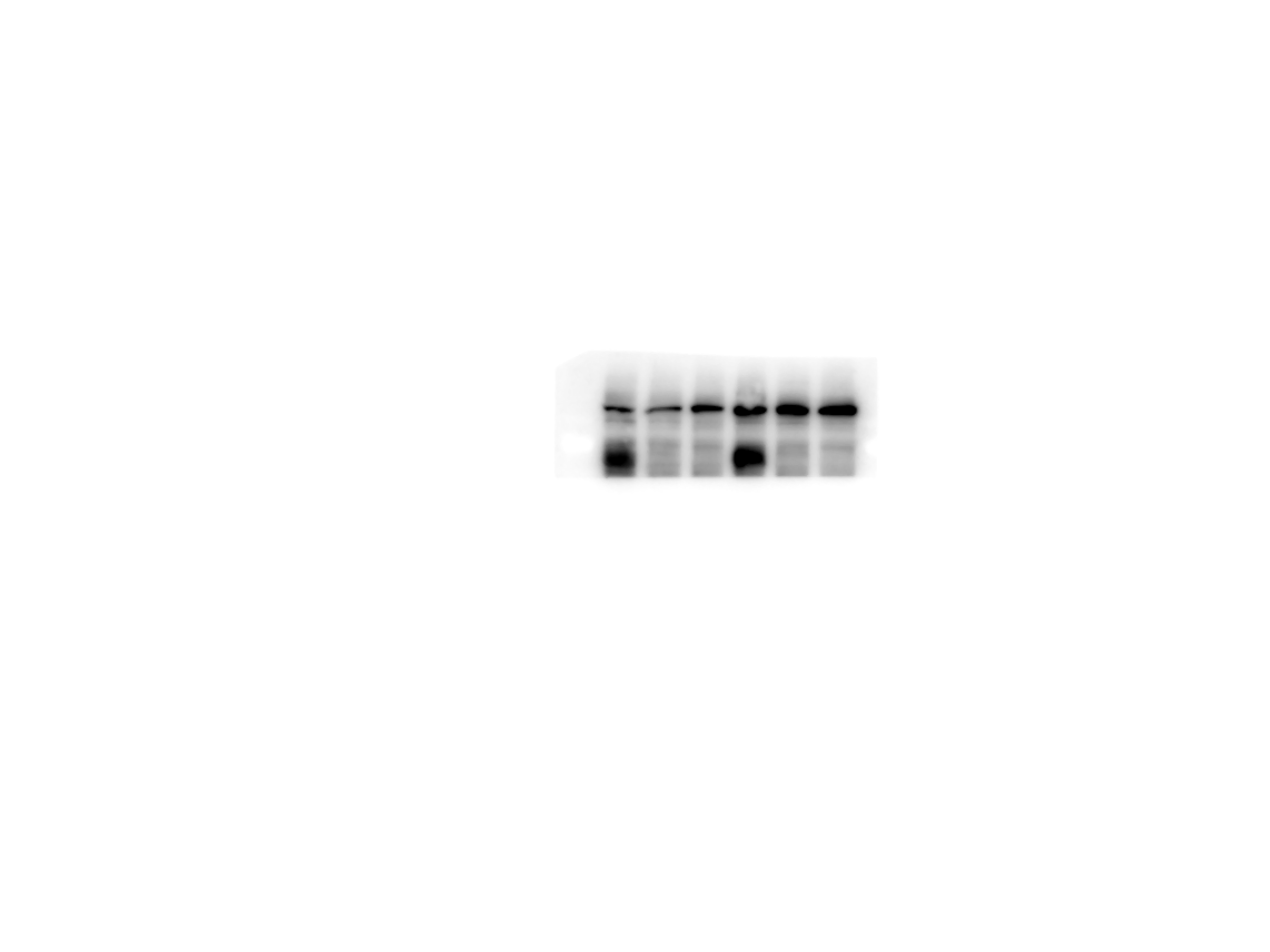

Supplement: Supplementary file 9 — Source data Fig. 2 [file 44321_2026_414_MOESM9_ESM.zip › Fig. 2/Fig. 2B/JHOC5 BMAL2.tif]

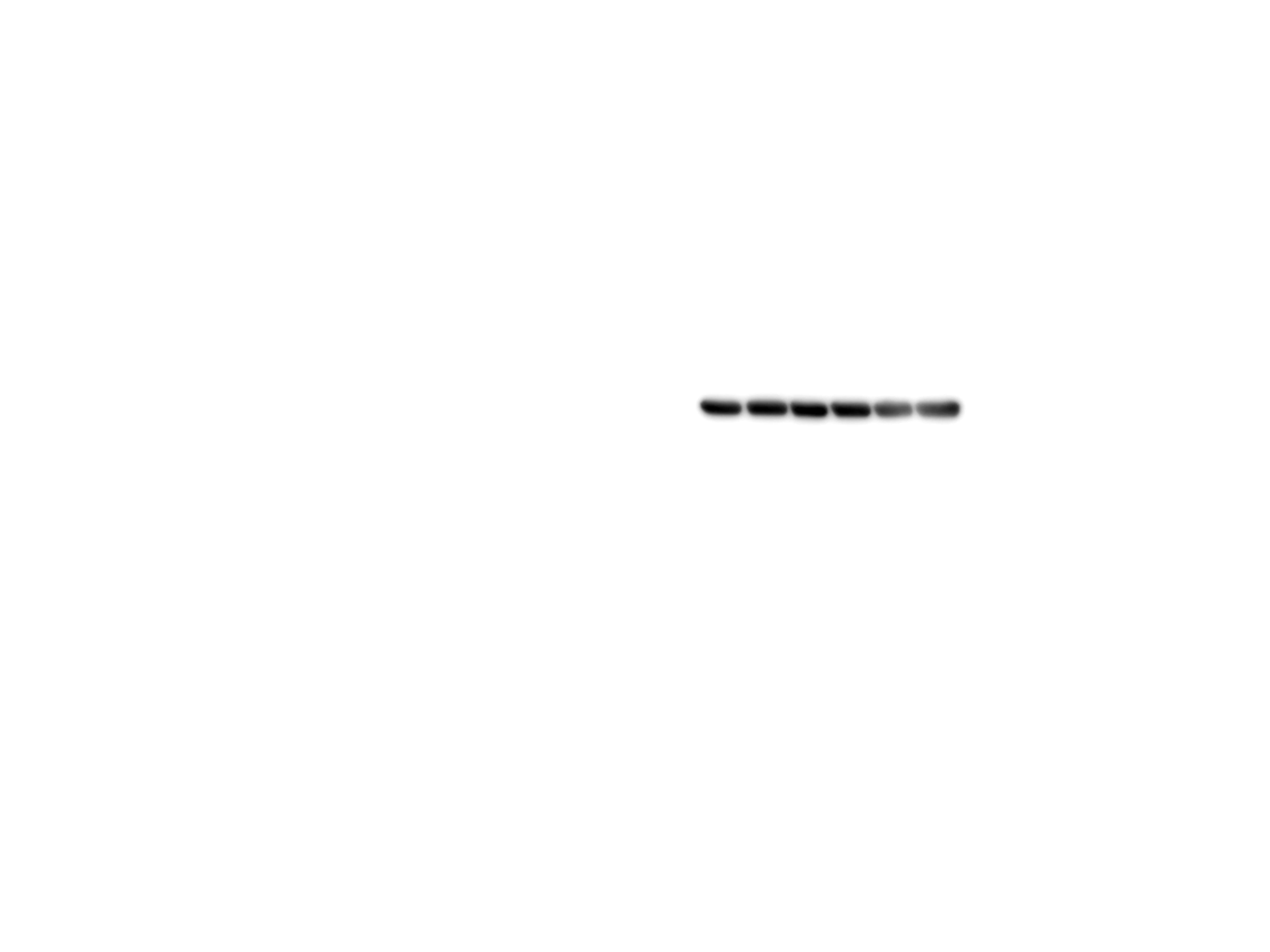

Supplement: Supplementary file 9 — Source data Fig. 2 [file 44321_2026_414_MOESM9_ESM.zip › Fig. 2/Fig. 2B/JHOC5 GAPDH.tif]

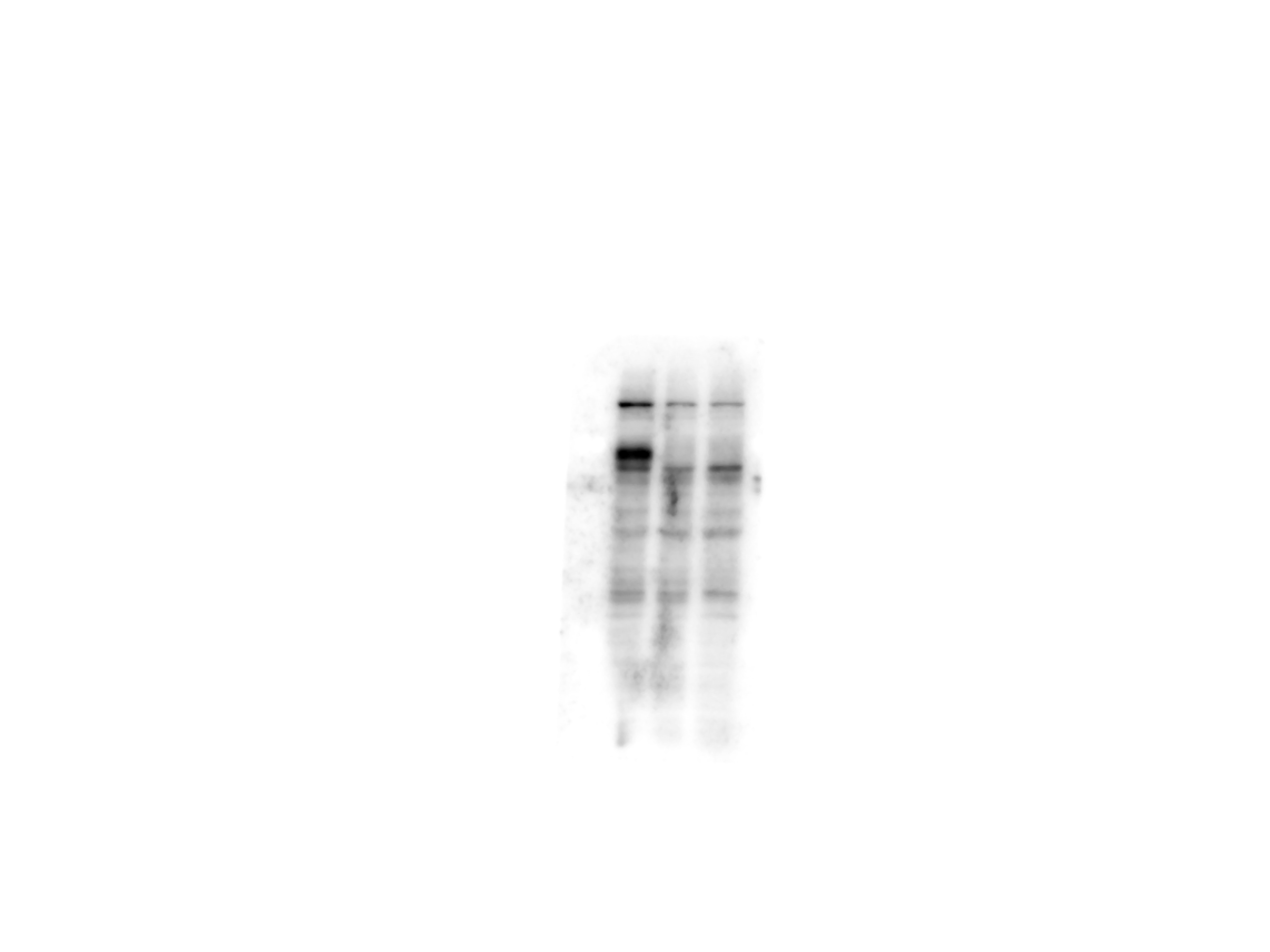

Supplement: Supplementary file 9 — Source data Fig. 2 [file 44321_2026_414_MOESM9_ESM.zip › Fig. 2/Fig. 2B/JHOC9 BMAL2.tif]

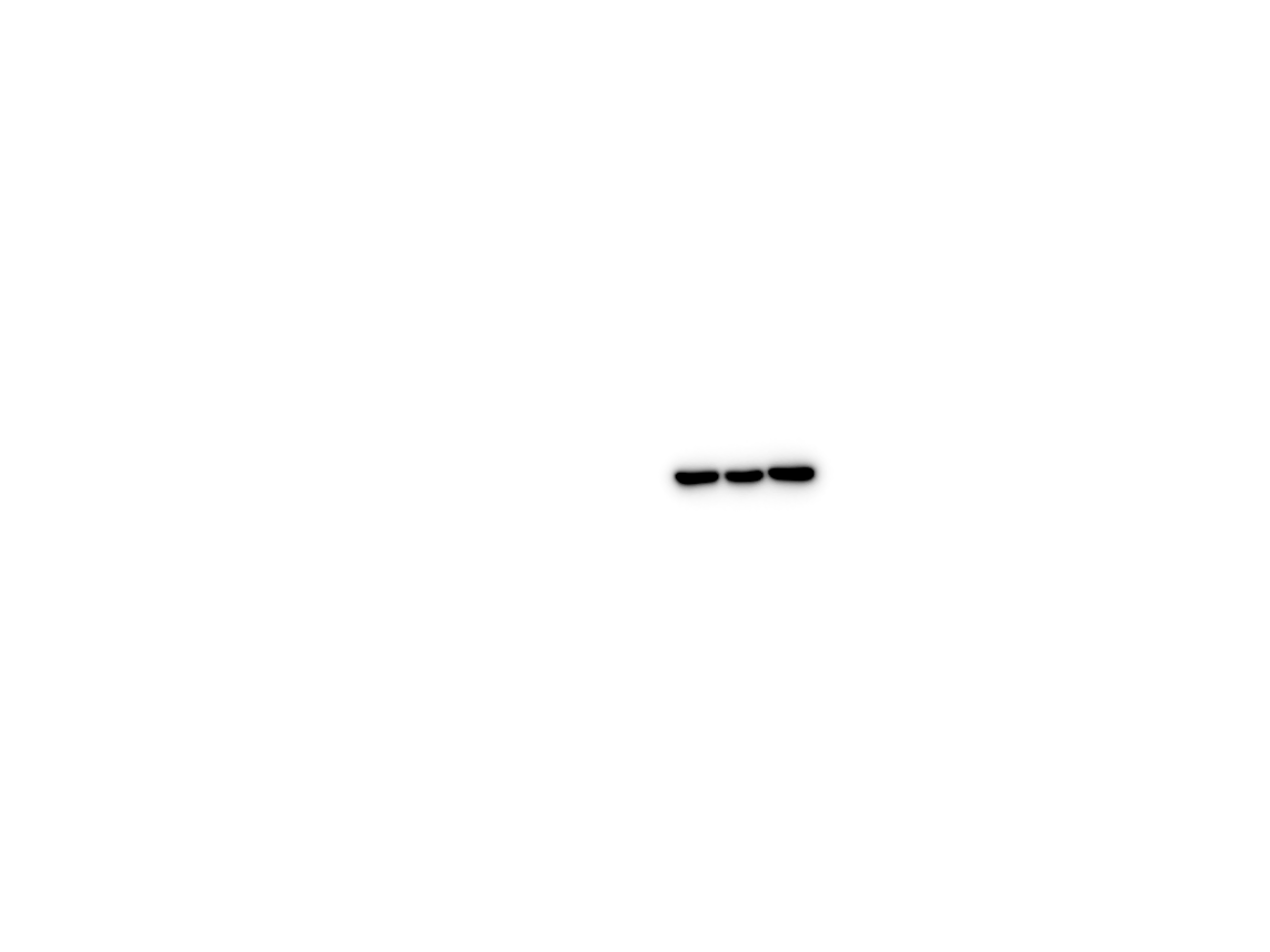

Supplement: Supplementary file 9 — Source data Fig. 2 [file 44321_2026_414_MOESM9_ESM.zip › Fig. 2/Fig. 2B/JHOC9 GAPDH.tif]

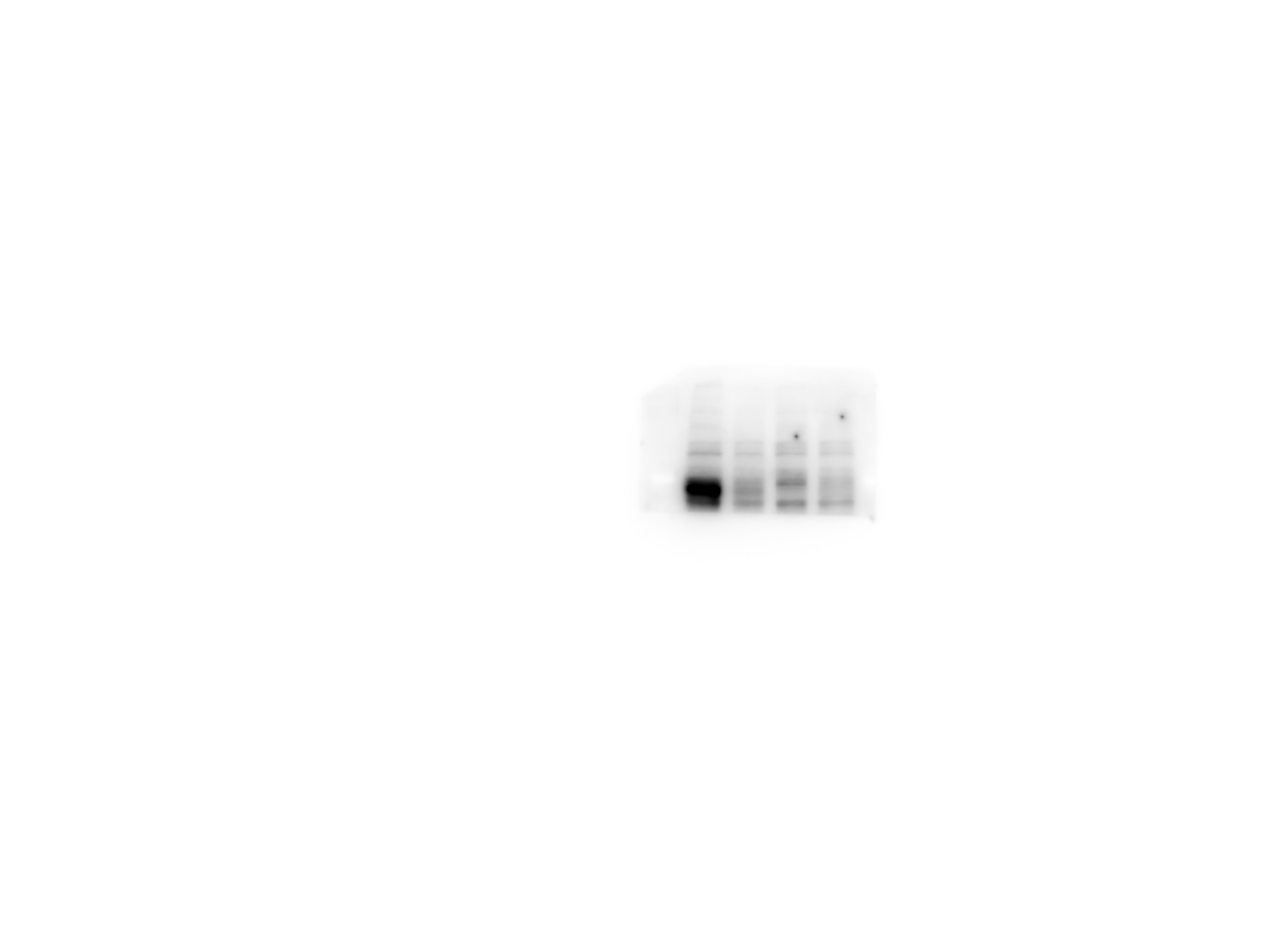

Supplement: Supplementary file 9 — Source data Fig. 2 [file 44321_2026_414_MOESM9_ESM.zip › Fig. 2/Fig. 2B/OVCA429 BMAL2.tif]

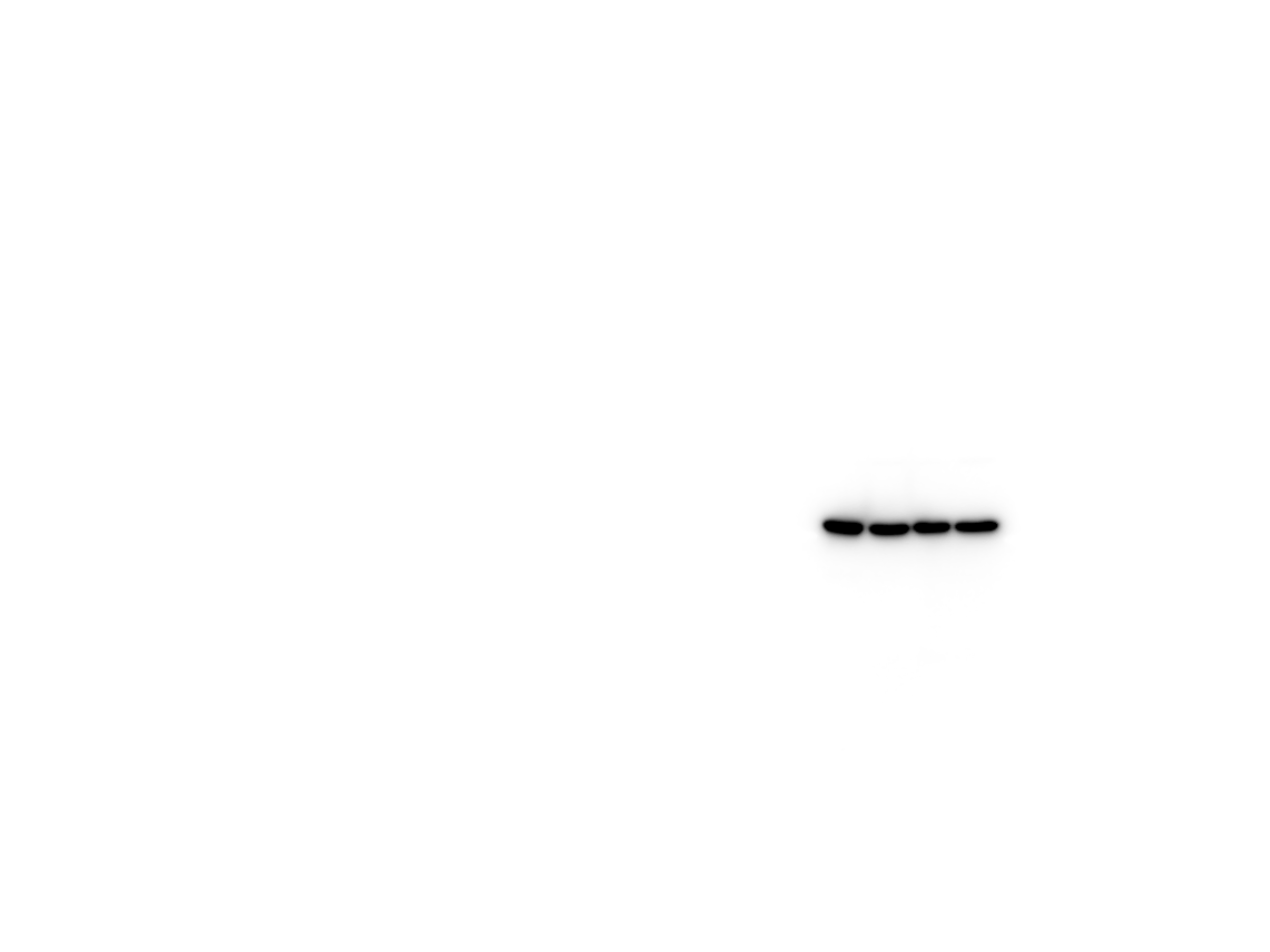

Supplement: Supplementary file 9 — Source data Fig. 2 [file 44321_2026_414_MOESM9_ESM.zip › Fig. 2/Fig. 2B/OVCA429 GAPDH.tif]

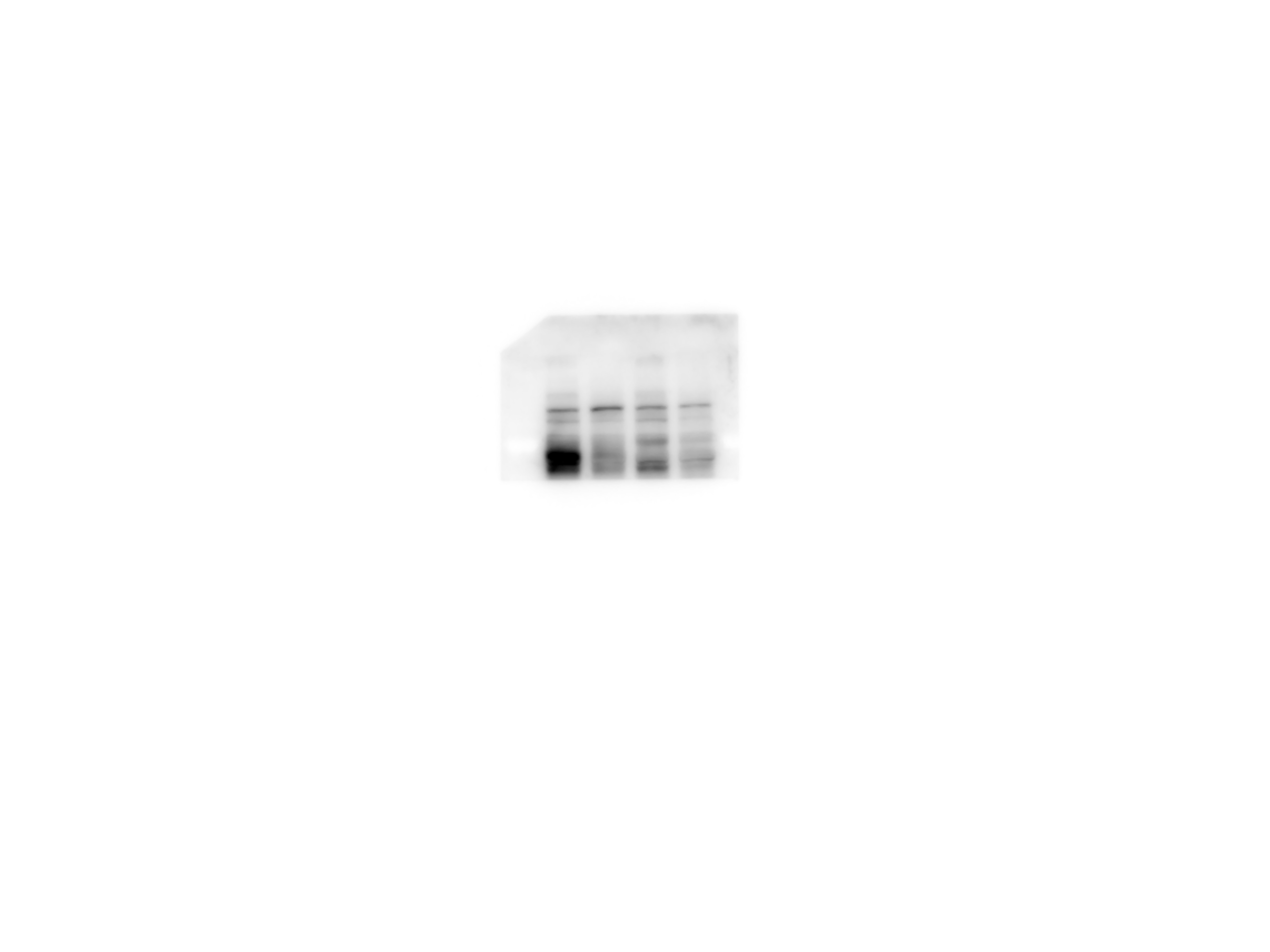

Supplement: Supplementary file 9 — Source data Fig. 2 [file 44321_2026_414_MOESM9_ESM.zip › Fig. 2/Fig. 2B/OVISE BMAL2.tif]

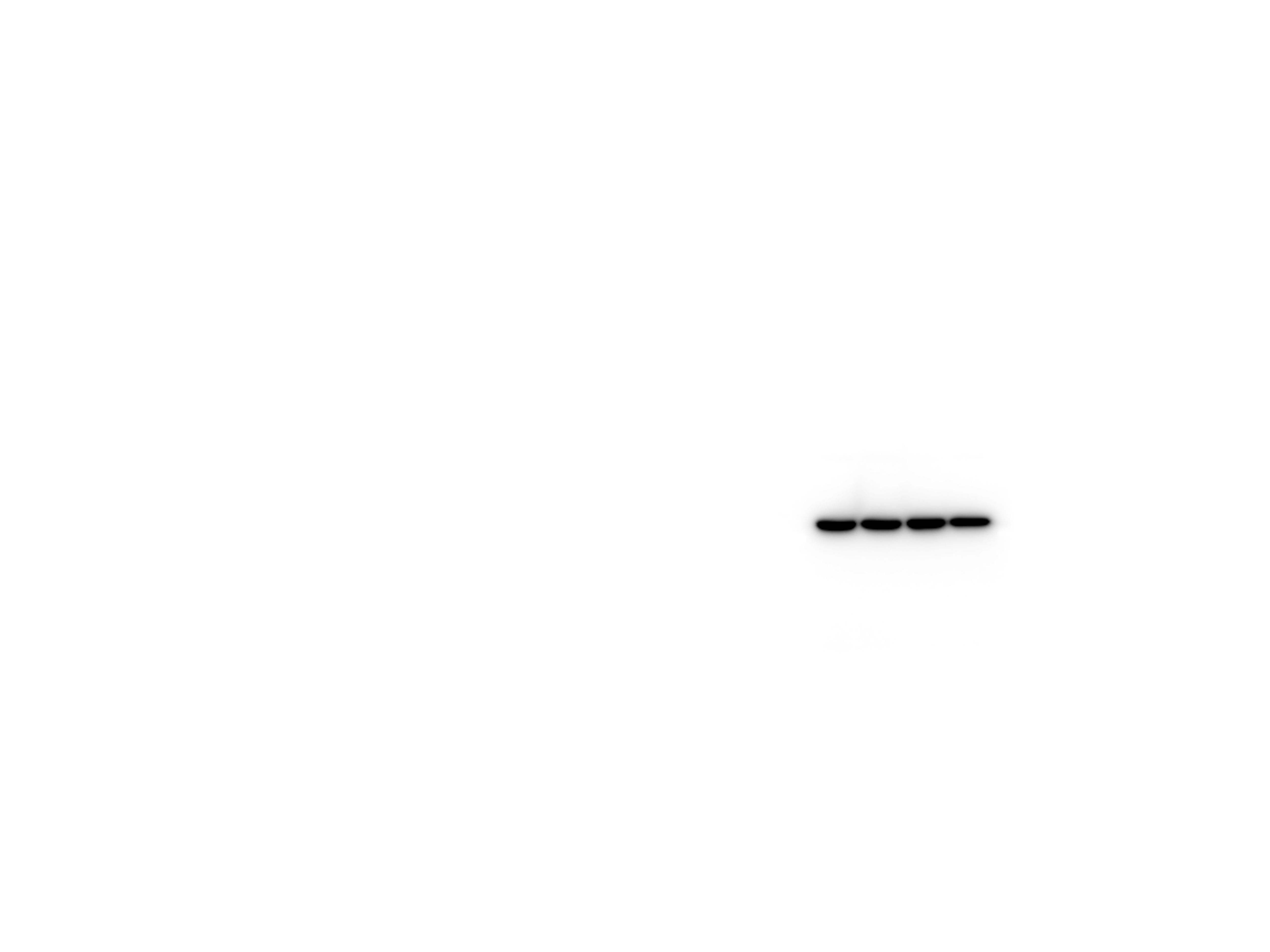

Supplement: Supplementary file 9 — Source data Fig. 2 [file 44321_2026_414_MOESM9_ESM.zip › Fig. 2/Fig. 2B/OVISE GAPDH.tif]

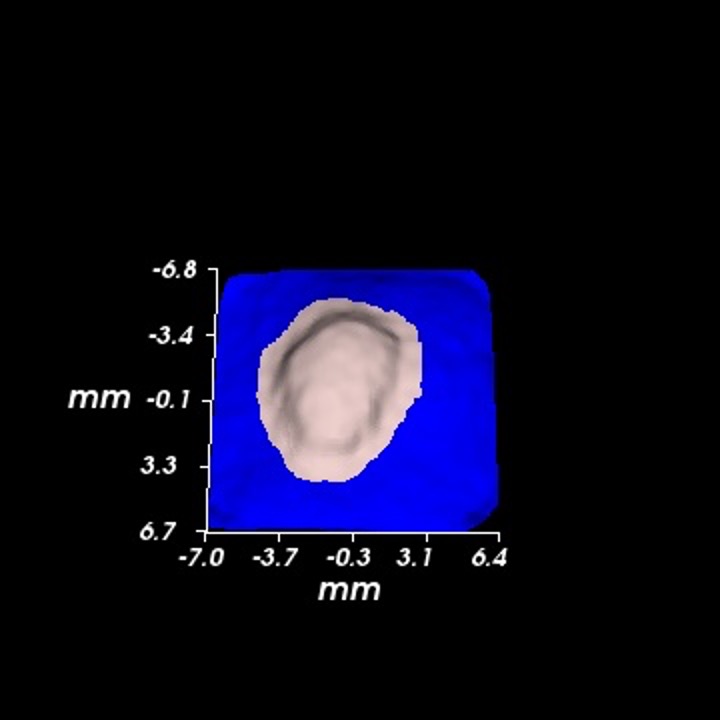

Supplement: Supplementary file 9 — Source data Fig. 2 [file 44321_2026_414_MOESM9_ESM.zip › Fig. 2/Fig. 2G/ES-2 #1.jpg]

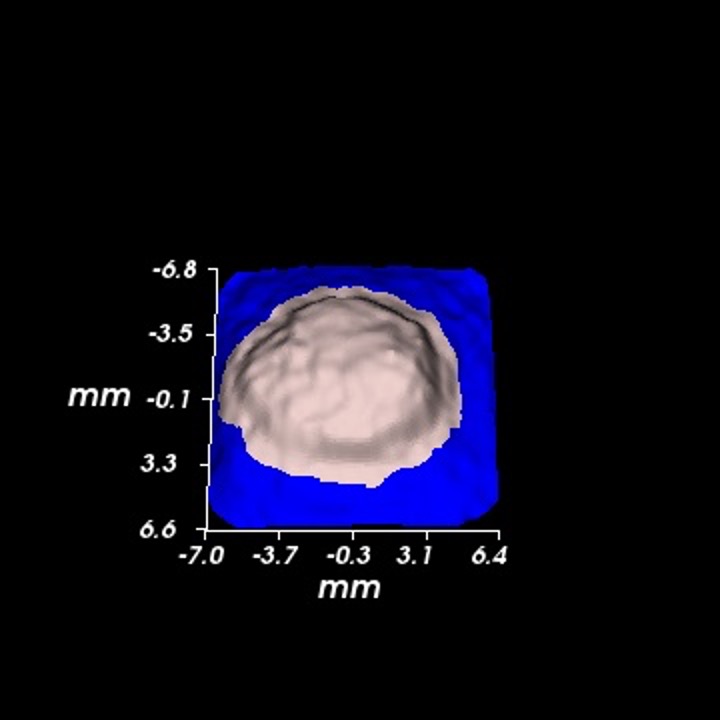

Supplement: Supplementary file 9 — Source data Fig. 2 [file 44321_2026_414_MOESM9_ESM.zip › Fig. 2/Fig. 2G/ES-2 shC.jpg]

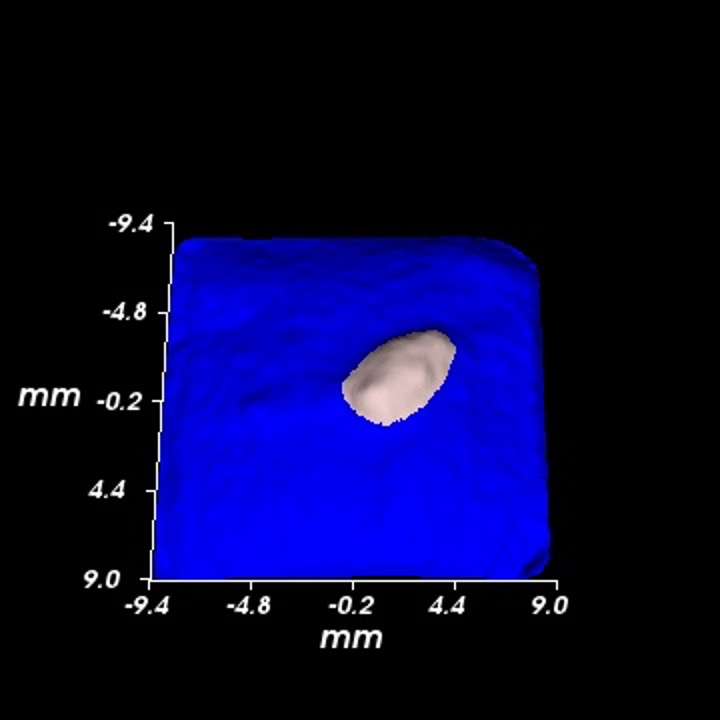

Supplement: Supplementary file 9 — Source data Fig. 2 [file 44321_2026_414_MOESM9_ESM.zip › Fig. 2/Fig. 2H/JHOC5 #1.jpg]

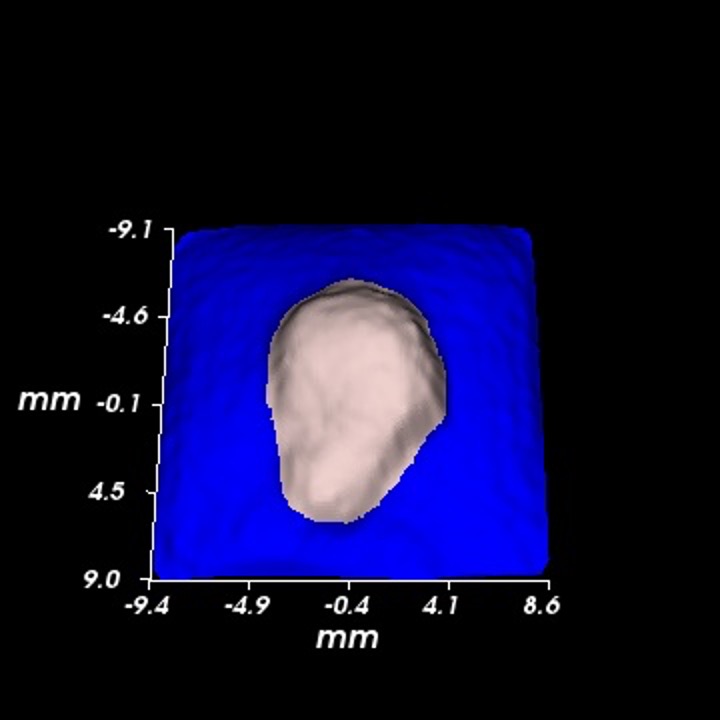

Supplement: Supplementary file 9 — Source data Fig. 2 [file 44321_2026_414_MOESM9_ESM.zip › Fig. 2/Fig. 2H/JHOC5 shC.jpg]

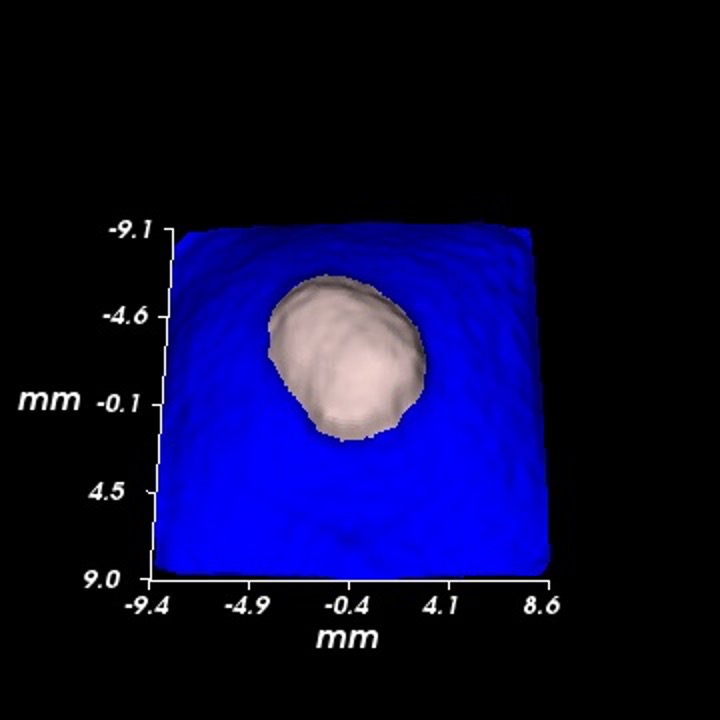

Supplement: Supplementary file 9 — Source data Fig. 2 [file 44321_2026_414_MOESM9_ESM.zip › Fig. 2/Fig. 2I/OVISE #1.jpg]

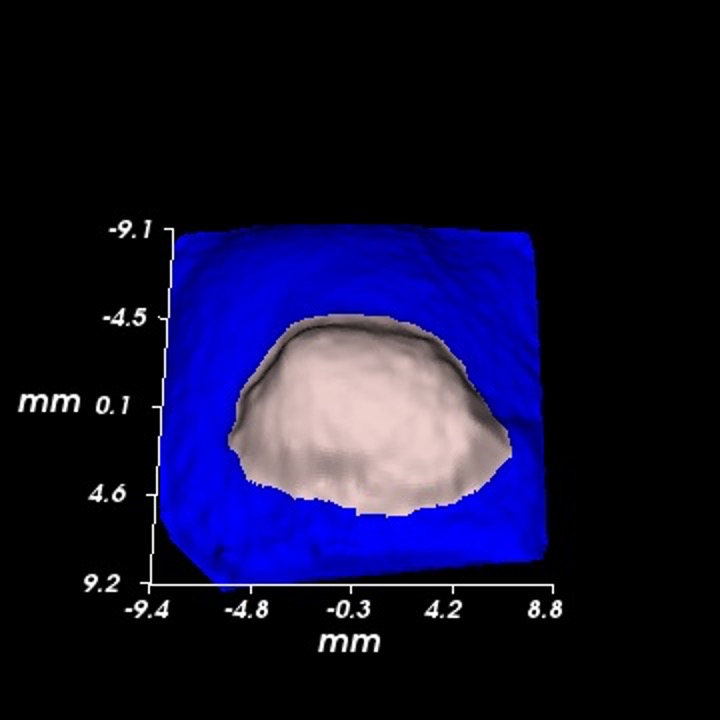

Supplement: Supplementary file 9 — Source data Fig. 2 [file 44321_2026_414_MOESM9_ESM.zip › Fig. 2/Fig. 2I/OVISE shC.jpg]

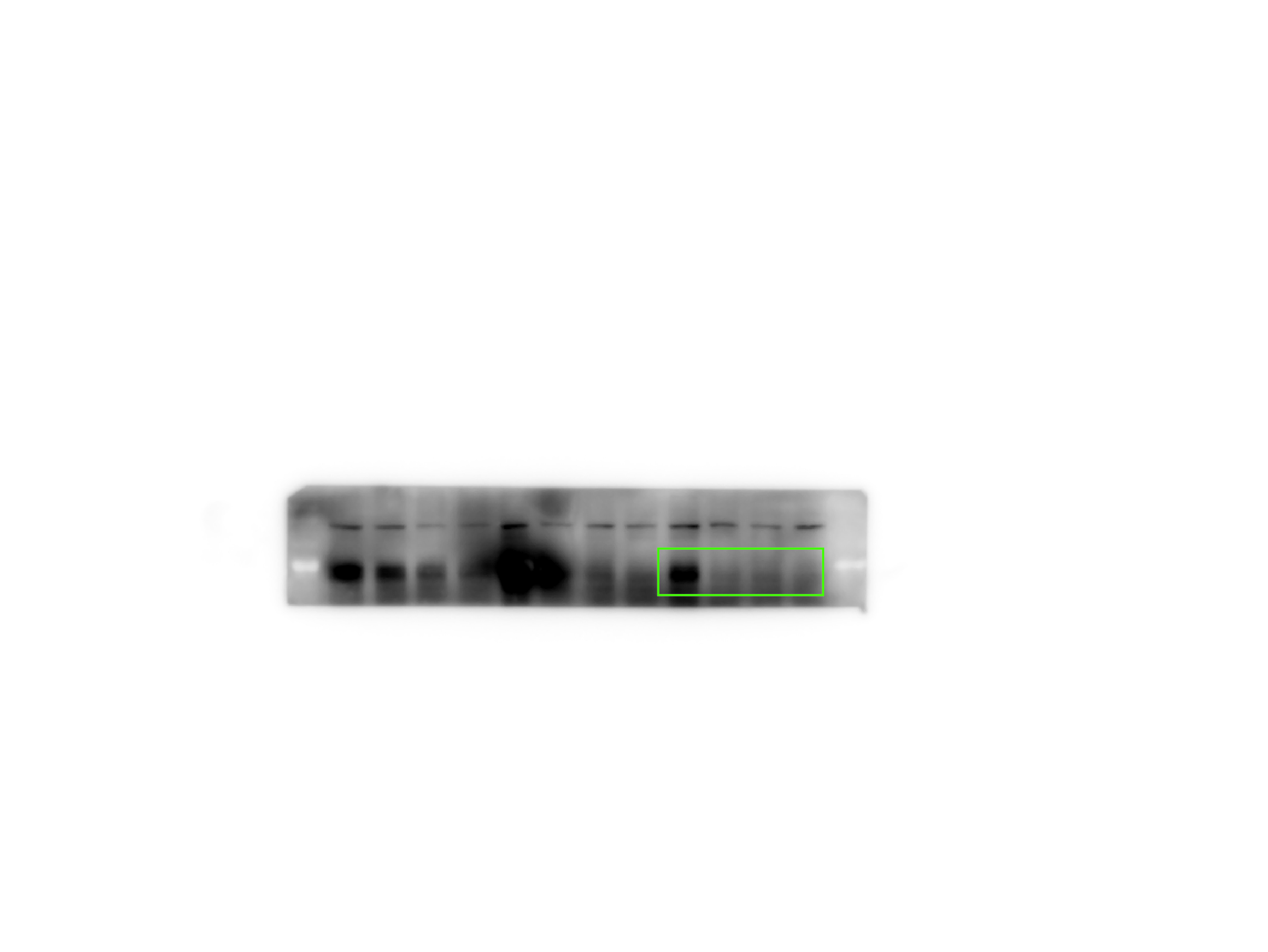

Supplement: Supplementary file 11 — Source data Fig. 4 [file 44321_2026_414_MOESM11_ESM.zip › Fig. 4/Fig. 4B/ES-2 BMAL2 IB.jpg]

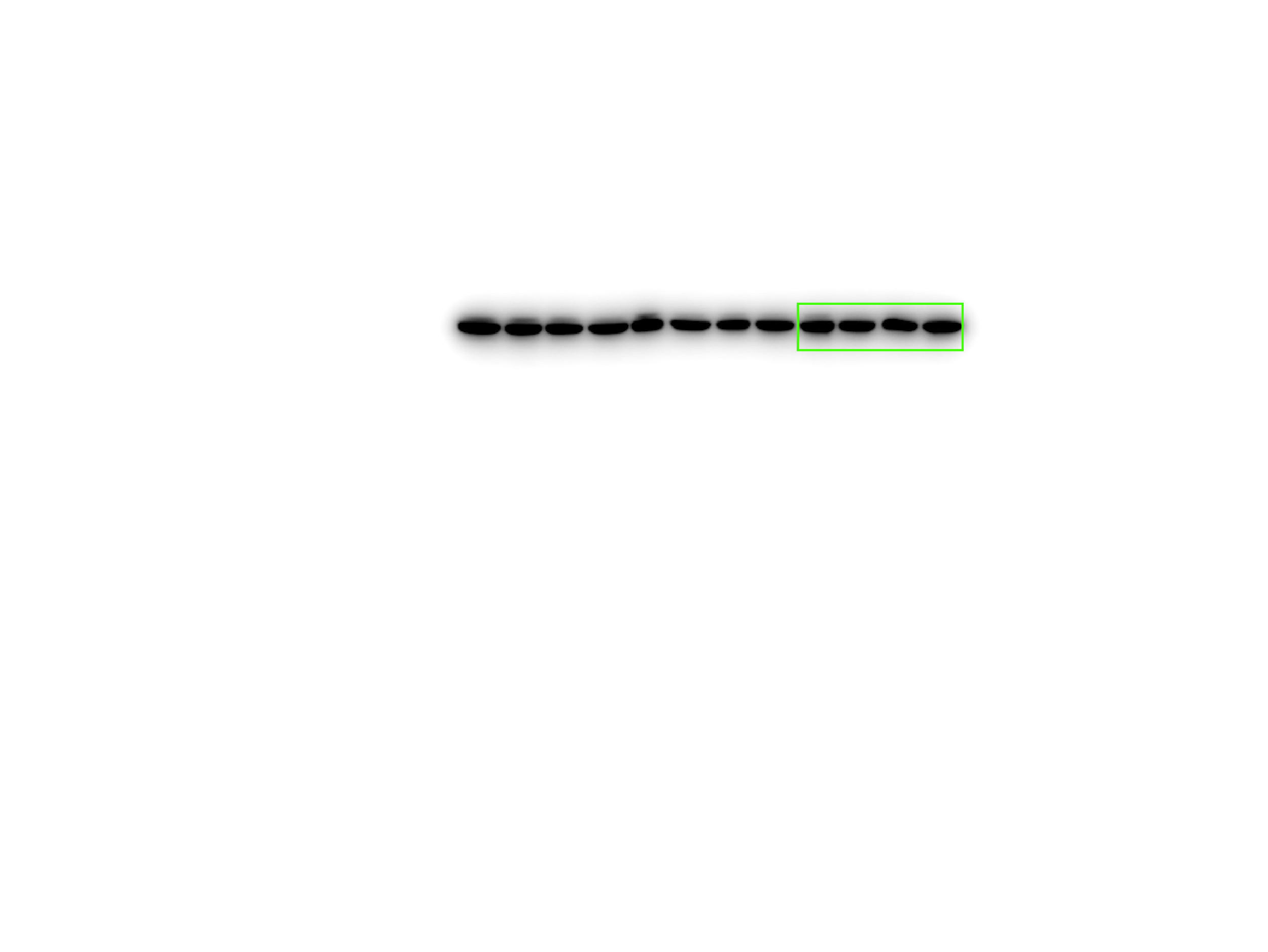

Supplement: Supplementary file 11 — Source data Fig. 4 [file 44321_2026_414_MOESM11_ESM.zip › Fig. 4/Fig. 4B/ES-2 GAPDH IB.jpg]

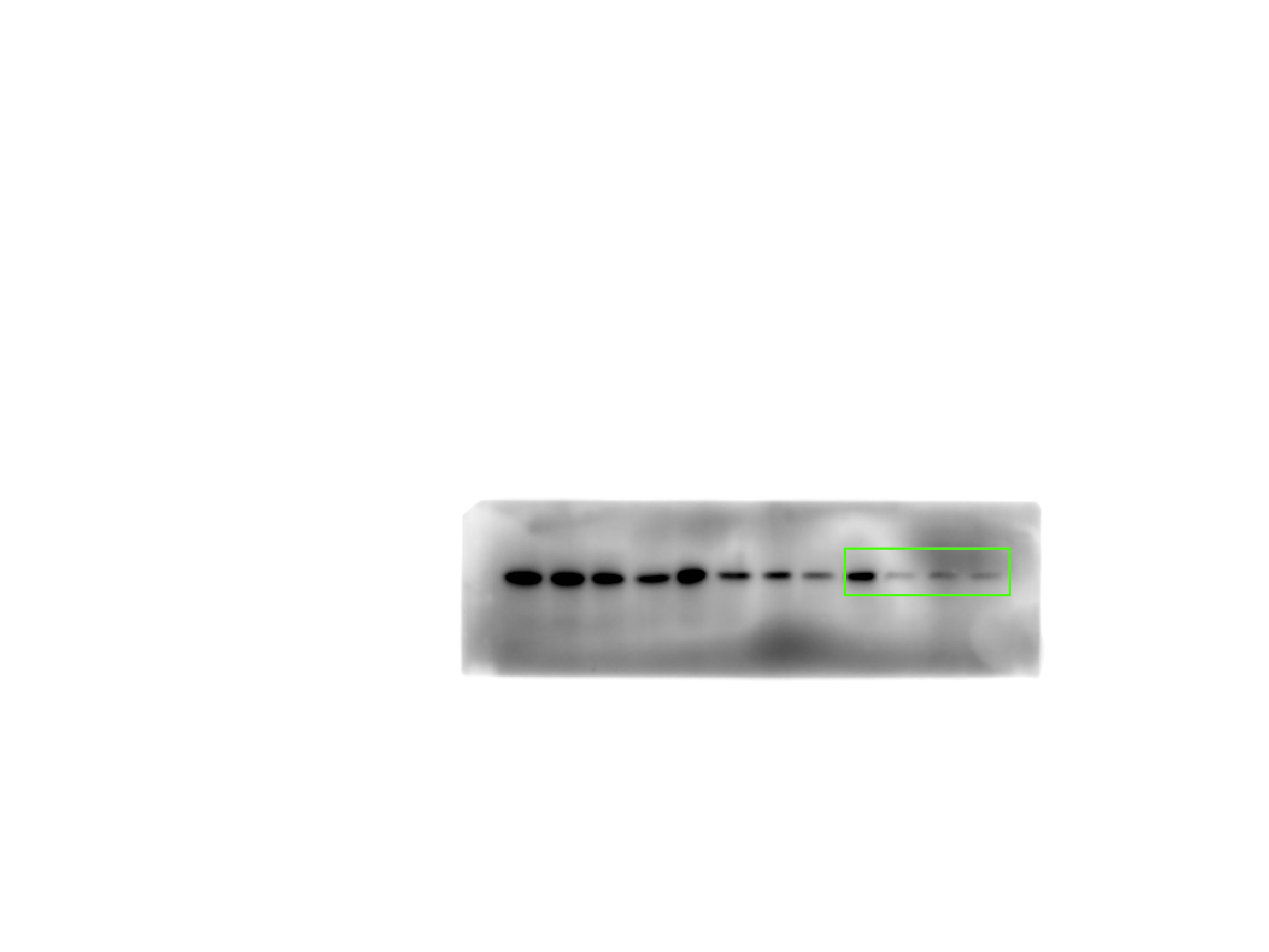

Supplement: Supplementary file 11 — Source data Fig. 4 [file 44321_2026_414_MOESM11_ESM.zip › Fig. 4/Fig. 4B/ES-2 RAD51 IB.jpg]

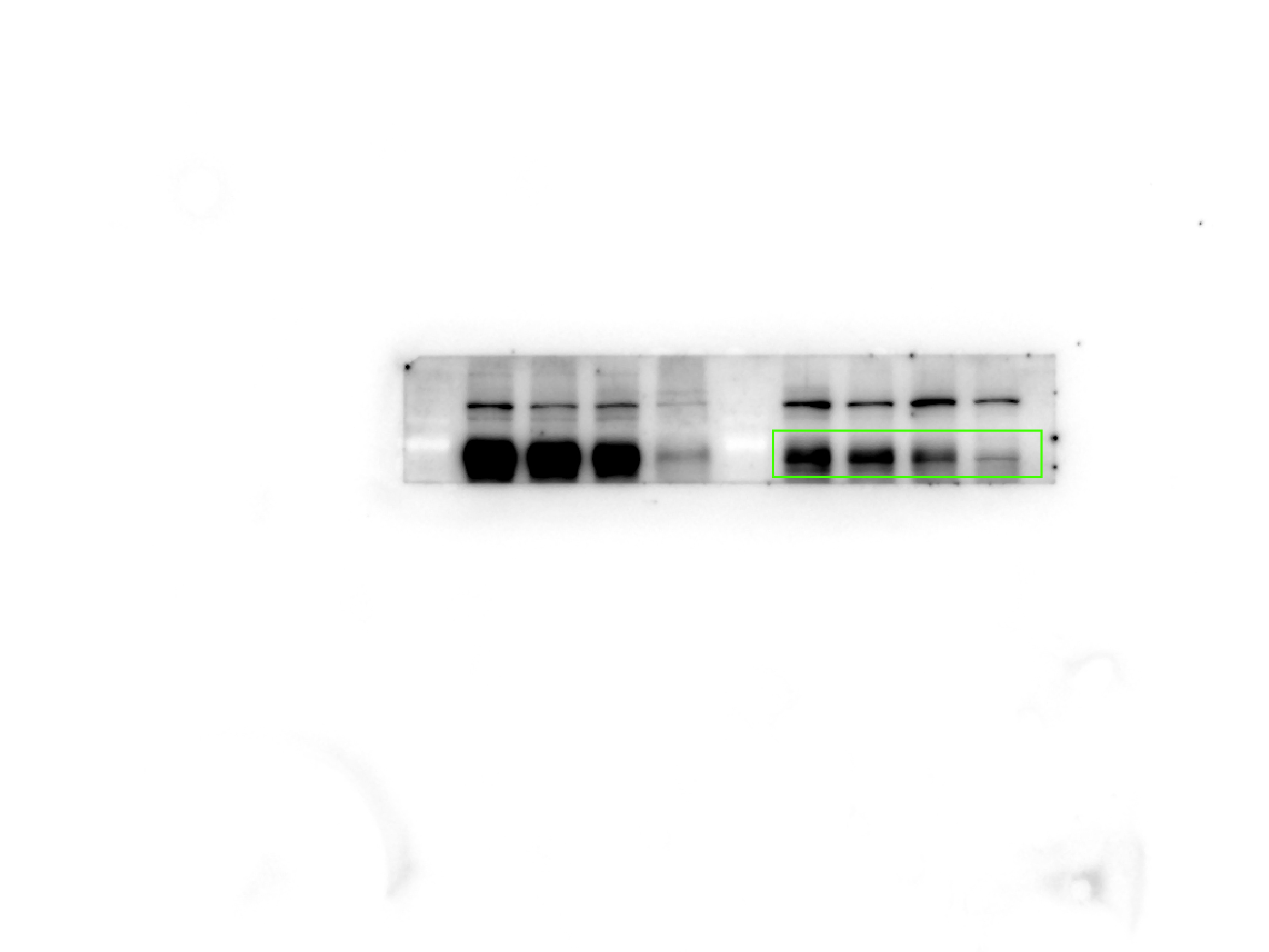

Supplement: Supplementary file 11 — Source data Fig. 4 [file 44321_2026_414_MOESM11_ESM.zip › Fig. 4/Fig. 4B/JHOC5 BMAL2 IB.jpg]

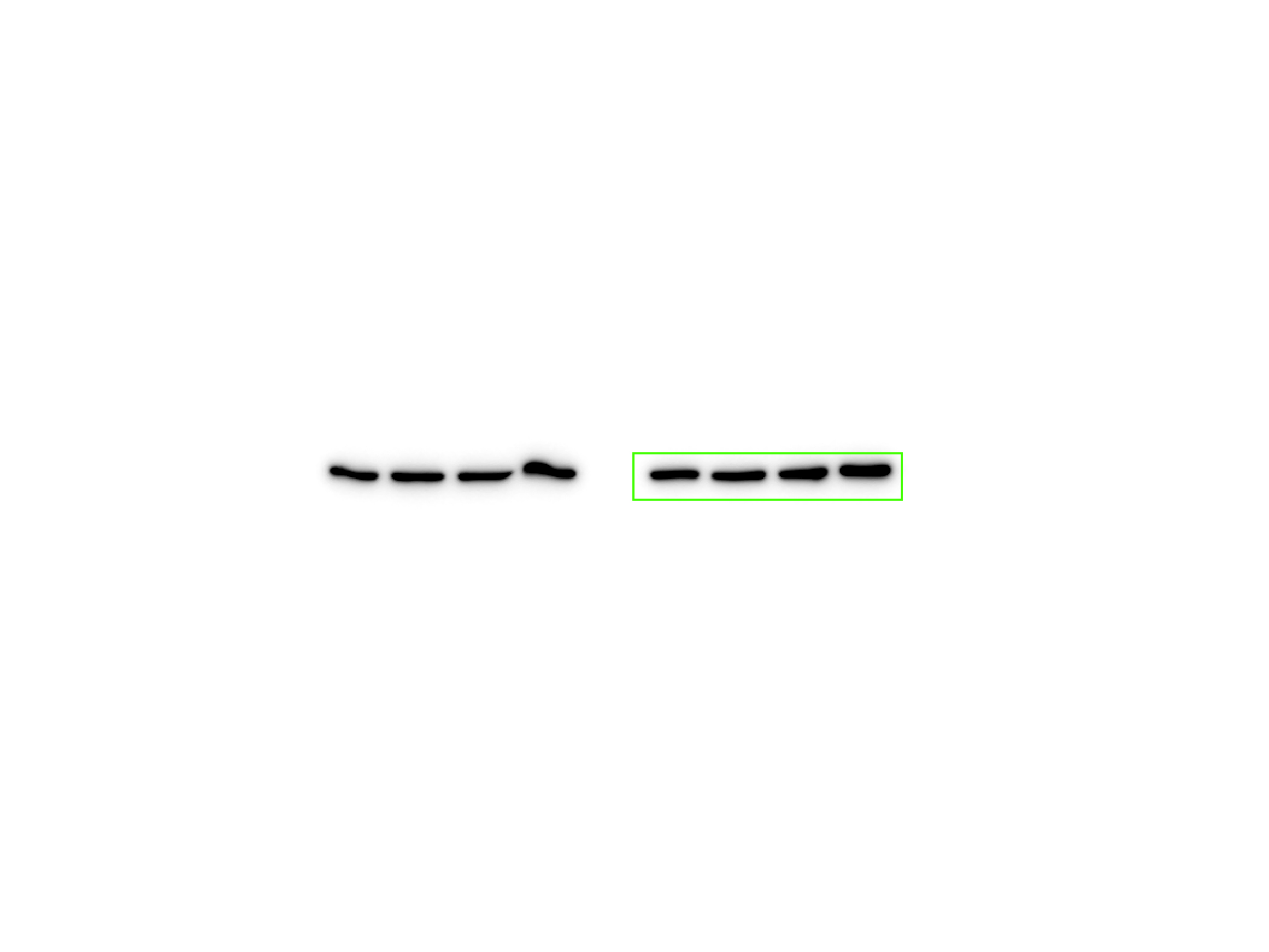

Supplement: Supplementary file 11 — Source data Fig. 4 [file 44321_2026_414_MOESM11_ESM.zip › Fig. 4/Fig. 4B/JHOC5 GAPDH IB.jpg]

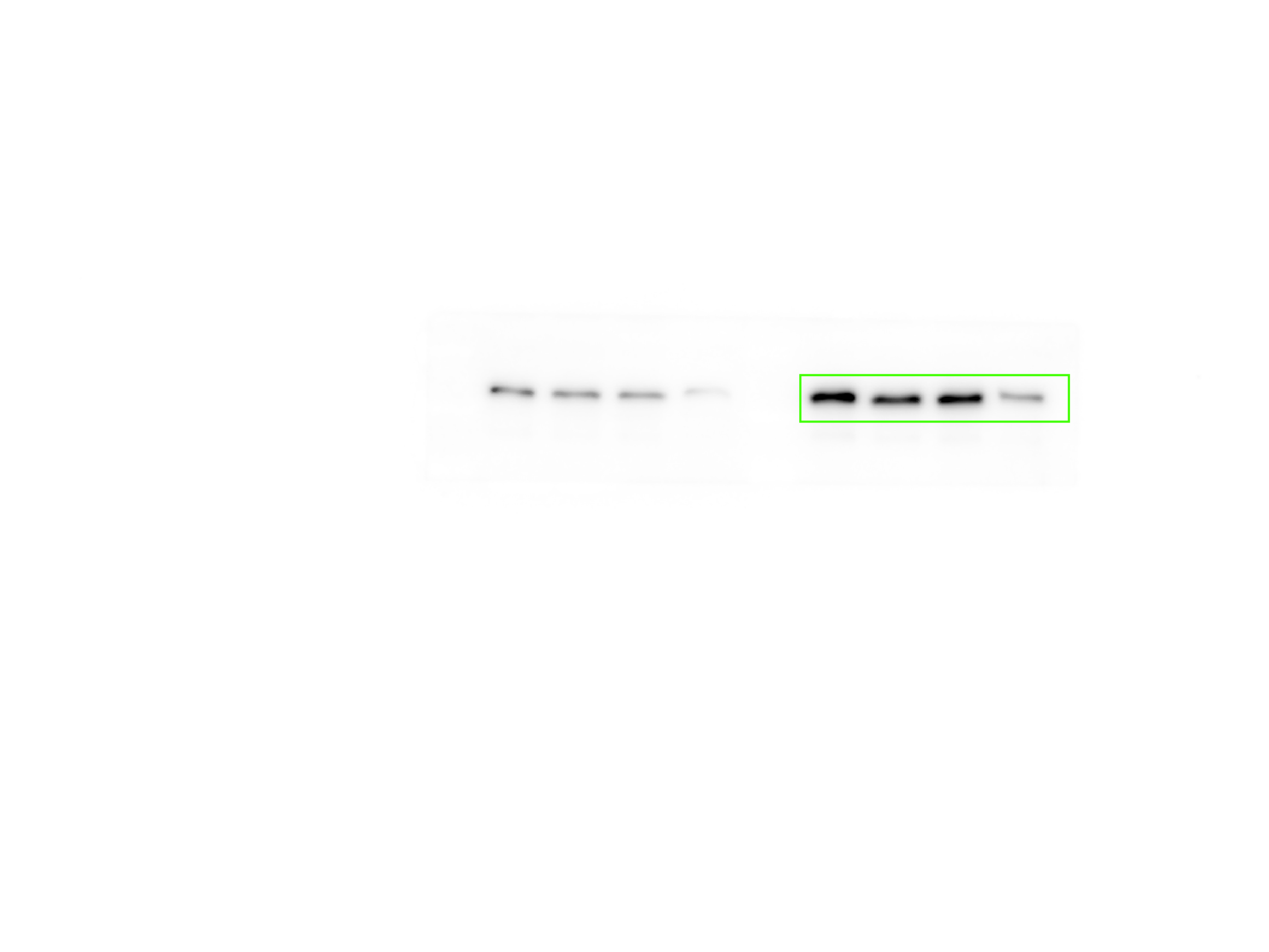

Supplement: Supplementary file 11 — Source data Fig. 4 [file 44321_2026_414_MOESM11_ESM.zip › Fig. 4/Fig. 4B/JHOC5 RAD51 IB.jpg]

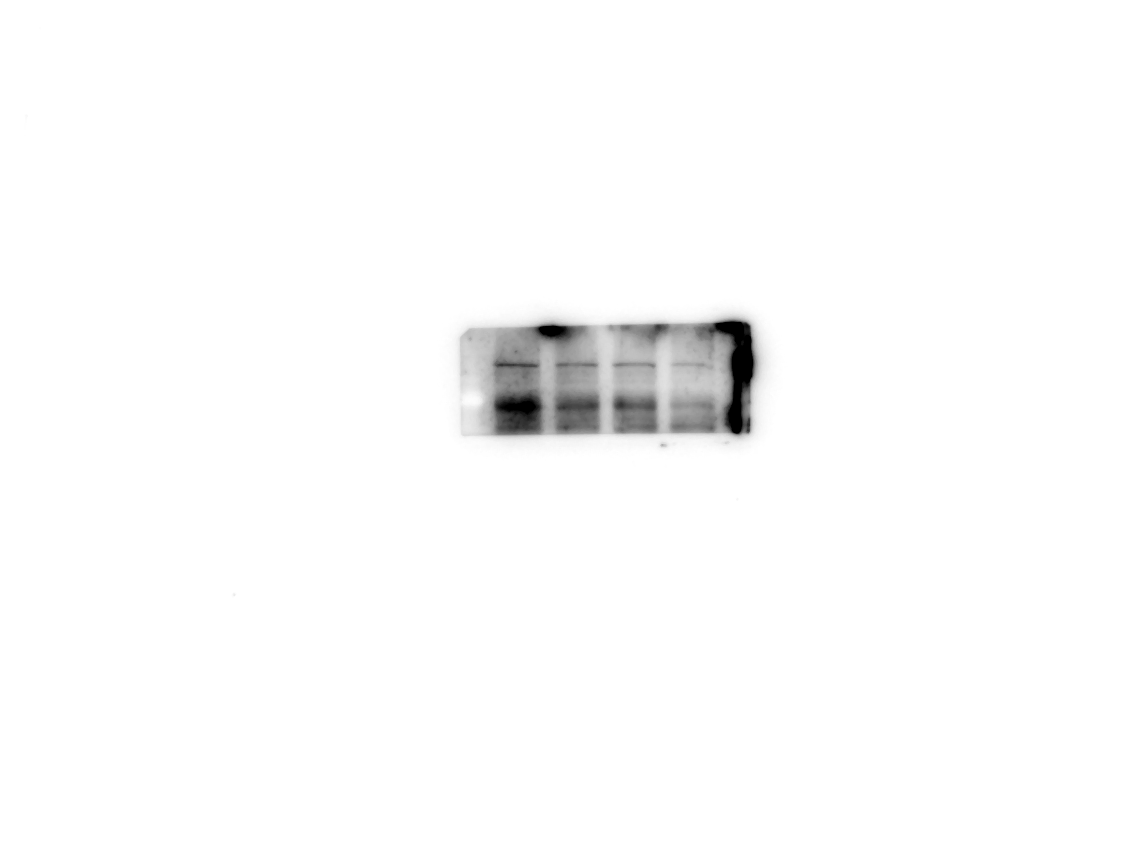

Supplement: Supplementary file 11 — Source data Fig. 4 [file 44321_2026_414_MOESM11_ESM.zip › Fig. 4/Fig. 4B/JHOC9 BMAL2 IB .tif]

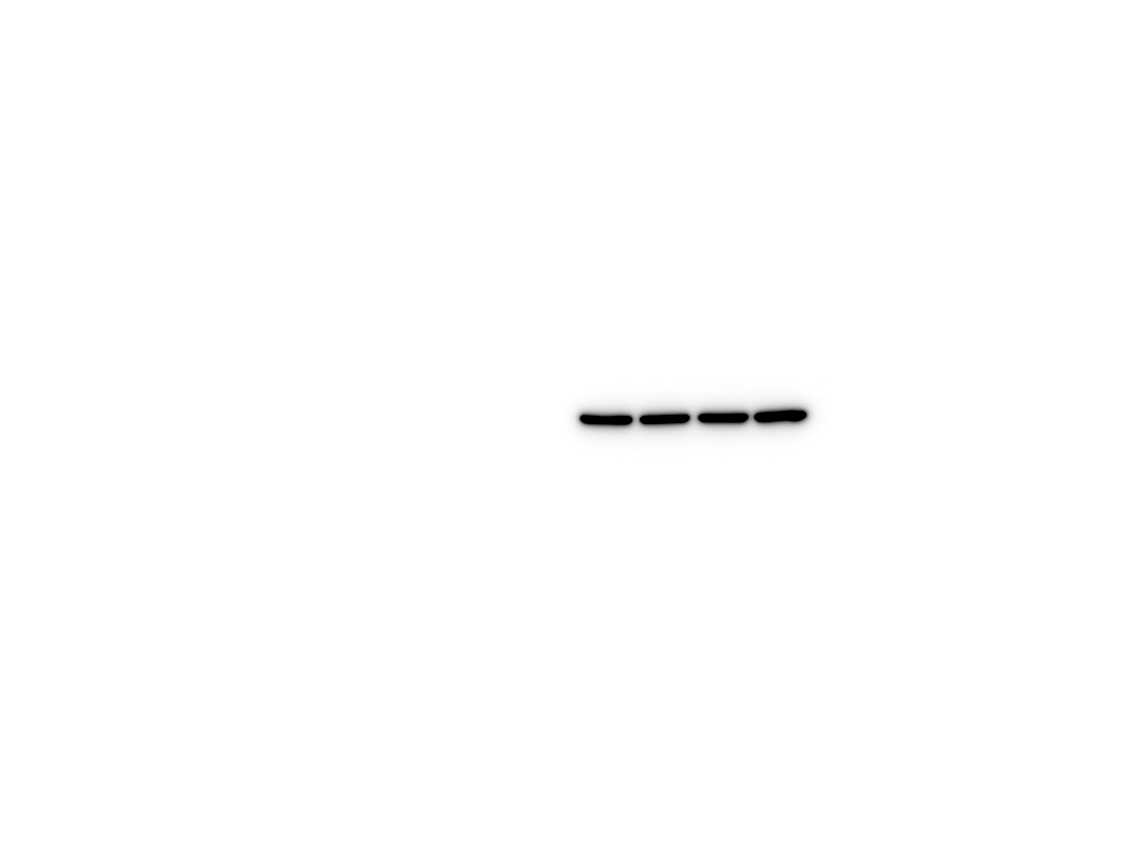

Supplement: Supplementary file 11 — Source data Fig. 4 [file 44321_2026_414_MOESM11_ESM.zip › Fig. 4/Fig. 4B/JHOC9 GAPDH IB.tif]

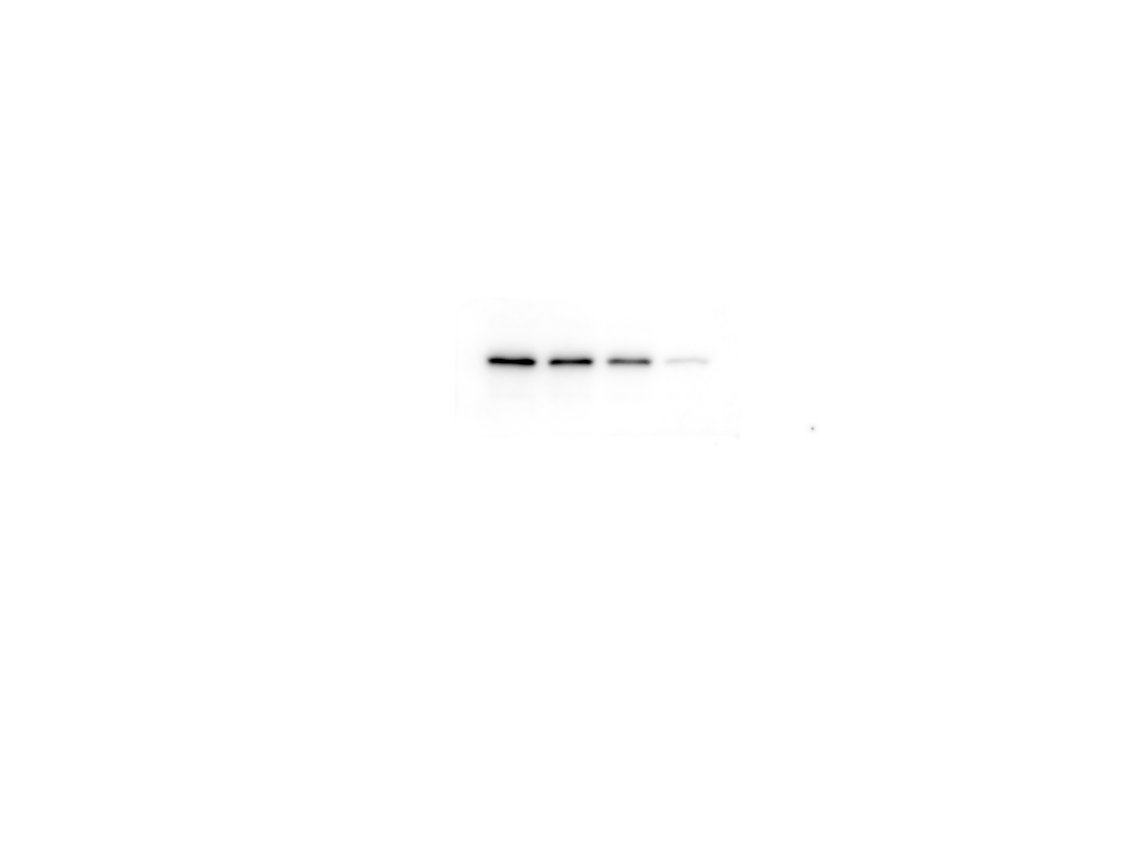

Supplement: Supplementary file 11 — Source data Fig. 4 [file 44321_2026_414_MOESM11_ESM.zip › Fig. 4/Fig. 4B/JHOC9 RAD51 IB .tif]

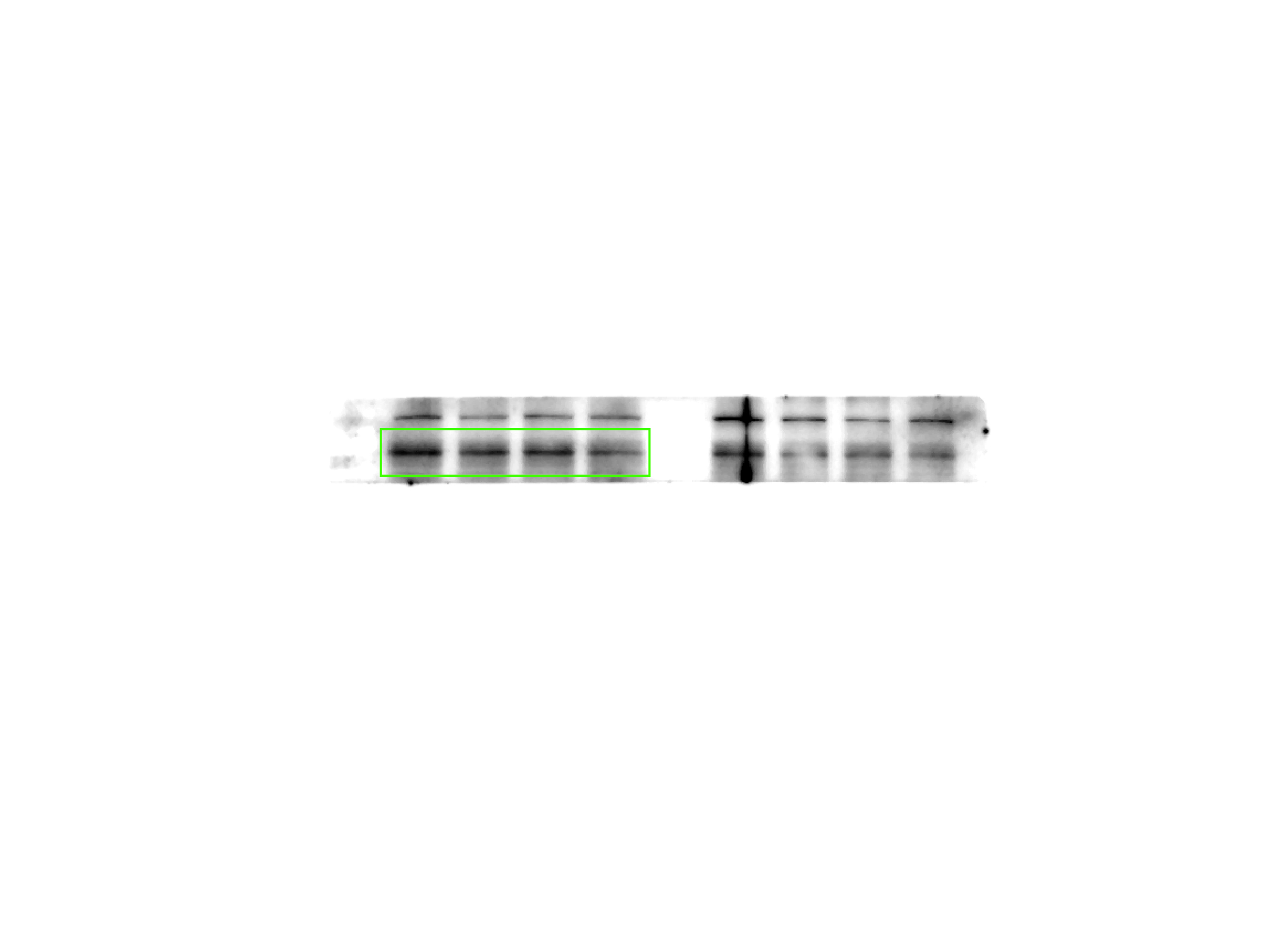

Supplement: Supplementary file 11 — Source data Fig. 4 [file 44321_2026_414_MOESM11_ESM.zip › Fig. 4/Fig. 4B/OVCA429 BMAL2 IB.jpg]

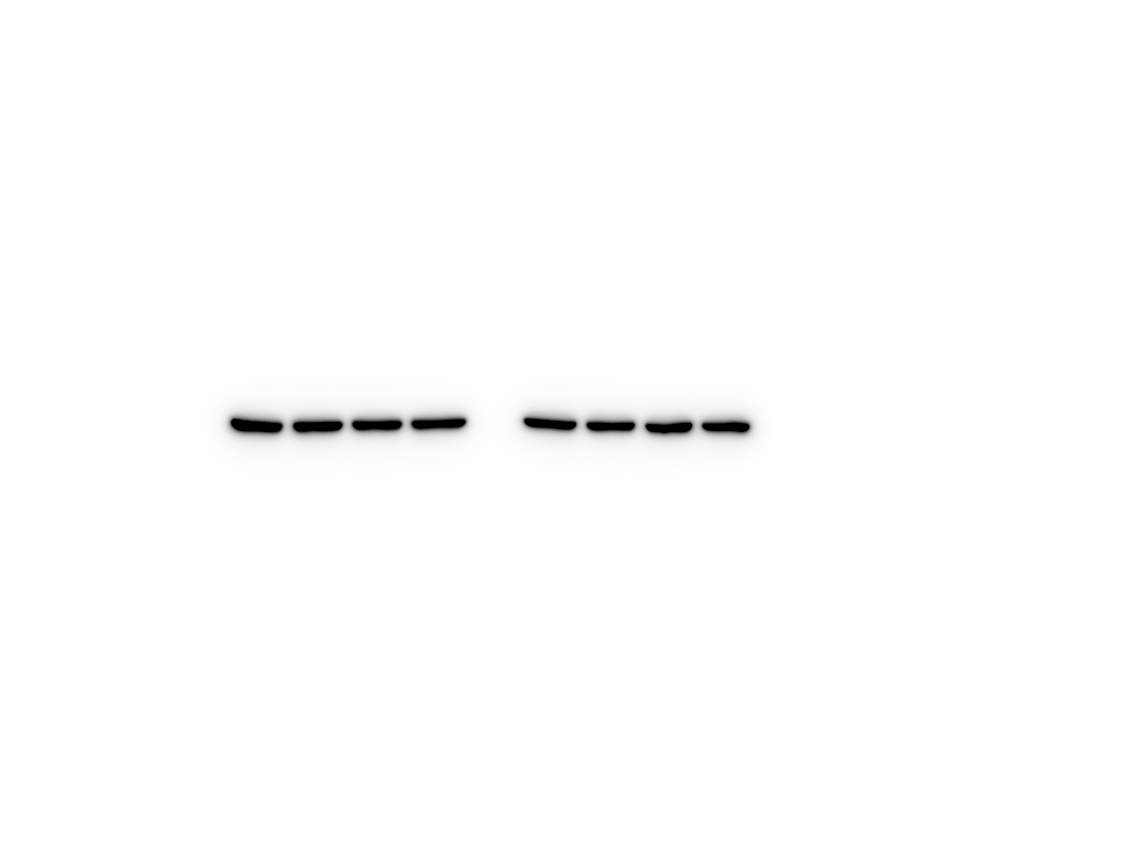

Supplement: Supplementary file 11 — Source data Fig. 4 [file 44321_2026_414_MOESM11_ESM.zip › Fig. 4/Fig. 4B/OVCA429 GAPDH IB .tif]

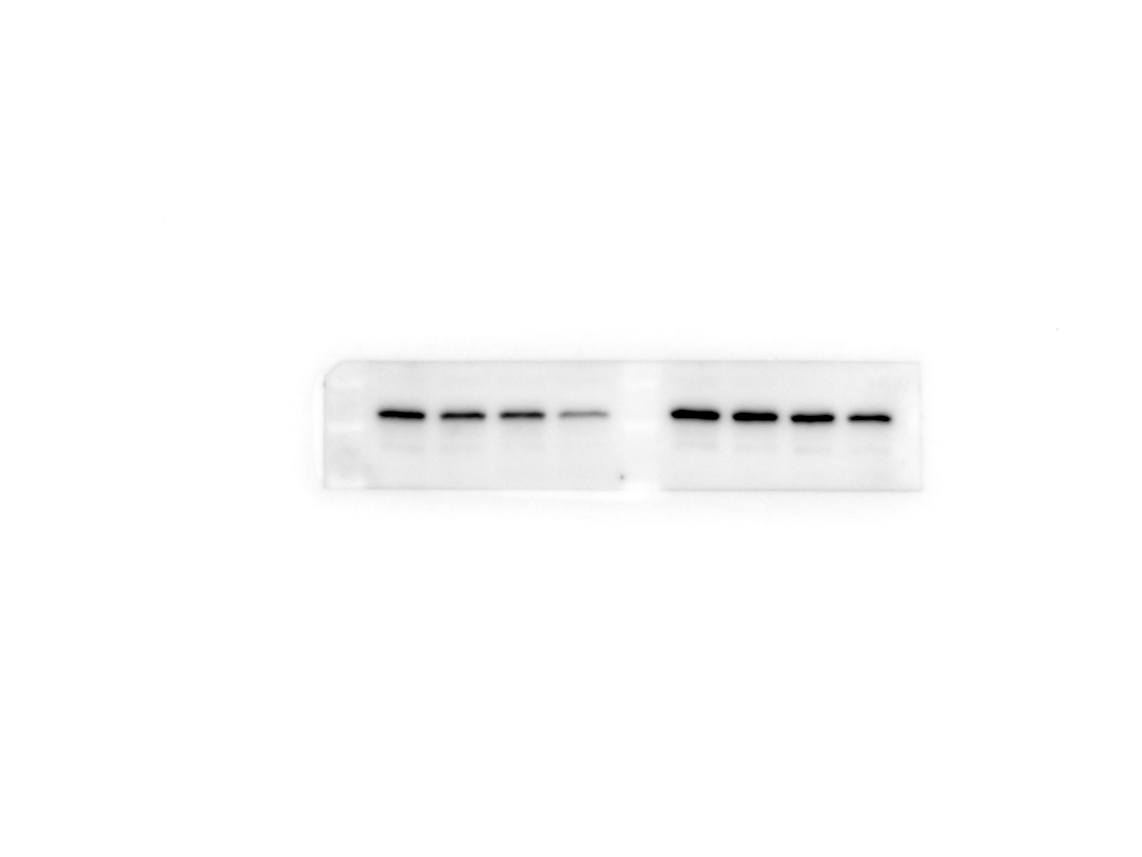

Supplement: Supplementary file 11 — Source data Fig. 4 [file 44321_2026_414_MOESM11_ESM.zip › Fig. 4/Fig. 4B/OVCA429 RAD51 IB.tif]

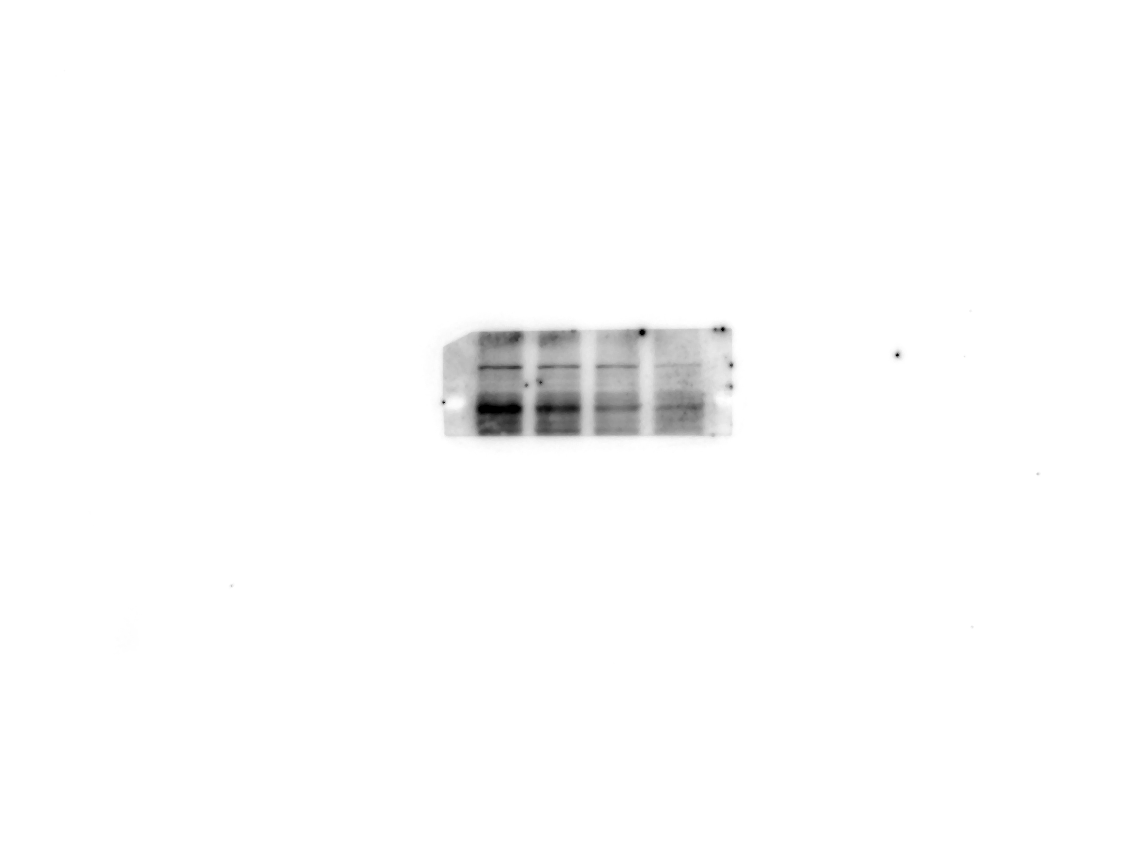

Supplement: Supplementary file 11 — Source data Fig. 4 [file 44321_2026_414_MOESM11_ESM.zip › Fig. 4/Fig. 4B/OVISE BMAL2 IB .tif]

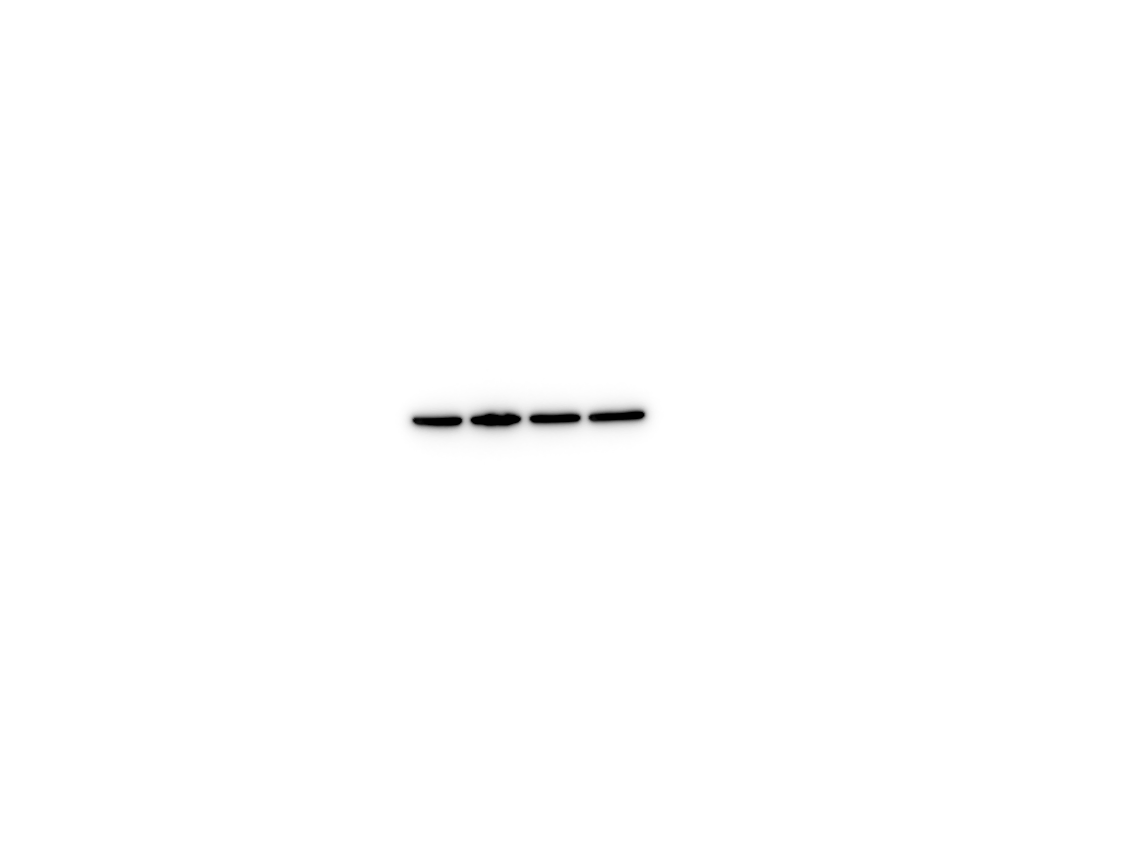

Supplement: Supplementary file 11 — Source data Fig. 4 [file 44321_2026_414_MOESM11_ESM.zip › Fig. 4/Fig. 4B/OVISE GAPDH IB.tif]

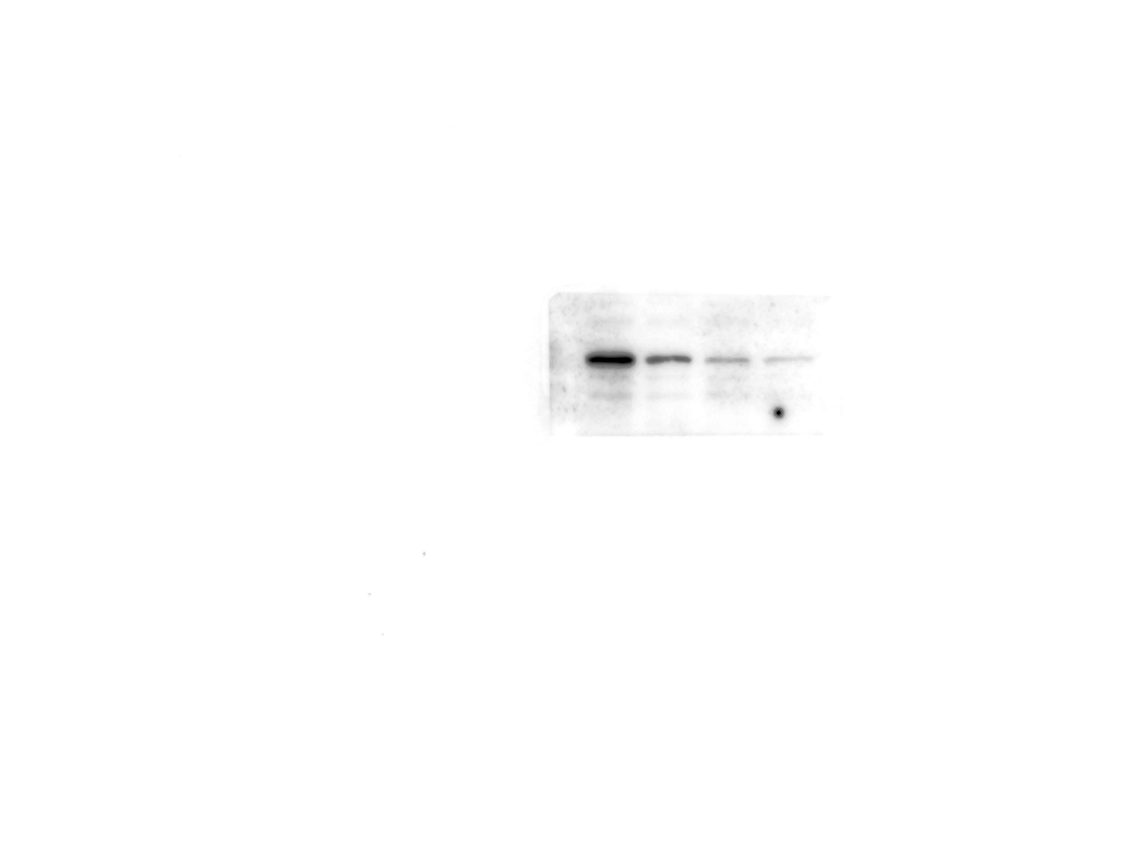

Supplement: Supplementary file 11 — Source data Fig. 4 [file 44321_2026_414_MOESM11_ESM.zip › Fig. 4/Fig. 4B/OVISE RAD51 IB.tif]

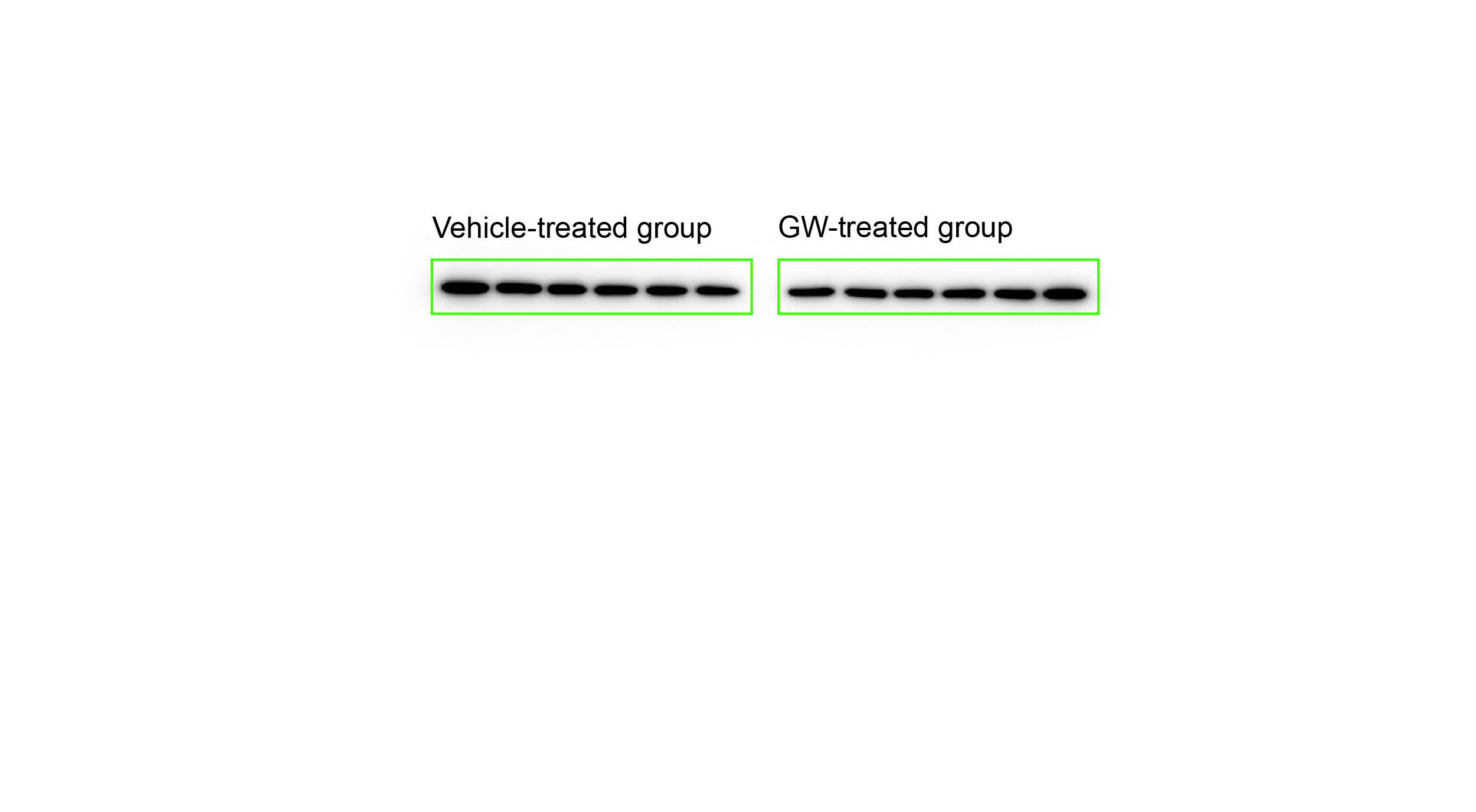

Supplement: Supplementary file 11 — Source data Fig. 4 [file 44321_2026_414_MOESM11_ESM.zip › Fig. 4/Fig. 4C/ES-2 GAPDH IB.jpg]

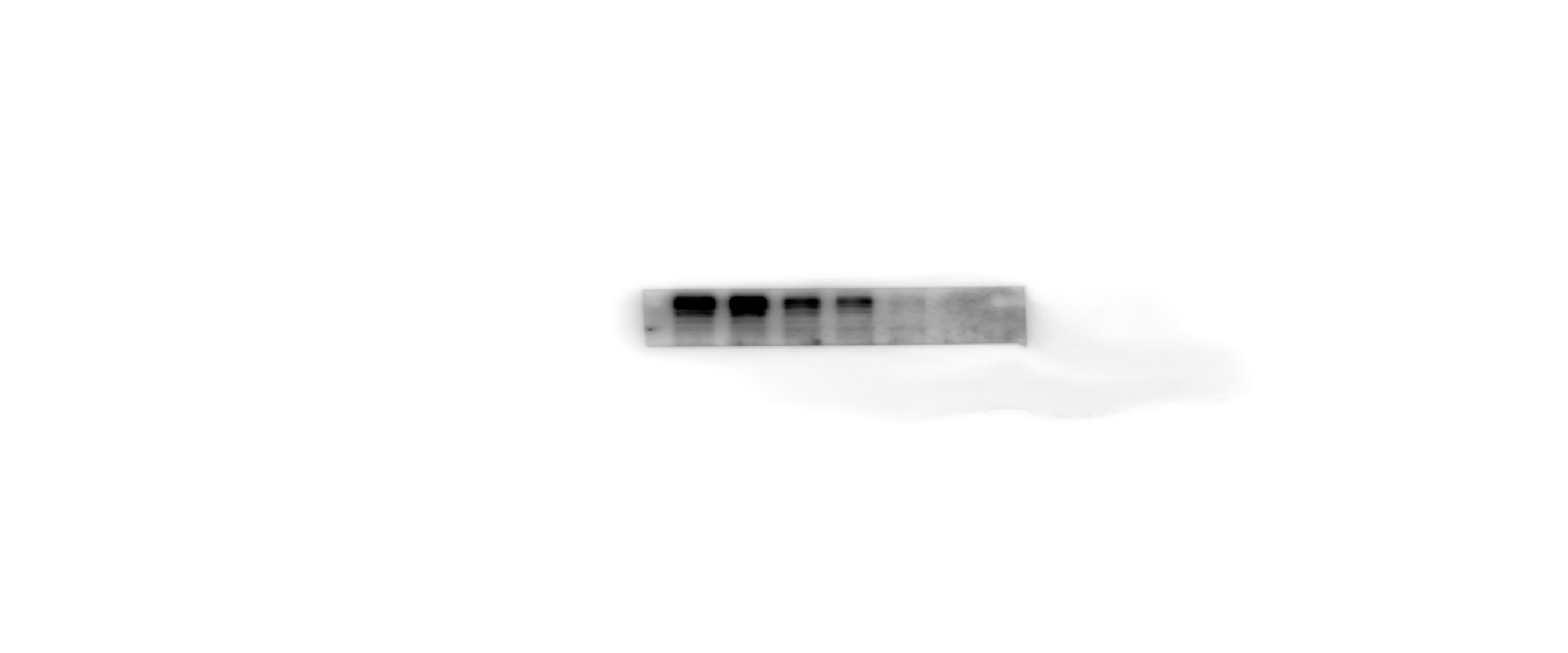

Supplement: Supplementary file 11 — Source data Fig. 4 [file 44321_2026_414_MOESM11_ESM.zip › Fig. 4/Fig. 4C/ES-2 GW BMAL2 IB.jpg]

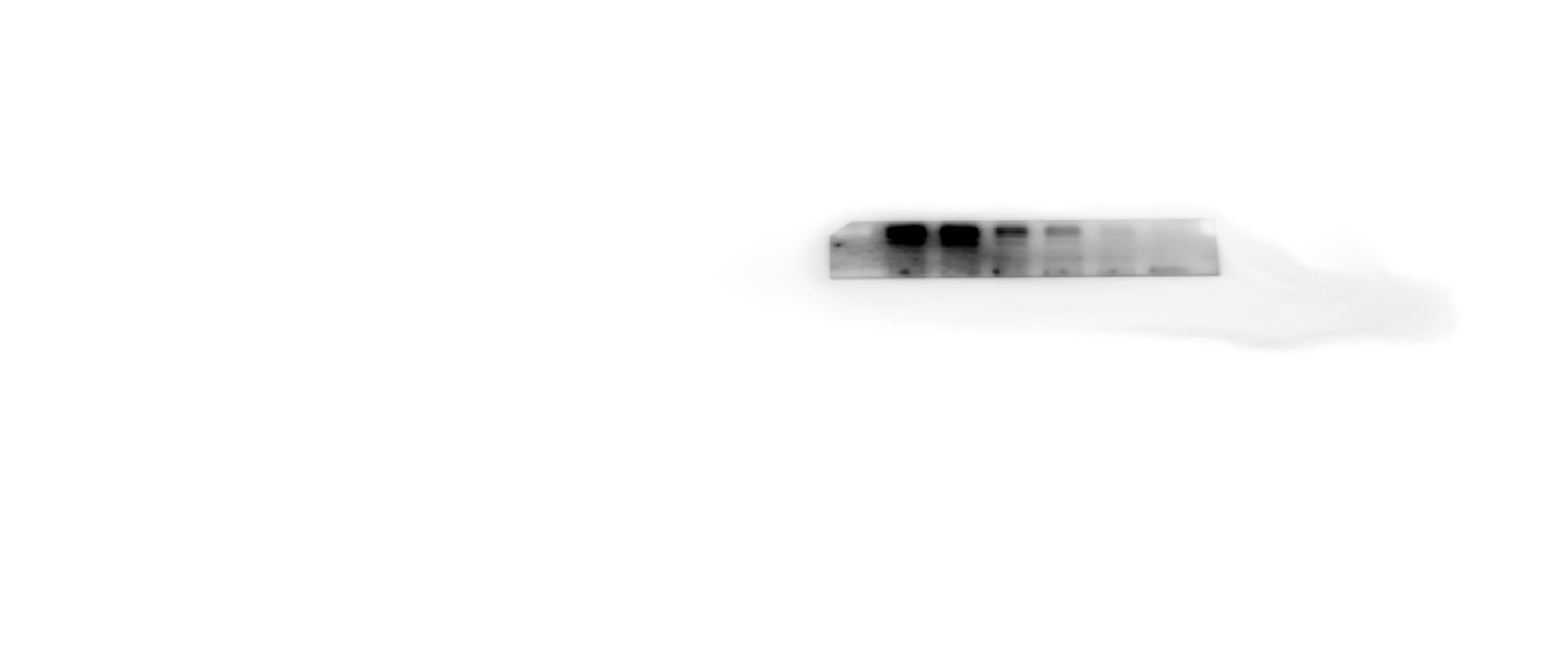

Supplement: Supplementary file 11 — Source data Fig. 4 [file 44321_2026_414_MOESM11_ESM.zip › Fig. 4/Fig. 4C/ES-2 Vehicle BMAL2 IB.jpg]

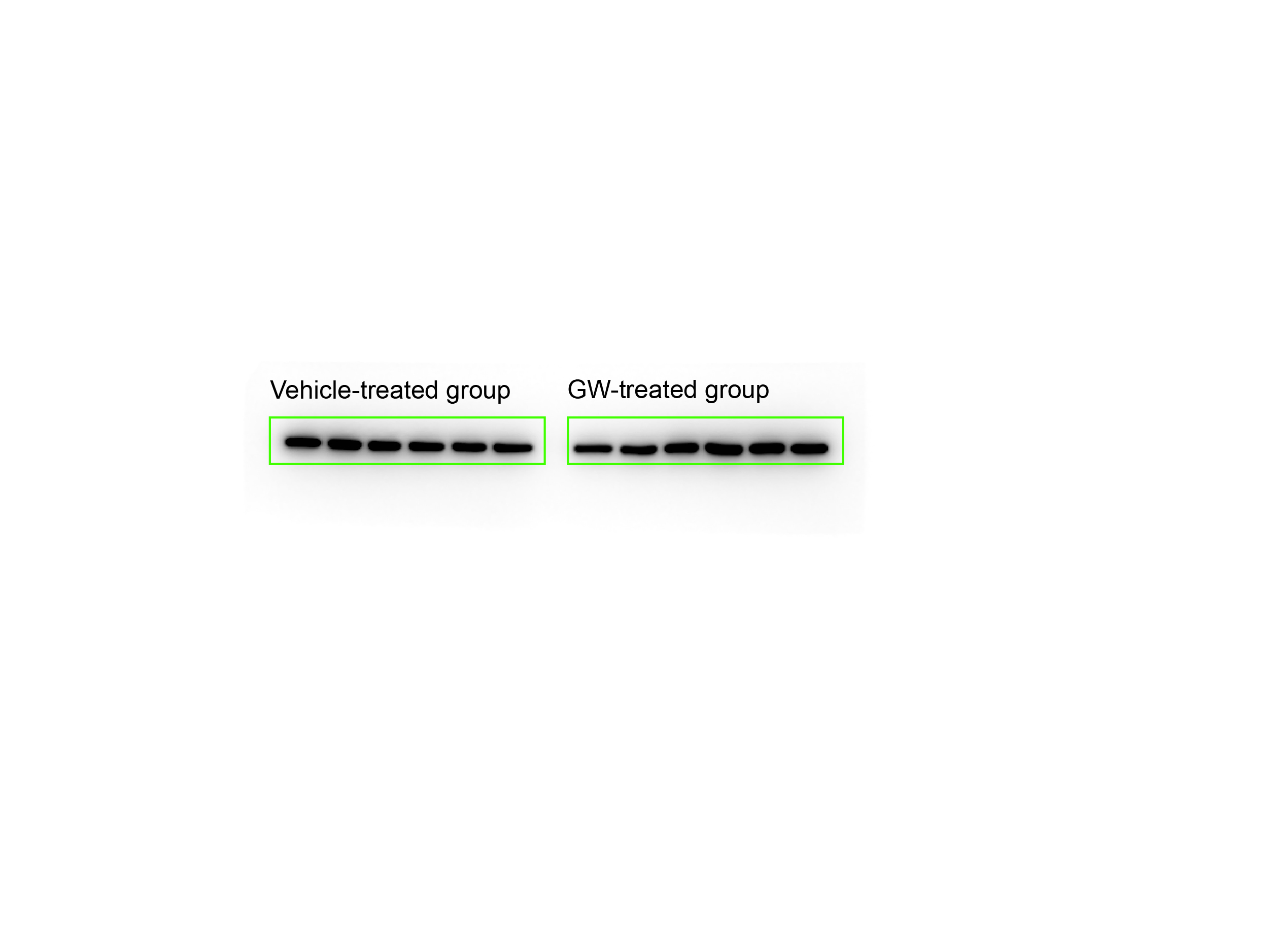

Supplement: Supplementary file 11 — Source data Fig. 4 [file 44321_2026_414_MOESM11_ESM.zip › Fig. 4/Fig. 4C/JHOC5 GAPDH.jpg]

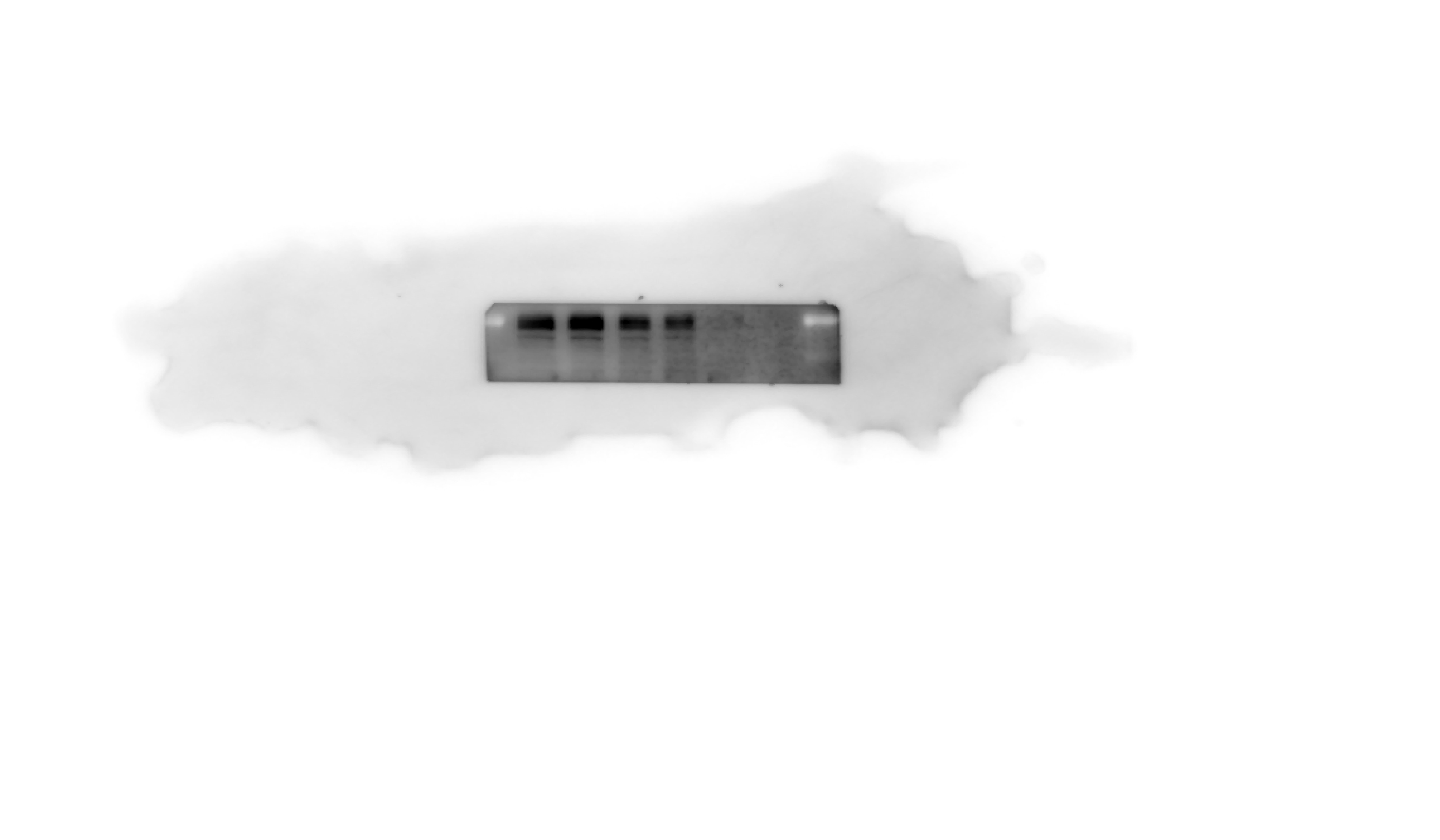

Supplement: Supplementary file 11 — Source data Fig. 4 [file 44321_2026_414_MOESM11_ESM.zip › Fig. 4/Fig. 4C/JHOC5 GW BMAL2.jpg]

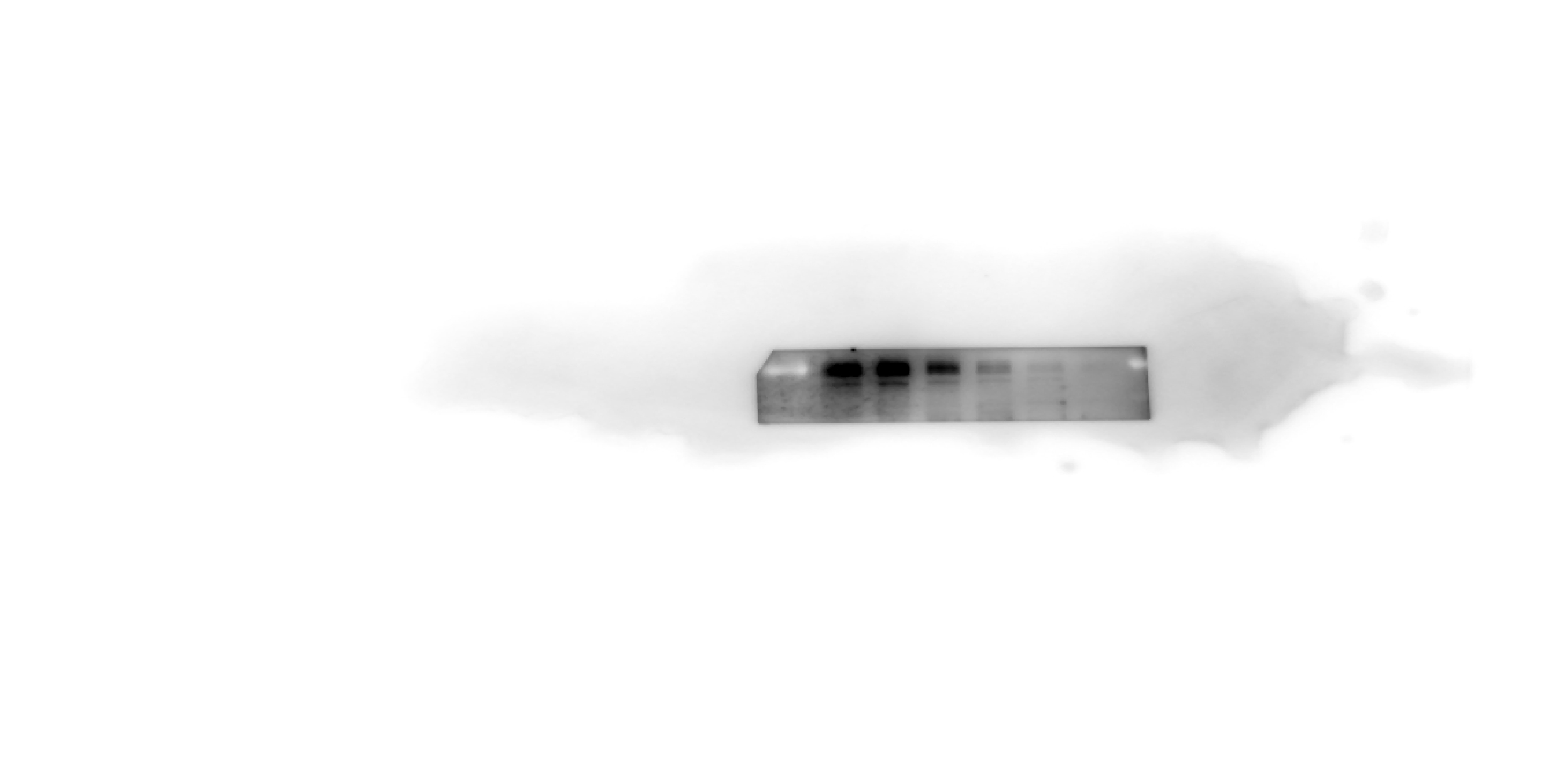

Supplement: Supplementary file 11 — Source data Fig. 4 [file 44321_2026_414_MOESM11_ESM.zip › Fig. 4/Fig. 4C/JHOC5 Vehicle BMAL2 IB.jpg]

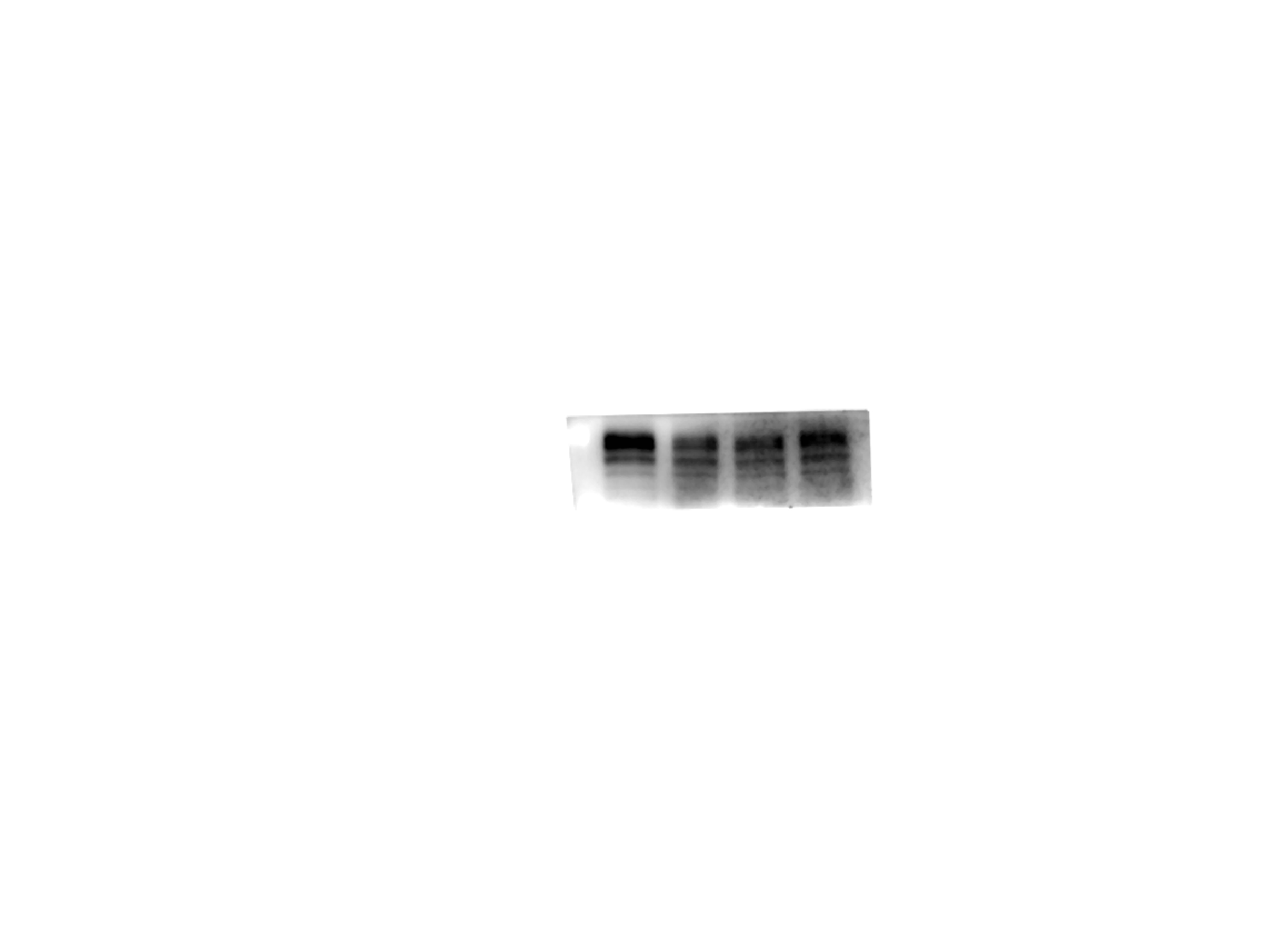

Supplement: Supplementary file 11 — Source data Fig. 4 [file 44321_2026_414_MOESM11_ESM.zip › Fig. 4/Fig. 4D/ES-2 BMAL2 IB.jpg]

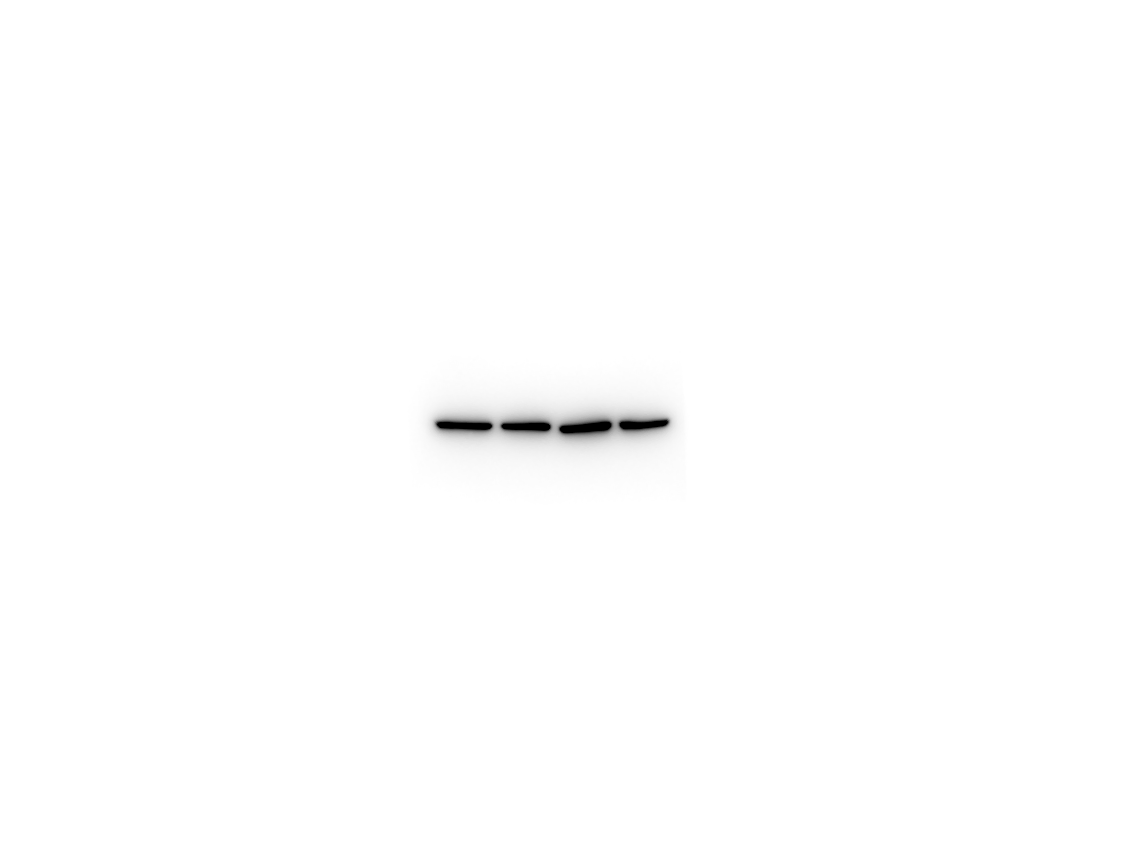

Supplement: Supplementary file 11 — Source data Fig. 4 [file 44321_2026_414_MOESM11_ESM.zip › Fig. 4/Fig. 4D/ES-2 GAPDH IB.tif]

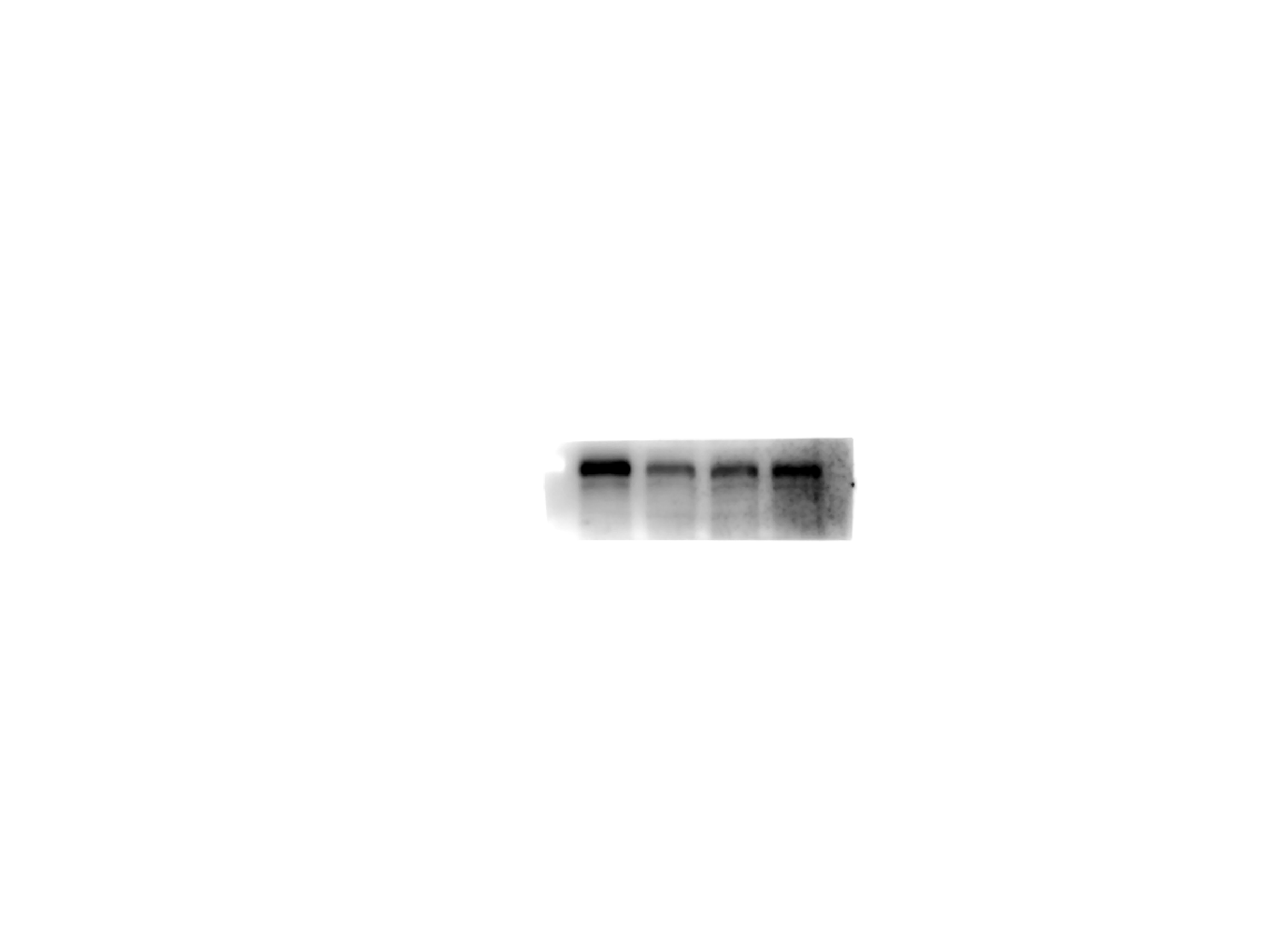

Supplement: Supplementary file 11 — Source data Fig. 4 [file 44321_2026_414_MOESM11_ESM.zip › Fig. 4/Fig. 4D/JHOC5 BMAL2 IB.jpg]

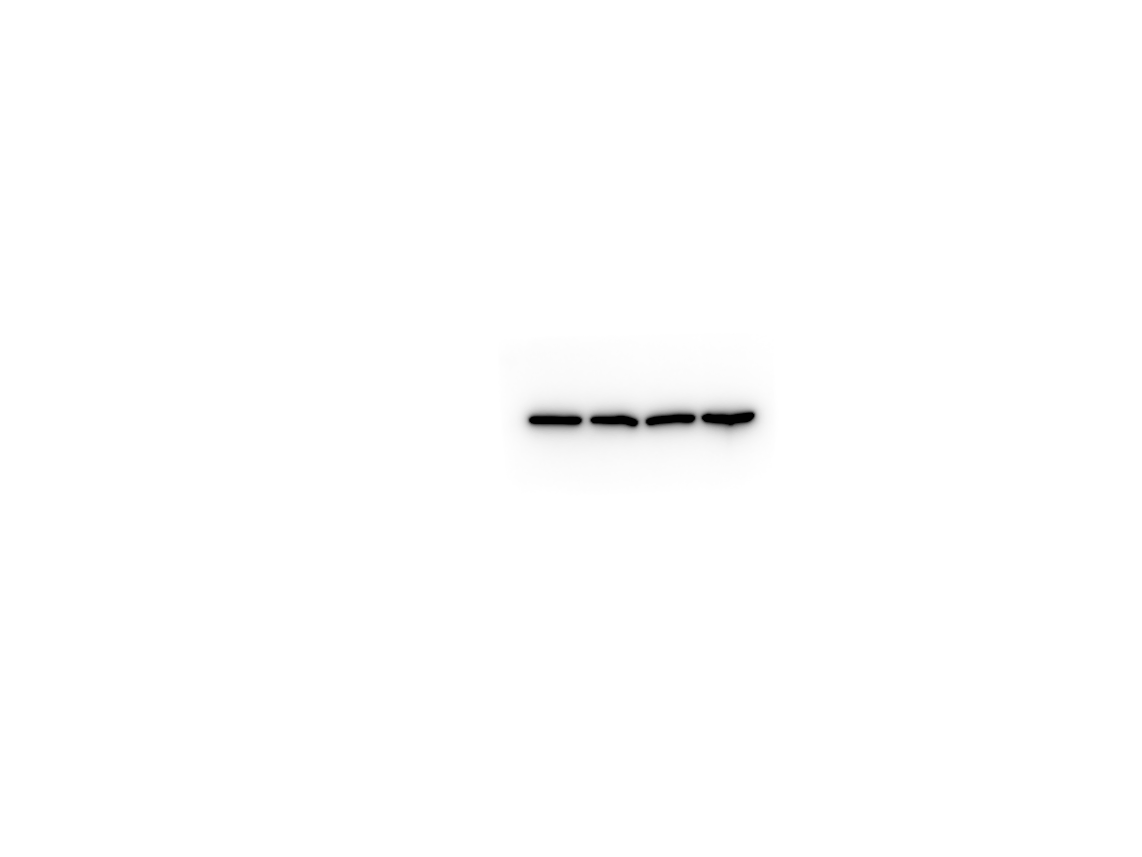

Supplement: Supplementary file 11 — Source data Fig. 4 [file 44321_2026_414_MOESM11_ESM.zip › Fig. 4/Fig. 4D/JHOC5 GAPDH IB.tif]

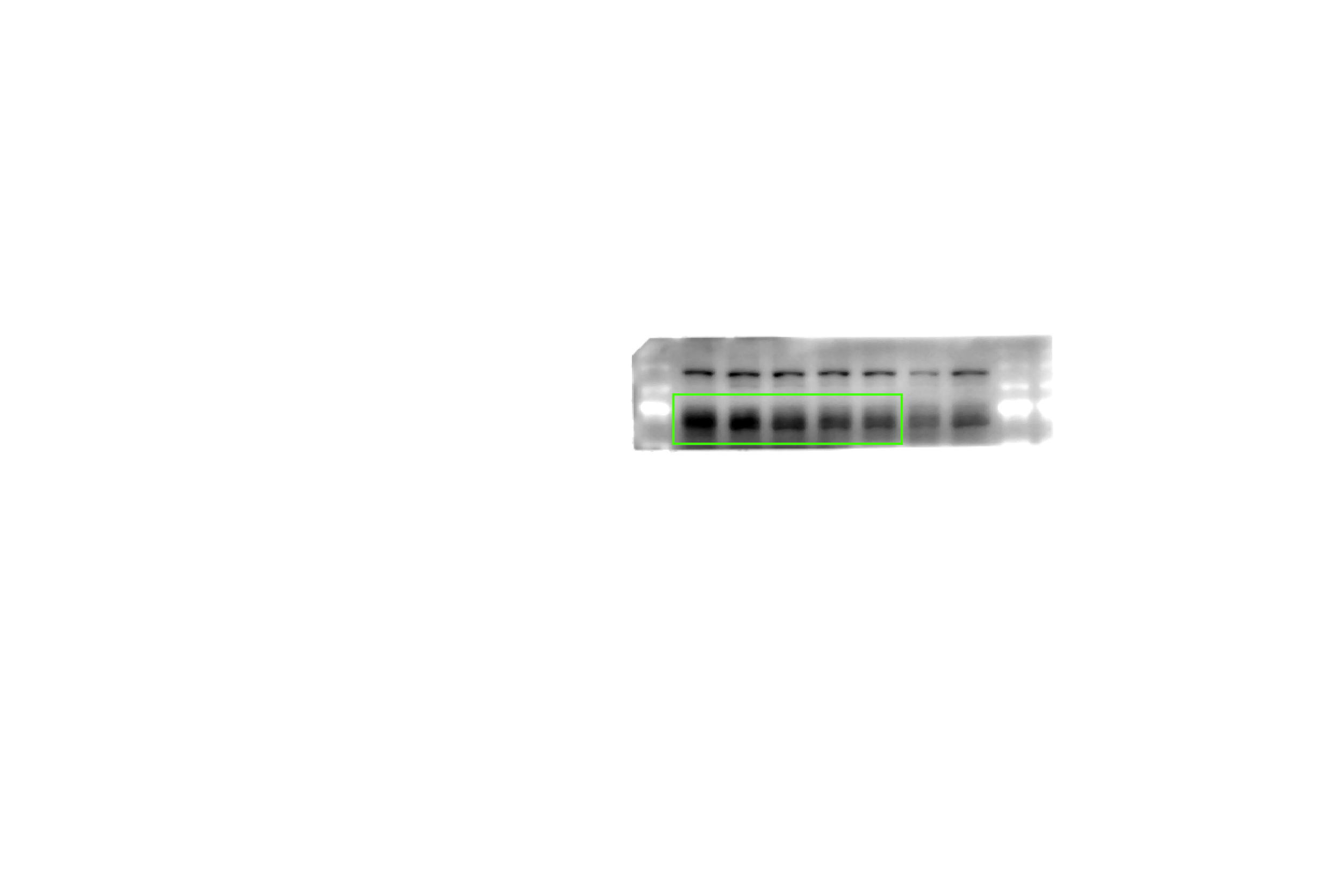

Supplement: Supplementary file 11 — Source data Fig. 4 [file 44321_2026_414_MOESM11_ESM.zip › Fig. 4/Fig. 4E/ES-2 GW-treated group BMAL2 IB.jpg]

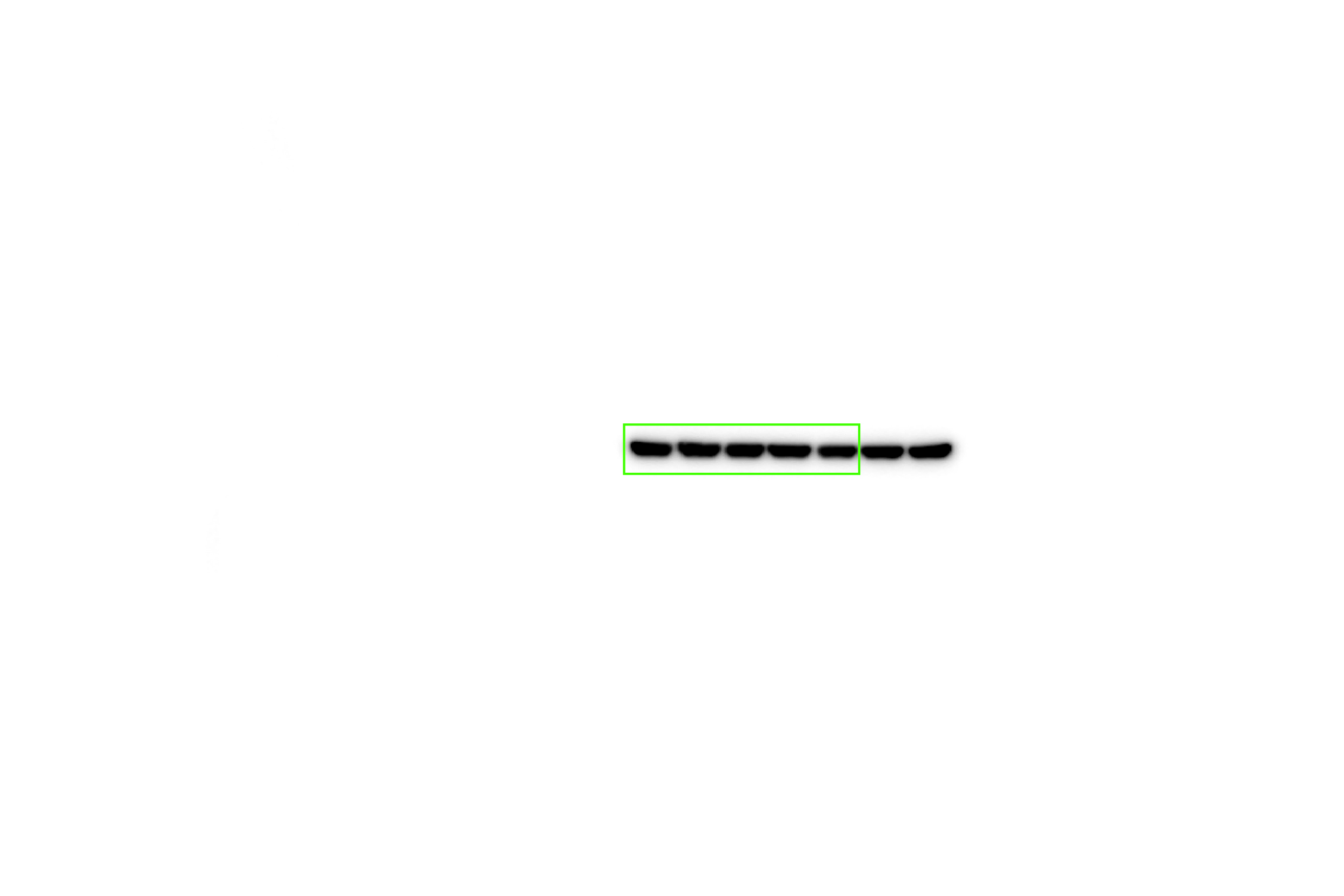

Supplement: Supplementary file 11 — Source data Fig. 4 [file 44321_2026_414_MOESM11_ESM.zip › Fig. 4/Fig. 4E/ES-2 GW-treated group GAPDH IB.jpg]

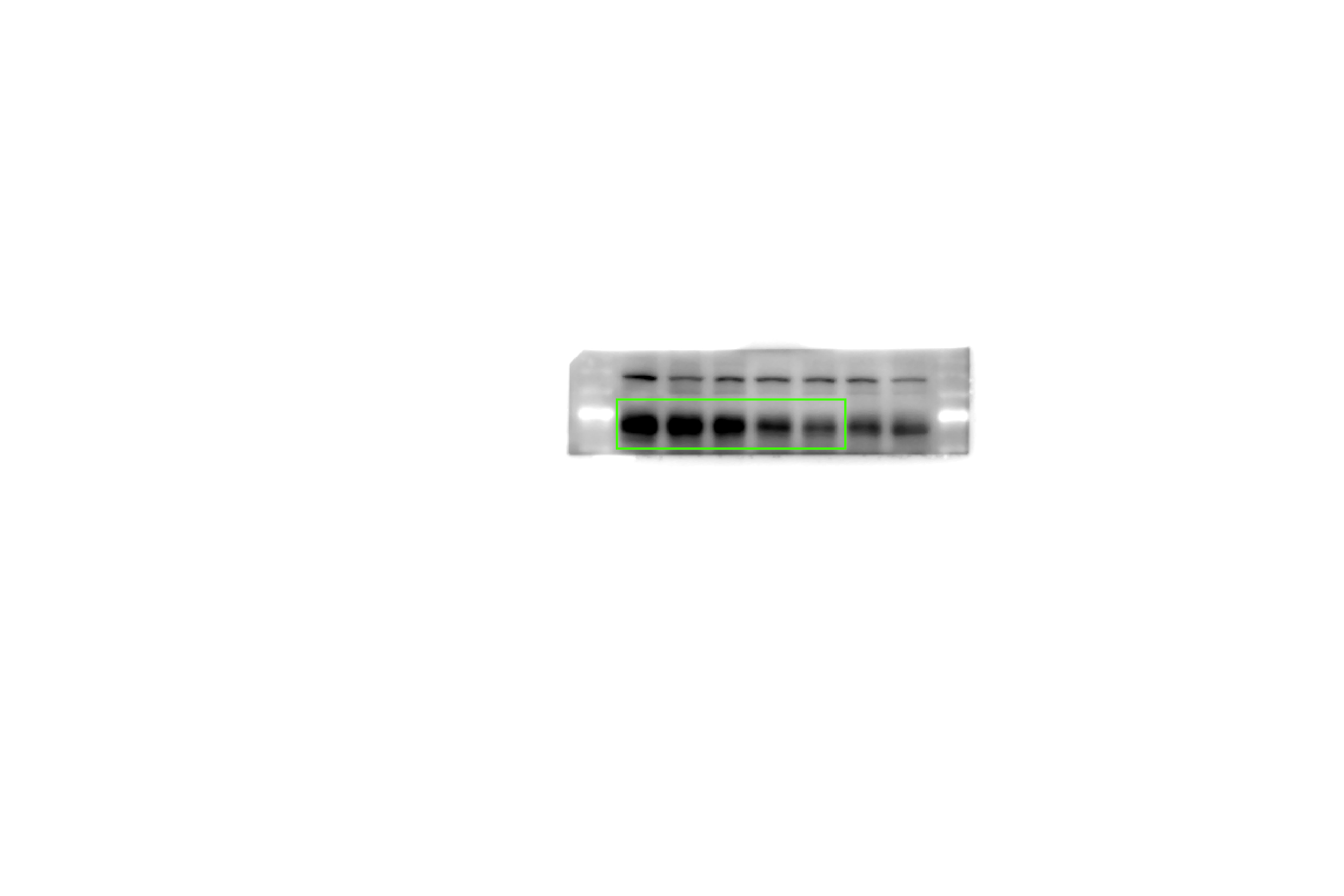

Supplement: Supplementary file 11 — Source data Fig. 4 [file 44321_2026_414_MOESM11_ESM.zip › Fig. 4/Fig. 4E/ES-2 Vehicle-treated group BMAL2 IB.jpg]

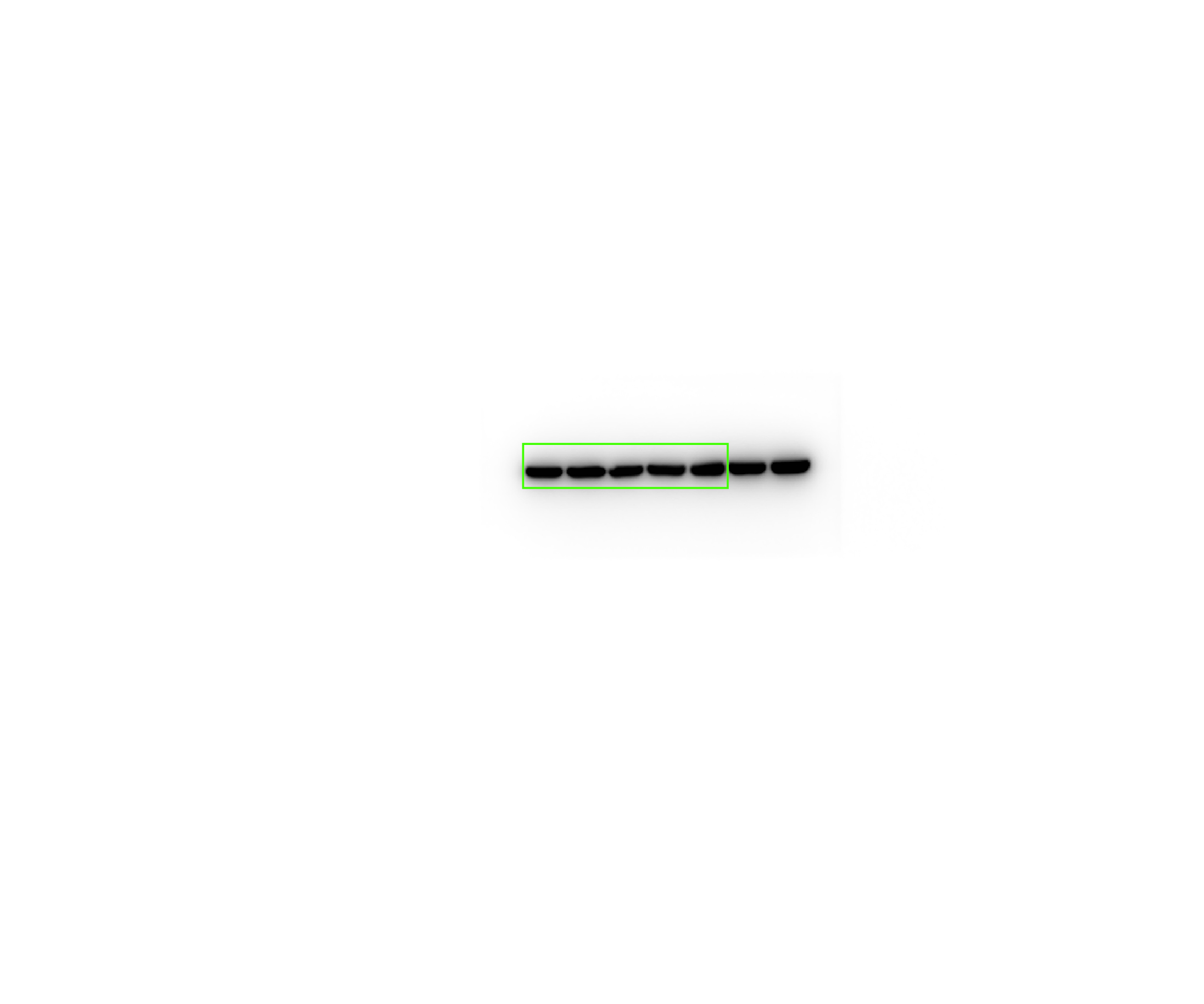

Supplement: Supplementary file 11 — Source data Fig. 4 [file 44321_2026_414_MOESM11_ESM.zip › Fig. 4/Fig. 4E/ES-2 Vehicle-treated group GAPDH IB.jpg]

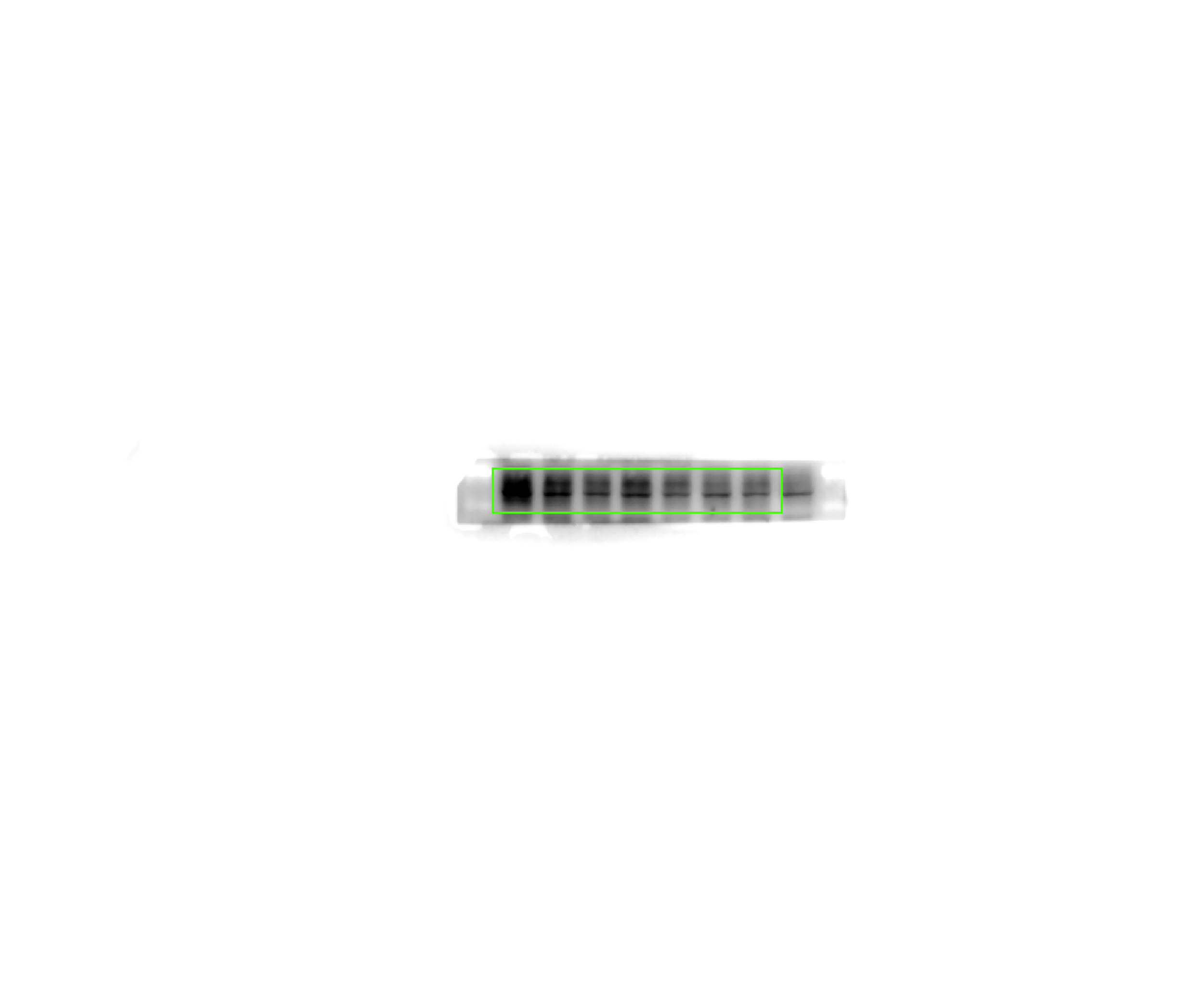

Supplement: Supplementary file 11 — Source data Fig. 4 [file 44321_2026_414_MOESM11_ESM.zip › Fig. 4/Fig. 4F/JHOC5 GW-treated group BMAL2 IB.jpg]

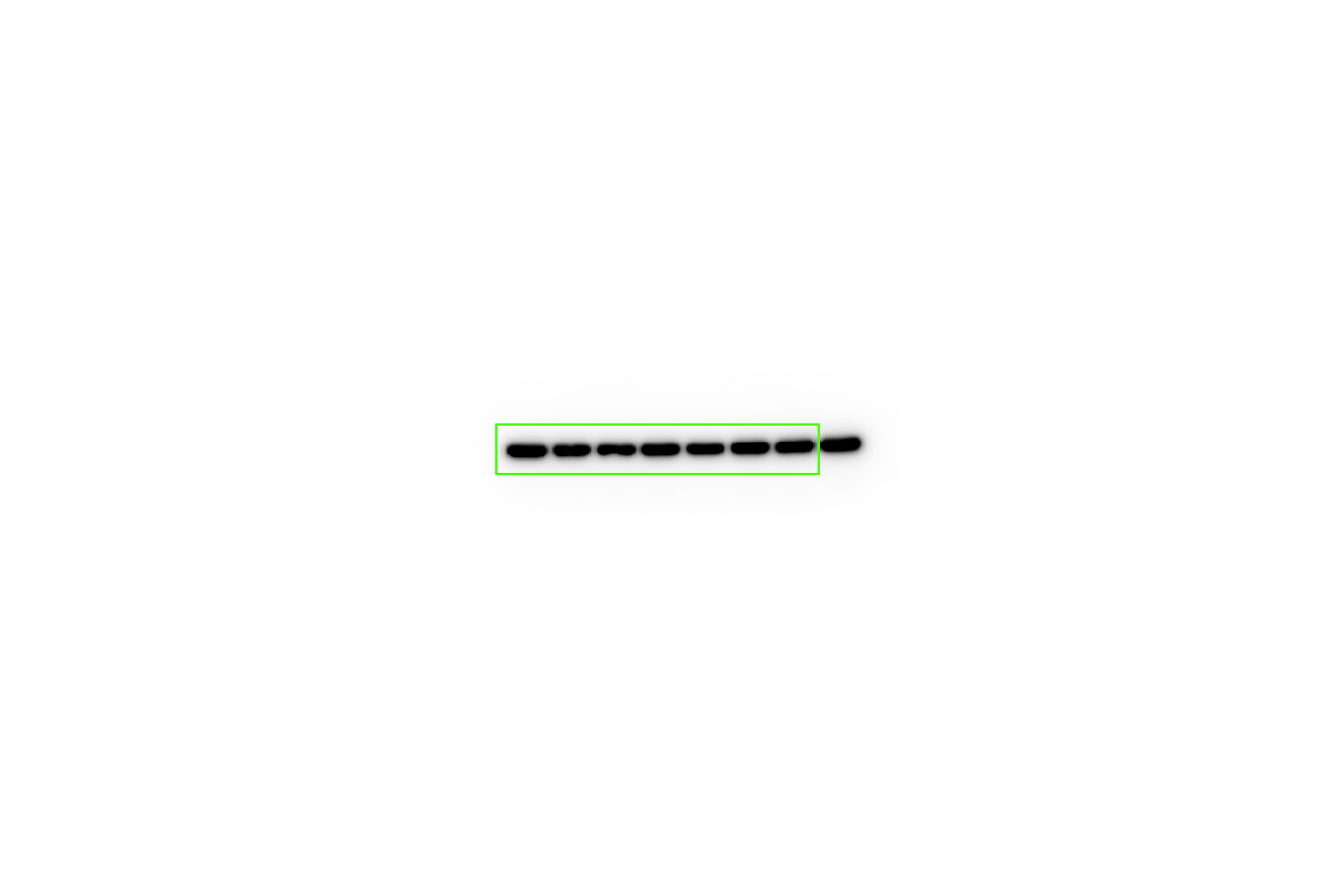

Supplement: Supplementary file 11 — Source data Fig. 4 [file 44321_2026_414_MOESM11_ESM.zip › Fig. 4/Fig. 4F/JHOC5 GW-treated group GAPDH IB.jpg]

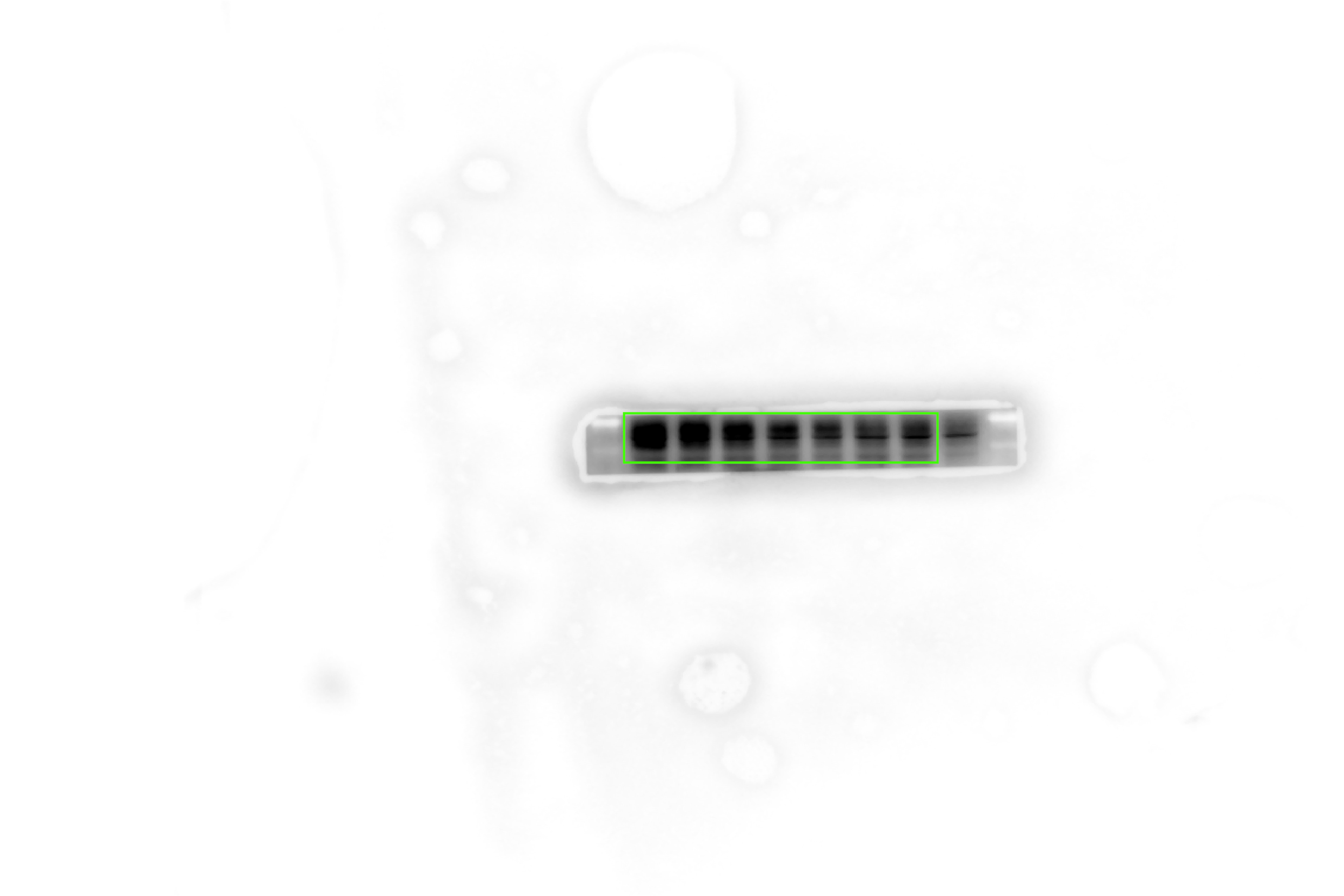

Supplement: Supplementary file 11 — Source data Fig. 4 [file 44321_2026_414_MOESM11_ESM.zip › Fig. 4/Fig. 4F/JHOC5 Vehicle-treated group BMAL2 IB.jpg]

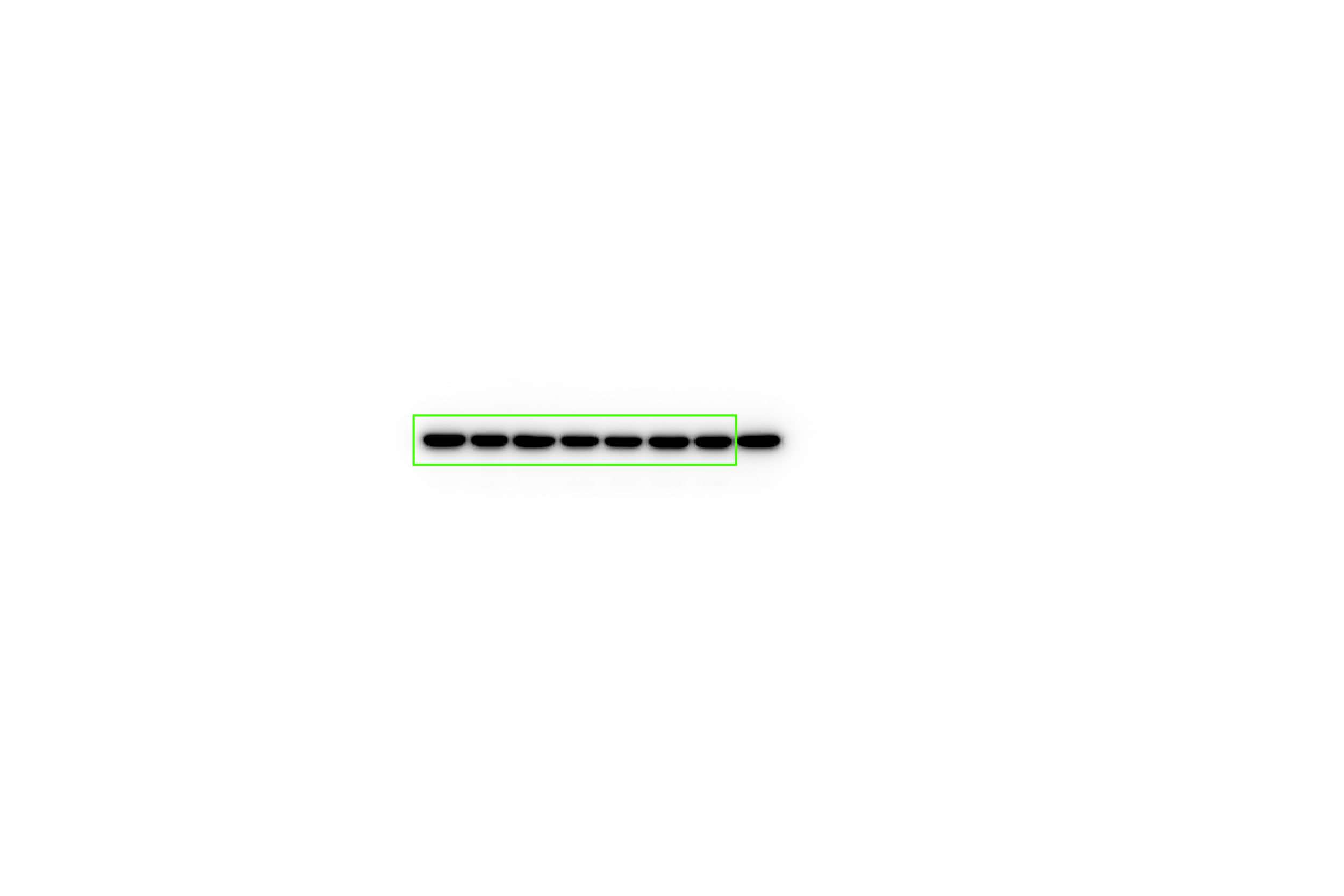

Supplement: Supplementary file 11 — Source data Fig. 4 [file 44321_2026_414_MOESM11_ESM.zip › Fig. 4/Fig. 4F/JHOC5 Vehicle-treated group GAPDH IB.jpg]

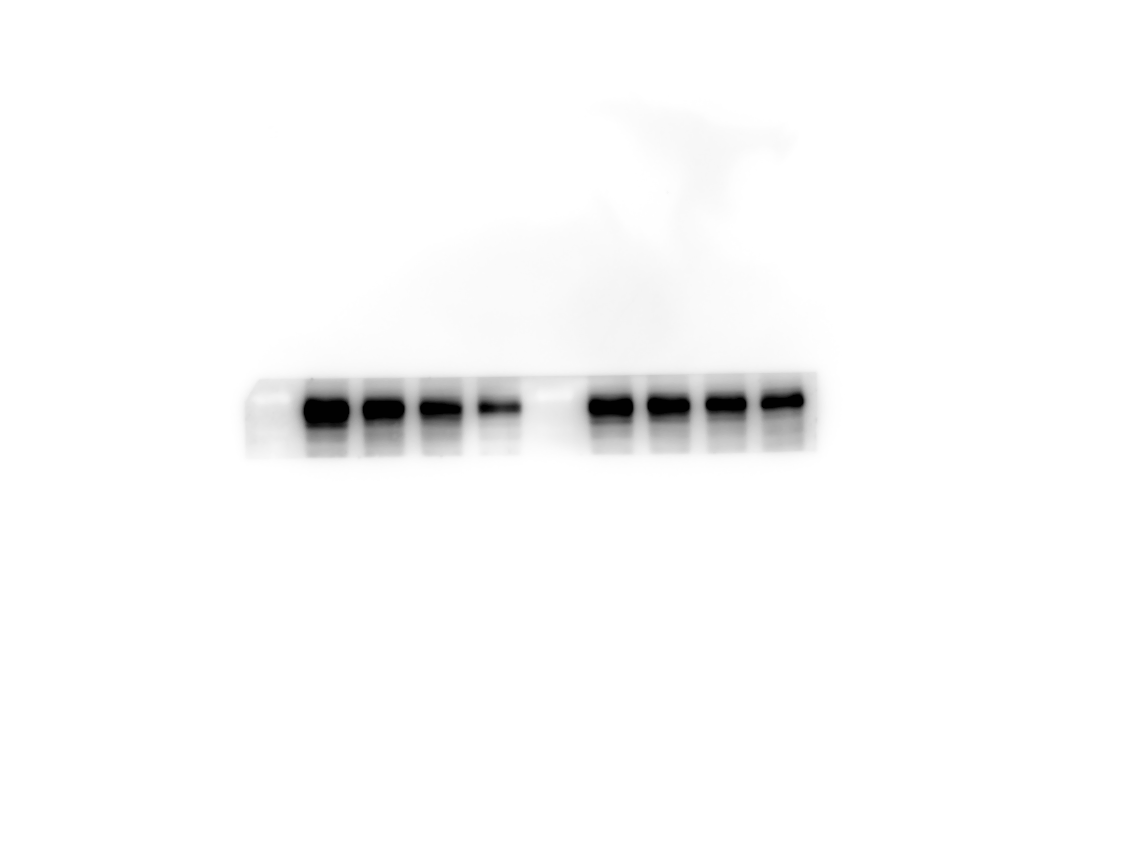

Supplement: Supplementary file 11 — Source data Fig. 4 [file 44321_2026_414_MOESM11_ESM.zip › Fig. 4/Fig. 4G/ES-2 BMAL2 IB.tif]

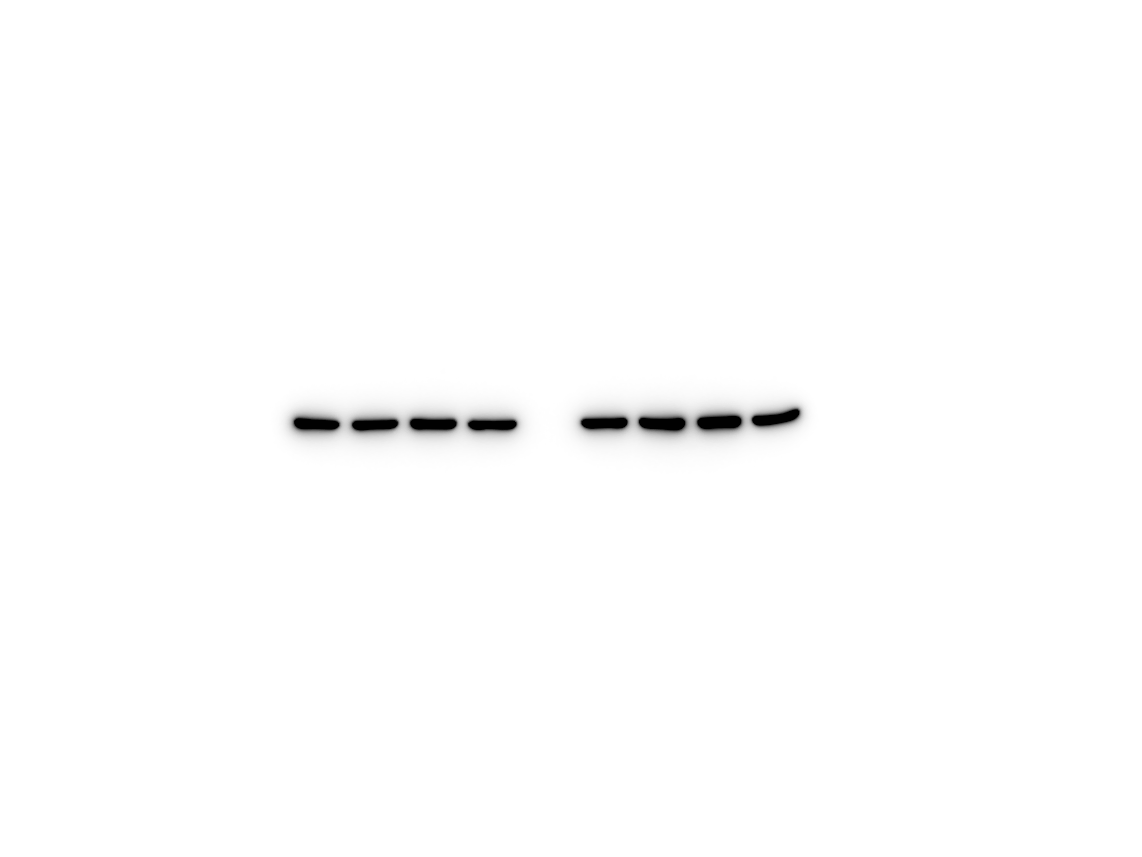

Supplement: Supplementary file 11 — Source data Fig. 4 [file 44321_2026_414_MOESM11_ESM.zip › Fig. 4/Fig. 4G/ES-2 GAPDH IB.tif]

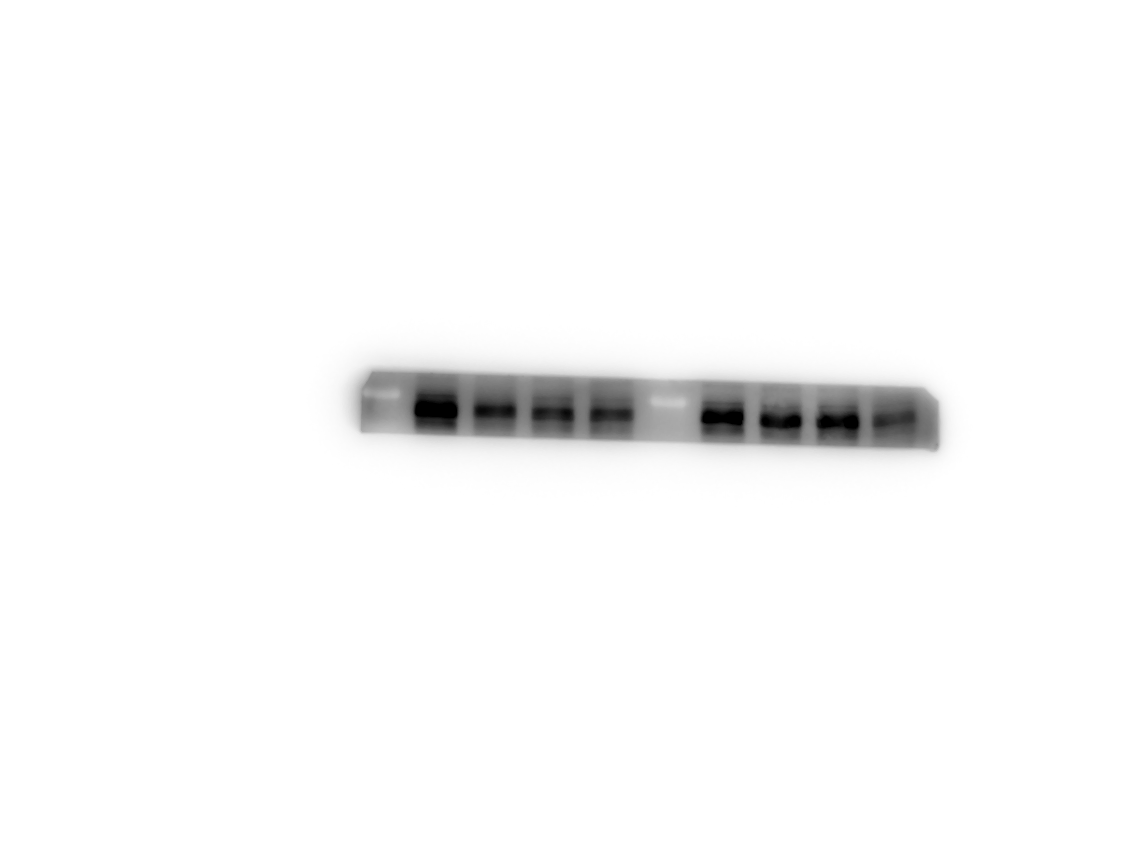

Supplement: Supplementary file 11 — Source data Fig. 4 [file 44321_2026_414_MOESM11_ESM.zip › Fig. 4/Fig. 4H/JHOC5 BMAL2 IB.tif]

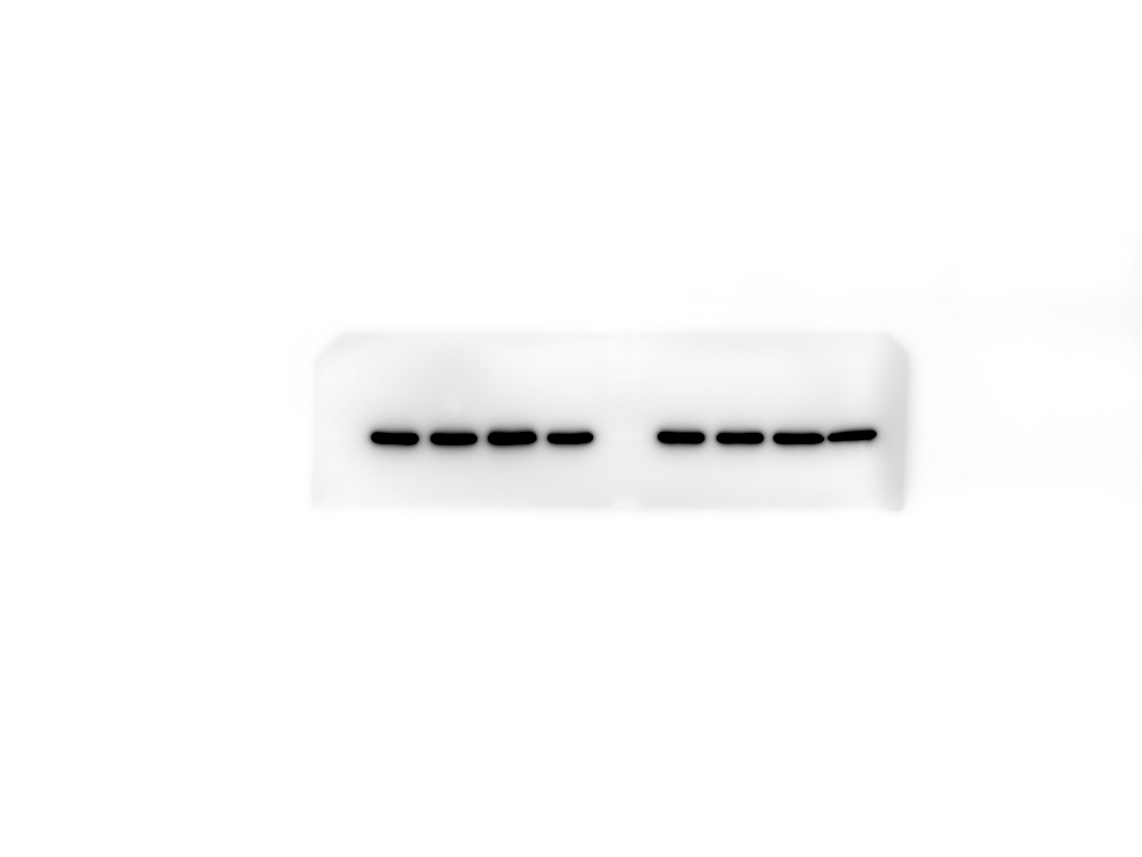

Supplement: Supplementary file 11 — Source data Fig. 4 [file 44321_2026_414_MOESM11_ESM.zip › Fig. 4/Fig. 4H/JHOC5 GAPDH IB.tif]

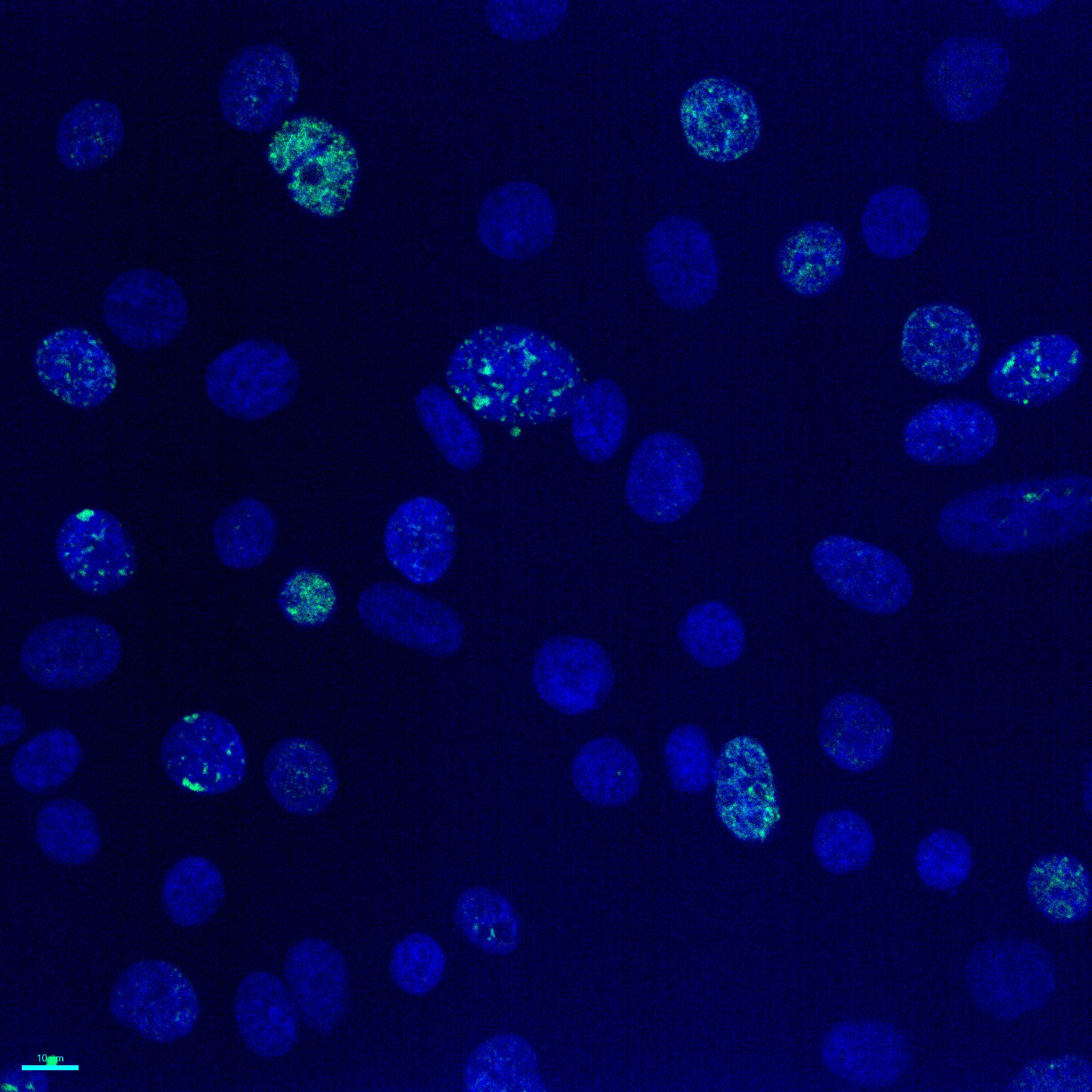

Supplement: Supplementary file 12 — Source data Fig. 5 [file 44321_2026_414_MOESM12_ESM.zip › Fig. 5/Fig. 5A/ES-2 10uM GW.jpg]

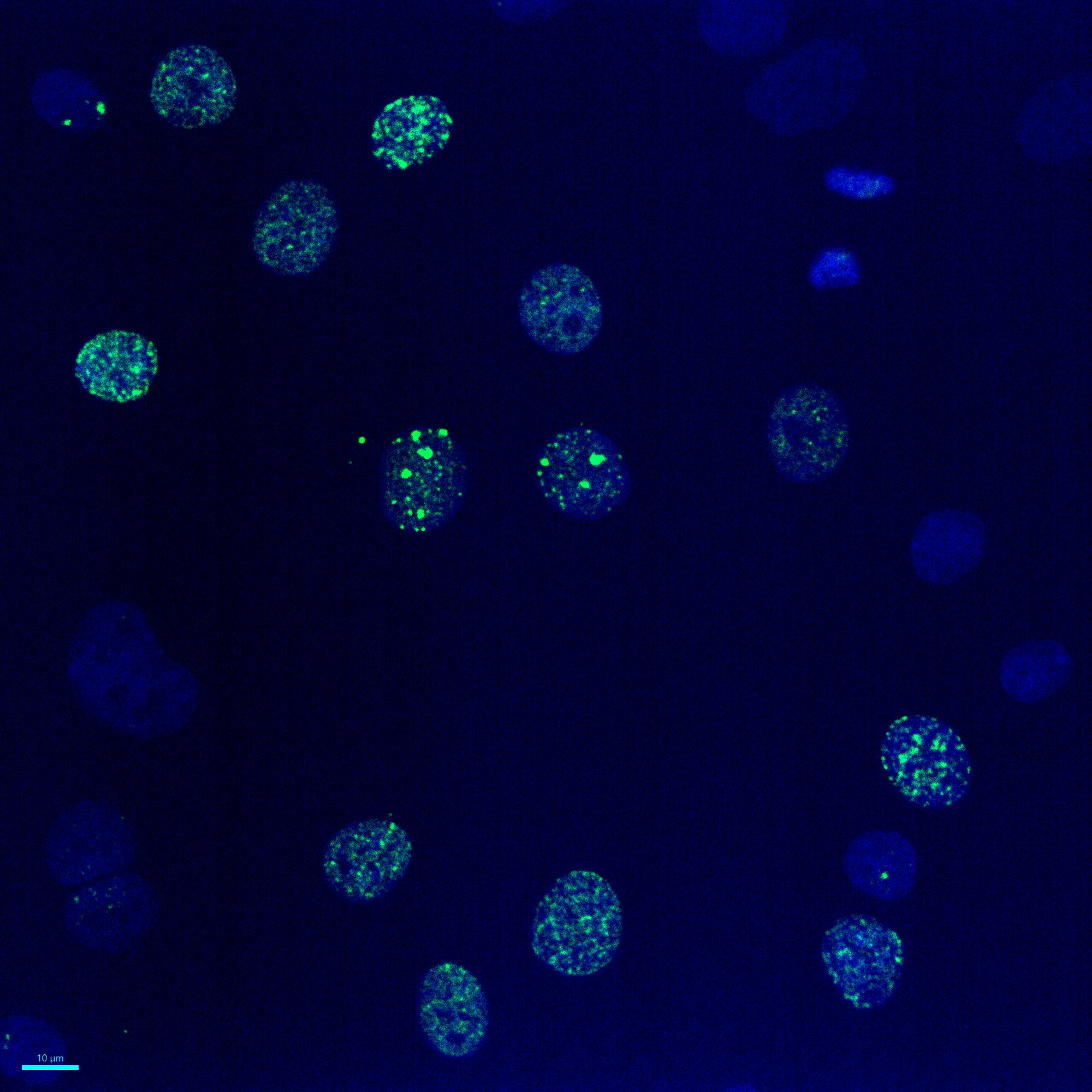

Supplement: Supplementary file 12 — Source data Fig. 5 [file 44321_2026_414_MOESM12_ESM.zip › Fig. 5/Fig. 5A/ES-2 20uM GW.jpg]

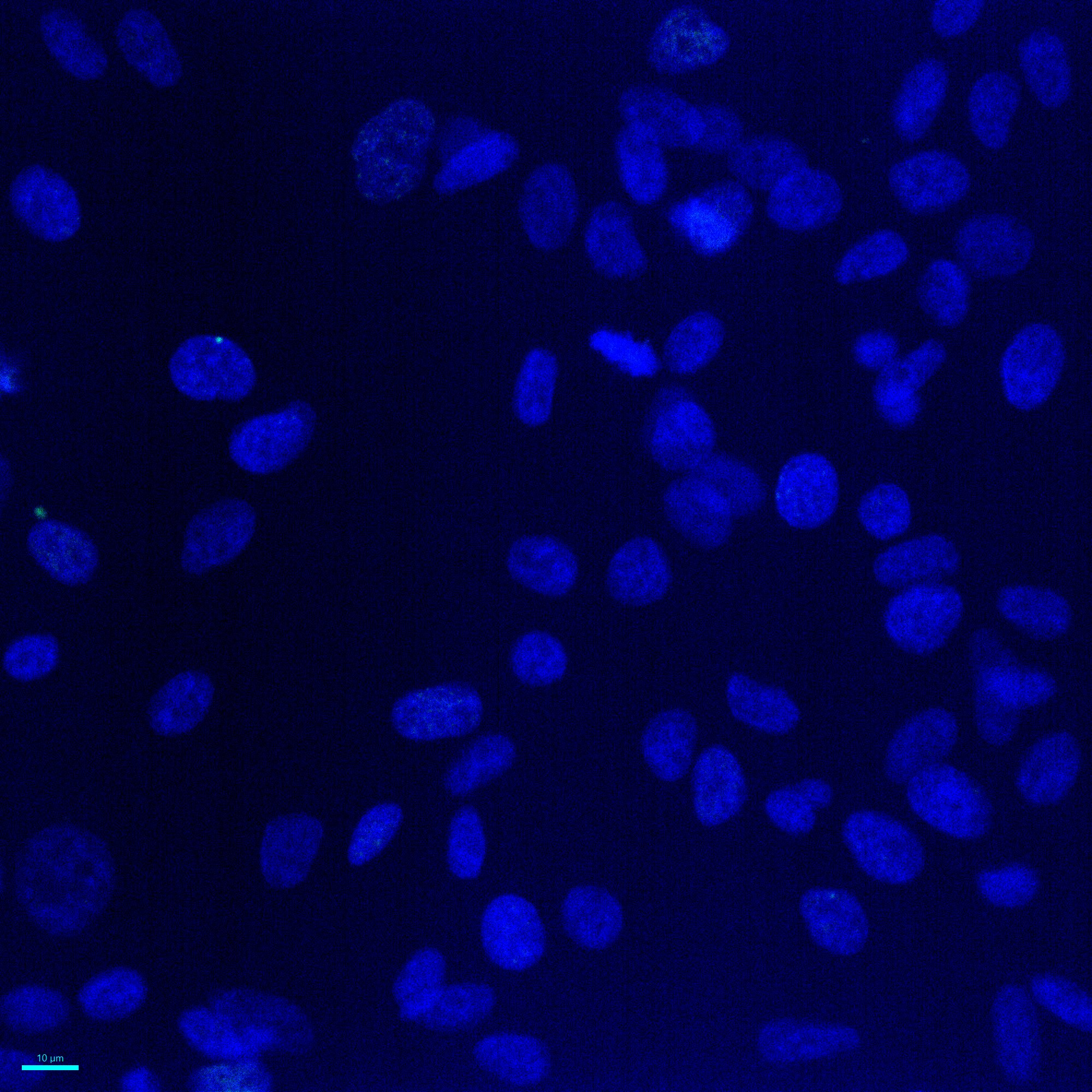

Supplement: Supplementary file 12 — Source data Fig. 5 [file 44321_2026_414_MOESM12_ESM.zip › Fig. 5/Fig. 5A/ES-2 Vehicle.jpg]

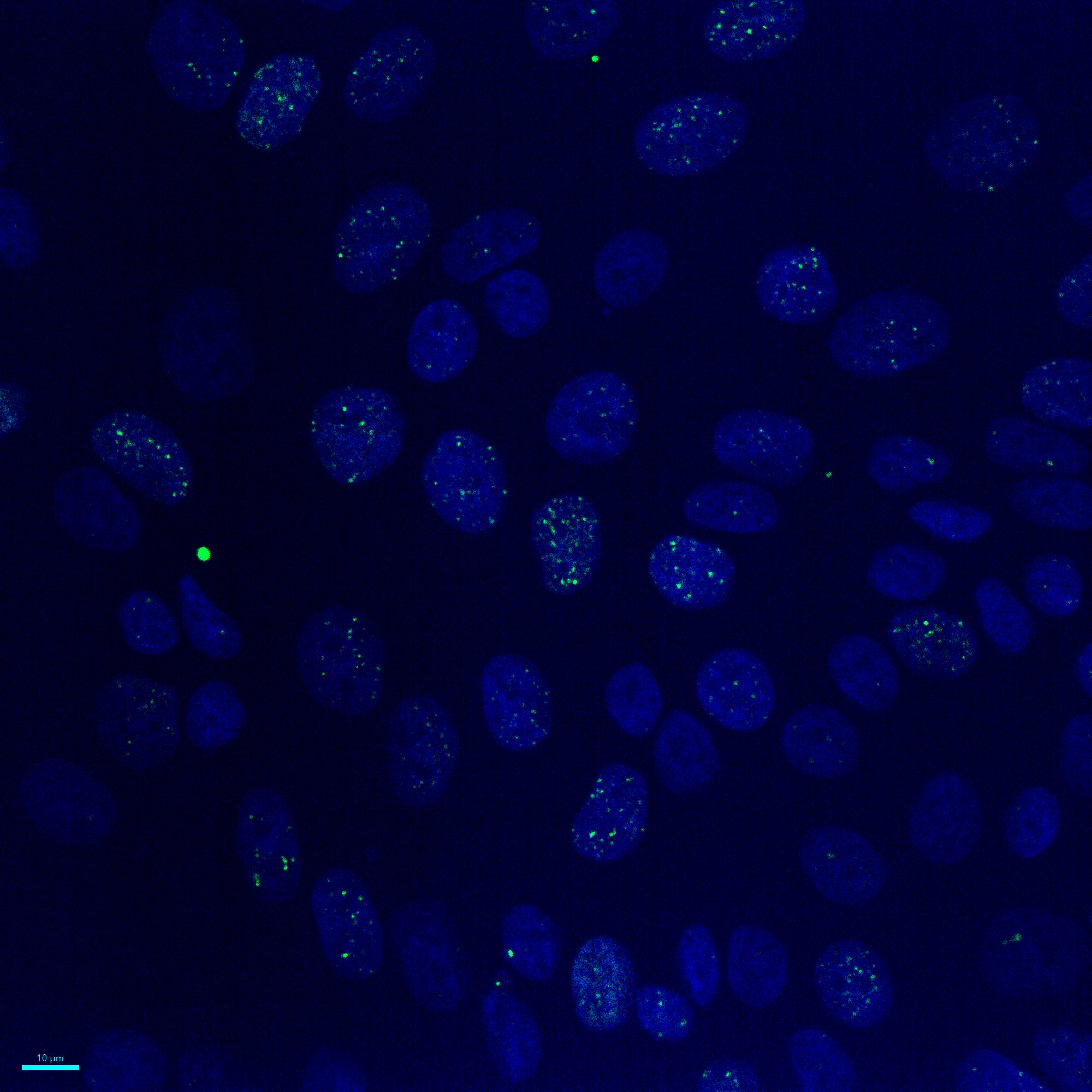

Supplement: Supplementary file 12 — Source data Fig. 5 [file 44321_2026_414_MOESM12_ESM.zip › Fig. 5/Fig. 5A/JHOC5 10uM GW.jpg]

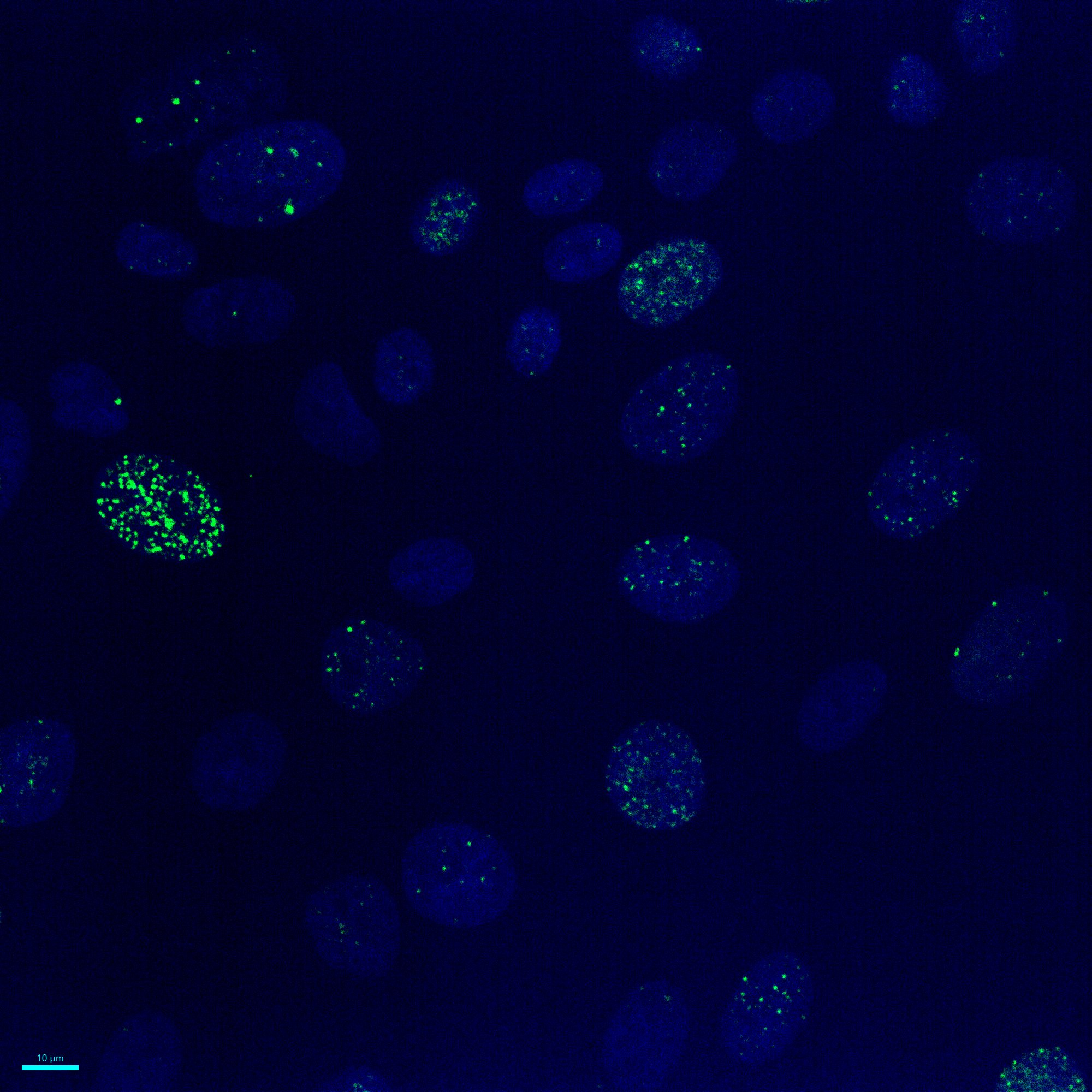

Supplement: Supplementary file 12 — Source data Fig. 5 [file 44321_2026_414_MOESM12_ESM.zip › Fig. 5/Fig. 5A/JHOC5 20uM GW.jpg]

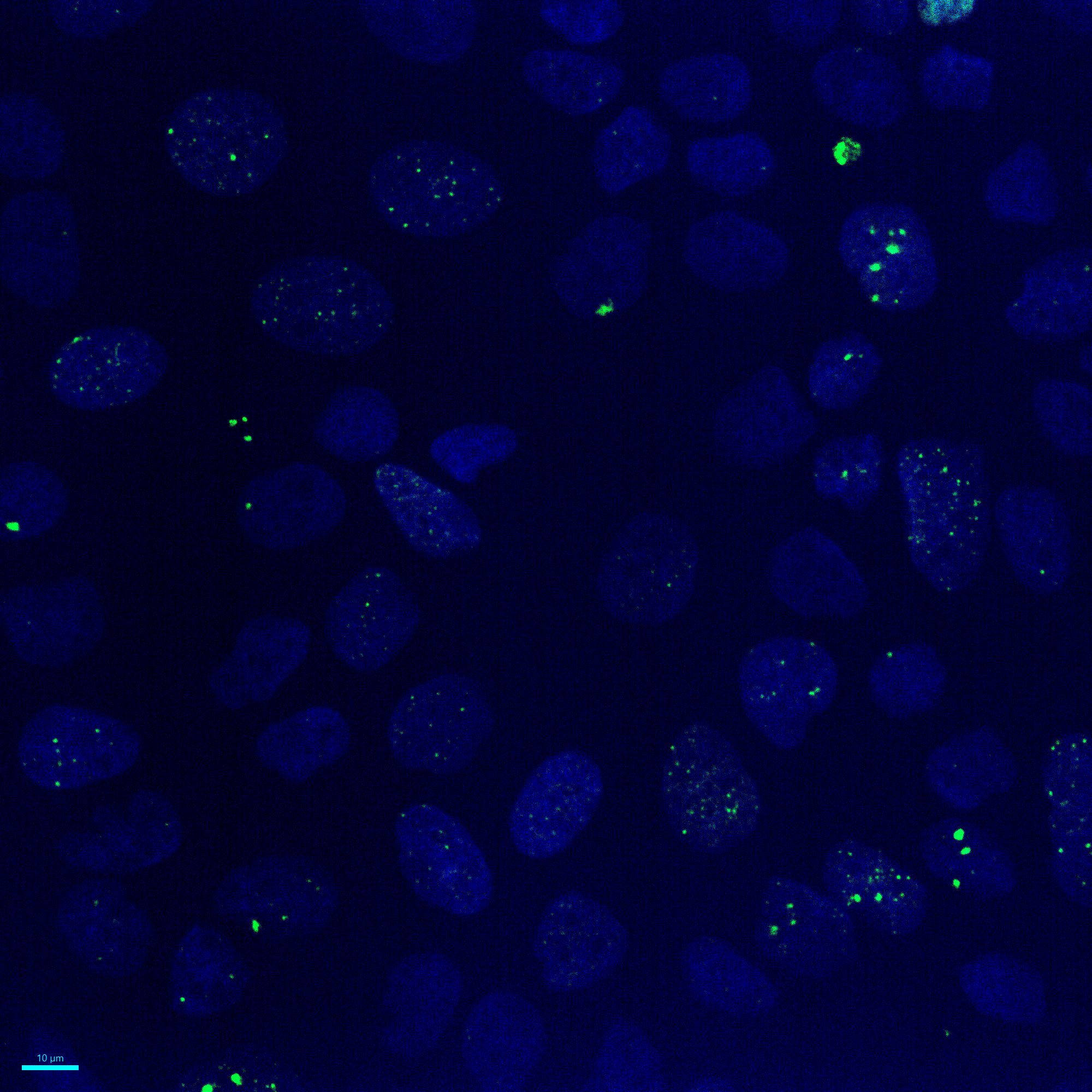

Supplement: Supplementary file 12 — Source data Fig. 5 [file 44321_2026_414_MOESM12_ESM.zip › Fig. 5/Fig. 5A/JHOC5 Vehicle.jpg]

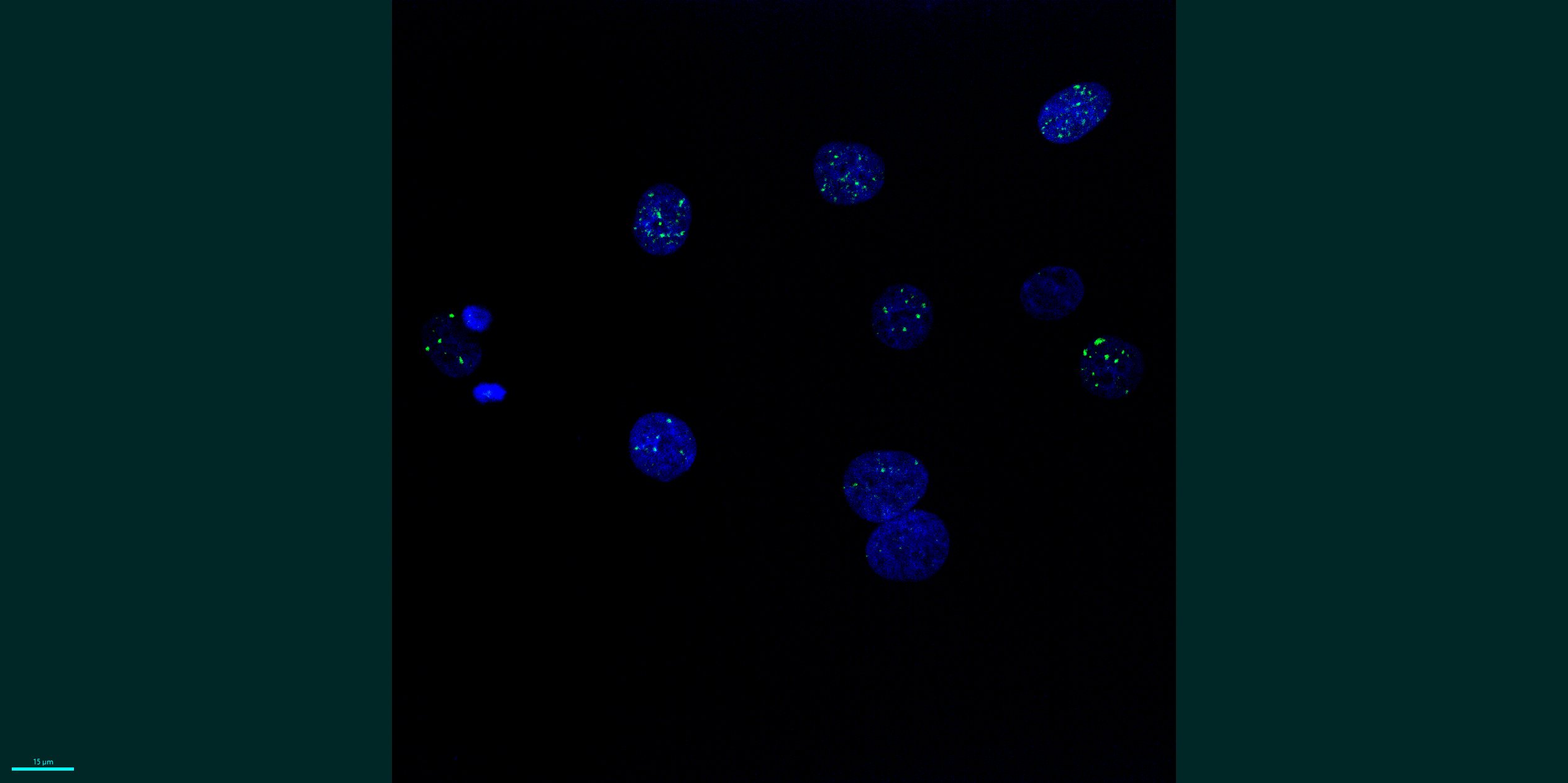

Supplement: Supplementary file 12 — Source data Fig. 5 [file 44321_2026_414_MOESM12_ESM.zip › Fig. 5/Fig. 5A/JHOC9 10uM GW.jpg]

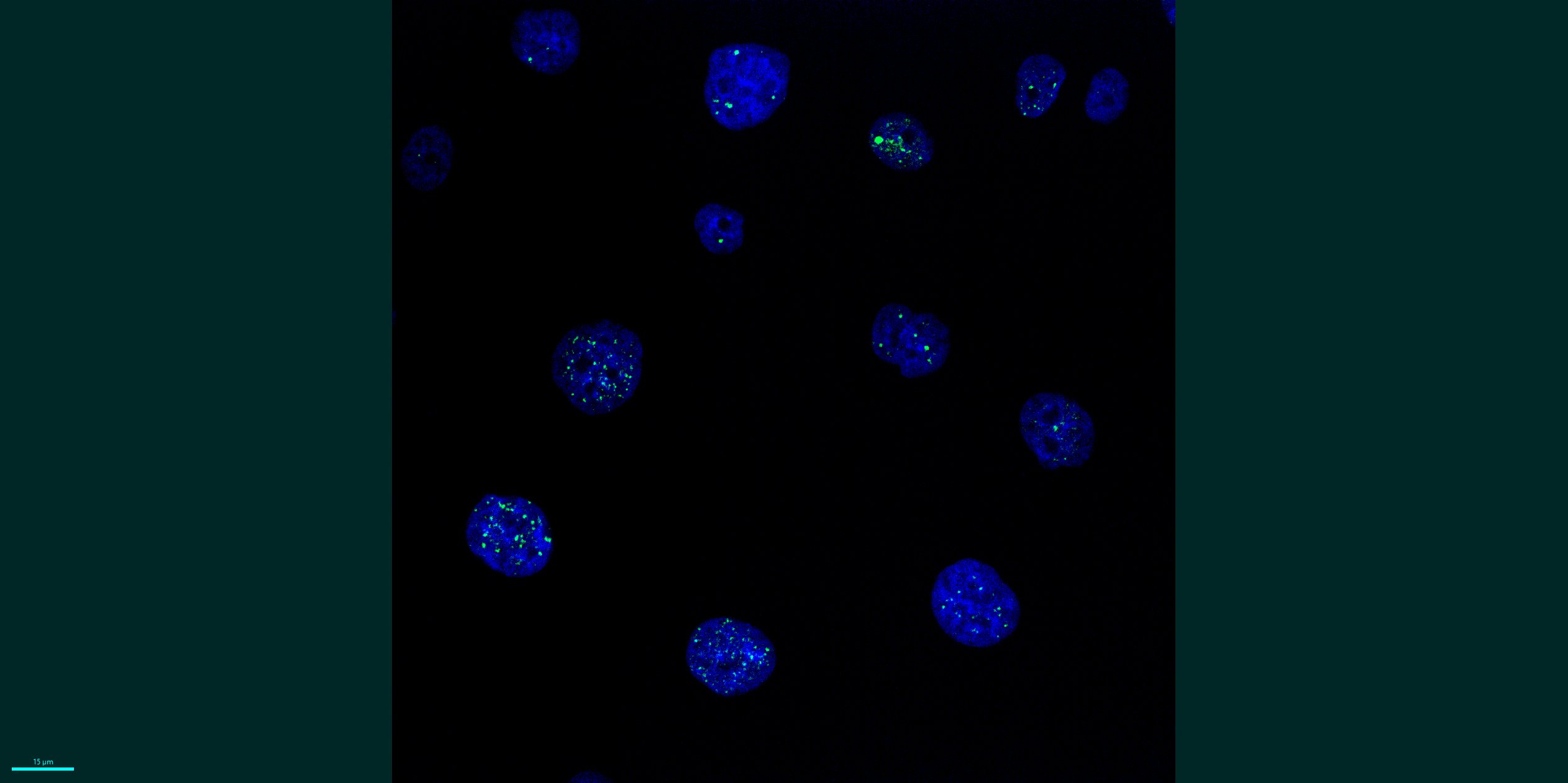

Supplement: Supplementary file 12 — Source data Fig. 5 [file 44321_2026_414_MOESM12_ESM.zip › Fig. 5/Fig. 5A/JHOC9 20uM GW.jpg]

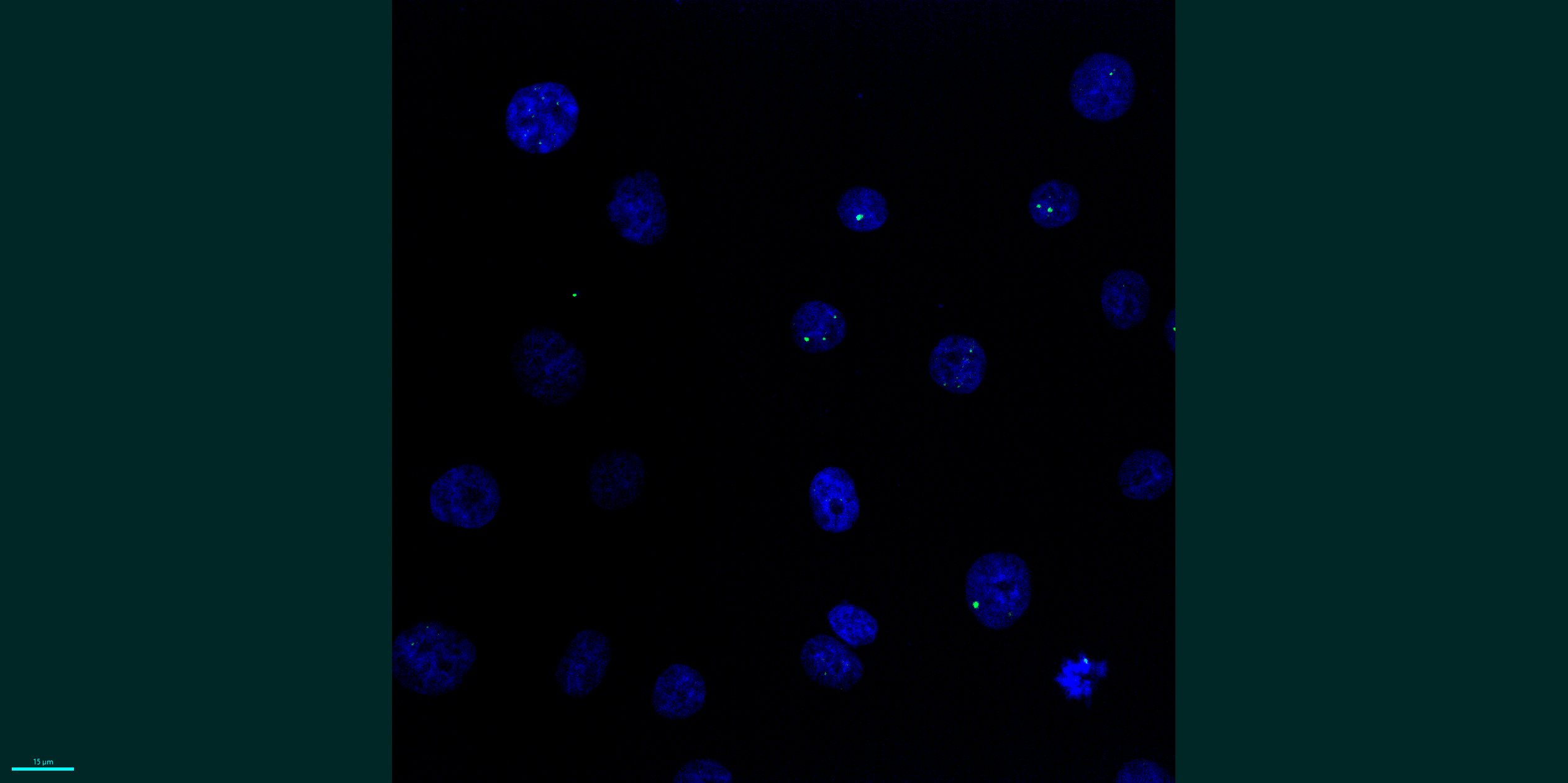

Supplement: Supplementary file 12 — Source data Fig. 5 [file 44321_2026_414_MOESM12_ESM.zip › Fig. 5/Fig. 5A/JHOC9 Vehicle.jpg]

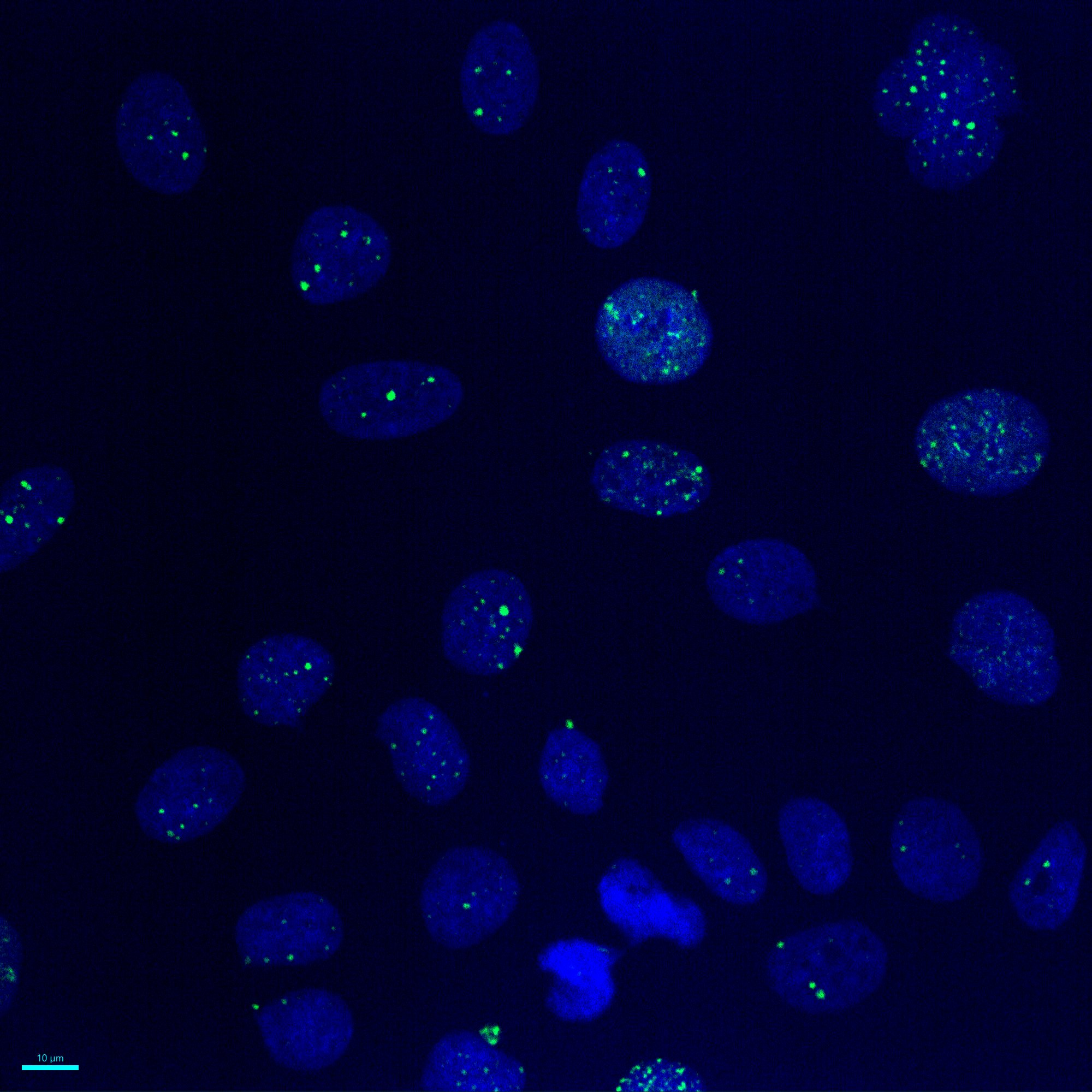

Supplement: Supplementary file 12 — Source data Fig. 5 [file 44321_2026_414_MOESM12_ESM.zip › Fig. 5/Fig. 5A/OVCA429 10uM GW.jpg]

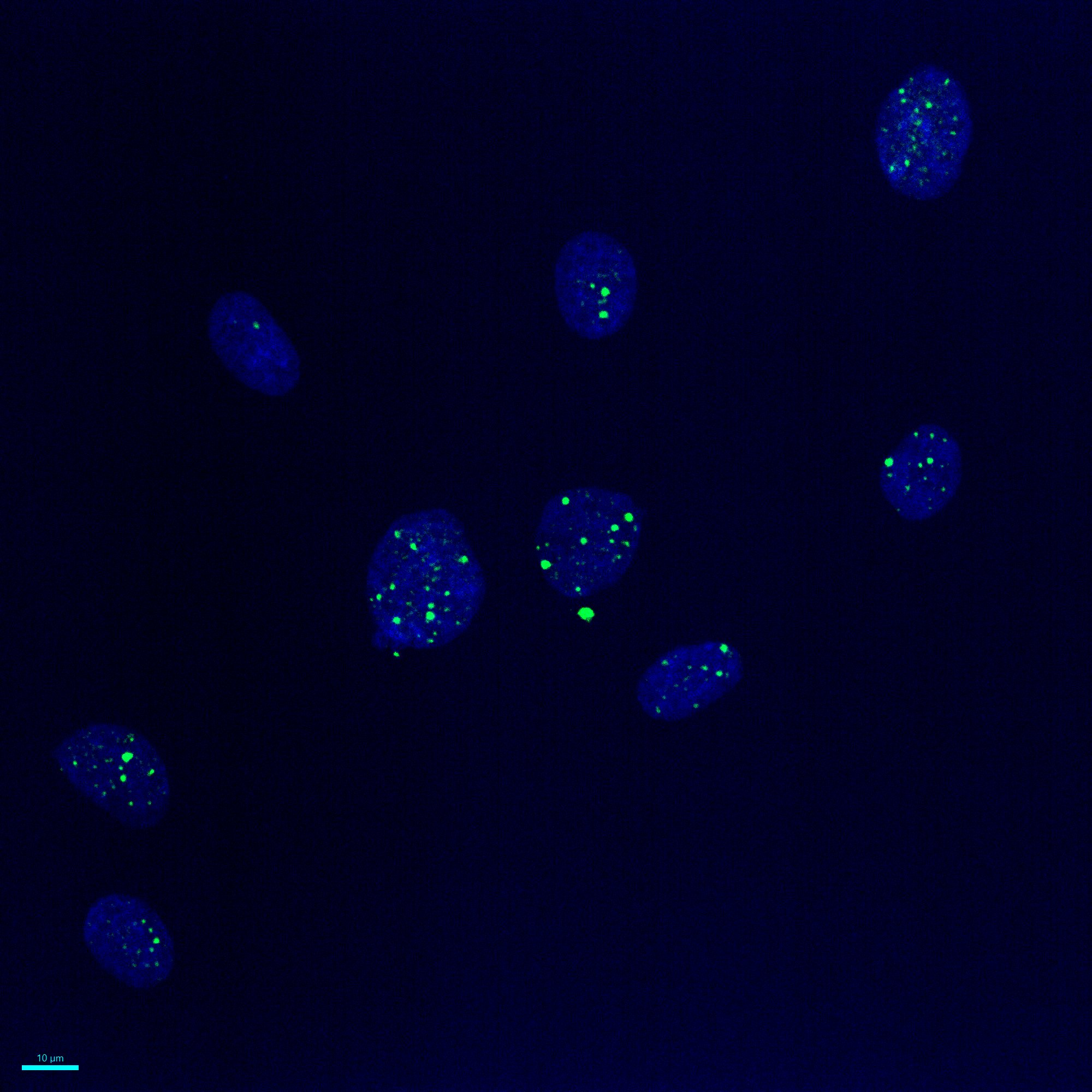

Supplement: Supplementary file 12 — Source data Fig. 5 [file 44321_2026_414_MOESM12_ESM.zip › Fig. 5/Fig. 5A/OVCA429 20uM GW.jpg]

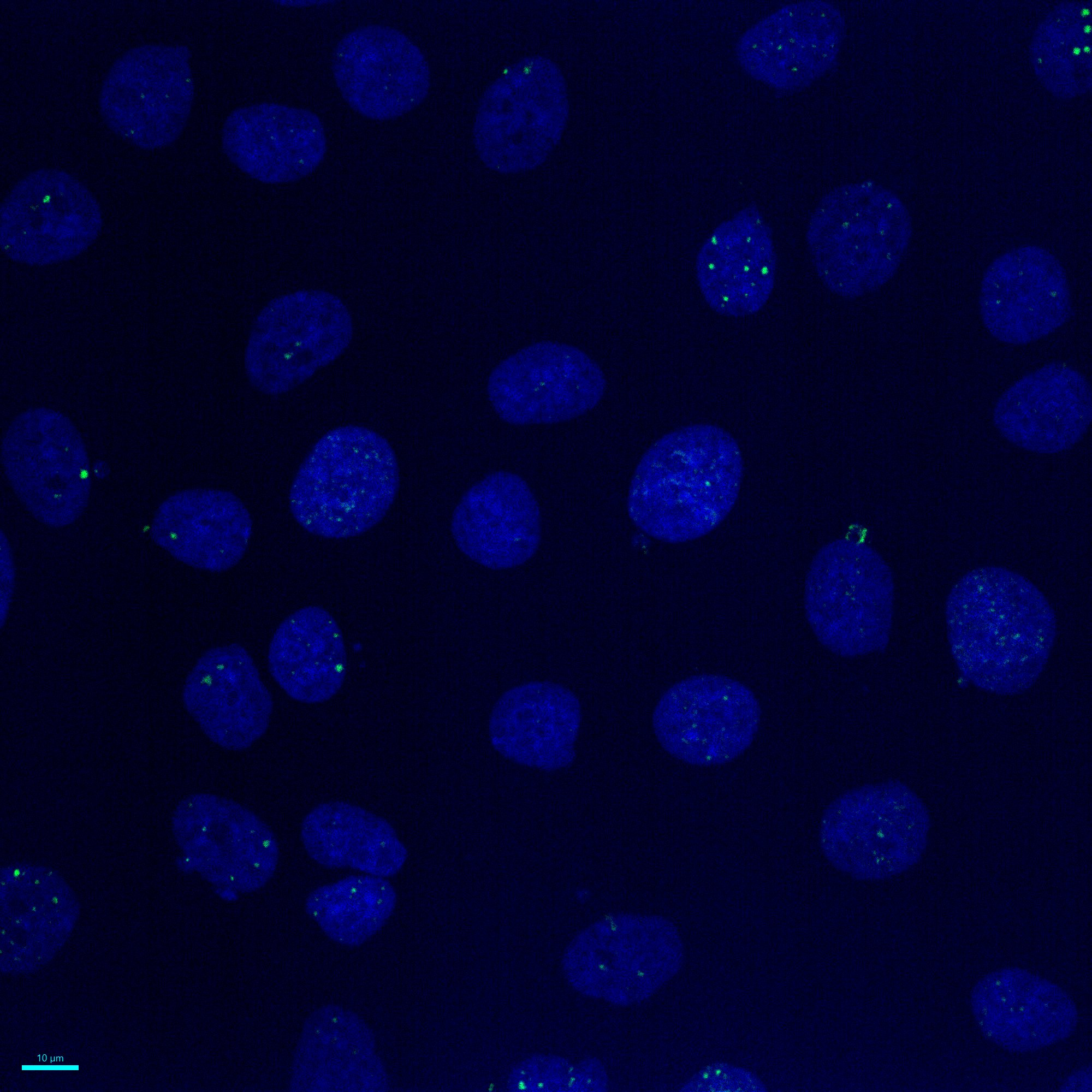

Supplement: Supplementary file 12 — Source data Fig. 5 [file 44321_2026_414_MOESM12_ESM.zip › Fig. 5/Fig. 5A/OVCA429 Vehicle.jpg]

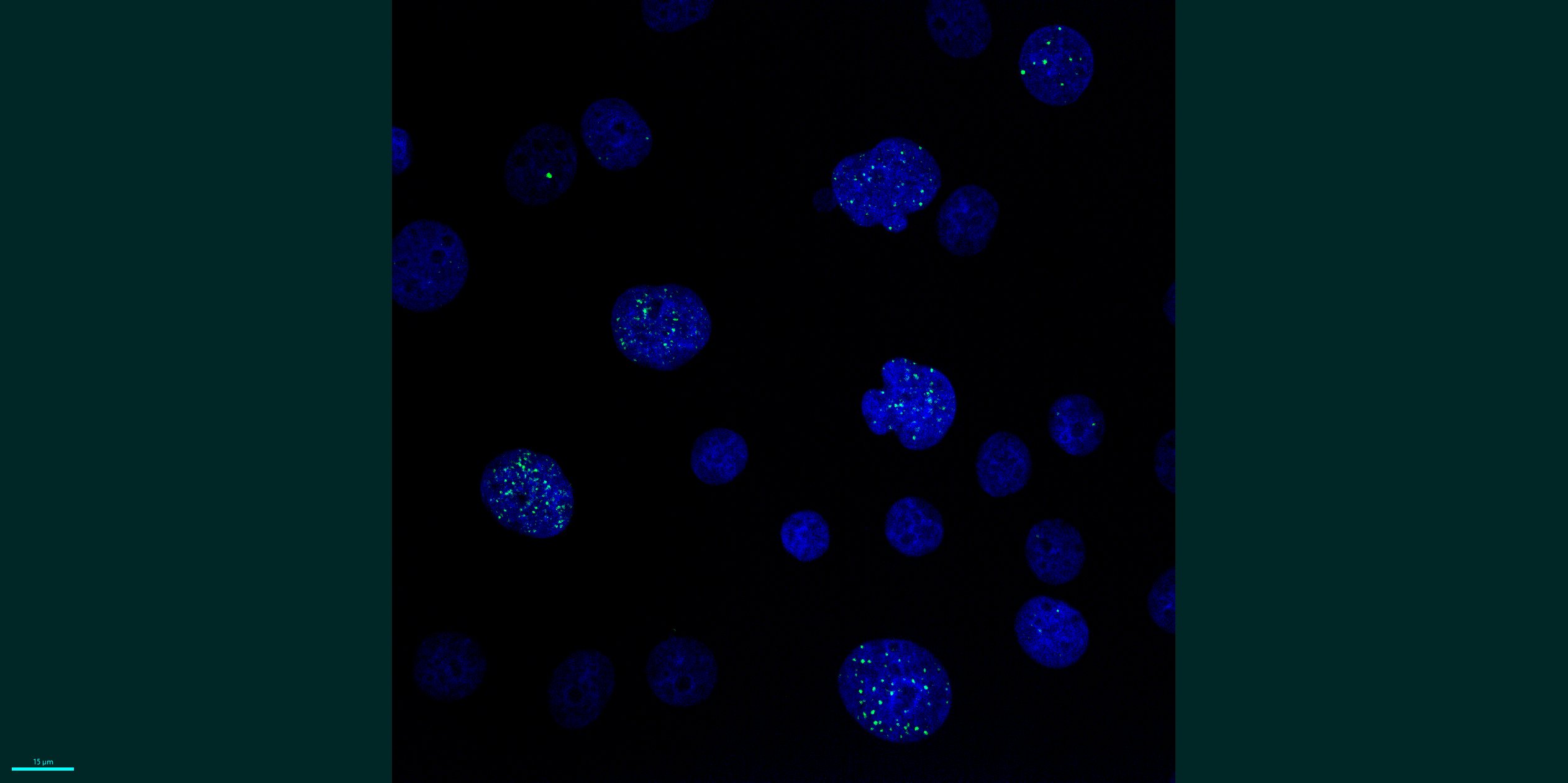

Supplement: Supplementary file 12 — Source data Fig. 5 [file 44321_2026_414_MOESM12_ESM.zip › Fig. 5/Fig. 5A/OVISE 10uM GW.jpg]

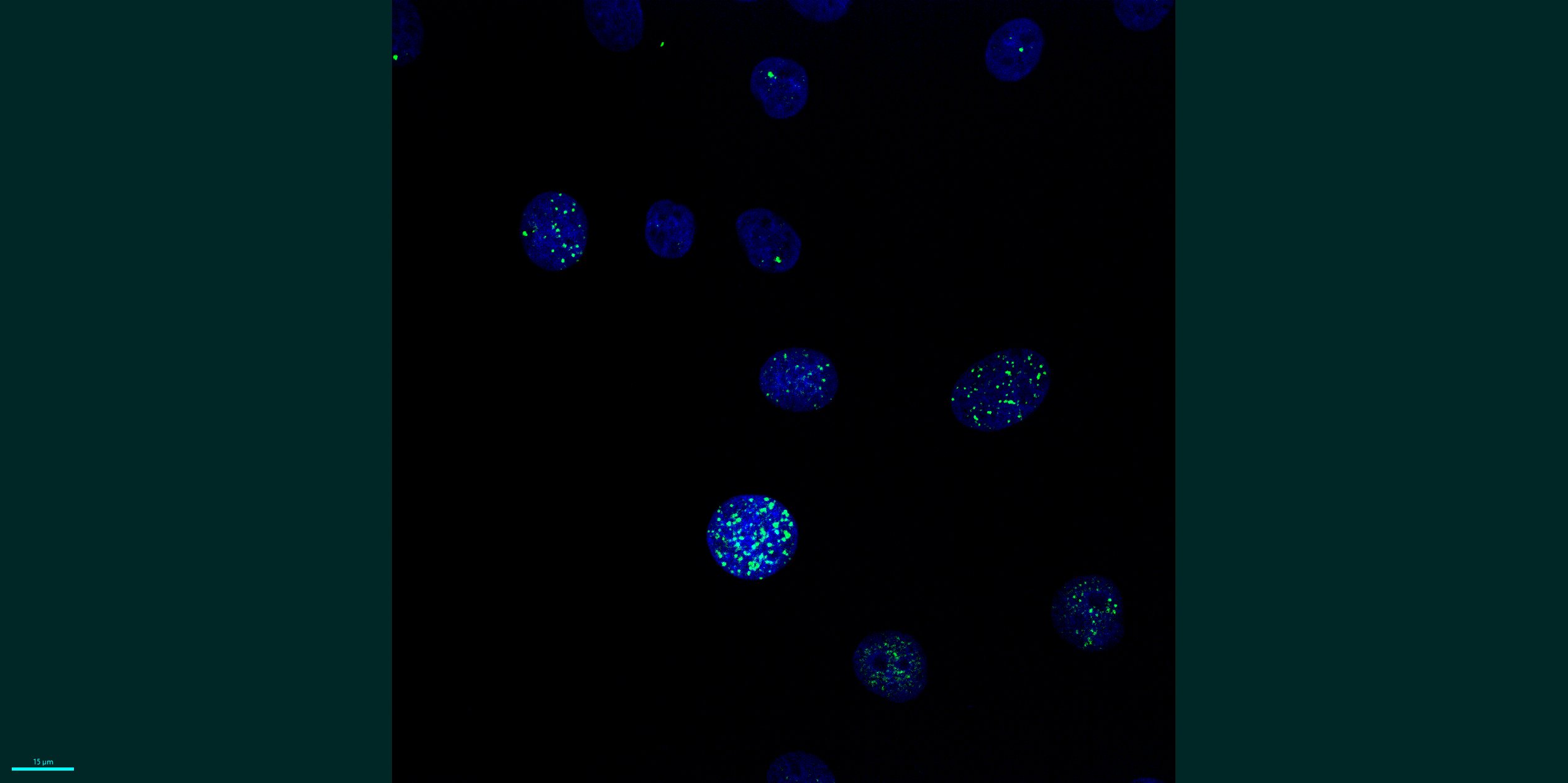

Supplement: Supplementary file 12 — Source data Fig. 5 [file 44321_2026_414_MOESM12_ESM.zip › Fig. 5/Fig. 5A/OVISE 20uM GW.jpg]

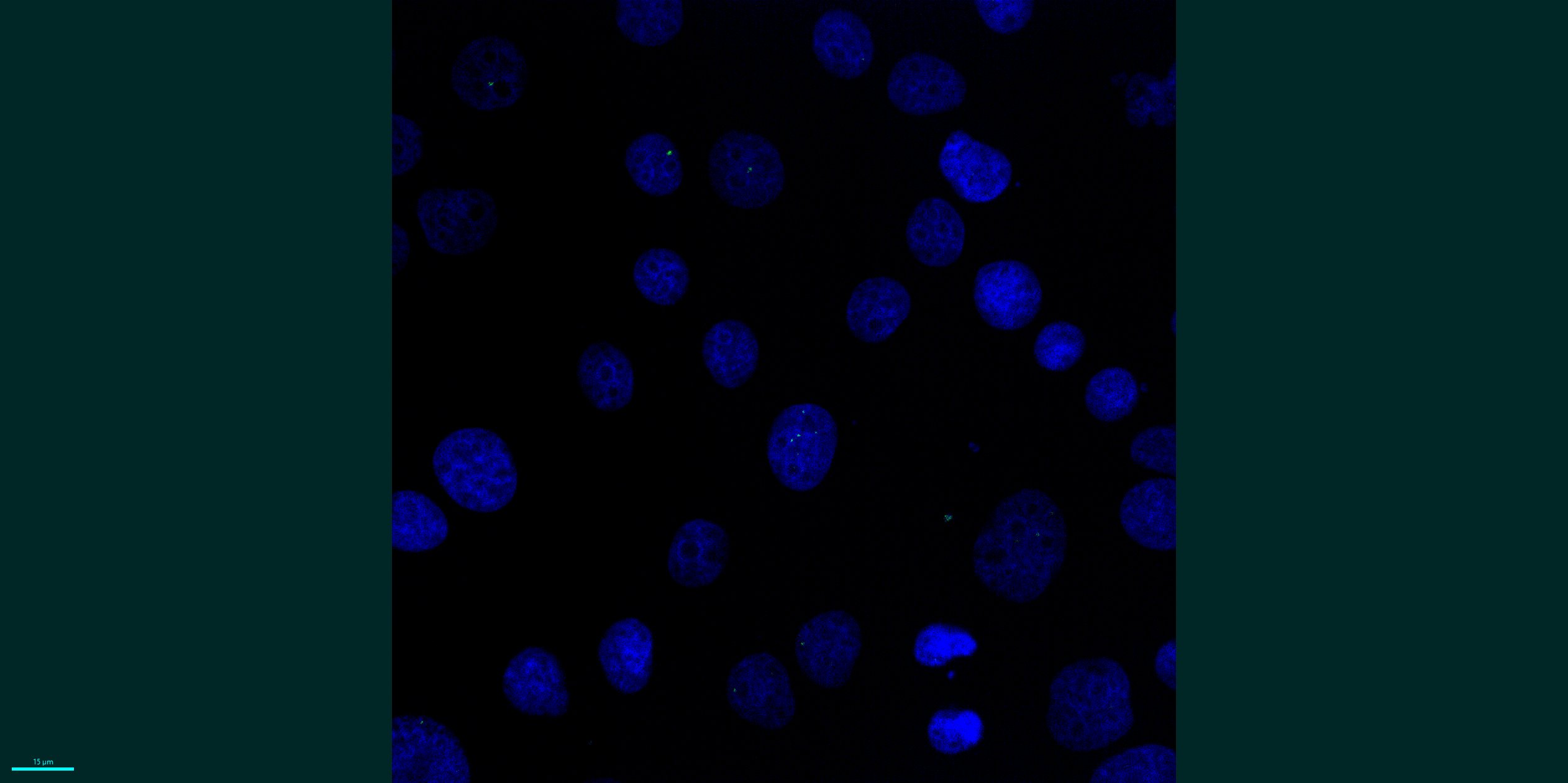

Supplement: Supplementary file 12 — Source data Fig. 5 [file 44321_2026_414_MOESM12_ESM.zip › Fig. 5/Fig. 5A/OVISE Vehicle.jpg]

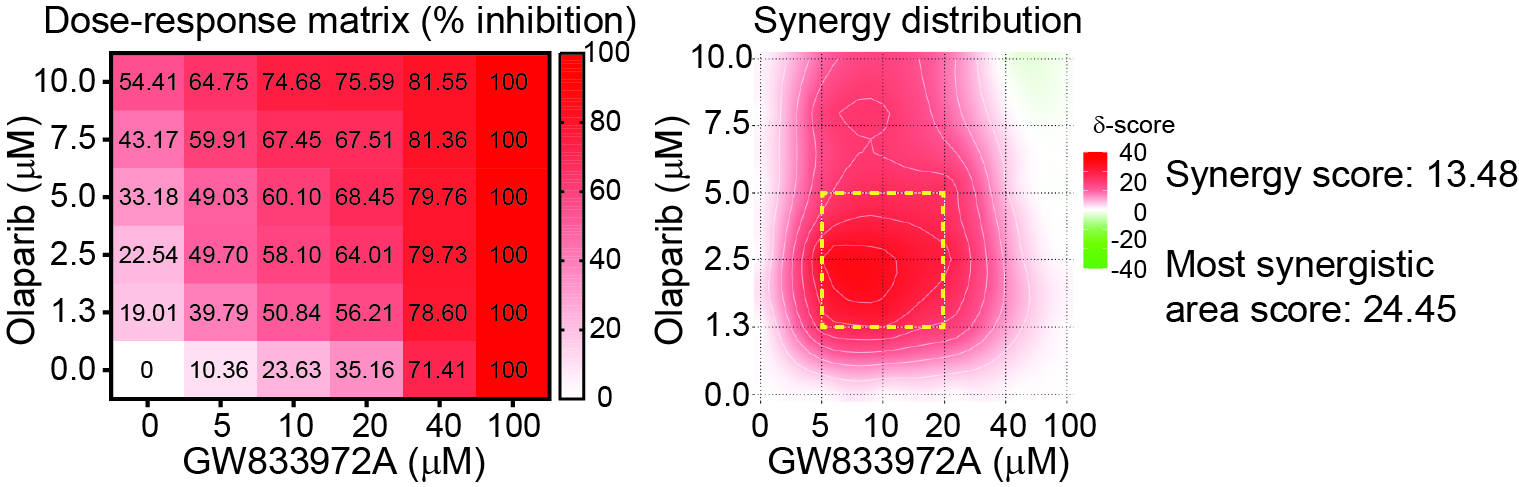

Supplement: Supplementary file 12 — Source data Fig. 5 [file 44321_2026_414_MOESM12_ESM.zip › Fig. 5/Fig. 5G/ES-2 Bliss.jpg]

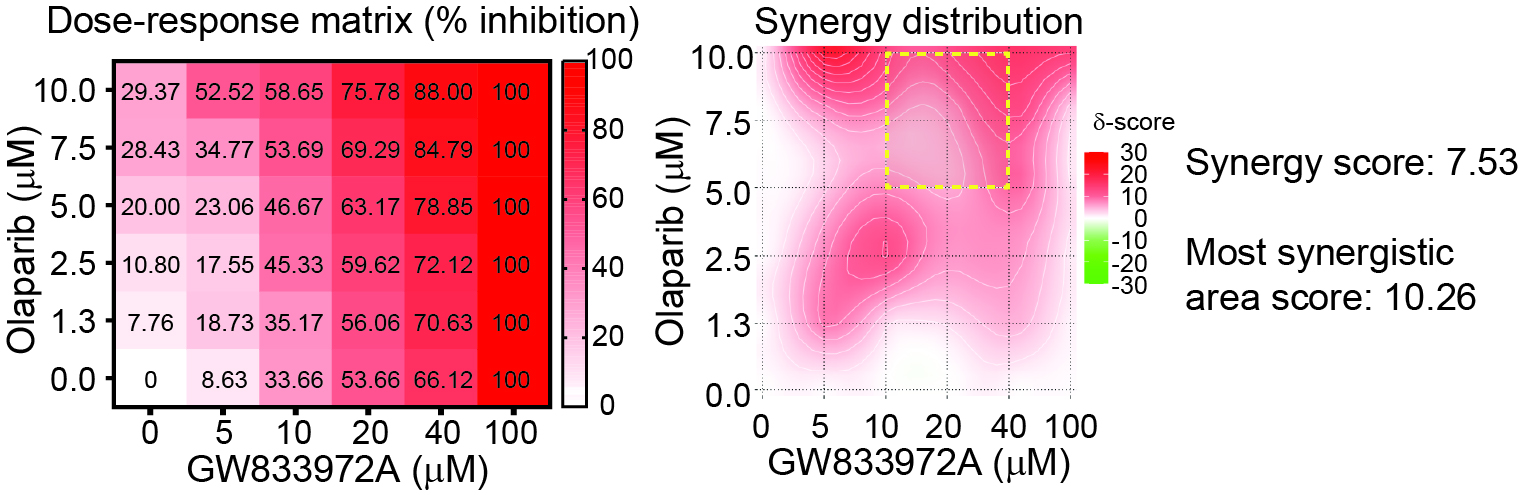

Supplement: Supplementary file 12 — Source data Fig. 5 [file 44321_2026_414_MOESM12_ESM.zip › Fig. 5/Fig. 5H/JHOC5 Bliss.jpg]

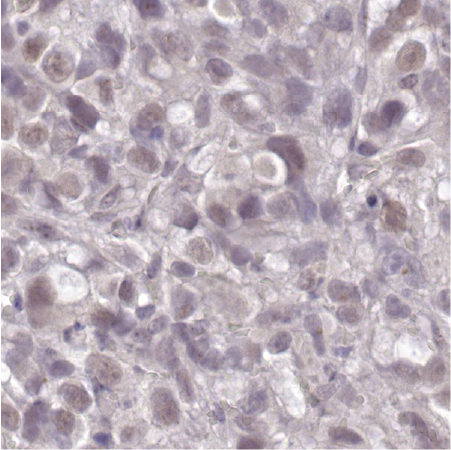

Supplement: Supplementary file 13 — Source data Fig. 6 [file 44321_2026_414_MOESM13_ESM.zip › EMM-2025-22875-V3-Figure_6_Source_Data-sd_V3/Fig. 6/Fig. 6C/ES2 GW BMAL2 IHC.png]

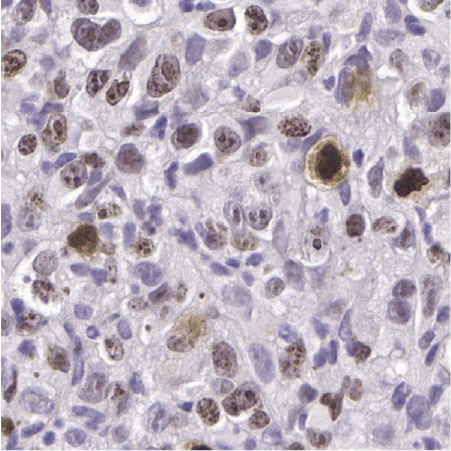

Supplement: Supplementary file 13 — Source data Fig. 6 [file 44321_2026_414_MOESM13_ESM.zip › EMM-2025-22875-V3-Figure_6_Source_Data-sd_V3/Fig. 6/Fig. 6C/ES2 GW Ki67 IHC.png]

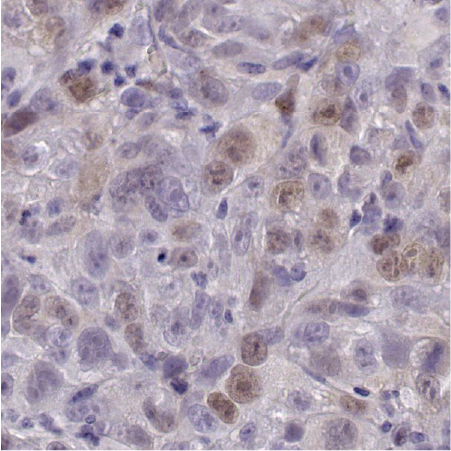

Supplement: Supplementary file 13 — Source data Fig. 6 [file 44321_2026_414_MOESM13_ESM.zip › EMM-2025-22875-V3-Figure_6_Source_Data-sd_V3/Fig. 6/Fig. 6C/ES2 GW RAD51 IHC.png]

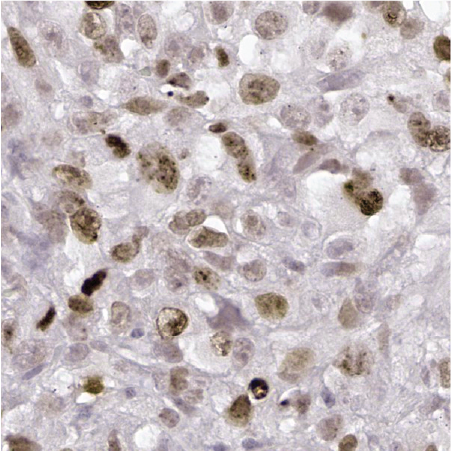

Supplement: Supplementary file 13 — Source data Fig. 6 [file 44321_2026_414_MOESM13_ESM.zip › EMM-2025-22875-V3-Figure_6_Source_Data-sd_V3/Fig. 6/Fig. 6C/ES2 GW yH2ax IHC.png]

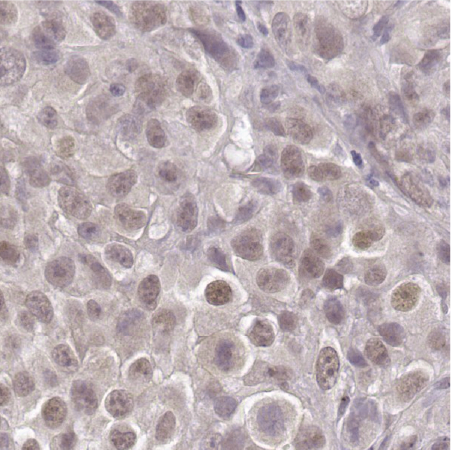

Supplement: Supplementary file 13 — Source data Fig. 6 [file 44321_2026_414_MOESM13_ESM.zip › EMM-2025-22875-V3-Figure_6_Source_Data-sd_V3/Fig. 6/Fig. 6C/ES2 Vehicle BMAL2 IHC.png]

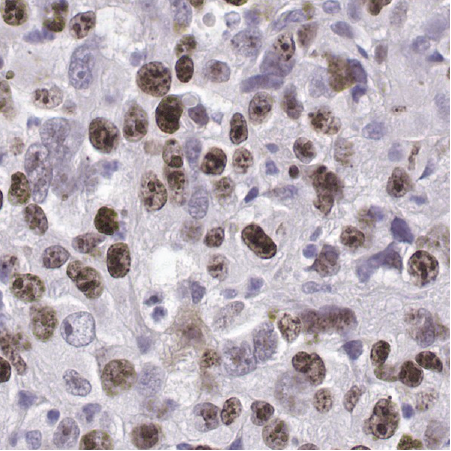

Supplement: Supplementary file 13 — Source data Fig. 6 [file 44321_2026_414_MOESM13_ESM.zip › EMM-2025-22875-V3-Figure_6_Source_Data-sd_V3/Fig. 6/Fig. 6C/ES2 Vehicle Ki67 IHC.png]

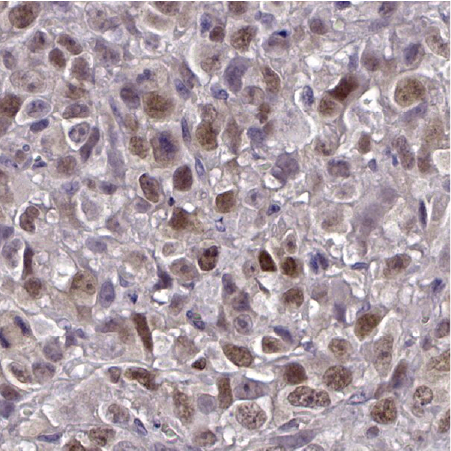

Supplement: Supplementary file 13 — Source data Fig. 6 [file 44321_2026_414_MOESM13_ESM.zip › EMM-2025-22875-V3-Figure_6_Source_Data-sd_V3/Fig. 6/Fig. 6C/ES2 Vehicle RAD51 IHC.png]

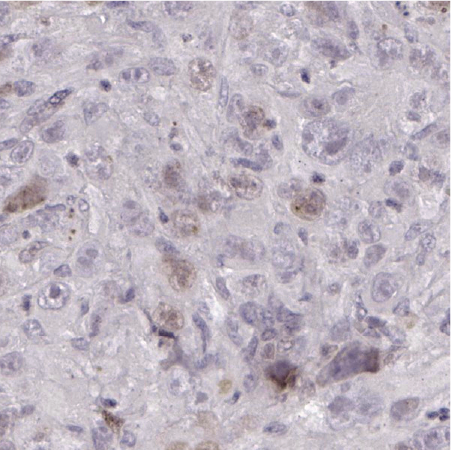

Supplement: Supplementary file 13 — Source data Fig. 6 [file 44321_2026_414_MOESM13_ESM.zip › EMM-2025-22875-V3-Figure_6_Source_Data-sd_V3/Fig. 6/Fig. 6C/ES2 Vehicle yh2ax IHC.png]

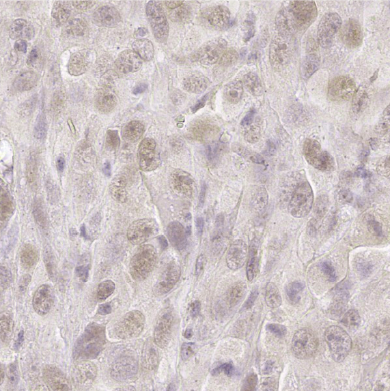

Supplement: Supplementary file 13 — Source data Fig. 6 [file 44321_2026_414_MOESM13_ESM.zip › EMM-2025-22875-V3-Figure_6_Source_Data-sd_V3/Fig. 6/Fig. 6E/JHOC5 GW BMAL2 IHC.png]

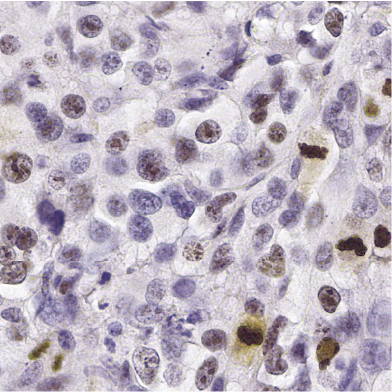

Supplement: Supplementary file 13 — Source data Fig. 6 [file 44321_2026_414_MOESM13_ESM.zip › EMM-2025-22875-V3-Figure_6_Source_Data-sd_V3/Fig. 6/Fig. 6E/JHOC5 GW Ki67 IHC.png]

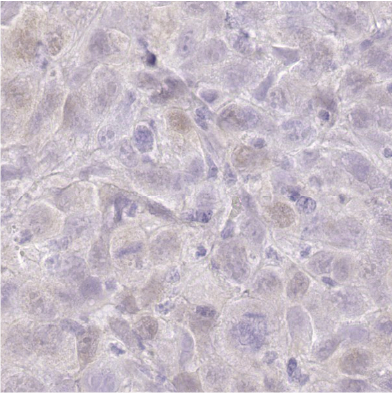

Supplement: Supplementary file 13 — Source data Fig. 6 [file 44321_2026_414_MOESM13_ESM.zip › EMM-2025-22875-V3-Figure_6_Source_Data-sd_V3/Fig. 6/Fig. 6E/JHOC5 GW RAD51 IHC.png]

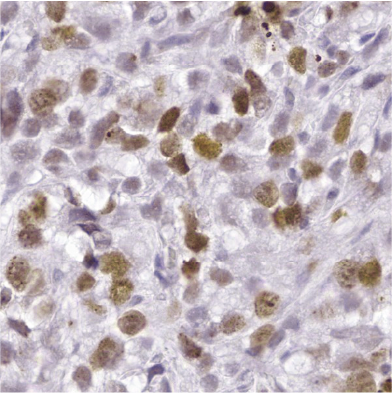

Supplement: Supplementary file 13 — Source data Fig. 6 [file 44321_2026_414_MOESM13_ESM.zip › EMM-2025-22875-V3-Figure_6_Source_Data-sd_V3/Fig. 6/Fig. 6E/JHOC5 GW yH2AX IHC.png]

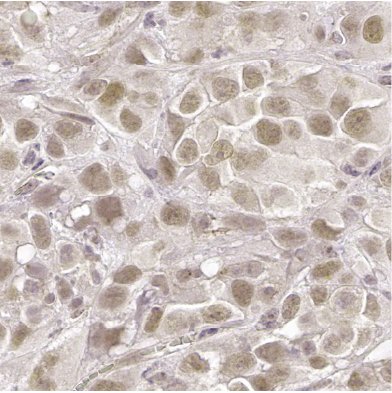

Supplement: Supplementary file 13 — Source data Fig. 6 [file 44321_2026_414_MOESM13_ESM.zip › EMM-2025-22875-V3-Figure_6_Source_Data-sd_V3/Fig. 6/Fig. 6E/JHOC5 Vehicle BMAL2 IHC.png]

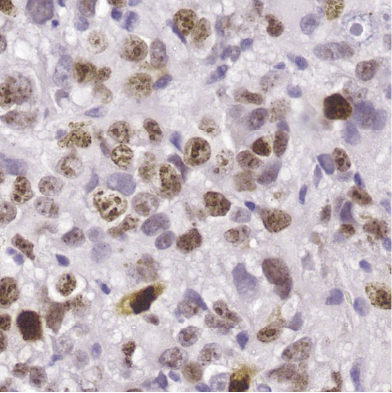

Supplement: Supplementary file 13 — Source data Fig. 6 [file 44321_2026_414_MOESM13_ESM.zip › EMM-2025-22875-V3-Figure_6_Source_Data-sd_V3/Fig. 6/Fig. 6E/JHOC5 Vehicle Ki67 IHC.png]

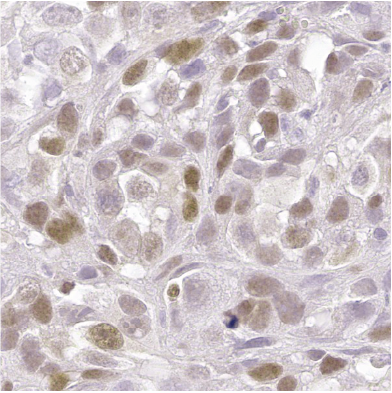

Supplement: Supplementary file 13 — Source data Fig. 6 [file 44321_2026_414_MOESM13_ESM.zip › EMM-2025-22875-V3-Figure_6_Source_Data-sd_V3/Fig. 6/Fig. 6E/JHOC5 Vehicle RAD51 IHC.png]

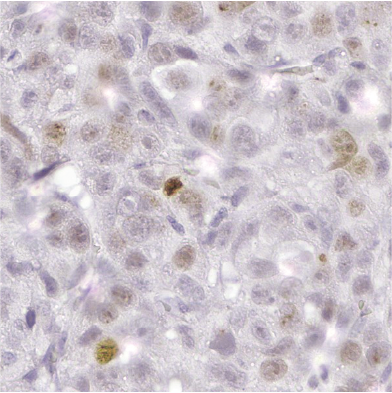

Supplement: Supplementary file 13 — Source data Fig. 6 [file 44321_2026_414_MOESM13_ESM.zip › EMM-2025-22875-V3-Figure_6_Source_Data-sd_V3/Fig. 6/Fig. 6E/JHOC5 Vehicle yH2AX IHC.png]

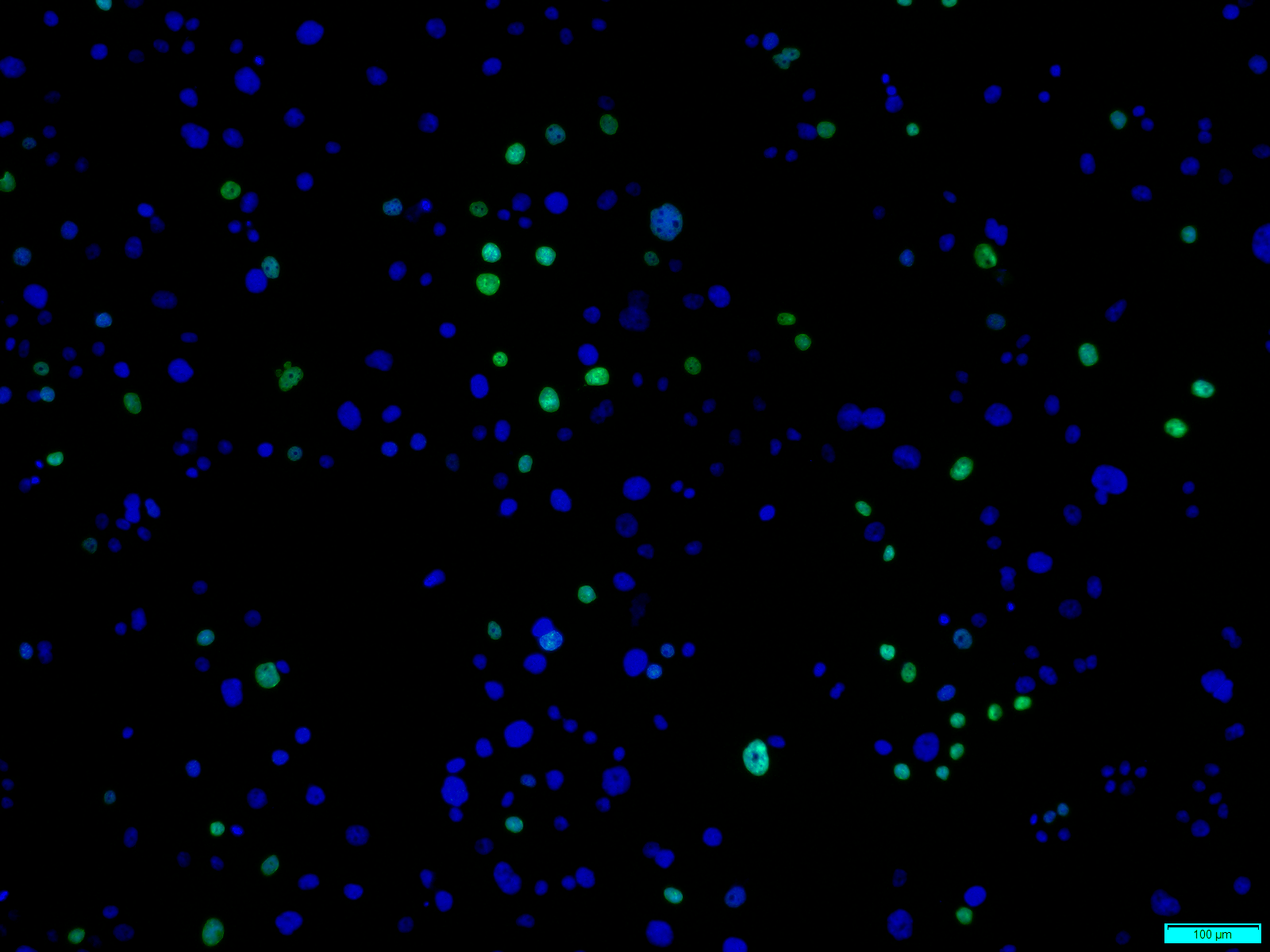

Supplement: Supplementary file 14 — Figure EV1 Source Data [file 44321_2026_414_MOESM14_ESM.zip › Fig. EV1/EV1A/ES-2 shBMAL2#1.tif]

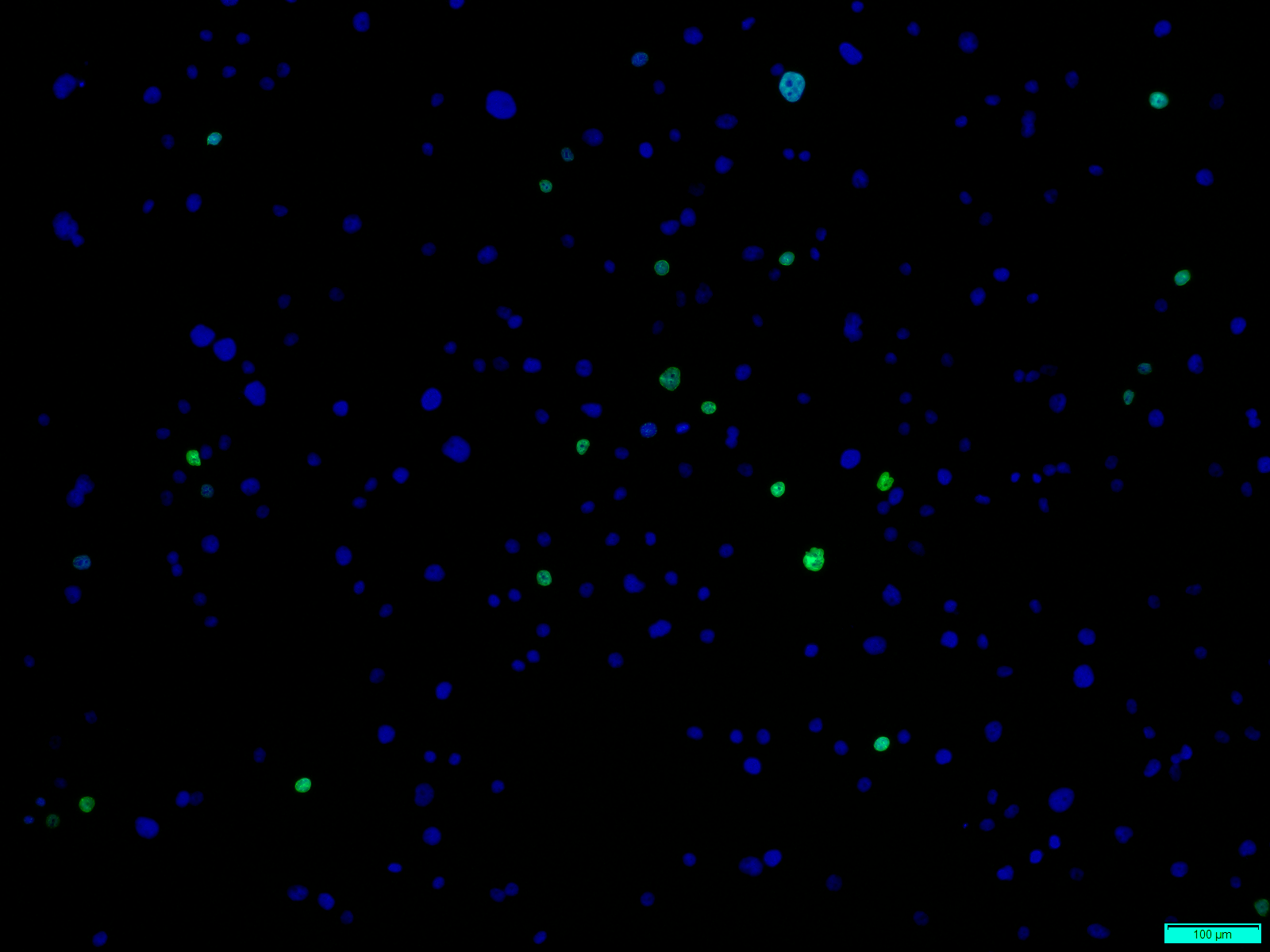

Supplement: Supplementary file 14 — Figure EV1 Source Data [file 44321_2026_414_MOESM14_ESM.zip › Fig. EV1/EV1A/ES-2 shBMAL2#2.tif]

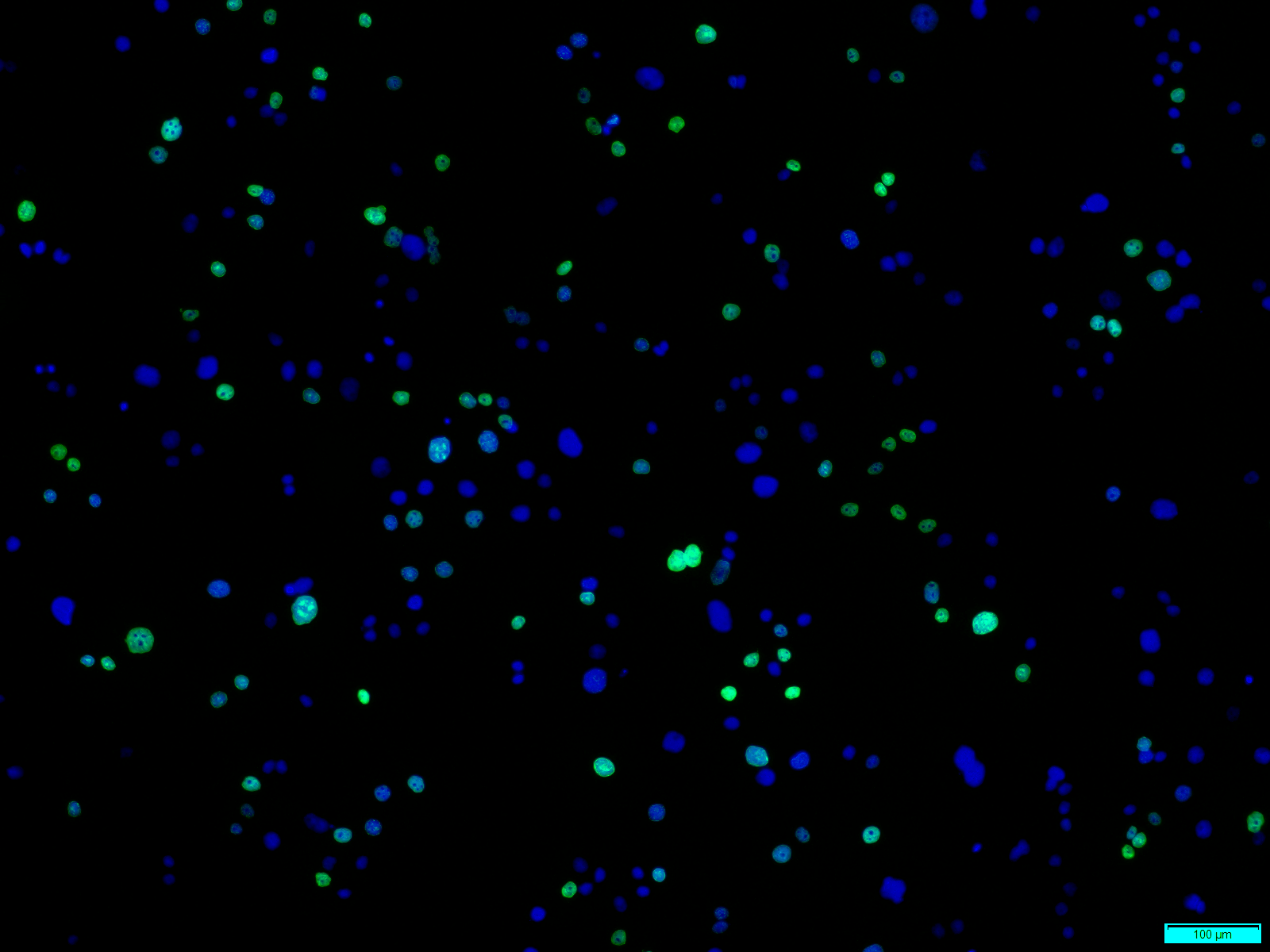

Supplement: Supplementary file 14 — Figure EV1 Source Data [file 44321_2026_414_MOESM14_ESM.zip › Fig. EV1/EV1A/ES-2 shCtrl.tif]

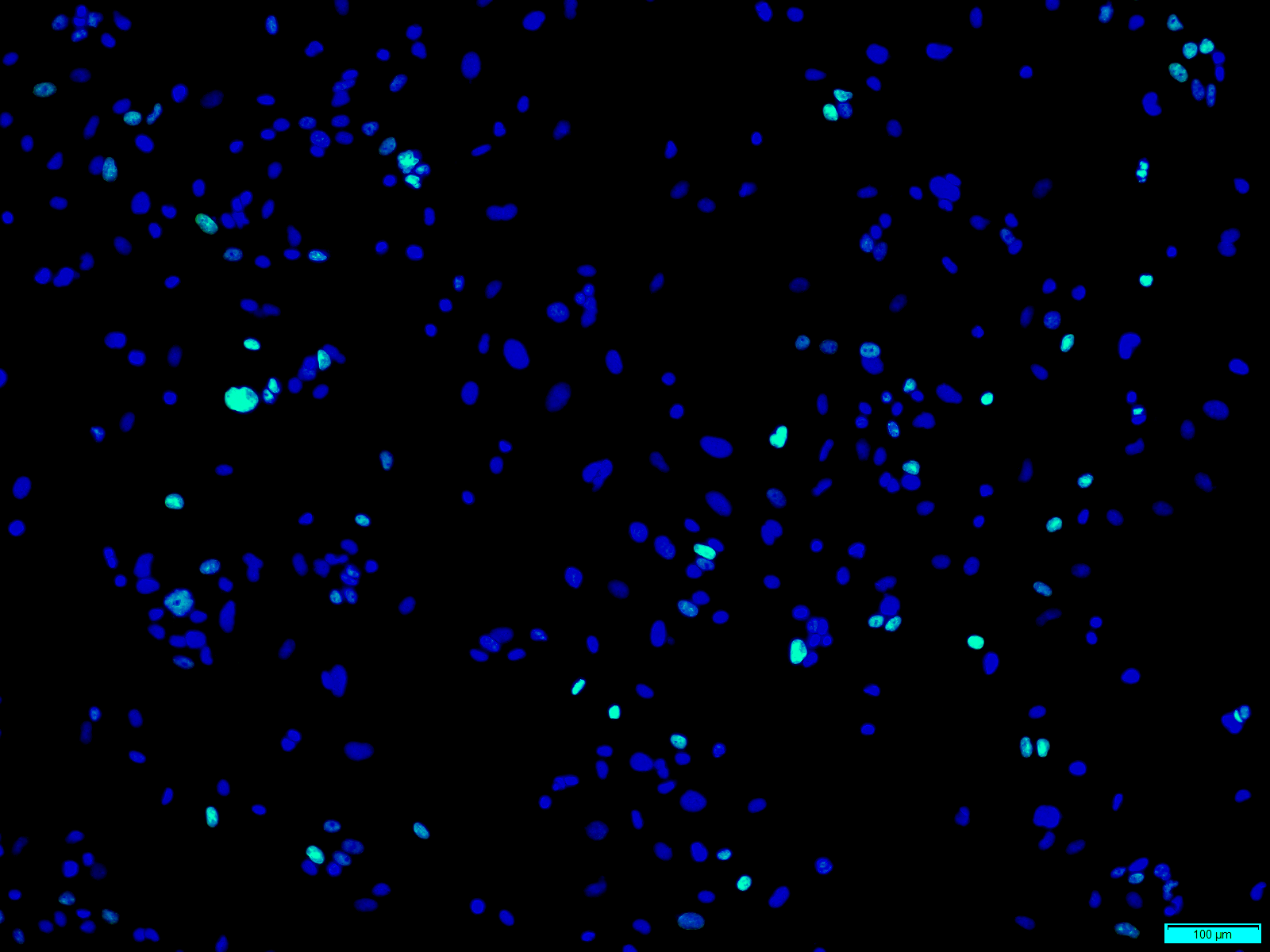

Supplement: Supplementary file 14 — Figure EV1 Source Data [file 44321_2026_414_MOESM14_ESM.zip › Fig. EV1/EV1A/JHOC5 shBMAL2#1.tif]

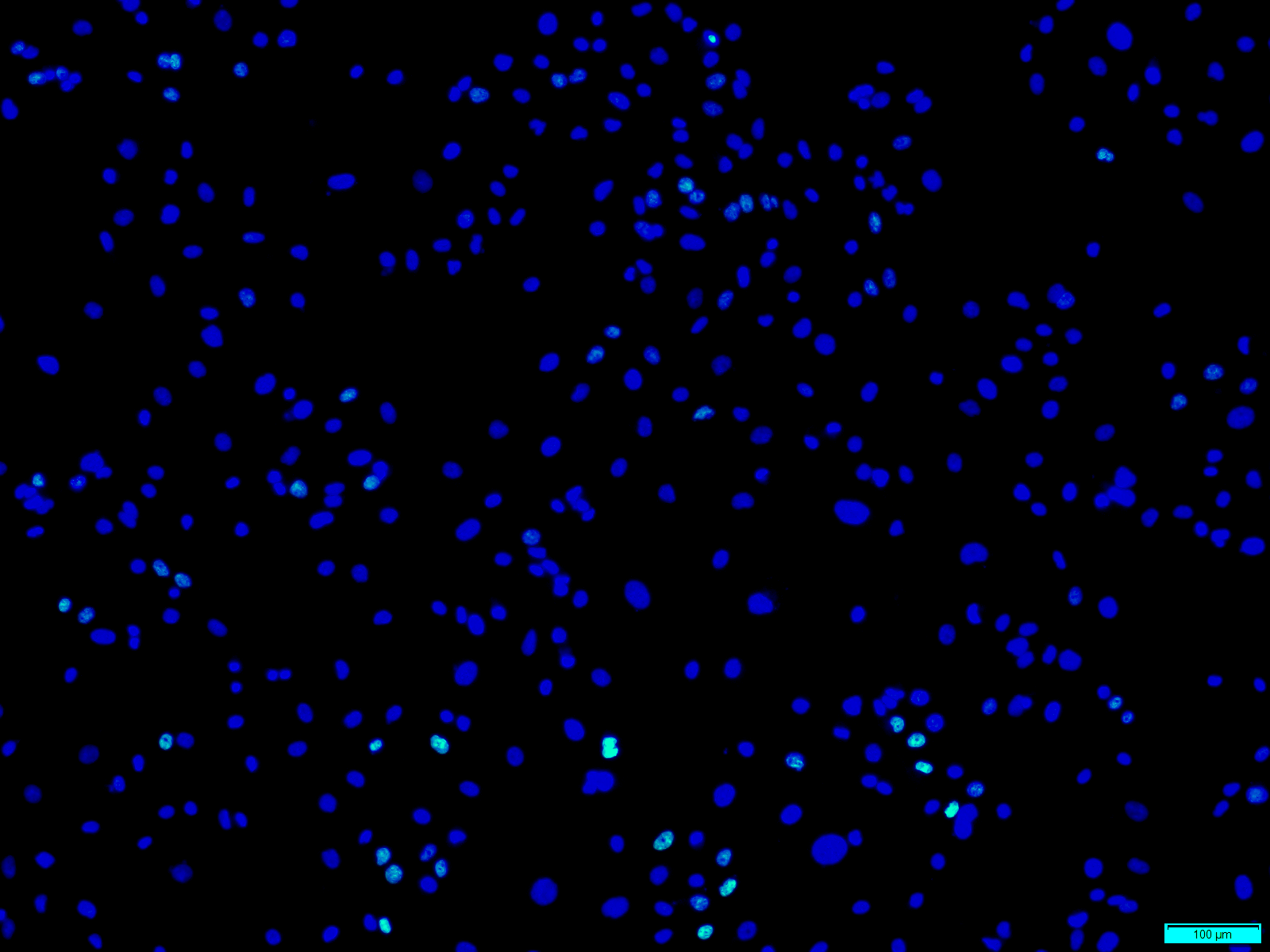

Supplement: Supplementary file 14 — Figure EV1 Source Data [file 44321_2026_414_MOESM14_ESM.zip › Fig. EV1/EV1A/JHOC5 shBMAL2#2.tif]

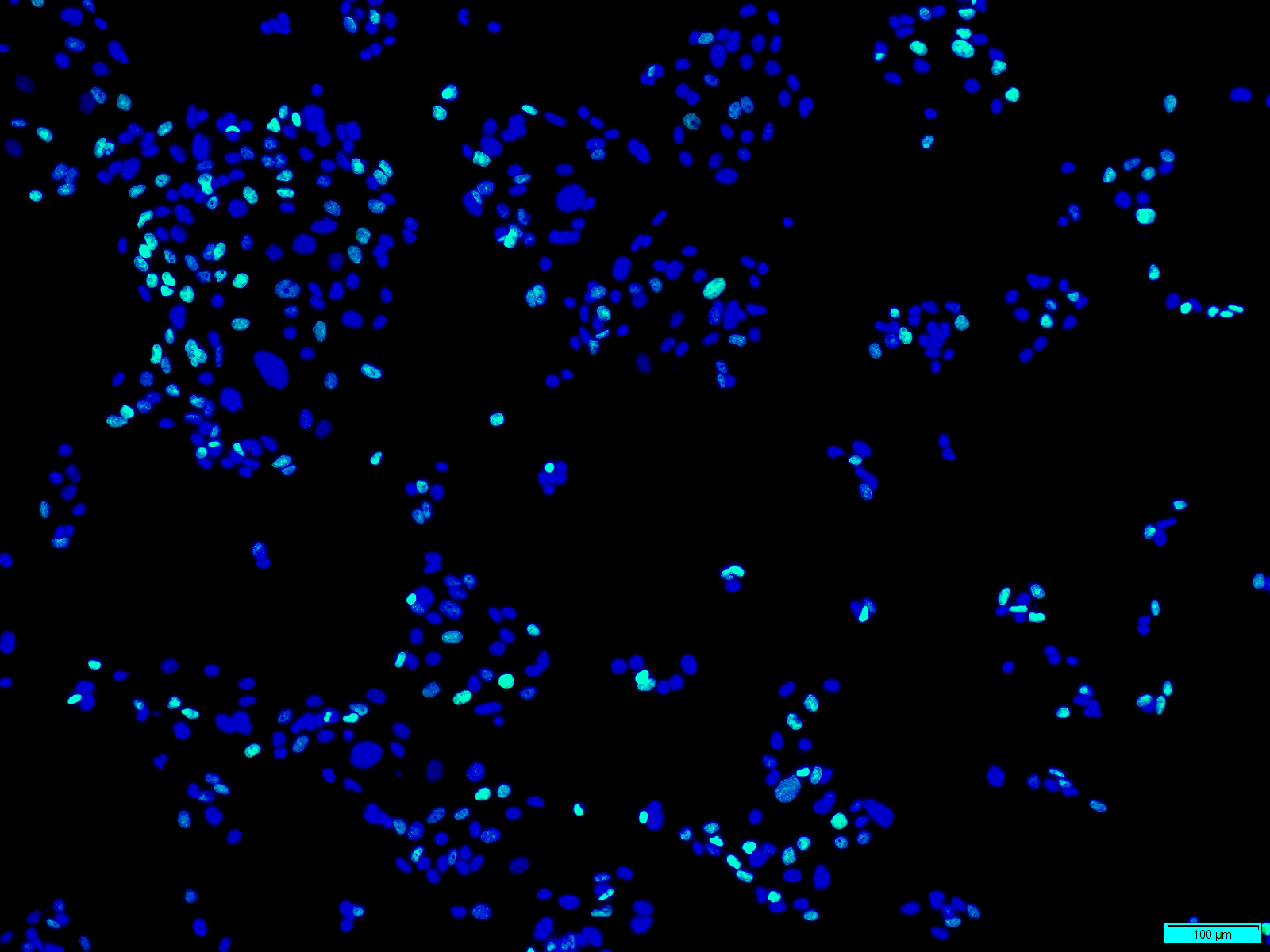

Supplement: Supplementary file 14 — Figure EV1 Source Data [file 44321_2026_414_MOESM14_ESM.zip › Fig. EV1/EV1A/JHOC5 shCtrl.tif]

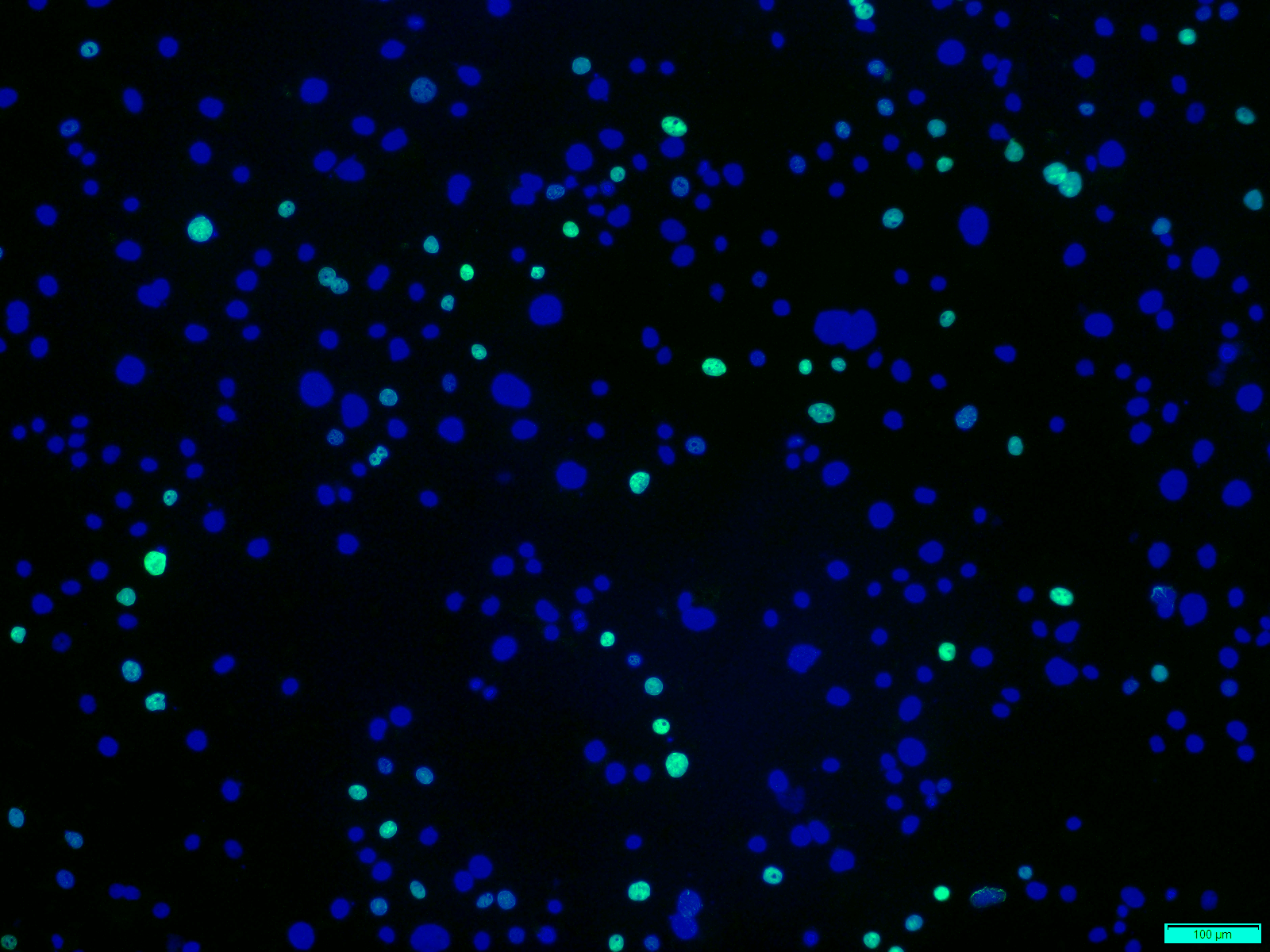

Supplement: Supplementary file 14 — Figure EV1 Source Data [file 44321_2026_414_MOESM14_ESM.zip › Fig. EV1/EV1A/JHOC9 shBMAL2#1.tif]

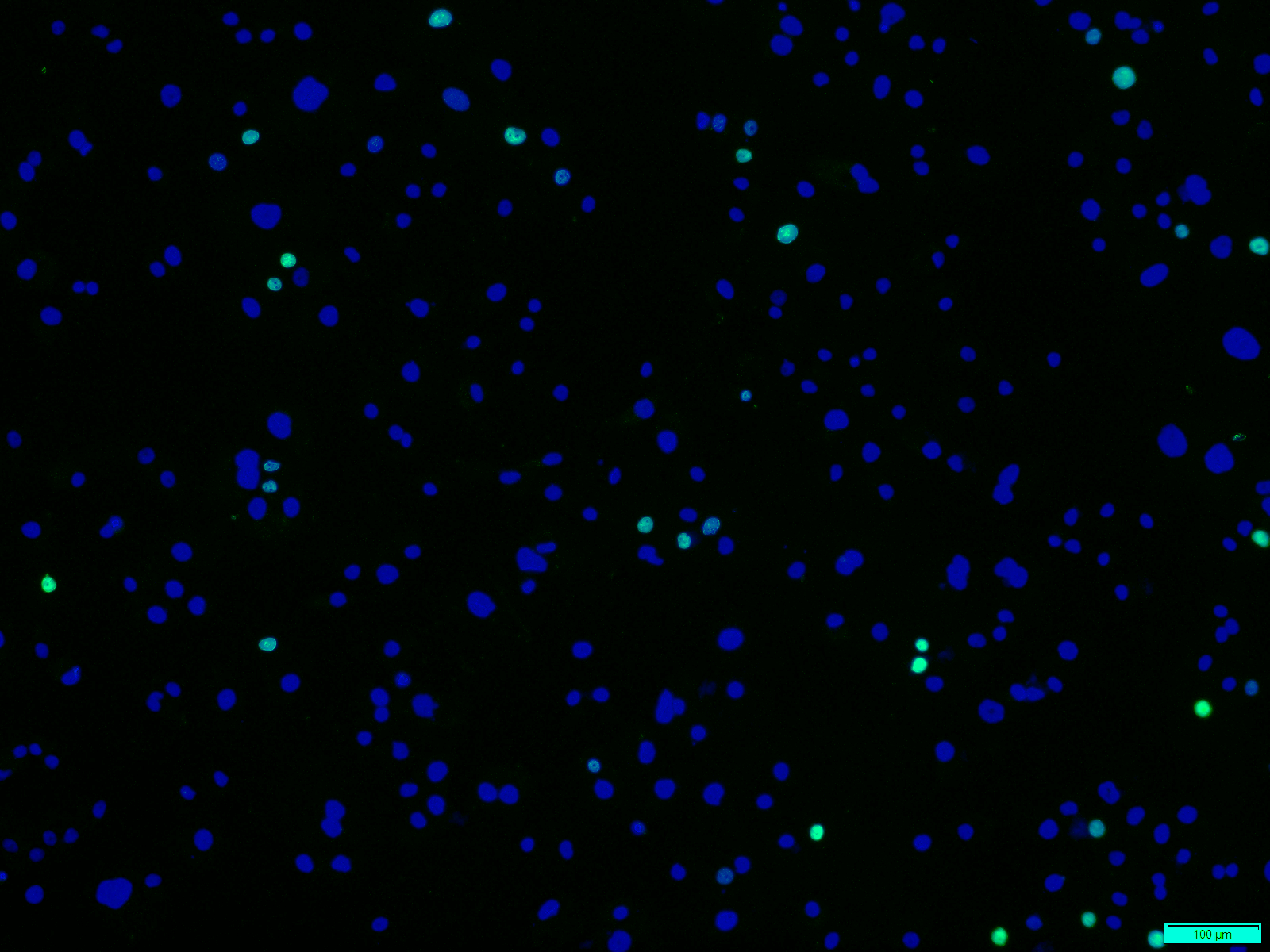

Supplement: Supplementary file 14 — Figure EV1 Source Data [file 44321_2026_414_MOESM14_ESM.zip › Fig. EV1/EV1A/JHOC9 shBMAL2#2.tif]

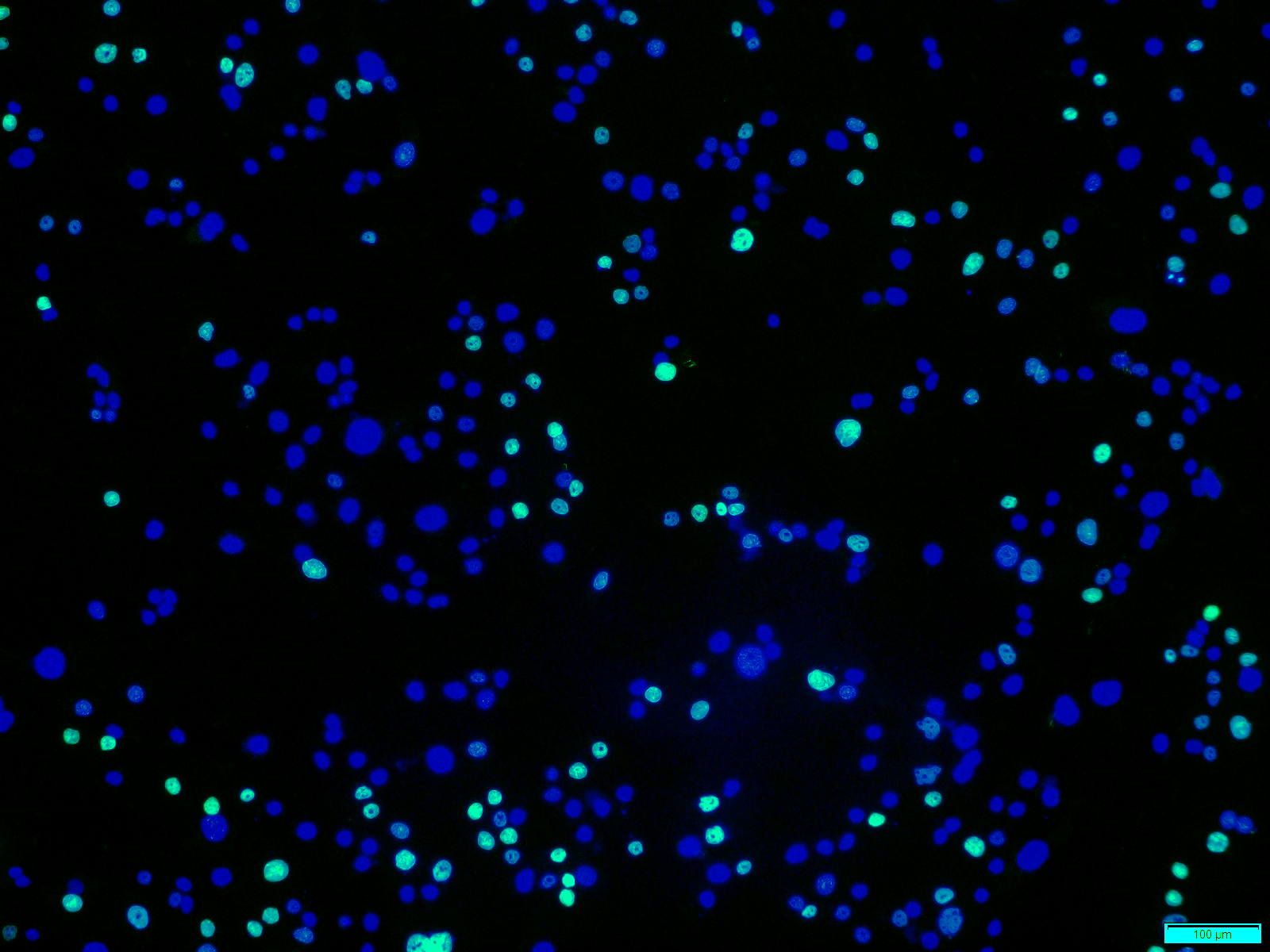

Supplement: Supplementary file 14 — Figure EV1 Source Data [file 44321_2026_414_MOESM14_ESM.zip › Fig. EV1/EV1A/JHOC9 shCtrl.tif]
