# Supplementary figures and images for: BMAL2 is a druggable target for ovarian clear cell carcinoma (OCCC) (part 2 of 3)
Source: EMBO Mol Med. 2026 Apr 3;18(5):1933–66. doi: 10.1038/s44321-026-00414-8 (PMC13179388; doi:10.1038/s44321-026-00414-8)

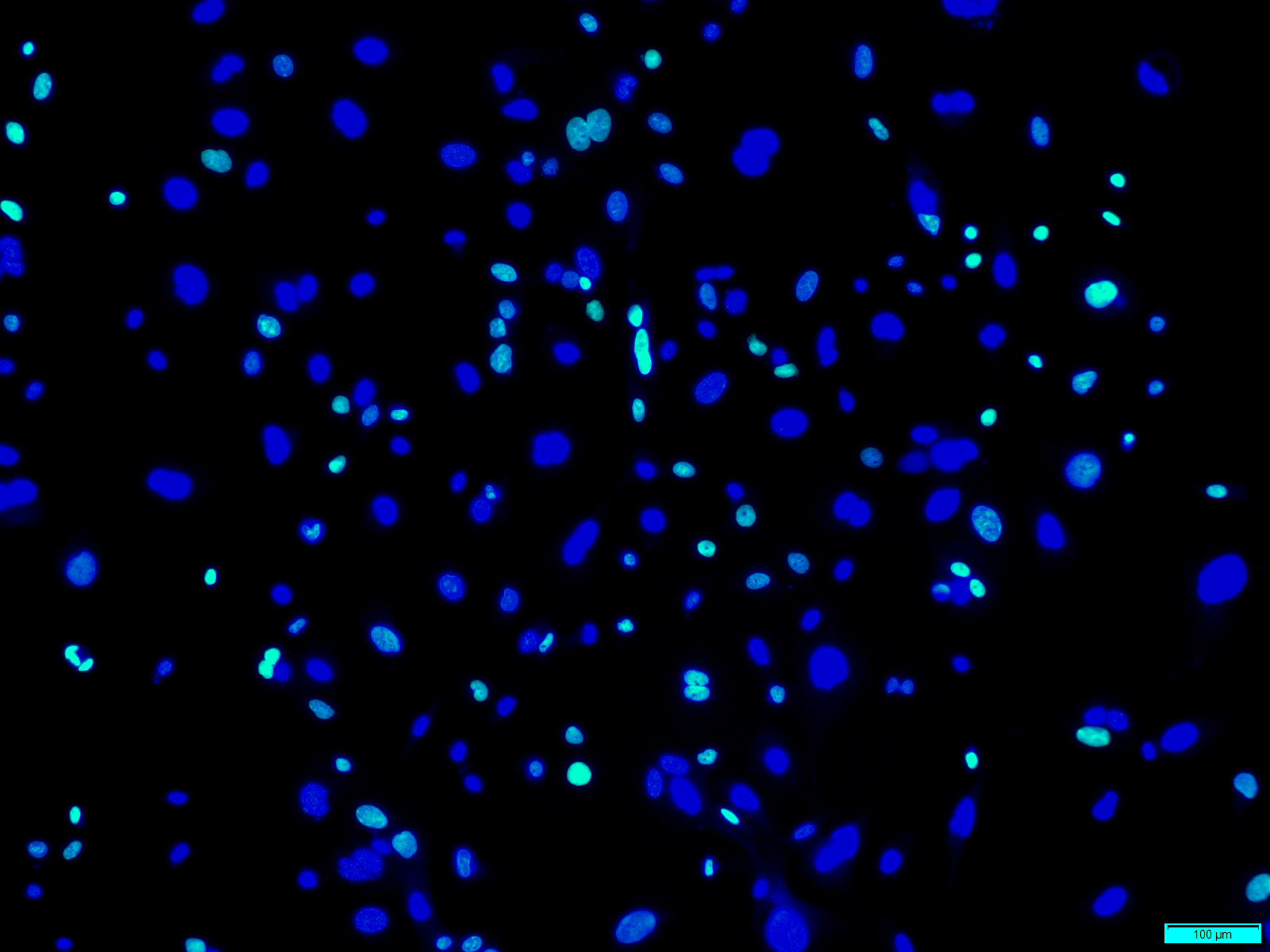

Supplement: Supplementary file 14 — Figure EV1 Source Data [file 44321_2026_414_MOESM14_ESM.zip › Fig. EV1/EV1A/OVCA429 shBMAL2#1.tif]

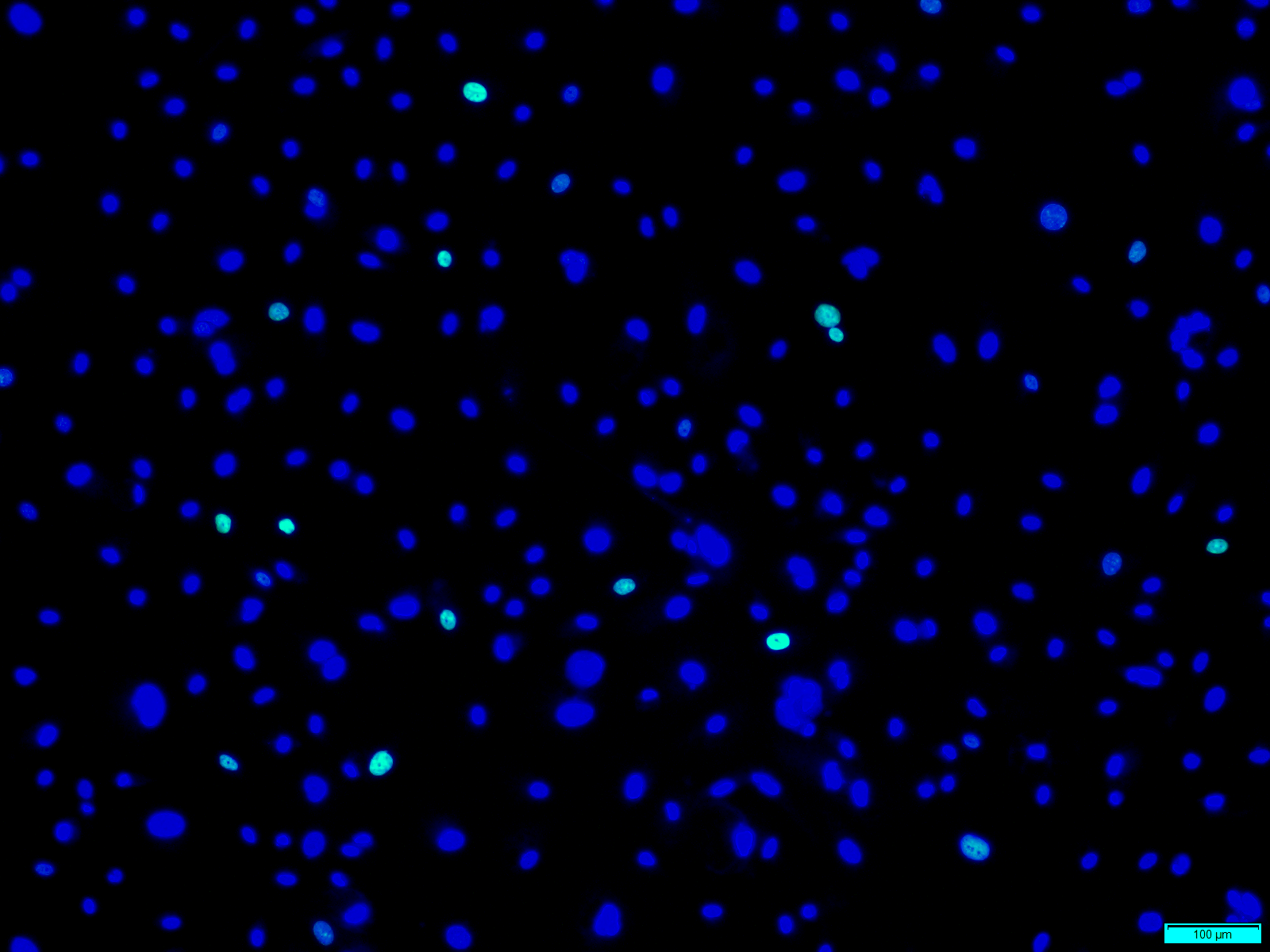

Supplement: Supplementary file 14 — Figure EV1 Source Data [file 44321_2026_414_MOESM14_ESM.zip › Fig. EV1/EV1A/OVCA429 shBMAL2#2.tif]

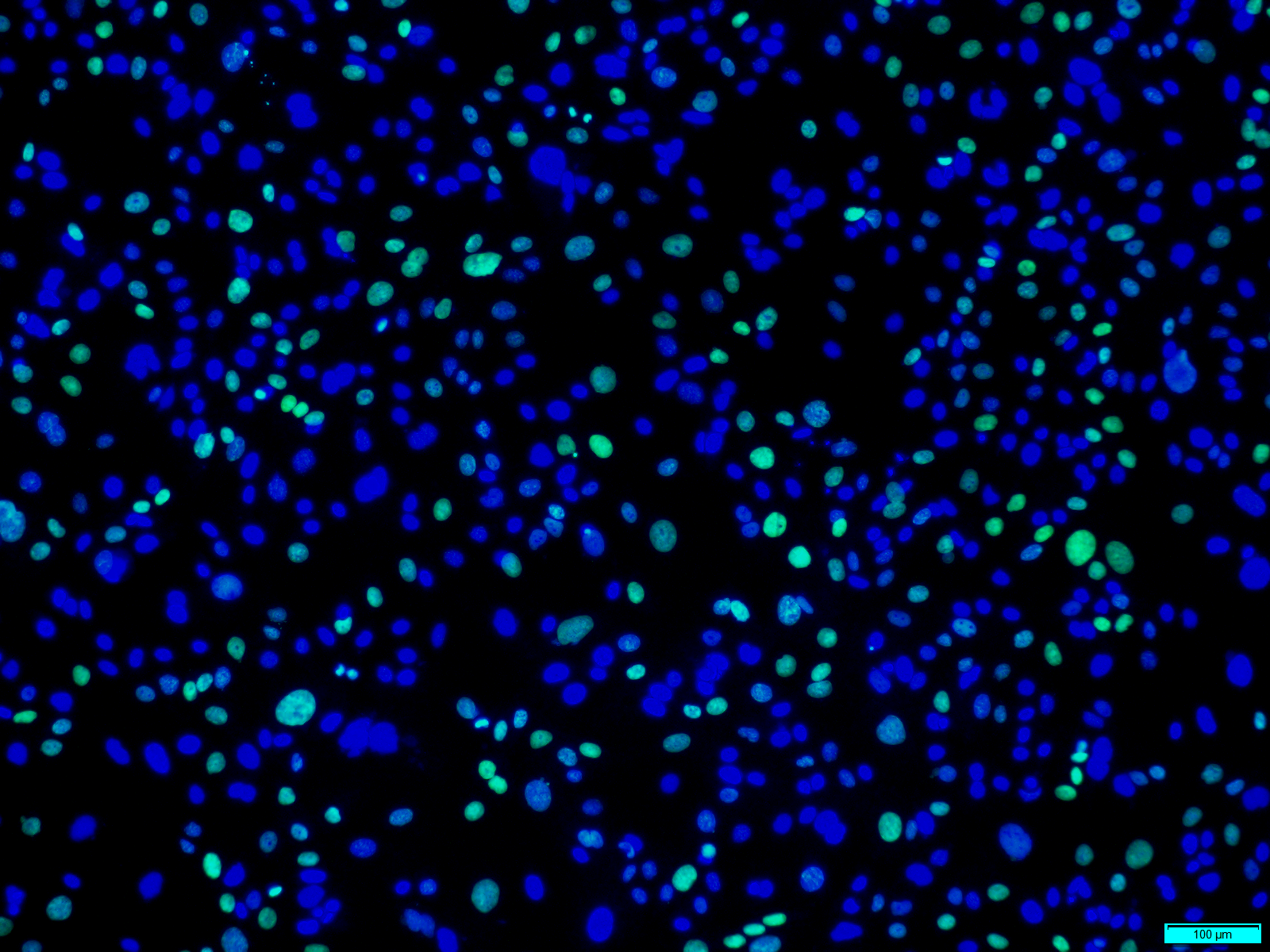

Supplement: Supplementary file 14 — Figure EV1 Source Data [file 44321_2026_414_MOESM14_ESM.zip › Fig. EV1/EV1A/OVCA429 shCtrl.tif]

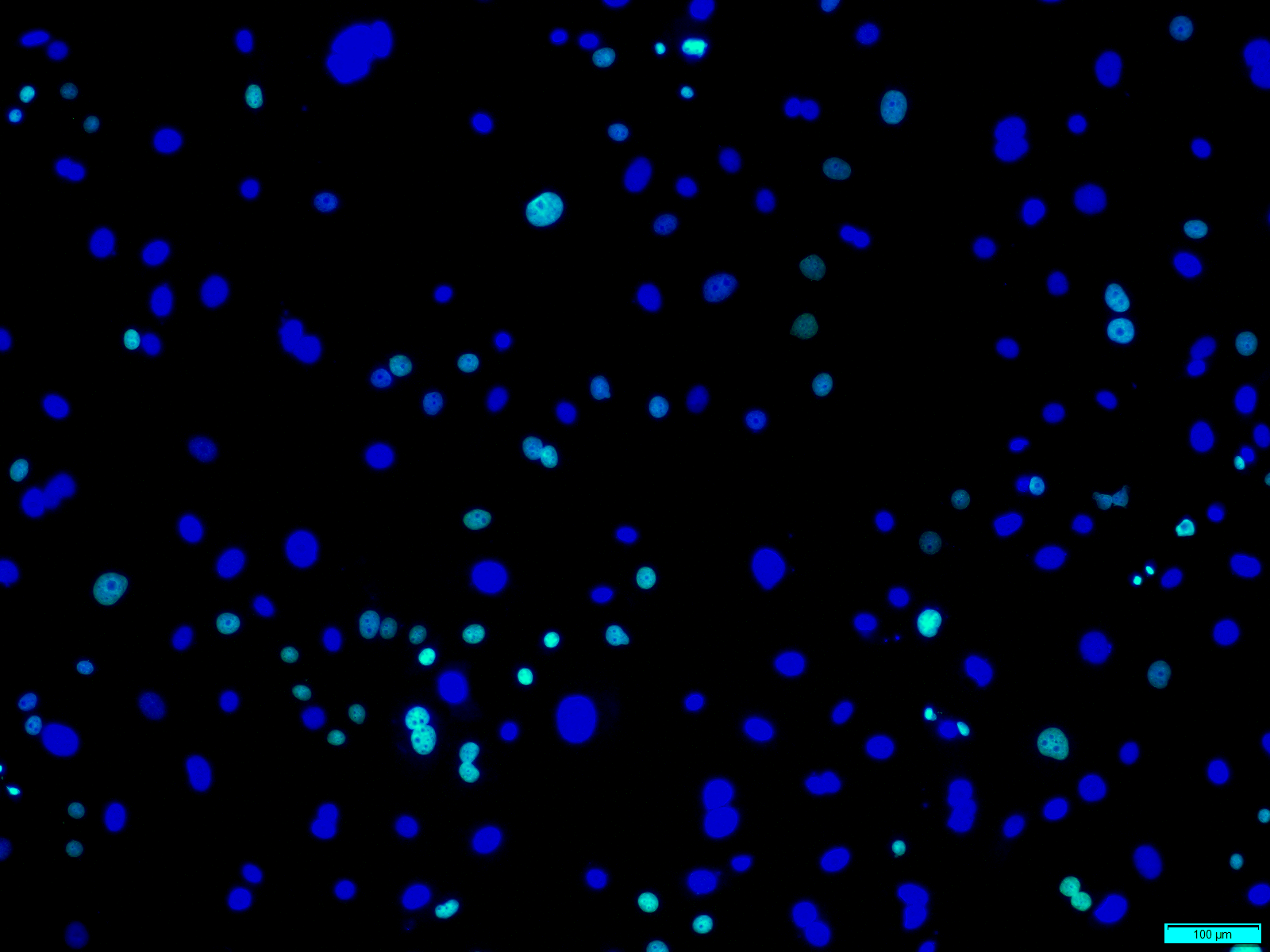

Supplement: Supplementary file 14 — Figure EV1 Source Data [file 44321_2026_414_MOESM14_ESM.zip › Fig. EV1/EV1A/OVISE shBMAL2#1.tif]

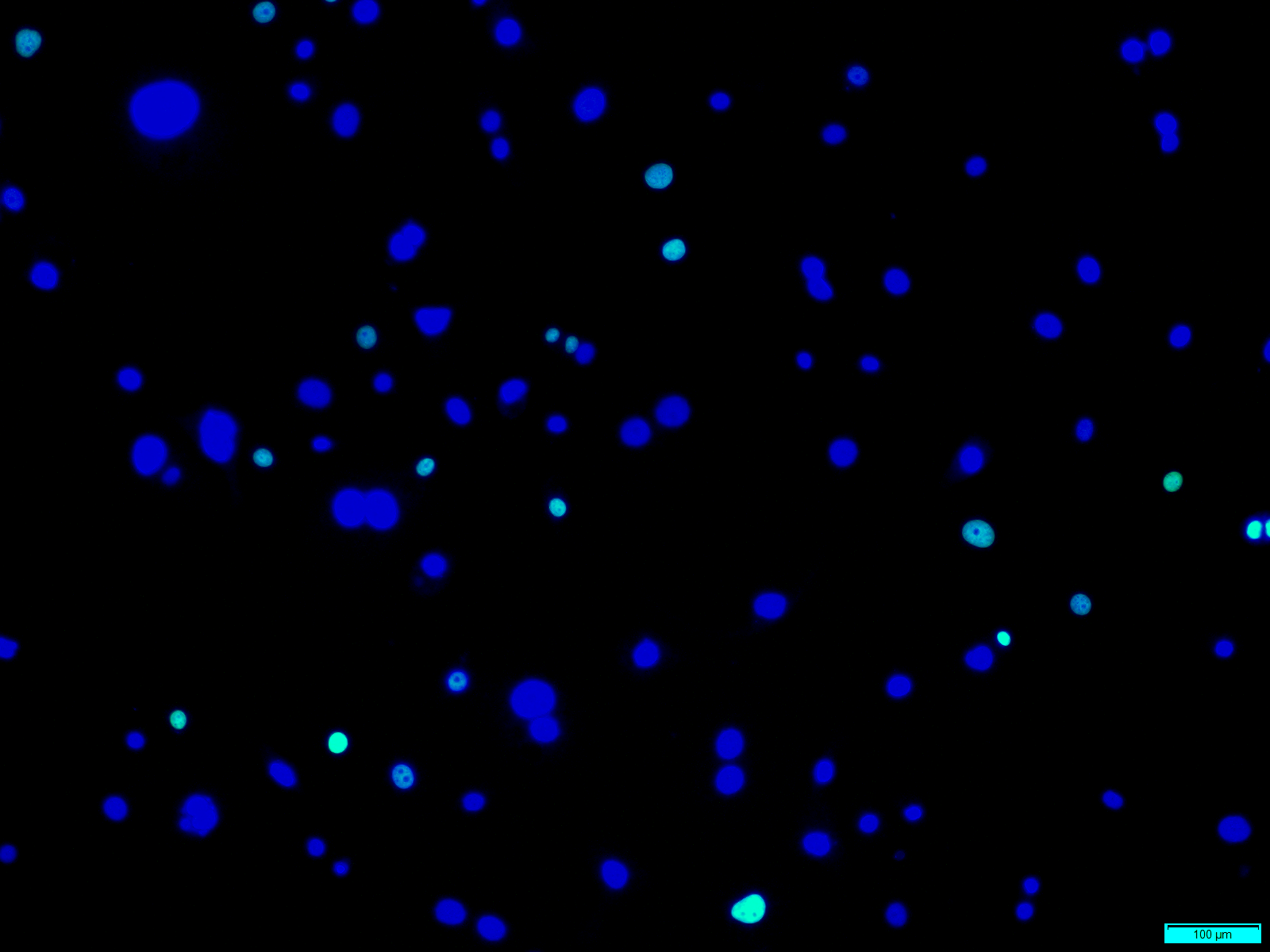

Supplement: Supplementary file 14 — Figure EV1 Source Data [file 44321_2026_414_MOESM14_ESM.zip › Fig. EV1/EV1A/OVISE shBMAL2#2.jpg]

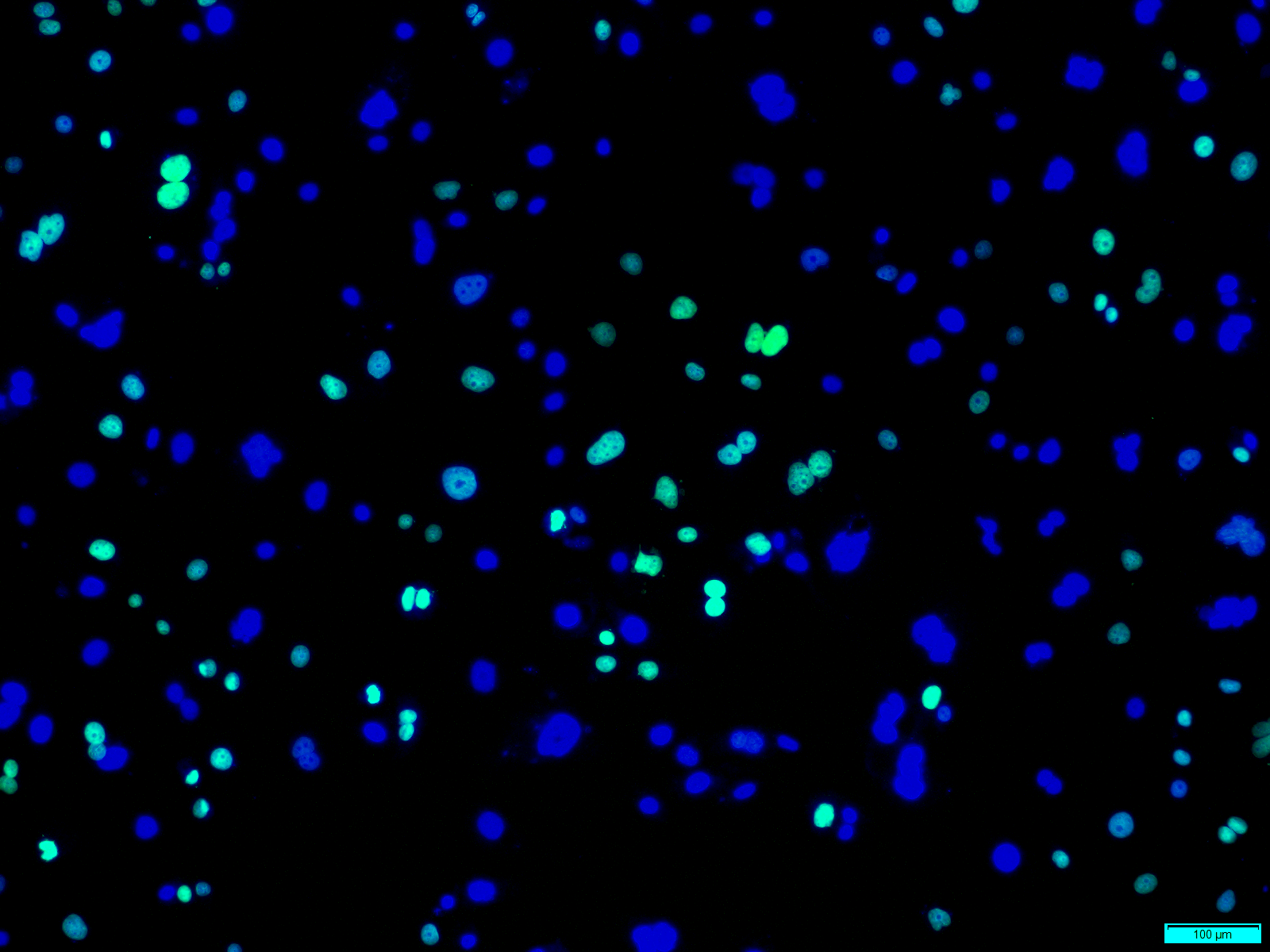

Supplement: Supplementary file 14 — Figure EV1 Source Data [file 44321_2026_414_MOESM14_ESM.zip › Fig. EV1/EV1A/OVISE shCtrl.tif]

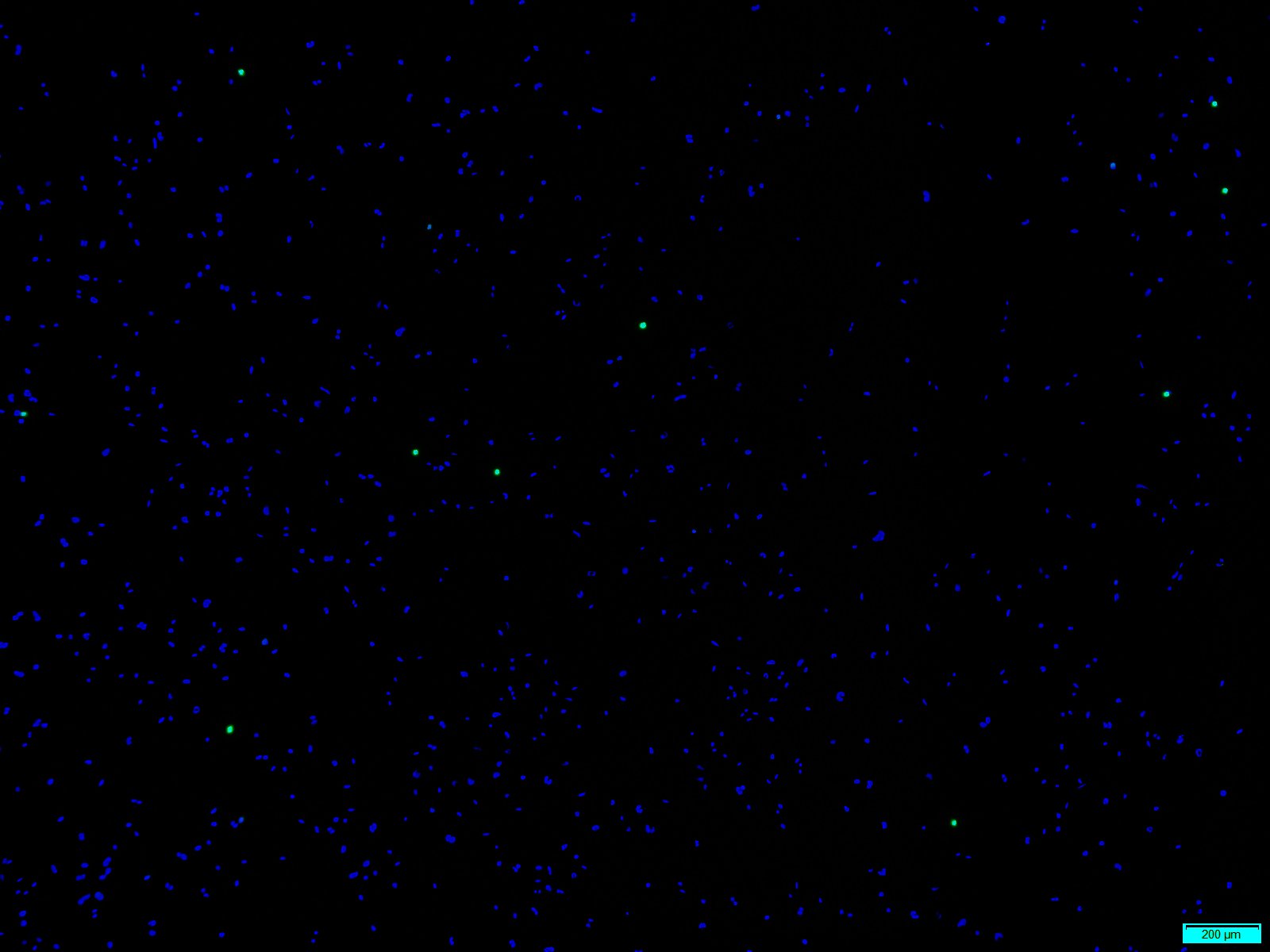

Supplement: Supplementary file 14 — Figure EV1 Source Data [file 44321_2026_414_MOESM14_ESM.zip › Fig. EV1/EV1B/ES-2 shBMAL2#1.jpg]

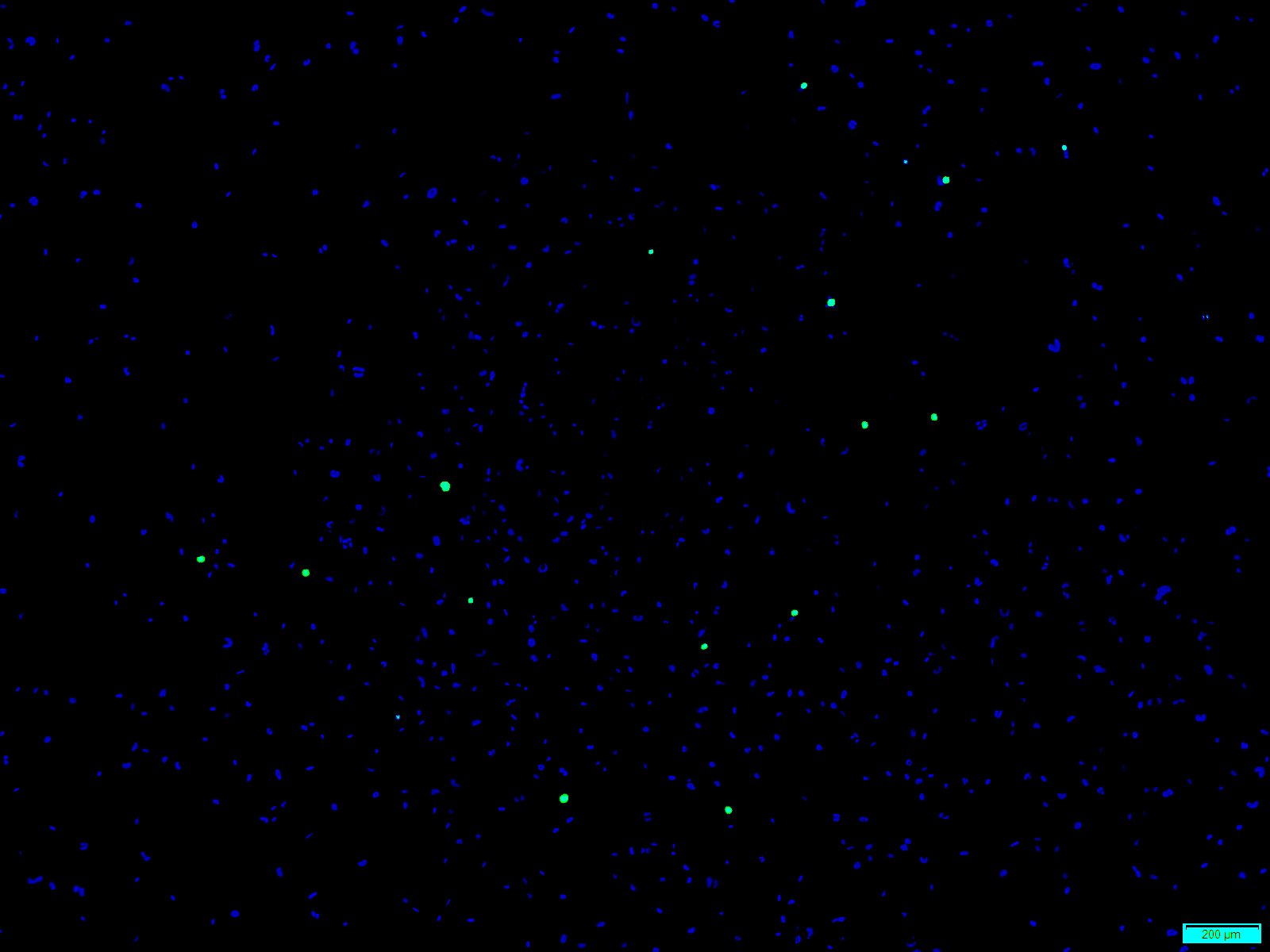

Supplement: Supplementary file 14 — Figure EV1 Source Data [file 44321_2026_414_MOESM14_ESM.zip › Fig. EV1/EV1B/ES-2 shBMAL2#2.jpg]

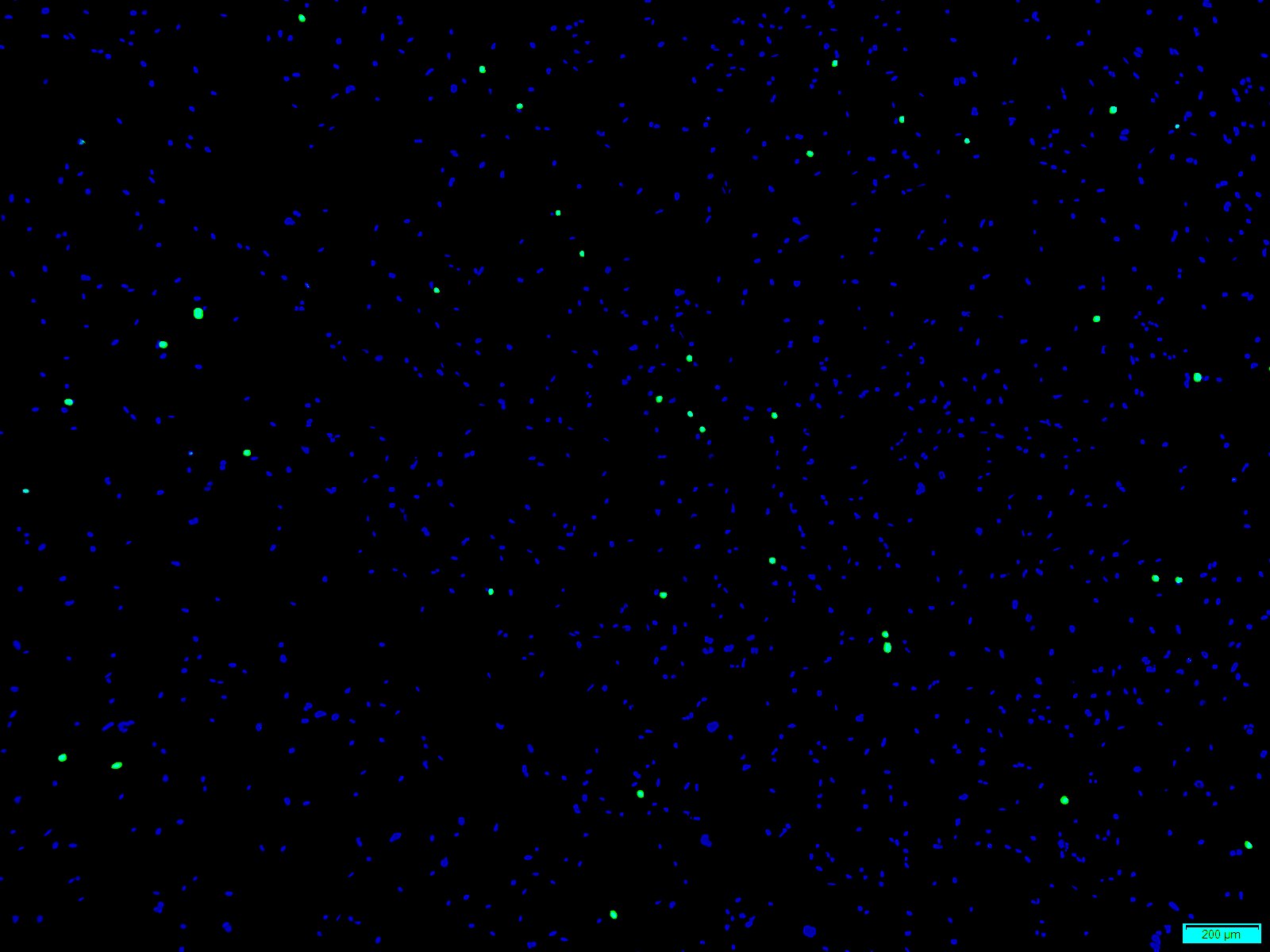

Supplement: Supplementary file 14 — Figure EV1 Source Data [file 44321_2026_414_MOESM14_ESM.zip › Fig. EV1/EV1B/ES-2 shCtrl.jpg]

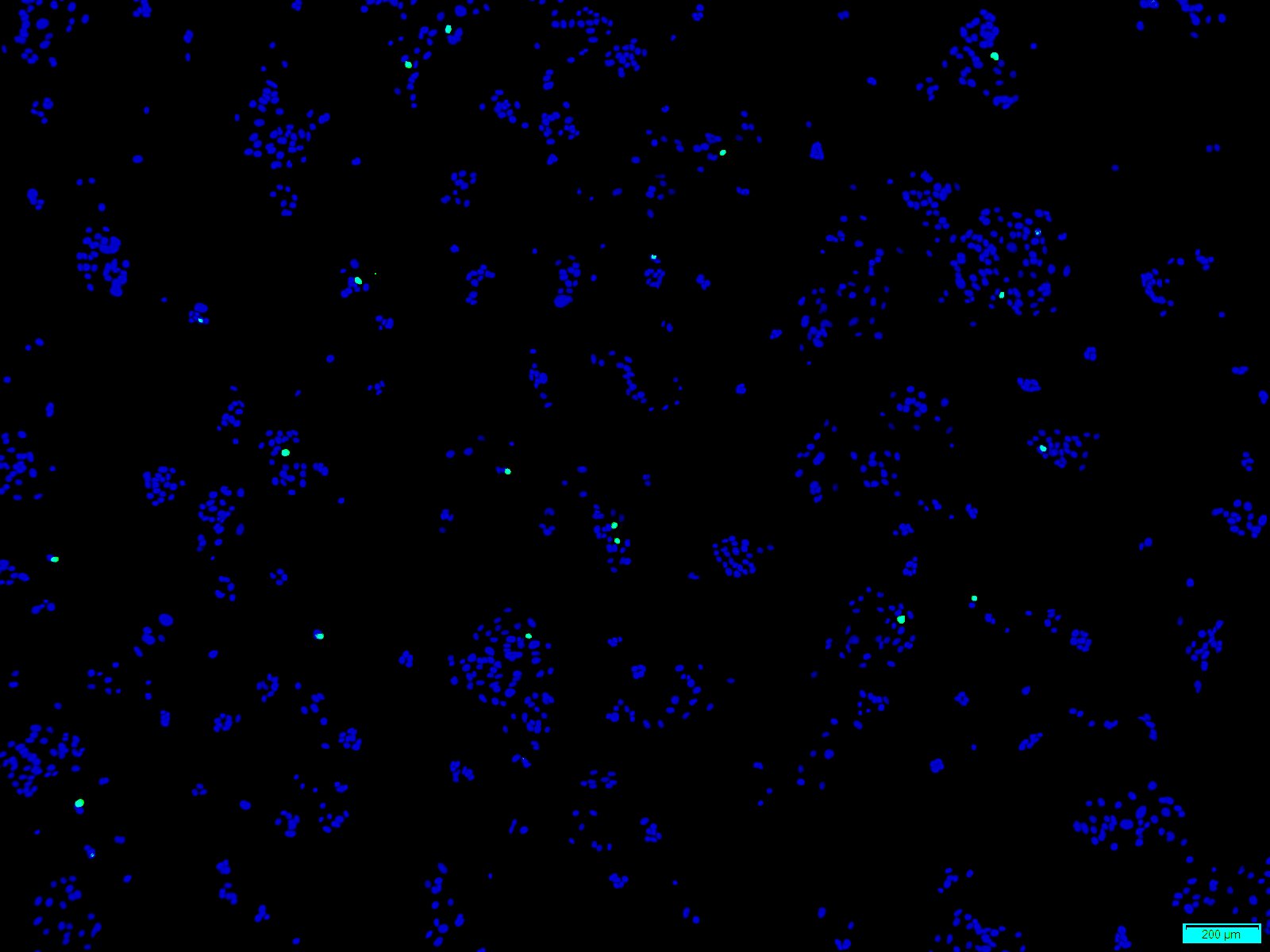

Supplement: Supplementary file 14 — Figure EV1 Source Data [file 44321_2026_414_MOESM14_ESM.zip › Fig. EV1/EV1B/JHOC5 shBMAL2#1.jpg]

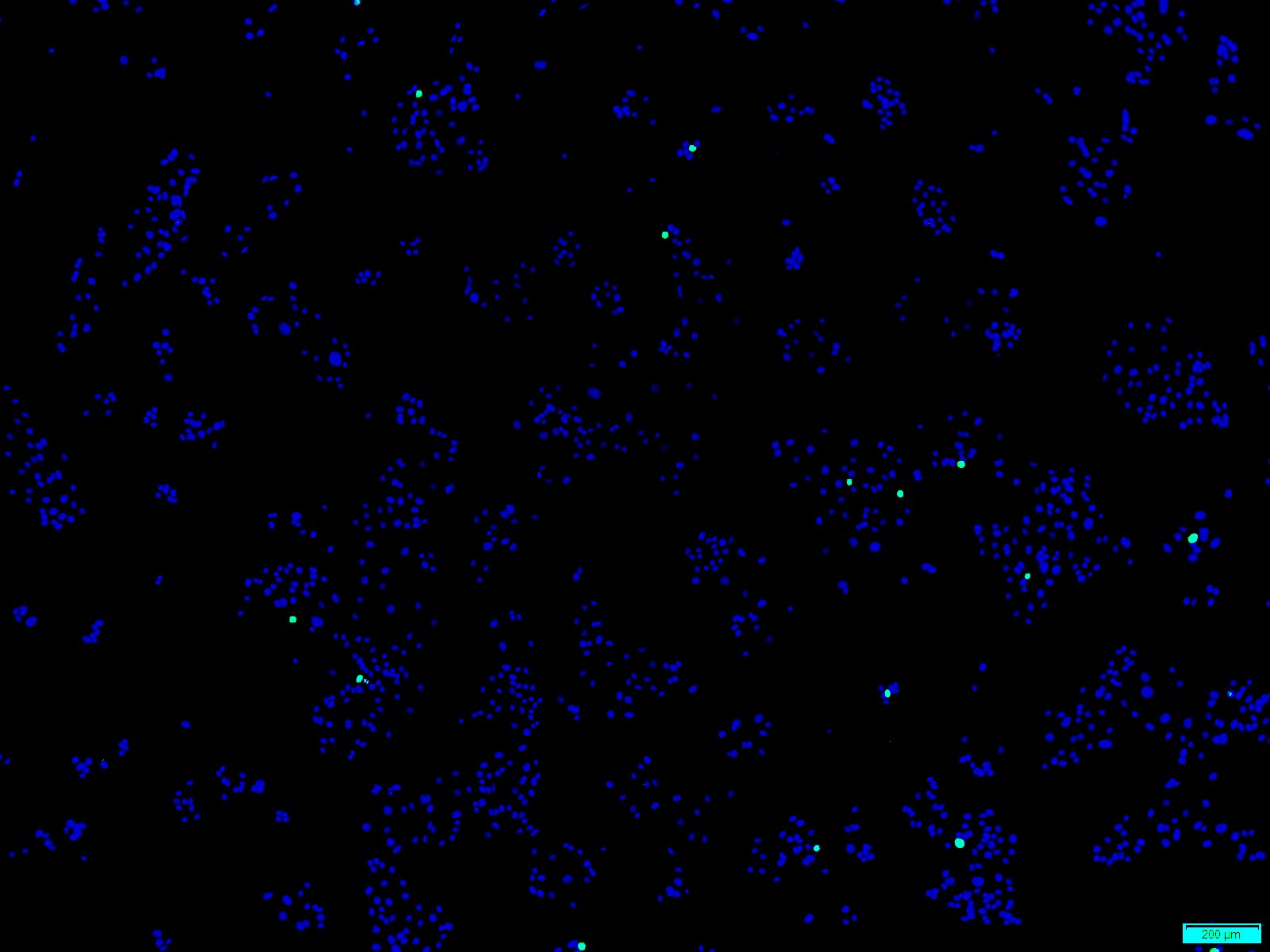

Supplement: Supplementary file 14 — Figure EV1 Source Data [file 44321_2026_414_MOESM14_ESM.zip › Fig. EV1/EV1B/JHOC5 shBMAL2#2.jpg]

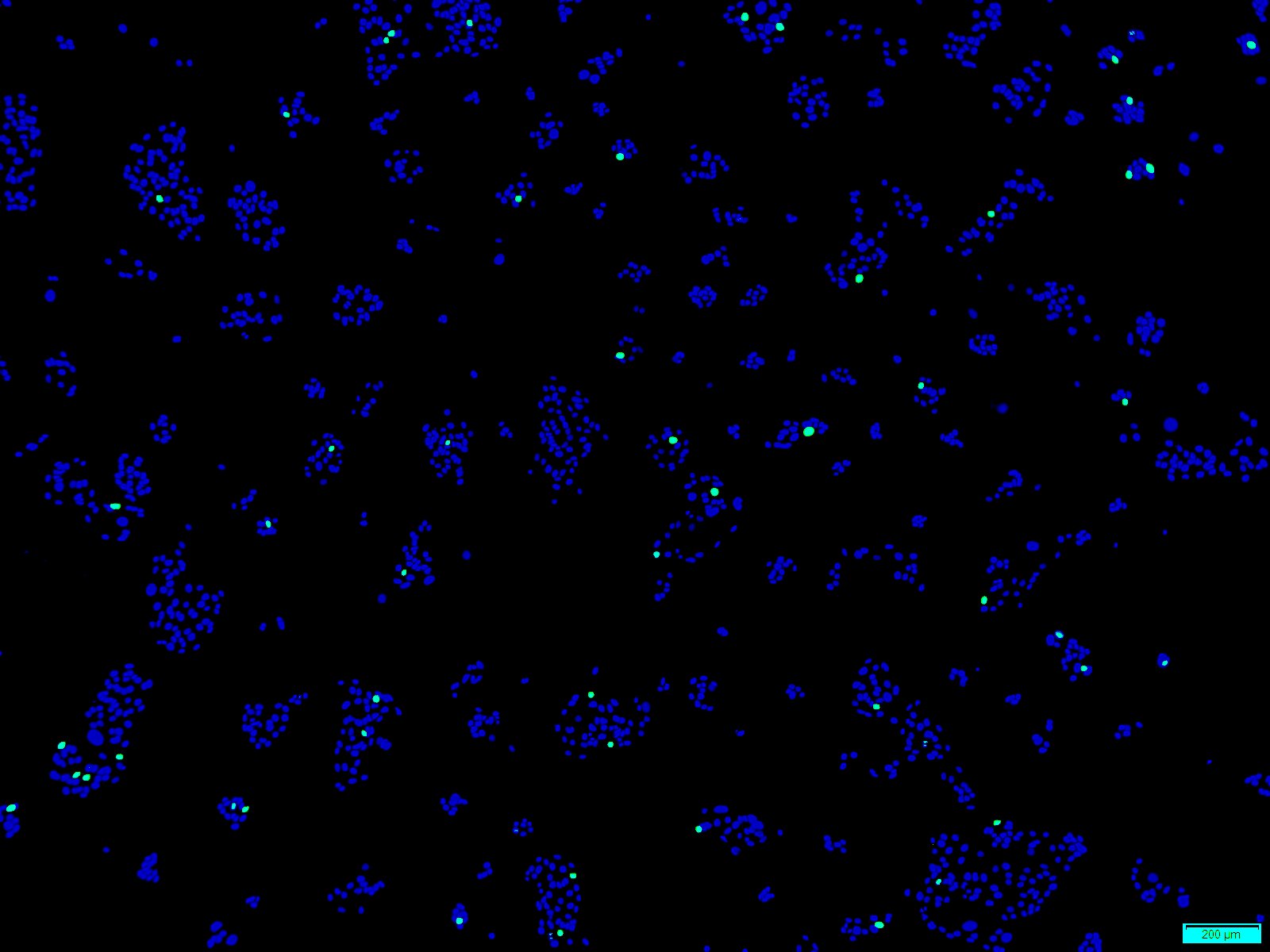

Supplement: Supplementary file 14 — Figure EV1 Source Data [file 44321_2026_414_MOESM14_ESM.zip › Fig. EV1/EV1B/JHOC5 shCtrl.jpg]

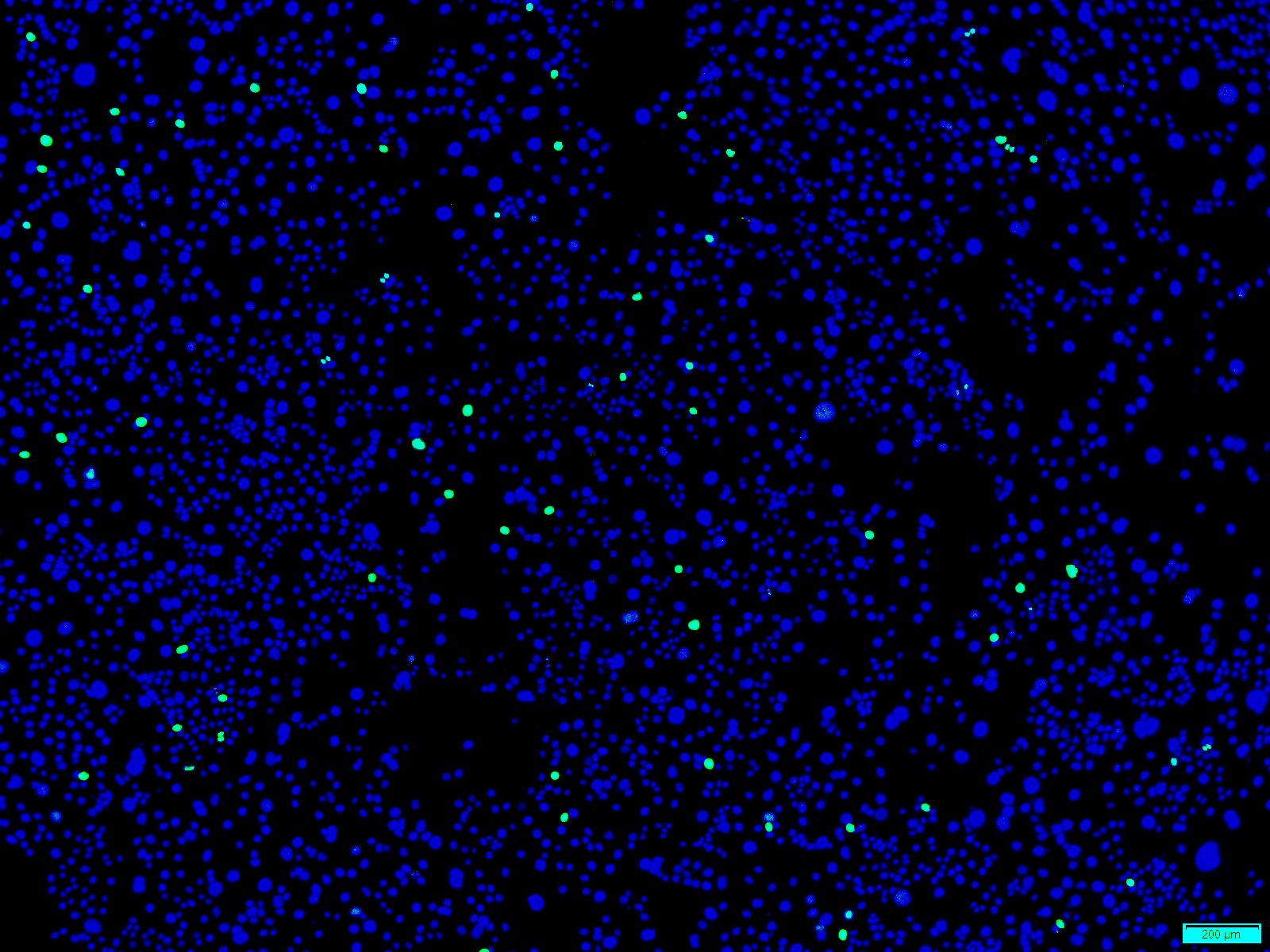

Supplement: Supplementary file 14 — Figure EV1 Source Data [file 44321_2026_414_MOESM14_ESM.zip › Fig. EV1/EV1B/JHOC9 shBMAL2#1.jpg]

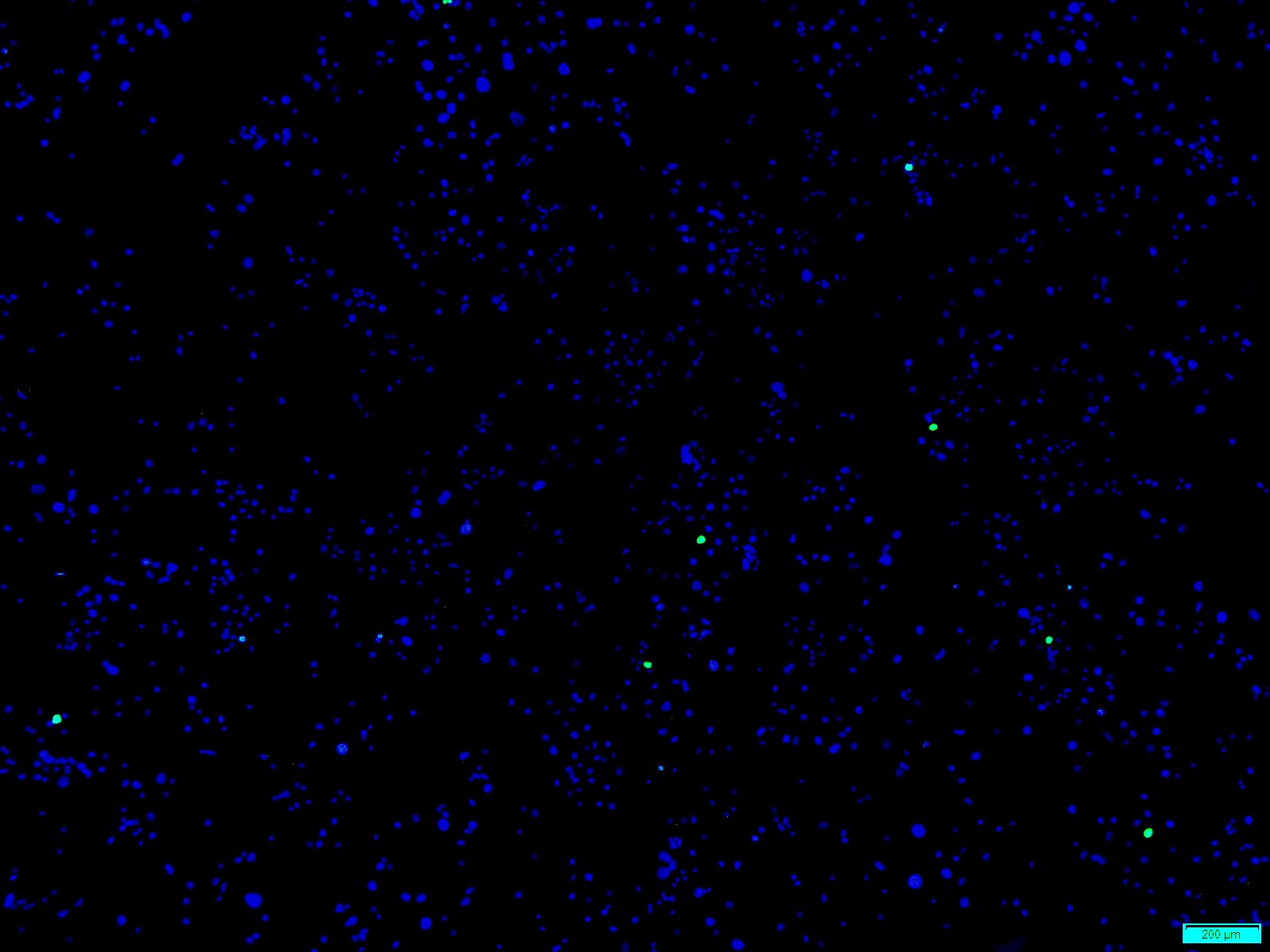

Supplement: Supplementary file 14 — Figure EV1 Source Data [file 44321_2026_414_MOESM14_ESM.zip › Fig. EV1/EV1B/JHOC9 shBMAL2#2.jpg]

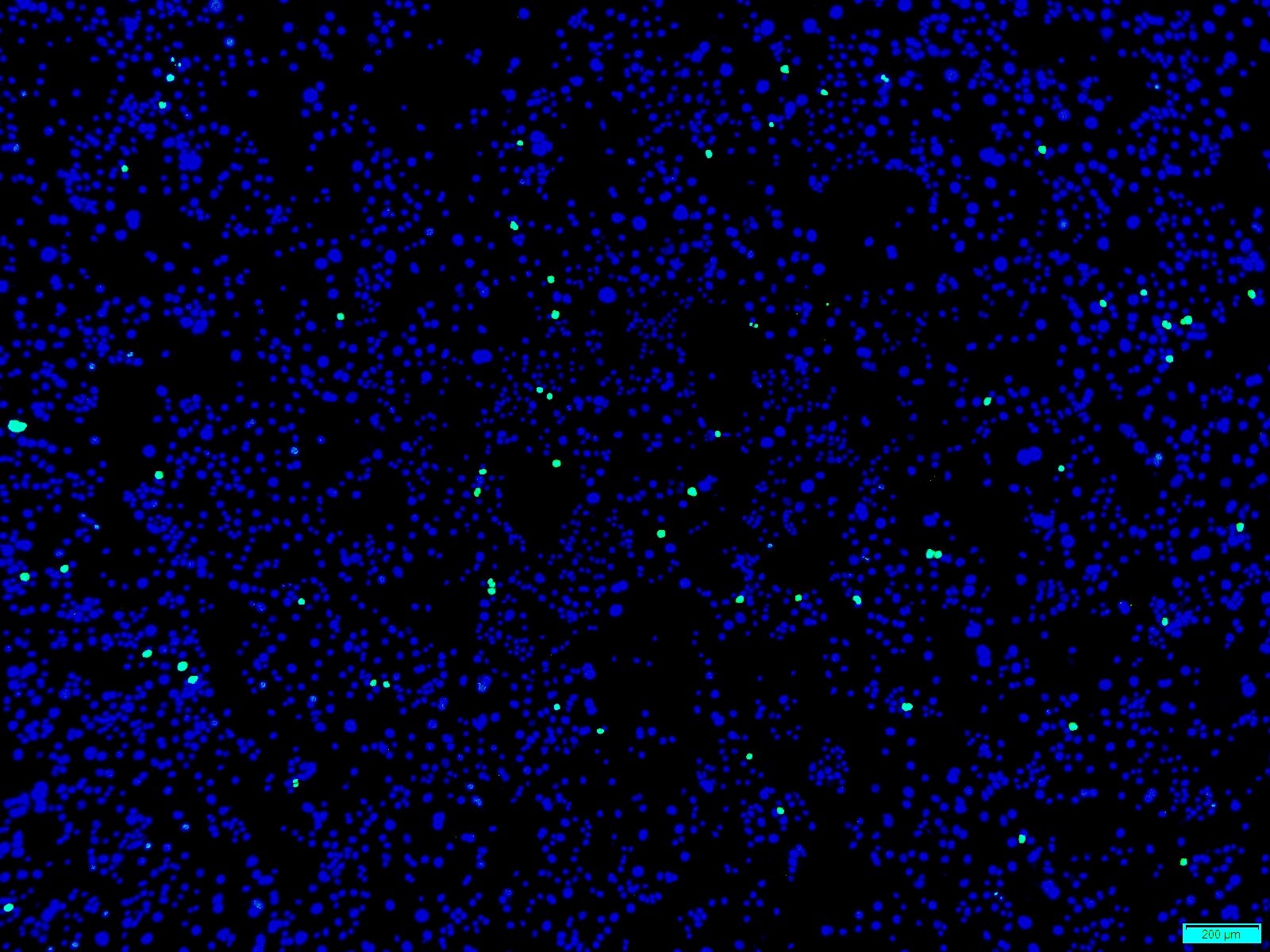

Supplement: Supplementary file 14 — Figure EV1 Source Data [file 44321_2026_414_MOESM14_ESM.zip › Fig. EV1/EV1B/JHOC9 shCtrl.jpg]

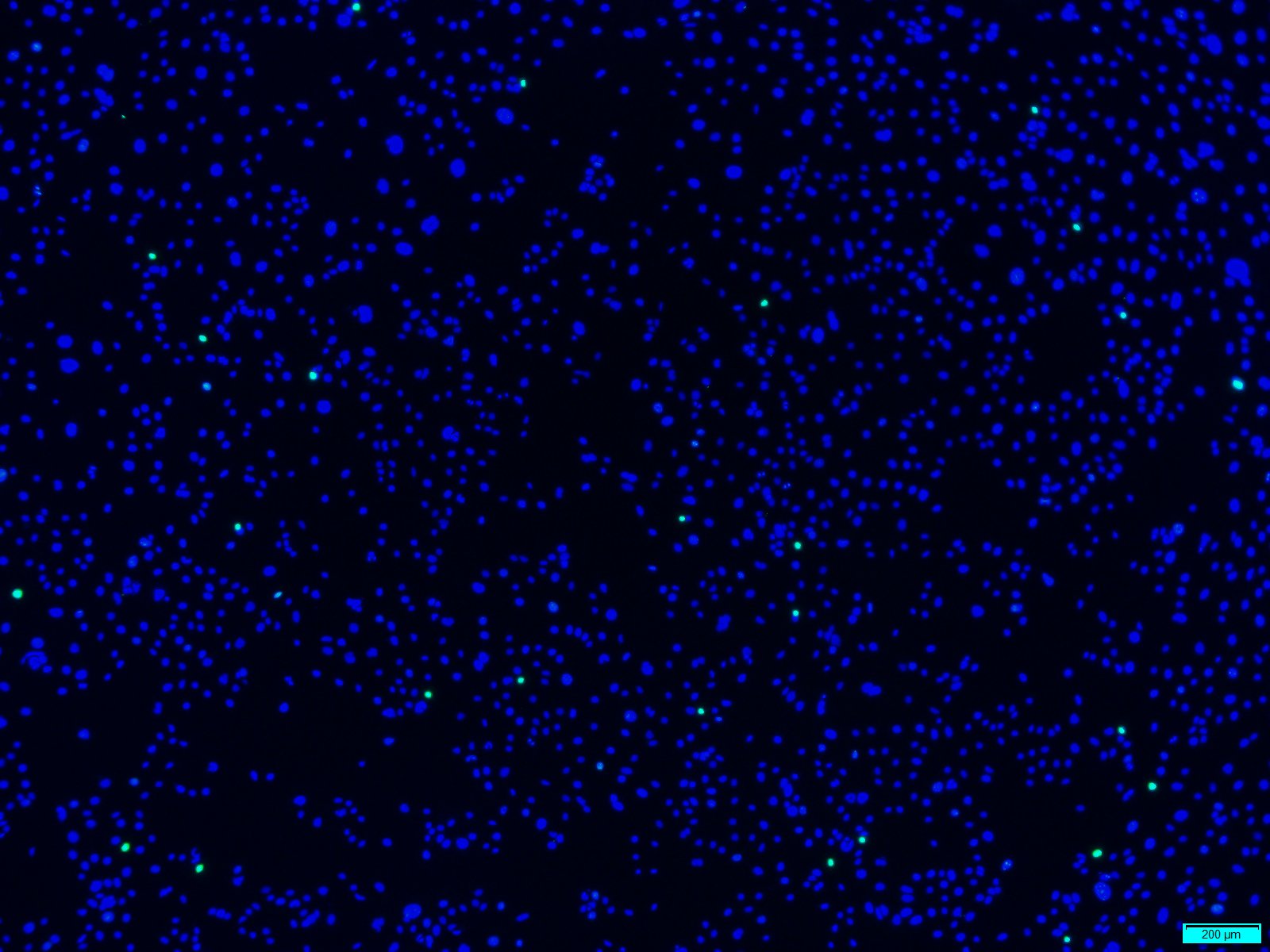

Supplement: Supplementary file 14 — Figure EV1 Source Data [file 44321_2026_414_MOESM14_ESM.zip › Fig. EV1/EV1B/OVCA429 shBMAL2#1.jpg]

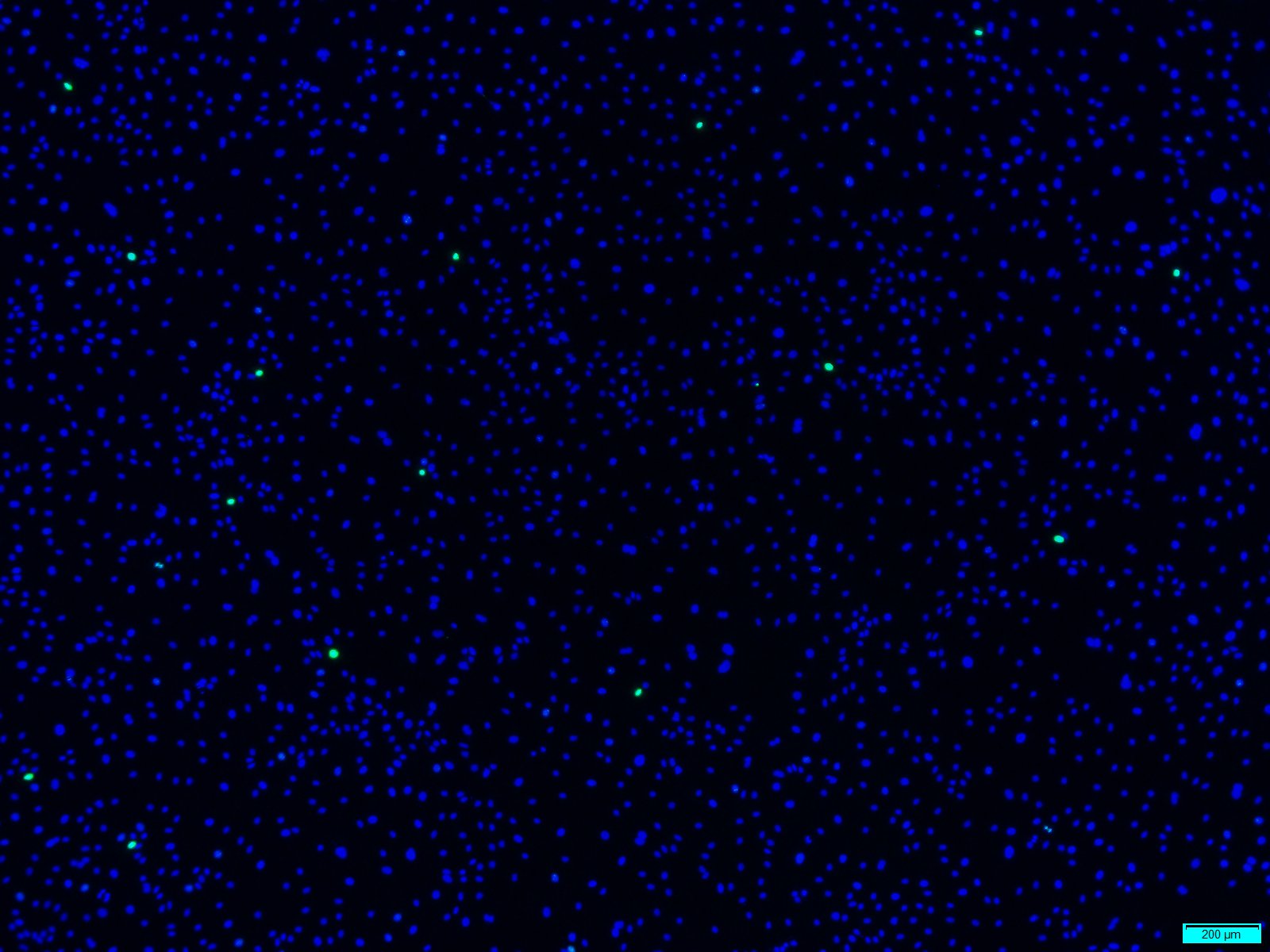

Supplement: Supplementary file 14 — Figure EV1 Source Data [file 44321_2026_414_MOESM14_ESM.zip › Fig. EV1/EV1B/OVCA429 shBMAL2#2.jpg]

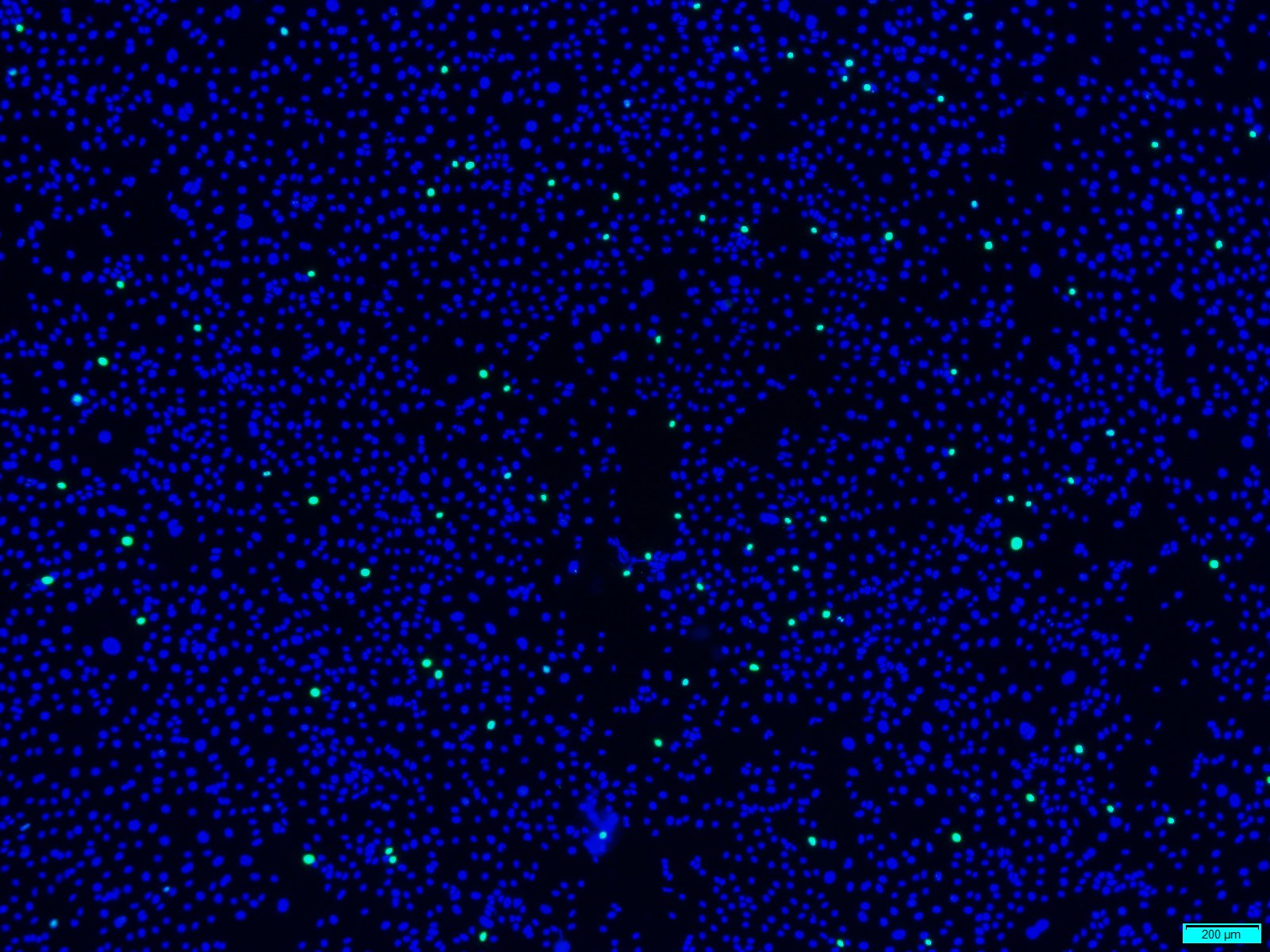

Supplement: Supplementary file 14 — Figure EV1 Source Data [file 44321_2026_414_MOESM14_ESM.zip › Fig. EV1/EV1B/OVCA429 shCtrl.jpg]

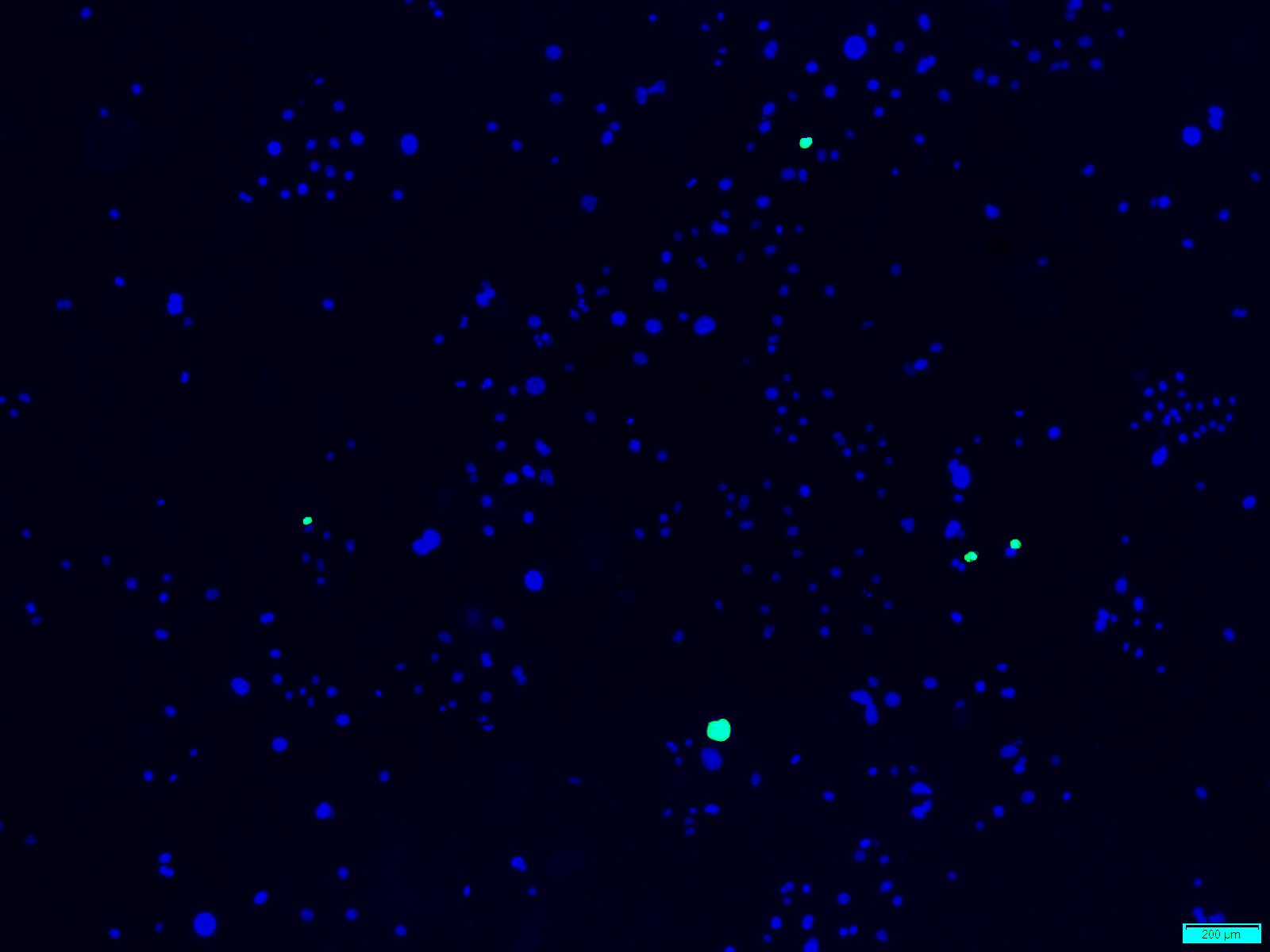

Supplement: Supplementary file 14 — Figure EV1 Source Data [file 44321_2026_414_MOESM14_ESM.zip › Fig. EV1/EV1B/OVISE shBMAL2#1.jpg]

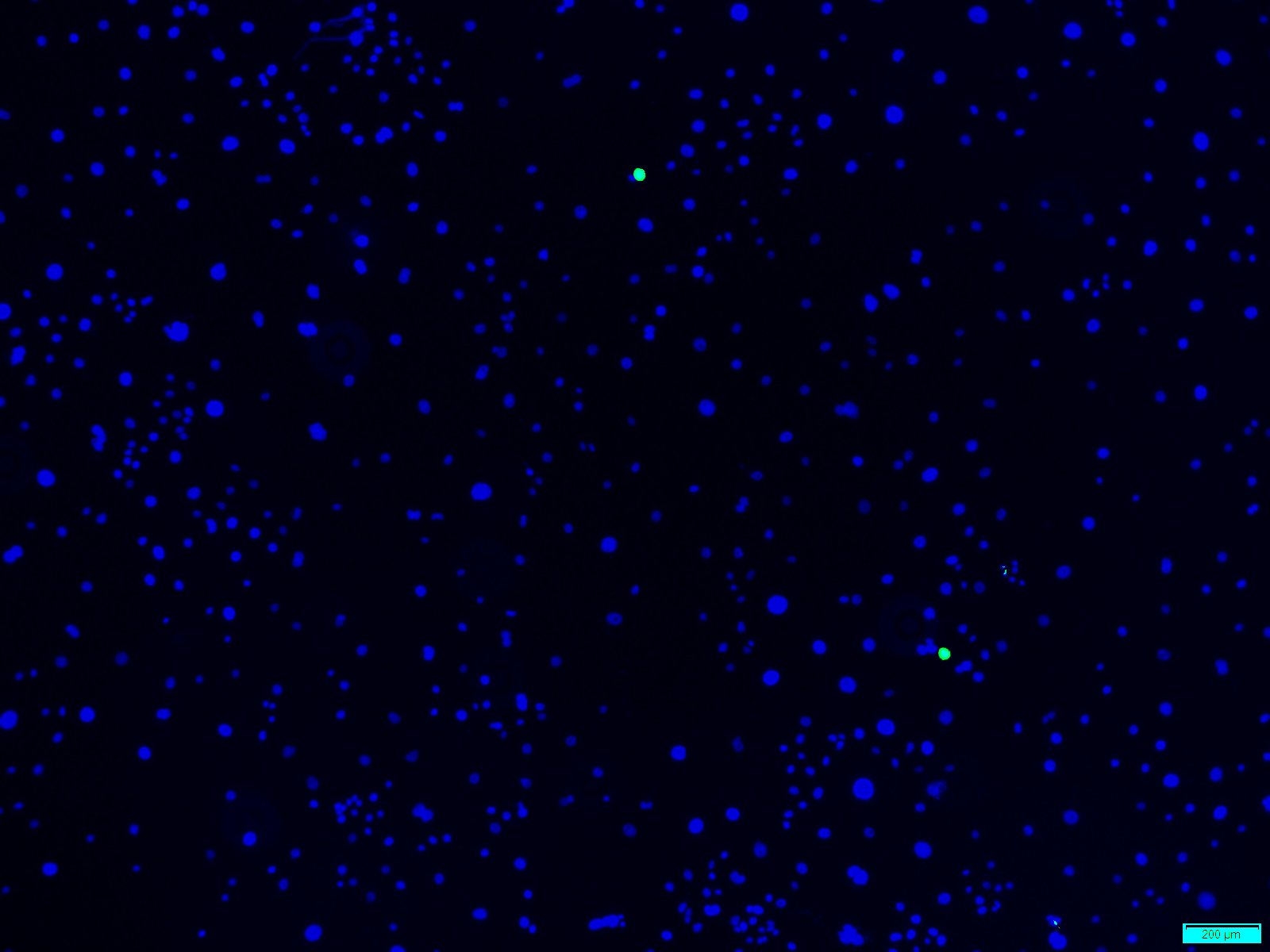

Supplement: Supplementary file 14 — Figure EV1 Source Data [file 44321_2026_414_MOESM14_ESM.zip › Fig. EV1/EV1B/OVISE shBMAL2#2.jpg]

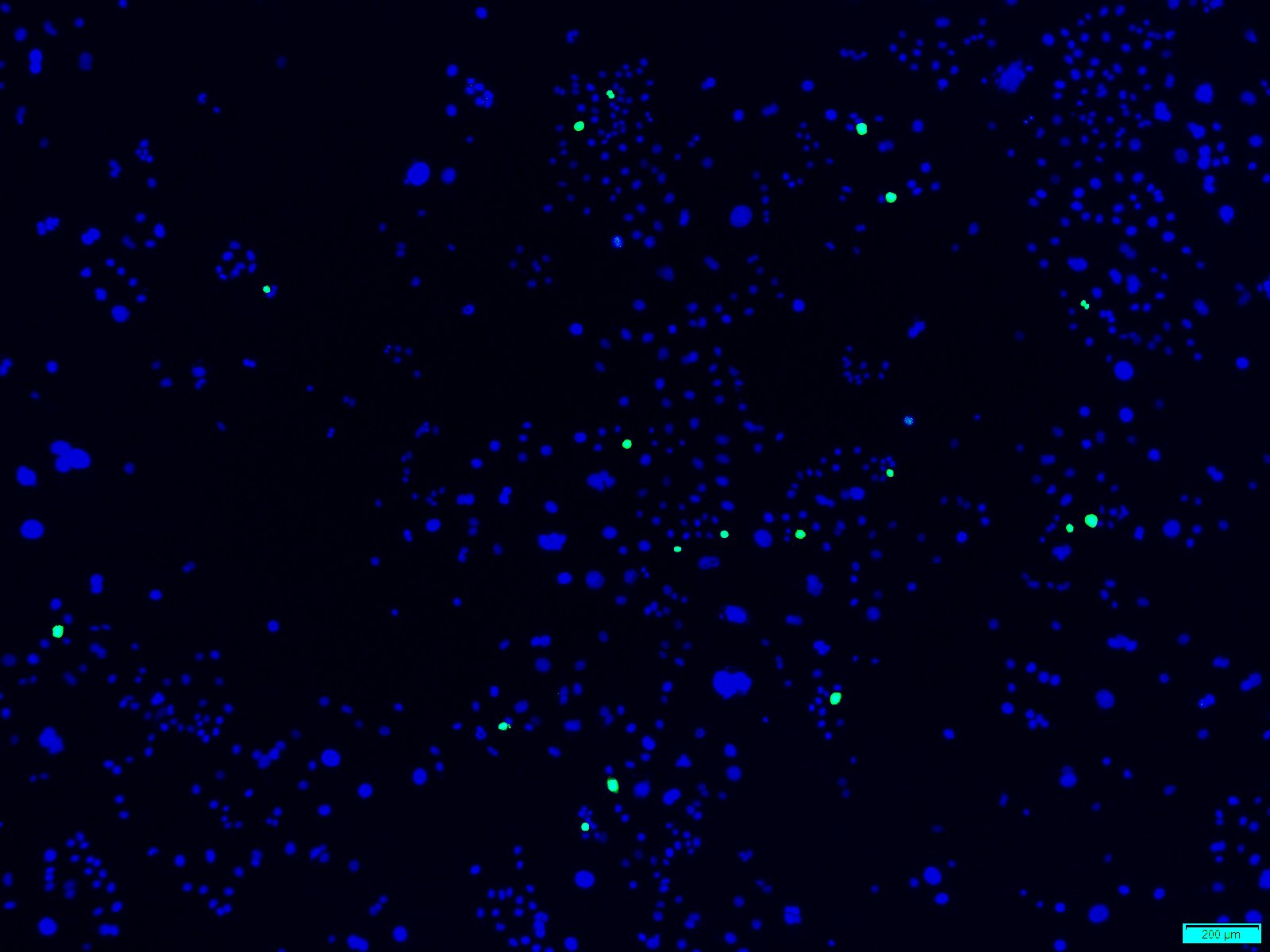

Supplement: Supplementary file 14 — Figure EV1 Source Data [file 44321_2026_414_MOESM14_ESM.zip › Fig. EV1/EV1B/OVISE shCtrl.jpg]

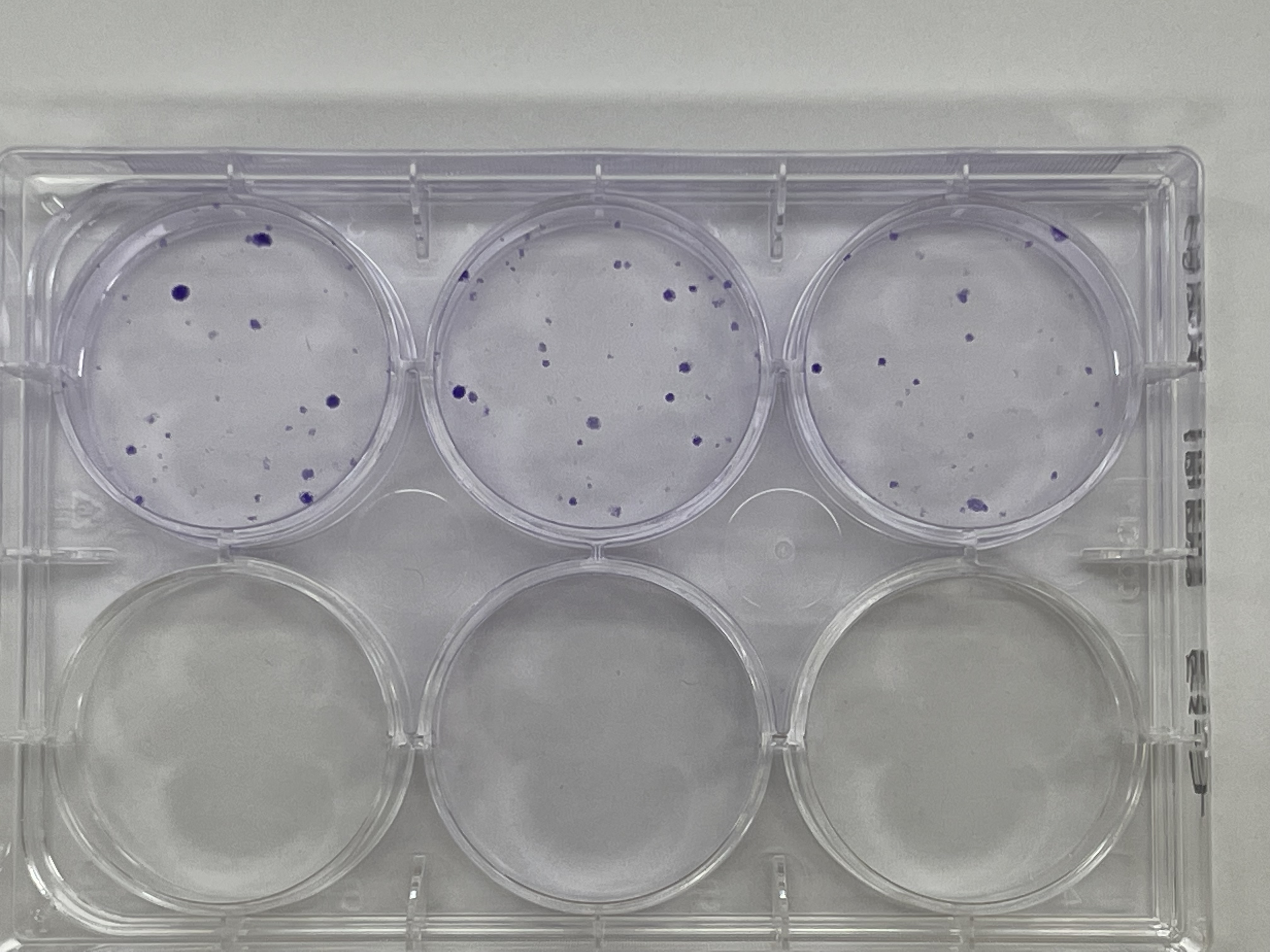

Supplement: Supplementary file 14 — Figure EV1 Source Data [file 44321_2026_414_MOESM14_ESM.zip › Fig. EV1/EV1C/ES-2 shBMAL2#2.png]

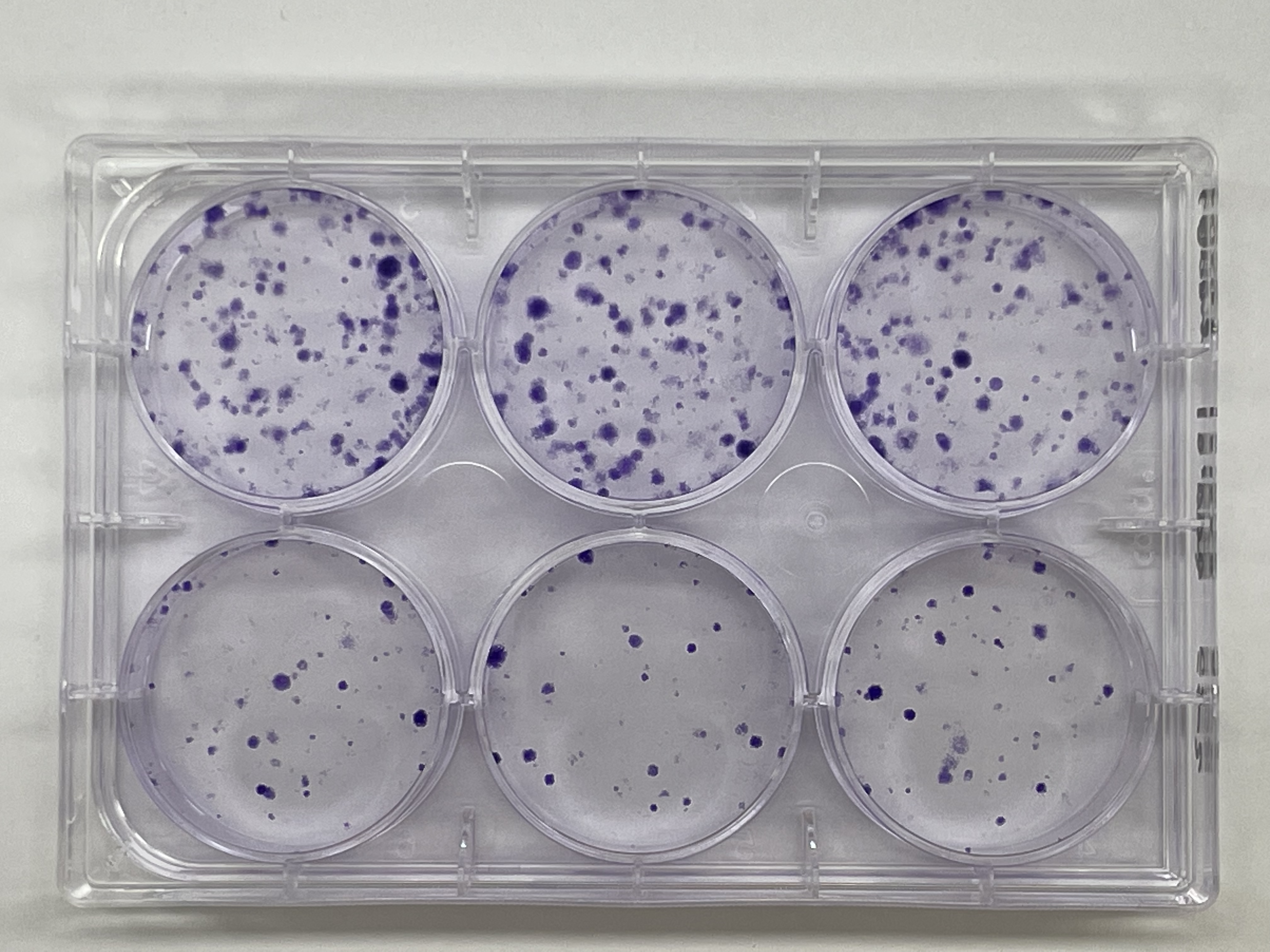

Supplement: Supplementary file 14 — Figure EV1 Source Data [file 44321_2026_414_MOESM14_ESM.zip › Fig. EV1/EV1C/ES-2 shCtrl (Top) shBMAL2#1 (Bottom).png]

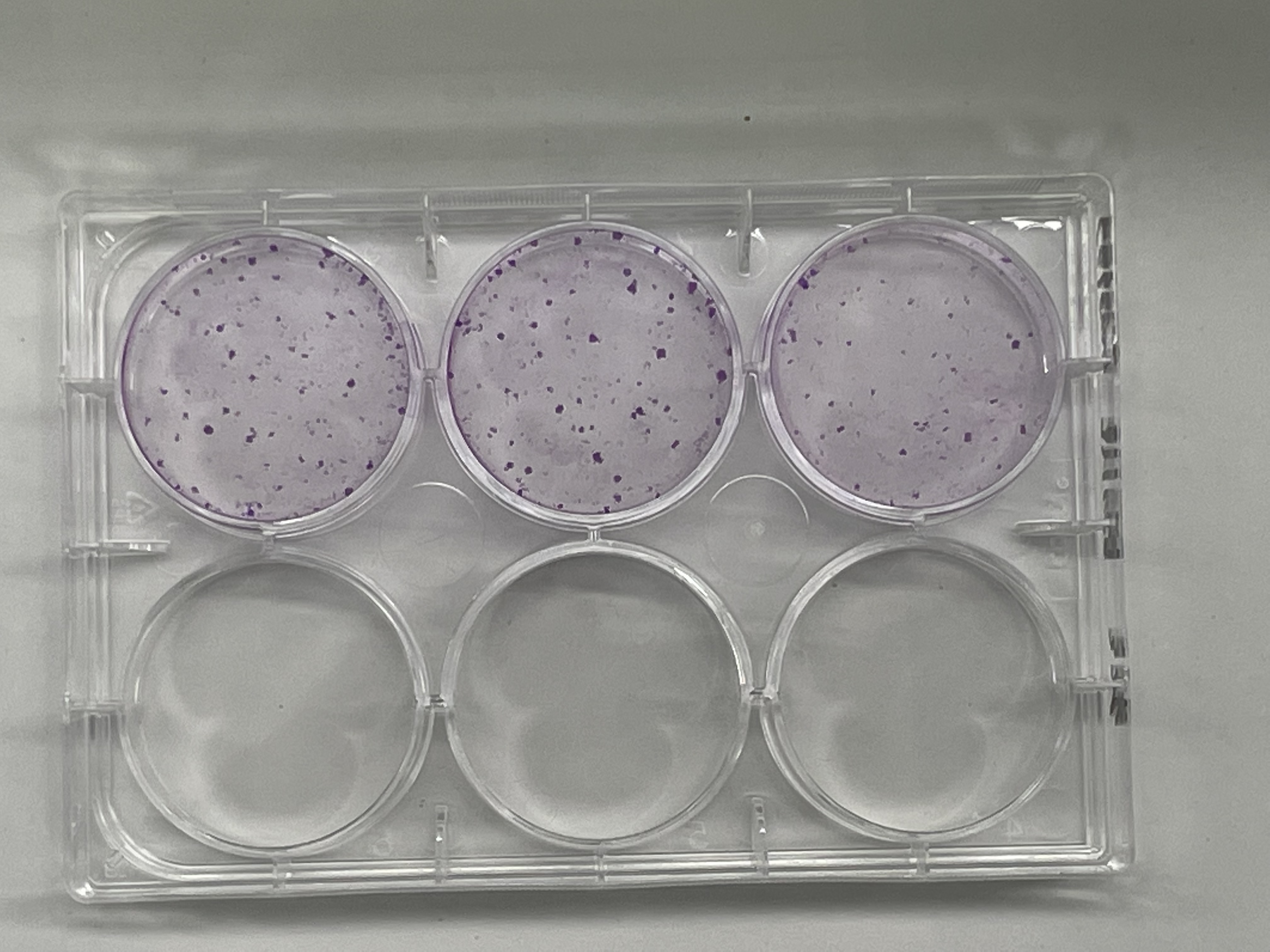

Supplement: Supplementary file 14 — Figure EV1 Source Data [file 44321_2026_414_MOESM14_ESM.zip › Fig. EV1/EV1C/JHOC9 shBMAL2#2.png]

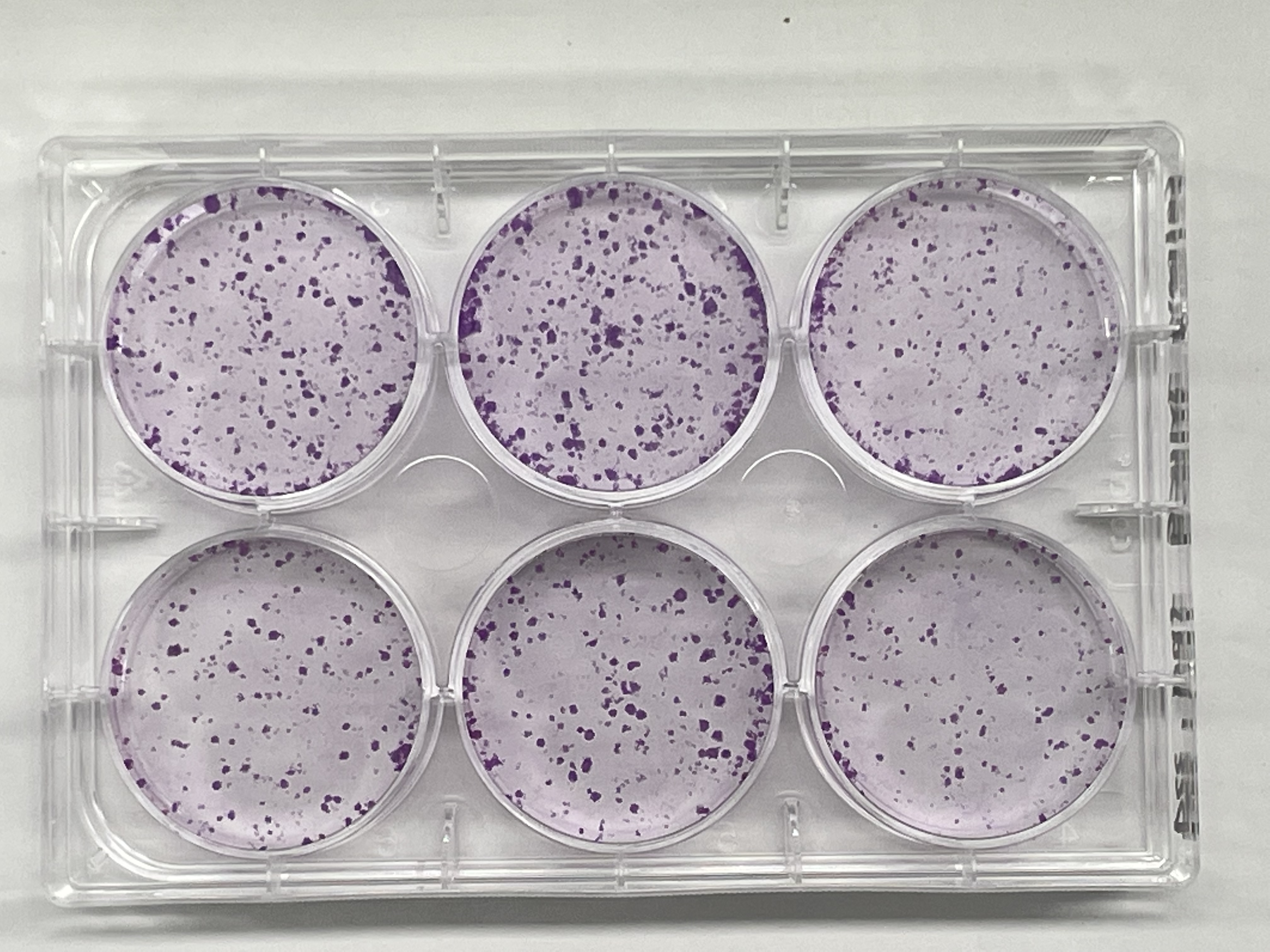

Supplement: Supplementary file 14 — Figure EV1 Source Data [file 44321_2026_414_MOESM14_ESM.zip › Fig. EV1/EV1C/JHOC9 shCtrl (Top) shBMAL2#1 (Bottom).png]

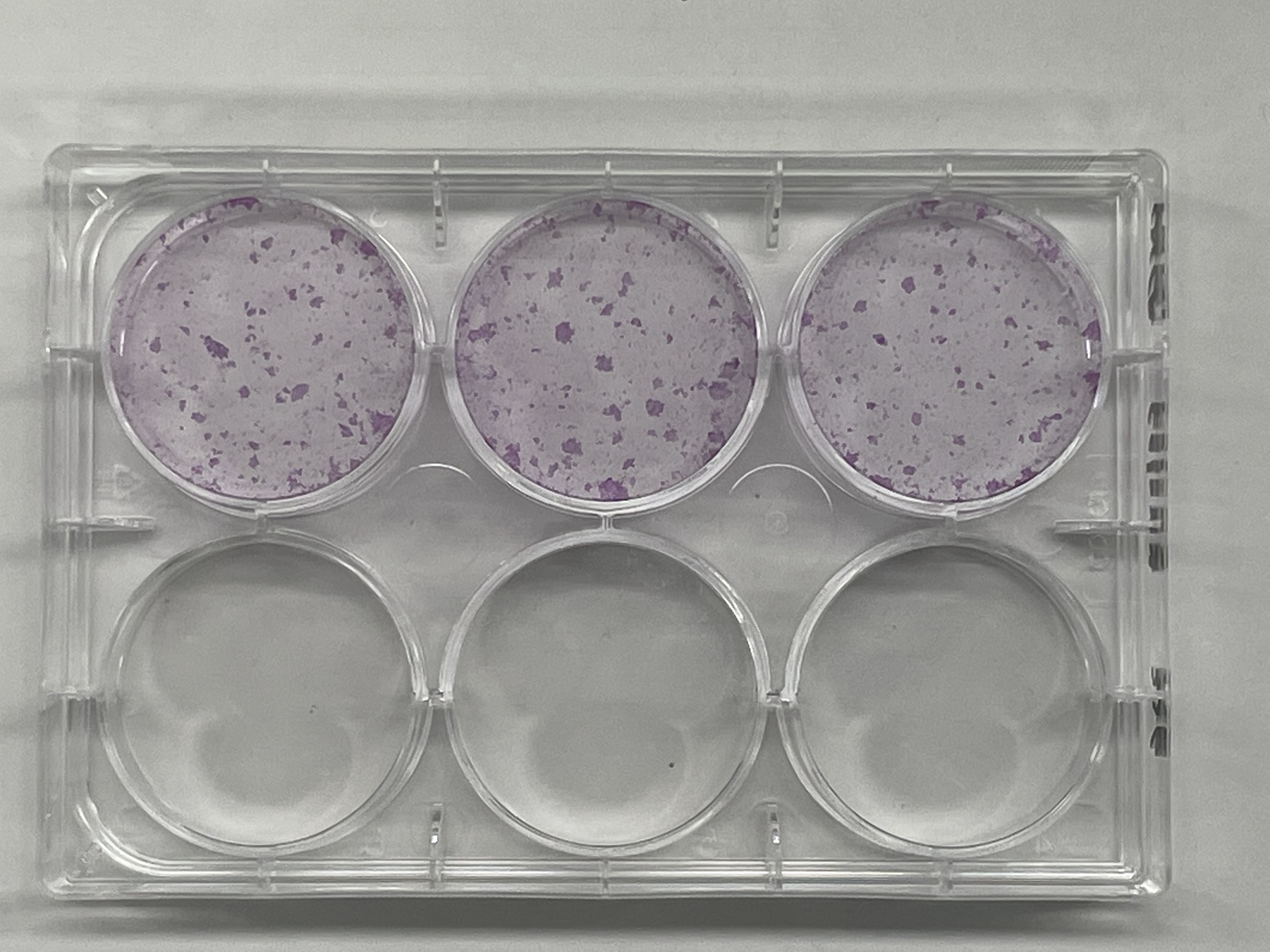

Supplement: Supplementary file 14 — Figure EV1 Source Data [file 44321_2026_414_MOESM14_ESM.zip › Fig. EV1/EV1C/OVCA429 shBMAL2#2.png]

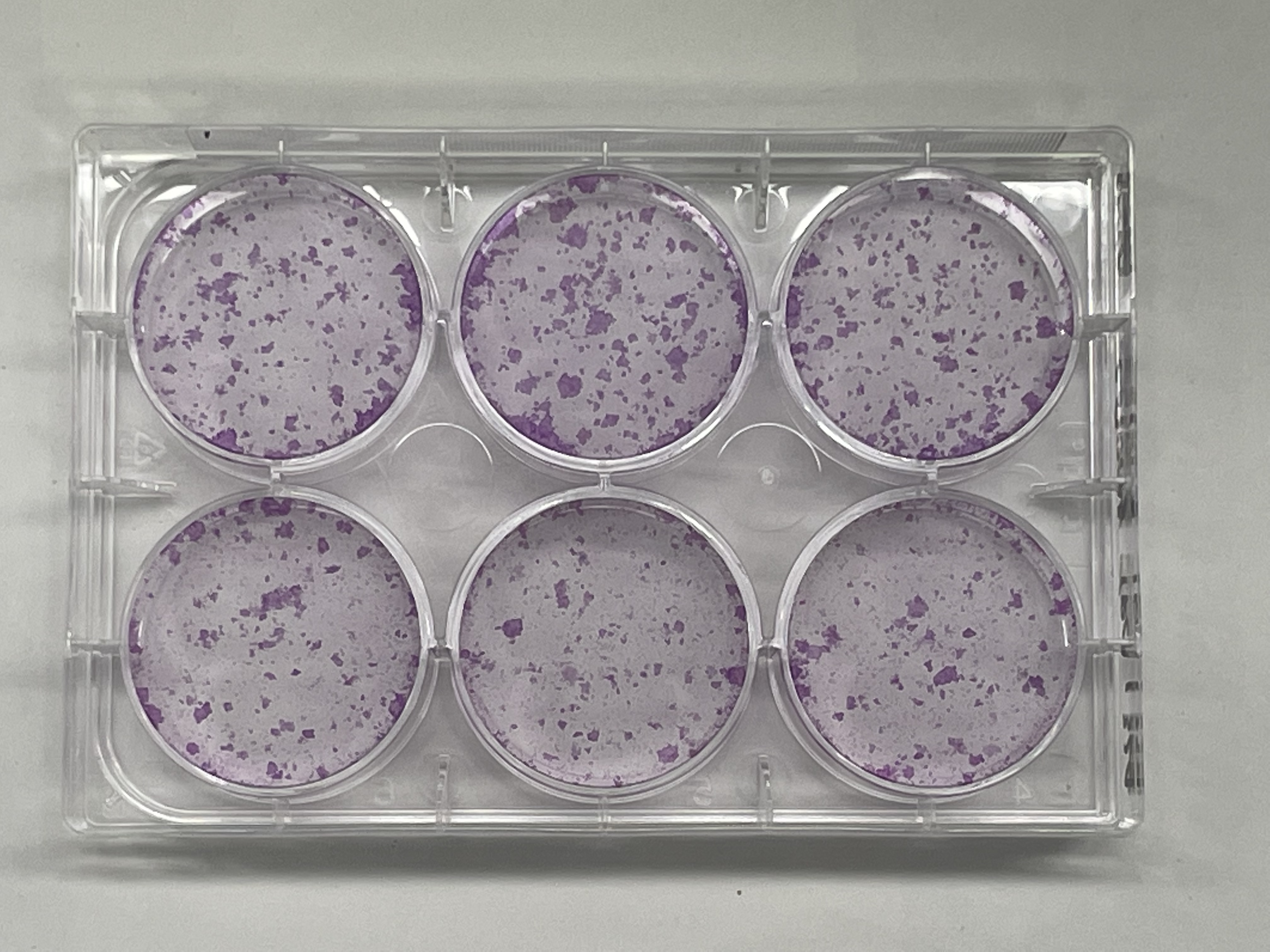

Supplement: Supplementary file 14 — Figure EV1 Source Data [file 44321_2026_414_MOESM14_ESM.zip › Fig. EV1/EV1C/OVCA429 shCtrl (Top) shBMAL2#1 (Bottom).png]

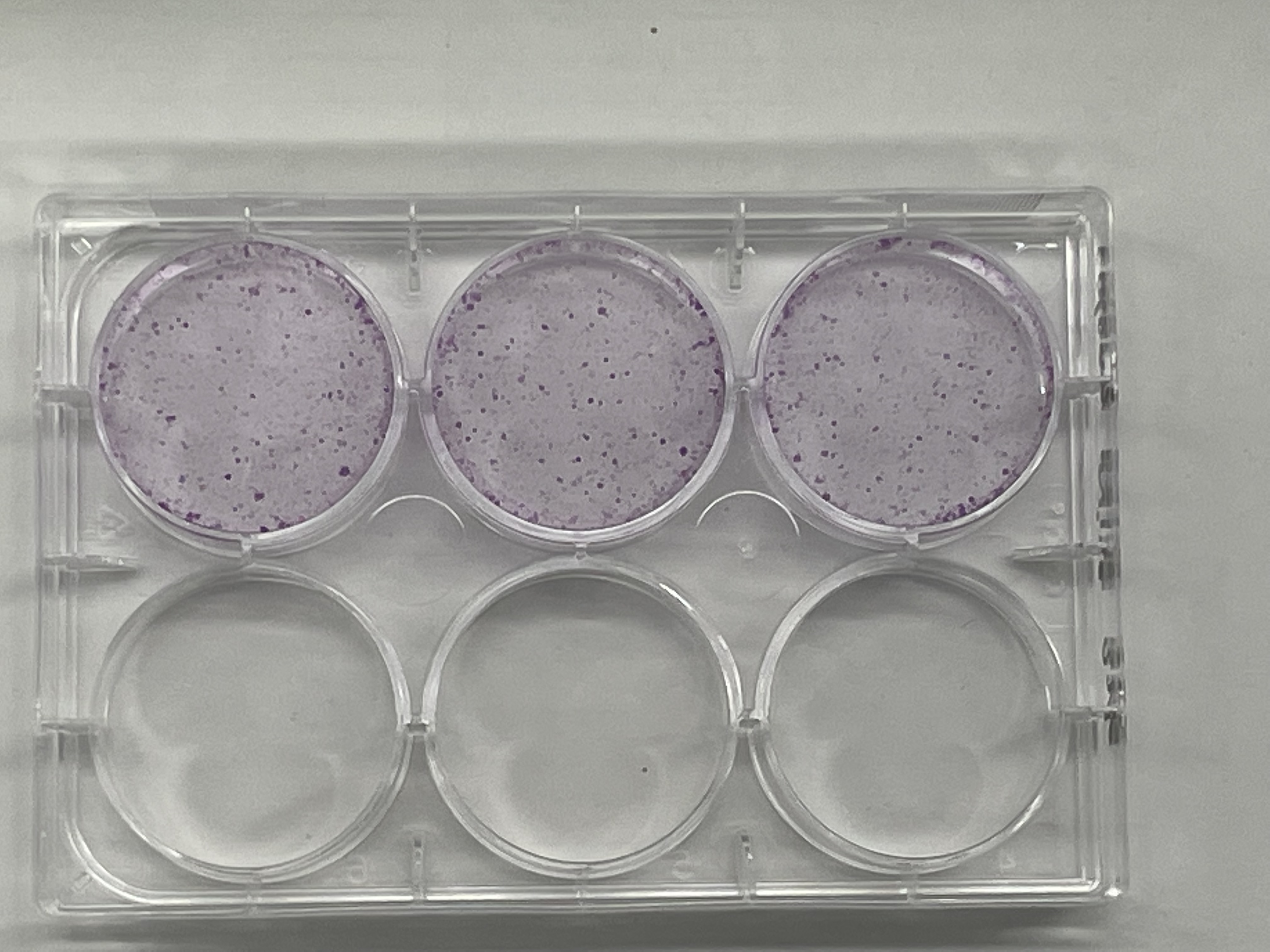

Supplement: Supplementary file 14 — Figure EV1 Source Data [file 44321_2026_414_MOESM14_ESM.zip › Fig. EV1/EV1C/OVISE shBMAL2#2.png]

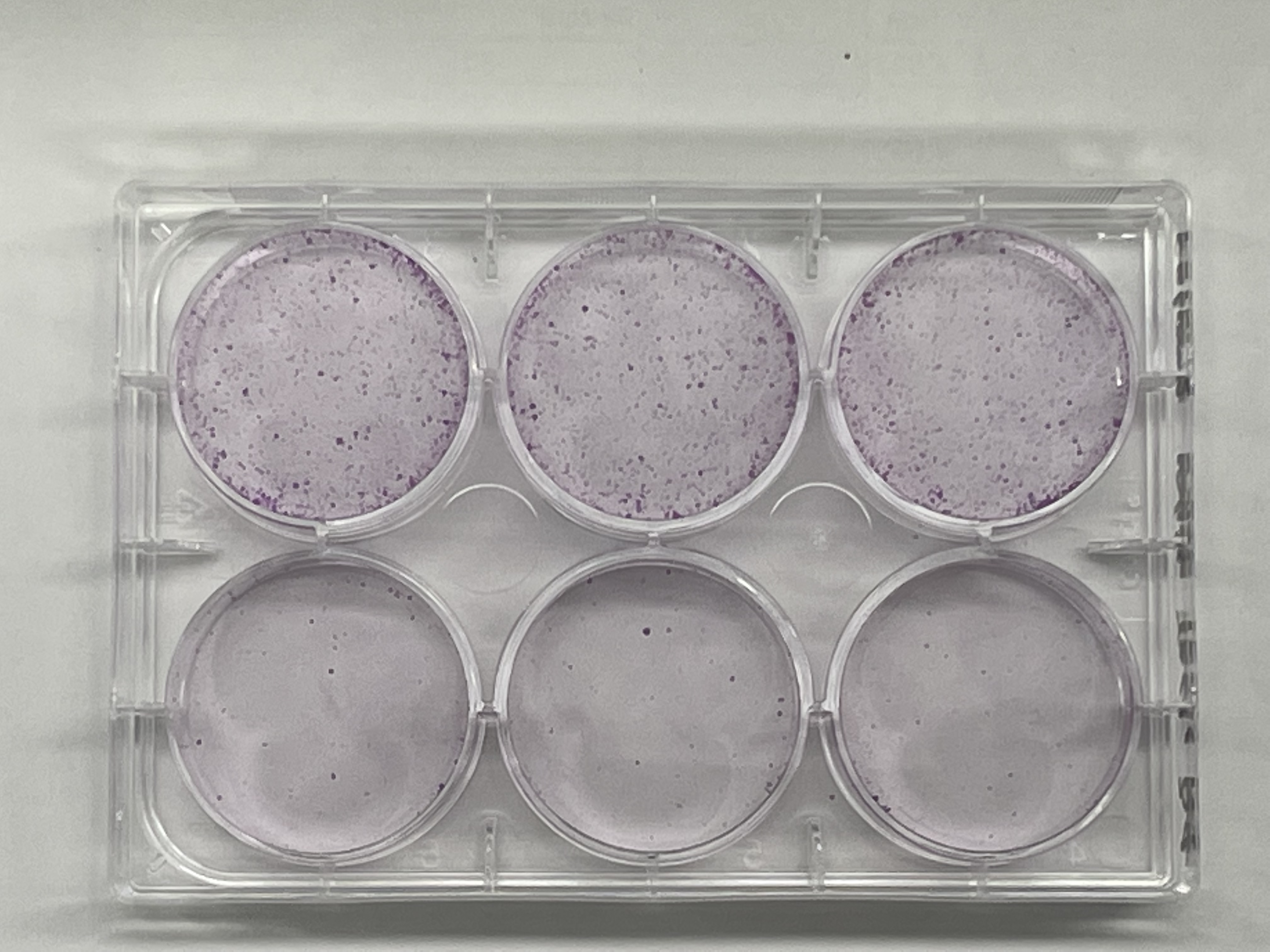

Supplement: Supplementary file 14 — Figure EV1 Source Data [file 44321_2026_414_MOESM14_ESM.zip › Fig. EV1/EV1C/OVISE shCtrl (Top) shBMAL2#1 (Bottom).png]

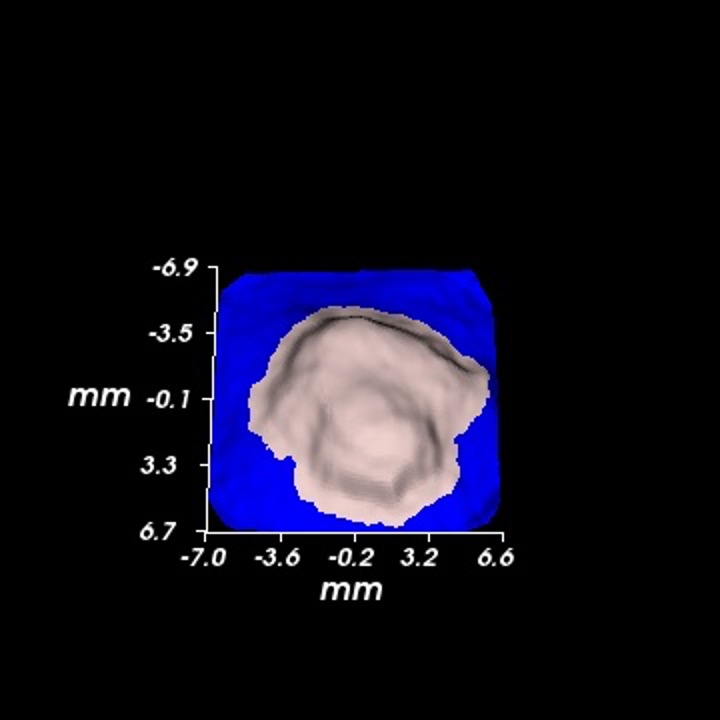

Supplement: Supplementary file 14 — Figure EV1 Source Data [file 44321_2026_414_MOESM14_ESM.zip › Fig. EV1/EV1D/ES-2 shBMAL2 F10.jpg]

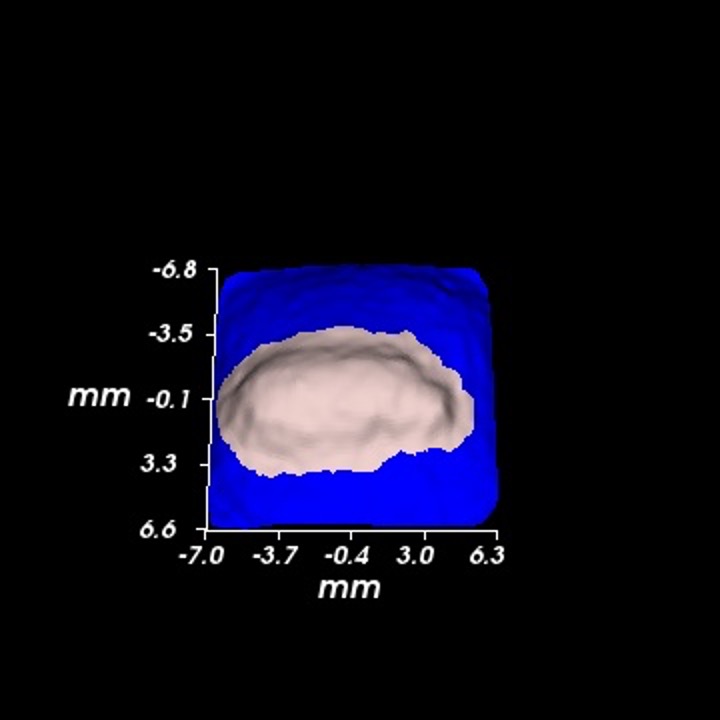

Supplement: Supplementary file 14 — Figure EV1 Source Data [file 44321_2026_414_MOESM14_ESM.zip › Fig. EV1/EV1D/ES-2 shBMAL2 F6.jpg]

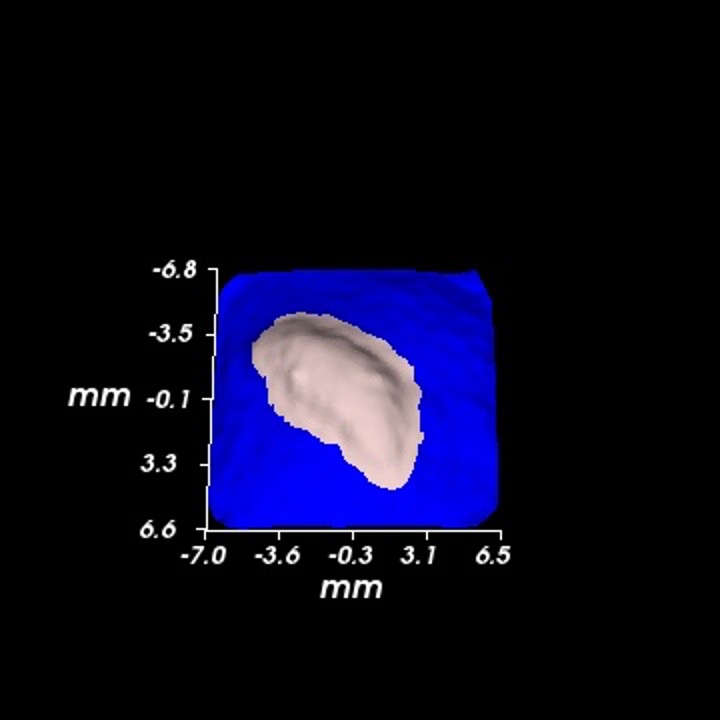

Supplement: Supplementary file 14 — Figure EV1 Source Data [file 44321_2026_414_MOESM14_ESM.zip › Fig. EV1/EV1D/ES-2 shBMAL2 F7.jpg]

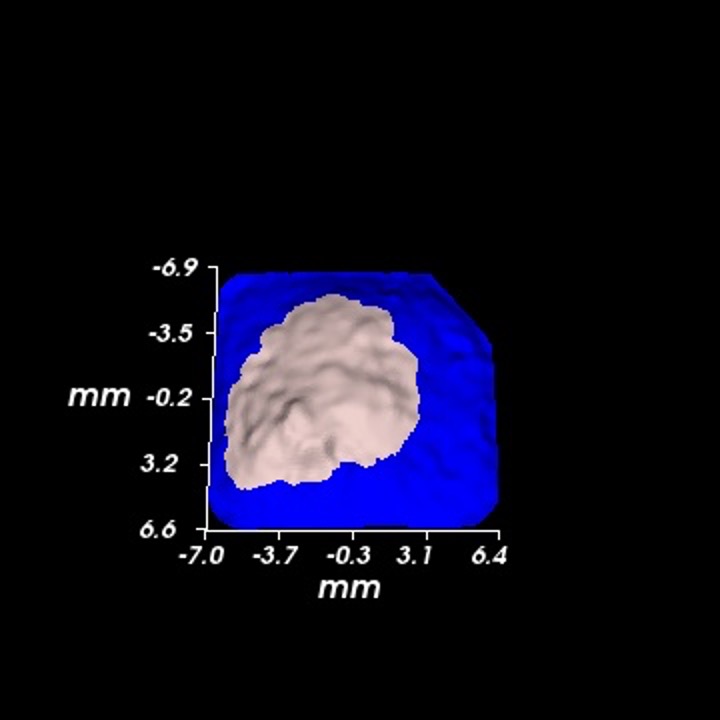

Supplement: Supplementary file 14 — Figure EV1 Source Data [file 44321_2026_414_MOESM14_ESM.zip › Fig. EV1/EV1D/ES-2 shBMAL2 F9.jpg]

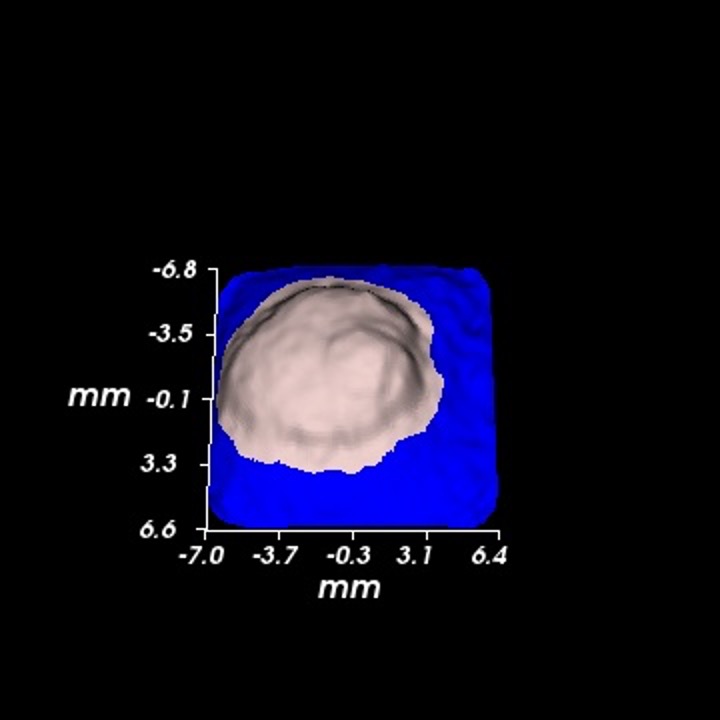

Supplement: Supplementary file 14 — Figure EV1 Source Data [file 44321_2026_414_MOESM14_ESM.zip › Fig. EV1/EV1D/ES-2 shCtrl F1.jpg]

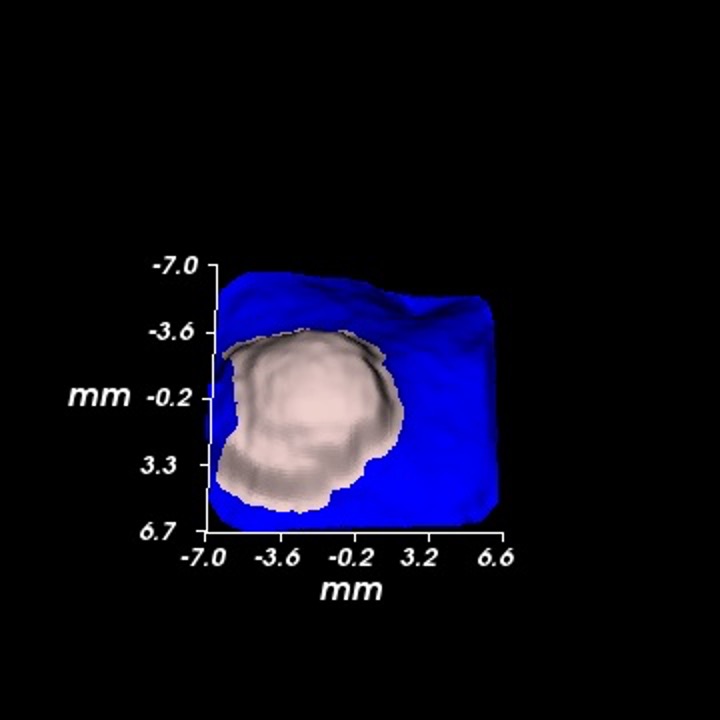

Supplement: Supplementary file 14 — Figure EV1 Source Data [file 44321_2026_414_MOESM14_ESM.zip › Fig. EV1/EV1D/ES-2 shCtrl F2.jpg]

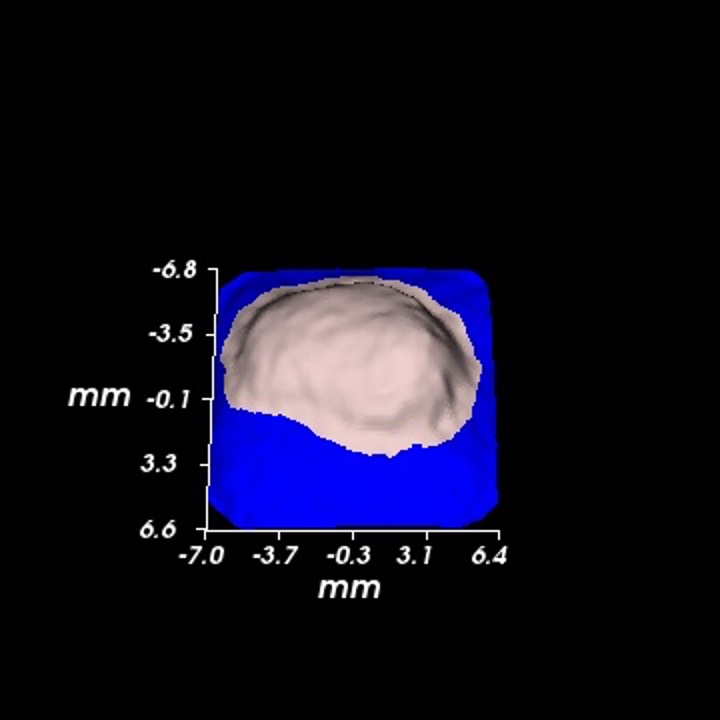

Supplement: Supplementary file 14 — Figure EV1 Source Data [file 44321_2026_414_MOESM14_ESM.zip › Fig. EV1/EV1D/ES-2 shCtrl F3.jpg]

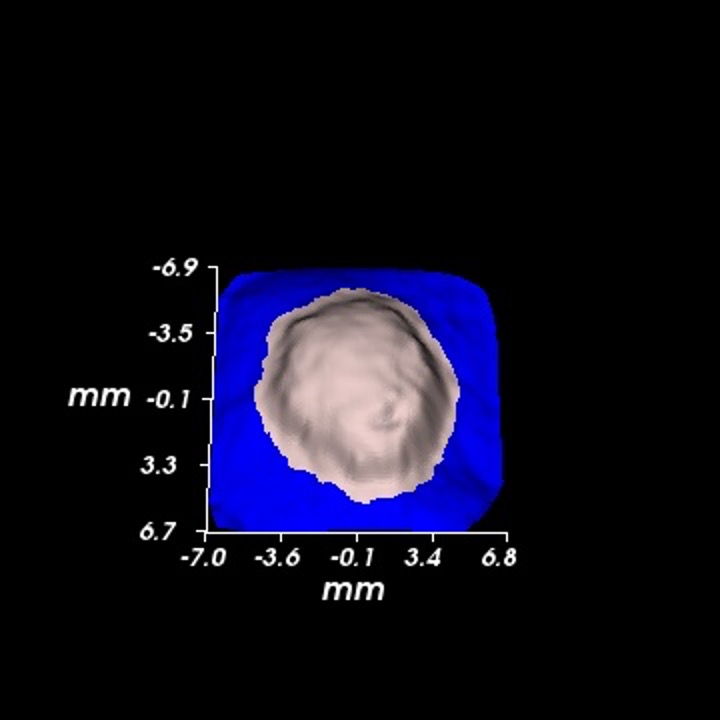

Supplement: Supplementary file 14 — Figure EV1 Source Data [file 44321_2026_414_MOESM14_ESM.zip › Fig. EV1/EV1D/ES-2 shCtrl F4.jpg]

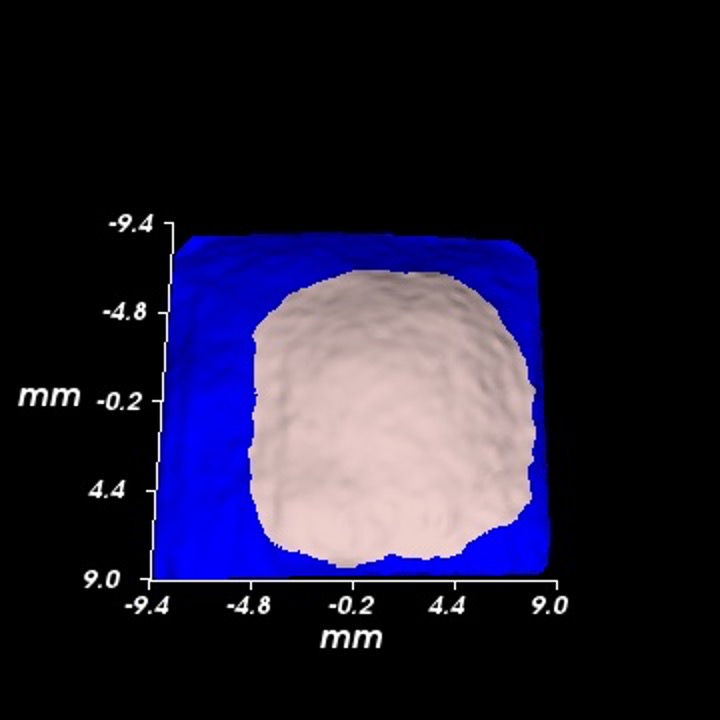

Supplement: Supplementary file 14 — Figure EV1 Source Data [file 44321_2026_414_MOESM14_ESM.zip › Fig. EV1/EV1D/JHOC5 shBMAL2 F10.jpg]

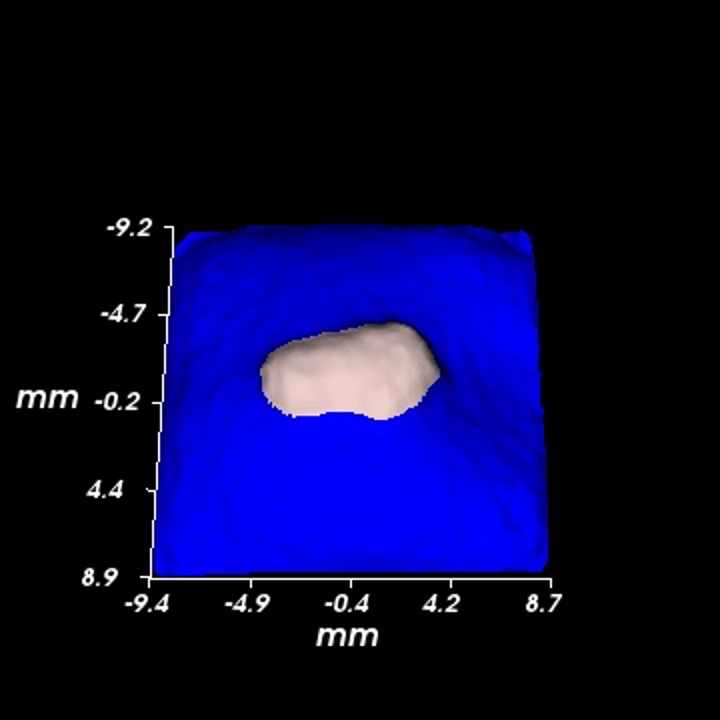

Supplement: Supplementary file 14 — Figure EV1 Source Data [file 44321_2026_414_MOESM14_ESM.zip › Fig. EV1/EV1D/JHOC5 shBMAL2 F6.jpg]

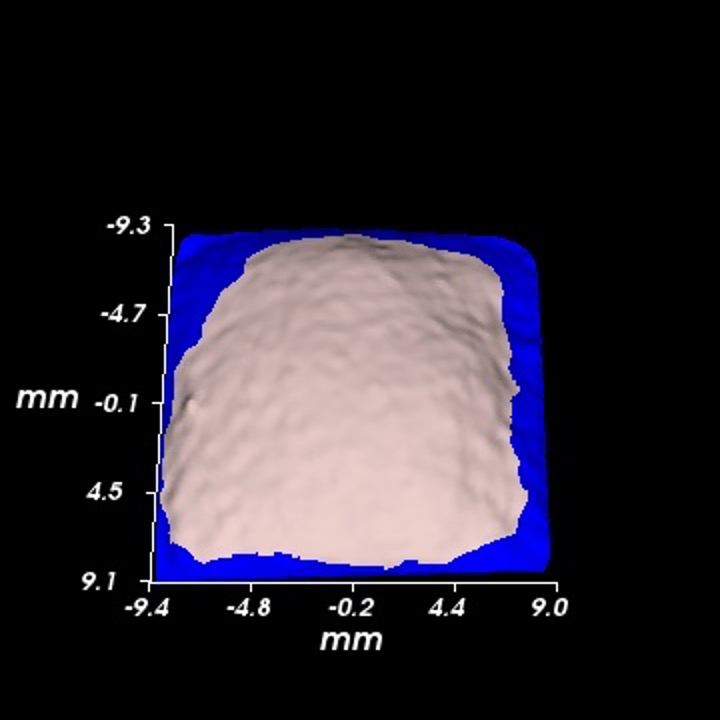

Supplement: Supplementary file 14 — Figure EV1 Source Data [file 44321_2026_414_MOESM14_ESM.zip › Fig. EV1/EV1D/JHOC5 shBMAL2 F8.jpg]

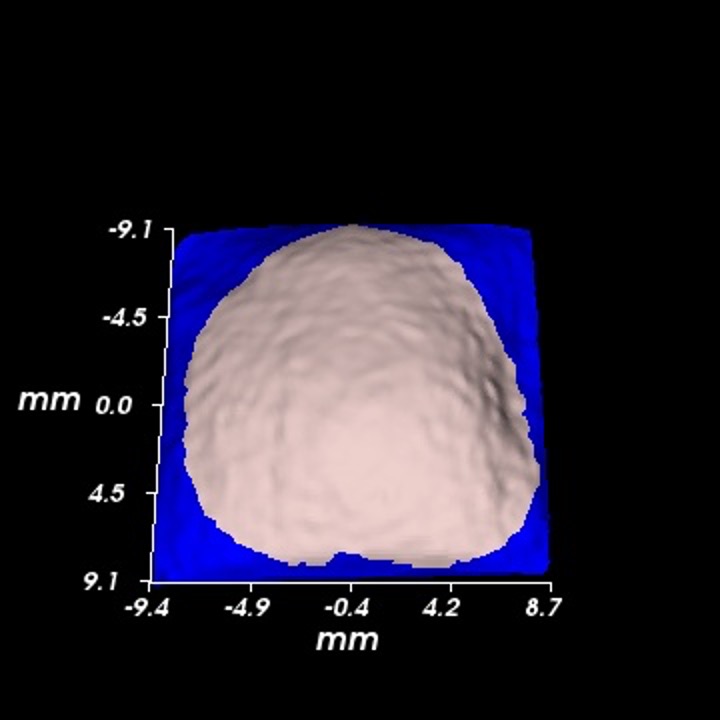

Supplement: Supplementary file 14 — Figure EV1 Source Data [file 44321_2026_414_MOESM14_ESM.zip › Fig. EV1/EV1D/JHOC5 shBMAL2 F9.jpg]

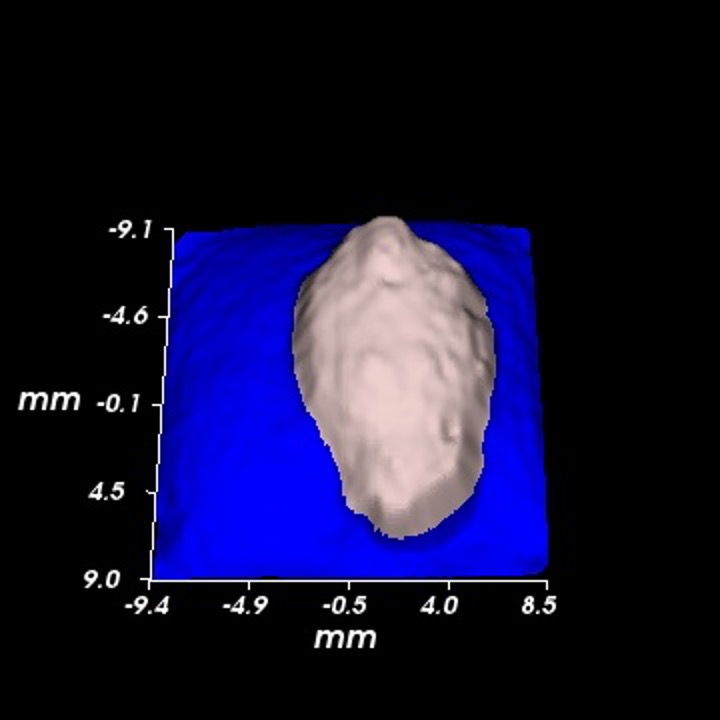

Supplement: Supplementary file 14 — Figure EV1 Source Data [file 44321_2026_414_MOESM14_ESM.zip › Fig. EV1/EV1D/JHOC5 shCtrl F1.jpg]

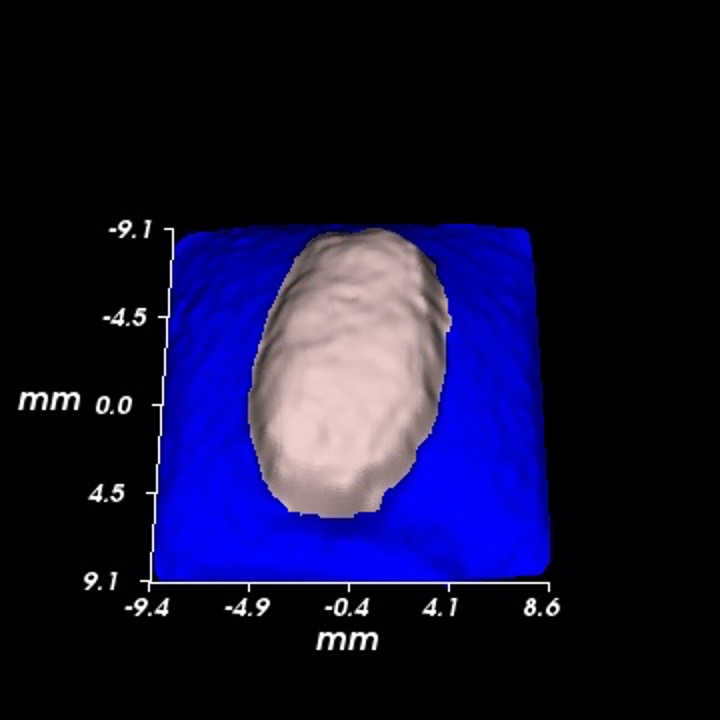

Supplement: Supplementary file 14 — Figure EV1 Source Data [file 44321_2026_414_MOESM14_ESM.zip › Fig. EV1/EV1D/JHOC5 shCtrl F3.jpg]

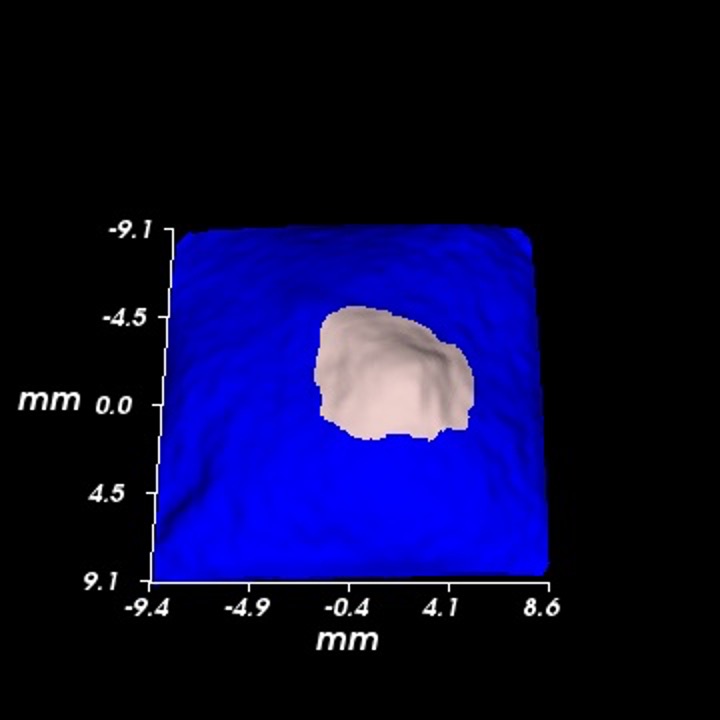

Supplement: Supplementary file 14 — Figure EV1 Source Data [file 44321_2026_414_MOESM14_ESM.zip › Fig. EV1/EV1D/JHOC5 shCtrl F4.jpg]

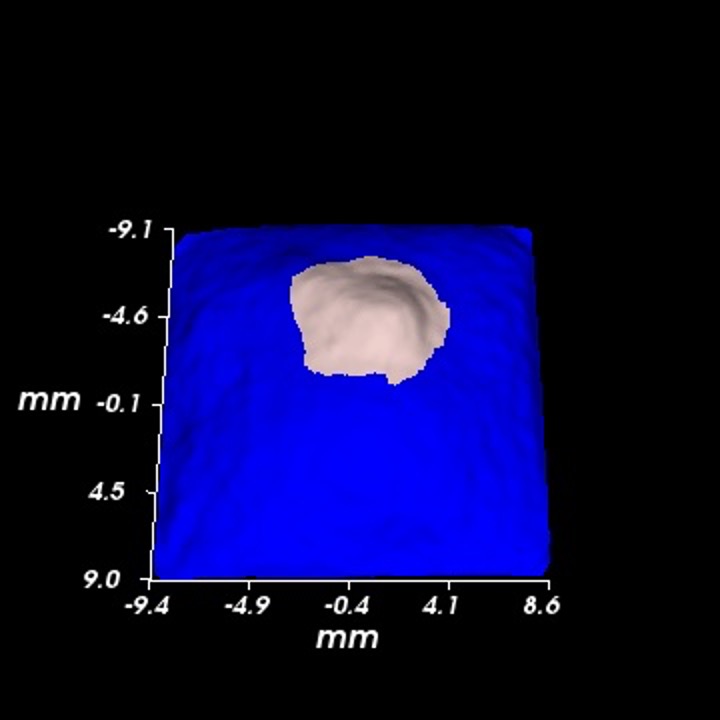

Supplement: Supplementary file 14 — Figure EV1 Source Data [file 44321_2026_414_MOESM14_ESM.zip › Fig. EV1/EV1D/JHOC5 shCtrl F5.jpg]

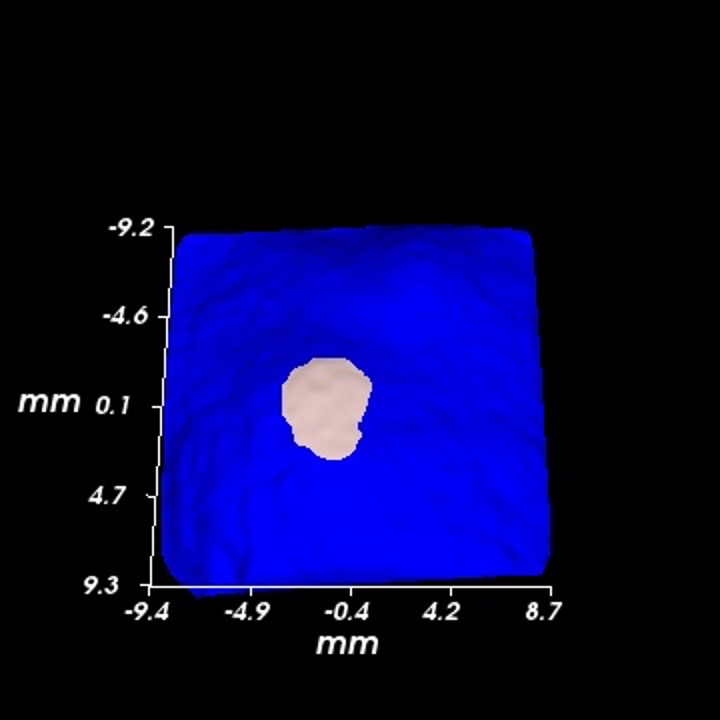

Supplement: Supplementary file 14 — Figure EV1 Source Data [file 44321_2026_414_MOESM14_ESM.zip › Fig. EV1/EV1D/OVISE shBMAL2 F10.jpg]

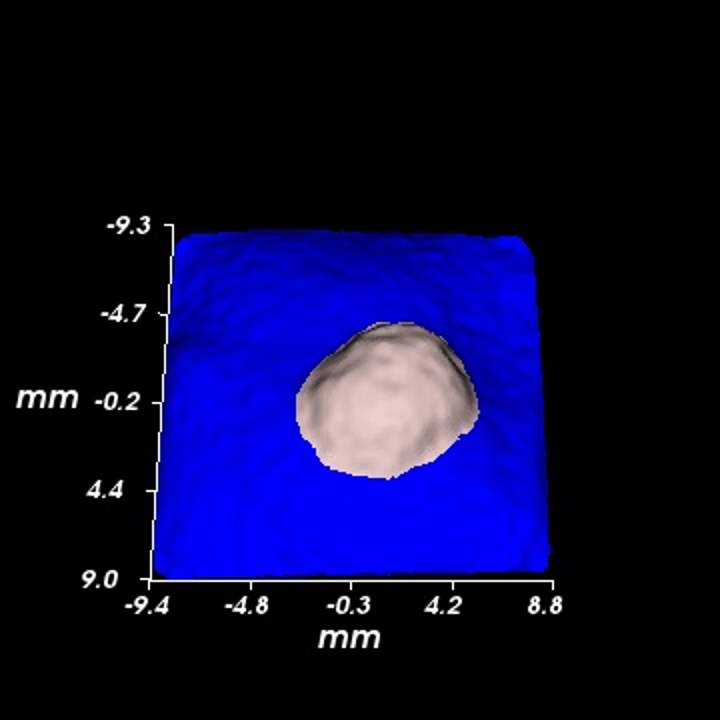

Supplement: Supplementary file 14 — Figure EV1 Source Data [file 44321_2026_414_MOESM14_ESM.zip › Fig. EV1/EV1D/OVISE shBMAL2 F6.jpg]

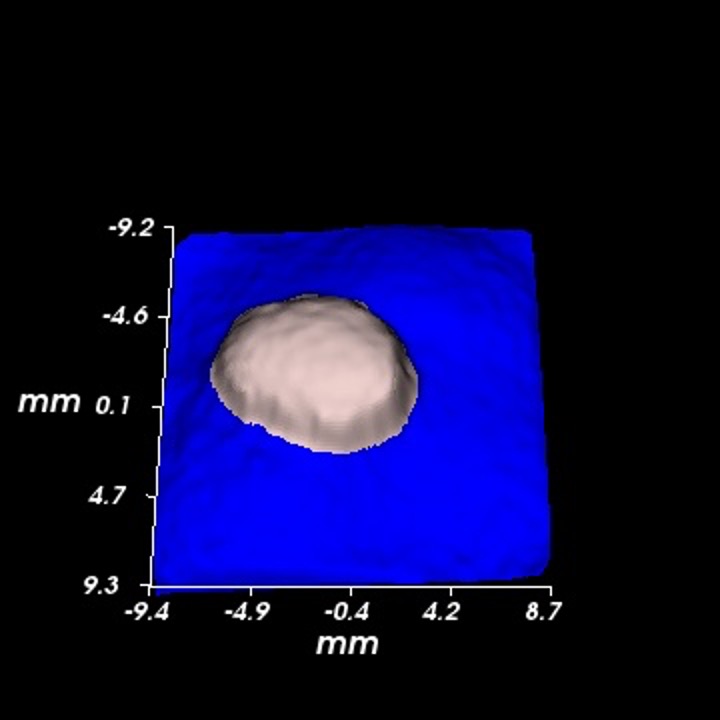

Supplement: Supplementary file 14 — Figure EV1 Source Data [file 44321_2026_414_MOESM14_ESM.zip › Fig. EV1/EV1D/OVISE shBMAL2 F8.jpg]

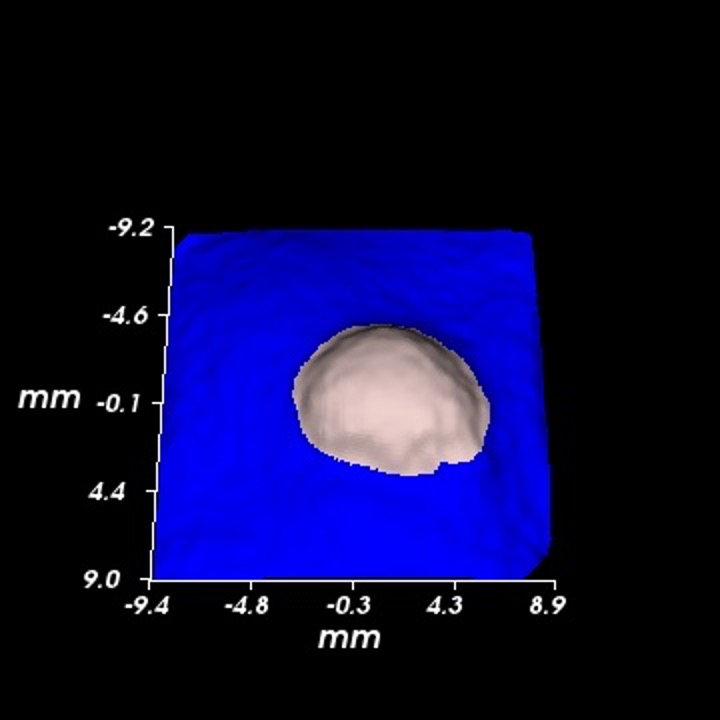

Supplement: Supplementary file 14 — Figure EV1 Source Data [file 44321_2026_414_MOESM14_ESM.zip › Fig. EV1/EV1D/OVISE shBMAL2 F9.jpg]

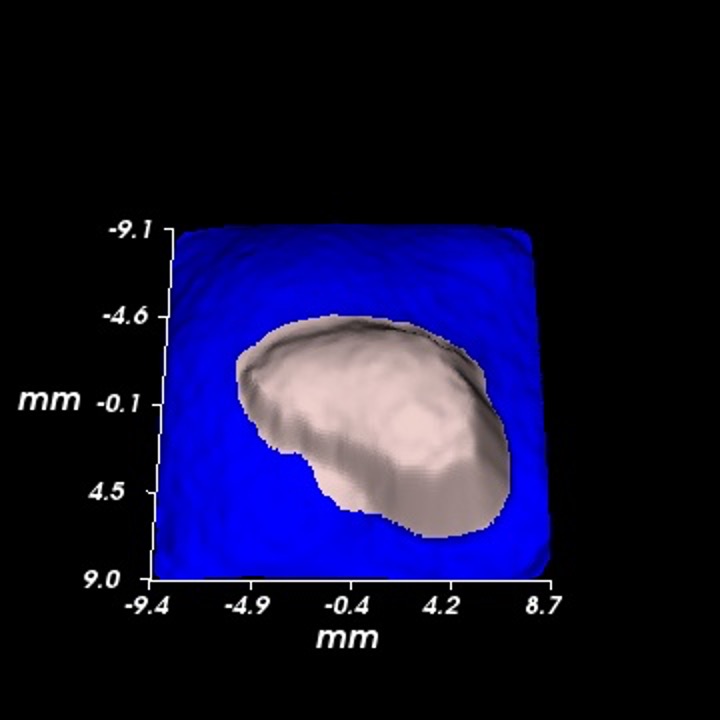

Supplement: Supplementary file 14 — Figure EV1 Source Data [file 44321_2026_414_MOESM14_ESM.zip › Fig. EV1/EV1D/OVISE shCtrl F1.jpg]

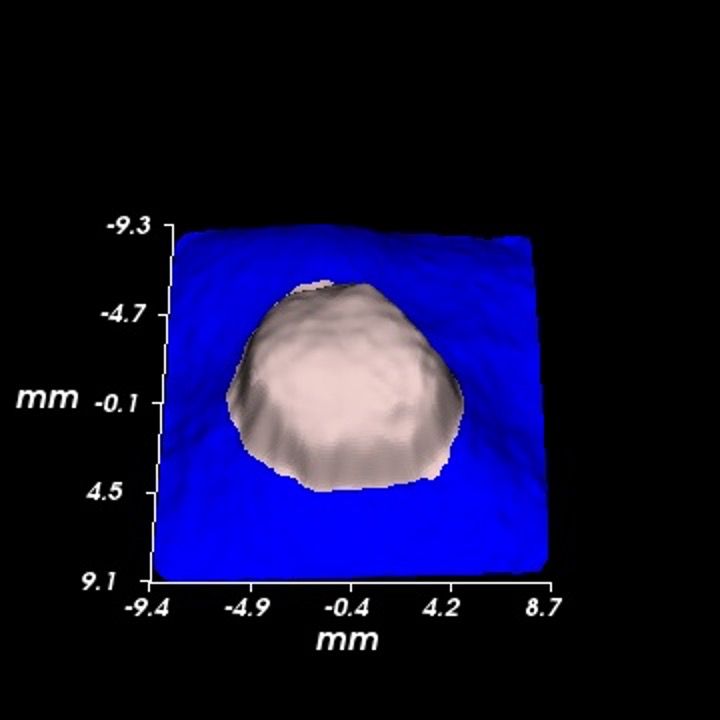

Supplement: Supplementary file 14 — Figure EV1 Source Data [file 44321_2026_414_MOESM14_ESM.zip › Fig. EV1/EV1D/OVISE shCtrl F2.jpg]

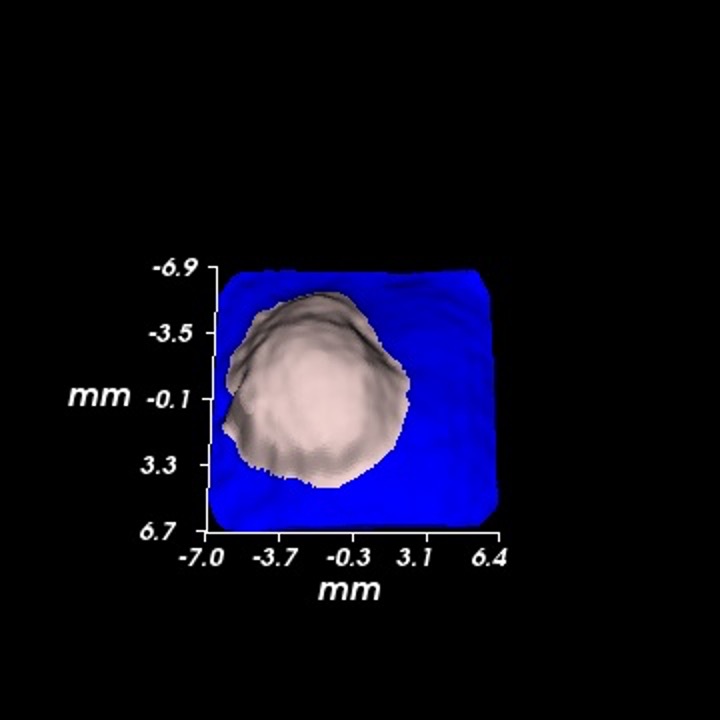

Supplement: Supplementary file 14 — Figure EV1 Source Data [file 44321_2026_414_MOESM14_ESM.zip › Fig. EV1/EV1D/OVISE shCtrl F3.jpg]

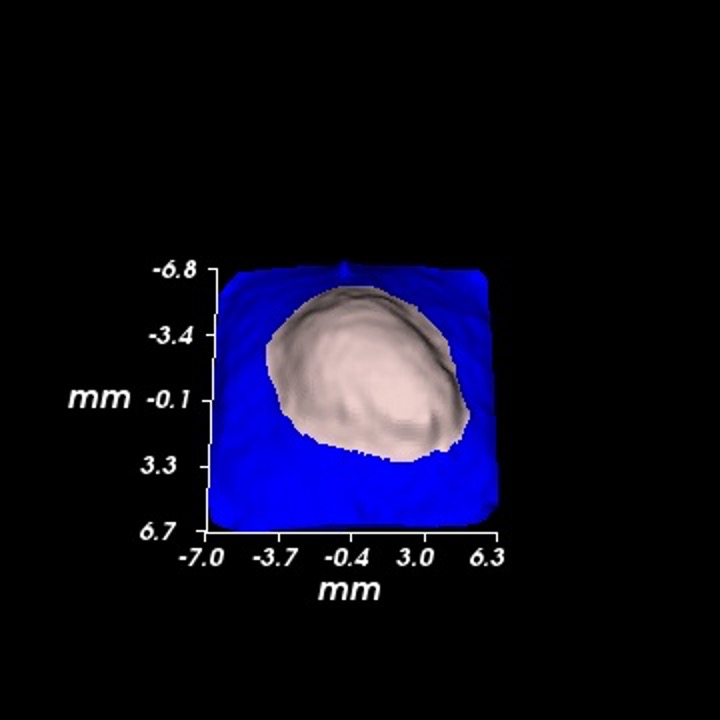

Supplement: Supplementary file 14 — Figure EV1 Source Data [file 44321_2026_414_MOESM14_ESM.zip › Fig. EV1/EV1D/OVISE shCtrl F4.jpg]

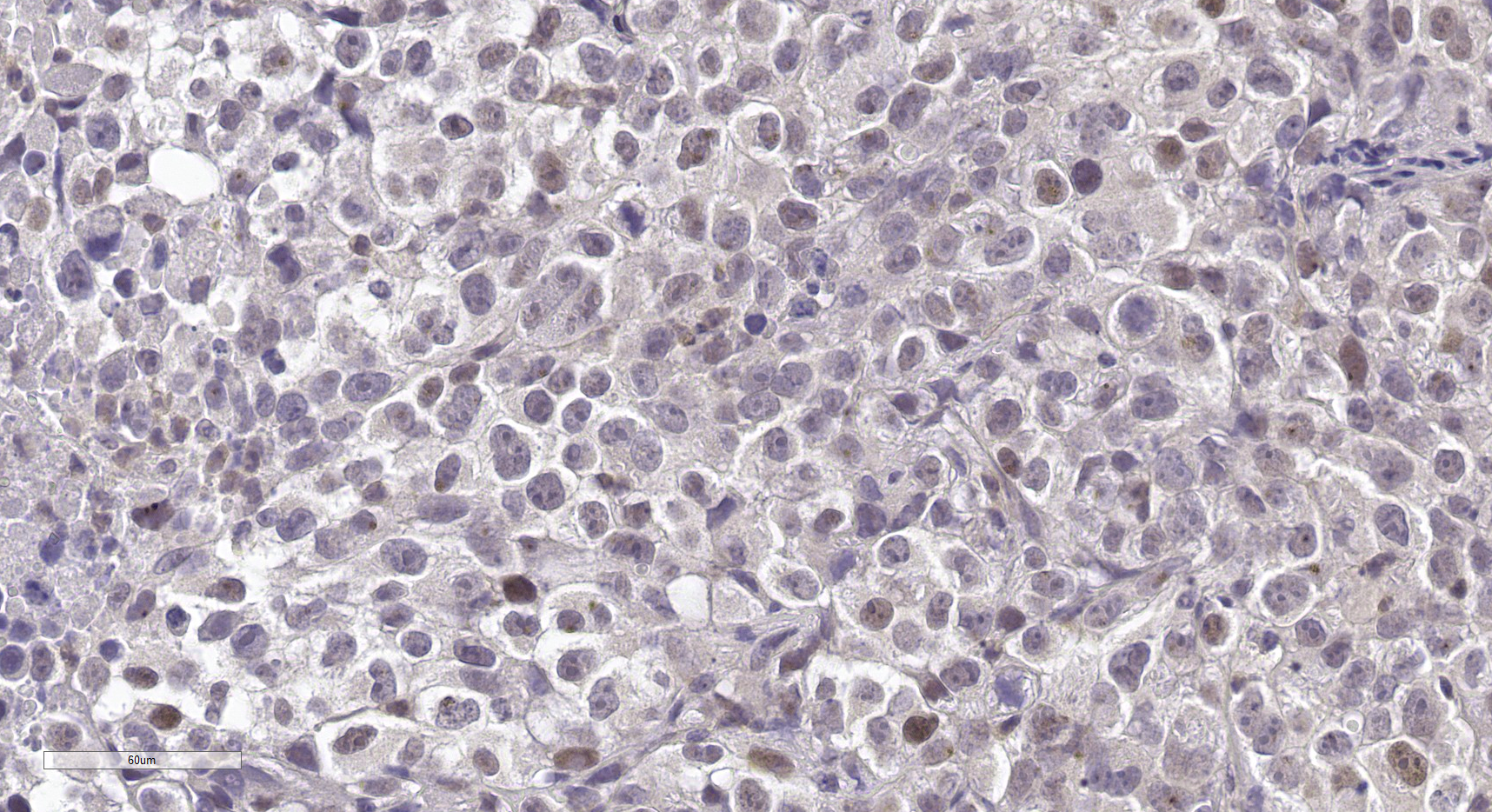

Supplement: Supplementary file 14 — Figure EV1 Source Data [file 44321_2026_414_MOESM14_ESM.zip › Fig. EV1/EV1E/ES-2 shBMAL2#1 BMAL2 IHC .tif]

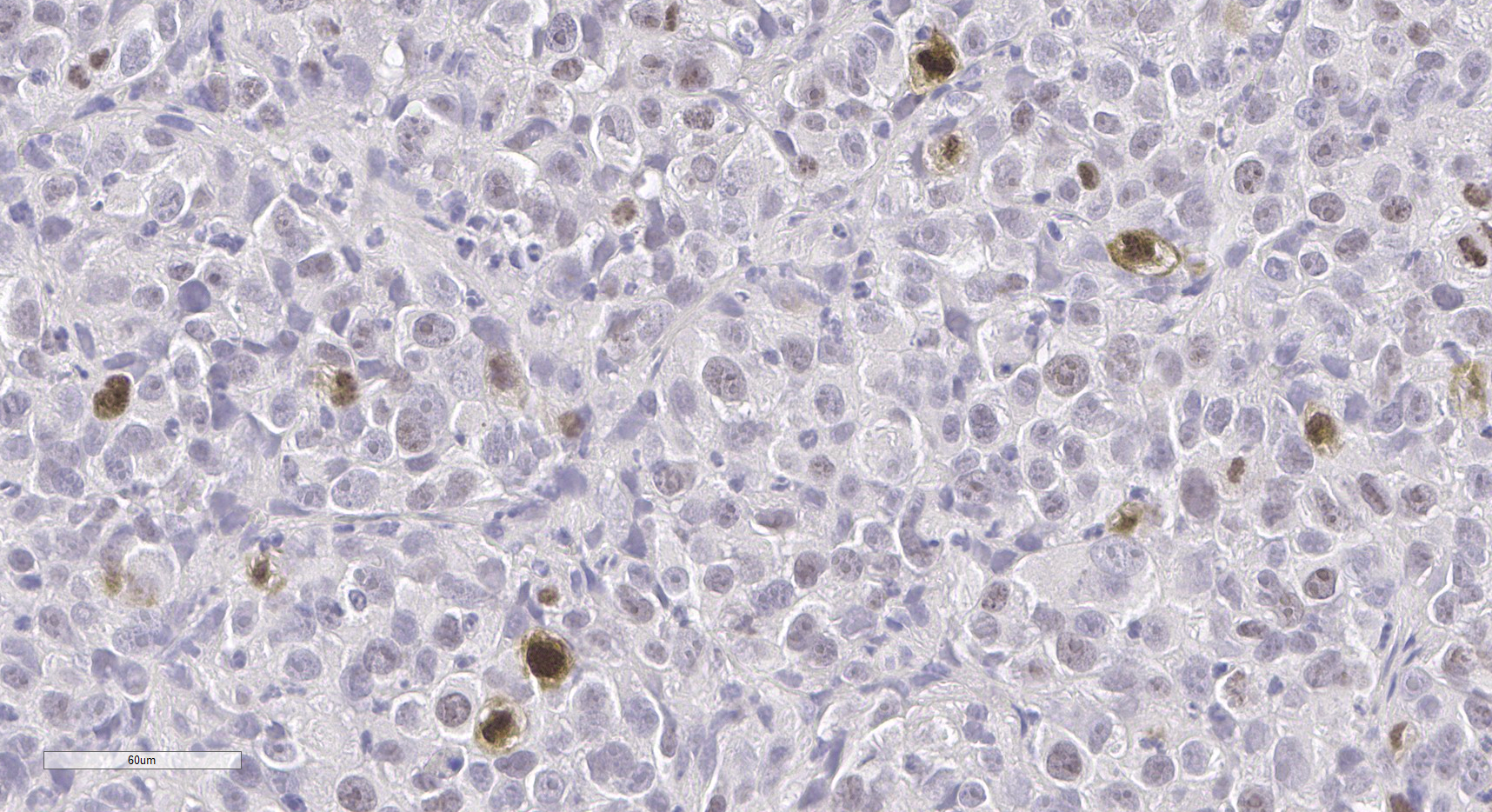

Supplement: Supplementary file 14 — Figure EV1 Source Data [file 44321_2026_414_MOESM14_ESM.zip › Fig. EV1/EV1E/ES-2 shBMAL2#1 Ki67 IHC.tif]

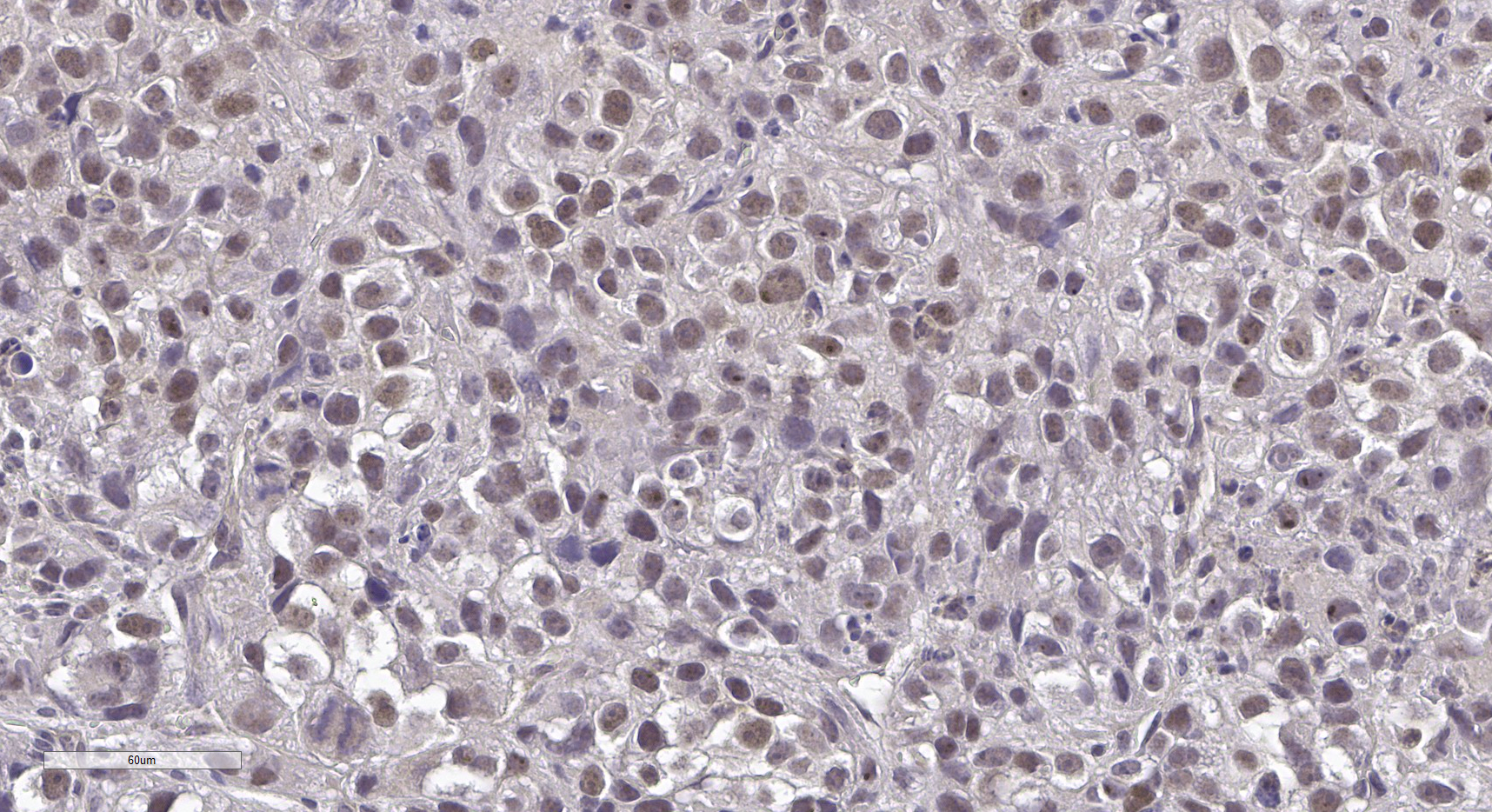

Supplement: Supplementary file 14 — Figure EV1 Source Data [file 44321_2026_414_MOESM14_ESM.zip › Fig. EV1/EV1E/ES-2 shCtrl BMAL2 IHC.tif]

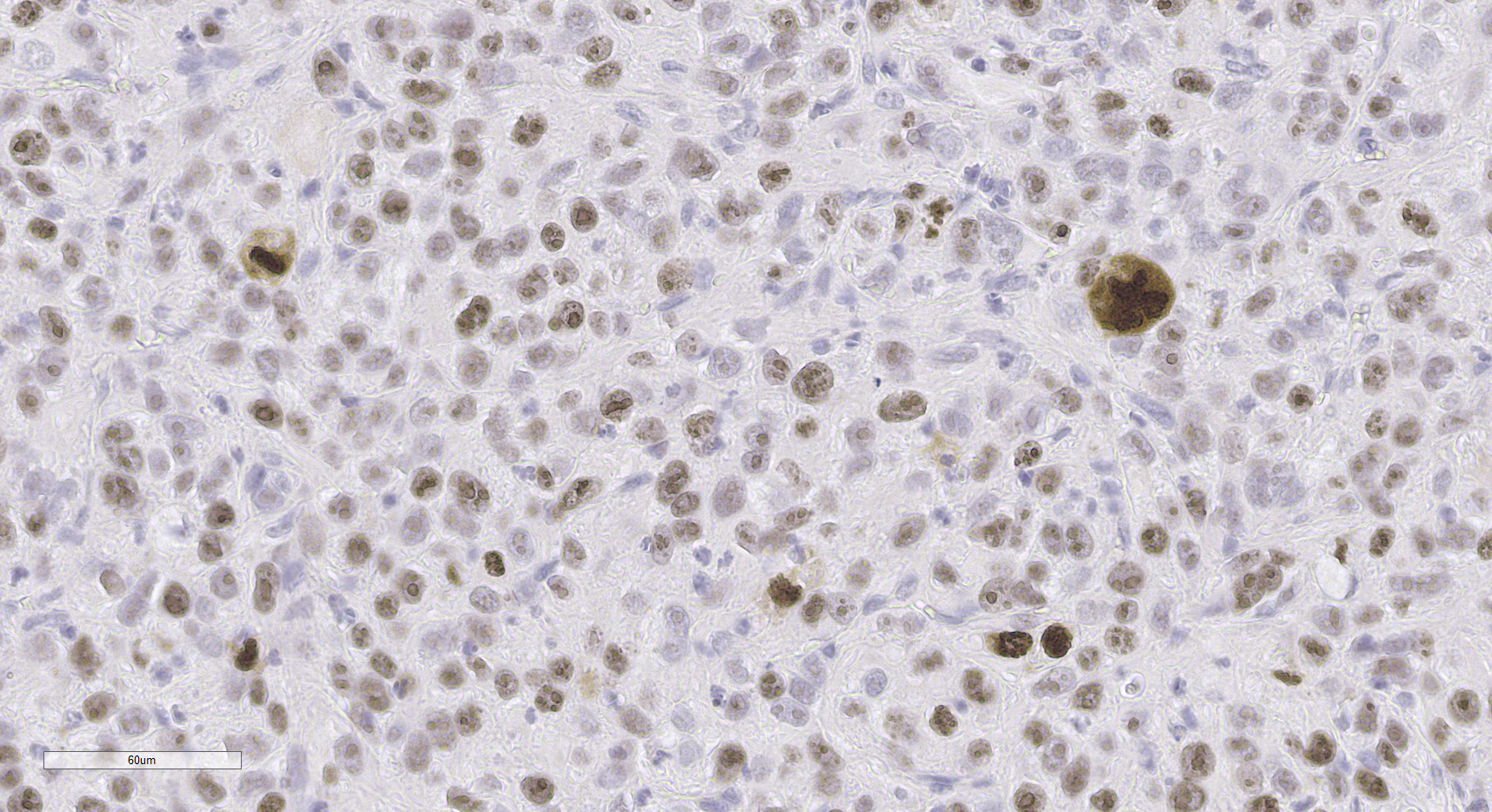

Supplement: Supplementary file 14 — Figure EV1 Source Data [file 44321_2026_414_MOESM14_ESM.zip › Fig. EV1/EV1E/ES-2 shCtrl Ki67 IHC.tif]

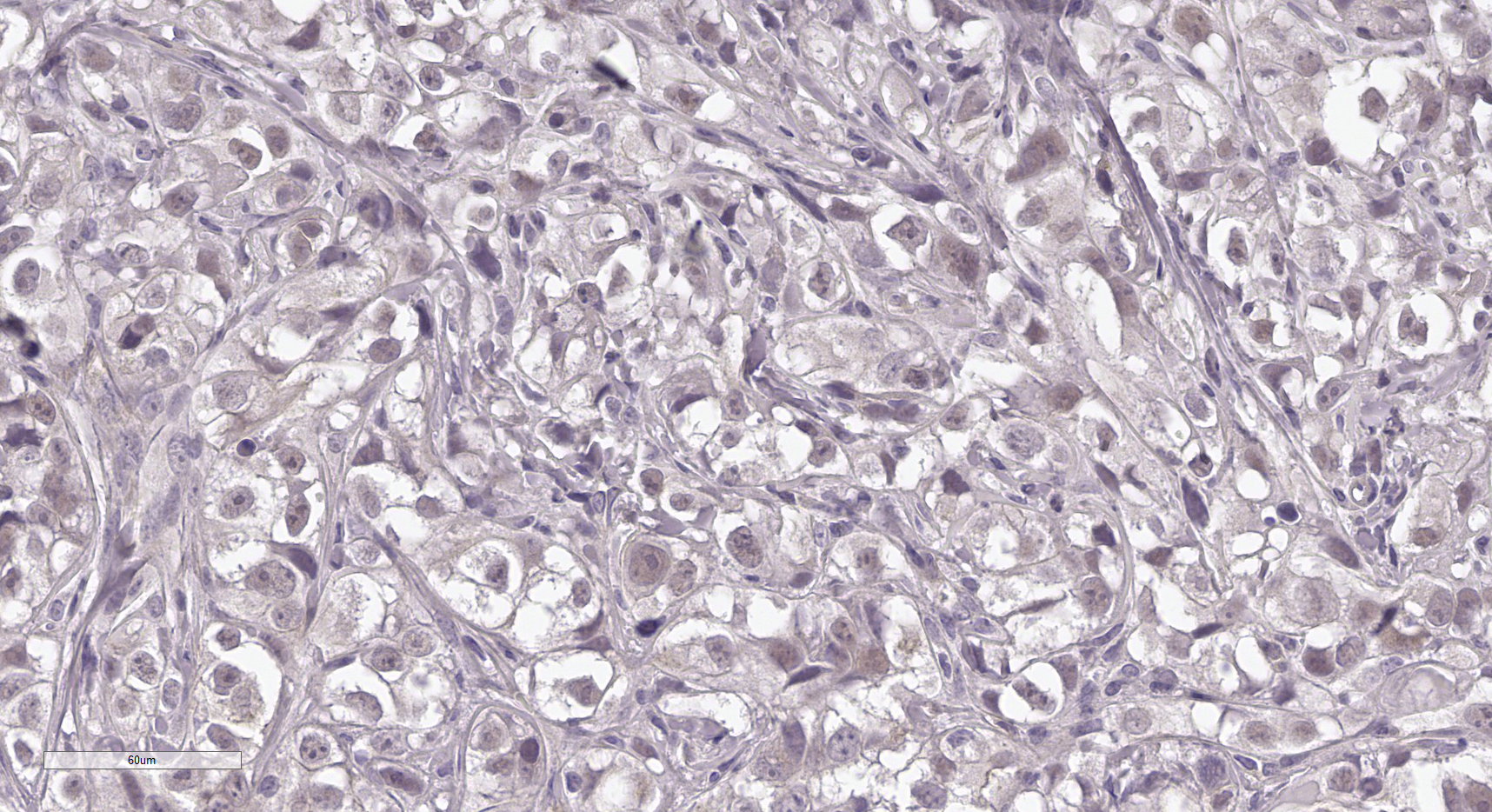

Supplement: Supplementary file 14 — Figure EV1 Source Data [file 44321_2026_414_MOESM14_ESM.zip › Fig. EV1/EV1E/JHOC5 shBMAL2#1 BMAL2 IHC .tif]

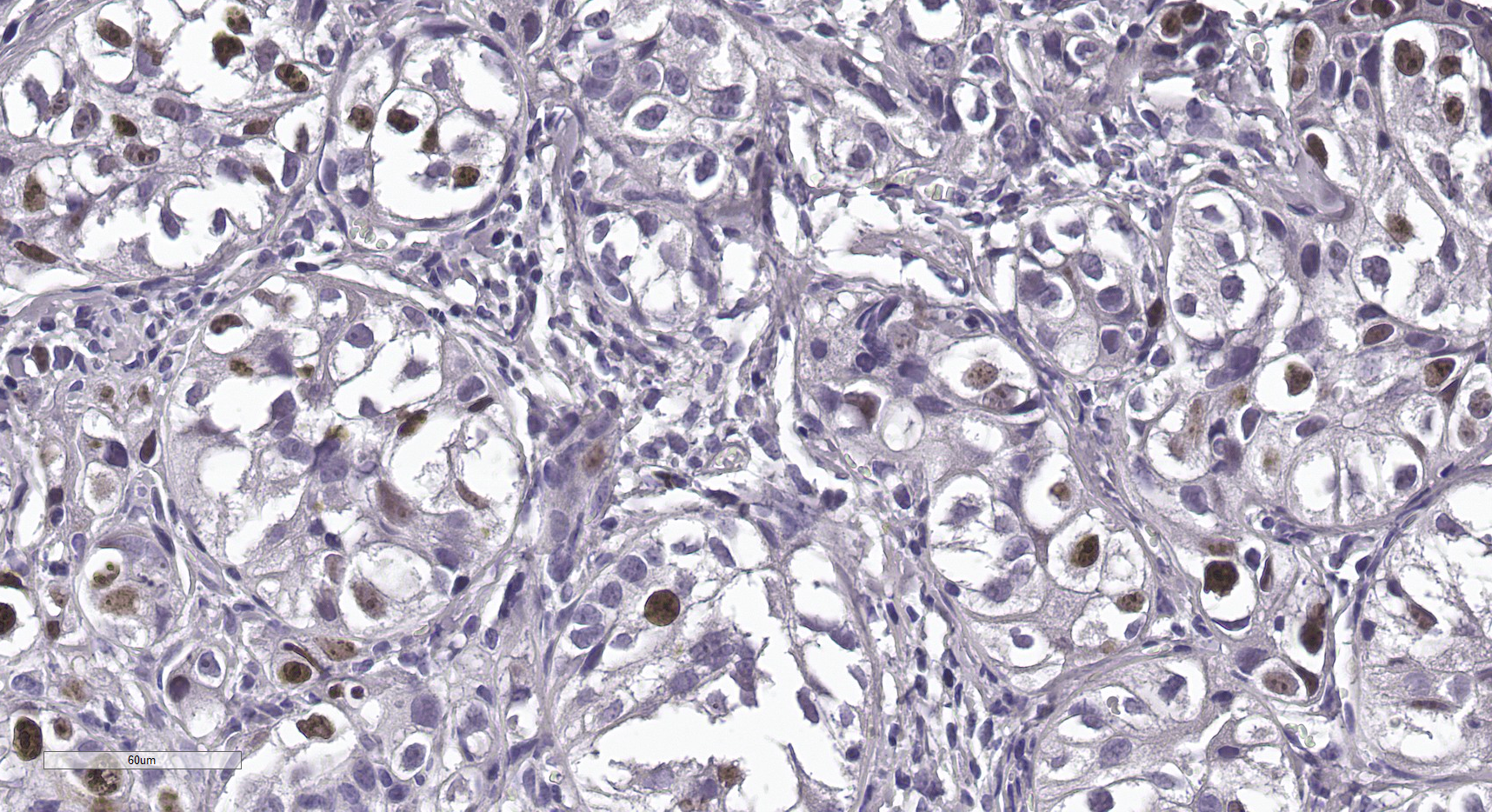

Supplement: Supplementary file 14 — Figure EV1 Source Data [file 44321_2026_414_MOESM14_ESM.zip › Fig. EV1/EV1E/JHOC5 shBMAL2#1 Ki67 IHC.tif]

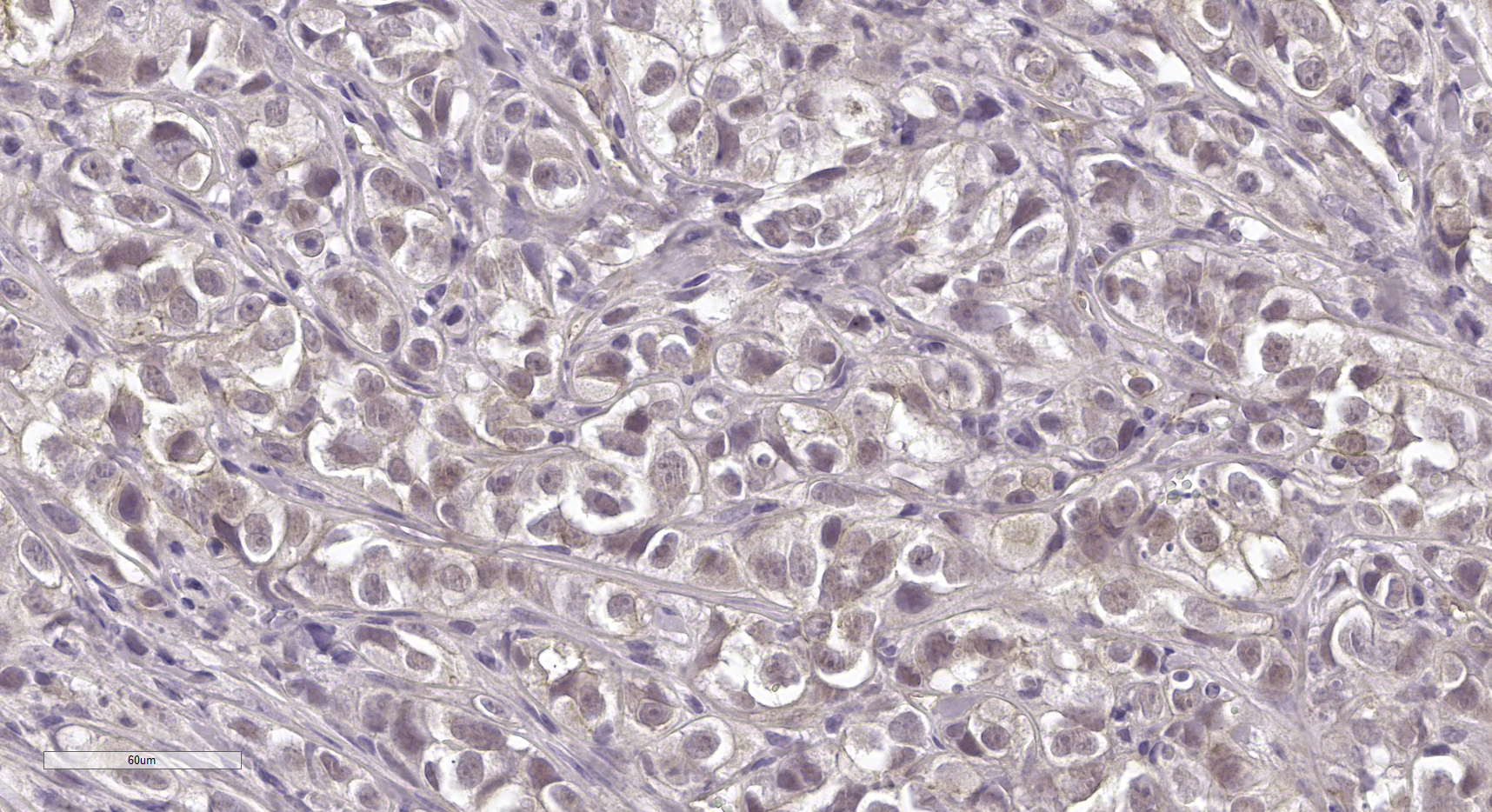

Supplement: Supplementary file 14 — Figure EV1 Source Data [file 44321_2026_414_MOESM14_ESM.zip › Fig. EV1/EV1E/JHOC5 shCtrl BMAL2 IHC.tif]

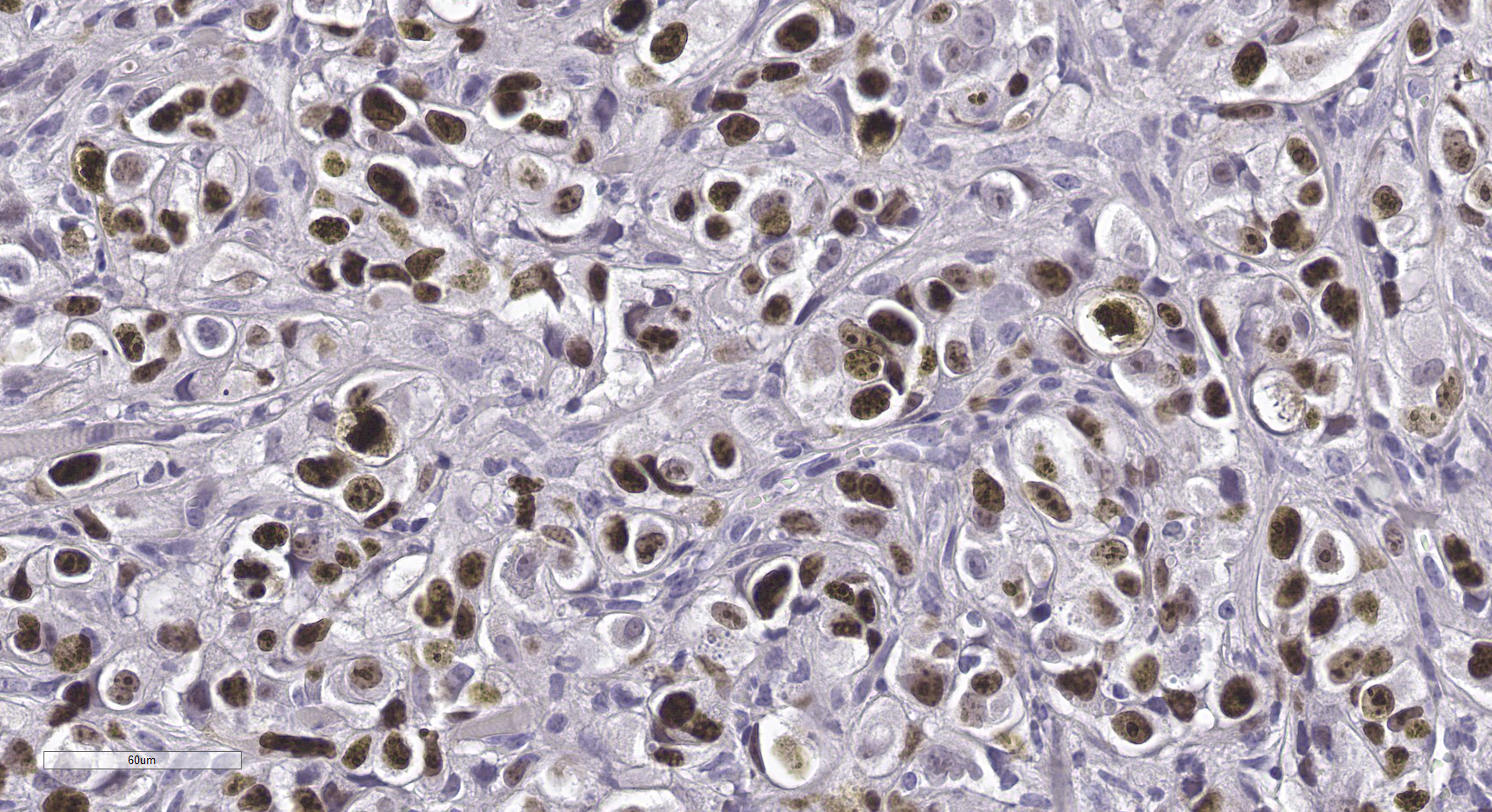

Supplement: Supplementary file 14 — Figure EV1 Source Data [file 44321_2026_414_MOESM14_ESM.zip › Fig. EV1/EV1E/JHOC5 shCtrl Ki67 IHC.tif]

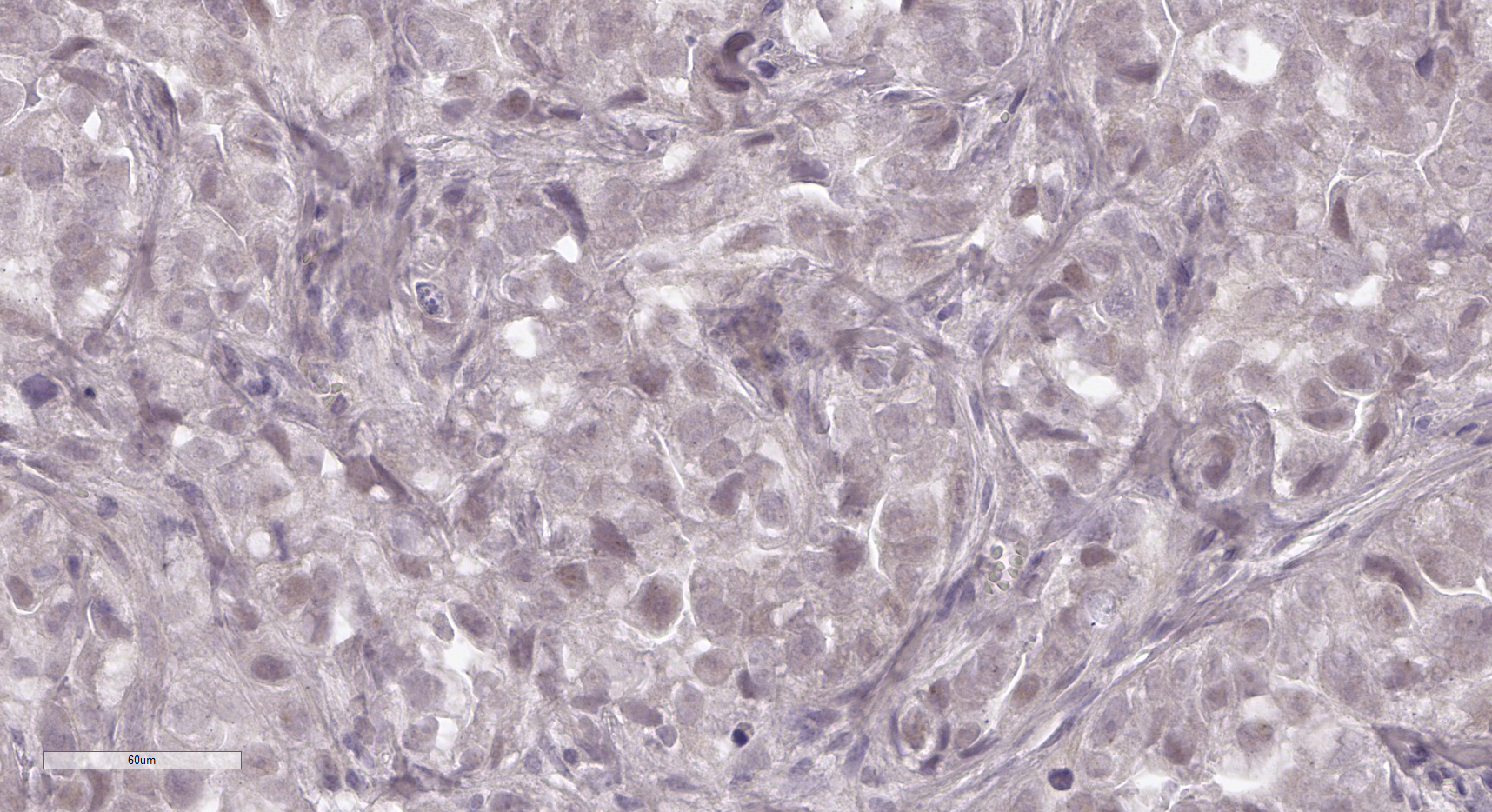

Supplement: Supplementary file 14 — Figure EV1 Source Data [file 44321_2026_414_MOESM14_ESM.zip › Fig. EV1/EV1E/OVISE shBMAL2#1 BMAL2 IHC.tif]

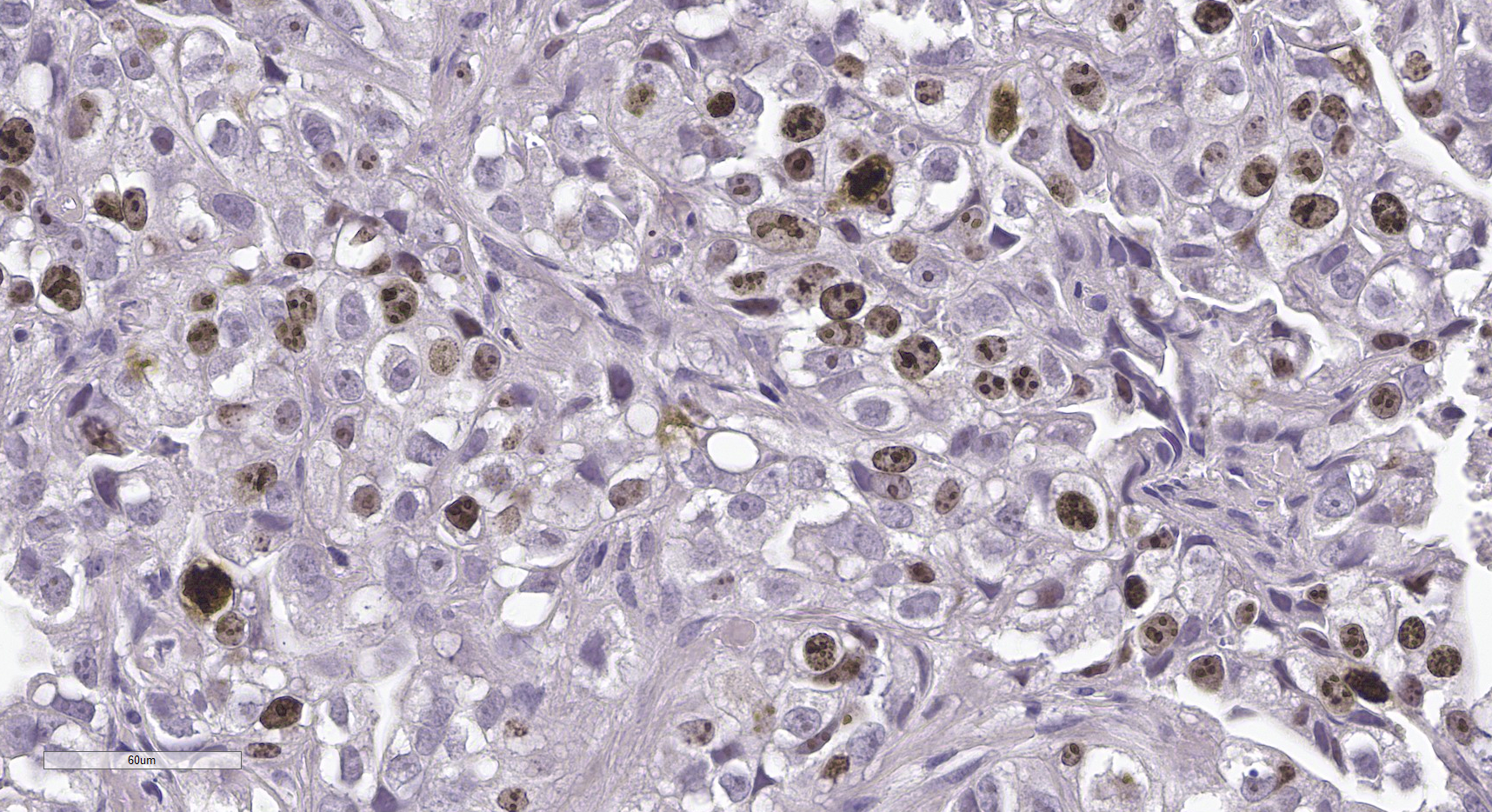

Supplement: Supplementary file 14 — Figure EV1 Source Data [file 44321_2026_414_MOESM14_ESM.zip › Fig. EV1/EV1E/OVISE shBMAL2#1 Ki67 IHC.tif]

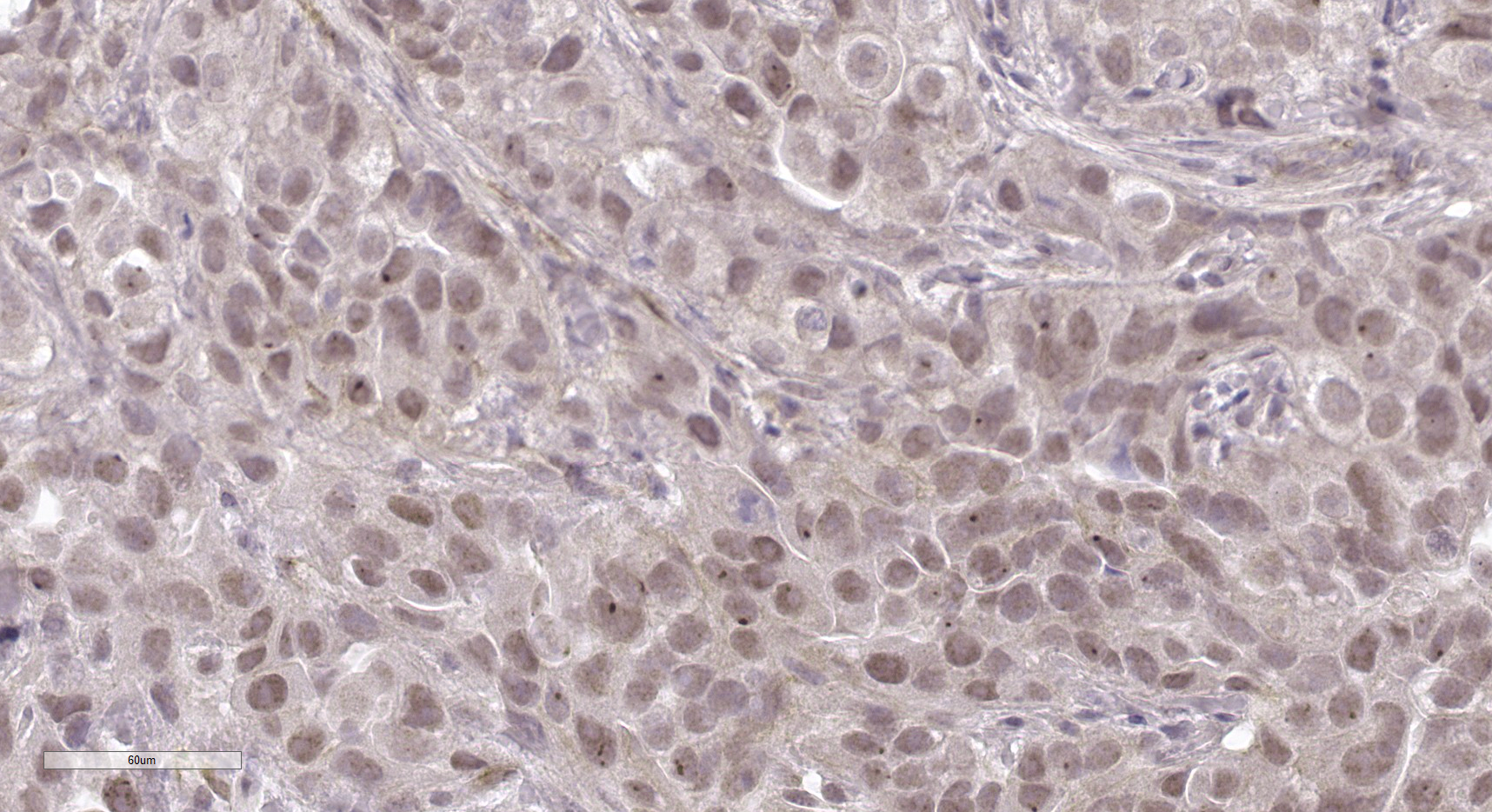

Supplement: Supplementary file 14 — Figure EV1 Source Data [file 44321_2026_414_MOESM14_ESM.zip › Fig. EV1/EV1E/OVISE shCtrl BMAL2 IHC.tif]

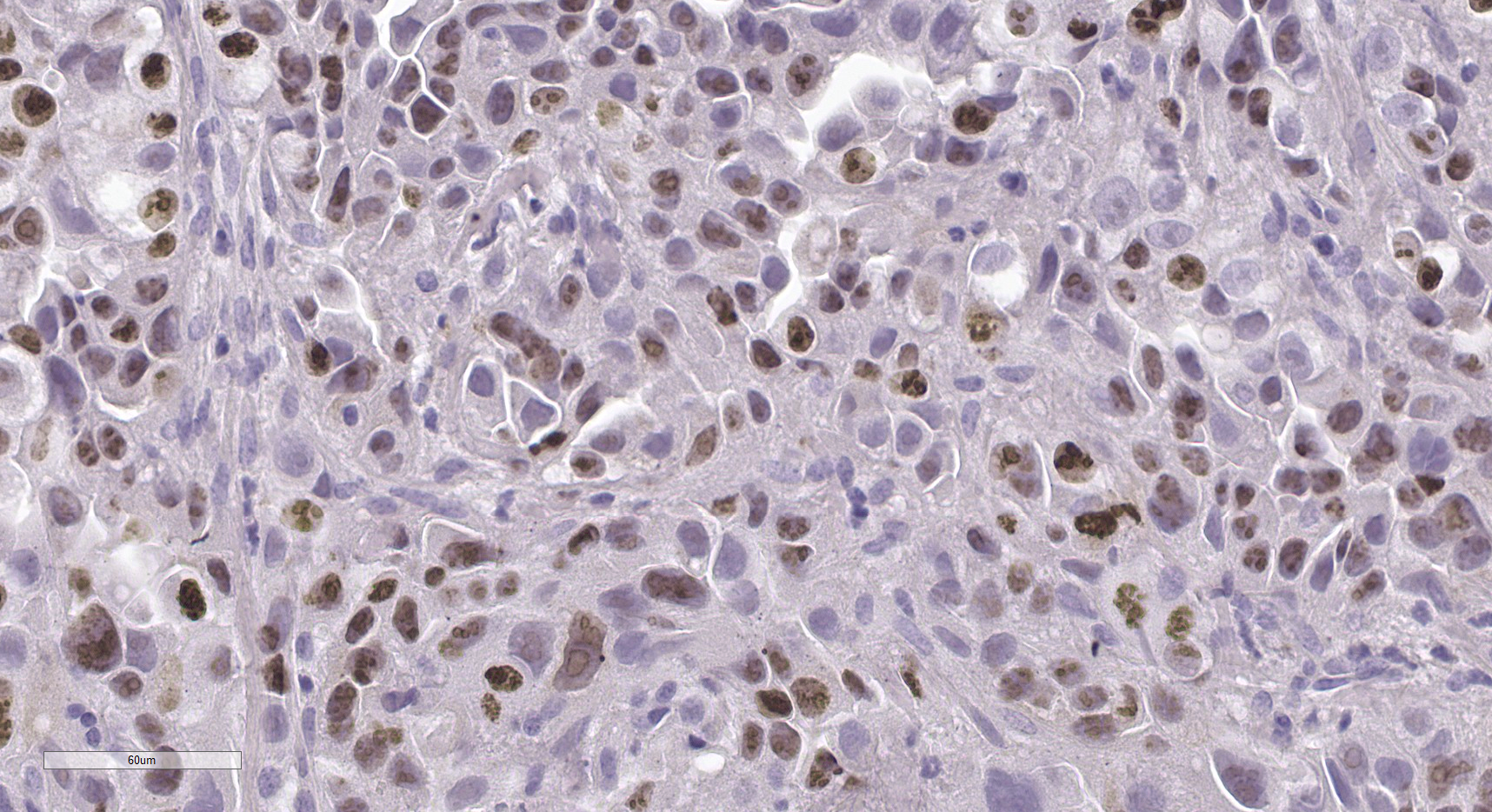

Supplement: Supplementary file 14 — Figure EV1 Source Data [file 44321_2026_414_MOESM14_ESM.zip › Fig. EV1/EV1E/OVISE shCtrl Ki67 IHC.tif]

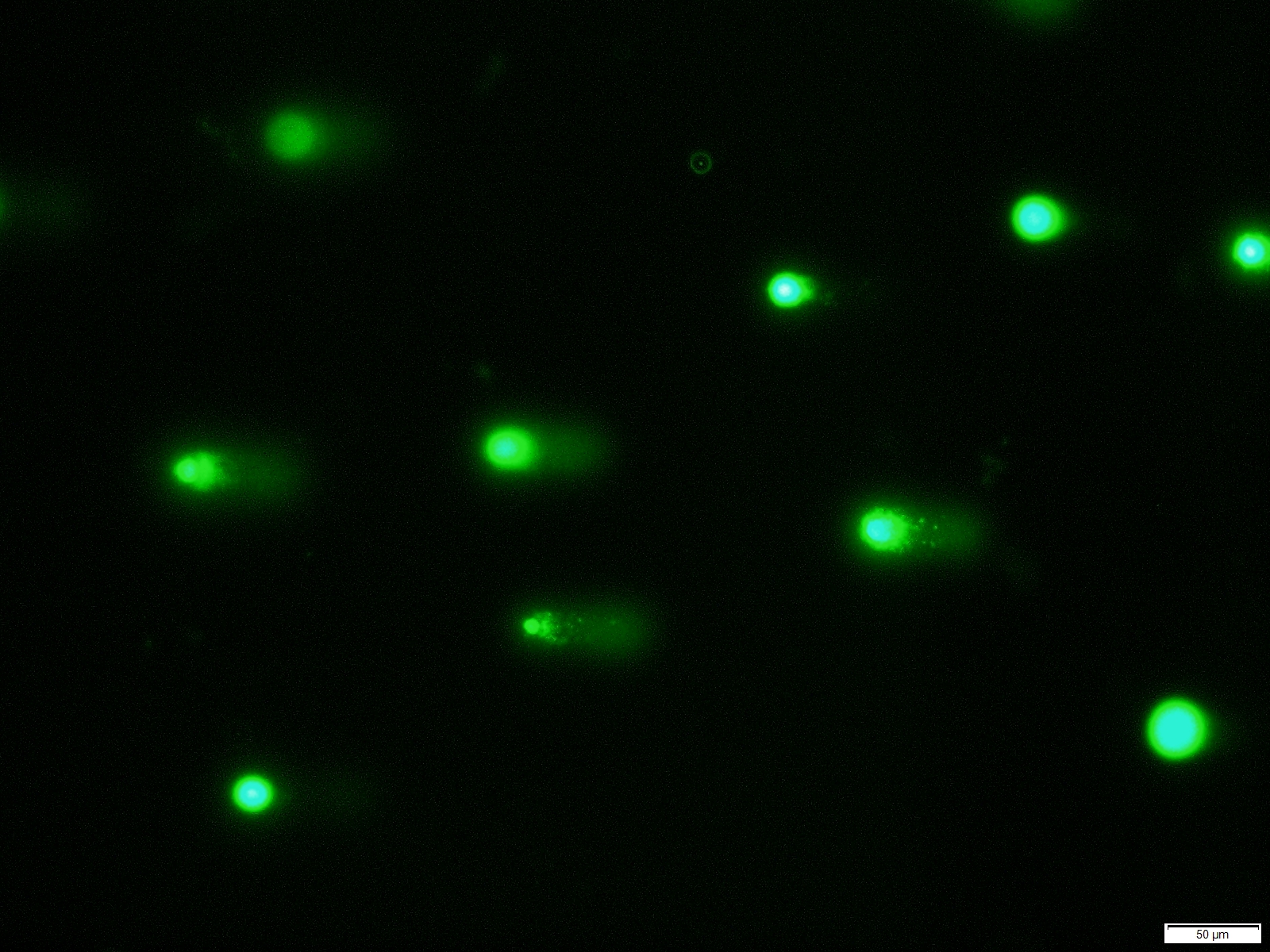

Supplement: Supplementary file 15 — Figure EV3 Source Data [file 44321_2026_414_MOESM15_ESM.zip › Fig. EV3/EV3A/ES-2 shBMAL2#1.jpg]

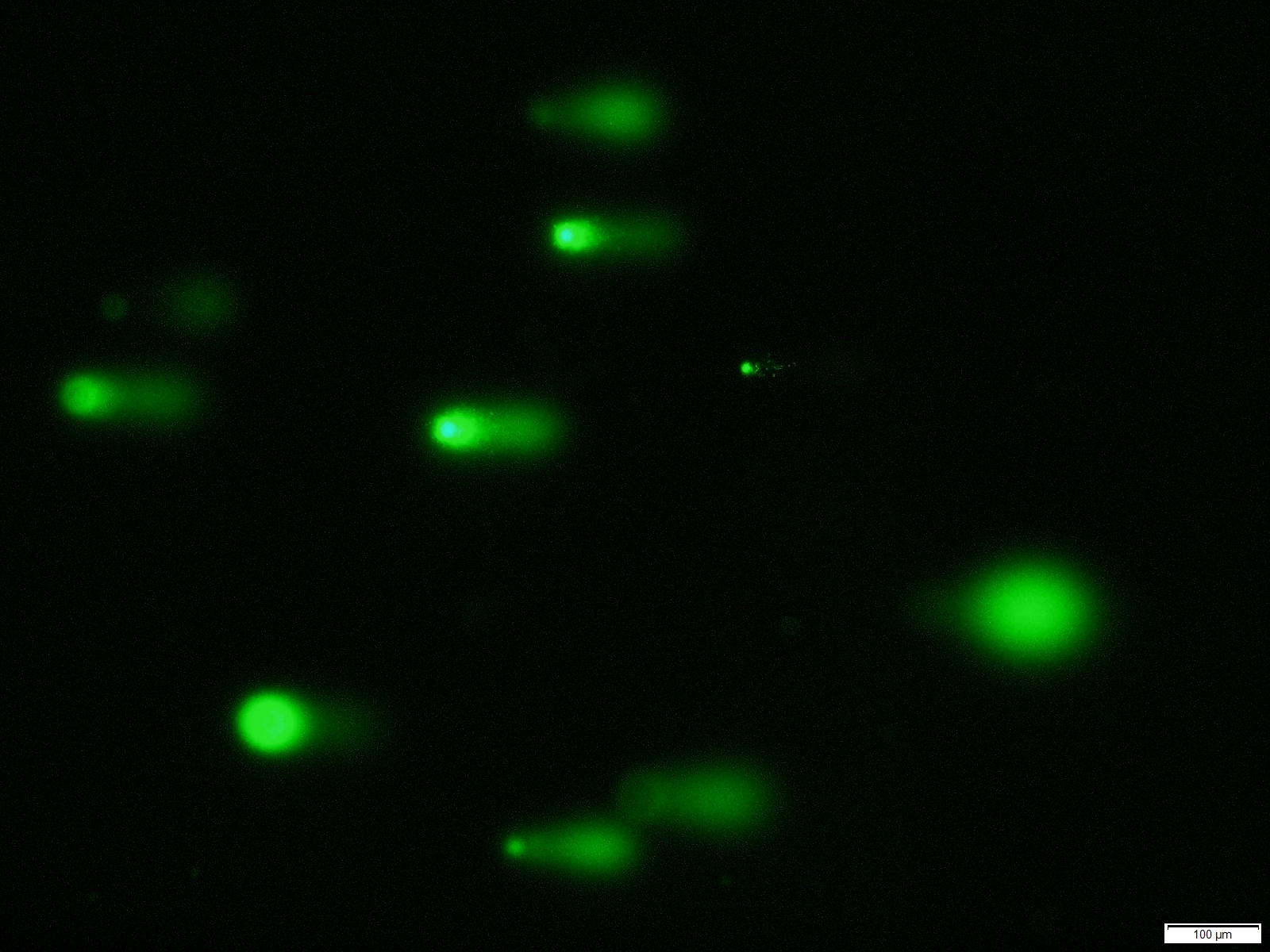

Supplement: Supplementary file 15 — Figure EV3 Source Data [file 44321_2026_414_MOESM15_ESM.zip › Fig. EV3/EV3A/ES-2 shBMAL2#2.jpg]

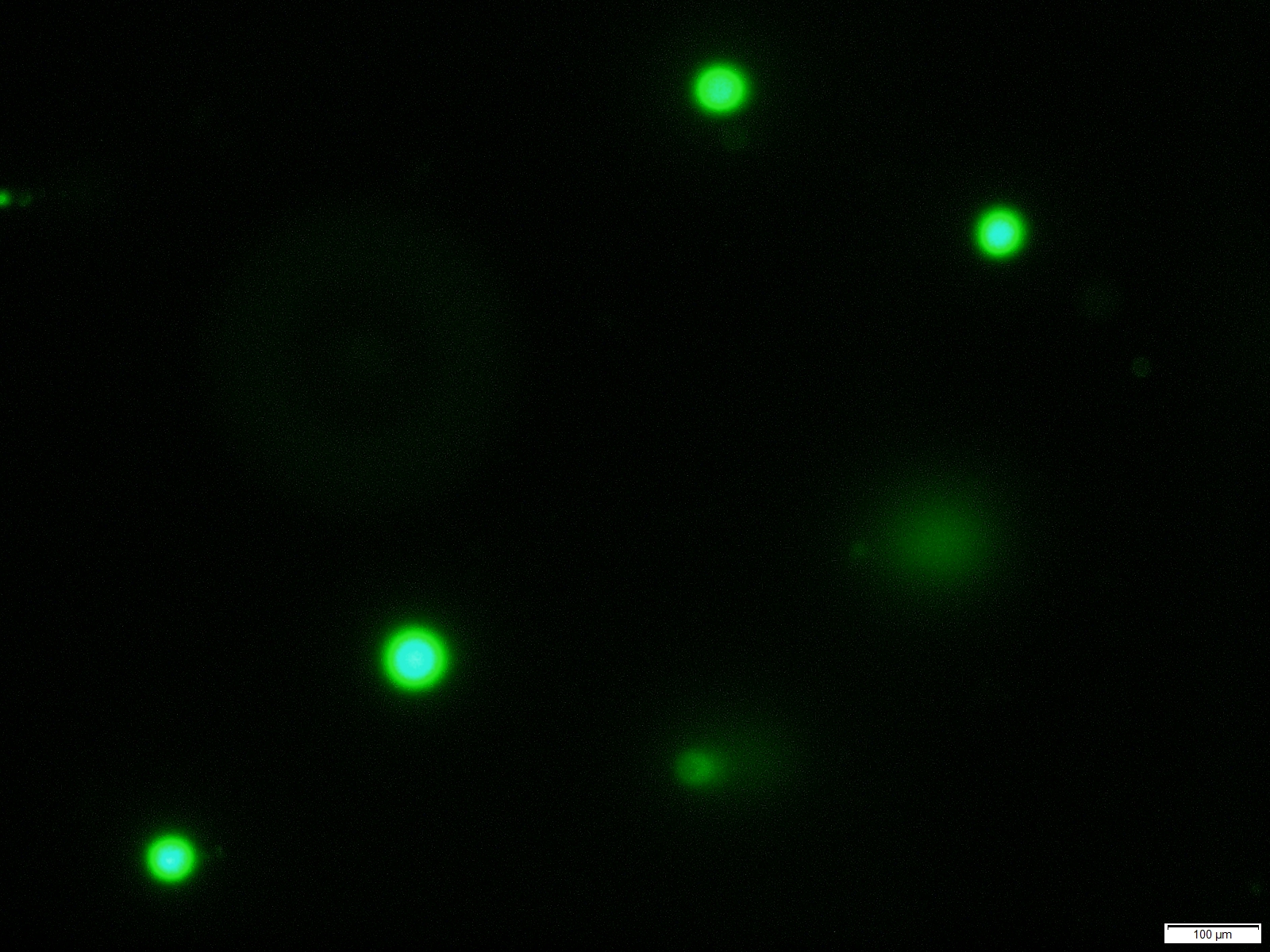

Supplement: Supplementary file 15 — Figure EV3 Source Data [file 44321_2026_414_MOESM15_ESM.zip › Fig. EV3/EV3A/ES-2 shCtrl.jpg]

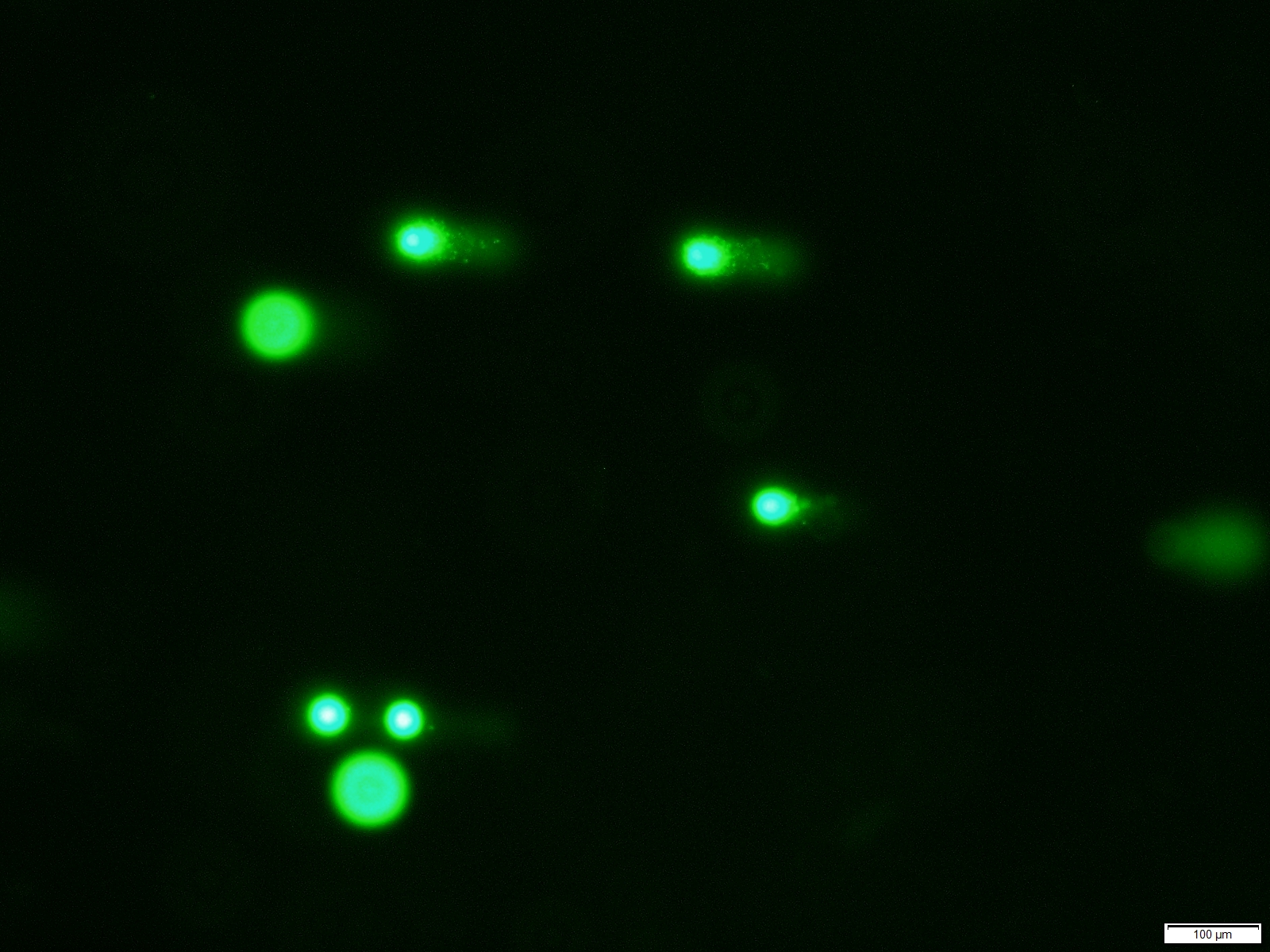

Supplement: Supplementary file 15 — Figure EV3 Source Data [file 44321_2026_414_MOESM15_ESM.zip › Fig. EV3/EV3A/JHOC5 shBMAL2#1.jpg]

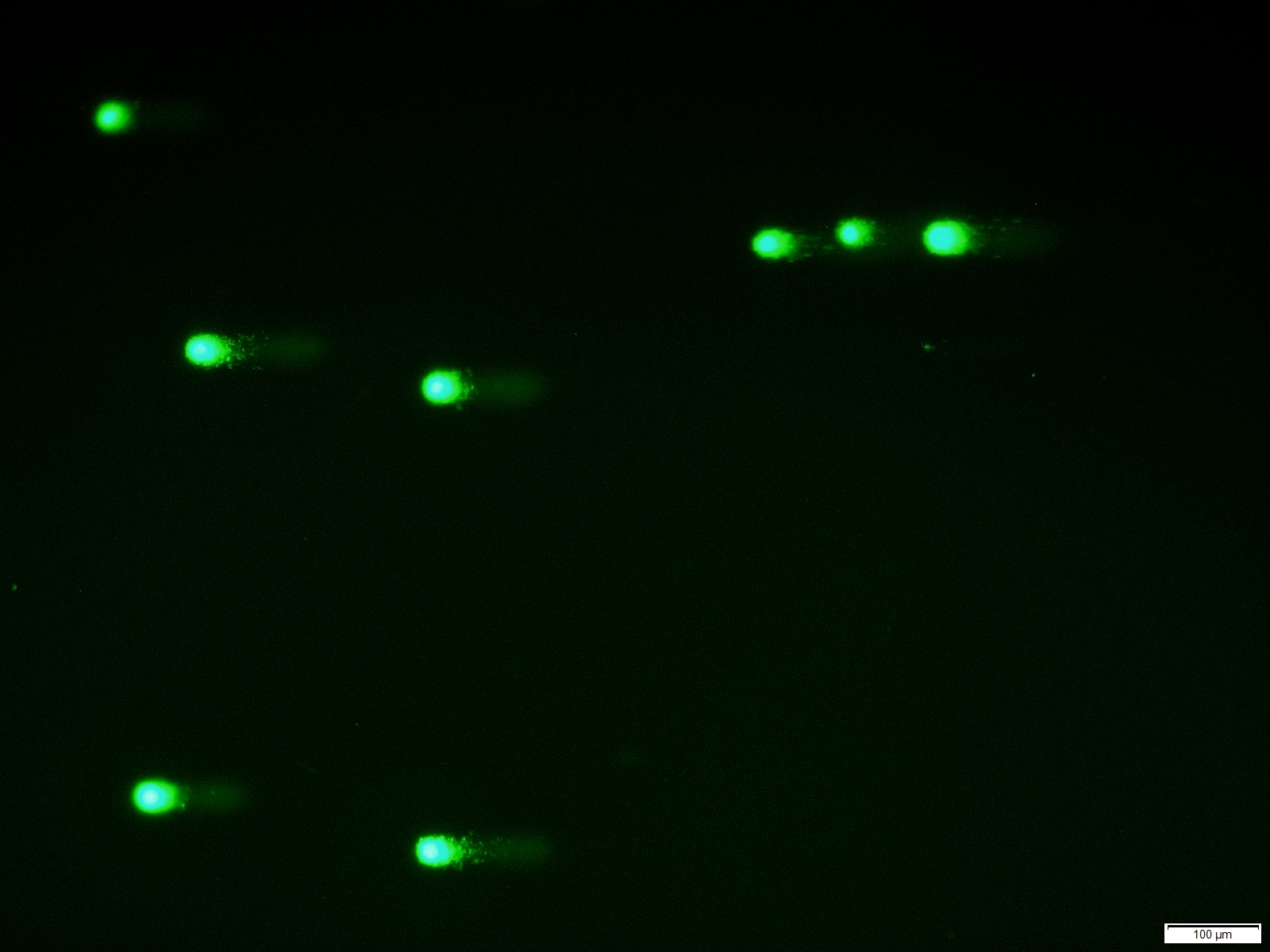

Supplement: Supplementary file 15 — Figure EV3 Source Data [file 44321_2026_414_MOESM15_ESM.zip › Fig. EV3/EV3A/JHOC5 shBMAL2#2.jpg]

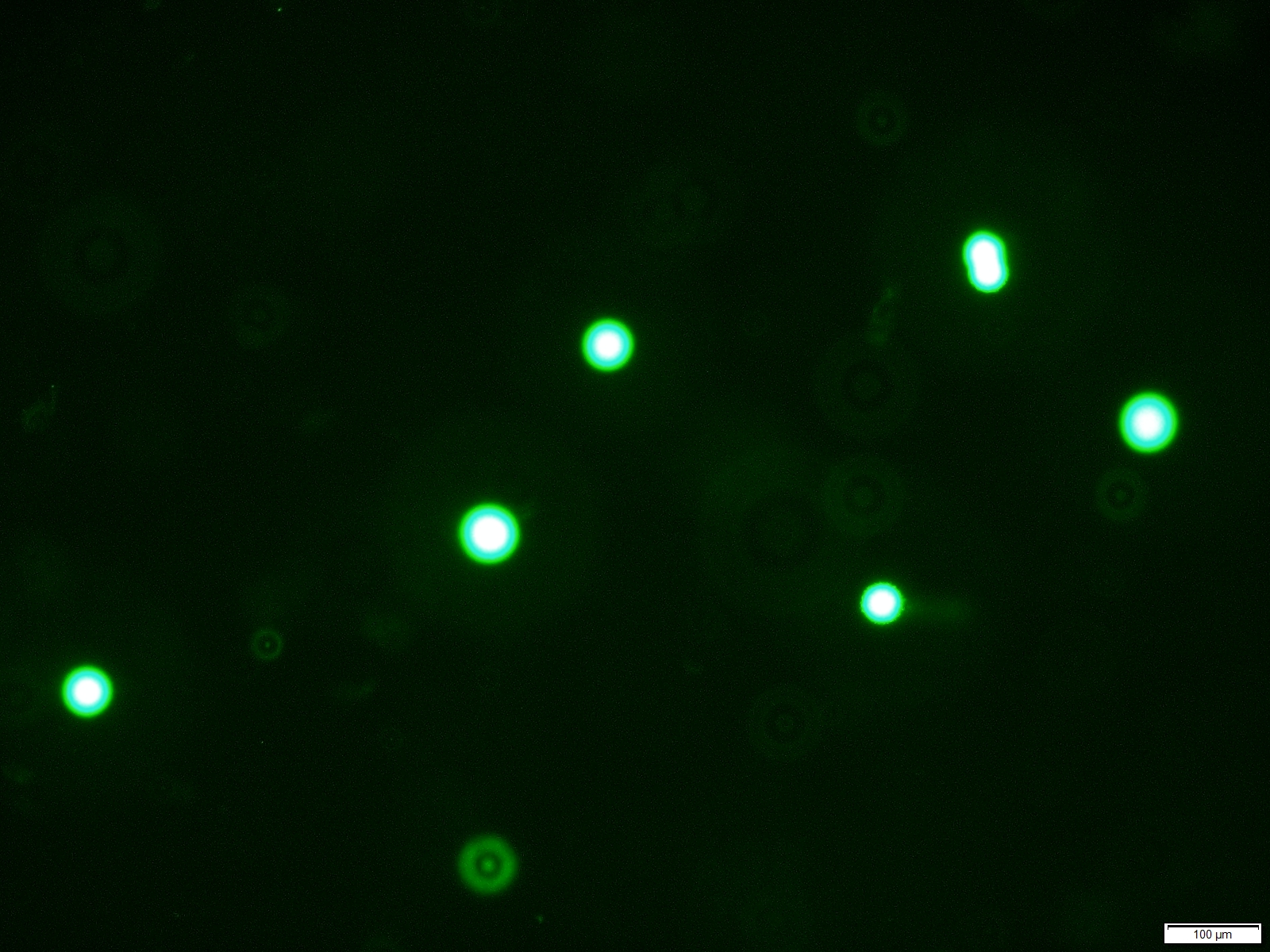

Supplement: Supplementary file 15 — Figure EV3 Source Data [file 44321_2026_414_MOESM15_ESM.zip › Fig. EV3/EV3A/JHOC5 shCtrl.jpg]

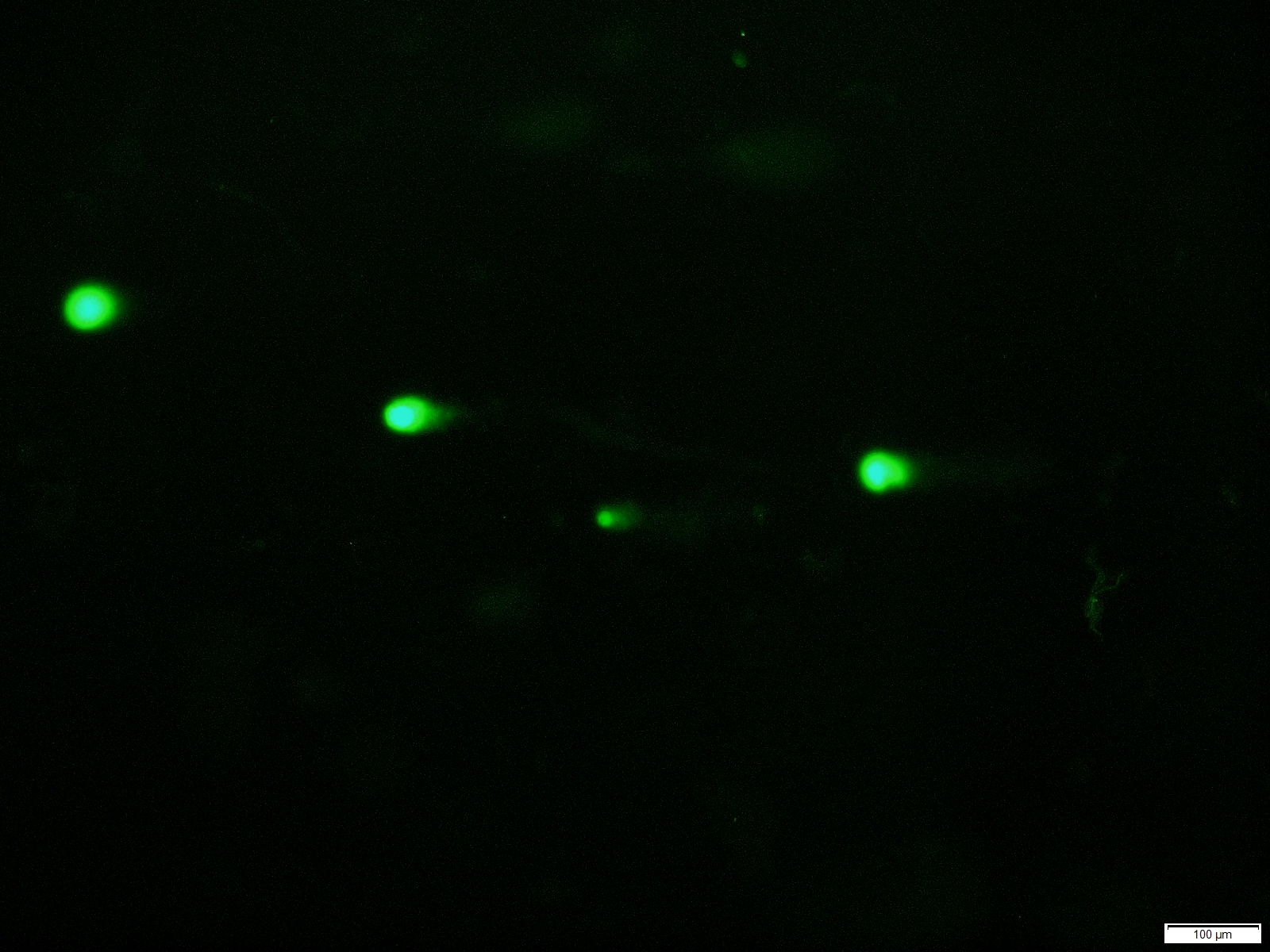

Supplement: Supplementary file 15 — Figure EV3 Source Data [file 44321_2026_414_MOESM15_ESM.zip › Fig. EV3/EV3A/JHOC9 shBMAL2#1.jpg]

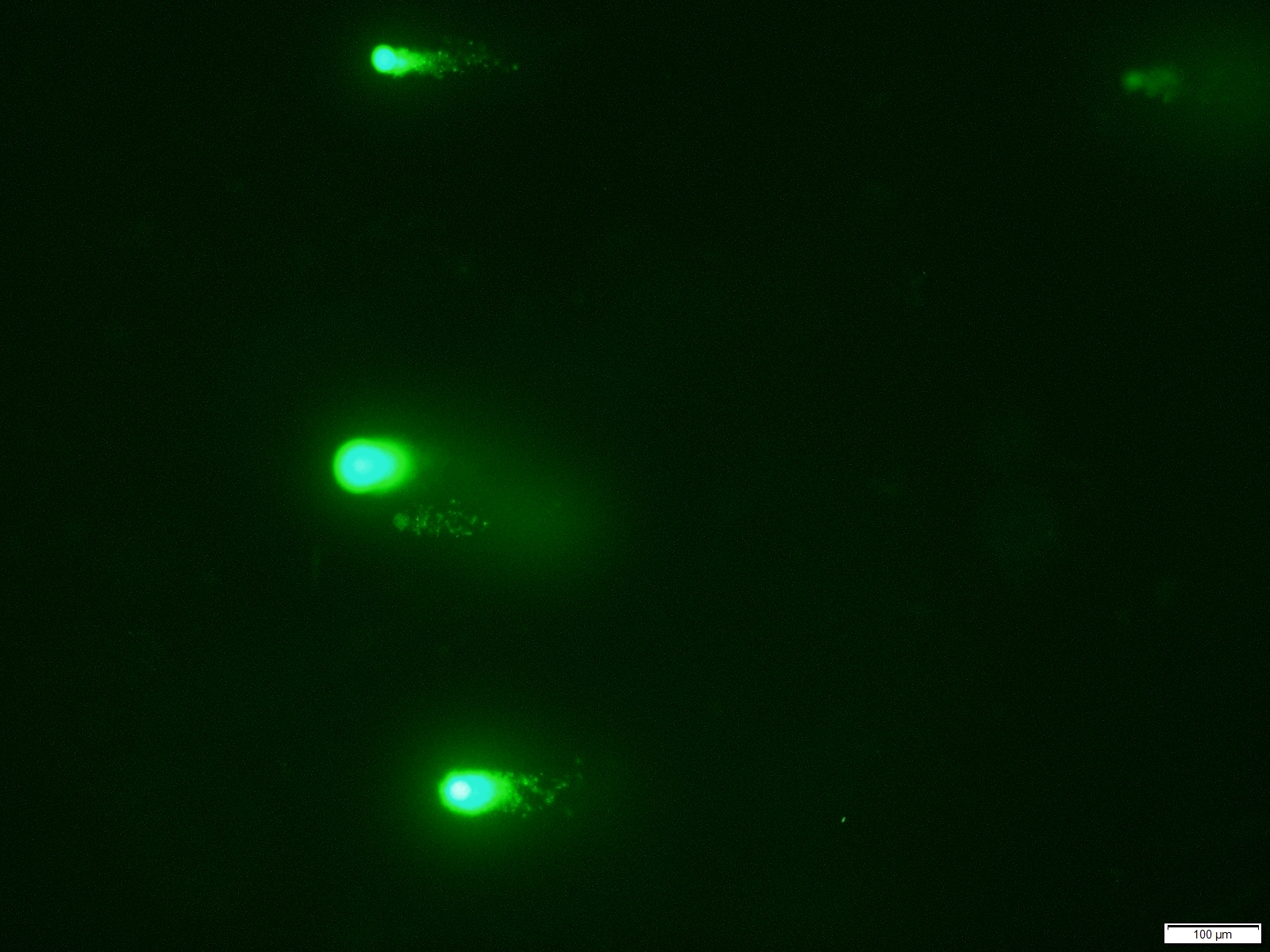

Supplement: Supplementary file 15 — Figure EV3 Source Data [file 44321_2026_414_MOESM15_ESM.zip › Fig. EV3/EV3A/JHOC9 shBMAL2#2.jpg]

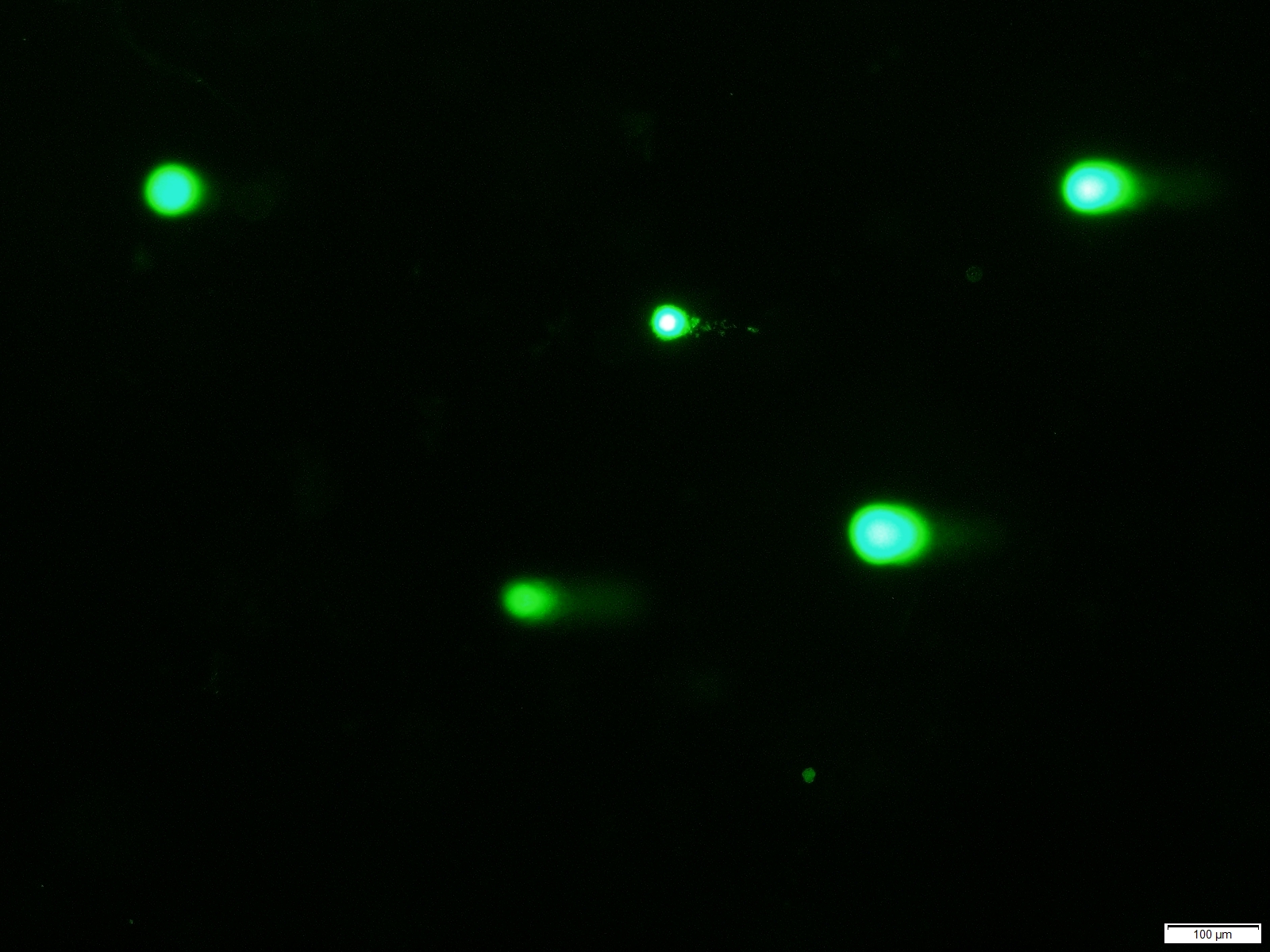

Supplement: Supplementary file 15 — Figure EV3 Source Data [file 44321_2026_414_MOESM15_ESM.zip › Fig. EV3/EV3A/JHOC9 shCtrl.jpg]

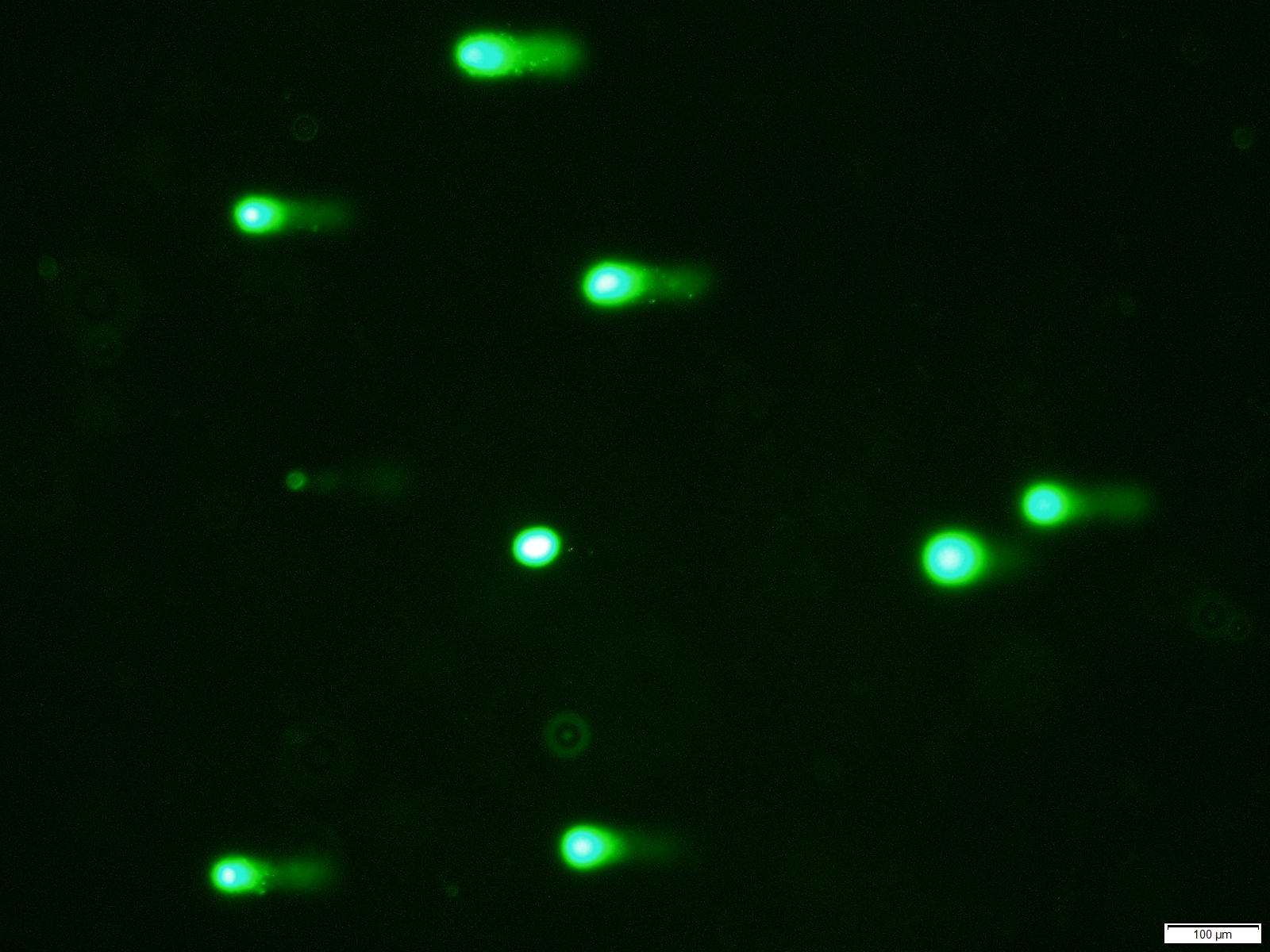

Supplement: Supplementary file 15 — Figure EV3 Source Data [file 44321_2026_414_MOESM15_ESM.zip › Fig. EV3/EV3A/OVCA429 shBMAL2#1.jpg]

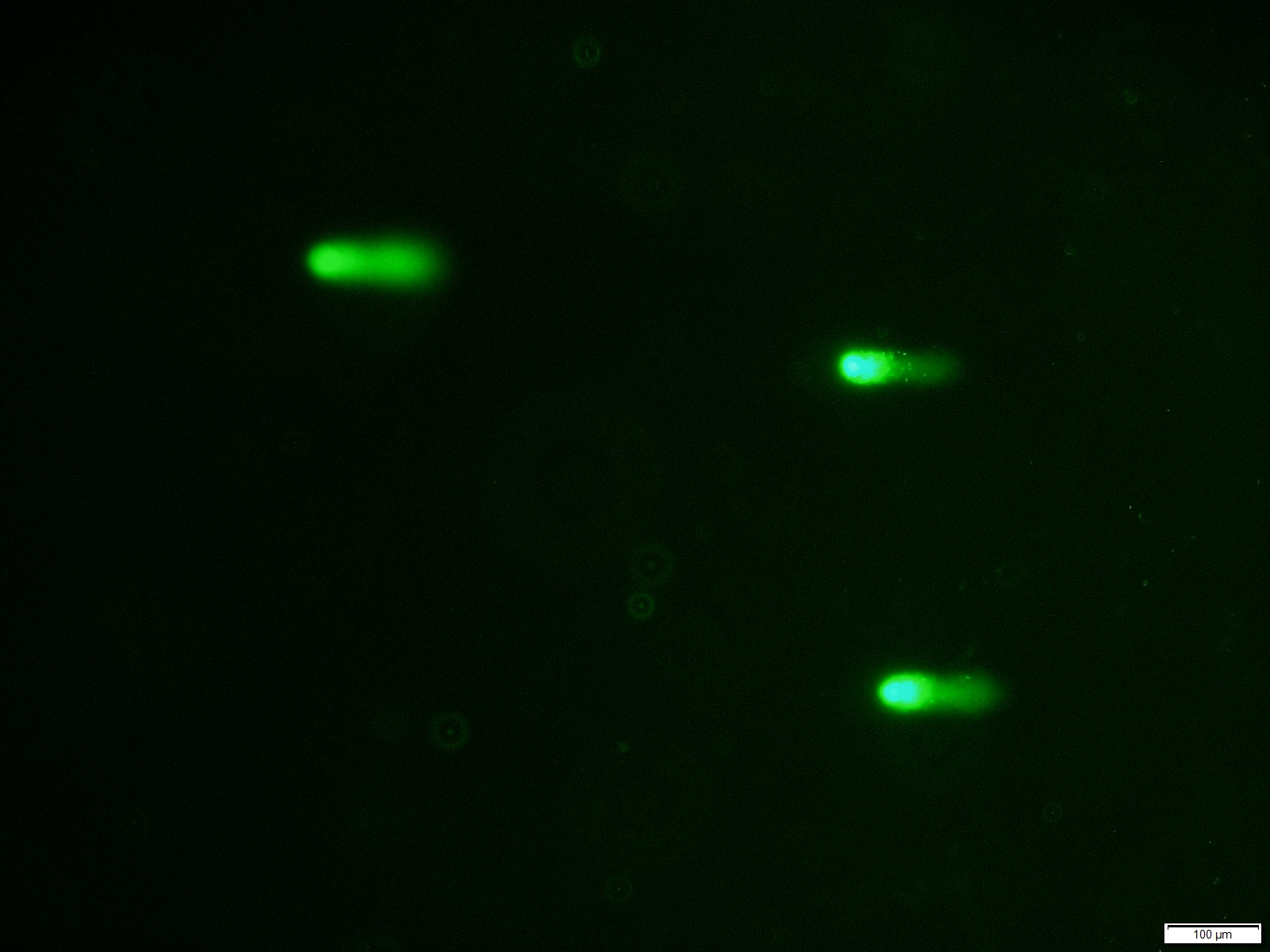

Supplement: Supplementary file 15 — Figure EV3 Source Data [file 44321_2026_414_MOESM15_ESM.zip › Fig. EV3/EV3A/OVCA429 shBMAL2#2.jpg]

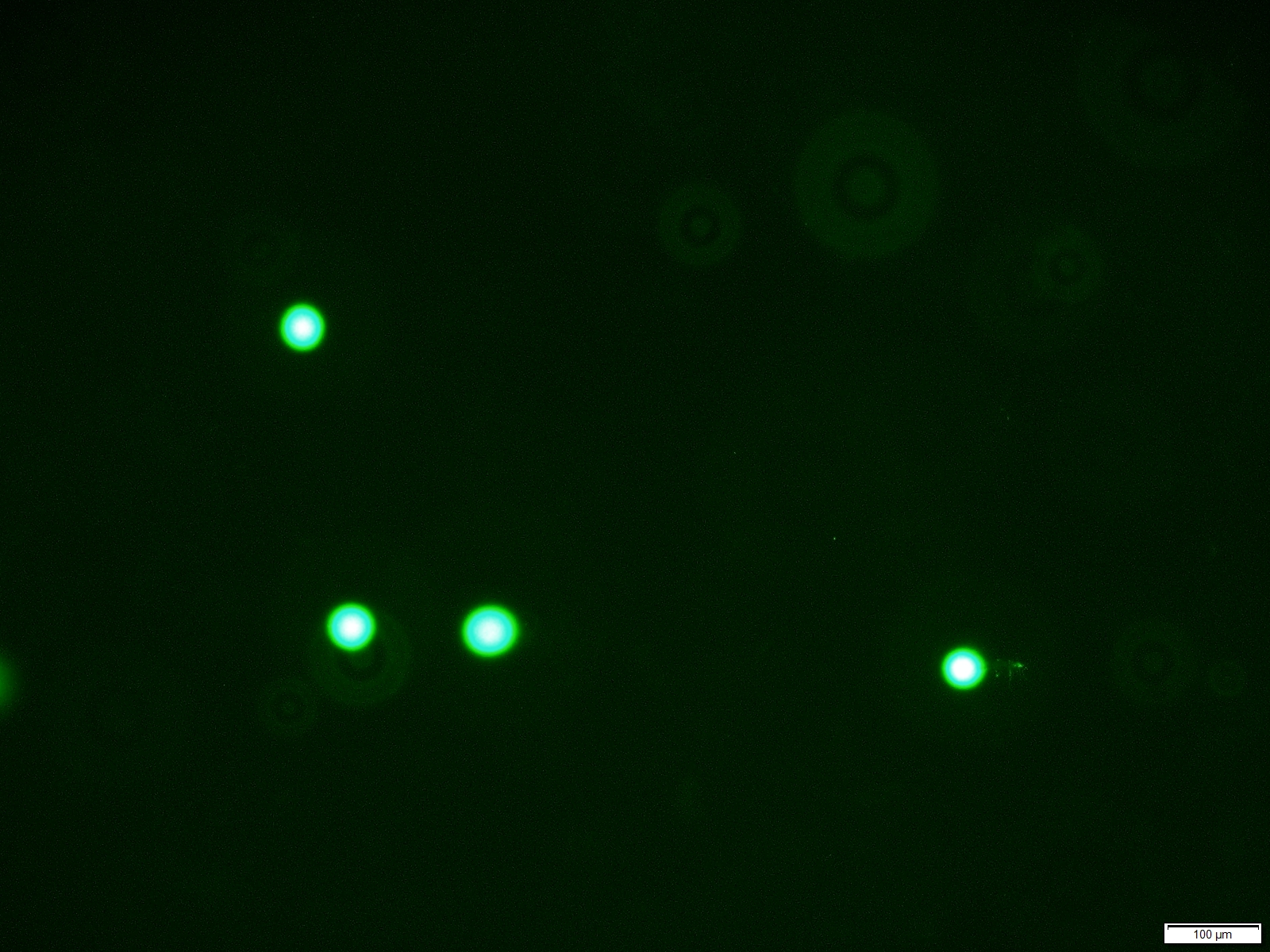

Supplement: Supplementary file 15 — Figure EV3 Source Data [file 44321_2026_414_MOESM15_ESM.zip › Fig. EV3/EV3A/OVCA429 shCtrl.jpg]

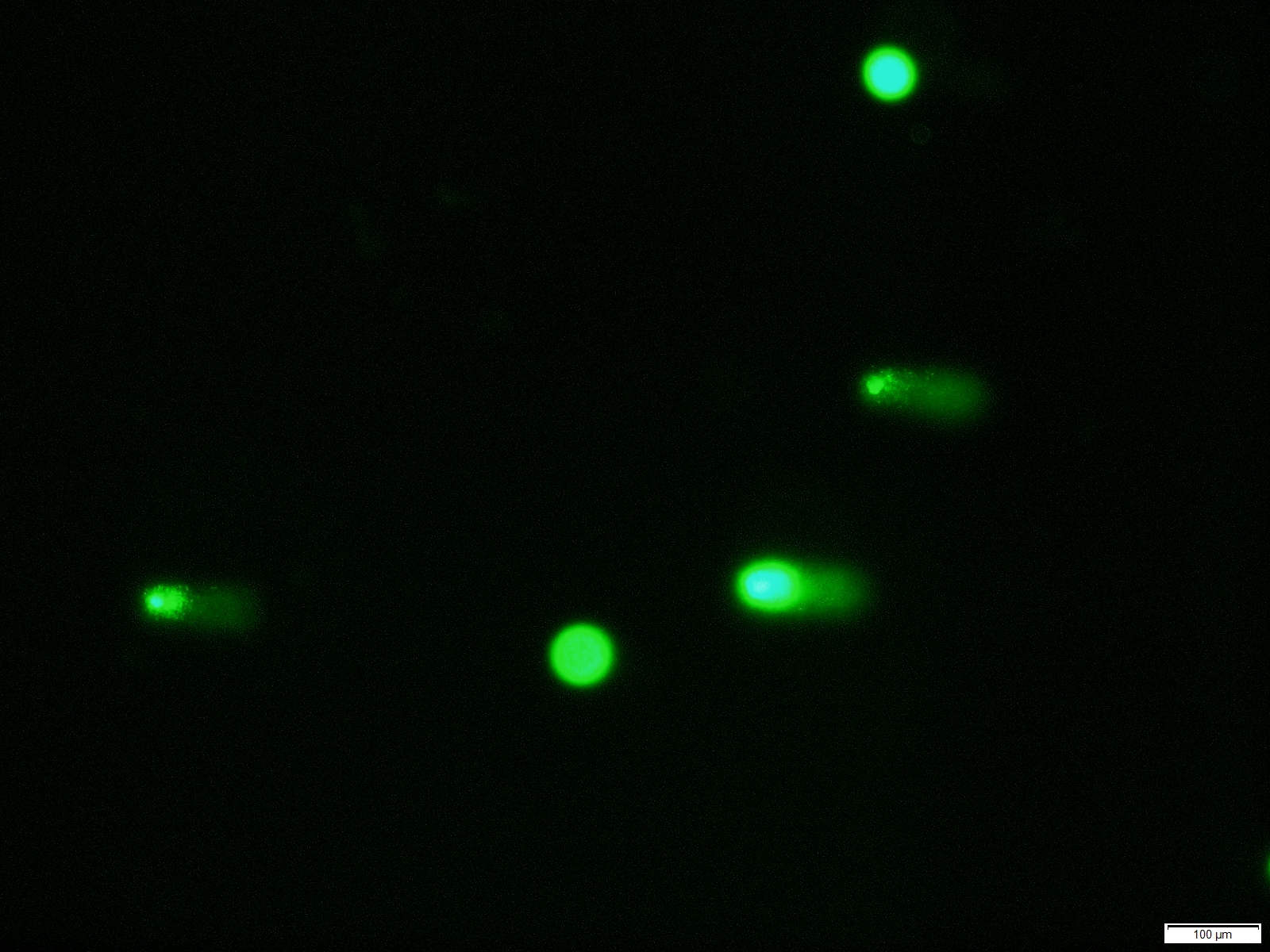

Supplement: Supplementary file 15 — Figure EV3 Source Data [file 44321_2026_414_MOESM15_ESM.zip › Fig. EV3/EV3A/OVISE shBMAL2#1.jpg]

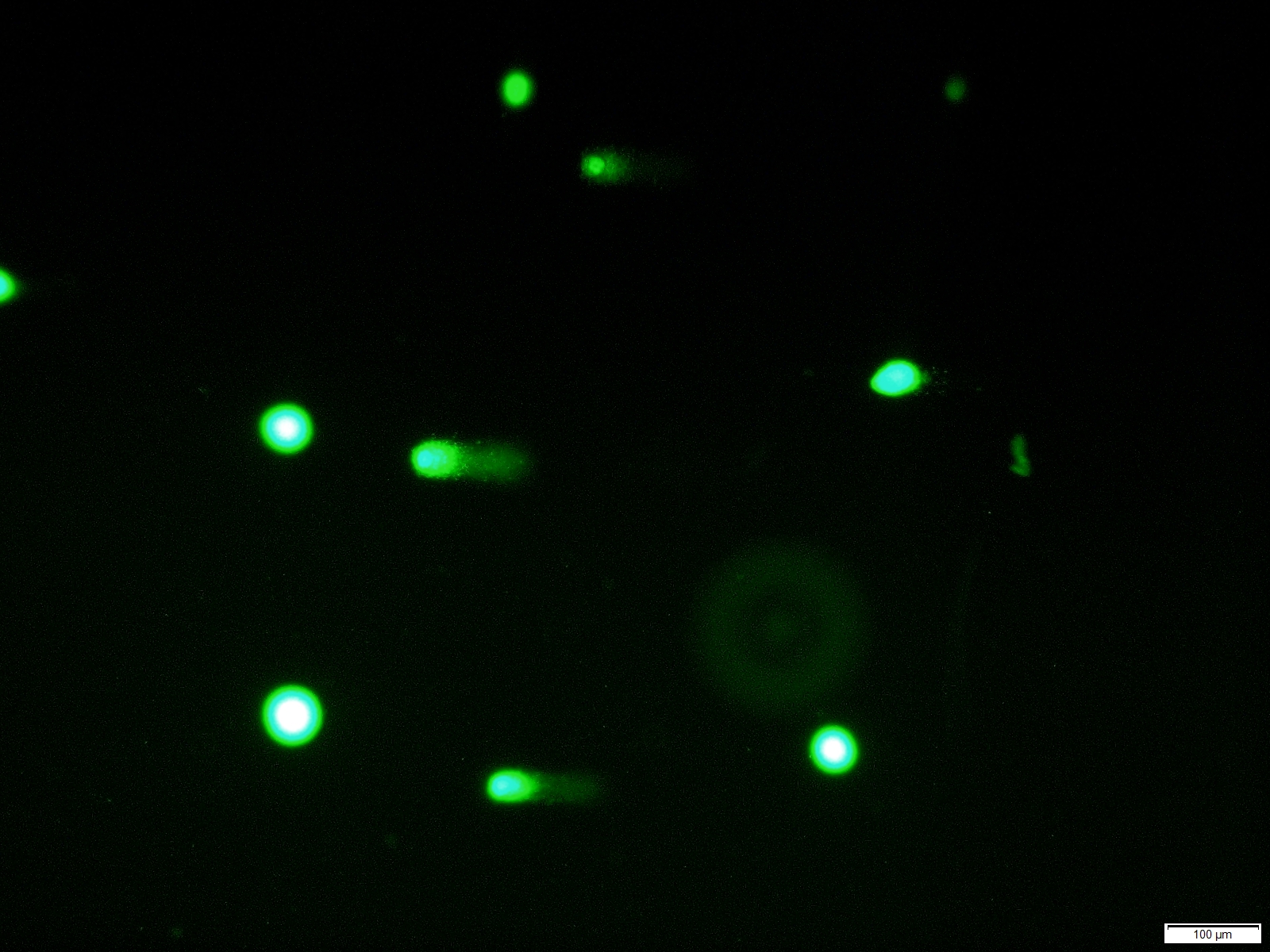

Supplement: Supplementary file 15 — Figure EV3 Source Data [file 44321_2026_414_MOESM15_ESM.zip › Fig. EV3/EV3A/OVISE shBMAL2#2.jpg]

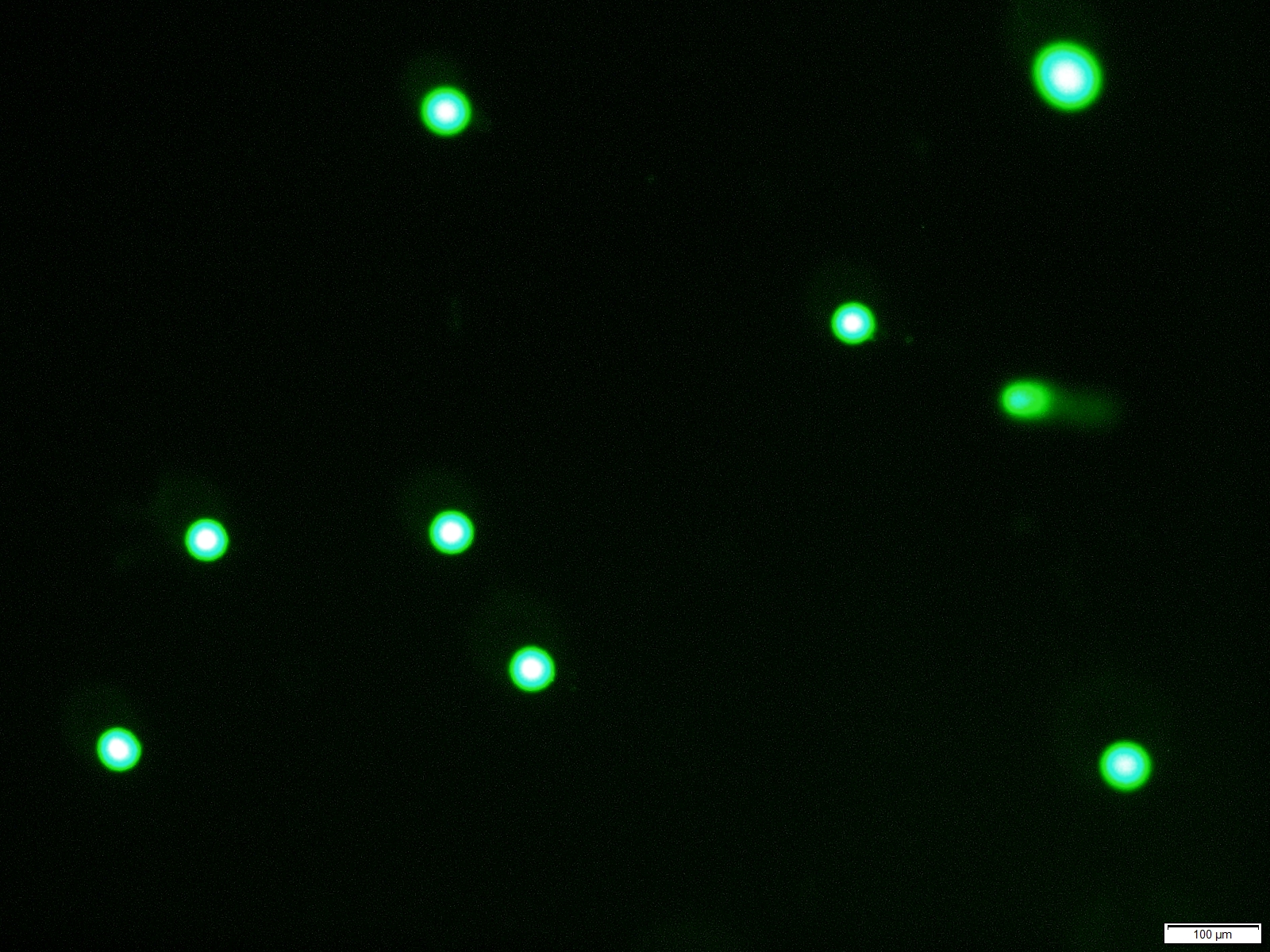

Supplement: Supplementary file 15 — Figure EV3 Source Data [file 44321_2026_414_MOESM15_ESM.zip › Fig. EV3/EV3A/OVISE shCtrl.jpg]

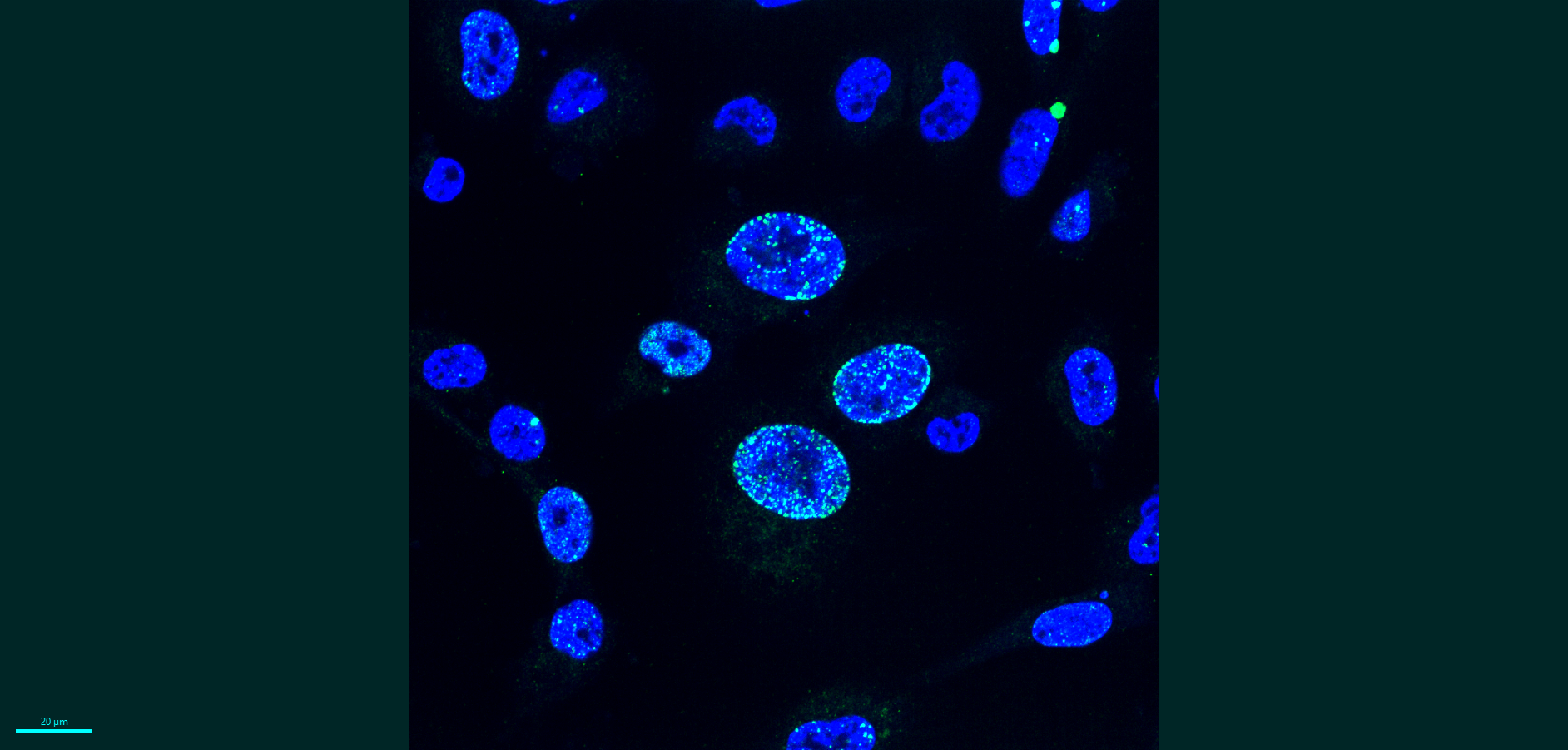

Supplement: Supplementary file 15 — Figure EV3 Source Data [file 44321_2026_414_MOESM15_ESM.zip › Fig. EV3/EV3B/ES-2 shBMAL2#1.png]

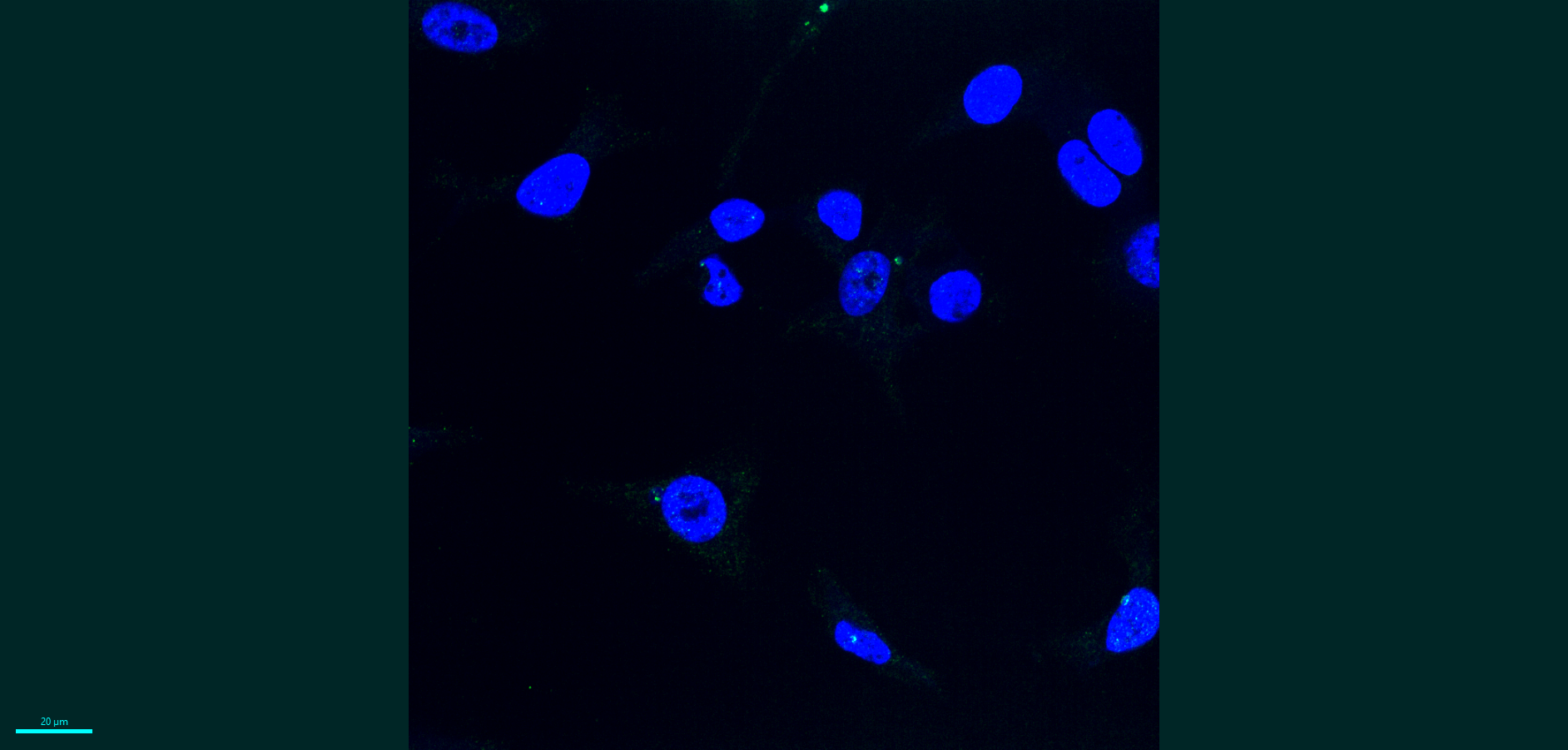

Supplement: Supplementary file 15 — Figure EV3 Source Data [file 44321_2026_414_MOESM15_ESM.zip › Fig. EV3/EV3B/ES-2 shBMAL2#2.png]

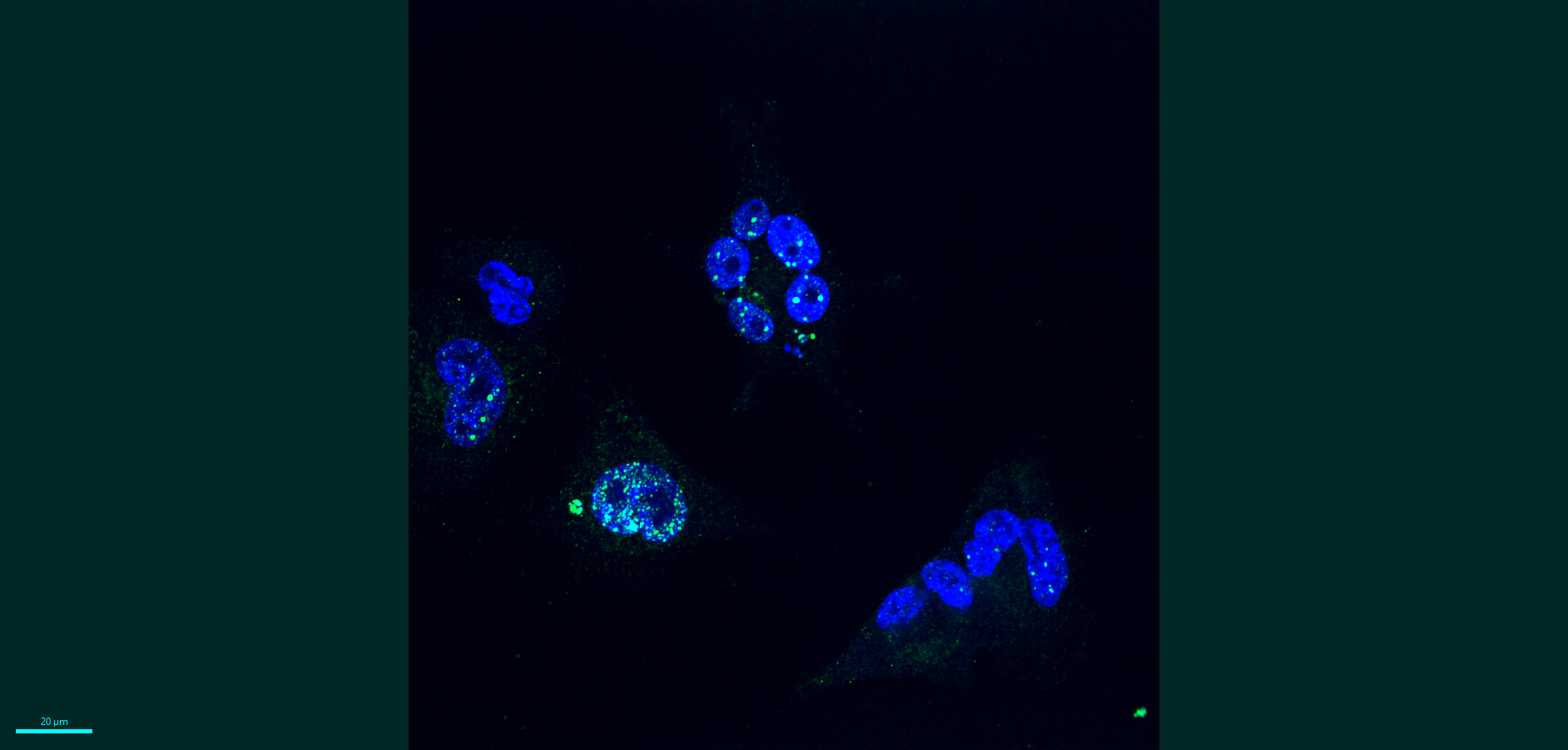

Supplement: Supplementary file 15 — Figure EV3 Source Data [file 44321_2026_414_MOESM15_ESM.zip › Fig. EV3/EV3B/ES2-326-63X-2z_merge.png]

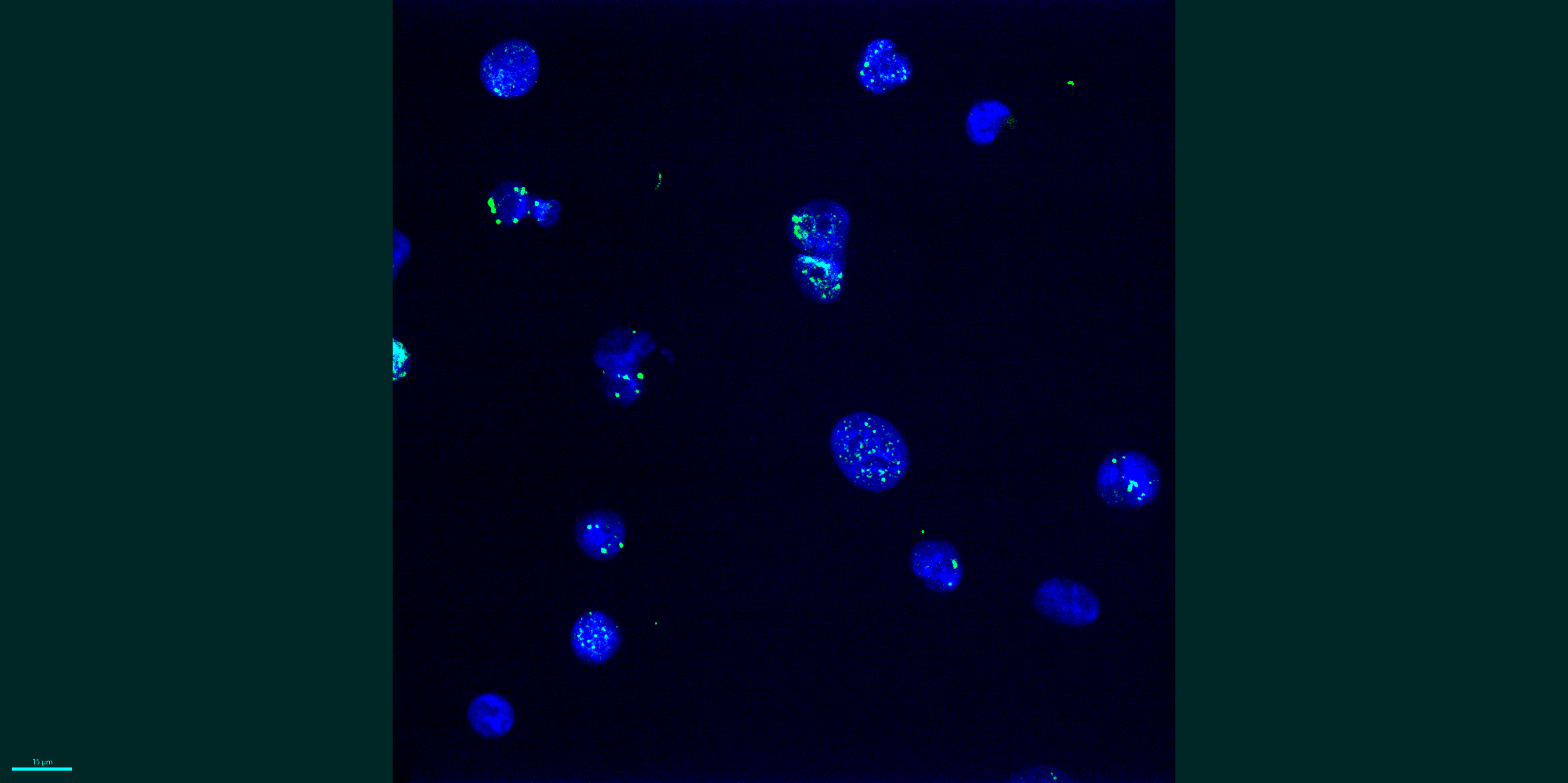

Supplement: Supplementary file 15 — Figure EV3 Source Data [file 44321_2026_414_MOESM15_ESM.zip › Fig. EV3/EV3B/JHOC5 shBMAL2#1.png]

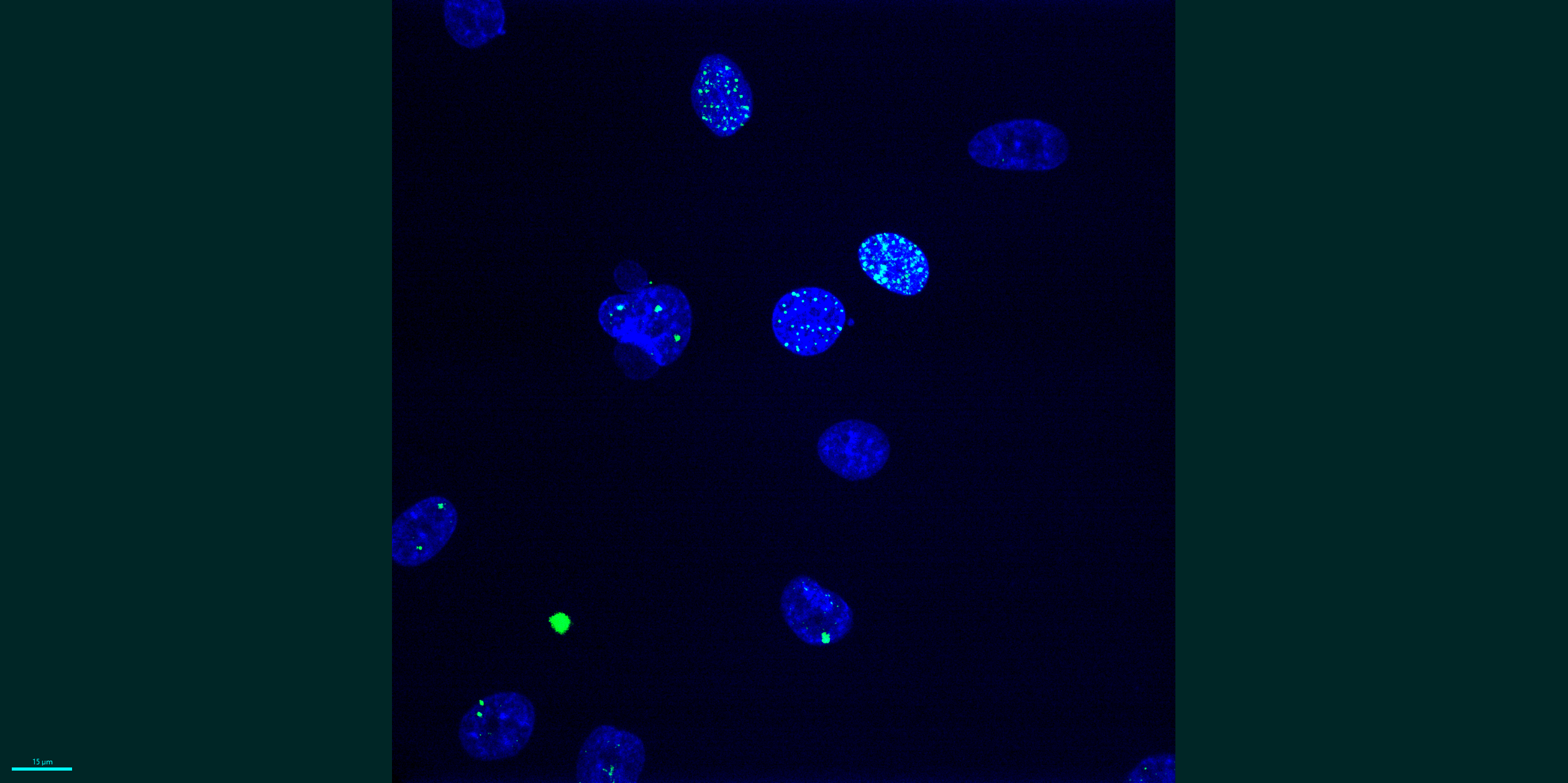

Supplement: Supplementary file 15 — Figure EV3 Source Data [file 44321_2026_414_MOESM15_ESM.zip › Fig. EV3/EV3B/JHOC5 shBMAL2#2.png]

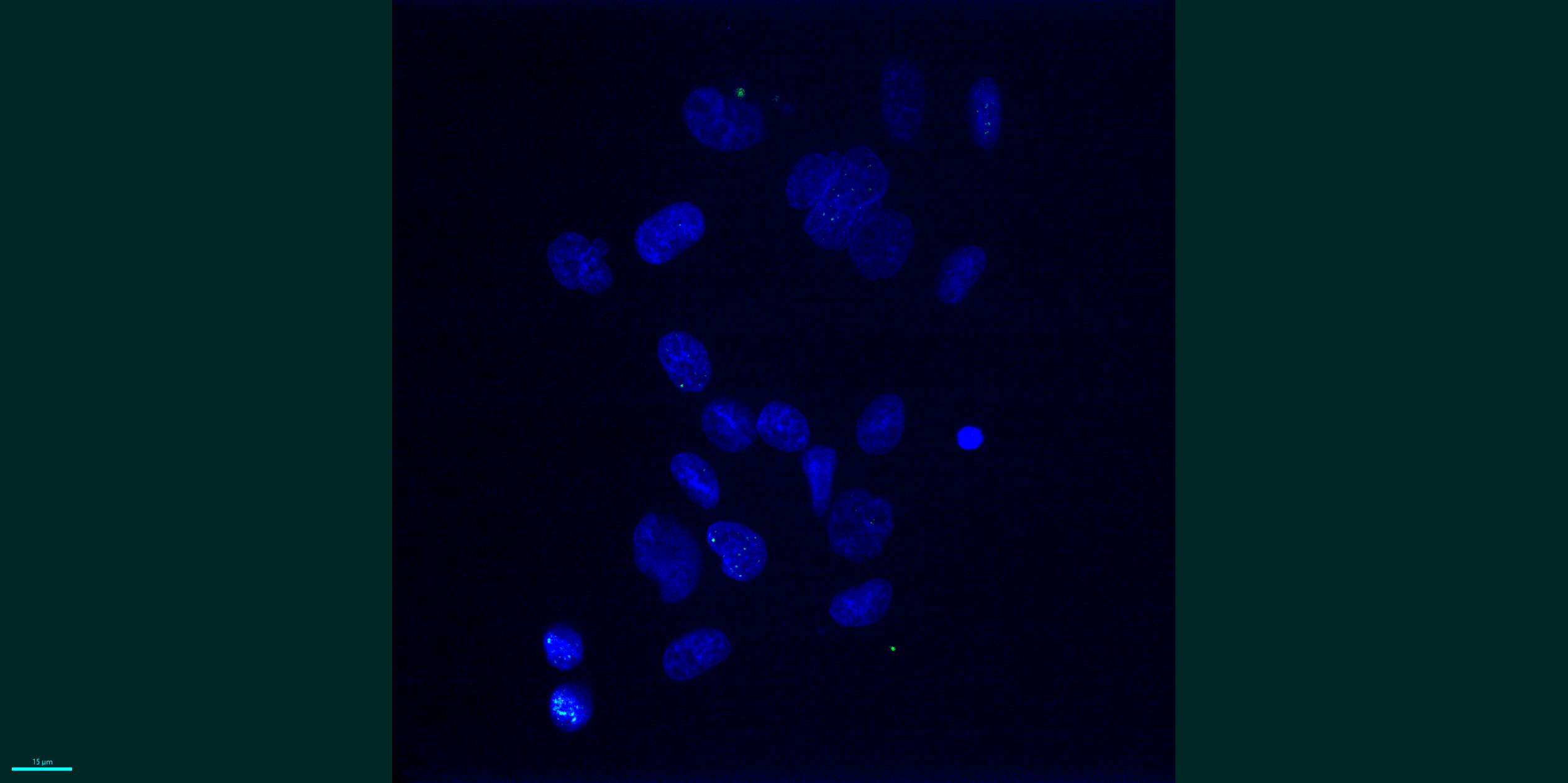

Supplement: Supplementary file 15 — Figure EV3 Source Data [file 44321_2026_414_MOESM15_ESM.zip › Fig. EV3/EV3B/JHOC5 shCtrl.png]

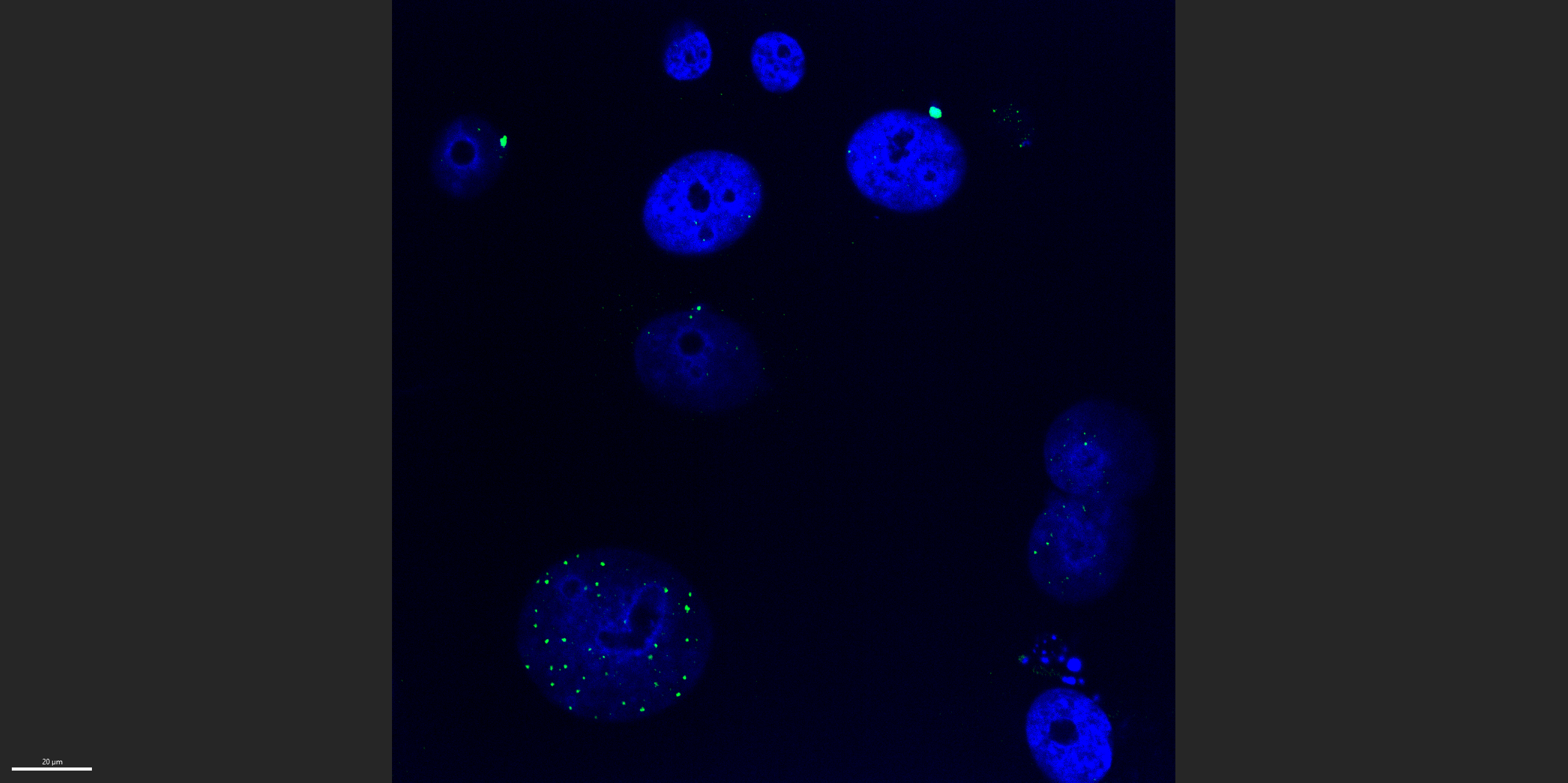

Supplement: Supplementary file 15 — Figure EV3 Source Data [file 44321_2026_414_MOESM15_ESM.zip › Fig. EV3/EV3B/JHOC9 shBMAL2#1.png]

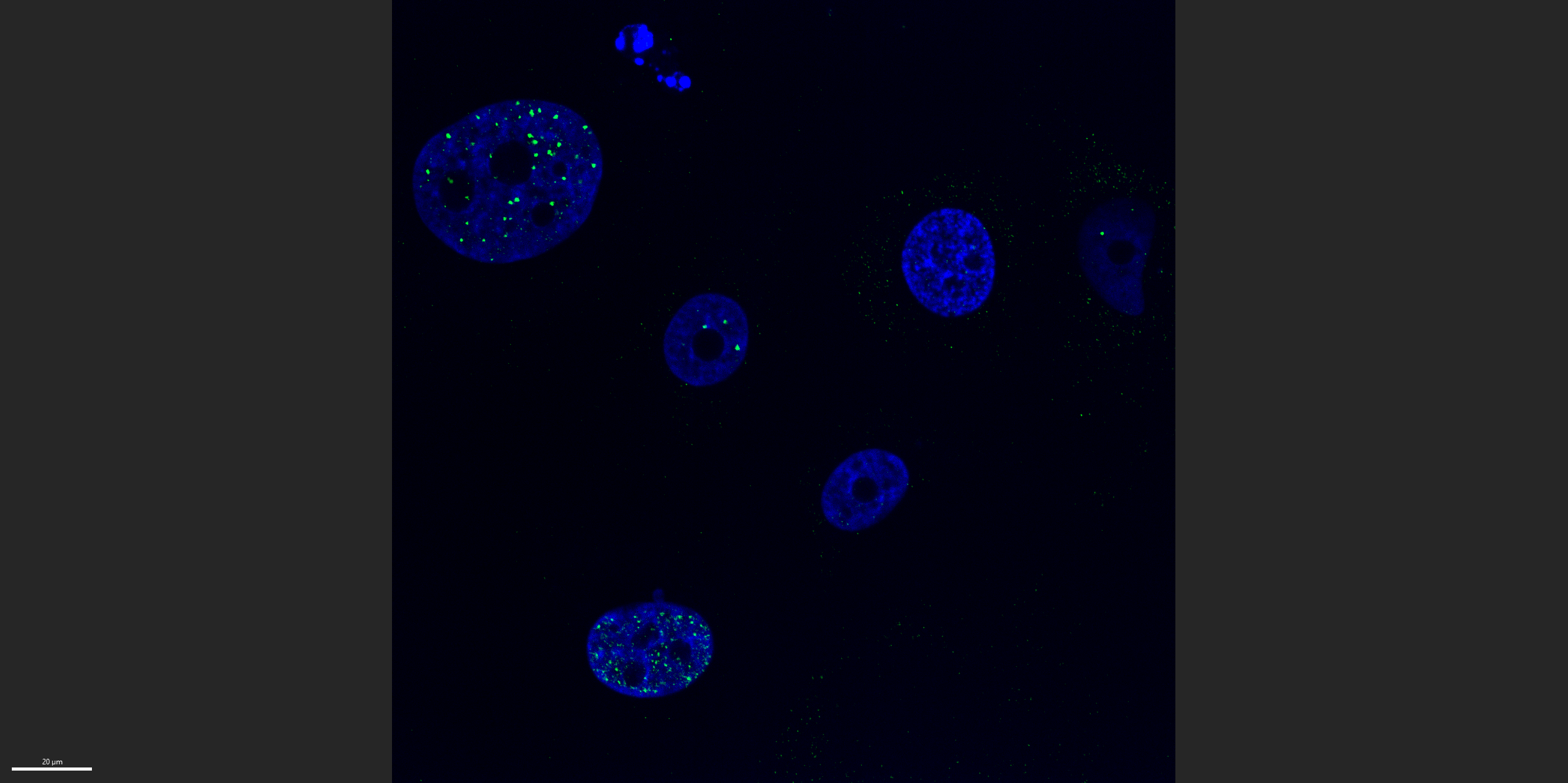

Supplement: Supplementary file 15 — Figure EV3 Source Data [file 44321_2026_414_MOESM15_ESM.zip › Fig. EV3/EV3B/JHOC9 shBMAL2#2.png]

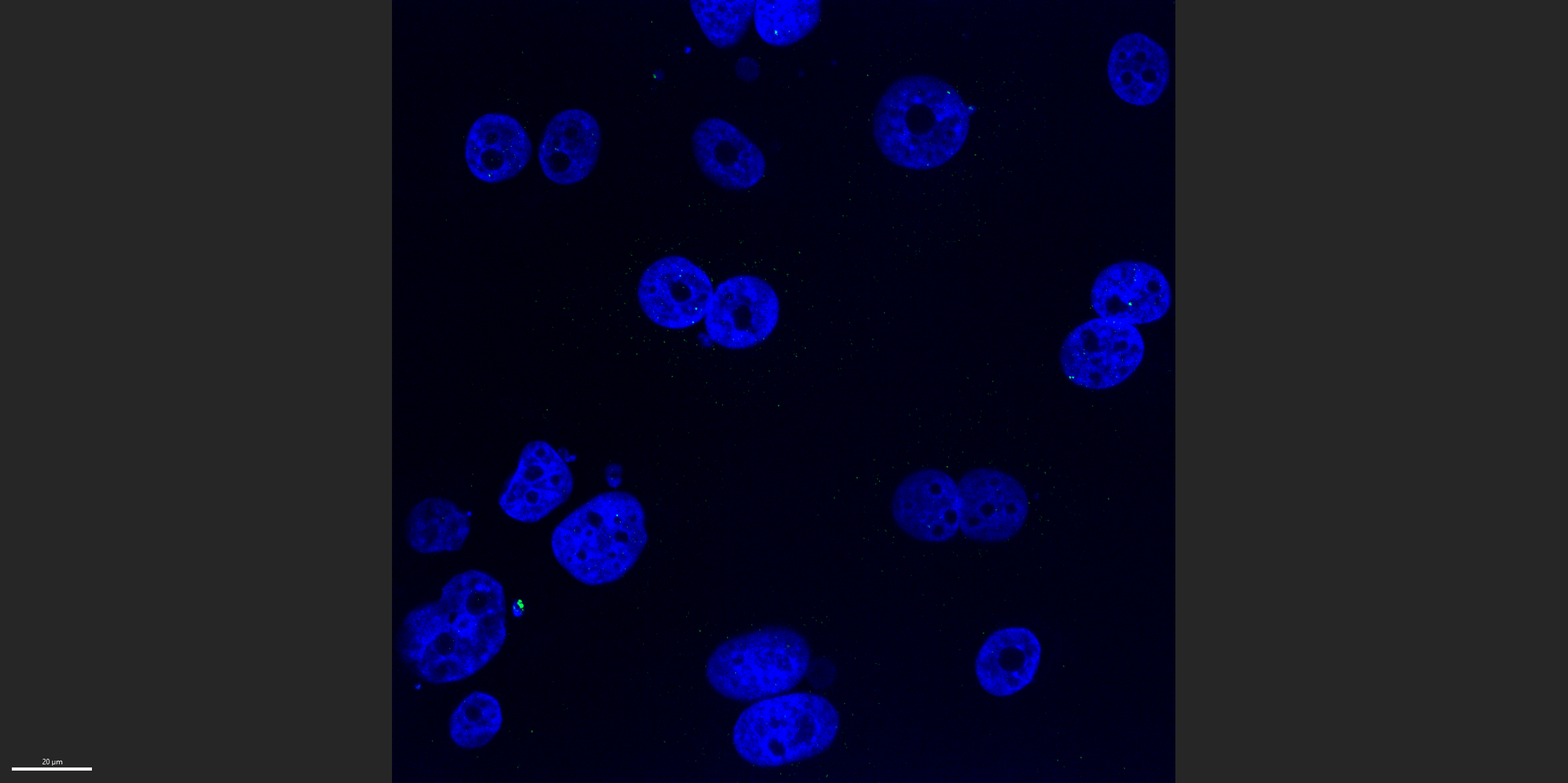

Supplement: Supplementary file 15 — Figure EV3 Source Data [file 44321_2026_414_MOESM15_ESM.zip › Fig. EV3/EV3B/JHOC9 shCtrl.png]

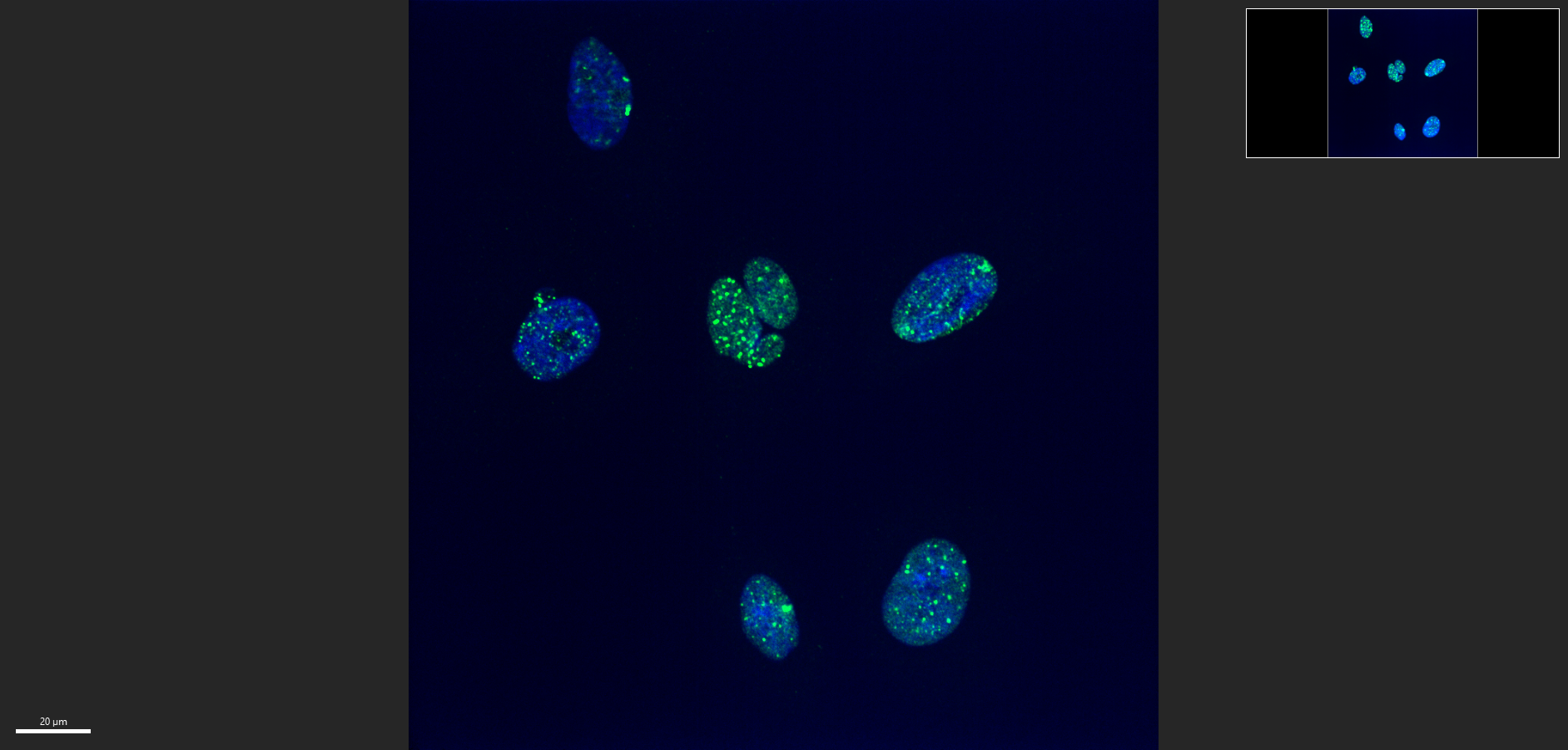

Supplement: Supplementary file 15 — Figure EV3 Source Data [file 44321_2026_414_MOESM15_ESM.zip › Fig. EV3/EV3B/OVCA429 shBMAL2#1.png]

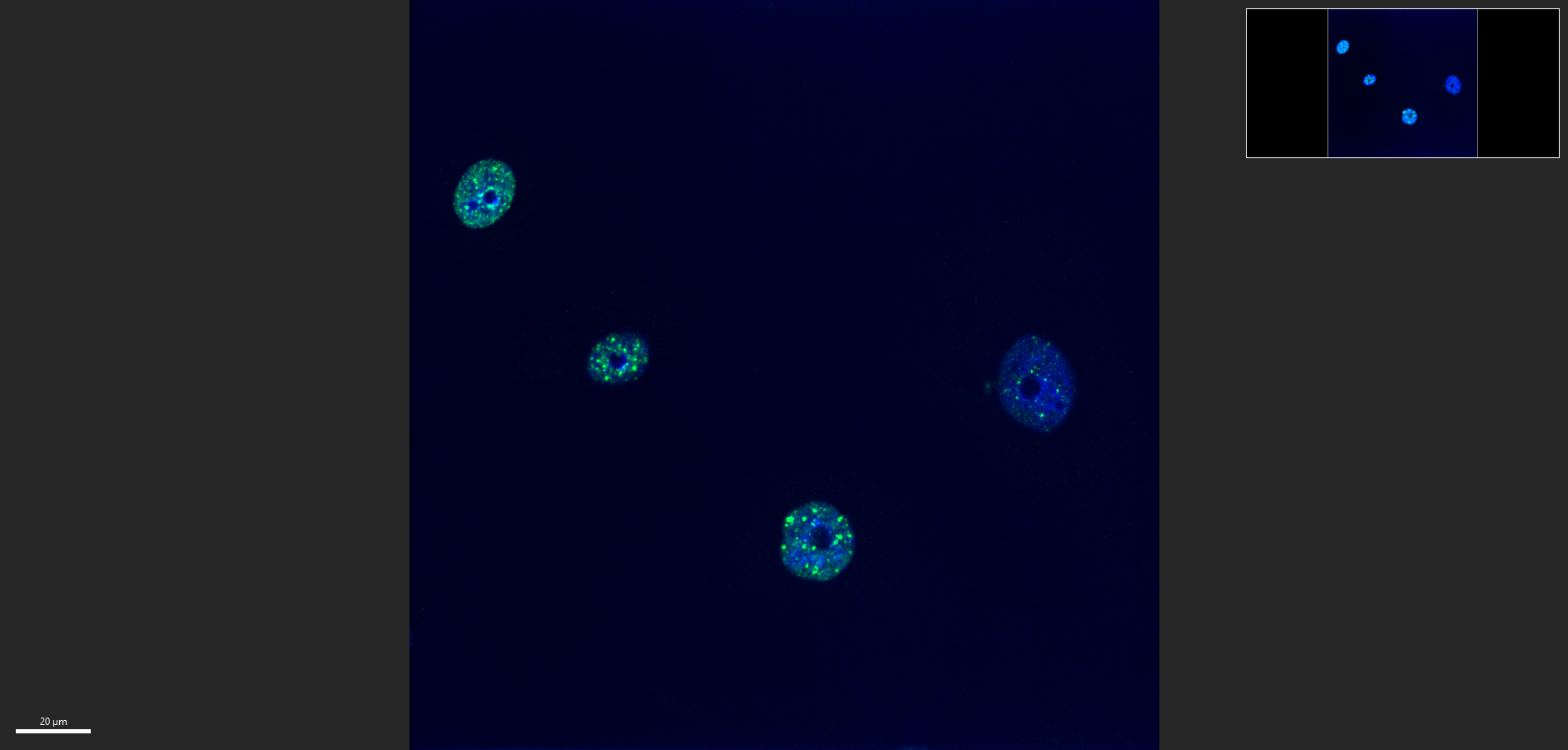

Supplement: Supplementary file 15 — Figure EV3 Source Data [file 44321_2026_414_MOESM15_ESM.zip › Fig. EV3/EV3B/OVCA429 shBMAL2#2.png]

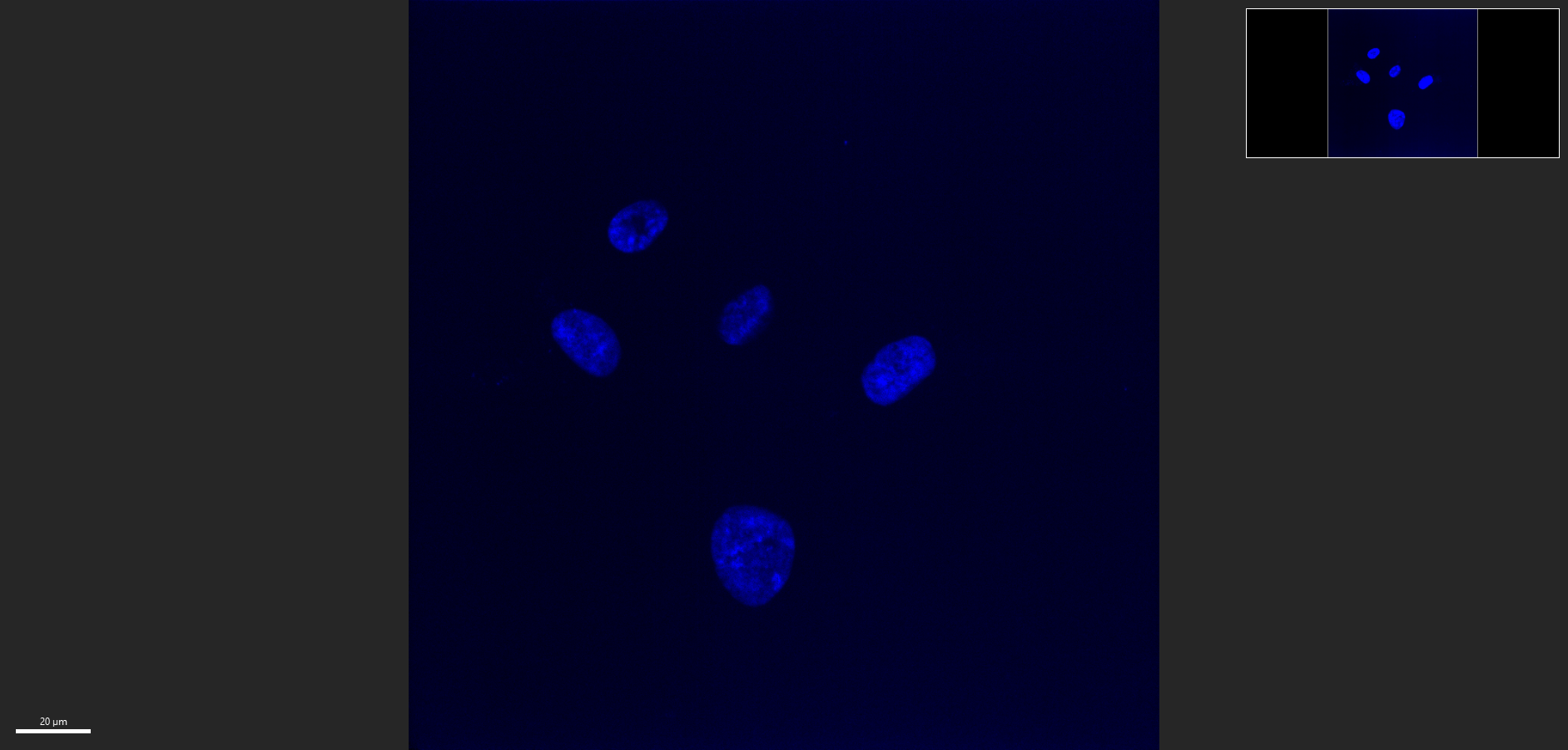

Supplement: Supplementary file 15 — Figure EV3 Source Data [file 44321_2026_414_MOESM15_ESM.zip › Fig. EV3/EV3B/OVCA429 shCtrl.png]

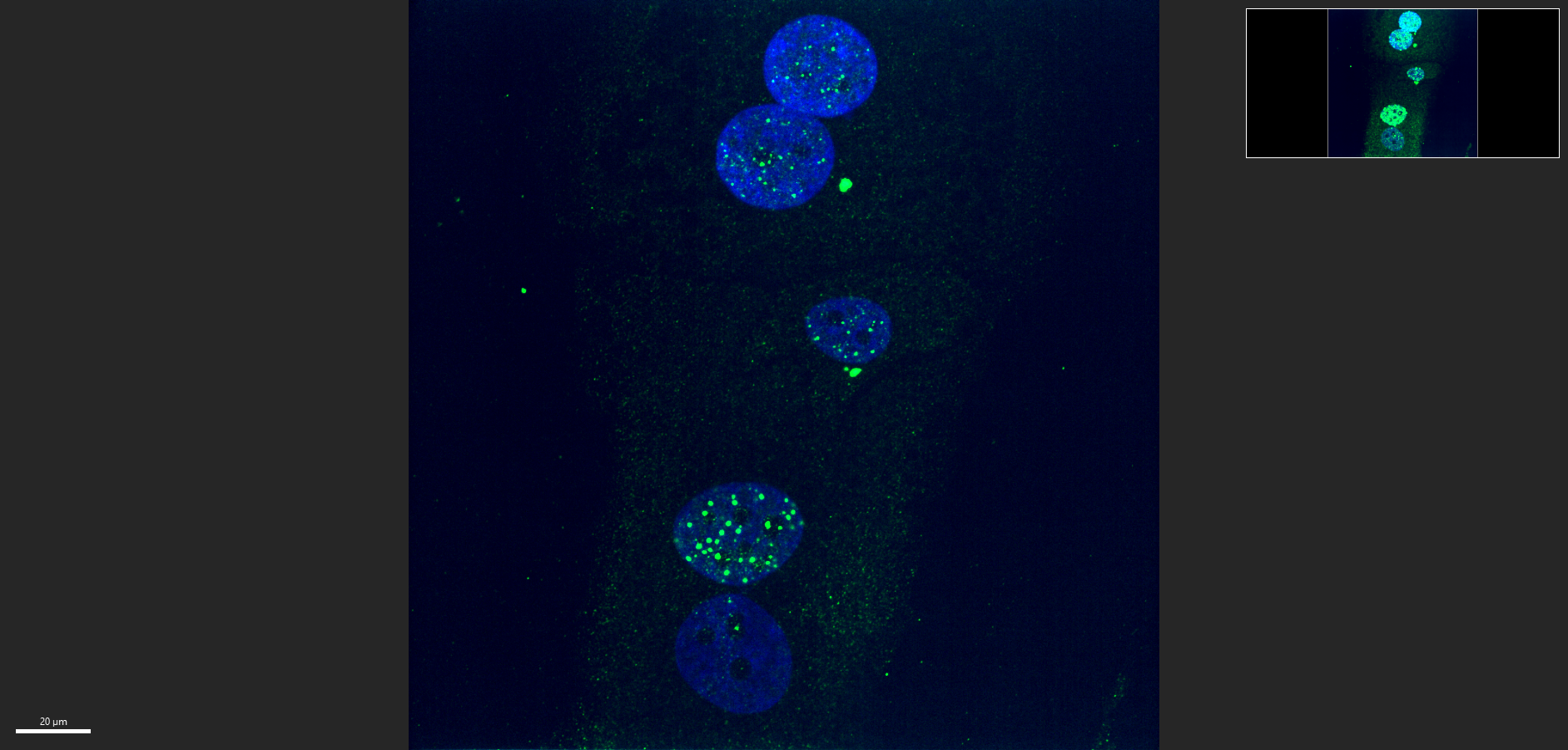

Supplement: Supplementary file 15 — Figure EV3 Source Data [file 44321_2026_414_MOESM15_ESM.zip › Fig. EV3/EV3B/OVISE shBMAL2#1.png]

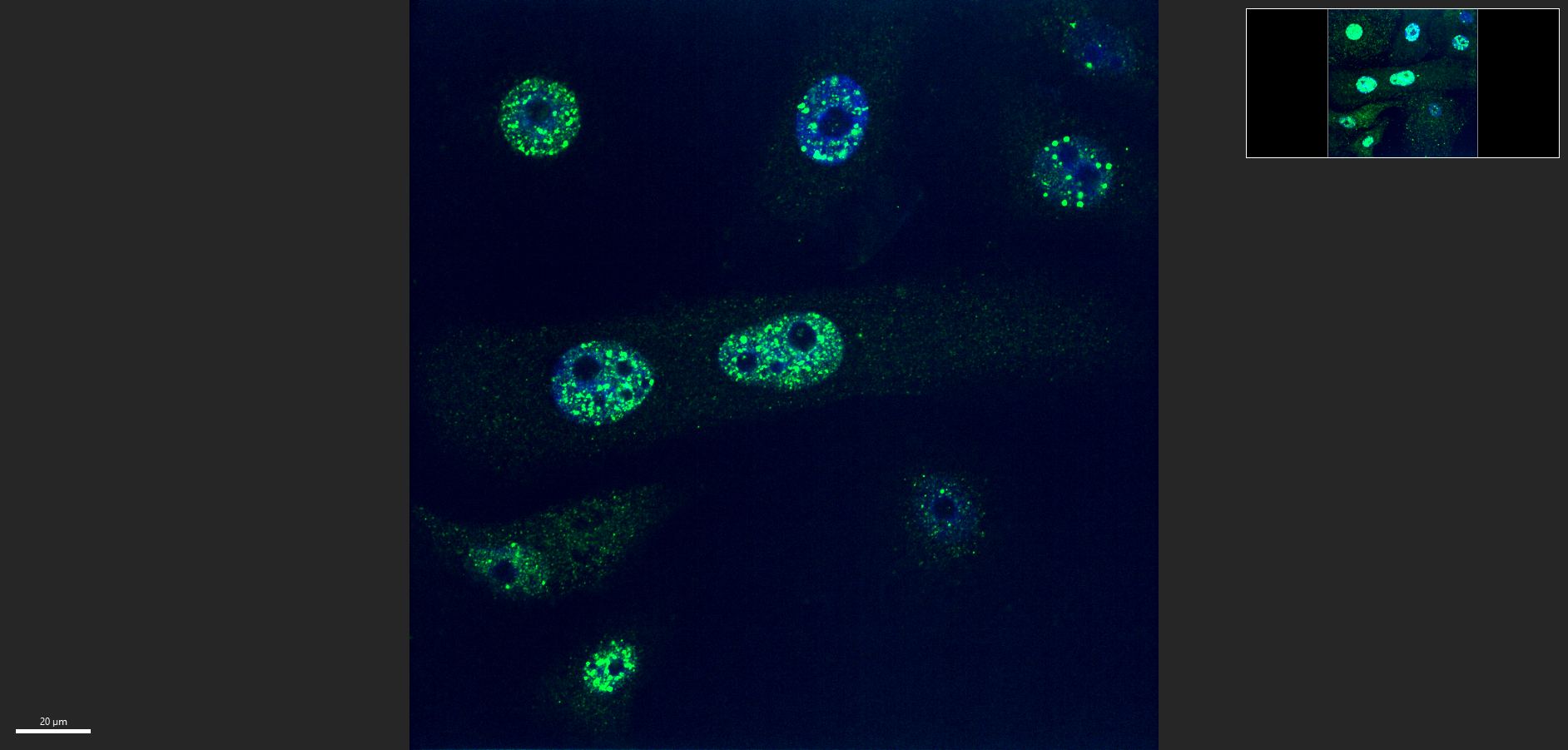

Supplement: Supplementary file 15 — Figure EV3 Source Data [file 44321_2026_414_MOESM15_ESM.zip › Fig. EV3/EV3B/OVISE shBMAL2#2.png]

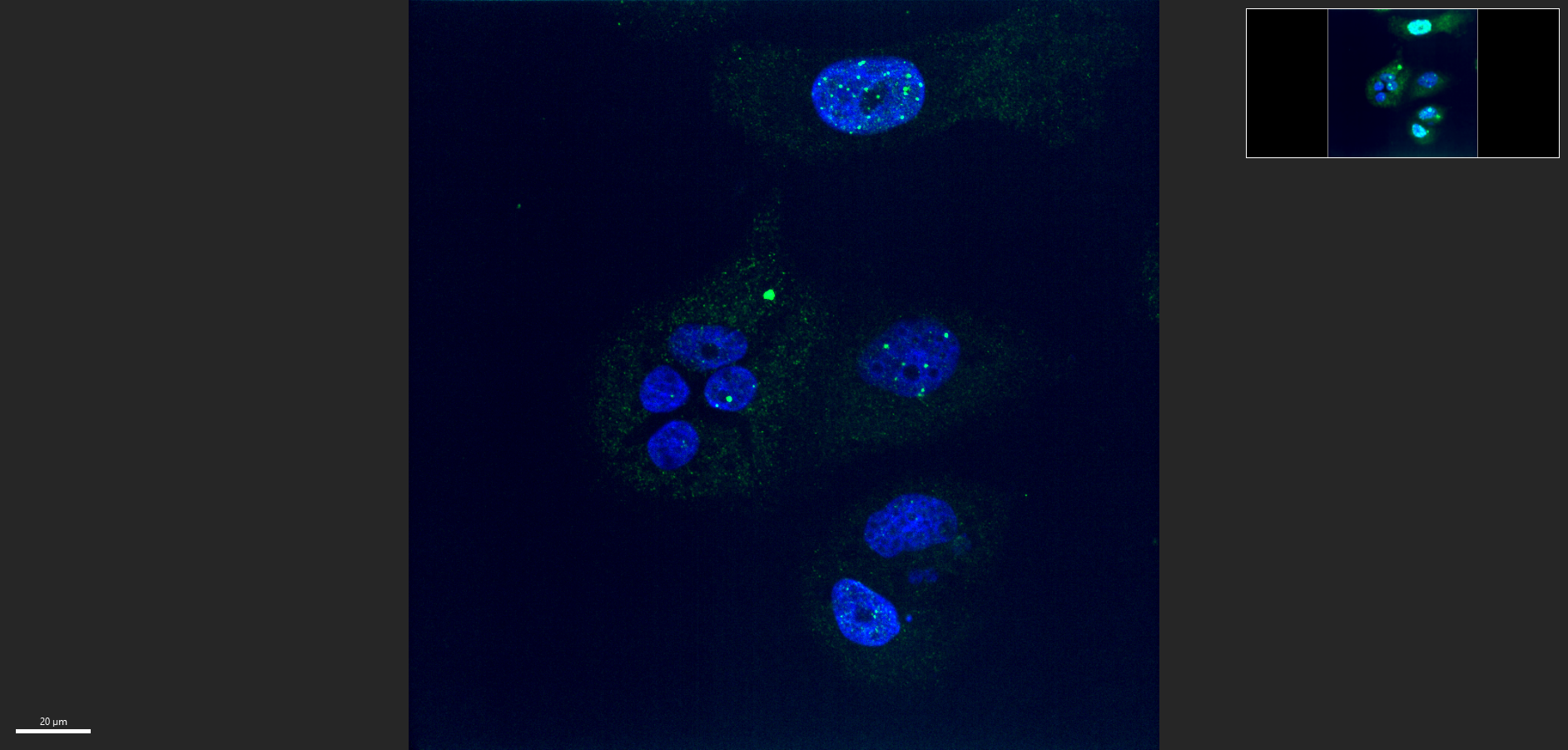

Supplement: Supplementary file 15 — Figure EV3 Source Data [file 44321_2026_414_MOESM15_ESM.zip › Fig. EV3/EV3B/OVISE shCtrl.png]

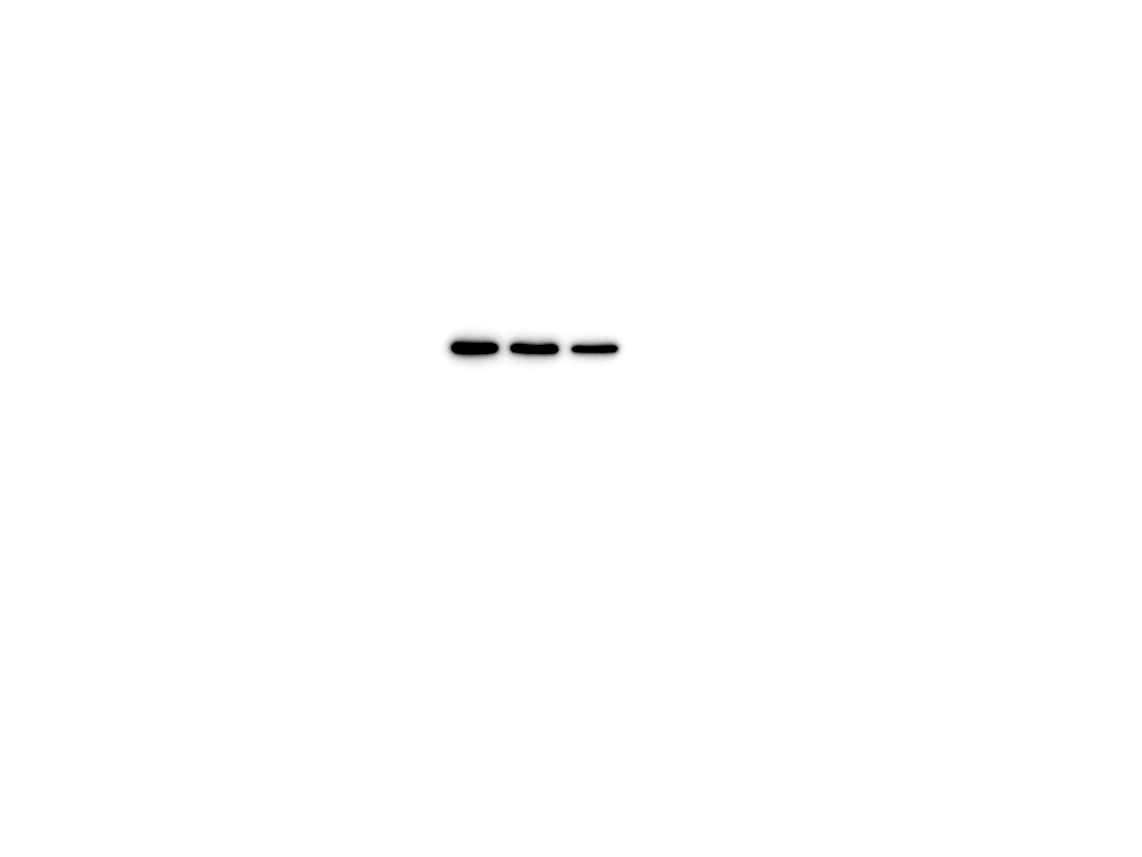

Supplement: Supplementary file 15 — Figure EV3 Source Data [file 44321_2026_414_MOESM15_ESM.zip › Fig. EV3/EV3C/ES-2 p84 IB.tif]

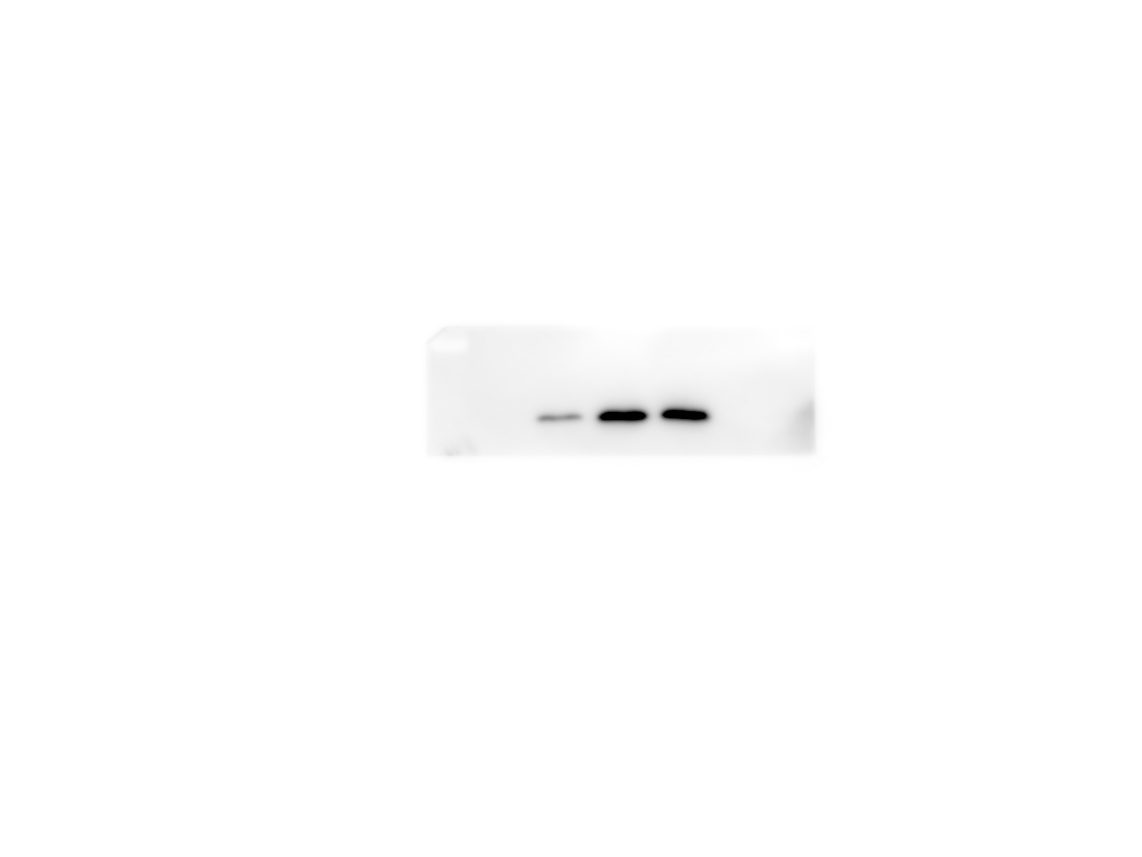

Supplement: Supplementary file 15 — Figure EV3 Source Data [file 44321_2026_414_MOESM15_ESM.zip › Fig. EV3/EV3C/ES-2 yH2ax IB.tif]

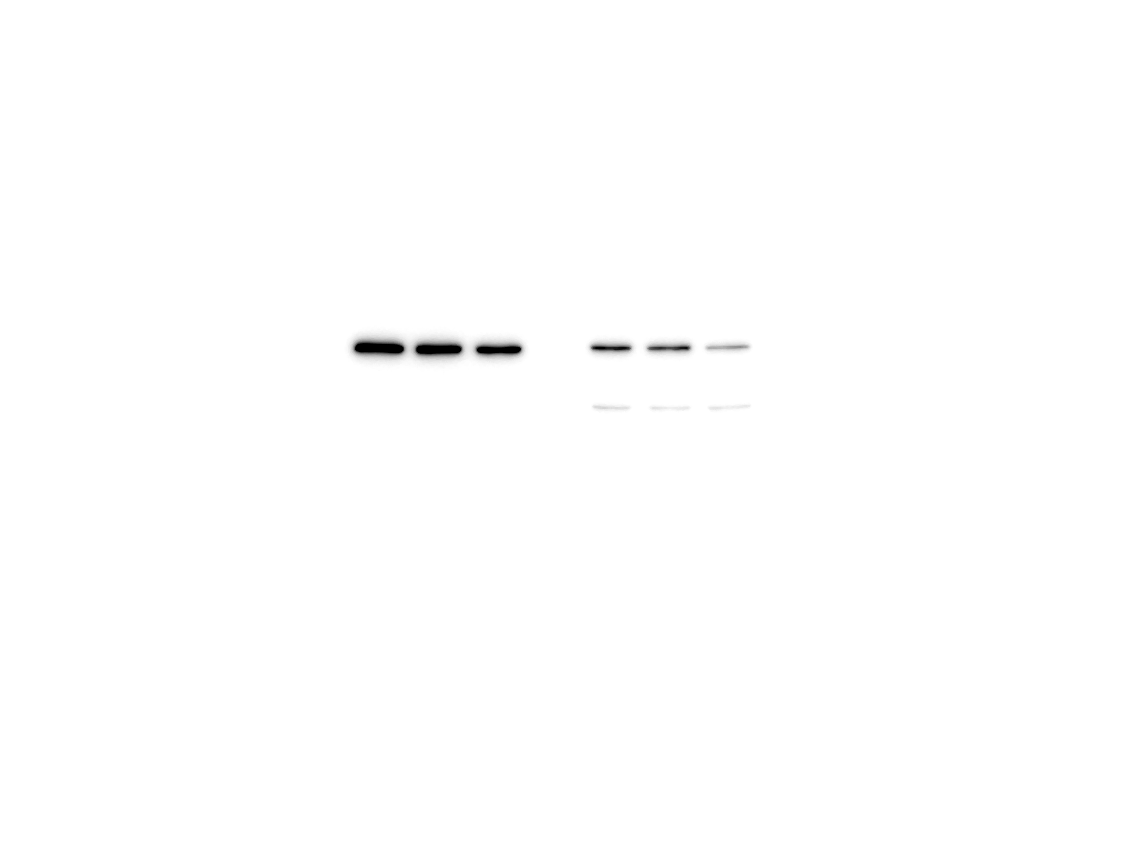

Supplement: Supplementary file 15 — Figure EV3 Source Data [file 44321_2026_414_MOESM15_ESM.zip › Fig. EV3/EV3C/JHOC5 p84 IB.tif]

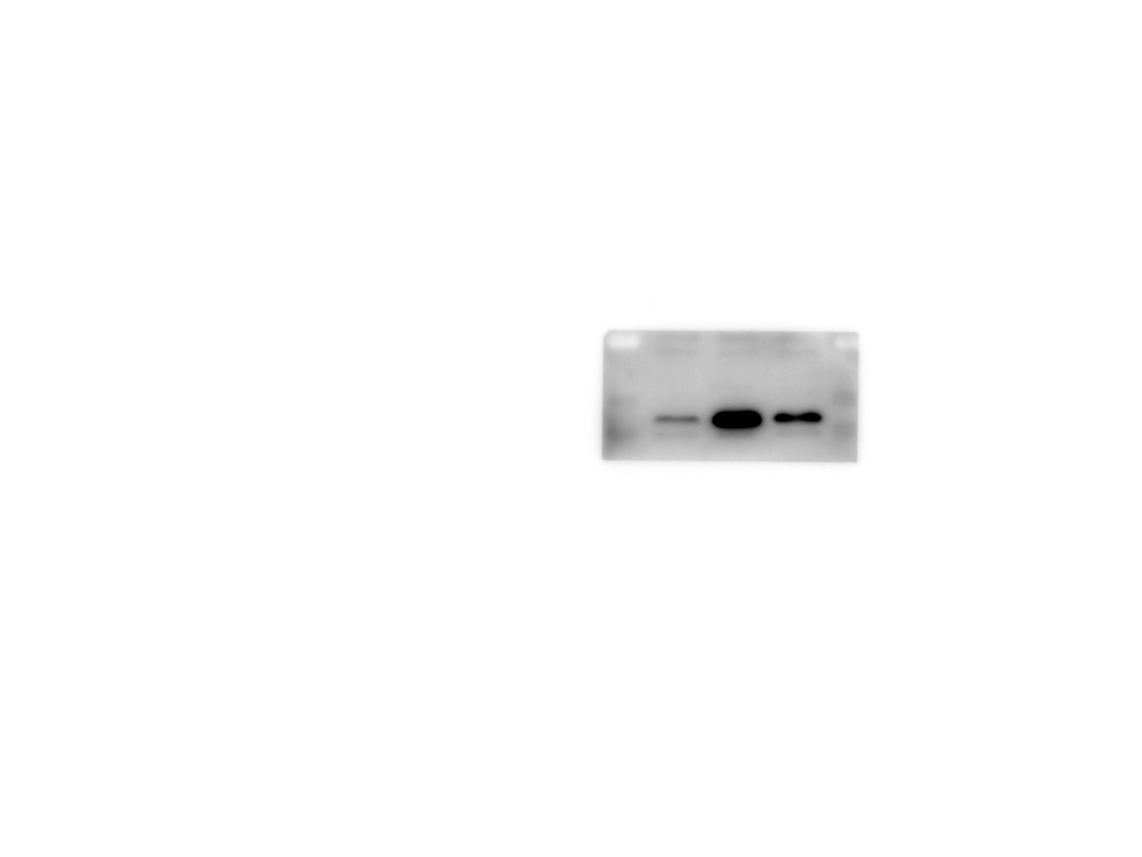

Supplement: Supplementary file 15 — Figure EV3 Source Data [file 44321_2026_414_MOESM15_ESM.zip › Fig. EV3/EV3C/JHOC5 yH2ax IB.tif]

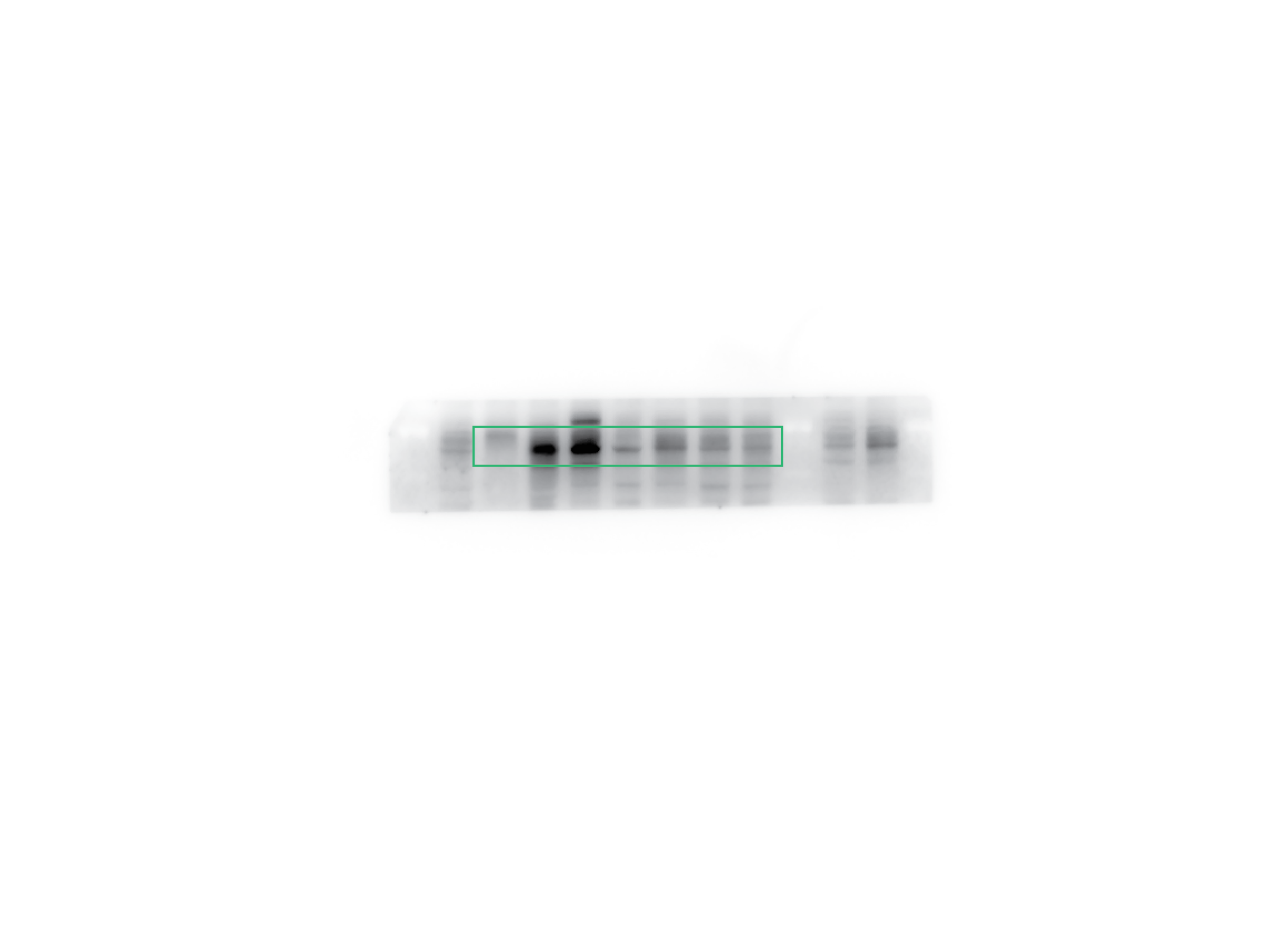

Supplement: Supplementary file 15 — Figure EV3 Source Data [file 44321_2026_414_MOESM15_ESM.zip › Fig. EV3/EV3D/BMAL2 IB.png]
