# Supplementary figures and images for: BMAL2 is a druggable target for ovarian clear cell carcinoma (OCCC) (part 3 of 3)
Source: EMBO Mol Med. 2026 Apr 3;18(5):1933–66. doi: 10.1038/s44321-026-00414-8 (PMC13179388; doi:10.1038/s44321-026-00414-8)

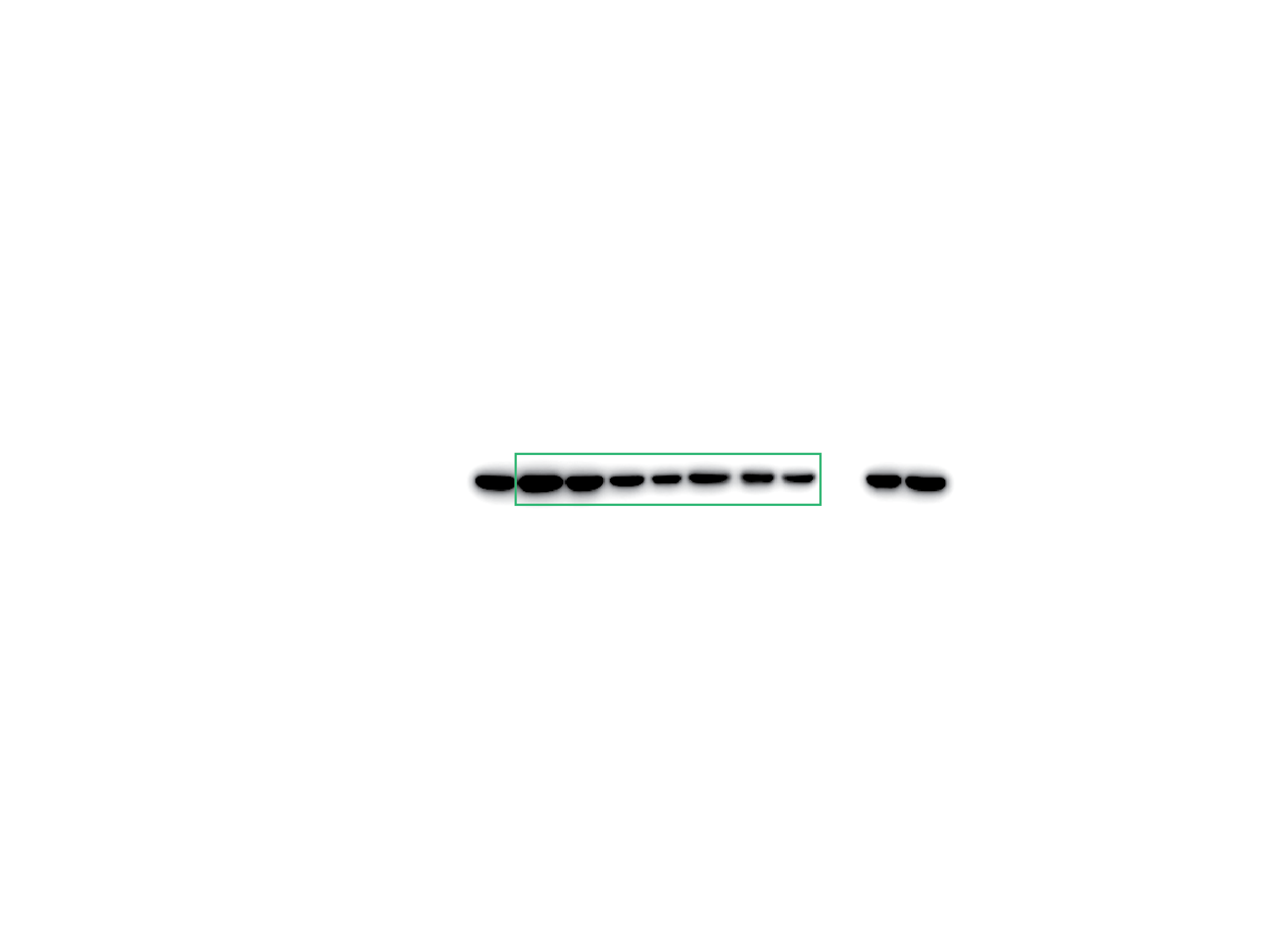

Supplement: Supplementary file 15 — Figure EV3 Source Data [file 44321_2026_414_MOESM15_ESM.zip › Fig. EV3/EV3D/GAPDH IB.png]

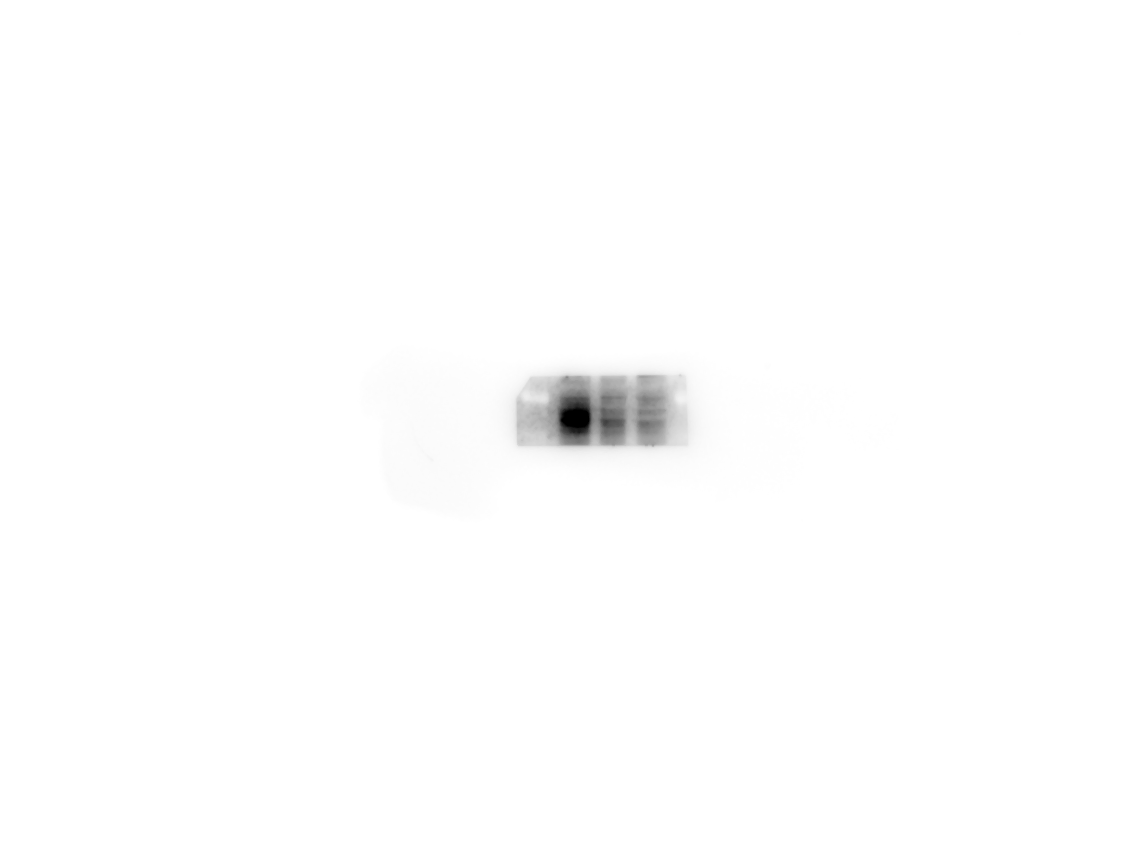

Supplement: Supplementary file 15 — Figure EV3 Source Data [file 44321_2026_414_MOESM15_ESM.zip › Fig. EV3/EV3E/HEYA8 BMAL2 IB.tif]

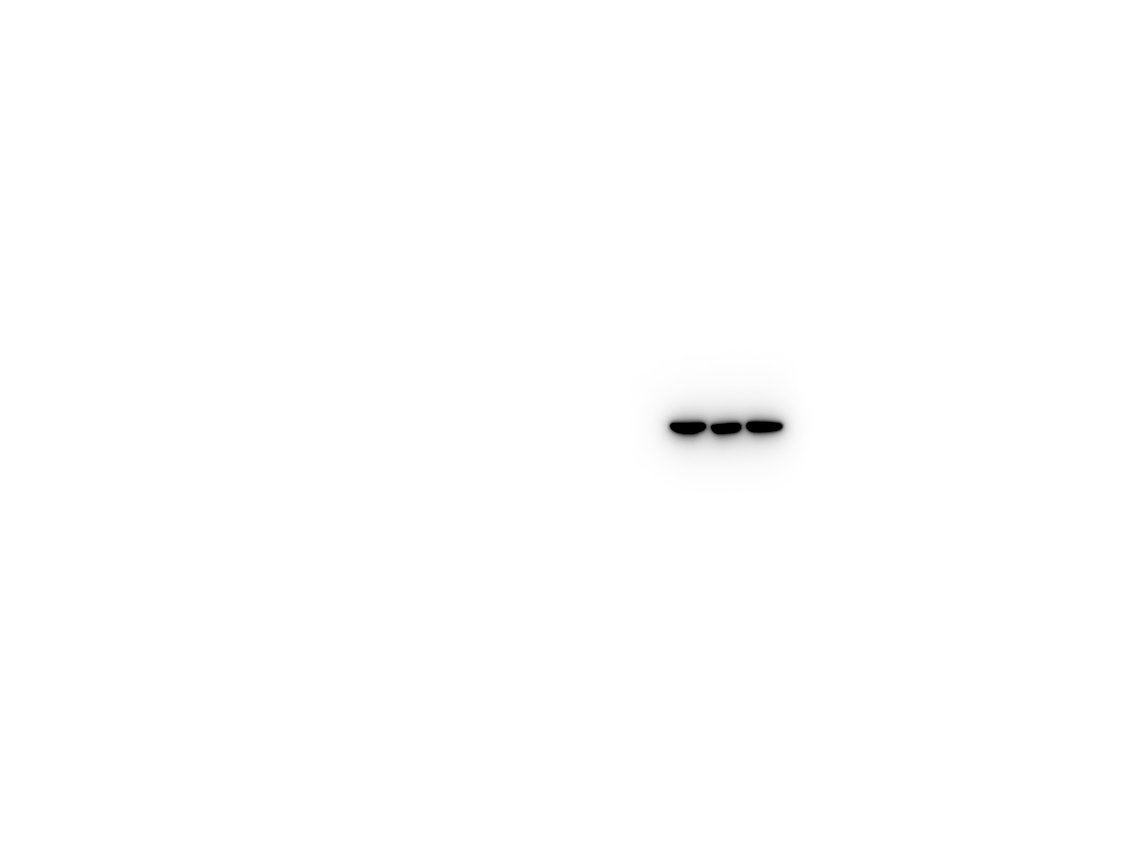

Supplement: Supplementary file 15 — Figure EV3 Source Data [file 44321_2026_414_MOESM15_ESM.zip › Fig. EV3/EV3E/HEYA8 GAPDH IB.tif]

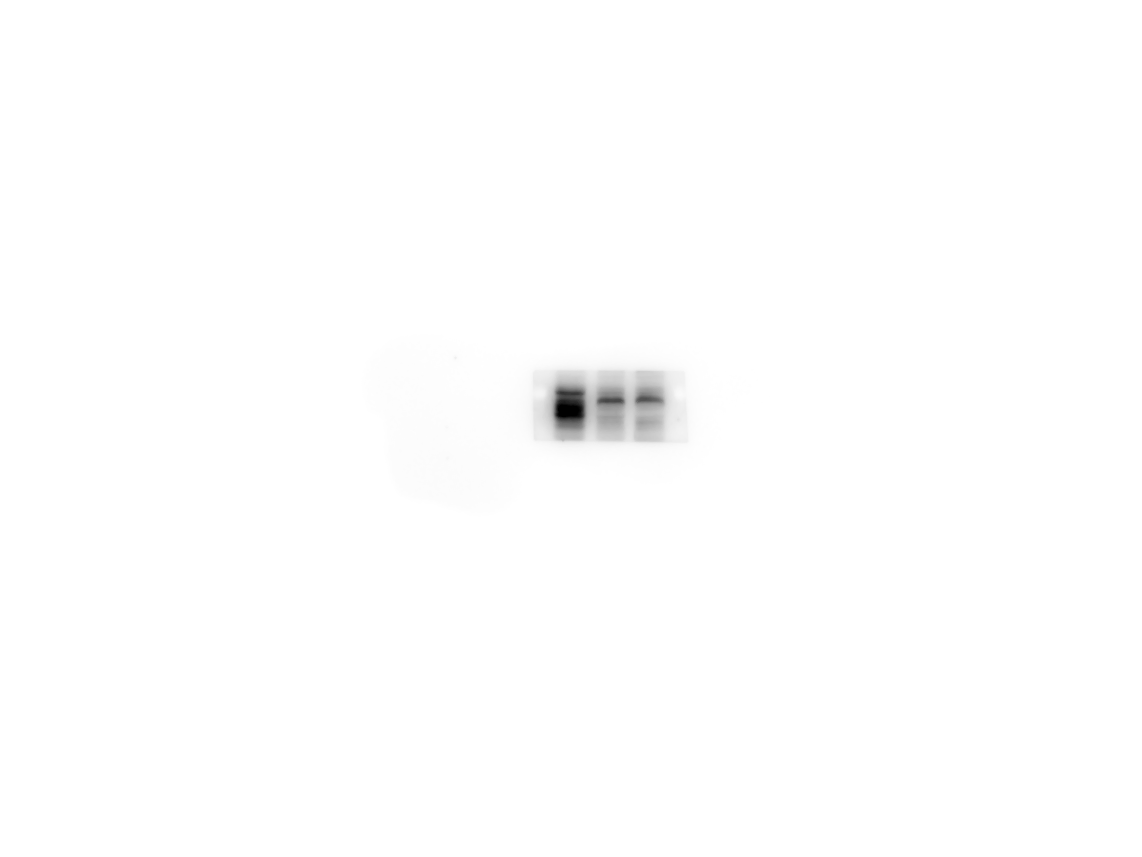

Supplement: Supplementary file 15 — Figure EV3 Source Data [file 44321_2026_414_MOESM15_ESM.zip › Fig. EV3/EV3E/KURAMOCHI BMAL2 IB.tif]

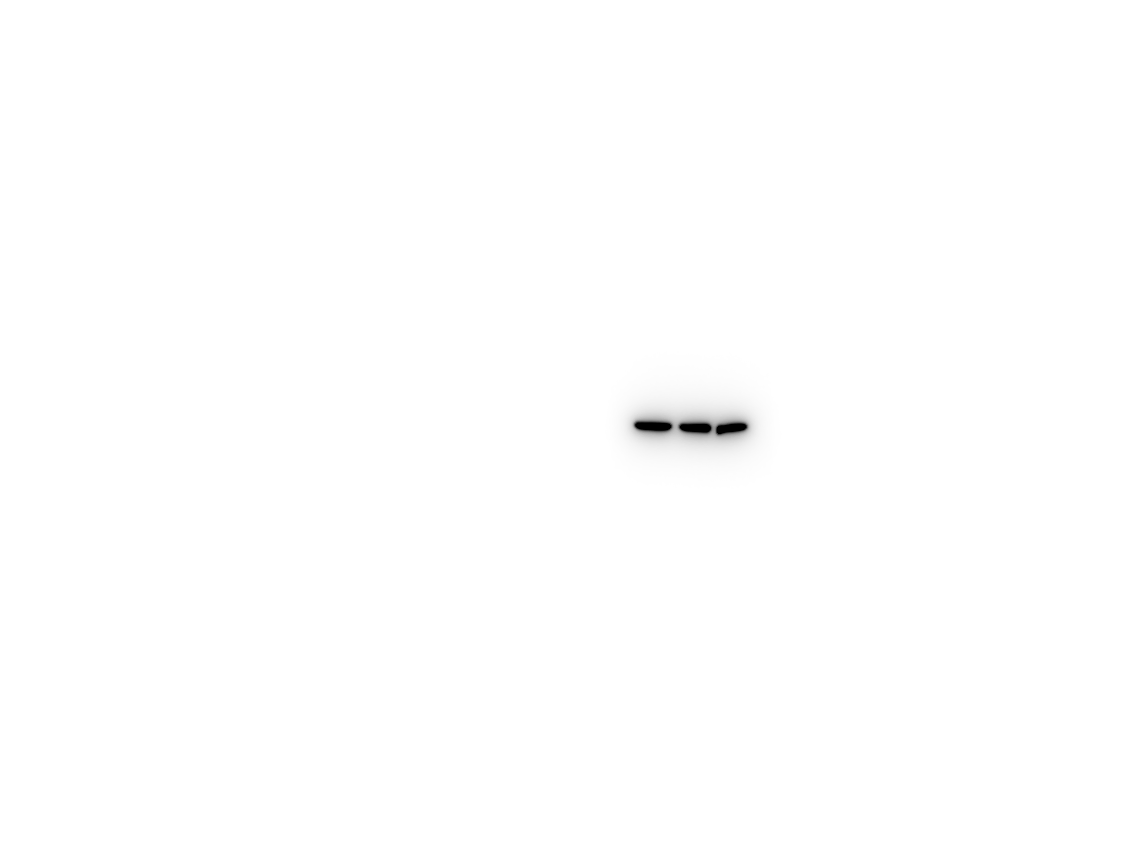

Supplement: Supplementary file 15 — Figure EV3 Source Data [file 44321_2026_414_MOESM15_ESM.zip › Fig. EV3/EV3E/KURAMOCHI GAPDH IB.tif]

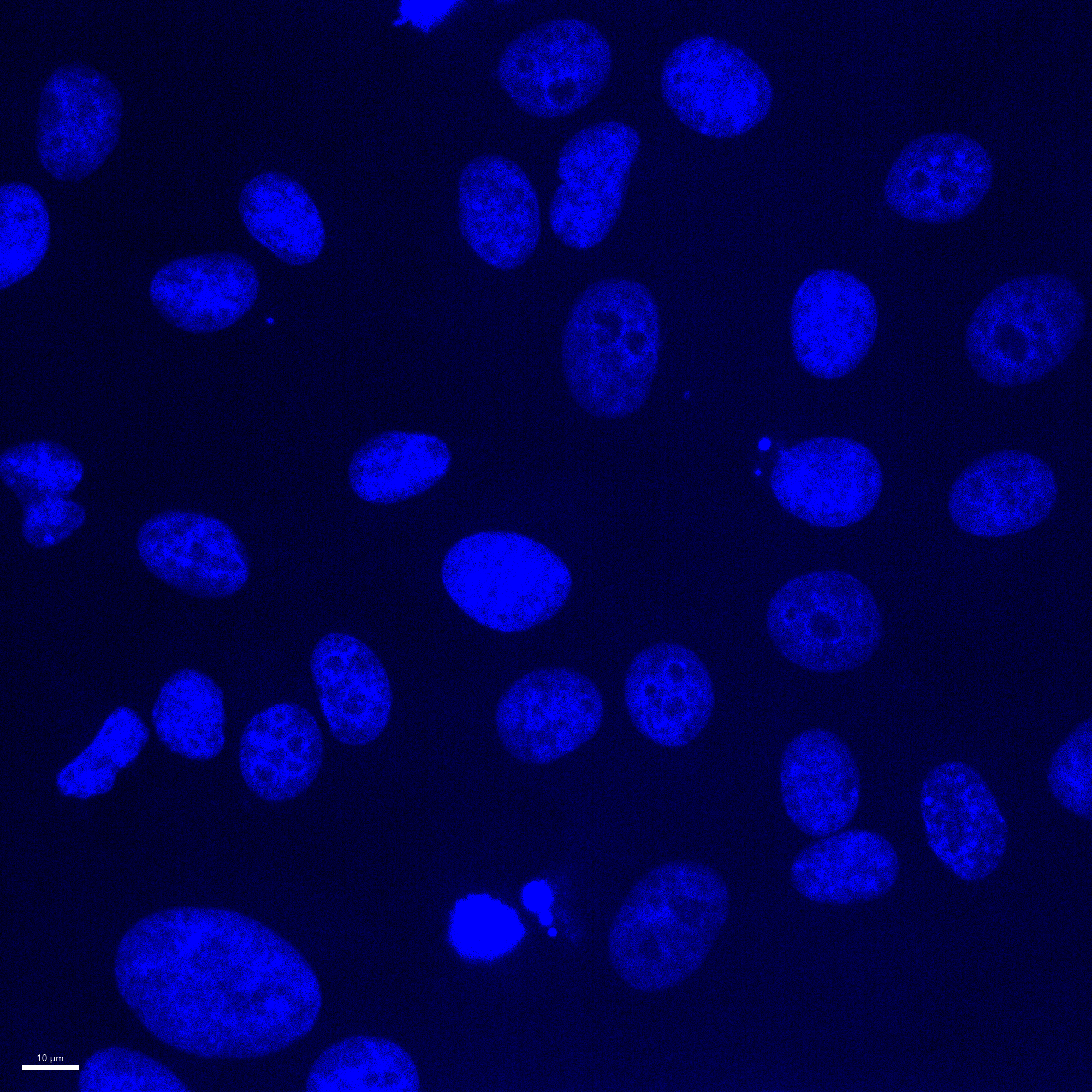

Supplement: Supplementary file 15 — Figure EV3 Source Data [file 44321_2026_414_MOESM15_ESM.zip › Fig. EV3/EV3F/HeyA8 shBMAL2#1 DAPI.tif]

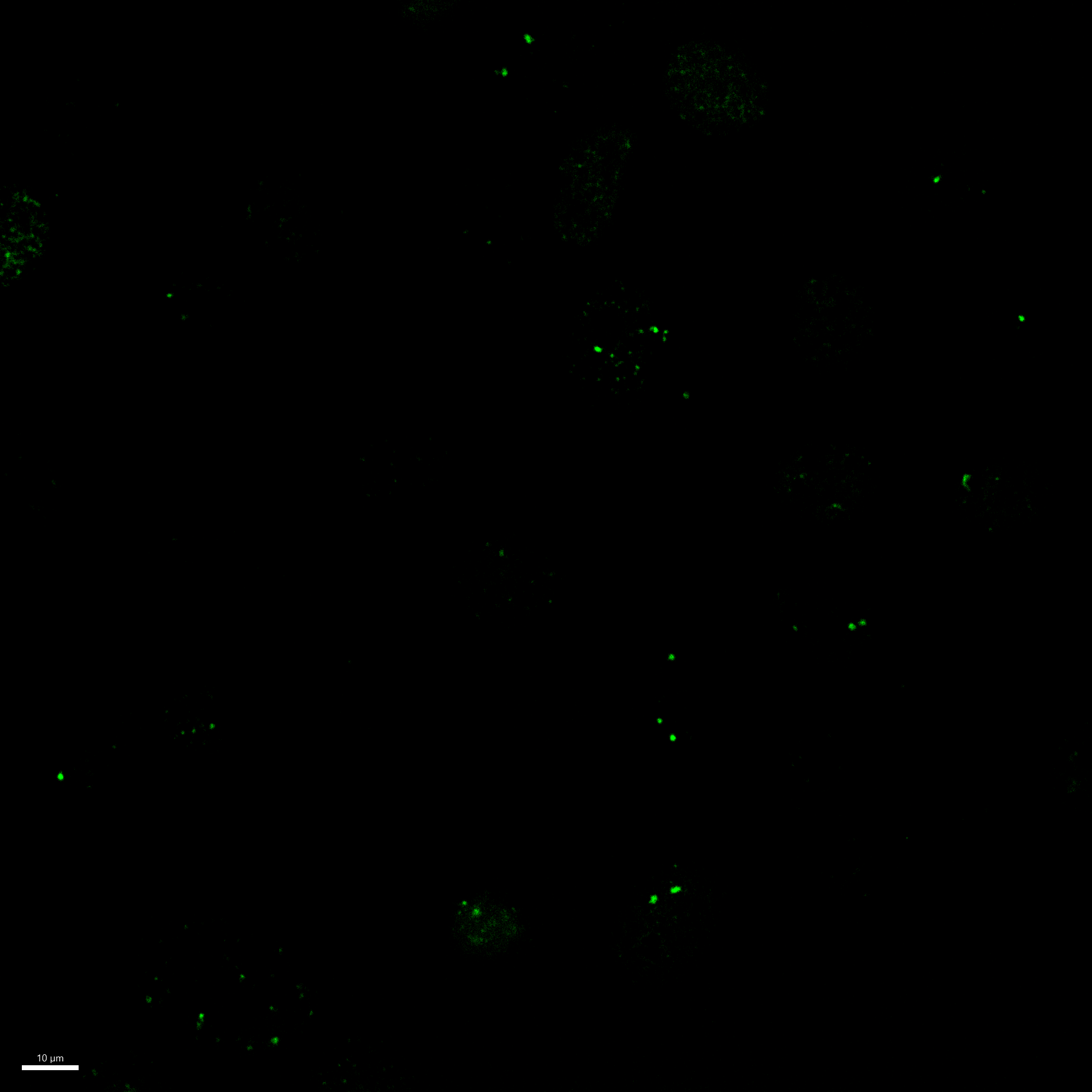

Supplement: Supplementary file 15 — Figure EV3 Source Data [file 44321_2026_414_MOESM15_ESM.zip › Fig. EV3/EV3F/HeyA8 shBMAL2#1 yH2ax.tif]

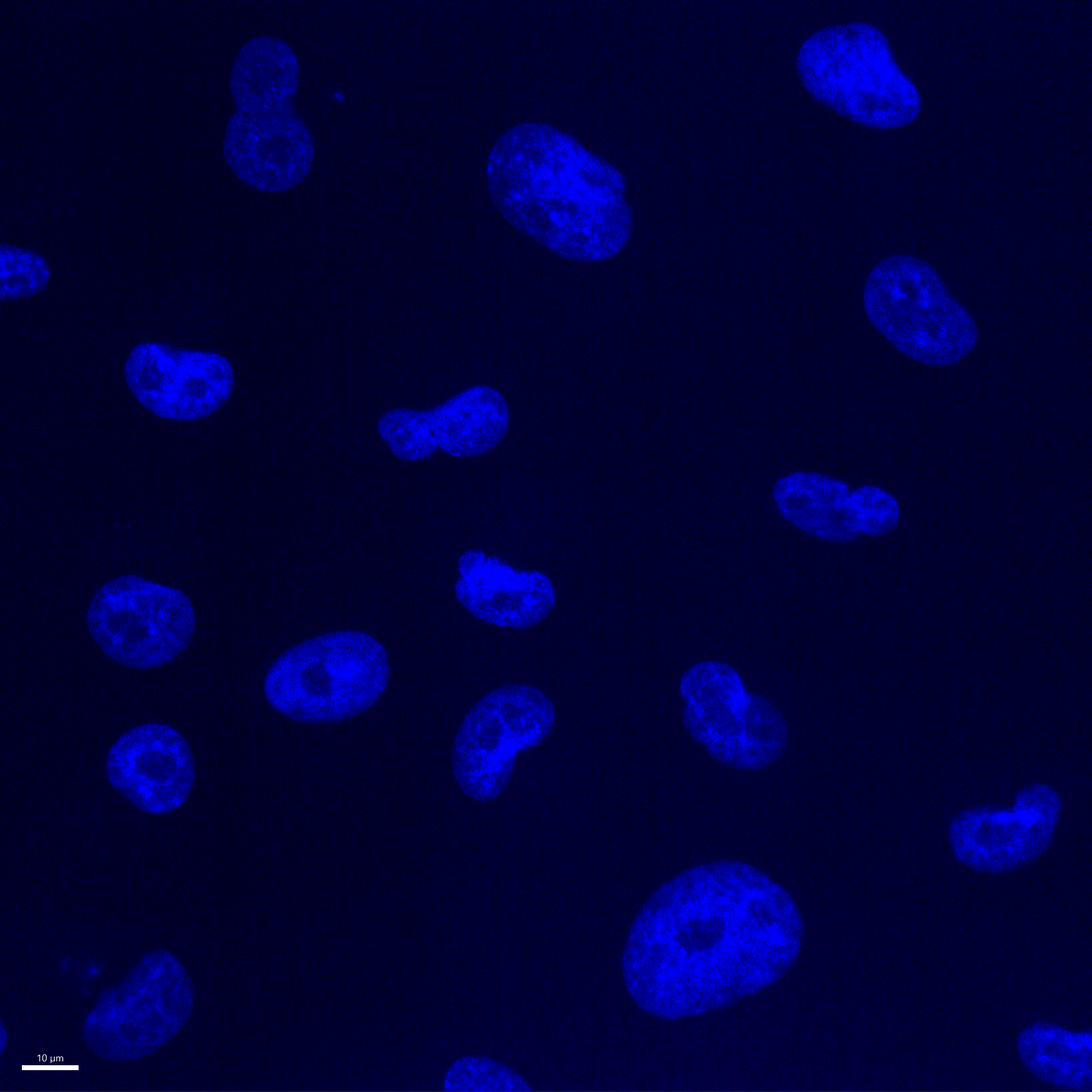

Supplement: Supplementary file 15 — Figure EV3 Source Data [file 44321_2026_414_MOESM15_ESM.zip › Fig. EV3/EV3F/HeyA8 shBMAL2#2 DAPI.tif]

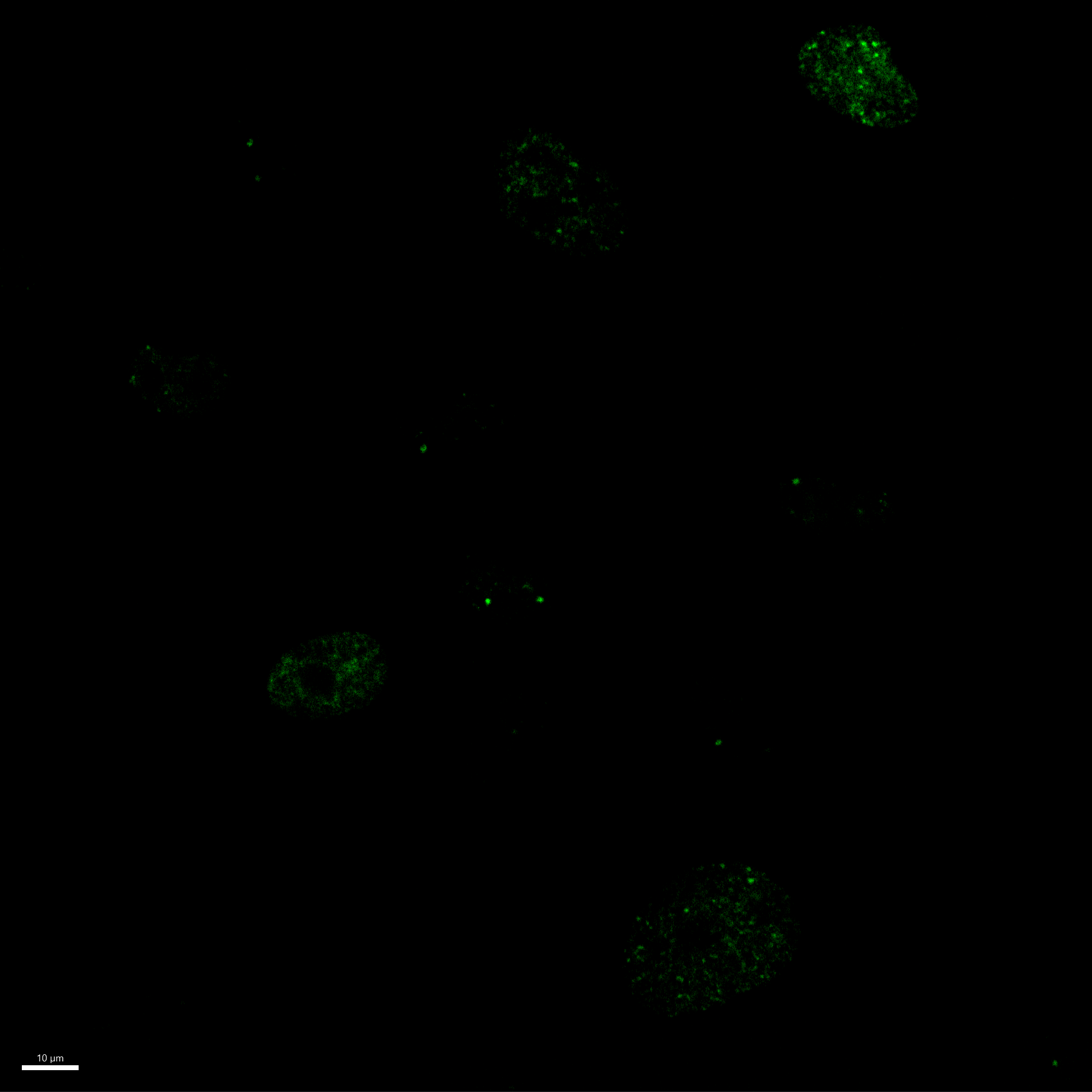

Supplement: Supplementary file 15 — Figure EV3 Source Data [file 44321_2026_414_MOESM15_ESM.zip › Fig. EV3/EV3F/HeyA8 shBMAL2#2 yh2ax.tif]

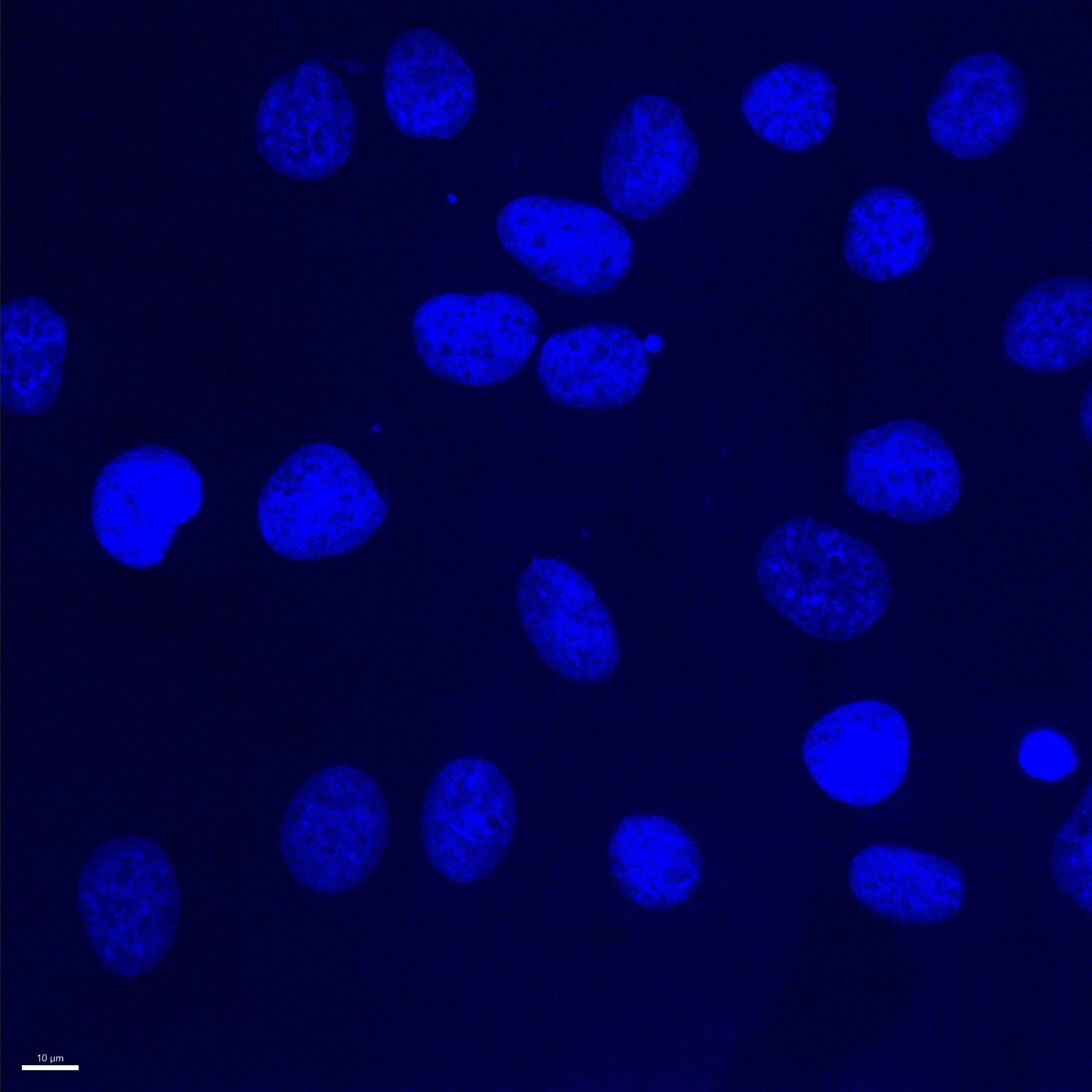

Supplement: Supplementary file 15 — Figure EV3 Source Data [file 44321_2026_414_MOESM15_ESM.zip › Fig. EV3/EV3F/HeyA8 shCtrl DAPI.tif]

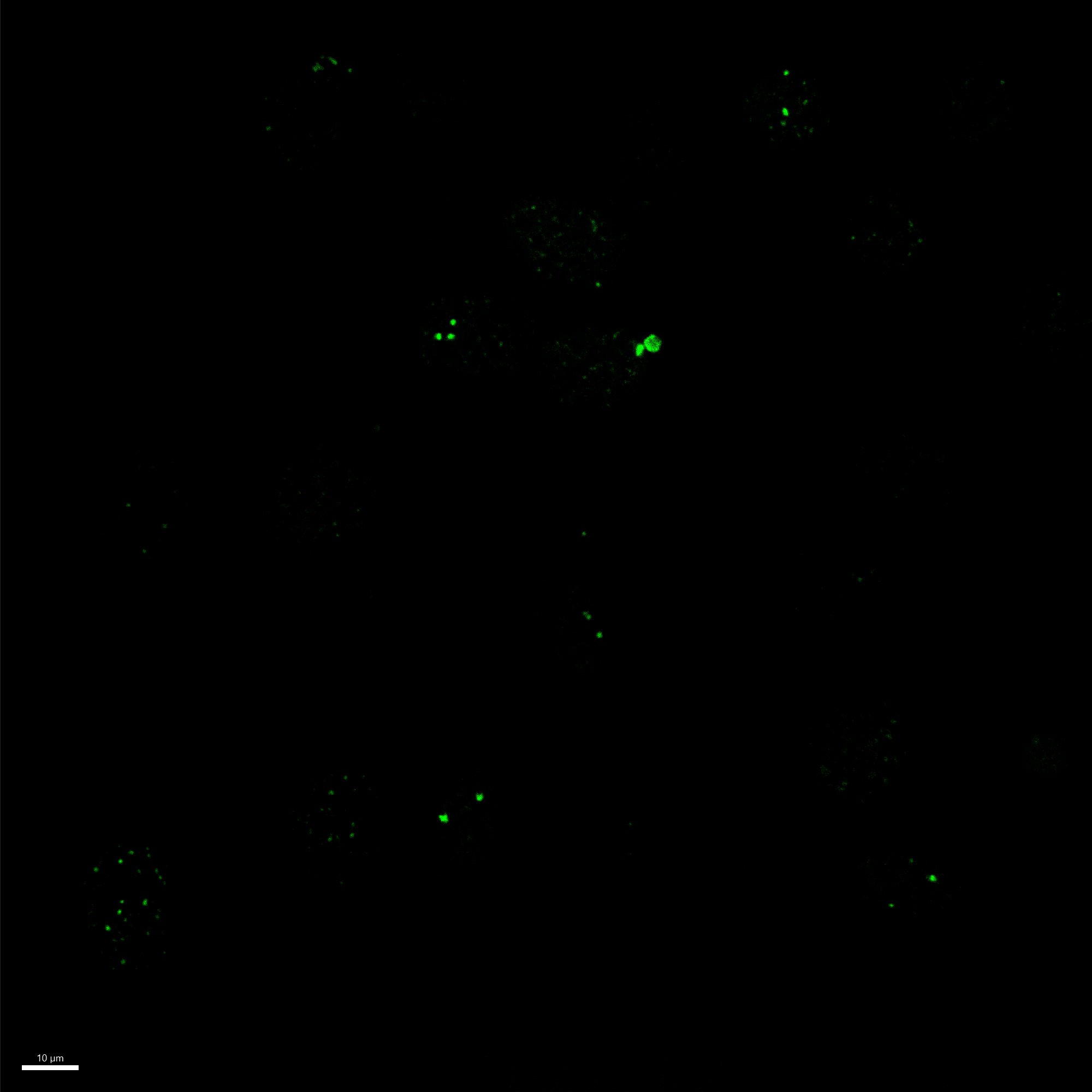

Supplement: Supplementary file 15 — Figure EV3 Source Data [file 44321_2026_414_MOESM15_ESM.zip › Fig. EV3/EV3F/HeyA8 shCtrl yH2ax.tif]

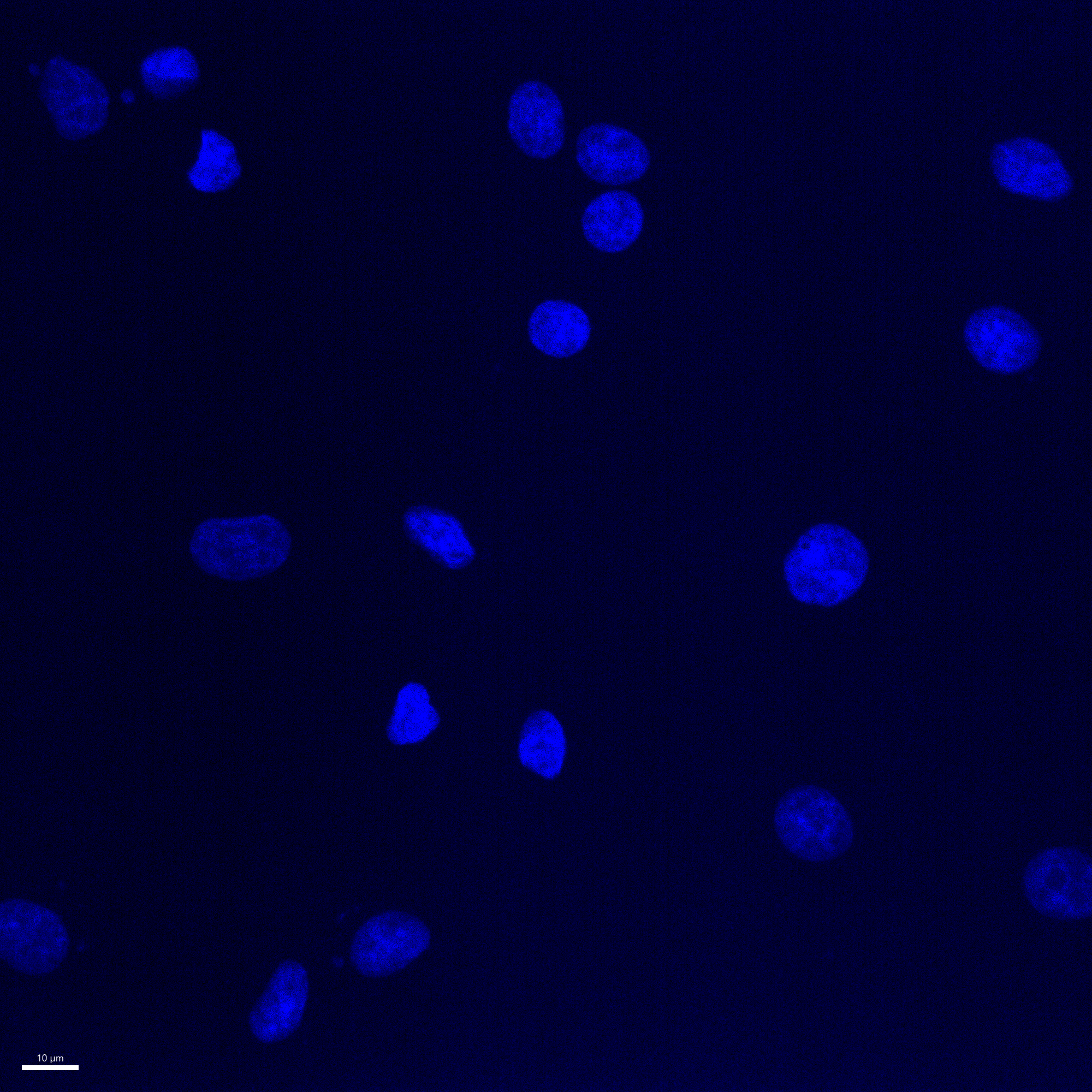

Supplement: Supplementary file 15 — Figure EV3 Source Data [file 44321_2026_414_MOESM15_ESM.zip › Fig. EV3/EV3F/Kuramochi shBMAL2#1 DAPI.tif]

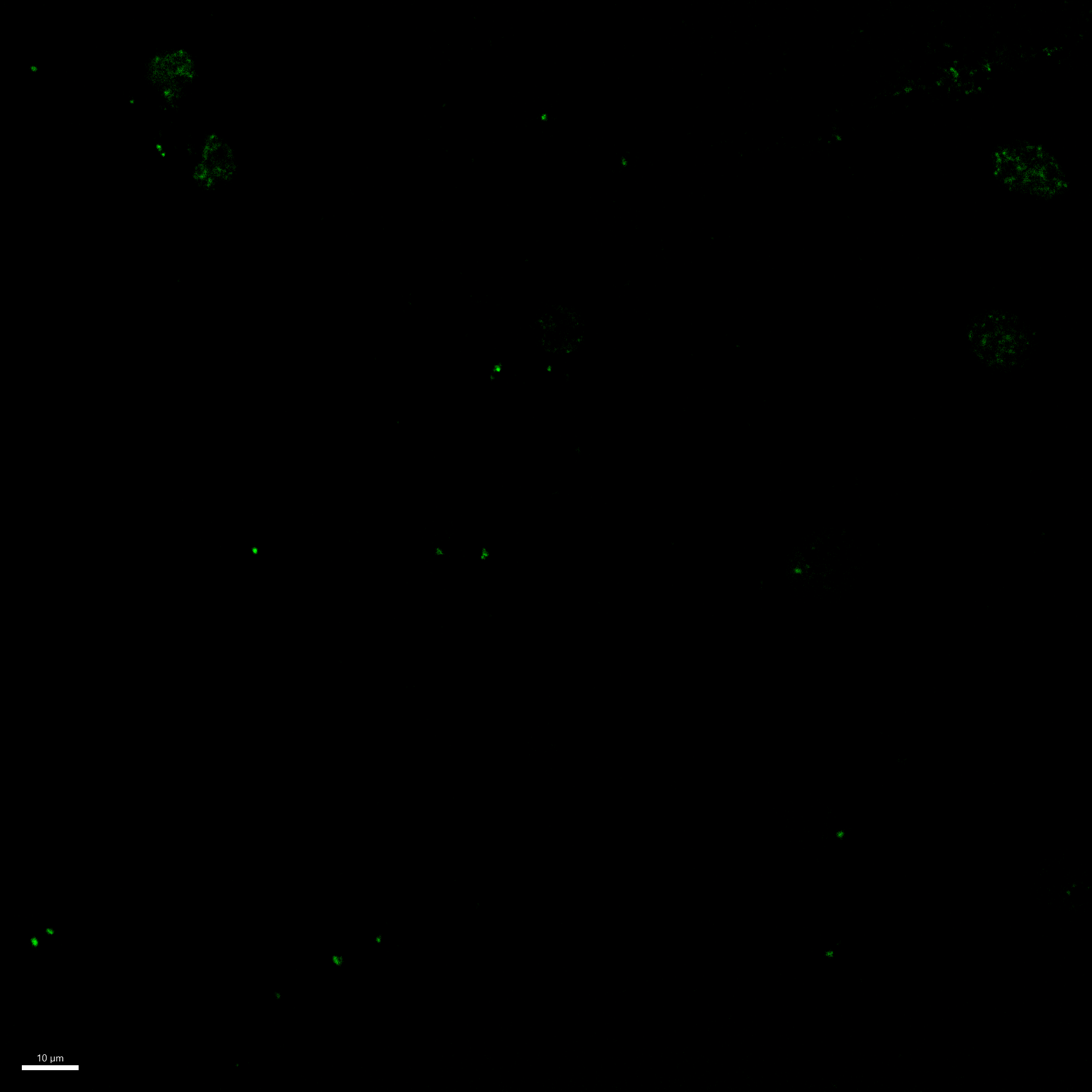

Supplement: Supplementary file 15 — Figure EV3 Source Data [file 44321_2026_414_MOESM15_ESM.zip › Fig. EV3/EV3F/Kuramochi shBMAL2#1 yH2ax.tif]

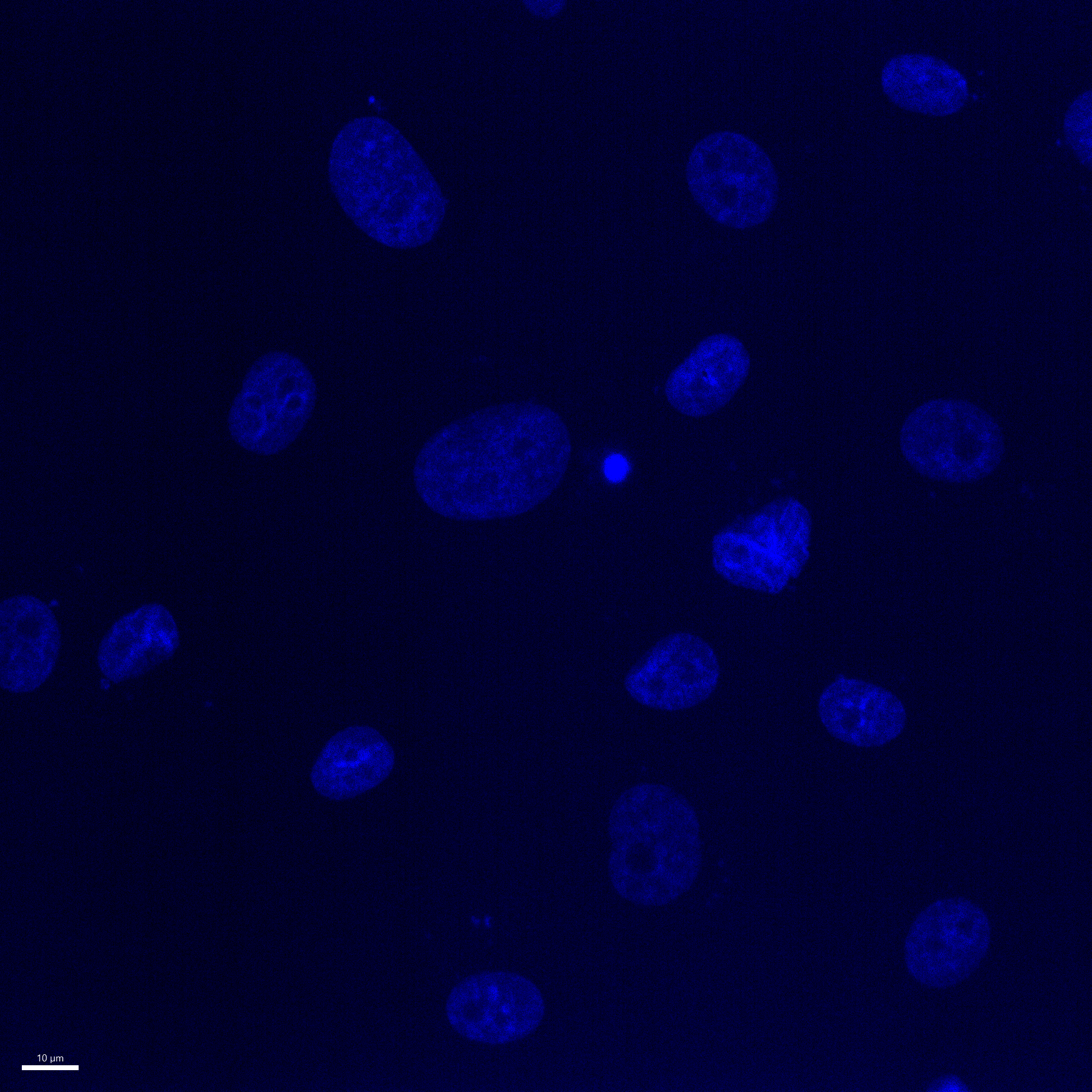

Supplement: Supplementary file 15 — Figure EV3 Source Data [file 44321_2026_414_MOESM15_ESM.zip › Fig. EV3/EV3F/Kuramochi shBMAL2#2 DAPI.tif]

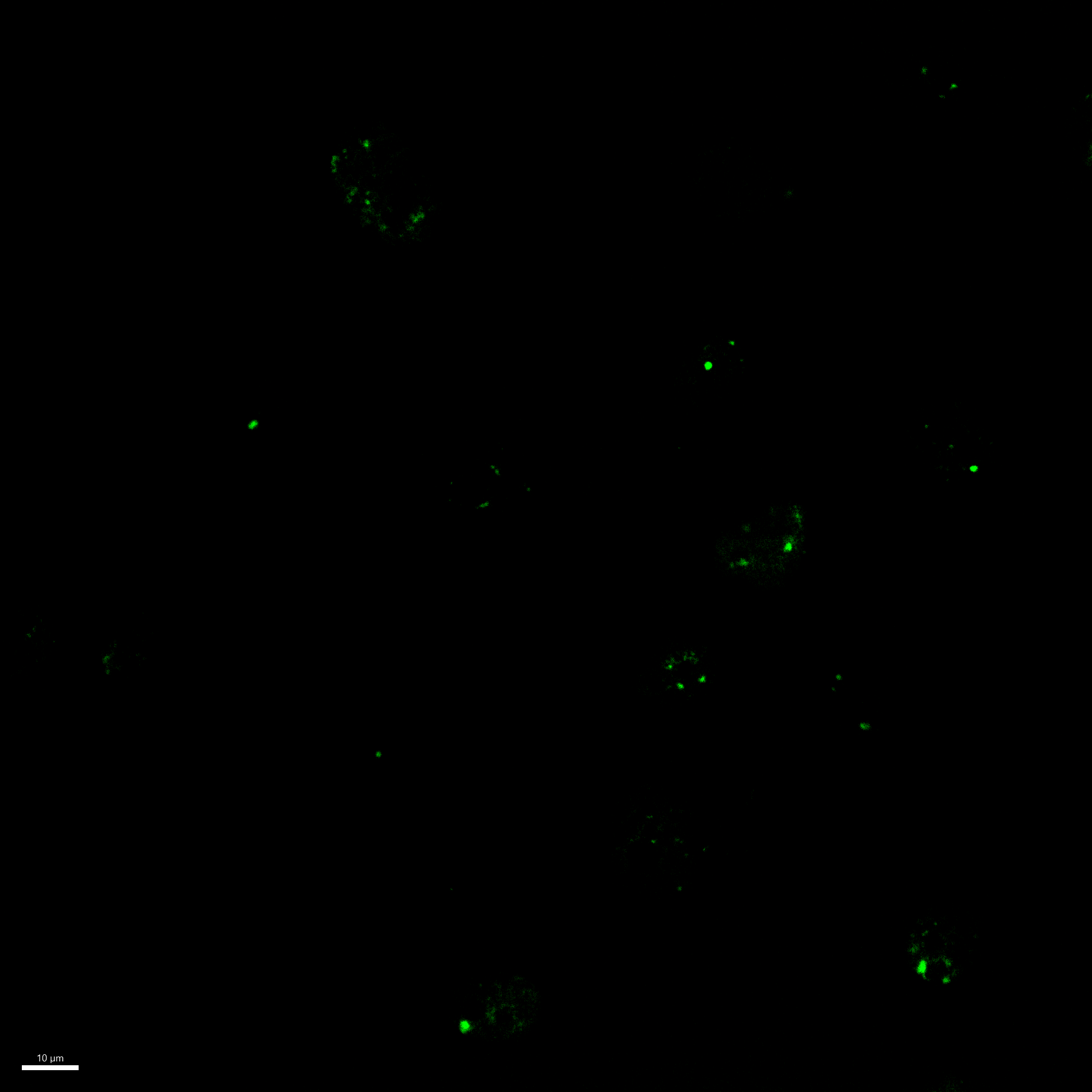

Supplement: Supplementary file 15 — Figure EV3 Source Data [file 44321_2026_414_MOESM15_ESM.zip › Fig. EV3/EV3F/Kuramochi shBMAL2#2 yH2ax.tif]

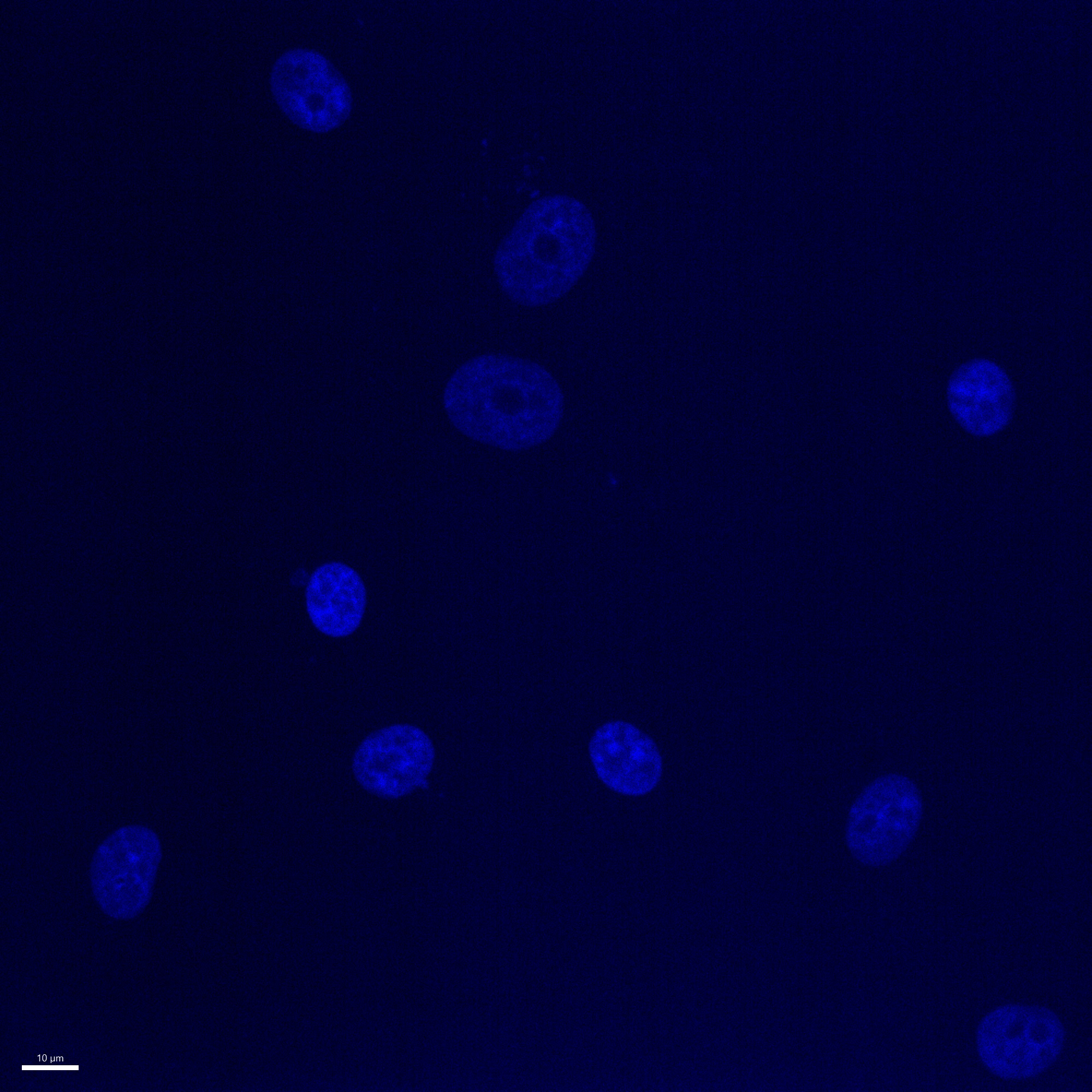

Supplement: Supplementary file 15 — Figure EV3 Source Data [file 44321_2026_414_MOESM15_ESM.zip › Fig. EV3/EV3F/Kuramochi shCtrl DAPI.tif]

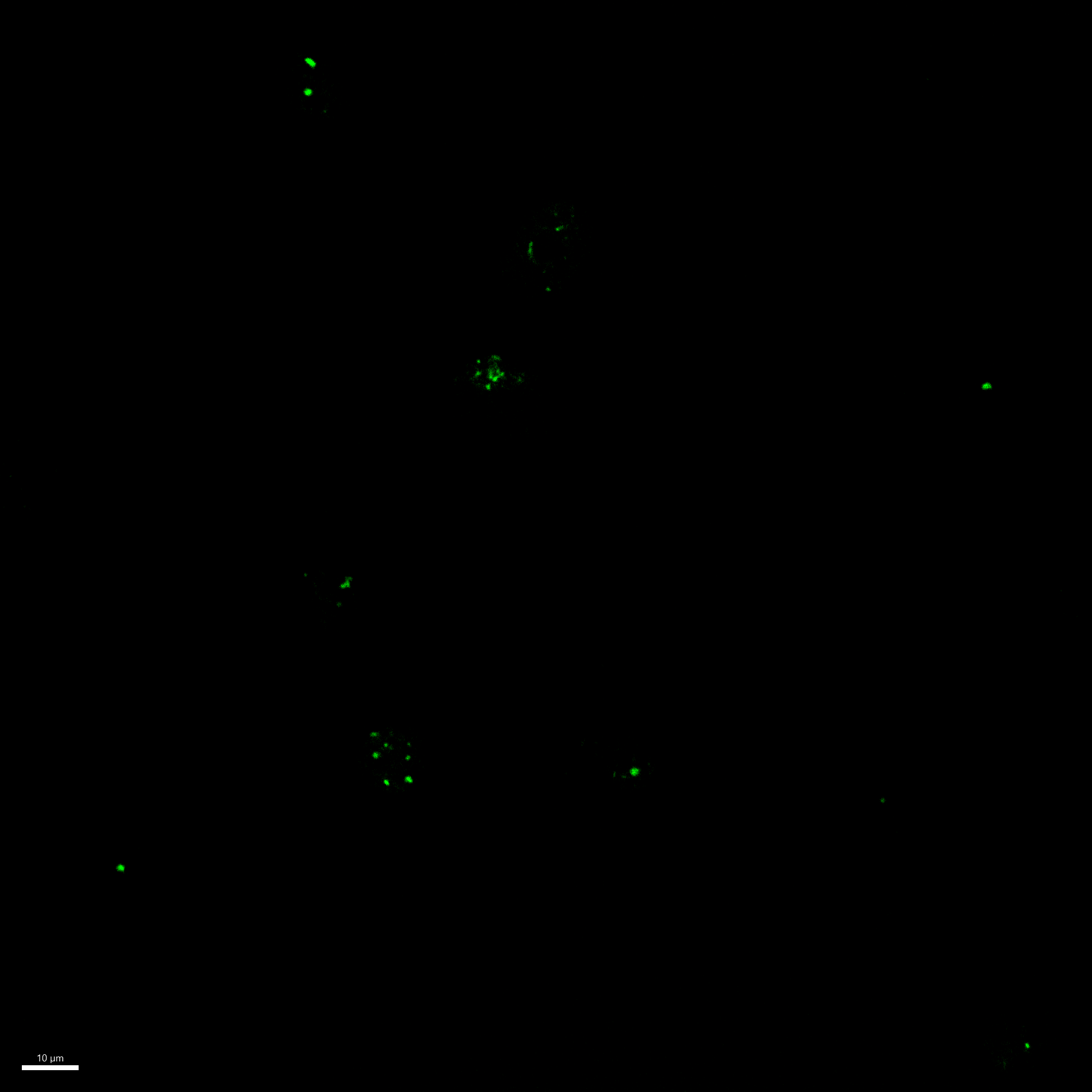

Supplement: Supplementary file 15 — Figure EV3 Source Data [file 44321_2026_414_MOESM15_ESM.zip › Fig. EV3/EV3F/Kuramochi shCtrl yH2ax.tif]

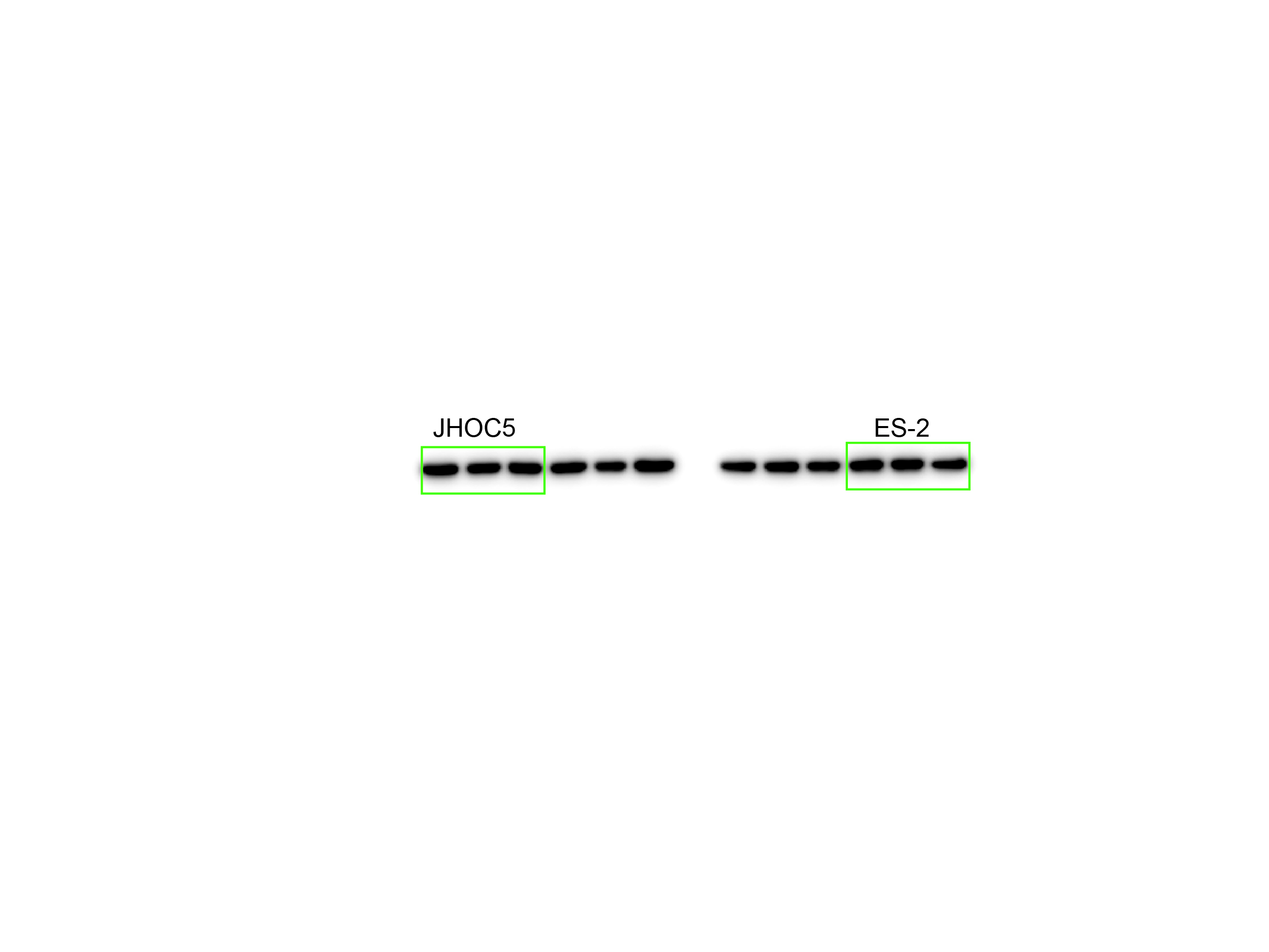

Supplement: Supplementary file 16 — Figure EV4 Source Data [file 44321_2026_414_MOESM16_ESM.zip › Fig. EV4/EV4D/ES-2, JHOC5 GAPDH IB.jpg]

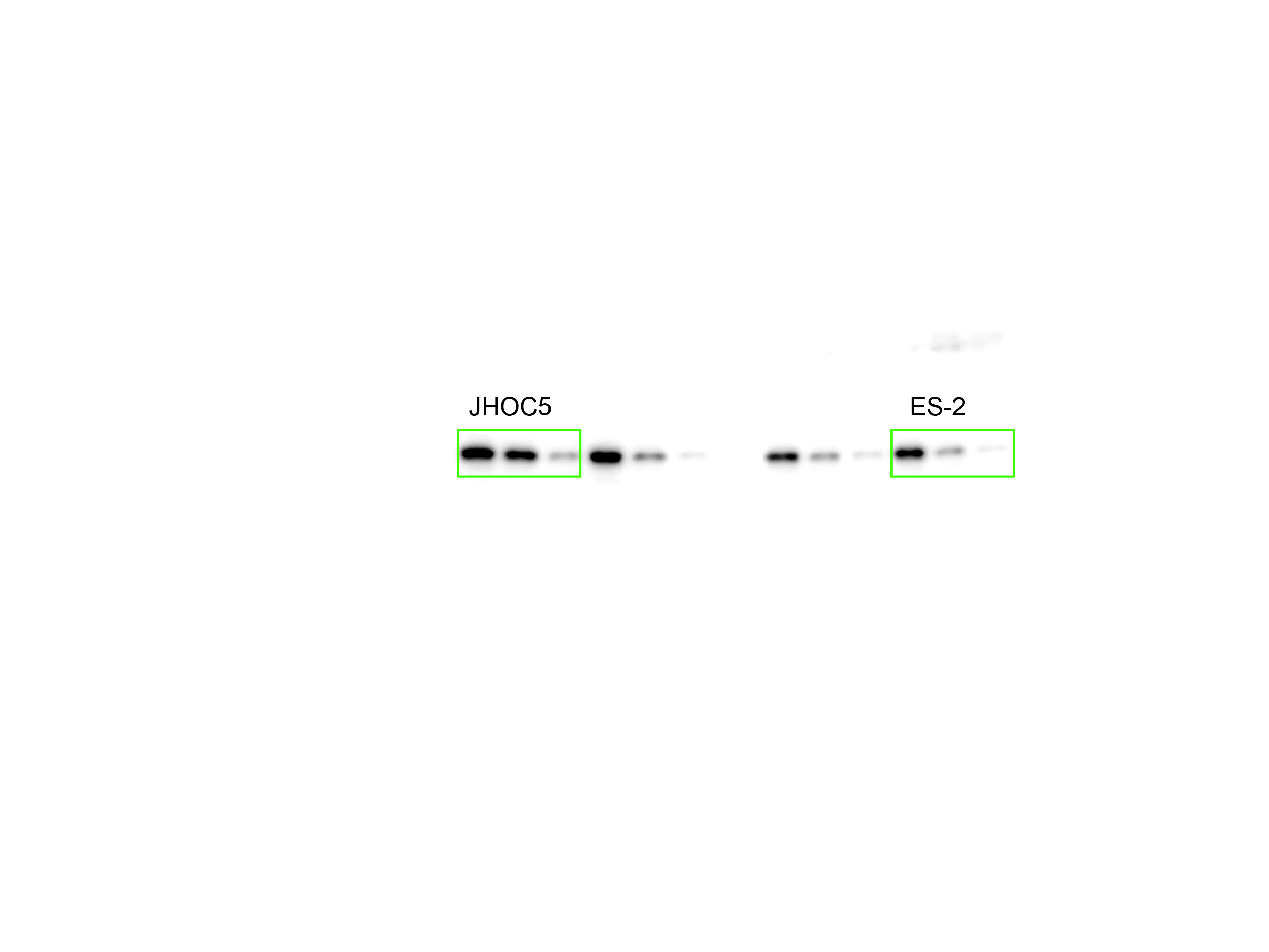

Supplement: Supplementary file 16 — Figure EV4 Source Data [file 44321_2026_414_MOESM16_ESM.zip › Fig. EV4/EV4D/ES-2, JHOC5 RAD51 IB.jpg]

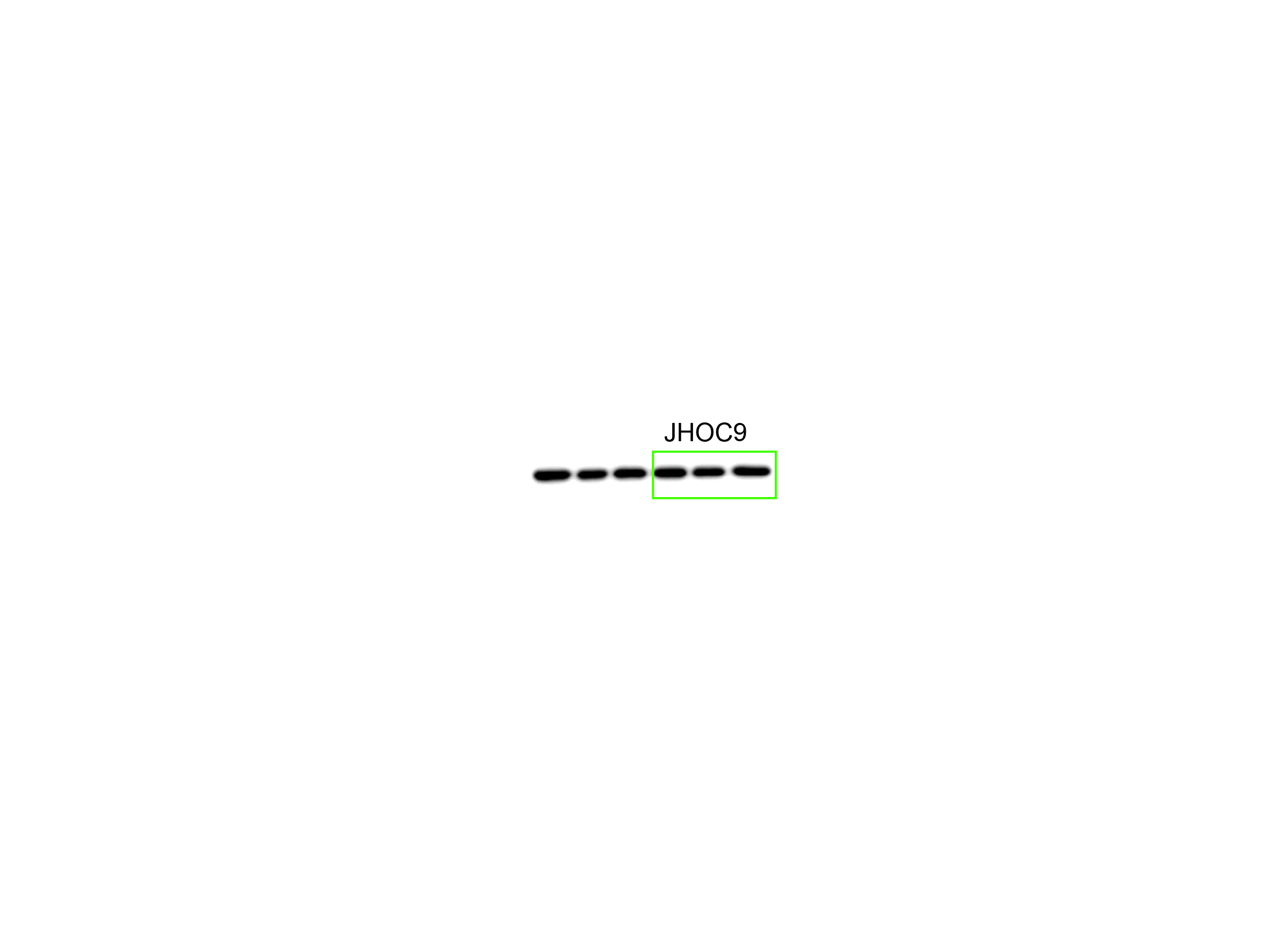

Supplement: Supplementary file 16 — Figure EV4 Source Data [file 44321_2026_414_MOESM16_ESM.zip › Fig. EV4/EV4D/JHOC9 GAPDH IB.jpg]

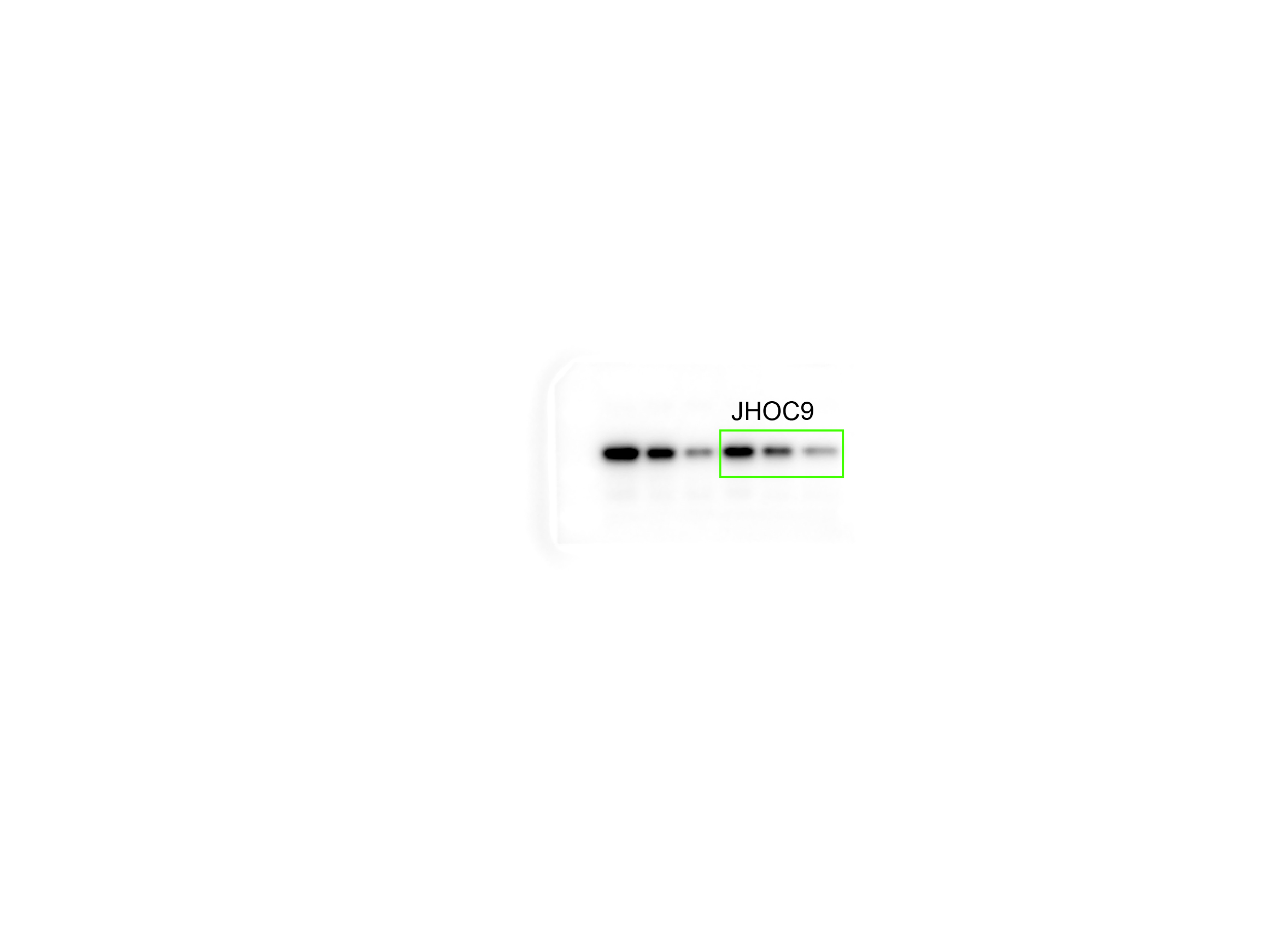

Supplement: Supplementary file 16 — Figure EV4 Source Data [file 44321_2026_414_MOESM16_ESM.zip › Fig. EV4/EV4D/JHOC9 RAD51 IB.jpg]

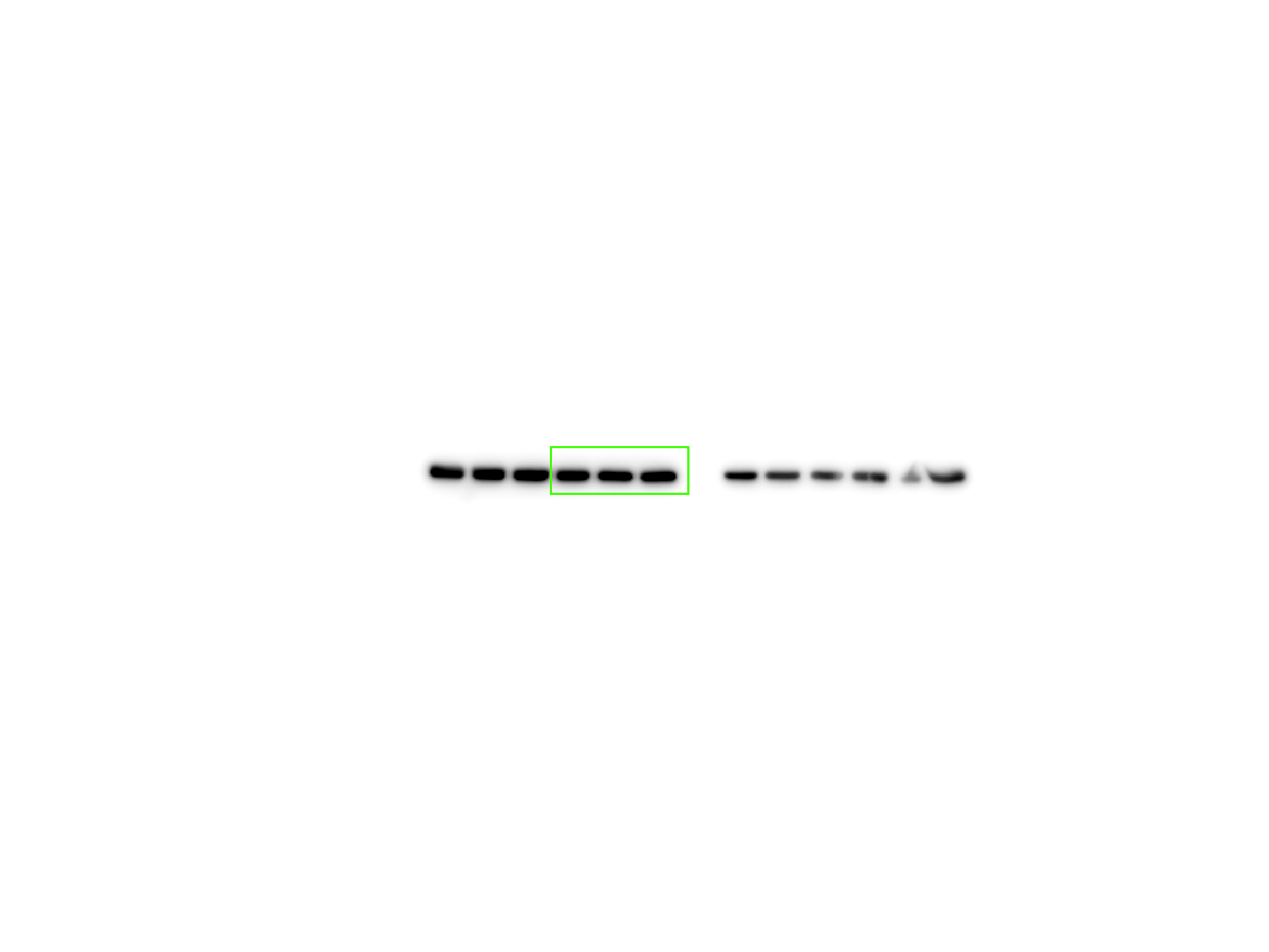

Supplement: Supplementary file 16 — Figure EV4 Source Data [file 44321_2026_414_MOESM16_ESM.zip › Fig. EV4/EV4D/OVCA429 GAPDH IB.jpg]

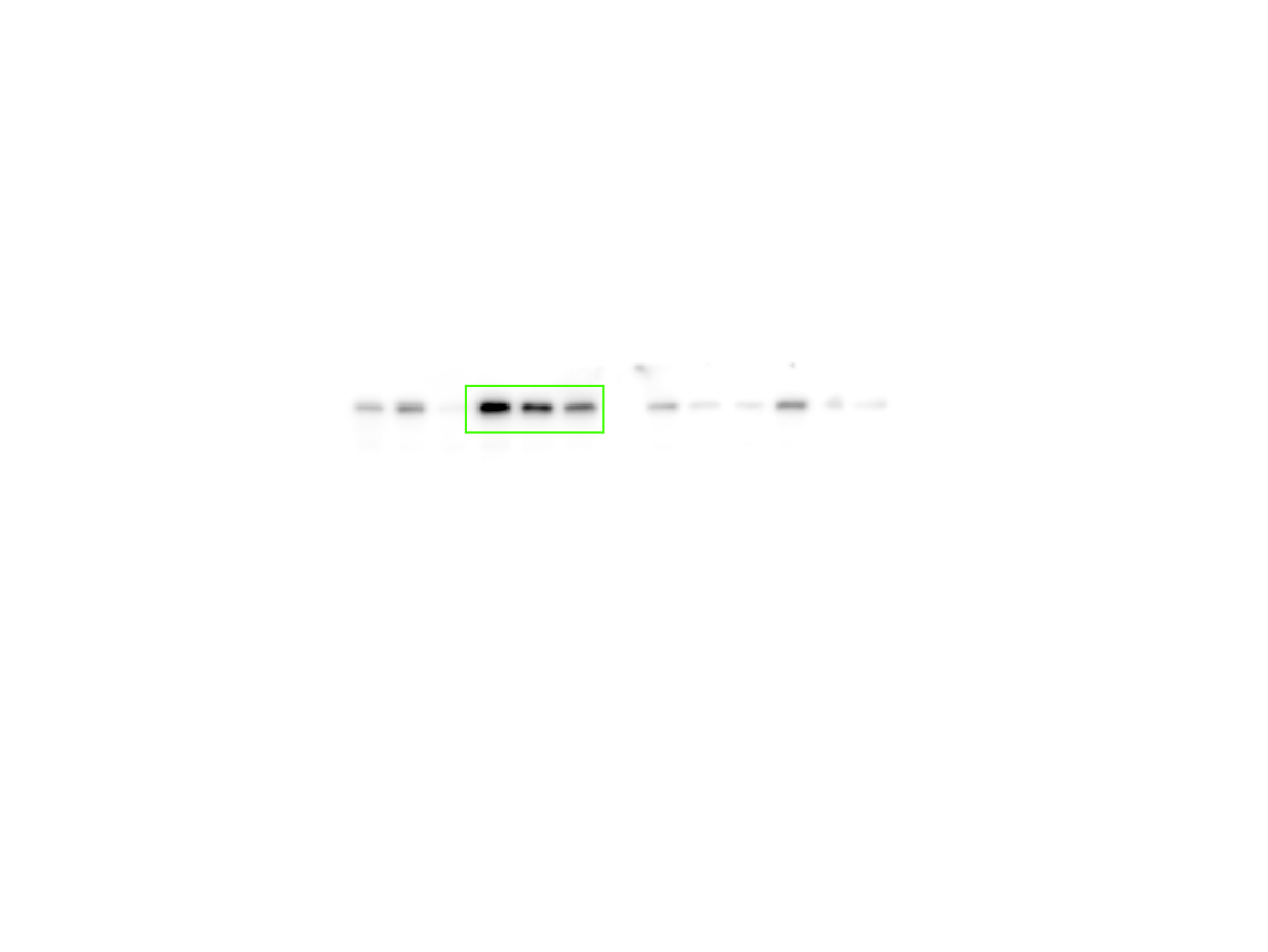

Supplement: Supplementary file 16 — Figure EV4 Source Data [file 44321_2026_414_MOESM16_ESM.zip › Fig. EV4/EV4D/OVCA429 RAD51 IB.jpg]

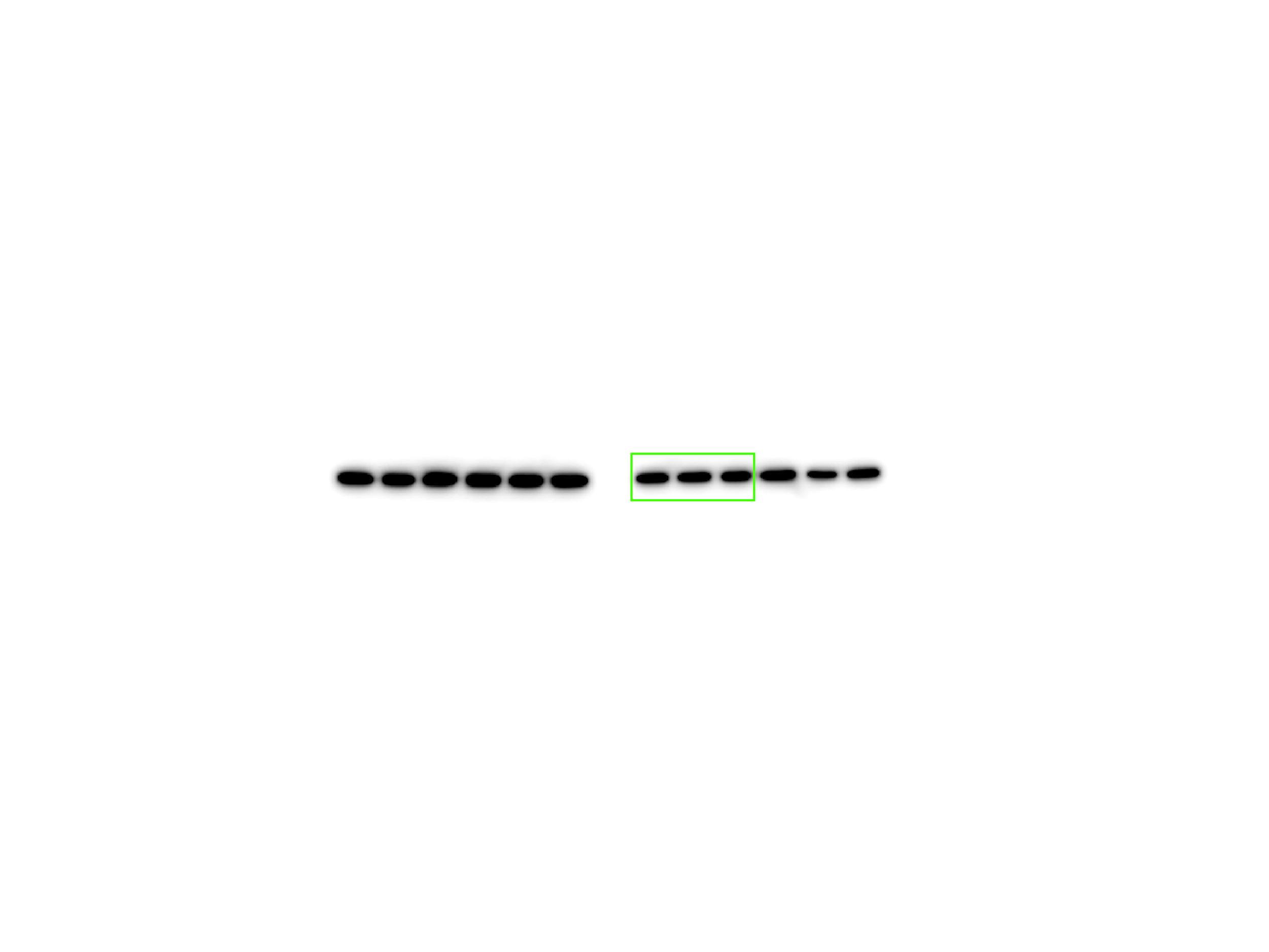

Supplement: Supplementary file 16 — Figure EV4 Source Data [file 44321_2026_414_MOESM16_ESM.zip › Fig. EV4/EV4D/OVISE GAPDH IB.jpg]

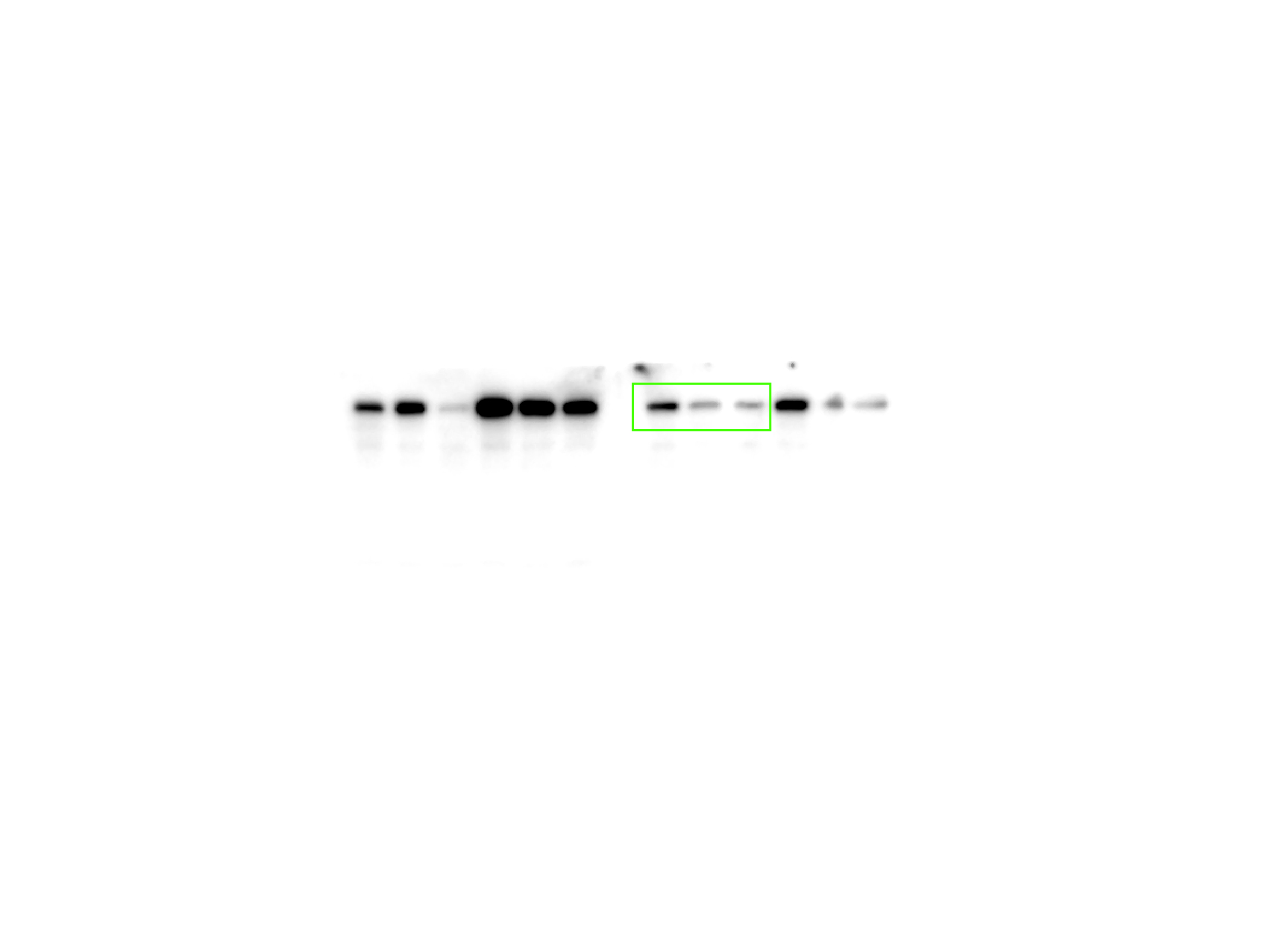

Supplement: Supplementary file 16 — Figure EV4 Source Data [file 44321_2026_414_MOESM16_ESM.zip › Fig. EV4/EV4D/OVISE RAD51 IB.jpg]

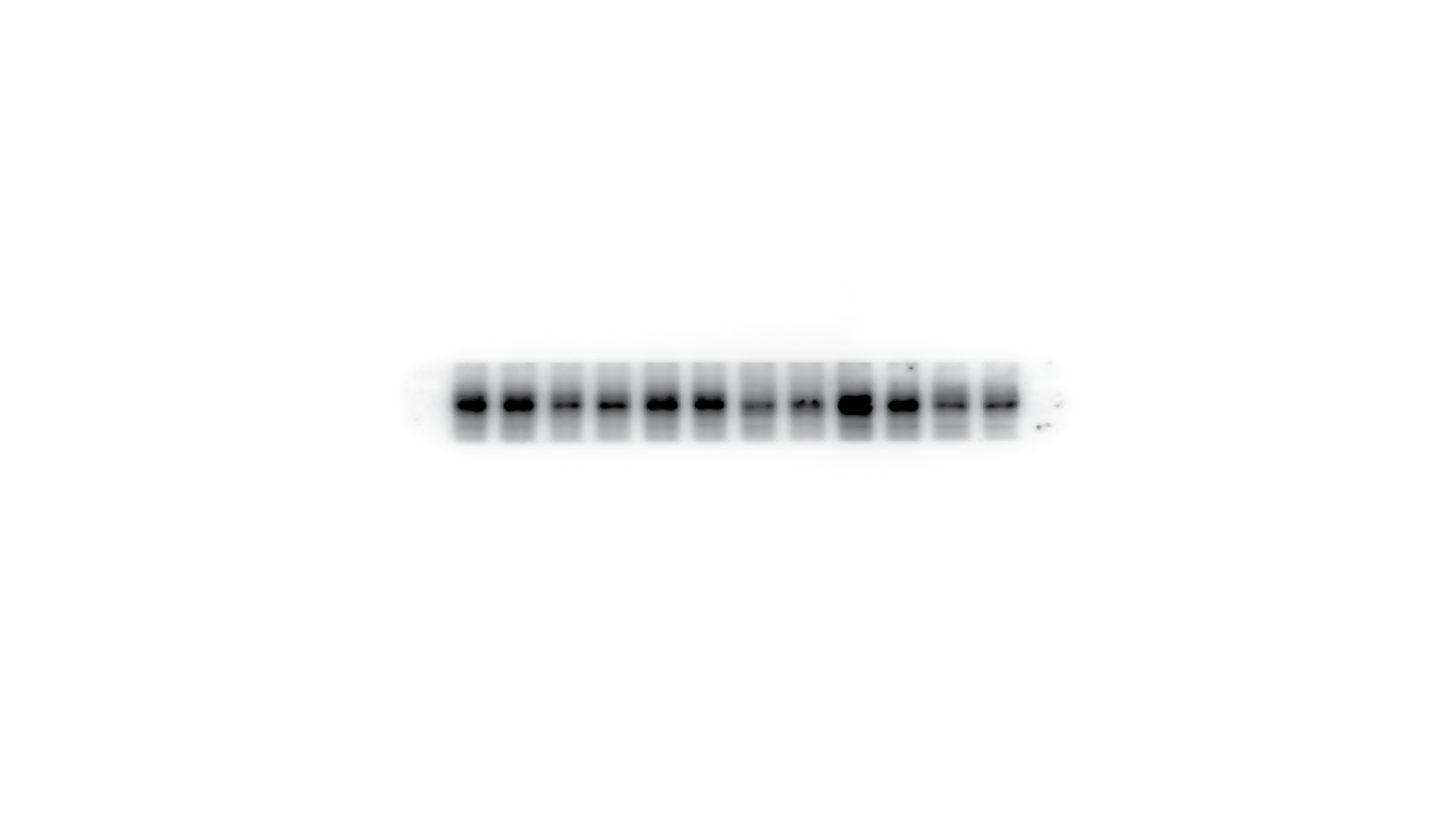

Supplement: Supplementary file 18 — Figure EV6 Source Data [file 44321_2026_414_MOESM18_ESM.zip › Fig. EV6/Fig. EV6C/Veh, compound 1-5 BMAL2 IB.png]

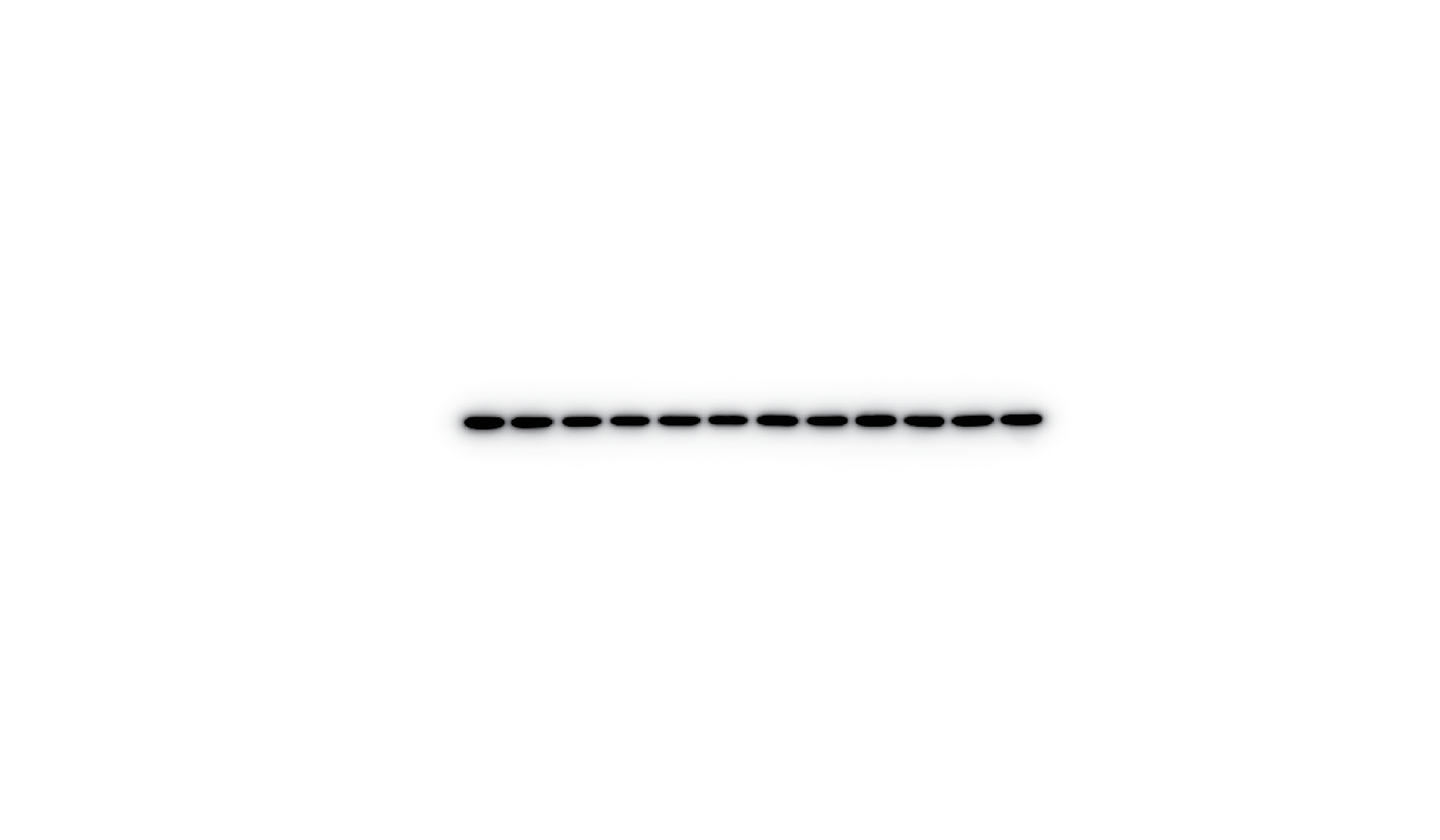

Supplement: Supplementary file 18 — Figure EV6 Source Data [file 44321_2026_414_MOESM18_ESM.zip › Fig. EV6/Fig. EV6C/Veh, Compound 1-5 GAPDH IB.png]

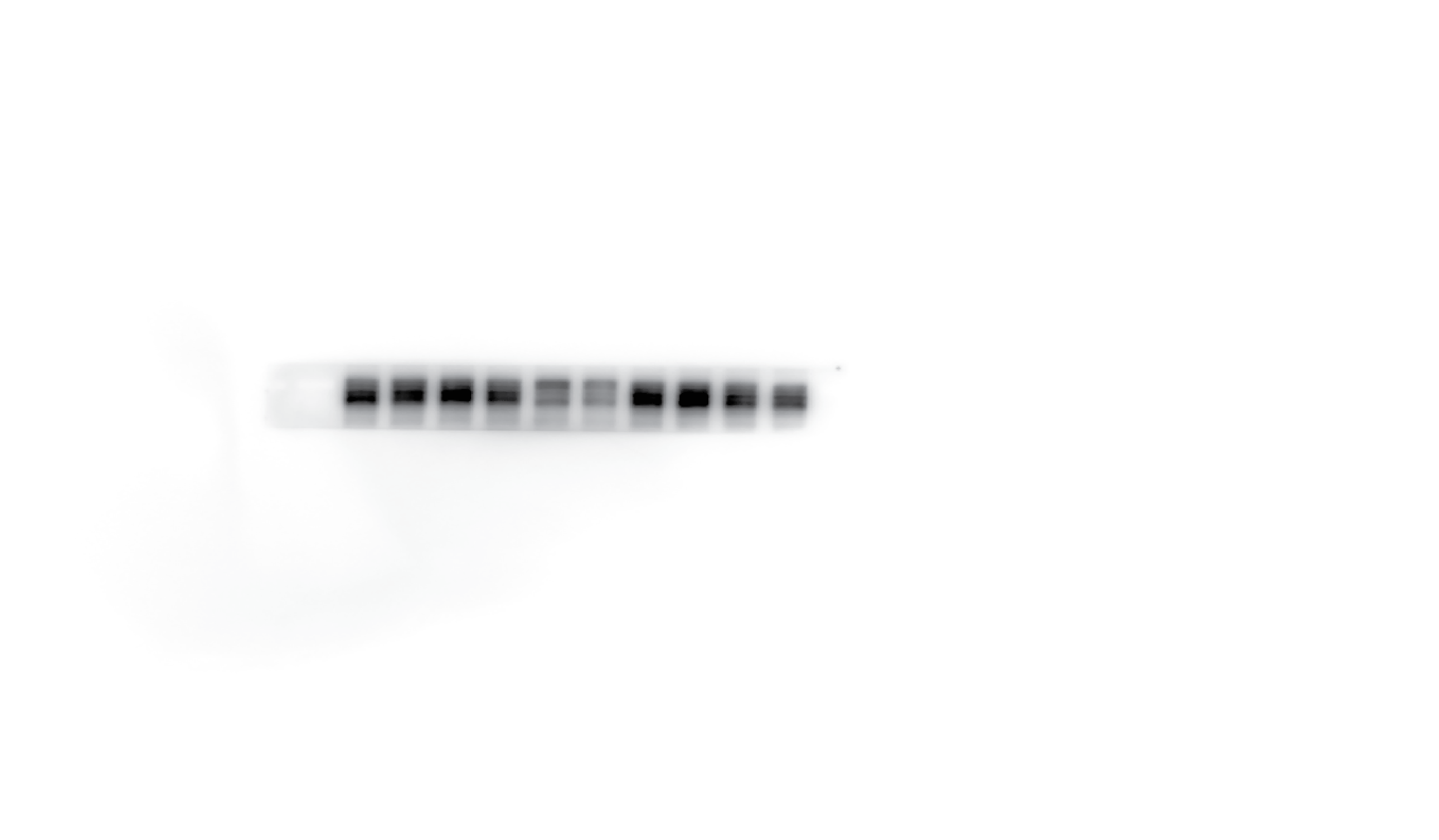

Supplement: Supplementary file 18 — Figure EV6 Source Data [file 44321_2026_414_MOESM18_ESM.zip › Fig. EV6/Fig. EV6C/Veh, Compound 6-9 BMAL2 IB.png]

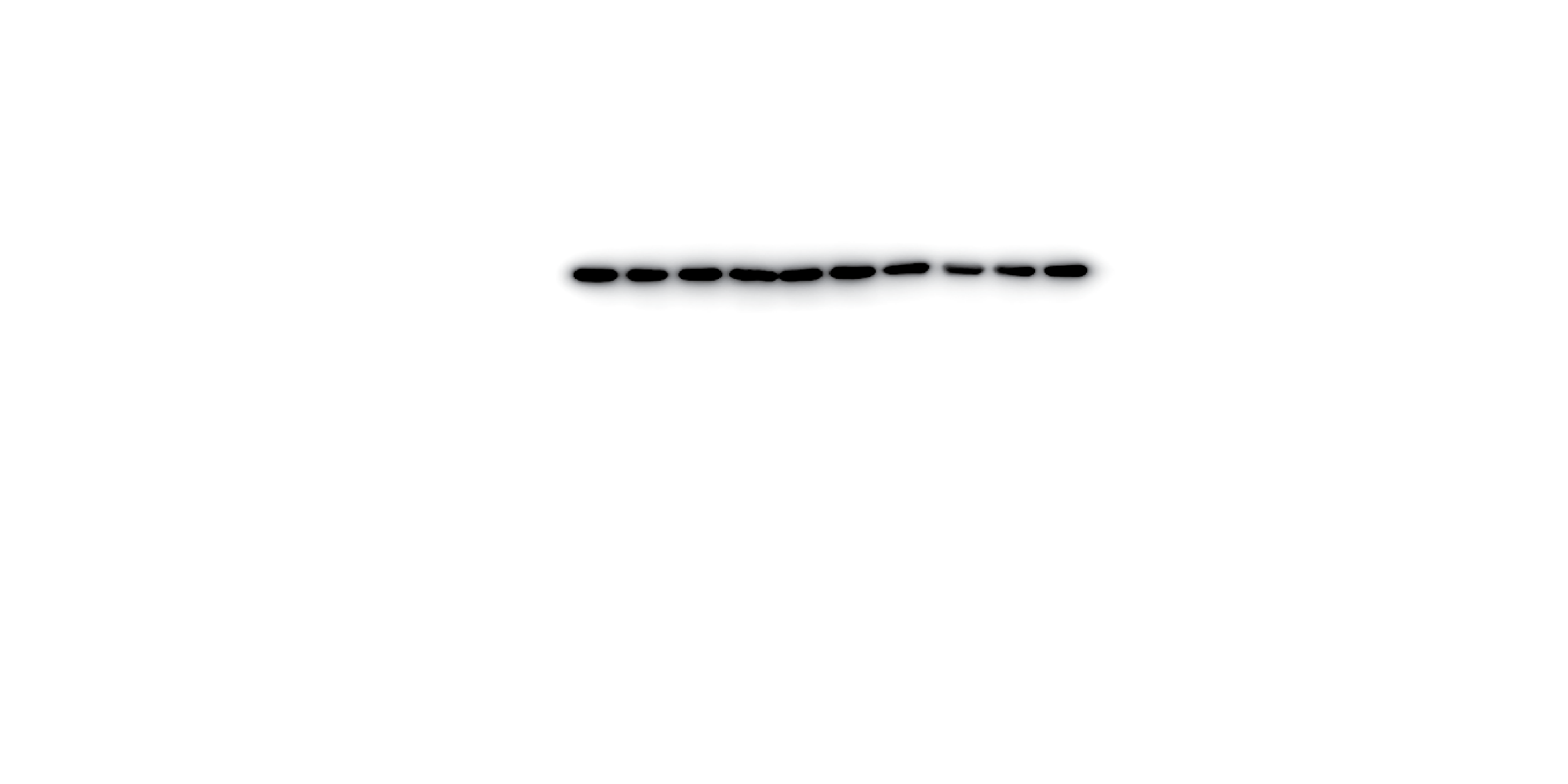

Supplement: Supplementary file 18 — Figure EV6 Source Data [file 44321_2026_414_MOESM18_ESM.zip › Fig. EV6/Fig. EV6C/Veh, Compound 6-9 GAPDH IB.png]

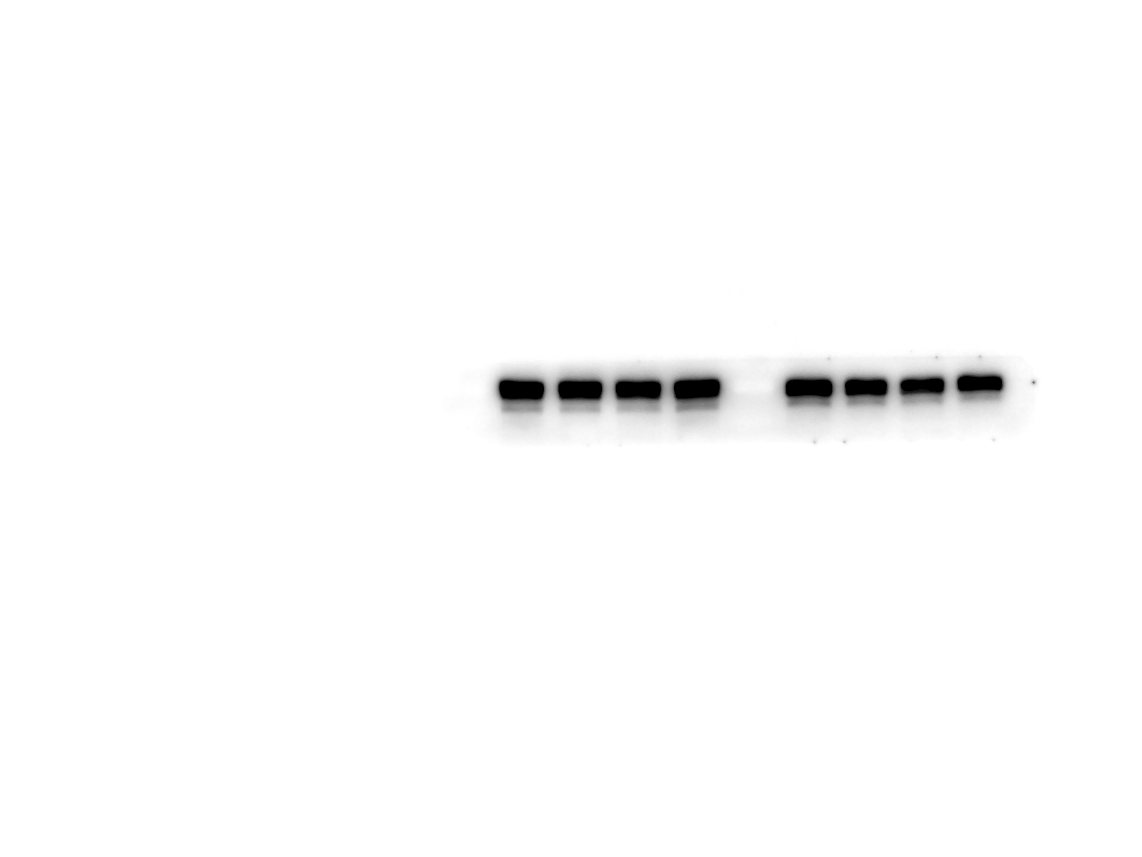

Supplement: Supplementary file 19 — Figure EV7 Source Data [file 44321_2026_414_MOESM19_ESM.zip › Fig. EV7/EV7B/BMAL1 IB (long exp).tif]

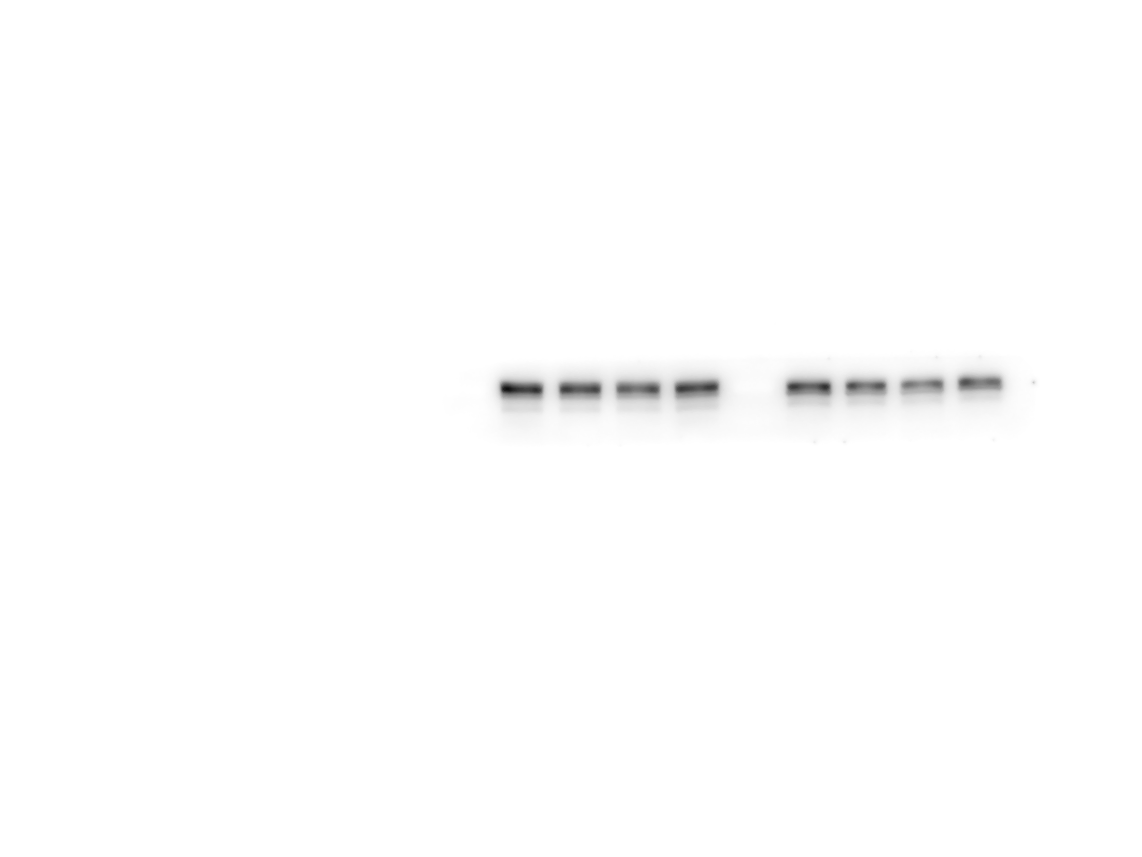

Supplement: Supplementary file 19 — Figure EV7 Source Data [file 44321_2026_414_MOESM19_ESM.zip › Fig. EV7/EV7B/BMAL1 IB.tif]

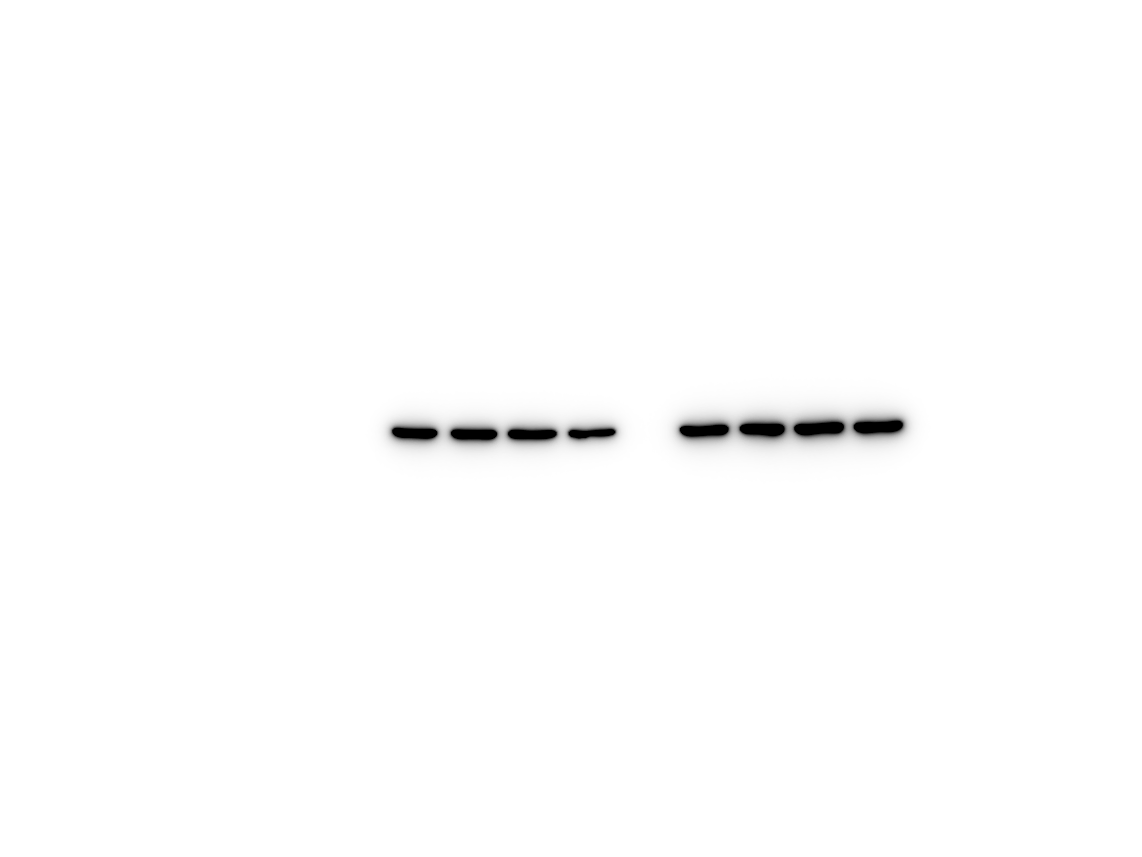

Supplement: Supplementary file 19 — Figure EV7 Source Data [file 44321_2026_414_MOESM19_ESM.zip › Fig. EV7/EV7B/GAPDH IB.tif]

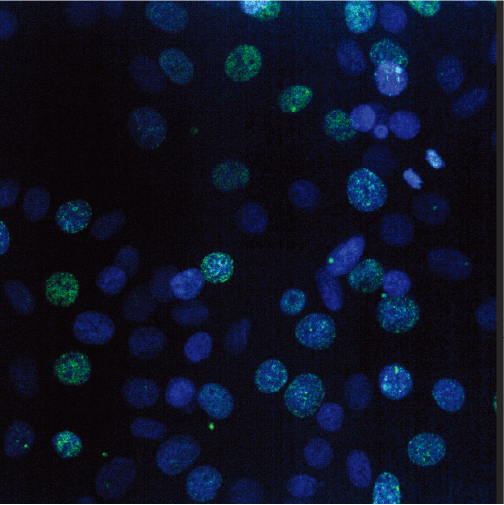

Supplement: Supplementary file 19 — Figure EV7 Source Data [file 44321_2026_414_MOESM19_ESM.zip › Fig. EV7/EV7D/ES2 10uM yH2AX DAPI IF.png]

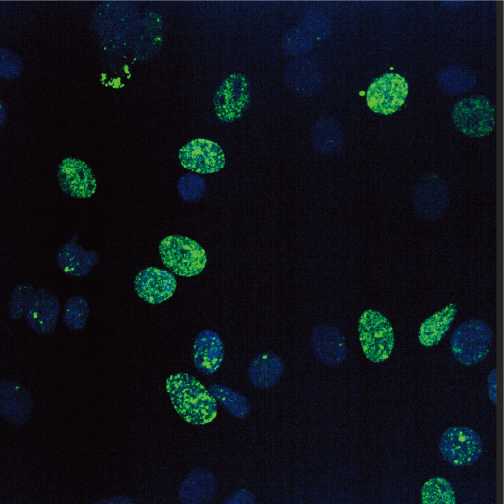

Supplement: Supplementary file 19 — Figure EV7 Source Data [file 44321_2026_414_MOESM19_ESM.zip › Fig. EV7/EV7D/ES2 20uM yH2AX DAPI IF.png]

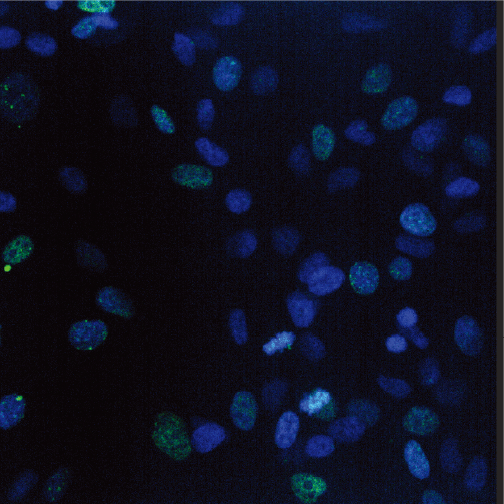

Supplement: Supplementary file 19 — Figure EV7 Source Data [file 44321_2026_414_MOESM19_ESM.zip › Fig. EV7/EV7D/ES2 Vehicle yH2AX DAPI IF.png]

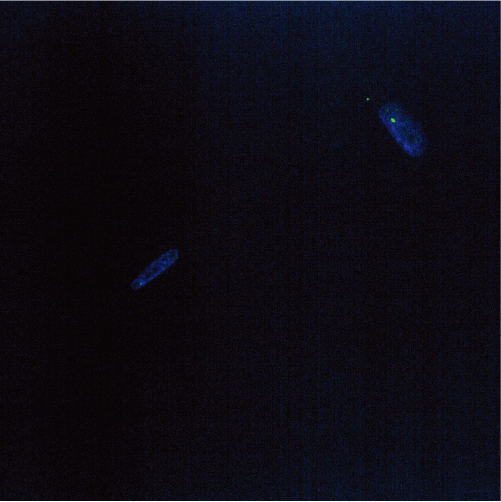

Supplement: Supplementary file 19 — Figure EV7 Source Data [file 44321_2026_414_MOESM19_ESM.zip › Fig. EV7/EV7D/WI38 10uM yH2AX DAPI IF.png]

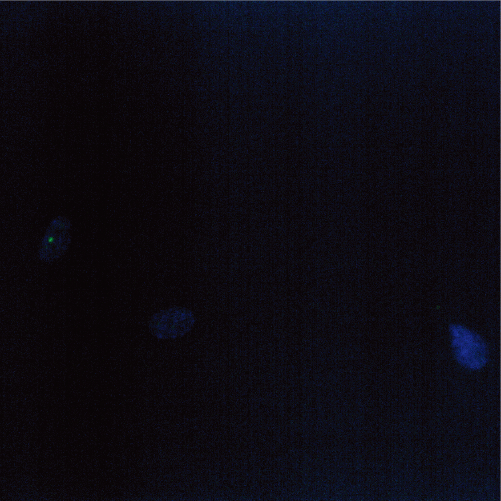

Supplement: Supplementary file 19 — Figure EV7 Source Data [file 44321_2026_414_MOESM19_ESM.zip › Fig. EV7/EV7D/WI38 20uM yH2AX DAPI IF.png]

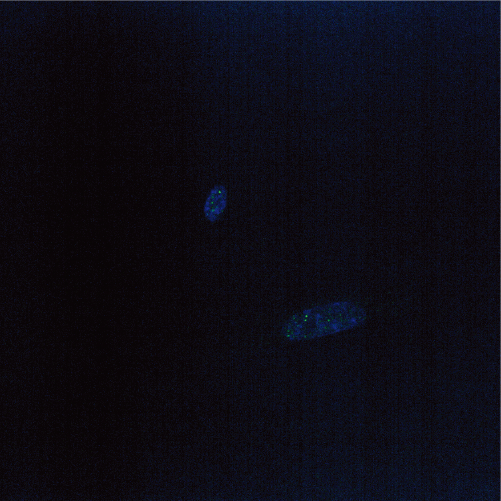

Supplement: Supplementary file 19 — Figure EV7 Source Data [file 44321_2026_414_MOESM19_ESM.zip › Fig. EV7/EV7D/WI38 Vehicle yH2AX DAPI IF.png]

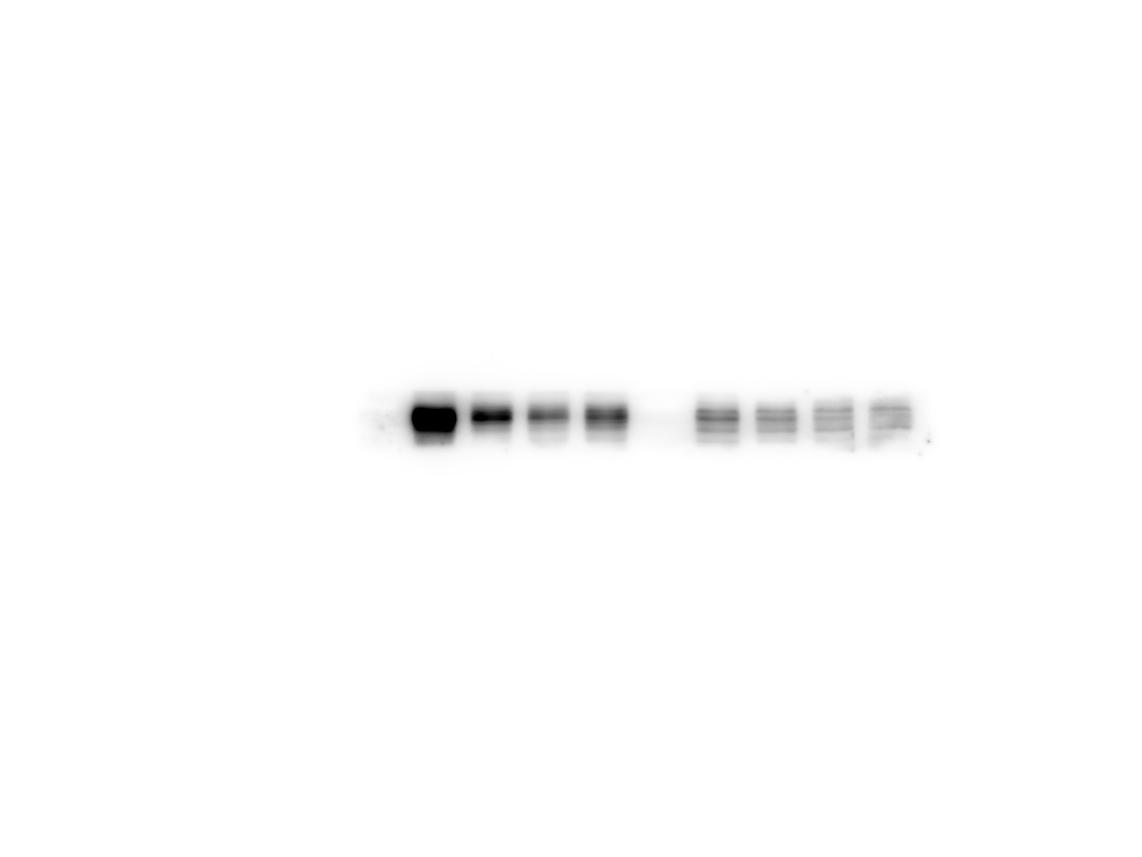

Supplement: Supplementary file 19 — Figure EV7 Source Data [file 44321_2026_414_MOESM19_ESM.zip › Fig. EV7/EV7F/BMAL2 IB.tif]

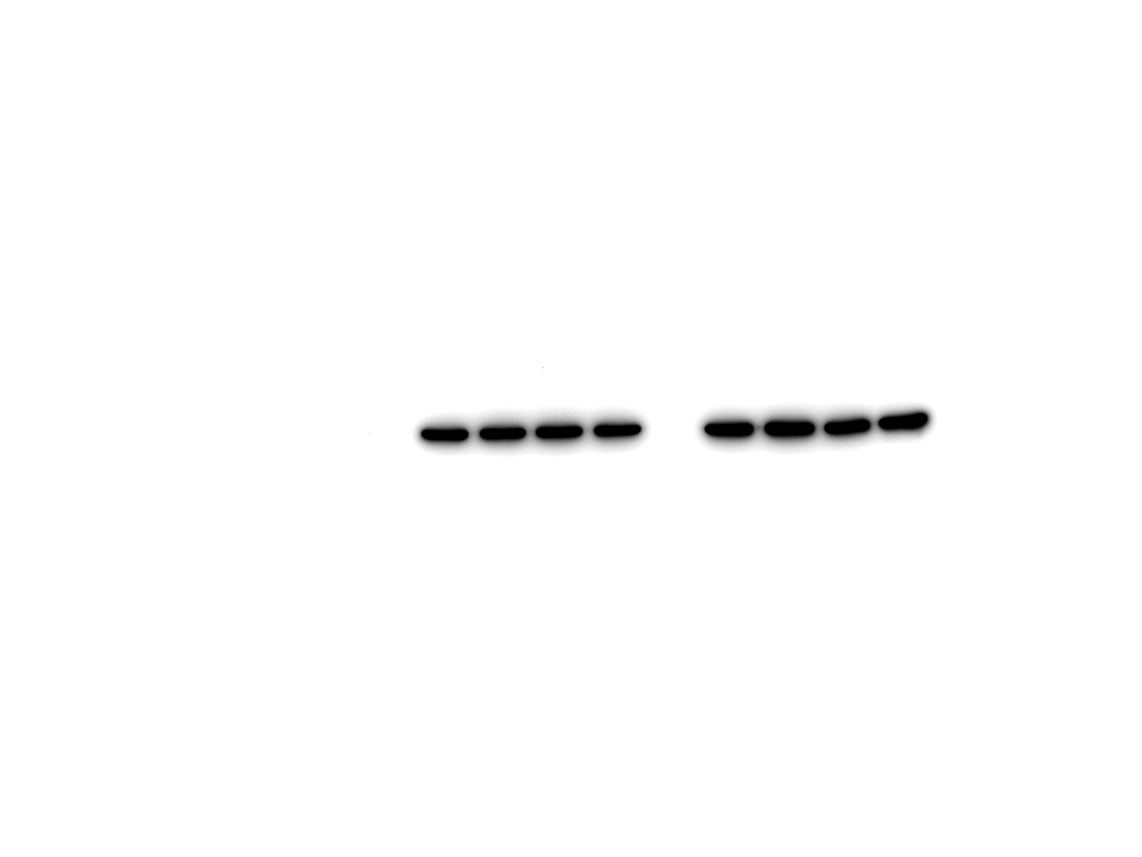

Supplement: Supplementary file 19 — Figure EV7 Source Data [file 44321_2026_414_MOESM19_ESM.zip › Fig. EV7/EV7F/GAPDH IB.tif]

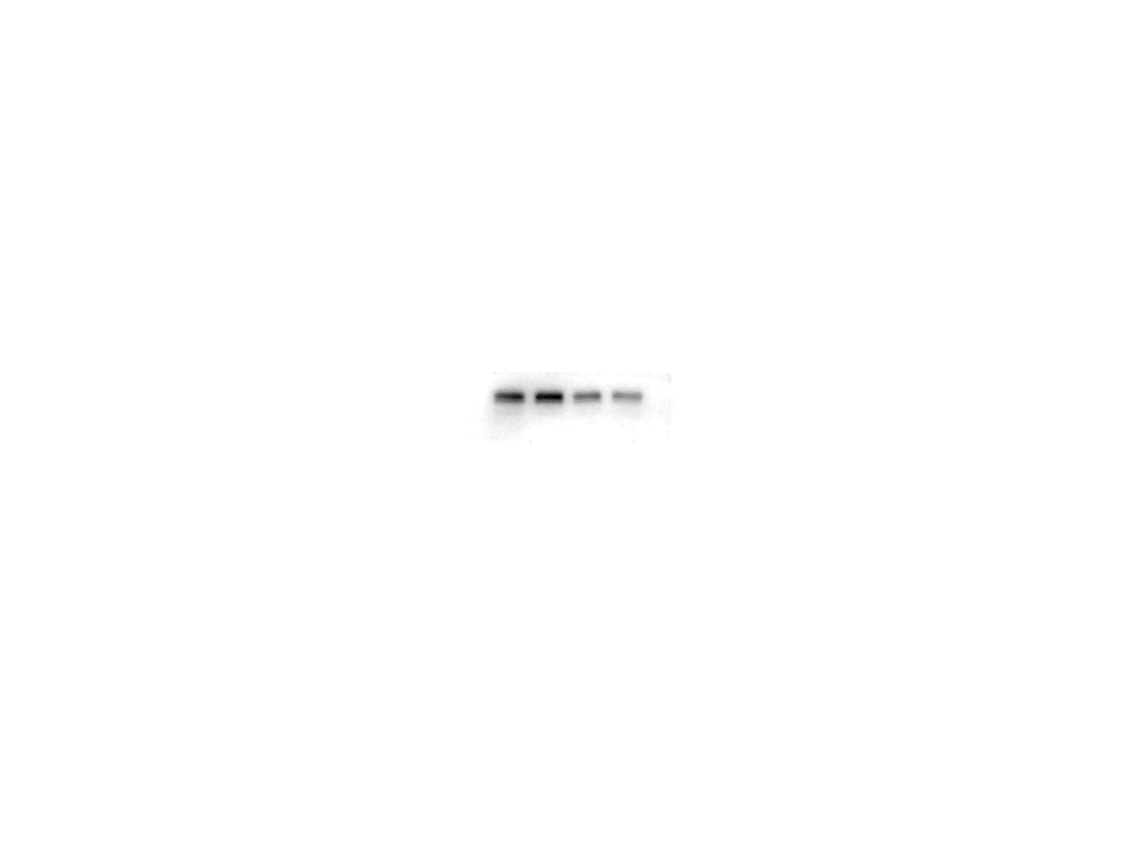

Supplement: Supplementary file 19 — Figure EV7 Source Data [file 44321_2026_414_MOESM19_ESM.zip › Fig. EV7/EV7G/RMG1 BMAL1 IB.tif]

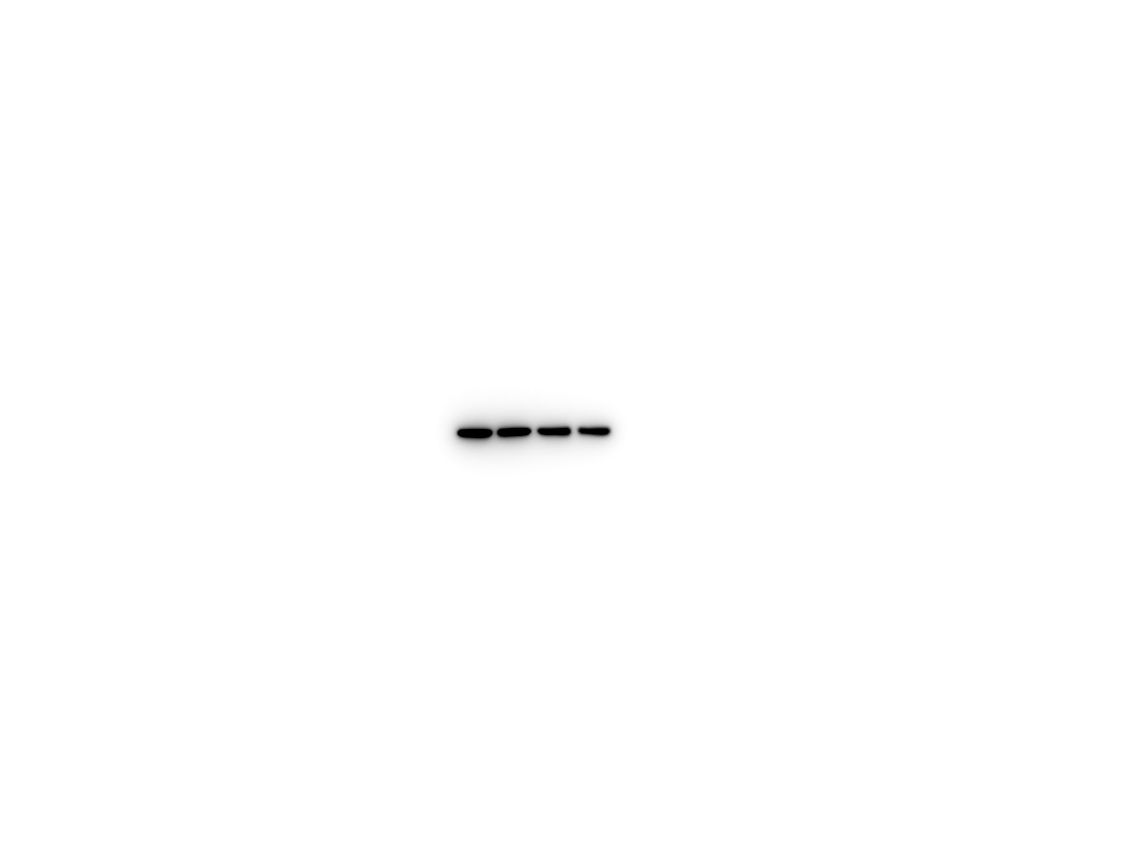

Supplement: Supplementary file 19 — Figure EV7 Source Data [file 44321_2026_414_MOESM19_ESM.zip › Fig. EV7/EV7G/RMG1 GAPDH IB.tif]

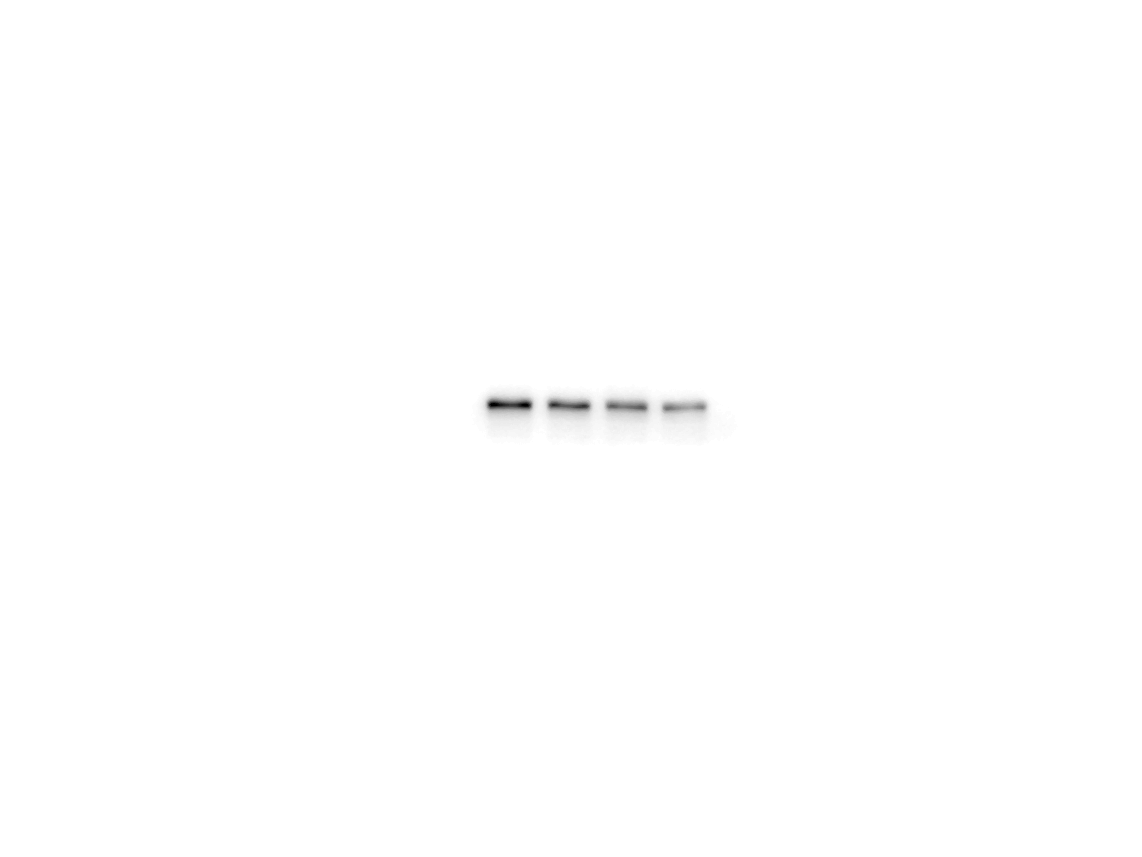

Supplement: Supplementary file 19 — Figure EV7 Source Data [file 44321_2026_414_MOESM19_ESM.zip › Fig. EV7/EV7G/TOV21G BMAL1 IB.tif]

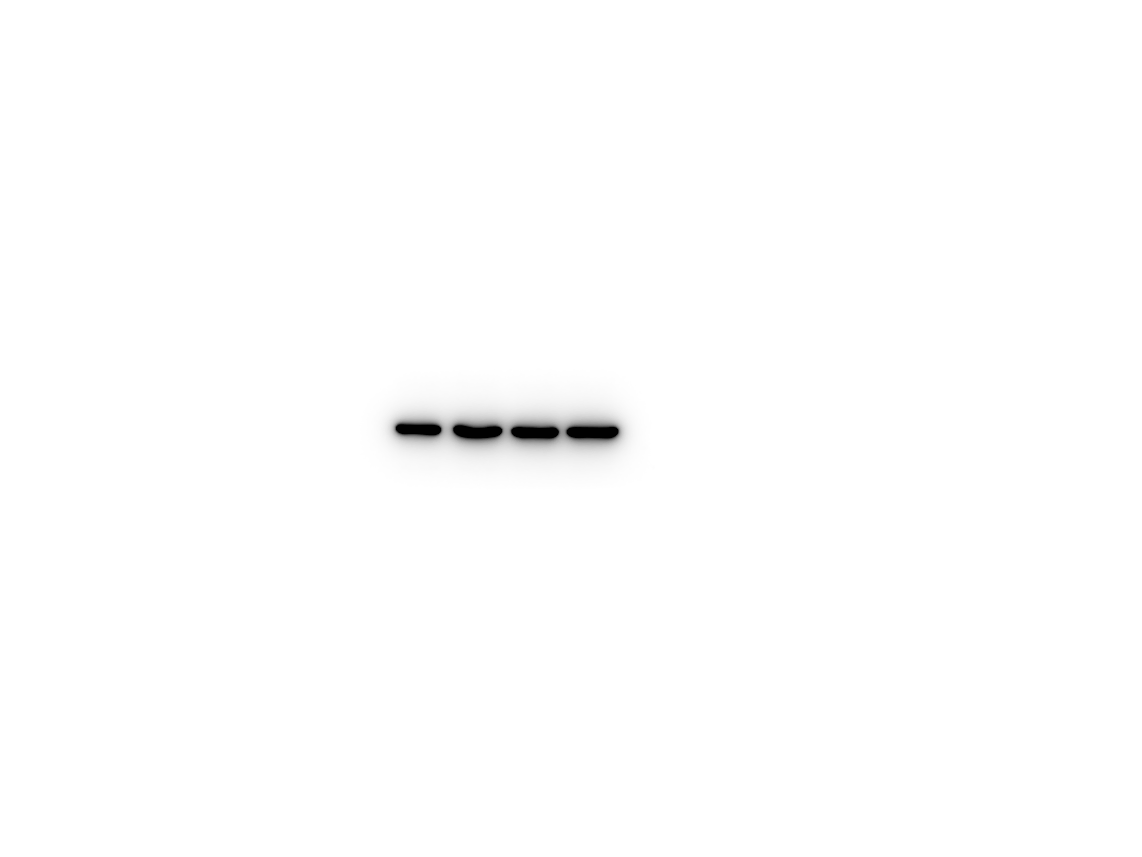

Supplement: Supplementary file 19 — Figure EV7 Source Data [file 44321_2026_414_MOESM19_ESM.zip › Fig. EV7/EV7G/TOV21G GAPDH IB.tif]

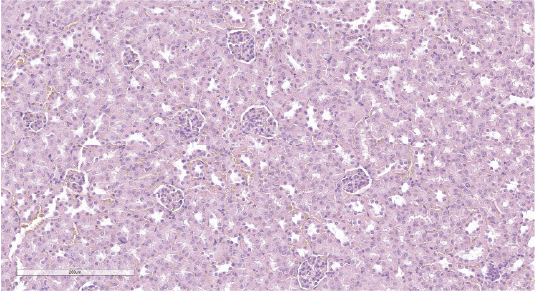

Supplement: Supplementary file 20 — Figure EV8 Source Data [file 44321_2026_414_MOESM20_ESM.zip › Fig. EV8/EV8D/ES-2 GW Kidney HE.png]

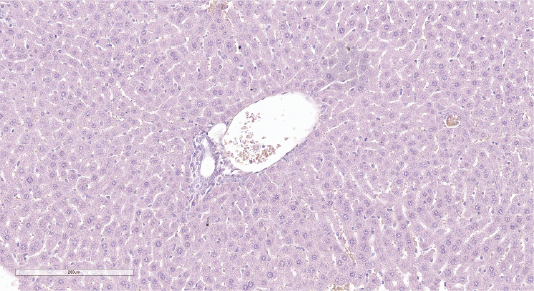

Supplement: Supplementary file 20 — Figure EV8 Source Data [file 44321_2026_414_MOESM20_ESM.zip › Fig. EV8/EV8D/ES-2 GW Liver HE.png]

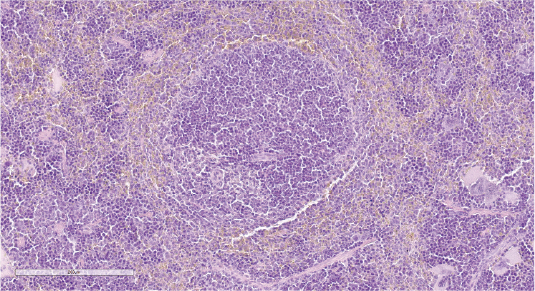

Supplement: Supplementary file 20 — Figure EV8 Source Data [file 44321_2026_414_MOESM20_ESM.zip › Fig. EV8/EV8D/ES-2 GW Spleen HE.png]

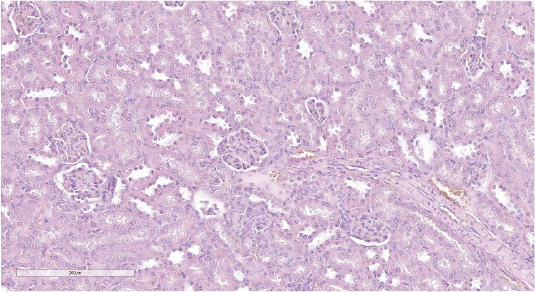

Supplement: Supplementary file 20 — Figure EV8 Source Data [file 44321_2026_414_MOESM20_ESM.zip › Fig. EV8/EV8D/ES-2 Vehicle Kidney HE.png]

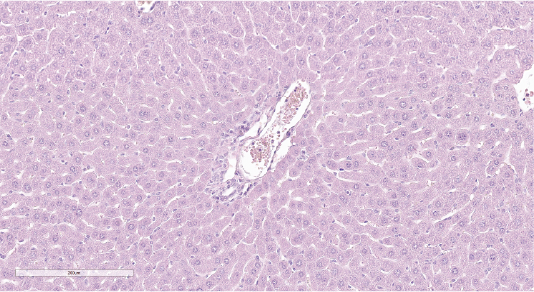

Supplement: Supplementary file 20 — Figure EV8 Source Data [file 44321_2026_414_MOESM20_ESM.zip › Fig. EV8/EV8D/ES-2 Vehicle Liver HE.png]

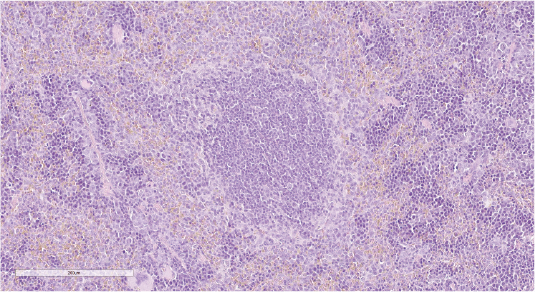

Supplement: Supplementary file 20 — Figure EV8 Source Data [file 44321_2026_414_MOESM20_ESM.zip › Fig. EV8/EV8D/ES-2 Vehicle Spleen HE.png]

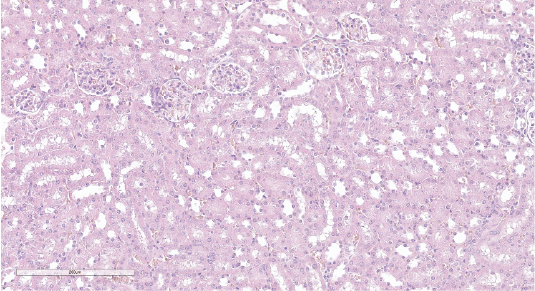

Supplement: Supplementary file 20 — Figure EV8 Source Data [file 44321_2026_414_MOESM20_ESM.zip › Fig. EV8/EV8E/JHOC5 GW Kidney HE.png]

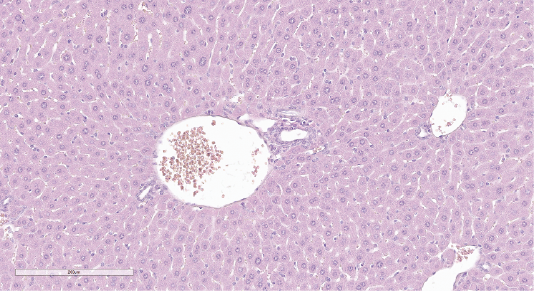

Supplement: Supplementary file 20 — Figure EV8 Source Data [file 44321_2026_414_MOESM20_ESM.zip › Fig. EV8/EV8E/JHOC5 GW Liver HE.png]

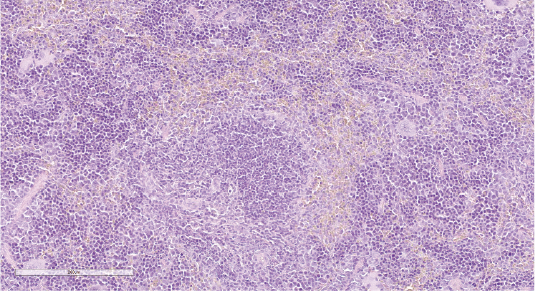

Supplement: Supplementary file 20 — Figure EV8 Source Data [file 44321_2026_414_MOESM20_ESM.zip › Fig. EV8/EV8E/JHOC5 GW Spleen HE.png]

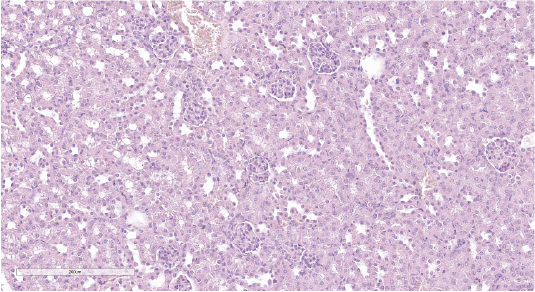

Supplement: Supplementary file 20 — Figure EV8 Source Data [file 44321_2026_414_MOESM20_ESM.zip › Fig. EV8/EV8E/JHOC5 Vehicle Kidney HE.png]

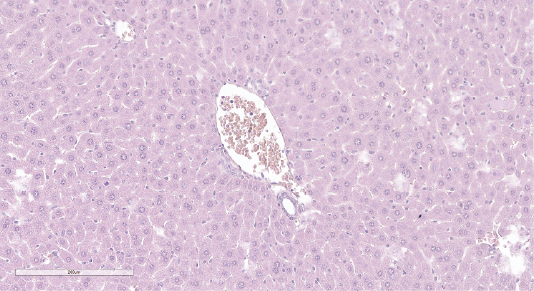

Supplement: Supplementary file 20 — Figure EV8 Source Data [file 44321_2026_414_MOESM20_ESM.zip › Fig. EV8/EV8E/JHOC5 Vehicle Liver HE.png]

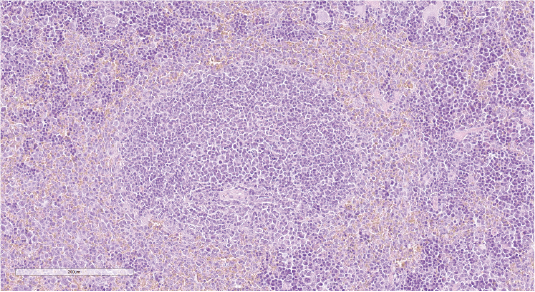

Supplement: Supplementary file 20 — Figure EV8 Source Data [file 44321_2026_414_MOESM20_ESM.zip › Fig. EV8/EV8E/JHOC5 Vehicle Spleen HE.png]

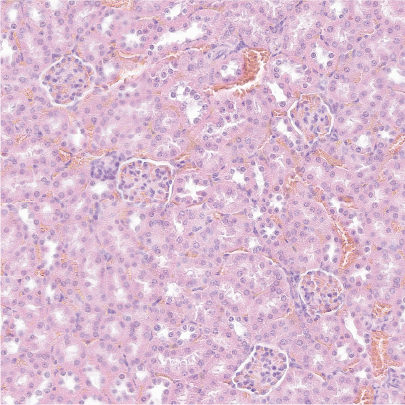

Supplement: Supplementary file 20 — Figure EV8 Source Data [file 44321_2026_414_MOESM20_ESM.zip › Fig. EV8/EV8F/ES-2 10mg GW Kidney HE.png]

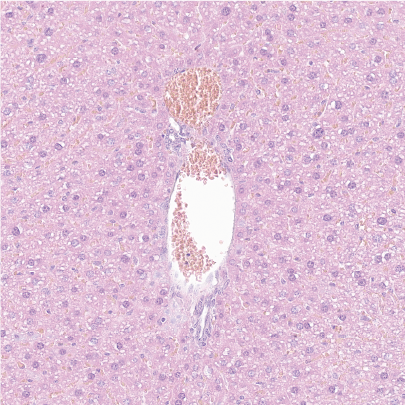

Supplement: Supplementary file 20 — Figure EV8 Source Data [file 44321_2026_414_MOESM20_ESM.zip › Fig. EV8/EV8F/ES-2 10mg GW Liver HE.png]

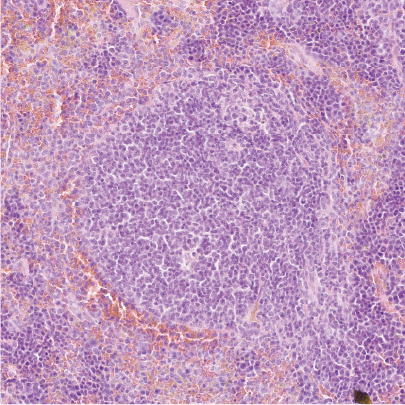

Supplement: Supplementary file 20 — Figure EV8 Source Data [file 44321_2026_414_MOESM20_ESM.zip › Fig. EV8/EV8F/ES-2 10mg GW Spleen HE.png]

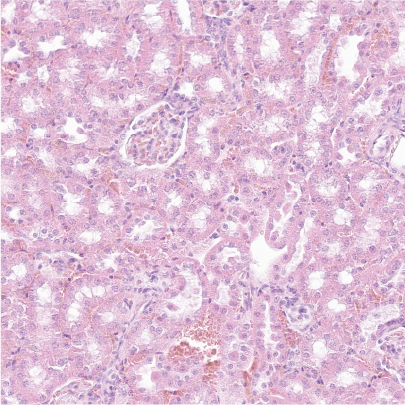

Supplement: Supplementary file 20 — Figure EV8 Source Data [file 44321_2026_414_MOESM20_ESM.zip › Fig. EV8/EV8F/ES-2 10mg Olaparib 10mg GW Kidney HE.png]

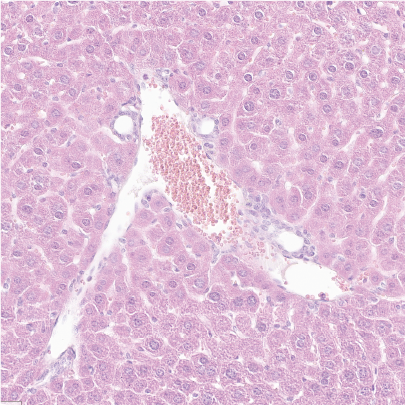

Supplement: Supplementary file 20 — Figure EV8 Source Data [file 44321_2026_414_MOESM20_ESM.zip › Fig. EV8/EV8F/ES-2 10mg Olaparib 10mg GW Liver HE.png]

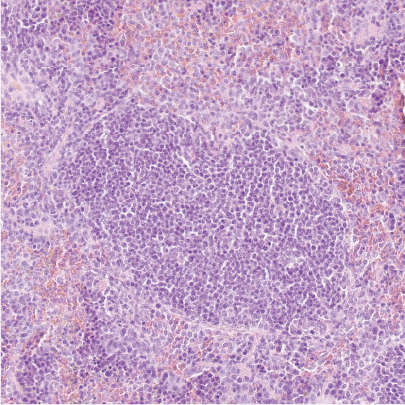

Supplement: Supplementary file 20 — Figure EV8 Source Data [file 44321_2026_414_MOESM20_ESM.zip › Fig. EV8/EV8F/ES-2 10mg Olaparib 10mg GW Spleen HE.png]

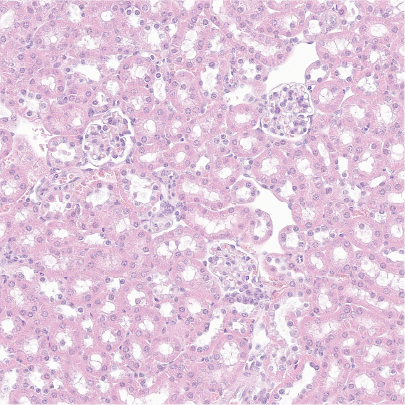

Supplement: Supplementary file 20 — Figure EV8 Source Data [file 44321_2026_414_MOESM20_ESM.zip › Fig. EV8/EV8F/ES-2 10mg Olaparib 20mg GW Kidney HE.png]

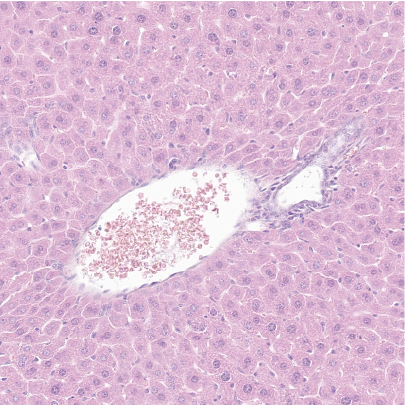

Supplement: Supplementary file 20 — Figure EV8 Source Data [file 44321_2026_414_MOESM20_ESM.zip › Fig. EV8/EV8F/ES-2 10mg Olaparib 20mg GW Liver HE.png]

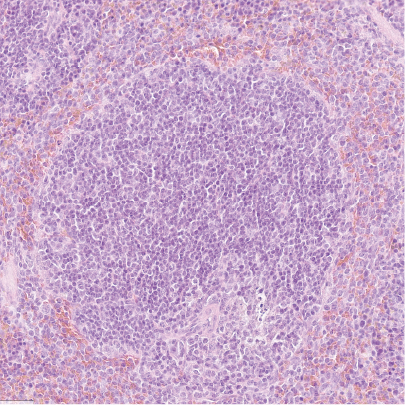

Supplement: Supplementary file 20 — Figure EV8 Source Data [file 44321_2026_414_MOESM20_ESM.zip › Fig. EV8/EV8F/ES-2 10mg Olaparib 20mg GW Spleen HE.png]

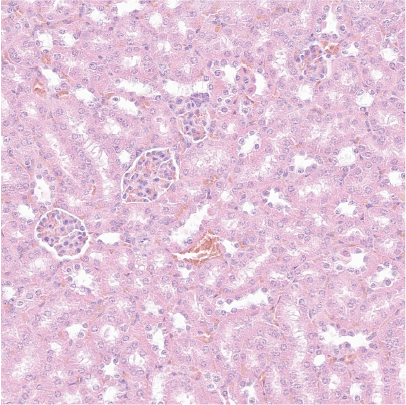

Supplement: Supplementary file 20 — Figure EV8 Source Data [file 44321_2026_414_MOESM20_ESM.zip › Fig. EV8/EV8F/ES-2 10mg Olaparib Kidney HE.png]

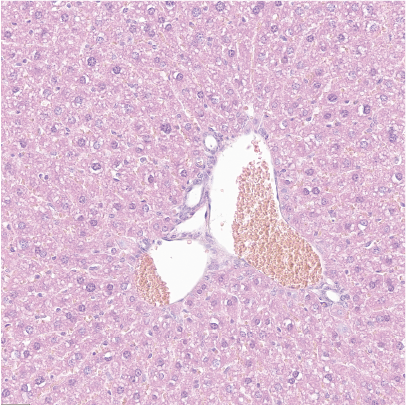

Supplement: Supplementary file 20 — Figure EV8 Source Data [file 44321_2026_414_MOESM20_ESM.zip › Fig. EV8/EV8F/ES-2 10mg Olaparib Liver HE.png]

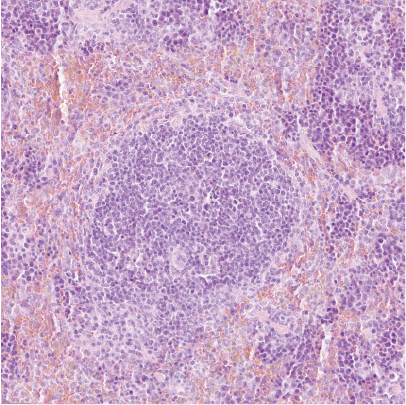

Supplement: Supplementary file 20 — Figure EV8 Source Data [file 44321_2026_414_MOESM20_ESM.zip › Fig. EV8/EV8F/ES-2 10mg Olaparib Spleen HE.png]

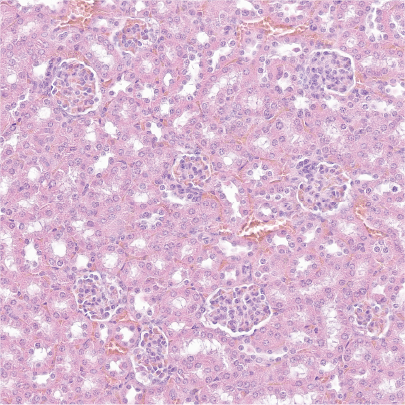

Supplement: Supplementary file 20 — Figure EV8 Source Data [file 44321_2026_414_MOESM20_ESM.zip › Fig. EV8/EV8F/ES-2 20mg GW Kidney HE.png]

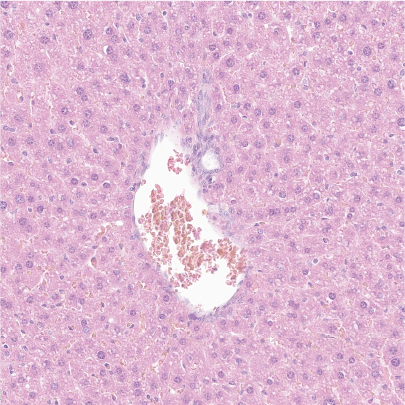

Supplement: Supplementary file 20 — Figure EV8 Source Data [file 44321_2026_414_MOESM20_ESM.zip › Fig. EV8/EV8F/ES-2 20mg GW Liver HE.png]

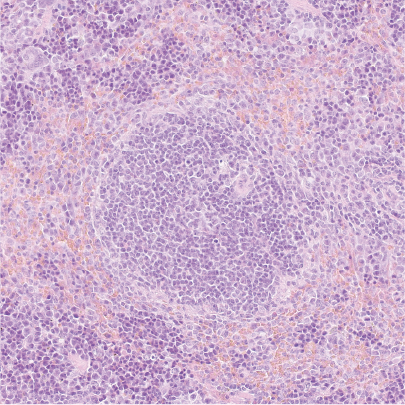

Supplement: Supplementary file 20 — Figure EV8 Source Data [file 44321_2026_414_MOESM20_ESM.zip › Fig. EV8/EV8F/ES-2 20mg GW Spleen HE.png]

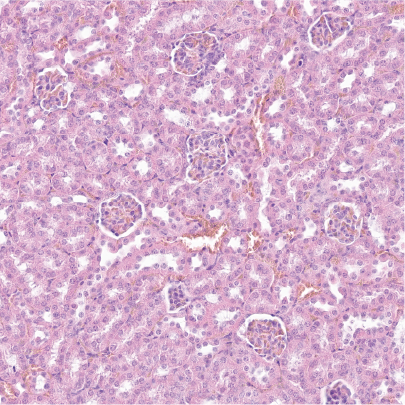

Supplement: Supplementary file 20 — Figure EV8 Source Data [file 44321_2026_414_MOESM20_ESM.zip › Fig. EV8/EV8F/ES-2 Vehicle Kidney HE.png]

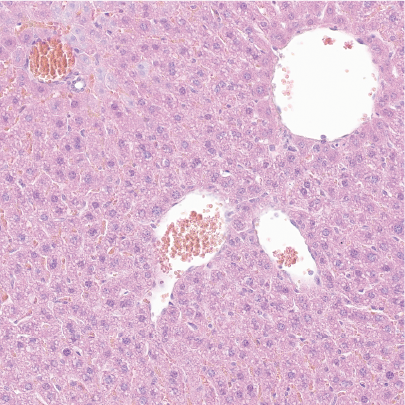

Supplement: Supplementary file 20 — Figure EV8 Source Data [file 44321_2026_414_MOESM20_ESM.zip › Fig. EV8/EV8F/ES-2 Vehicle Liver HE.png]

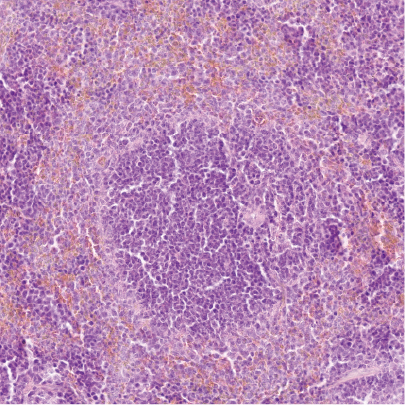

Supplement: Supplementary file 20 — Figure EV8 Source Data [file 44321_2026_414_MOESM20_ESM.zip › Fig. EV8/EV8F/ES-2 Vehicle Spleen HE.png]

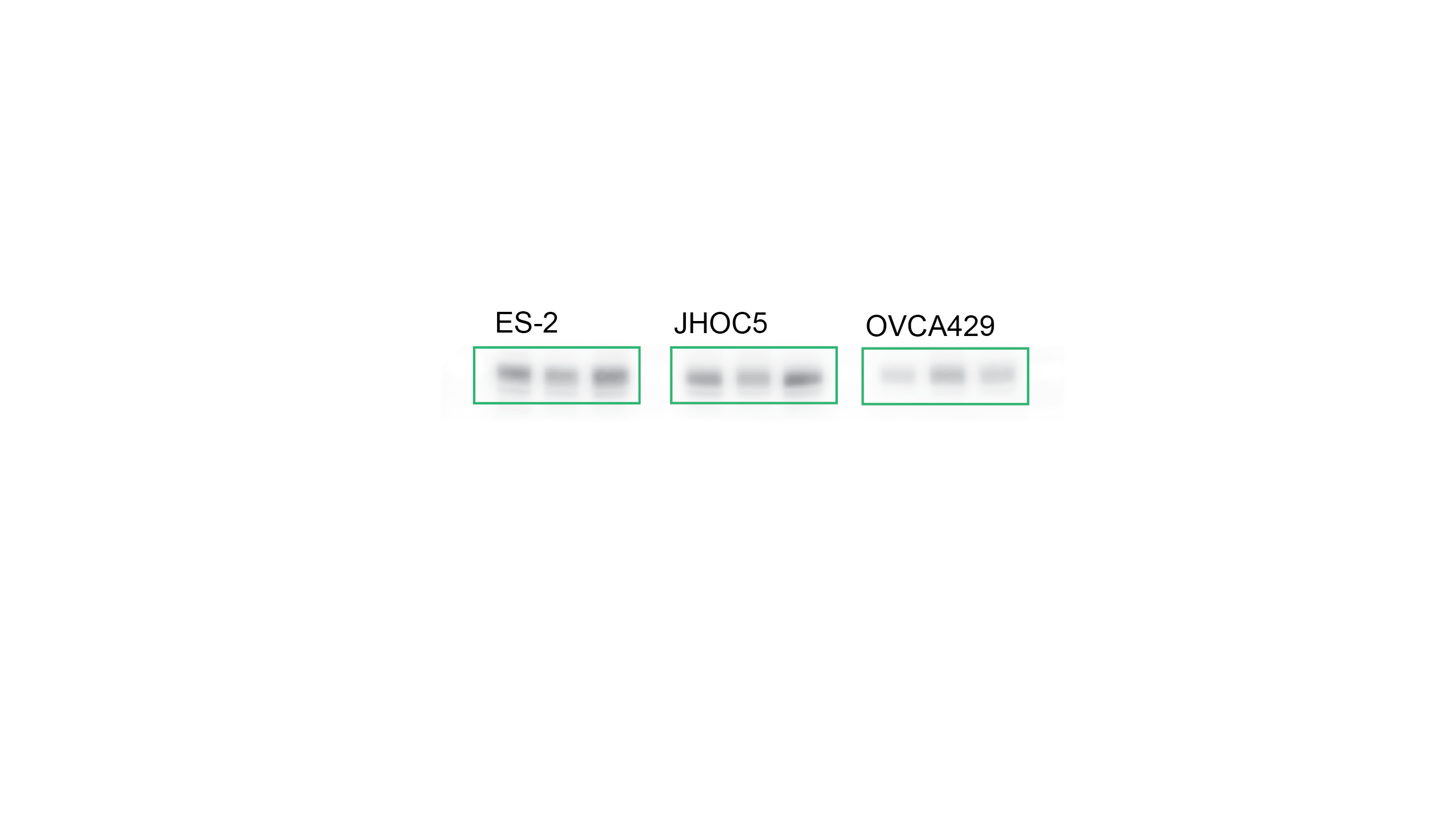

Supplement: Supplementary file 21 — Figure EV9 Source Data [file 44321_2026_414_MOESM21_ESM.zip › Fig. EV9/EV9A/ES-2, JHOC5, OVCA429 BMAL1 IB.png]

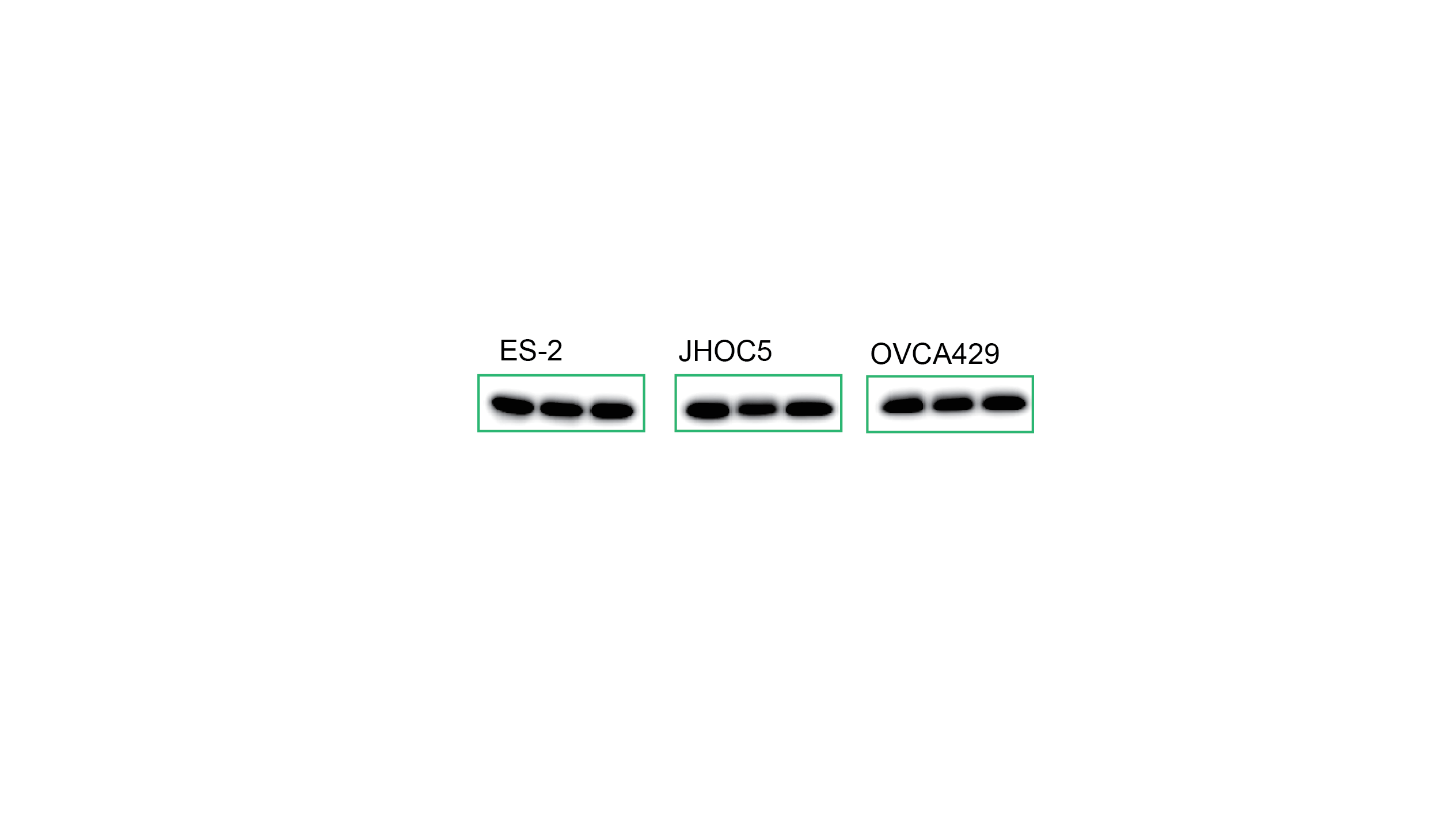

Supplement: Supplementary file 21 — Figure EV9 Source Data [file 44321_2026_414_MOESM21_ESM.zip › Fig. EV9/EV9A/ES-2, JHOC5, OVCA429 GAPDH IB.png]

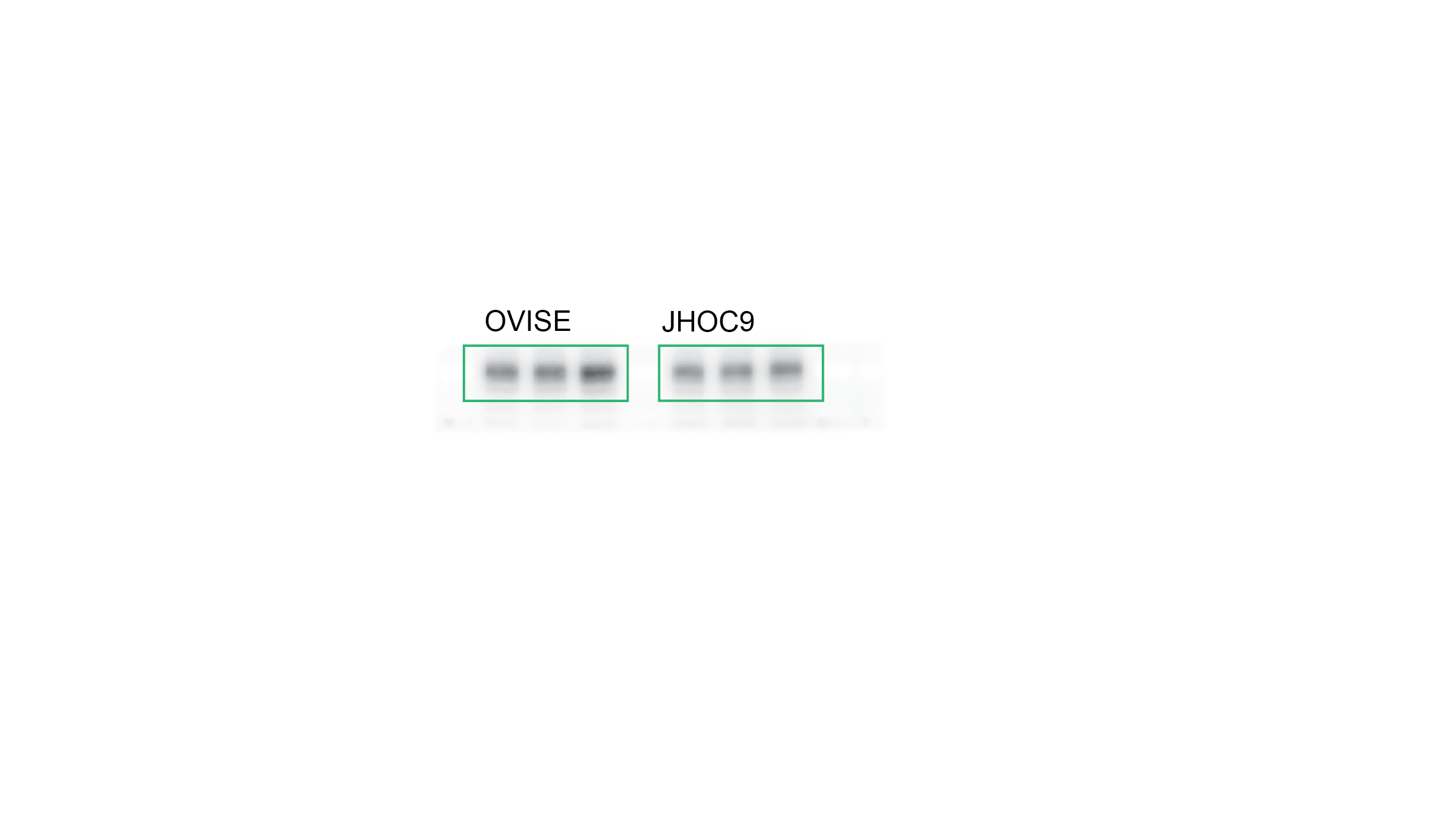

Supplement: Supplementary file 21 — Figure EV9 Source Data [file 44321_2026_414_MOESM21_ESM.zip › Fig. EV9/EV9A/OVISE, JHOC9 BMAL1 IB.png]

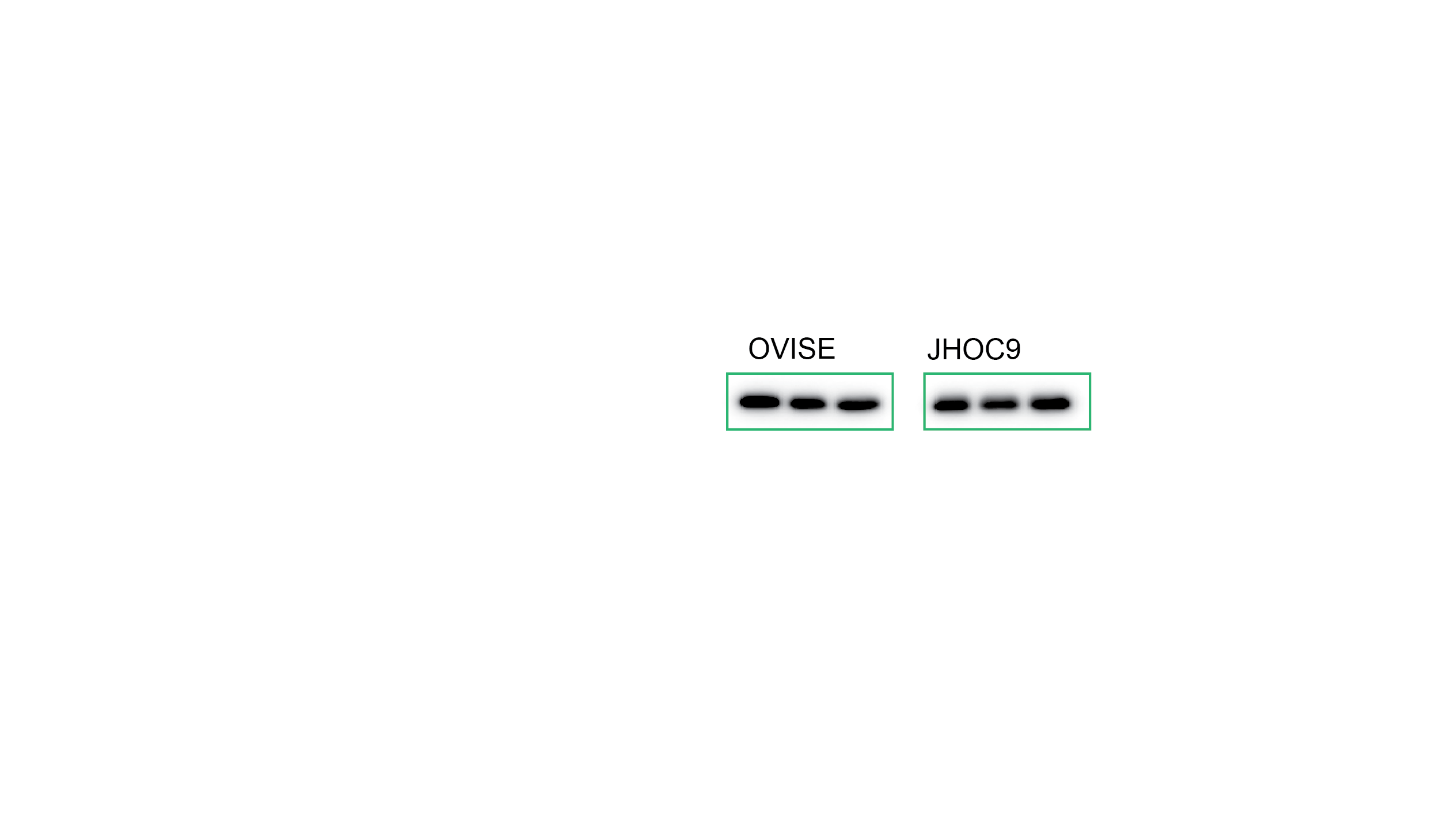

Supplement: Supplementary file 21 — Figure EV9 Source Data [file 44321_2026_414_MOESM21_ESM.zip › Fig. EV9/EV9A/OVISE, JHOC9 GAPDH IB.png]
