# Supplementary material for: BMAL2 is a druggable target for ovarian clear cell carcinoma (OCCC)
Source: EMBO Mol Med. 2026 Apr 3;18(5):1933–66. doi: 10.1038/s44321-026-00414-8 (PMC13179388; doi:10.1038/s44321-026-00414-8)
Supplement: Supplementary file 22 — Expanded View Figures [file 44321_2026_414_MOESM22_ESM.pdf]

Expanded View Figures

Figure EV1. BMAL2 depletion inhibits tumorigenic ability in OCCC cells.

(**A**) Representative EdU staining of OCCC cells without (shCtrl) or with BMAL2 depletion (shBMAL2#1 or #2). Scale bar indicates 100 µm. (**B**) Representative phospho-histone H3 (pHH3) staining of shCtrl and shBMAL2 OCCC cells. Scale bar indicates 200 µm. (**C**) Representative images of clonogenic assays in OCCC cells. (**D**) Individual 3D ultrasound tumor images from xenograft models in NUDE mice using shCtrl or shBMAL2 ES-2, JHOC5 and OVISE cells. Five mice were used for each group. The size of each tumor is indicated. (**E**) Representative images of BMAL2 and Ki67 IHC staining using serial tumor sections from ES-2, JHOC5 or OVISE derived tumors. Scale bars indicate 60 µm. Source data are available online for this figure.

© The Author(s)

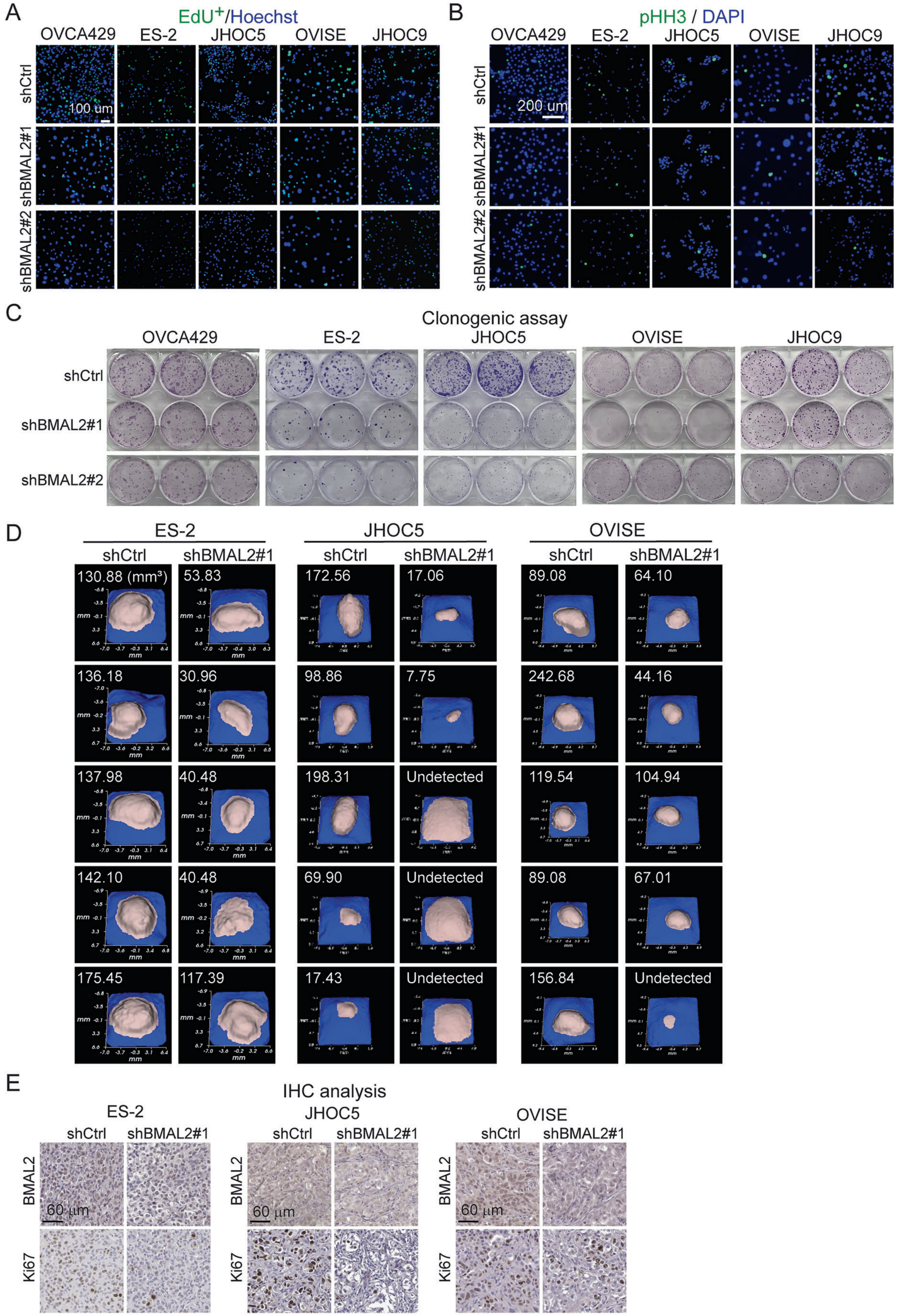
A
EdU+/Hoechst
OVCA429
ES-2
JHOC5
OVISE
JHOC9
shCtrl
shBMAL2#1
shBMAL2#2
100 um
B
pHH3 / DAPI
200 um
C
Clonogenic assay
D
130.88 (mm³)
53.83
172.56
17.06
89.08
64.10
136.18
30.96
98.86
7.75
242.68
44.16
137.98
40.48
198.31
Undetected
119.54
104.94
142.10
40.48
69.90
Undetected
89.08
67.01
175.45
117.39
17.43
Undetected
156.84
Undetected
E
IHC analysis
BMAL2
Ki67
60 μm

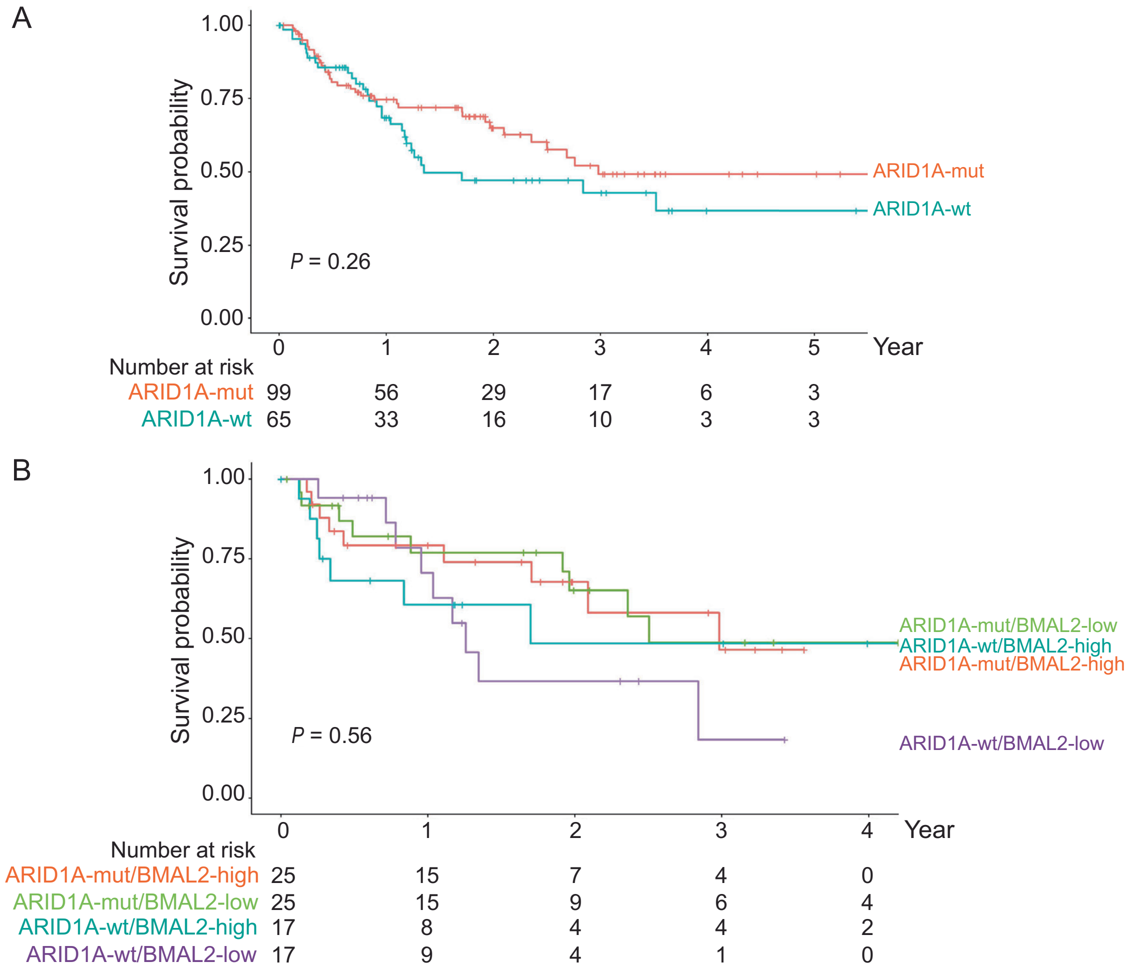

Figure EV2. BMAL2 function may be independent of ARID1A status in OCCC.

(**A**) Kaplan–Meier overall survival (OS) analysis of OCCC patients grouped by ARID1A status. ARID1A-mut group ($n = 99$) is indicated by the red line; ARID1A-wt group ($n = 65$) is indicated by the cyan line. $P = 0.26$. The P value was determined by the log-rank test. (**B**) Kaplan–Meier OS analysis of ARID1A-mut and ARID1A-wt OCCC patients grouped by BMAL2 expression level. The first (BMAL2-high) and fourth (BMAL2-low) quartiles, both within and between ARID1A-wt ($n = 17$ for each group) and ARID1A-mut ($n = 25$ for each group) cases, were compared. ARID1A-mut/BMAL2-high group ($n = 25$) is indicated by red line; ARID1A-mut/BMAL2-low group ($n = 25$) is indicated by green line; ARID1A-wt/BMAL2-high group ($n = 17$) is indicated by cyan line; ARID1A-wt/BMAL2-low group ($n = 17$) is indicated by purple line. $P = 0.56$. The P value was determined by the log-rank test.

© The Author(s)

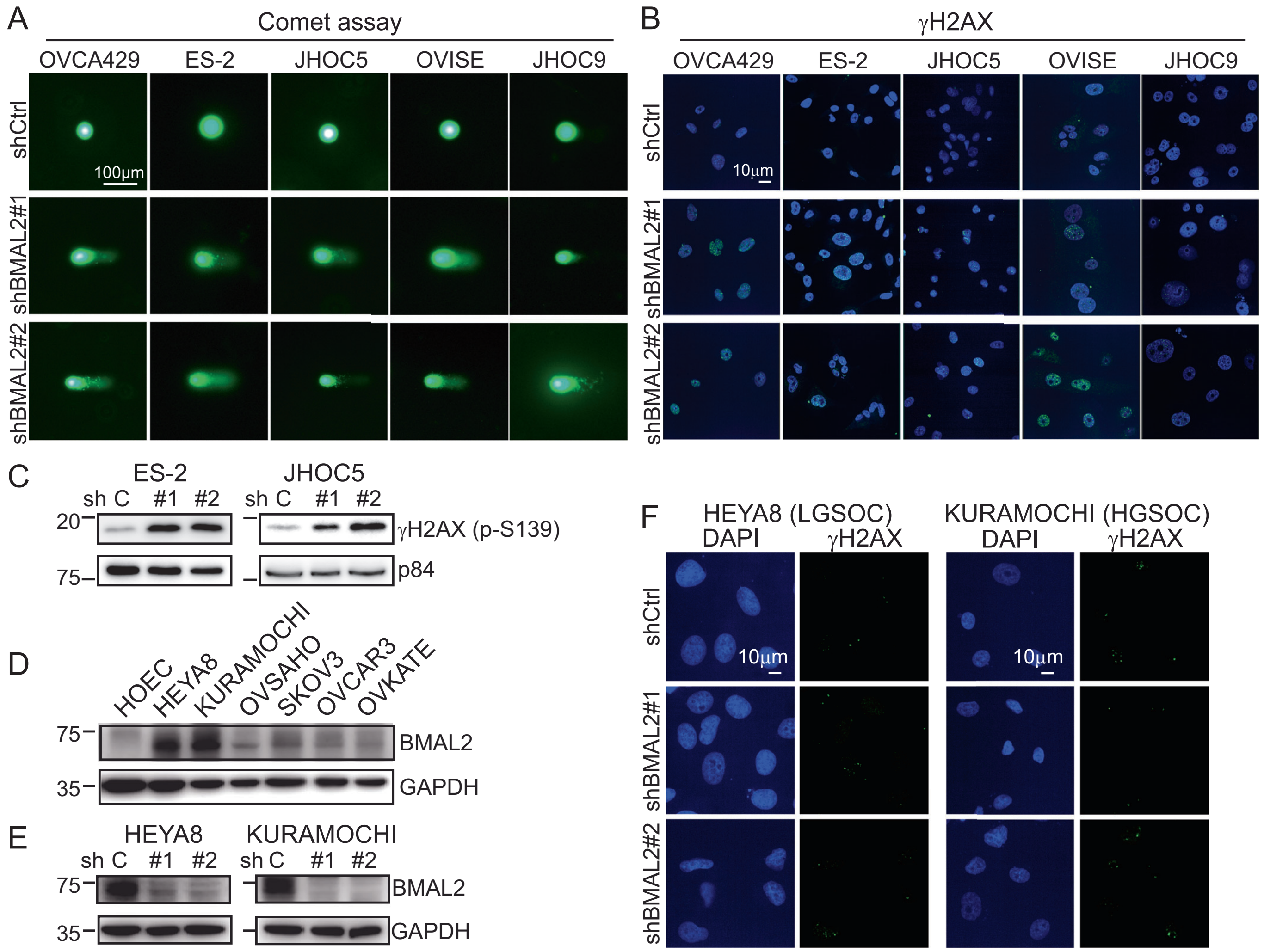

Figure EV3. BMAL2 depletion increases endogenous DNA damage in OCCC cells, but not serous type ovarian cancers.

(**A**) Representative comet assay images of OCCC cells without (shCtrl) or with BMAL2 depletion (shBMAL2#1 or #2). These cells were not treated with DNA damage agents. Scale bar indicates 100 μm. (**B**) Representative γH2AX staining of shCtrl and shBMAL2 OCCC cells. These cells were not treated with DNA damage agents. Scale bar indicates 10 μm. (**C**) IB of γH2AX protein with p84 as a nuclear protein loading control. Blots shown are from one representative experiment of three replicates. (**D**) IB of BMAL2 protein expression with GAPDH as a loading control in serous ovarian cancer cell lines. Blots shown are from one representative experiment of three replicates. (**E**) IB of BMAL2 protein expression with GAPDH as a loading control in BMAL2-depleted (shBMAL2#1 or #2) HEYA8 and KURAMOCHI cells. Blots shown are from one representative experiment of three replicates. (**F**) Representative γH2AX staining of shCtrl and shBMAL2 serous type OC cells. These cells were not treated with DNA damage agents. Scale bars indicate 10 μm. Source data are available online for this figure.

 © The Author(s)

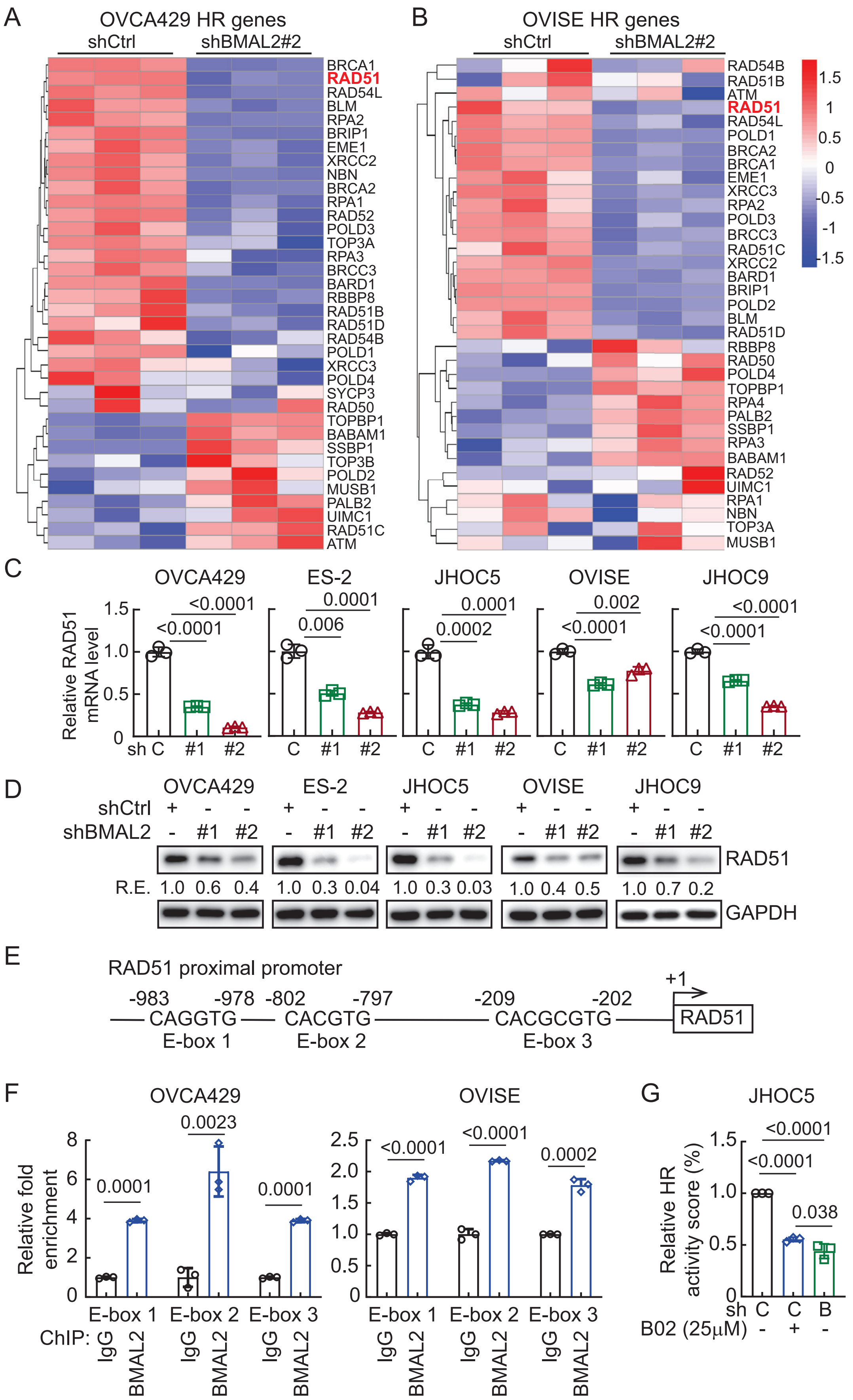
A
OVCA429 HR genes
shCtrl
shBMAL2#2
BRCA1
RAD51
RAD54L
BLM
RPA2
BRIP1
EME1
XRCC2
NBN
BRCA2
RPA1
RAD52
POLD3
TOP3A
RPA3
BRCC3
BARD1
RBBP8
RAD51B
RAD51D
RAD54B
POLD1
XRCC3
POLD4
SYCP3
RAD50
TOPBP1
BABAM1
SSBP1
TOP3B
POLD2
MUSB1
PALB2
UIMC1
RAD51C
ATM
B
OVISE HR genes
shCtrl
shBMAL2#2
RAD54B
RAD51B
ATM
RAD51
RAD54L
POLD1
BRCA2
BRCA1
EME1
XRCC3
RPA2
POLD3
BRCC3
RAD51C
XRCC2
BARD1
BRIP1
POLD2
BLM
RAD51D
RBBP8
RAD50
POLD4
TOPBP1
RPA4
PALB2
SSBP1
RPA3
BABAM1
RAD52
UIMC1
RPA1
NBN
TOP3A
MUSB1
1.5
1
0.5
0
-0.5
-1
-1.5
C
OVCA429
ES-2
JHOC5
OVISE
JHOC9
Relative RAD51 mRNA level
<0.0001
<0.0001
0.0001
0.006
0.0001
0.0002
0.002
<0.0001
<0.0001
<0.0001
sh C #1 #2
D
OVCA429
ES-2
JHOC5
OVISE
JHOC9
shCtrl
shBMAL2
#1 #2
RAD51
R.E. 1.0 0.6 0.4 1.0 0.3 0.04 1.0 0.3 0.03 1.0 0.4 0.5 1.0 0.7 0.2
GAPDH
E
RAD51 proximal promoter
-983 -978 -802 -797 -209 -202 +1
CAGGTG
CACGTG
CACGCGTG
RAD51
E-box 1
E-box 2
E-box 3
F
OVCA429
OVISE
Relative fold enrichment
0.0001
0.0023
0.0001
<0.0001
<0.0001
0.0002
E-box 1 E-box 2 E-box 3
ChIP: IgG BMAL2
G
JHOC5
Relative HR activity score (%)
<0.0001
<0.0001
0.038
sh C C B
B02 (25μM) - + -

Figure EV4. BMAL2 depletion downregulates genes in the homologous recombination (HR) pathway, including RAD51.

(**A**, **B**) Heatmap of HR genes of the KEGG pathway (hsa03440) in OVCA429 (**A**) and OVISE (**B**) cells without or with BMAL2 depletion. (**C**) qRT-PCR analysis of RAD51 level in OCCC cells without (shCtrl) or with BMAL2 depletion (shBMAL2#1 or #2). RNA18S5 was used as an internal control. Three independent experiments were performed, and data were means ± SD from one representative experiment ($n = 3$). ***$P < 0.001$; ****$P < 0.0001$. Significant differences are based on an unpaired *t*-test. (**D**) IB of RAD51 protein expression with GAPDH as a loading control in shCtrl or shBMAL2 OCCC cells. Blots shown are from one representative experiment of three replicates. RE relative expression. (**E**) Diagram shows three putative E-boxes on the *RAD51* promoter predicted using the EPD eukaryotic promoter database. (**F**) ChIP-qPCR analysis of BMAL2 on the *RAD51* promoter E-box regions. Three independent experiments were performed, and data are means ± SD from one representative experiment with significant differences detected by an unpaired *t*-test. ***$P < 0.001$; ****$P < 0.0001$. (**G**) HR activity of shCtrl or shBMAL2 JHOC5 cells, assessed 72 h after adenovirus infection. Data were shown as mean ± SD with *P* value based on one-way ANOVA test ($n = 3$). Source data are available online for this figure.

 © The Author(s)

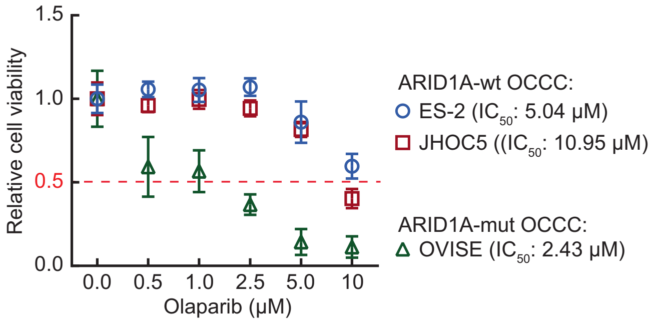

Figure EV5. ARID1A-mut OCCC cells are more sensitive to PARPi than ARID1A-wt OCCC cells.

Cell viability assays using ES-2, JHOC5 and OVISE cells treated with vehicle (0), 0.5, 1, 2.5, 5, or 10 μM Olaparib. Data were shown as mean ± SD ($n = 3$). The half-maximal inhibitory concentration (IC_{50}) of Olaparib for each cell line is indicated. Source data are available online for this figure.

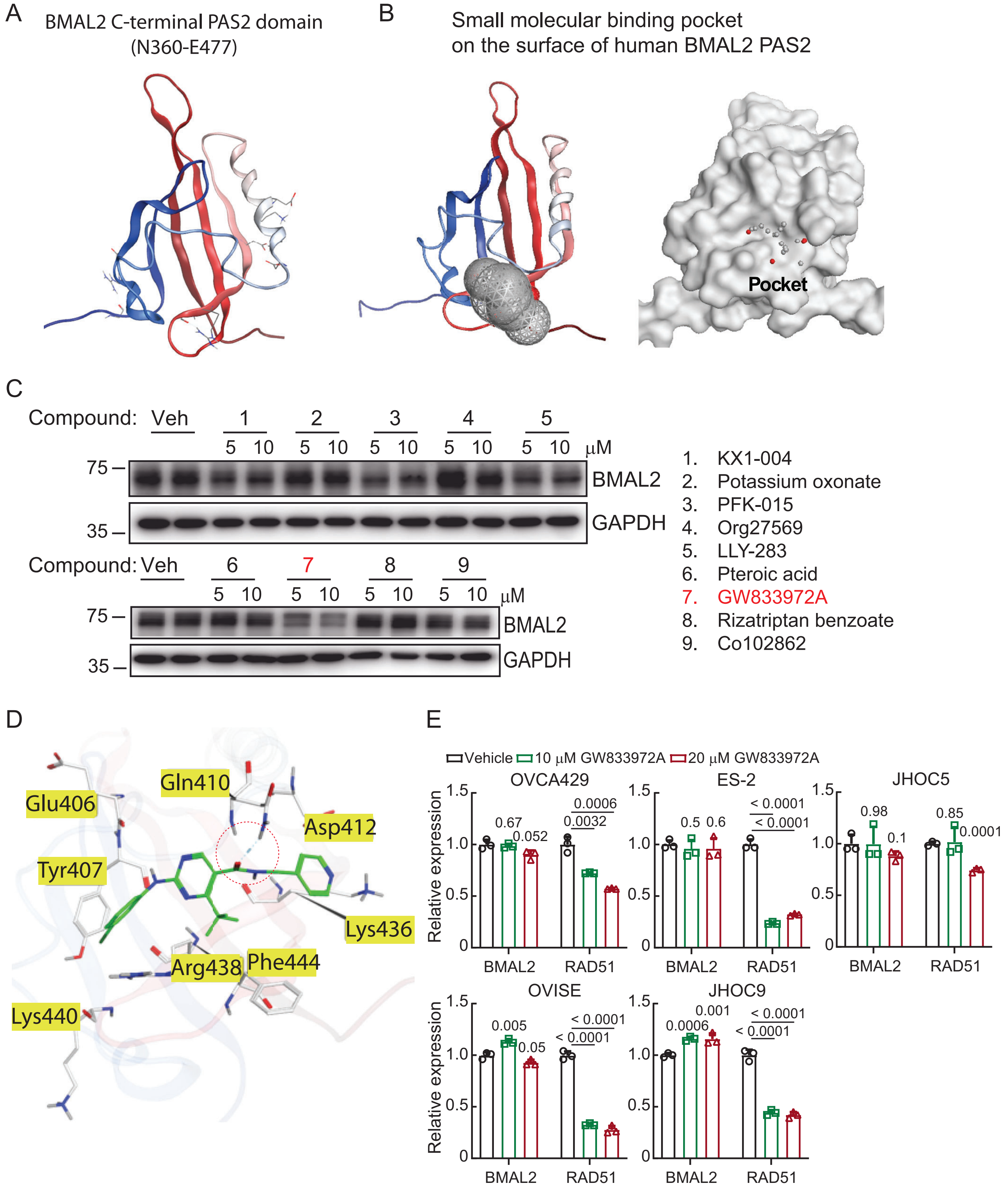
A
BMAL2 C-terminal PAS2 domain
(N360-E477)
B
Small molecular binding pocket
on the surface of human BMAL2 PAS2
Pocket
C
Compound: Veh 1 2 3 4 5
5 10 5 10 5 10 5 10 5 10 μM
75
BMAL2
GAPDH
35
Compound: Veh 6 7 8 9
5 10 5 10 5 10 5 10 μM
75
BMAL2
GAPDH
35
1. KX1-004
2. Potassium oxonate
3. PFK-015
4. Org27569
5. LLY-283
6. Pteroic acid
7. GW833972A
8. Rizatriptan benzoate
9. Co102862
D
Glu406
Gln410
Asp412
Tyr407
Lys436
Arg438
Phe444
Lys440
E
Vehicle
10 μM GW833972A
20 μM GW833972A
OVCA429
ES-2
JHOC5
OVISE
JHOC9
Relative expression
BMAL2
RAD51
0.67
0.052
0.0006
0.0032
0.5
0.6
< 0.0001
0.98
0.1
0.85
0.0001
0.005
0.05
< 0.0001
0.001
0.0006

 © The Author(s)

Figure EV6. Virtual screening of potential small compounds for BMAL2 inhibition.

(**A**) 3D structure of human BMAL2 PAS2 domain (2KDK). The N-terminal to C-terminal structure was colored from blue to red. (**B**) The small molecular binding pocket on the surface of human BMAL2 PAS2 domain was identified using MOE-SiteFinder, where the pocket was served as the docking area for the virtual screening. The docking area was defined by a docking box with the length, width and height of 19.67 Å × 20.33 Å × 16.67 Å respectively, and the total volume was 6664 $Å^3$, where the inner contour volume was 2614 $Å^3$. (**C**) Left panel: IB of BMAL2 protein with GAPDH as a loading control in ES-2 cells treated with vehicle (DMSO), 5 or 10 µM selected compounds. Blots shown are from one representative experiment of three replicates. Right panel: The top nine bioactive compounds with low K_d value used for evaluation. (**D**) Binding modes of GW833972A to human BMAL2. BMAL2-compound complex, in which the interaction of GW833972A with the contact residues of the BMAL2 PAS2 domain were shown. (**E**) qRT-PCR analysis of BMAL2 and RAD51 levels in OCCC cells treated with vehicle, 10 or 20 µM GW833972A. RNA18S5 was used as an internal control. Three independent experiments were performed, and data were means ± SD from one representative experiment ($n = 3$). Significant differences are based on an unpaired t-test. Source data are available online for this figure.

© The Author(s)

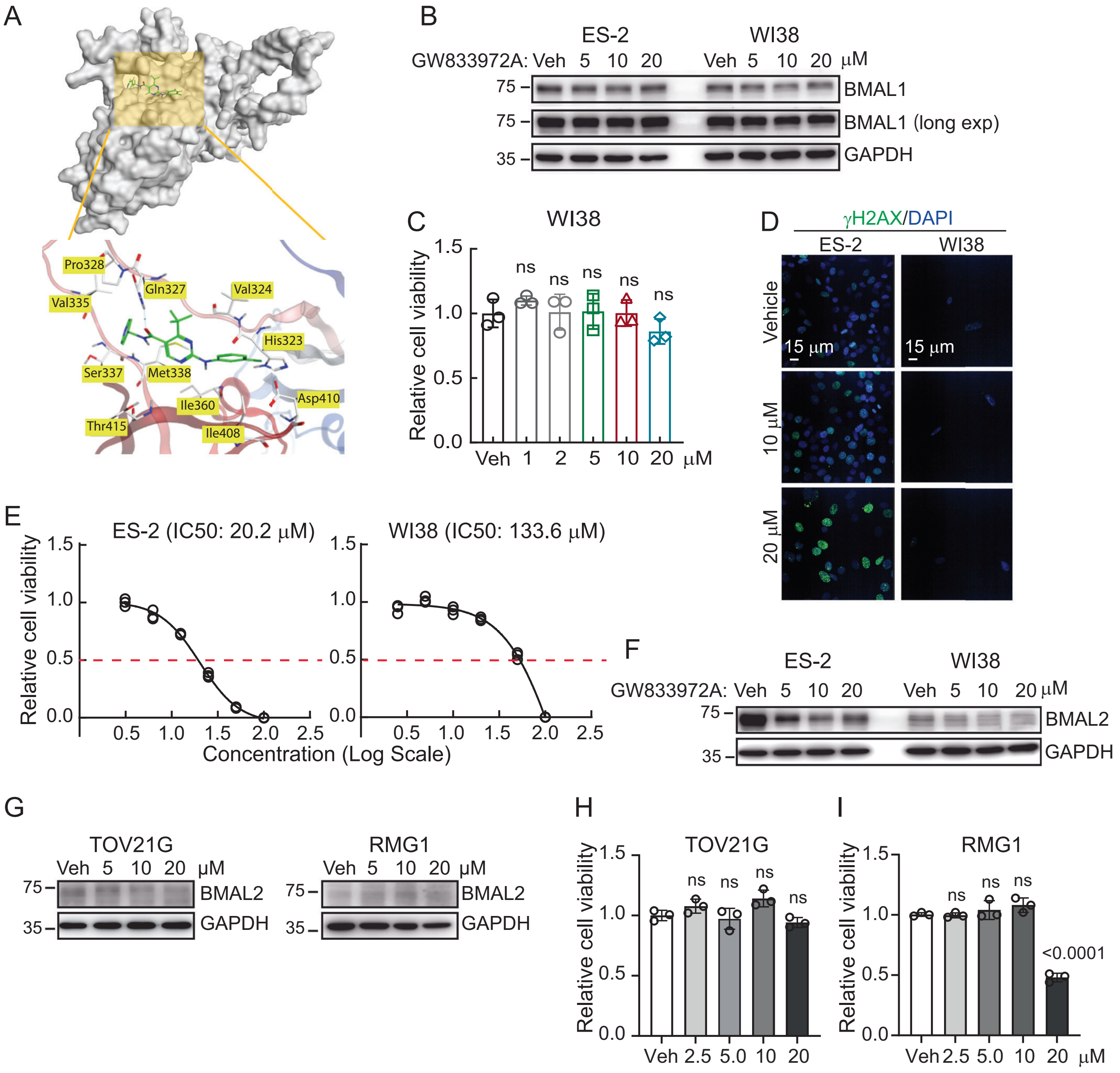

Figure EV7. GW833972A can effectively target BMAL2- expressing cancer cells while having low adverse effects on normal cells.

(**A**) Binding mode of GW833972A to human BMAL1. BMAL1-compound complex, in which the BMAL1 core circadian modulator (CCM) binding pocket in the PAS-B domain was shown as a molecular surface, and GW833972A was shown in green. Interaction of GW833972A to the contact residues of BMAL1 were shown. (**B**) IB of BMAL1 protein with GAPDH as a loading control in ES-2 and WI38 cells treated with vehicle (DMSO), 5, 10, or 20 μM GW833972A. Blots shown are from one representative experiment of three replicates. (**C**) Cell viability assays using WI38 cells treated with vehicle (DMSO), 5, 10, or 20 μM GW833972A. Data were shown as mean ± SD with *P* value based on unpaired *t*-test ($n = 3$). ns not significant. The experiments were repeated three times. (**D**) Representative γH2AX staining of ES-2 and WI38 cells treated with vehicle (DMSO), 10 or 20 μM GW833972A. Scale bar indicates 15 μm. (**E**) Dose-response curves for the assessment of cell viability in ES-2 and WI38 cells treated by GW833972A from 0 to 100 μM. The curves were plotted with log10 [GW833972A (μM)]. IC50 for each cell line was indicated. (**F**) IB of BMAL2 protein with GAPDH as a loading control in ES-2 and WI38 cells treated with vehicle (DMSO), 5, 10, or 20 μM GW833972A. Blots shown are from one representative experiment of three replicates. (**G**) IB of BMAL2 protein with GAPDH as a loading control in TOV21G and RMG1 cells treated with vehicle (DMSO), 5, 10, or 20 μM GW833972A. Blots shown are from one representative experiment of three replicates. (**H**, **I**) Cell viability assays using TOV21G (**H**) and RMG1 (**I**) cells treated with vehicle (DMSO), 5, 10, or 20 μM GW833972A. Data were shown as mean ± SD with *P* value based on unpaired *t*-test ($n = 3$). ns not significant. The experiments were repeated three times. Source data are available online for this figure.

 © The Author(s)

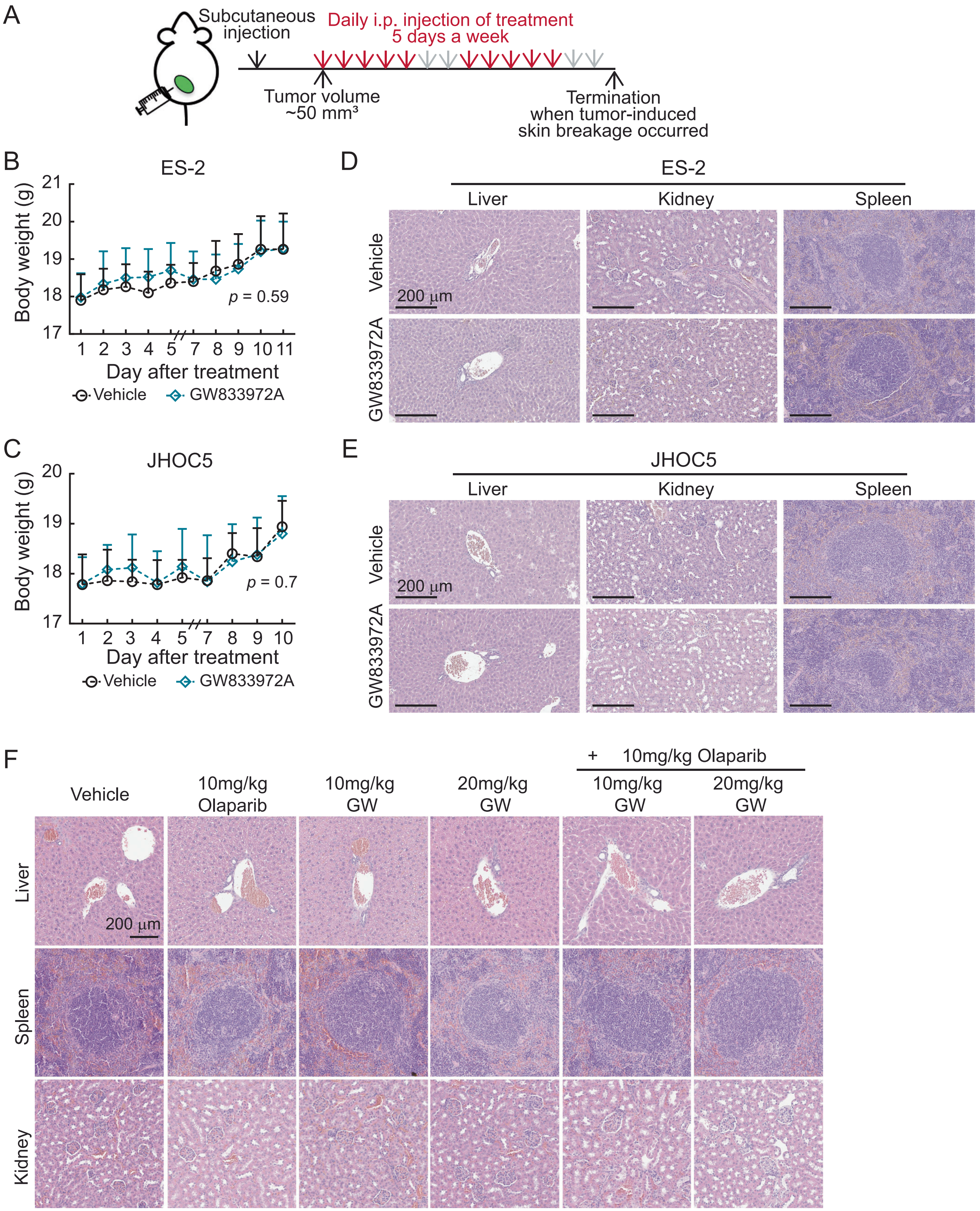
A
Subcutaneous injection
Daily i.p. injection of treatment 5 days a week
Tumor volume ~50 mm³
Termination when tumor-induced skin breakage occurred
B
ES-2
Body weight (g)
p = 0.59
Day after treatment
Vehicle
GW833972A
C
JHOC5
Body weight (g)
p = 0.7
Day after treatment
Vehicle
GW833972A
D
ES-2
Liver
Kidney
Spleen
Vehicle
GW833972A
200 µm
E
JHOC5
Liver
Kidney
Spleen
Vehicle
GW833972A
200 µm
F
+ 10mg/kg Olaparib
Vehicle
10mg/kg Olaparib
10mg/kg GW
20mg/kg GW
10mg/kg GW
20mg/kg GW
Liver
Spleen
Kidney
200 µm

Figure EV8. Subcutaneous xenograft models using ES-2 and JHOC5 cells.

(**A**) Diagram of the procedure used for subcutaneous xenografts. 2×10^6 ES-2 or 5×10^6 JHOC5 cells mixed with Matrigel in a 1:1 ratio were subcutaneously injected into the lower flanks of the mouse. When tumors reached 50 mm^3, mice were intraperitoneally injected with either vehicle or 10 mg/kg GW833972A daily for 5 days a week. Mice were euthanized when tumors in the control group reached a 2-cm diameter or when the tumor-induced skin breakage occurred in the vehicle control group. (**B**) Subcutaneous xenograft model in NUDE mice using ES-2 cells with or without 10 mg/kg GW833972A treatment. Five mice were used for each group, and data were shown as mean ± SD ($n = 5$). Nonlinear regression (curve fit) analysis was used to test for significant differences between the body weight curve of the vehicle control and GW833972A-treated group (*P* value is indicated). (**C**) Subcutaneous xenograft model in NUDE mice using JHOC5 cells with or without 10 mg/kg GW833972A treatment. Five mice were used for each group, and data were shown as mean ± SD ($n = 5$). Data formatting is as described for (**B**). (**D**) Representative images of hematoxylin and eosin (H&E) staining using liver, kidney, and spleen tissue sections from mice bearing ES-2-derived tumors. Scale bars indicate 200 μm. (**E**) Representative images of hematoxylin and eosin (H&E) staining using liver, kidney and spleen tissue sections from mice bearing JHOC5-derived tumors. Scale bars indicate 200 μm. (**F**) Representative images of hematoxylin and eosin (H&E) staining using liver, kidney, and spleen tissue sections from mice bearing ES-2 derived tumors treated with either vehicle, 10 mg/kg Olaparib, 10 mg/kg or 20 mg/kg GW833972A (GW), or GW833972A-Olaparib combination. Scale bars indicate 200 μm. Source data are available online for this figure.

© The Author(s)

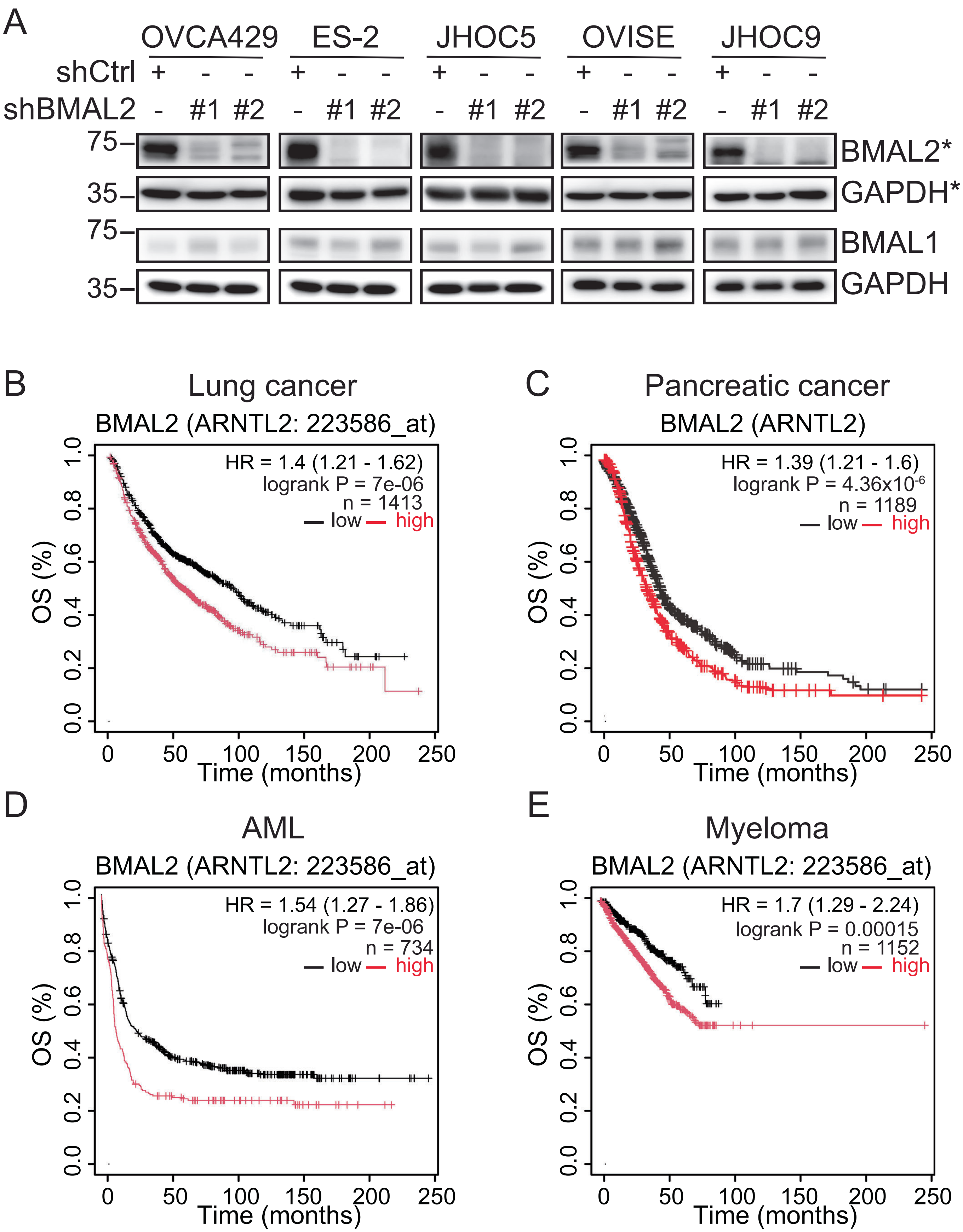
A
OVCA429
ES-2
JHOC5
OVISE
JHOC9
shCtrl + - -
shBMAL2 - #1 #2
75
35
BMAL2*
GAPDH*
BMAL1
GAPDH
B
Lung cancer
BMAL2 (ARNTL2: 223586_at)
HR = 1.4 (1.21 - 1.62)
logrank P = 7e-06
n = 1413
low high
OS (%)
Time (months)
C
Pancreatic cancer
BMAL2 (ARNTL2)
HR = 1.39 (1.21 - 1.6)
logrank P = 4.36x10-6
n = 1189
low high
D
AML
BMAL2 (ARNTL2: 223586_at)
HR = 1.54 (1.27 - 1.86)
logrank P = 7e-06
n = 734
low high
E
Myeloma
BMAL2 (ARNTL2: 223586_at)
HR = 1.7 (1.29 - 2.24)
logrank P = 0.00015
n = 1152
low high

◀ **Figure EV9. High BMAL2 expression is correlated with poor clinical outcomes in several cancer types other than OC.**

(**A**) Depletion of BMAML2 did not affect BMAL1 level in OCCC cells. IB of BMAL1 and BMAL2 protein levels with GAPDH as a loading control in OCCC cell lines without (shCtrl) or with BMAL2 depletion (shBMAL2#1 or #2). Blots shown are from one representative experiment of three replicates. *, BMAL2 blots presented here were from Fig. 2B. (**B–E**) Kaplan–Meier overall survival (OS) analysis of lung cancer (**B**), pancreatic cancer (**C**), AML (**D**), and myeloma (**E**) cancer patients grouped by BMAL2 expression. The BMAL2 high group is indicated by a red line and the BMAL2 low group is indicated by a black line. The *P* value was determined by the log-rank test. The results shown here are based upon data generated by the KM plotter (https://kmplot.com/analysis/). Source data are available online for this figure.

 © The Author(s)
